# Palladium-catalysed asymmetric cascade transformations of 4-alken-2-ynyl carbonates to construct complex frameworks†

**DOI:** 10.1039/d4sc07823c

**Published:** 2024-12-27

**Authors:** Ze-Liang He, Li Li, Zhi-Chao Chen, Wei Du, Ying-Chun Chen

**Affiliations:** a Key Laboratory of Drug-Targeting and Drug Delivery System of the Education Ministry and Sichuan Province, Sichuan Research Center for Drug Precision Industrial Technology, West China School of Pharmacy, Sichuan University Chengdu 610041 China chenzhichao@scu.edu.cn ycchen@scu.edu.cn +86 28 85502609; b College of Pharmacy, Third Military Medical University Shapingba Chongqing 400038 China

## Abstract

As a class of readily available and multifunctional building blocks, the chemistry of 4-alken-2-ynyl carbonates remains to be explored. Presented herein is a palladium-catalysed cascade transformative reaction between 4-alken-2-ynyl carbonates and *ortho*-functionalised activated alkenes. Achiral 1,1-bisalkyl-4-alken-2-ynyl carbonates undergo highly regioselective propargylic substitution with *ortho*-hydroxyphenyl-tethered activated alkenes, and an auto-tandem vinylogous addition, unusual central-carbon Tsuji–Trost alkylation, protonation and β-H elimination process is followed to furnish fused and spirocyclic frameworks with high structural complexity. Even kinetic transformations with racemic 1-monoalkylated 4-alken-2-ynyl carbonates can be accomplished in the assemblies with *ortho*-aminophenyl-tethered activated alkenes to afford the analogous alkaloid architectures. This palladium-catalysed auto-tandem protocol exhibits excellent chemo-, regio-, stereoselectivity and reaction efficacy, and substantial functionality compatibility is also observed.

## Abstract 2

A palladium-catalysed asymmetric auto-tandem reaction between 4-alken-2-ynyl carbonates and diverse ortho-functionalized activated alkenes is reported, giving complex frameworks in moderate yields with excellent chemo-, regio- and stereoselectivity.

## Graphical abstract

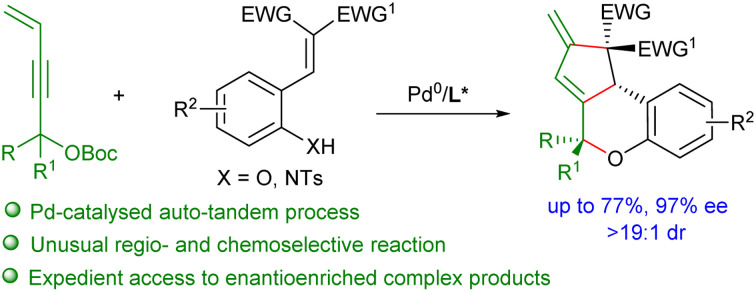

## Introduction

**Scheme 1:**
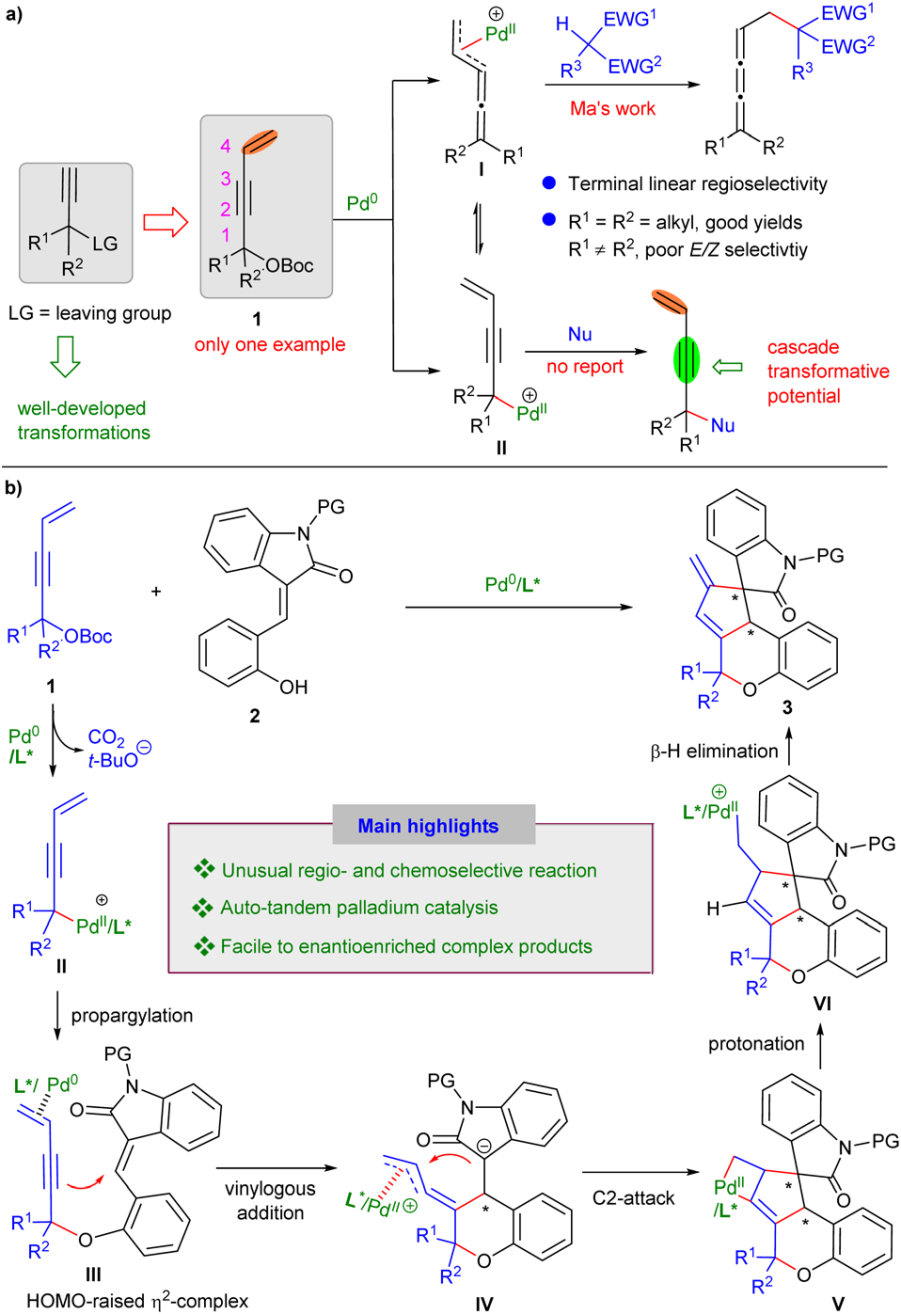
Typical reaction pathways of propargylic derivatives and our design for palladium-catalysed cascade transformations of 4-alken-2-ynyl carbonates. (a) Transformations from propargylic substrates to 4-alken-2-ynyl carbonates. (b) This work: Pd^0^-catalysed cascade transformations of 4-alken-2-ynyl carbonates.

Owing to their ready availability and versatile reactivity, propargylic substrates have emerged as valuable reagents in organic synthesis.^1^ Upon the oxidative addition of propargylic alcohol derivatives with transition metals, the formed allenyl or propargyl metal species can undergo a variety of transformations, such as allenylation,^2^ 1,3-dienylation,^3^ propargylation^4^ and others,^5^ to afford structurally diverse products, even enantioselectively. In addition, pre-installation of a pendent alkene moiety into propargylic skeleton, as in 4-alken-2-ynyl carbonates 1, would further enrich the transformative potential, since more reactive sites might be envisaged upon activation by transition metals. In this regard, Ma recently revealed that vinylidene-π-allyl palladium intermediates I, which were generated *in situ* from 1,1-bisalkyl-substituted 4-alken-2-ynyl carbonates 1 and Pd^0^, could undergo Tsuji−Trost-type reaction with stabilized carbon-centred nucleophiles to furnish achiral 1,2,3-butatrienes with exclusive linear selectivity.^6^ Unfortunately, the reactivity of racemic propargylic substrates (*R*^1^ ≠ *R*^2^) was not well investigated in this work, and poor *E*/*Z* selectivity was observed for the sole example. In addition, switching the regioselectivity of nucleophilic substitution to the more sterically hindered C1 position *via* potential species II is a formidable challenge, but would provide valuable opportunities in latent reaction design owing to the versatile features of the enyne products (Scheme 1a).^7^ Taking advantage of the multiple catalytic roles of palladium,^8^ here, we would like to present an unprecedented cascade transformative reaction between 4-alken-2-ynyl carbonates 1 and *ortho*-functionalised activated alkenes, such as 3-olefinic oxindoles 2. As outlined in Scheme 1b, the oxidative addition of Pd^0^ to carbonates 1 would generate propargylic palladium complexes II. In sharp contrast to Ma's work, exclusive C1-regioselective substitution with *O*-centered nucleophiles was observed to deliver multifunctional propargylated intermediates III. By employing our recently developed π-Lewis base catalysis,^9^ Pd^0^ further enhanced the nucleophilicity of the alkyne moiety by forming η^2^-complexes with increased highest occupied molecular orbital (HOMO) energy levels, thus facilitating intramolecular vinylogous addition to the 3-olefinic oxindole motif to produce ene-π-allyl-Pd intermediates IV. Intriguingly, an unusual nucleophilic attack on the central carbon of π-allylpalladium-type species occurred to afford palladacyclobutanes V.^10^ Subsequent protonation and β-H elimination delivered fused and spirocyclic architectures 3 with high molecular complexity.^3,11^ It is noteworthy that highly regio-, chemo- and enantioselective assemblies were achieved in this palladium-based auto-tandem catalysis.^12^

## Results and discussion

### Reaction optimisation

The reaction of 4-alken-2-ynyl carbonate 1a and (*E*)-3-(2-hydroxybenzylidene)oxindole 2a was initially examined in MeCN at 80 °C. The reaction proceeded smoothly in the presence of Pd(PPh_3_)_4_, and product 3a was obtained straightforwardly in a good yield through the proposed pathway as virtually a single diastereomer (Table 1, entry 1). Next, we turned our attention to the enantioselective synthesis of 3a. After a brief survey of various chiral ligands, we quickly found that bisphosphine ligands exhibited good catalytic performance for the current transformations.^13^ Fair enantioselectivity was observed for ligands L1 and L2 in combination with Pd_2_dba_3_ (entries 2 and 3), and ligand L3 having an anthracene diamine backbone substantially boosted both the efficiency and enantiocontrol (entry 4). Lowering the temperature enhanced the enantioselectivity, although a longer time was required to achieve better conversions (entry 5). A solvent survey suggested enantiocontrol was slightly improved in polar solvents (entries 6–8), and a higher yield was obtained in diluted DMA (entry 9). Then, several additives were screened,^13^ and KHCO_3_ was found to be beneficial to the yield (entry 10). A satisfactory yield with an excellent ee value was finally obtained by employing 1.5 equivalents of carbonate 1a at 45 °C (entry 11), whereas the yield was reduced significantly when using 2.5 mol% of Pd_2_dba_3_ (entry 12).

**Table 1:** Screening conditions for the asymmetric auto-tandem reaction^a^

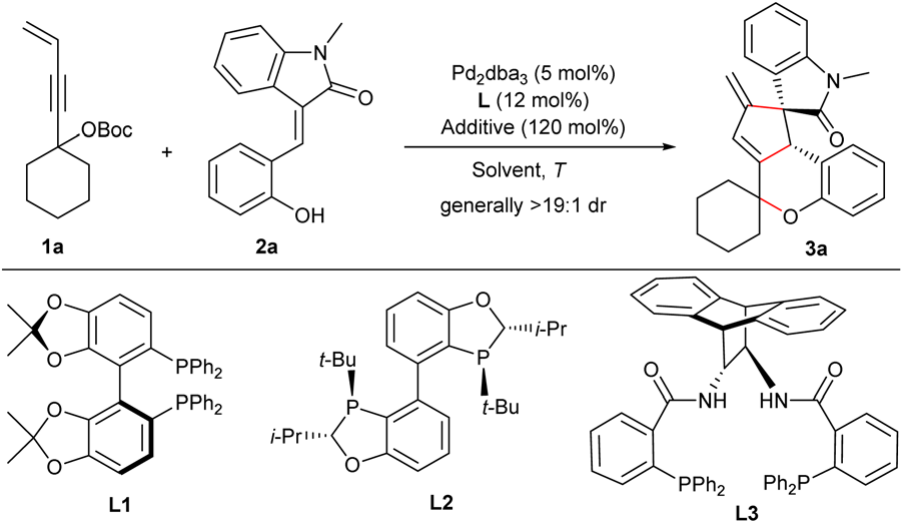							
Entry	L	Solvent	*T* (°C)	*t* (h)	Additive	Yield^b^ (%)	ee^c^ (%)
1^d^	—	MeCN	80	36	—	78	—
2	L1	MeCN	80	24	—	56	29
3	L2	MeCN	80	24	—	33	58
4	L3	MeCN	80	24	—	63	74
5	L3	MeCN	50	48	—	63	85
6	L3	NMP	50	48	—	58	87
7	L3	DMF	50	48	—	62	85
8	L3	DMA	50	48	—	57	88
9^e^	L3	DMA	50	48	—	60	88
10^e^	L3	DMA	50	48	KHCO_3_	73	89
11^e^^,^^f^	L3	DMA	45	60	KHCO_3_	72	91
12^e^^,^^f^^,^^g^	L3	DMA	45	60	KHCO_3_	48	91

a Unless noted otherwise, reactions were performed with carbonate 1a (0.1 mmol), alkene 2a (0.12 mmol), Pd_2_dba_3_ (5 mol%), ligand L (12 mol%) and additive (1.2 equiv.) in degassed solvent (1.0 mL) under Ar.

b Yield of the isolated product.

c Determined by HPLC analysis on a chiral stationary phase, and >19 : 1 dr was generally obtained through ^1^H NMR analysis.

d With Pd(PPh_3_)_4_ (5 mol%).

e In DMA (2.0 mL).

f With 1a (0.15 mmol) and 2a (0.1 mmol).

g With Pd_2_dba_3_ (2.5 mol%) and L3 (6 mol%).

### Substrate scope and limitations

**Scheme 2:**
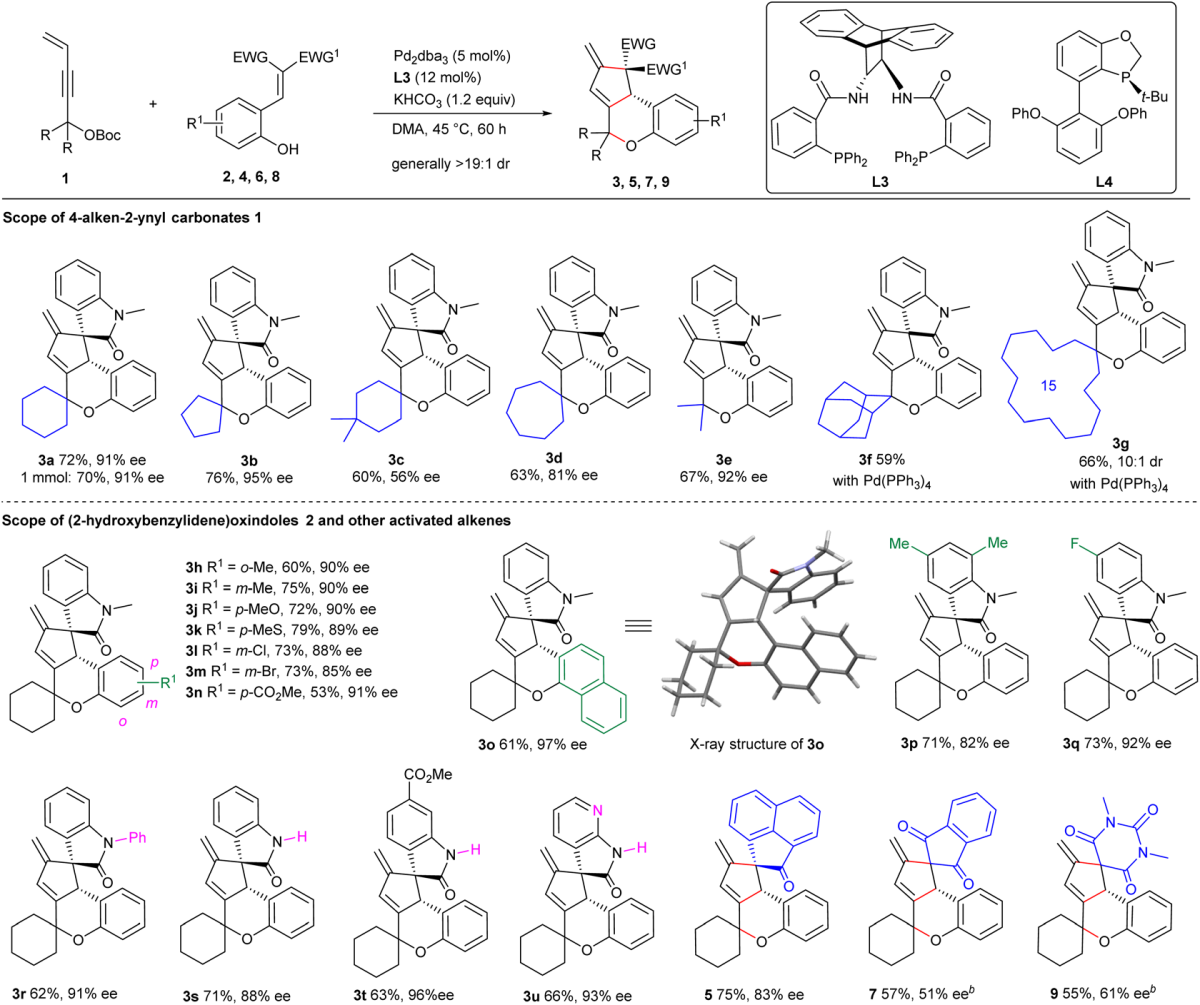
Substrate scope of asymmetric auto-tandem reaction of achiral 4-alken-2-ynyl carbonates 1 and diverse activated alkenes.^*a*^Unless noted otherwise, reactions were performed with carbonate 1 (0.15 mmol), activated alkene (0.1 mmol), Pd_2_dba_3_ (5 mol%), L3 (12 mol%) and KHCO_3_ (1.2 equiv.) in degassed dry DMA (2.0 mL) at 45 °C for 60 h under Ar; yields refer to the isolated product; dr was determined by ^1^H NMR analysis of the crude product; ee was determined by HPLC analysis on a chiral stationary phase. ^*b*^With L4 (20 mol%) in toluene (1.0 mL) at 80 °C for 24 h.

Under the optimised conditions, the scope of the 3-vinyl propargylic carbonates 1 was first explored in the reactions with (*E*)-3-(2-hydroxybenzylidene) oxindole 2a under the catalysis of Pd_2_dba_3_/L3 with KHCO_3_ as an additive. As summarised in Scheme 2, an array of carbonates 1 derived from different cyclic ketones reacted smoothly with 2a, generally affording the corresponding products 3a–3d in moderate yields with remarkable diastereo- and enantioselectivity, even in a 1.0 mmol scale reaction (product 3a), whereas a moderate ee value was observed for product 3c bearing a 4,4-dimethylcyclohexane moiety. Pleasingly, product 3e having *gem*-dimethyl groups was furnished with comparably good results. Unfortunately, no apparent conversion was observed when using propargylic carbonates bearing an admantyl or cyclopentadecyl group, while racemic 3f and 3g could be obtained in moderate yields catalysed by Pd(PPh_3_)_4_. Next, the scope of functionalized alkenes 2 was evaluated (Scheme 2). Substrates with a broad range of electron-donating and -withdrawing groups at various positions of the phenyl or oxindole unit were well tolerated, generally affording the desired products 3h–3q in moderate yields with high levels of enantioselectivity. Notably, similarly good data were obtained for (7-aza)oxindoles 2 having an *N*-phenyl or even a free NH group (products 3r–3u).

In addition to 3-olefinic oxindoles 2, activated alkenes derived from other skeletons were also applicable. As outlined in Scheme 2, benzylideneacenaphthenone 4 was successfully assembled with carbonate 1a to deliver 5 in good yield and enantioselectivity under the standard conditions. Moreover, both activated alkenes 6 and 8 condensed from 1,3-indandione and barbituric acid with salicylaldehyde, respectively, proved to be reliable counterparts in the reactions with 1a under the catalysis of Pd_2_dba_3_/L4, albeit with moderate enantiocontrol (products 7 and 9). These results not only showcased the robustness of the current catalytic strategy, but also enriched the structural diversity of the frameworks constructed.

**Scheme 3:**
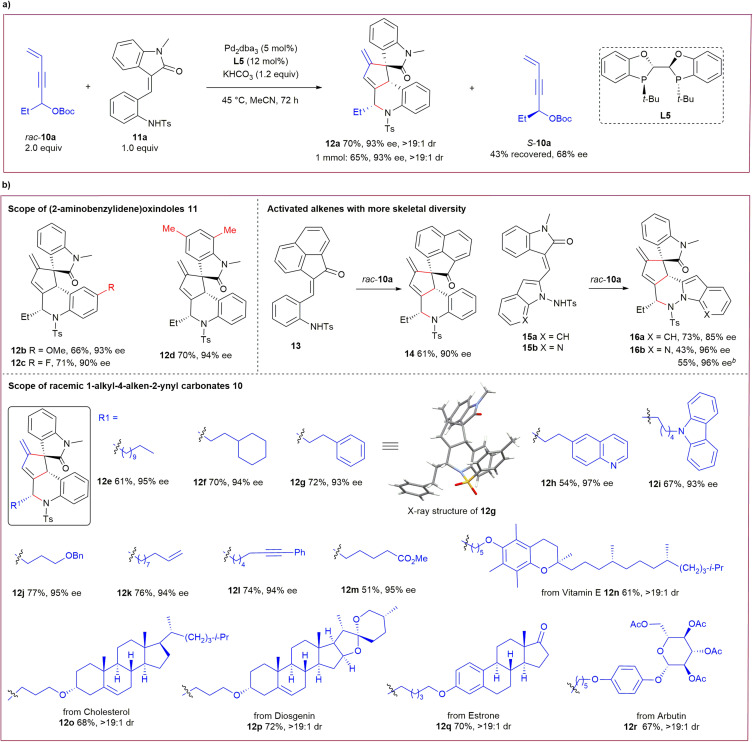
Substrate scope for kinetic transformations of racemic 1-alkyl-substituted 4-alken-2-ynyl carbonates 10.^*a*^(a) Kinetic transformations of racemic 1-alkyl-4-alken-2-ynyl carbonates *via* auto-tandem catalysis. (b) Substrate scope investigation.^*a*^Unless noted otherwise, reactions were performed with racemic carbonate 10 (0.2 mmol), activated alkene (0.1 mmol), Pd_2_dba_3_ (5 mol%), L5 (12 mol%) and KHCO_3_ (1.2 equiv.) in degassed MeCN (1.0 mL) at 45 °C for 72 h under Ar; ^*b*^With *rac*-10a (0.3 mmol).

The successful cascade transformations of achiral 1,1-bisalkyl-substituted 4-alken-2-ynyl carbonates 1 inspired us to investigate the potential application of more challenging racemic propargylic carbonates. As illustrated in Scheme 3a, gratifyingly, 1-ethyl carbonate *rac*-10a (2 equiv.) could be efficiently utilised in similar asymmetric cascade transformations with (2-aminobenzylidene) oxindole 11a catalysed by Pd_2_dba_3_ and ligand L5, furnishing an analogous alkaloid architecture 12a in a moderate yield with excellent stereoselectivity, even on a larger scale. In addition, simultaneous kinetic resolution for recovered *rac*-10a was observed, albeit with moderate enantioselectivity.^14^

The substrate scope for this type of kinetic transformation is substantial. As summarised in Scheme 3b, good yields and high enantioselectivity were uniformly obtained for activated alkenes 11 with different substituents on the aryl unit under the standard catalytic conditions (products 12b–12d). Activated alkene 13 and newly designed 15 smoothly participated in the reactions with *rac*-10a to produce complex frameworks 14 and 16a–16b with high efficiency. Importantly, a spectrum of racemic propargylic carbonates 10 with diverse 1-alkyl substitutions, including those bearing various functionalities, were compatible in the reactions with activated alkene 11a, yielding products 12e–12m with high enantiocontrol. This method also provides an efficient tool for the late-stage modification of bioactive molecules. A broad range of chiral drugs (or their fragments) containing a 3-vinyl motif were competent in this process, which led to the complex products 12n–12r in moderate yields with excellent diastereoselectivity.

**Scheme 4:**
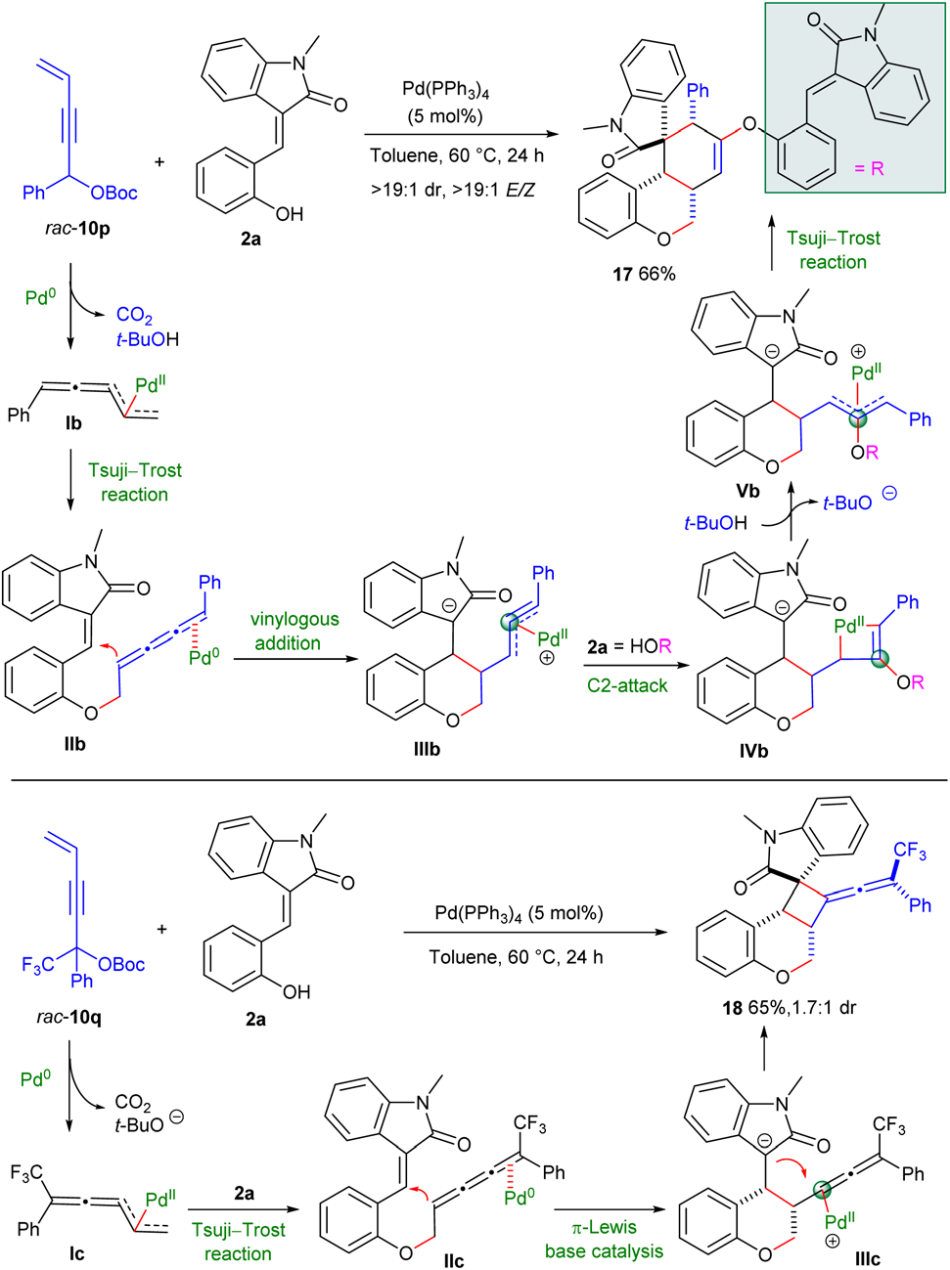
Divergent transformations of 1-aryl-4-alken-2-ynyl carbonates.

Of particular note, 1-phenyl-substituted carbonate *rac*-10p underwent *O*-allylic alkylation with (*E*)-3-(2-hydroxybenzylidene) oxindole 2a with distinct terminal regioselectivity under the catalysis of Pd(PPh_3_)_4_,^6^ probably resulting from the generation of thermally more stable 1-phenyl allenyl-π-allyl species Ib. The formed 1,2,3-butatriene intermediate IIb could be similarly HOMO-activated by Pd(0) *via* a π-Lewis base pattern, thus facilitating intramolecular vinylogous addition to the 3-olefinic oxindole motif. Interestingly, the resultant η^3^-propargylpalladium species IIIb was C2-attacked by another molecule of 2a, delivering palladacyclobutene intermediate IVb. Subsequent protonation and intramolecular Tsuji–Trost reaction of Vb would furnish spirocyclic product 17 with high diastereo- and *E*/*Z*-selectivity (Scheme 4).^5^ Moreover, tertiary propargylic carbonate *rac*-10q was found to undergo similar Tsuji–Trost reaction/vinylogous addition with 2a efficiently through intermediates Ic and IIc, respectively, whereas the formed η^1^-allenylpalladium moiety of IIIc was intramolecularly captured by enolate to provide intriguing cyclobutane-fused chromanone 18 having a tetrasubstituted exocyclic allene motif in a moderate yield with fair diastereoselectivity.^2*g*–*i*^ Although the asymmetric variants were not applicable at the current stage, these explorations clearly demonstrated the versatile reactivity of 4-alken-2-ynyl carbonates and significantly enriched the product diversity.

### Synthetic transformations

**Scheme 5:**
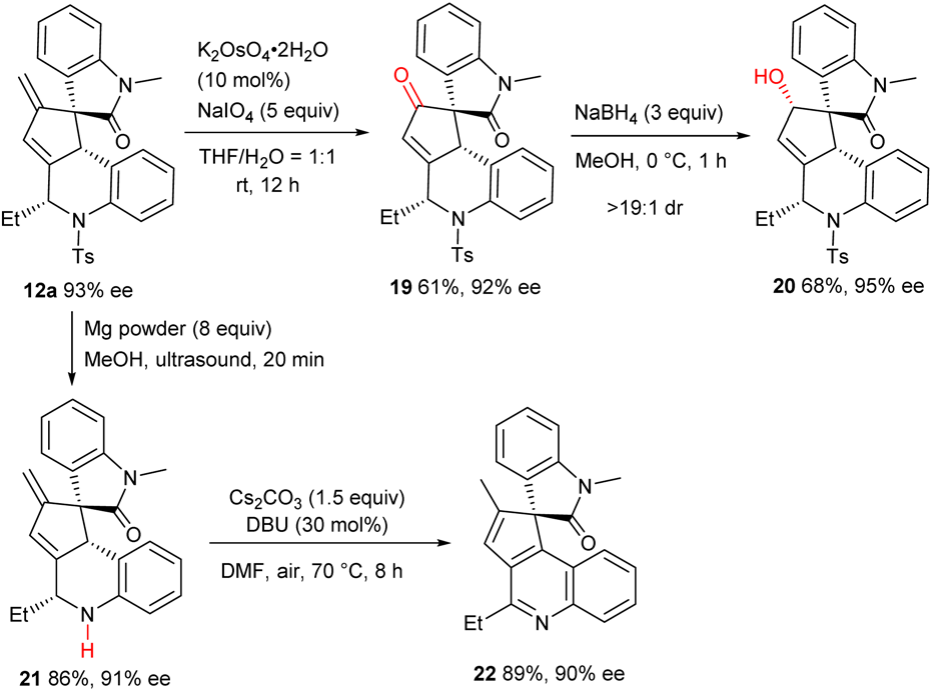
Transformations of product 12a.

Further transformations successfully exemplified the synthetic utility of the multifunctional products. As depicted in Scheme 5, osmium-catalysed oxidative cleavage of the terminal olefin moiety of 12a afforded ketone 19 in a moderate yield, which was further reduced to alcohol 20 with exclusive diastereoselectivity. Additionally, the *N*-Ts group was efficiently removed to deliver amine 21, and fused quinoline 22 was obtained in high yield *via* a base-promoted isomerization/oxidative aromatization process. It should be noted that the spirooxindole-fused polycycles and their derivatives are core subunits of many bioactive natural products and drug candidates.^15^

## Conclusions

In summary, we successfully developed a cascade transformative reaction between 4-alken-2-ynyl carbonates and *ortho*-functionalized activated alkenes under auto-tandem palladium catalysis. This process showed high regio-, chemo-, and stereoselectivity through integrating classical palladium-mediated nucleophilic substitution with our newly uncovered π-Lewis base catalysis into a one-pot fashion. Both achiral 1,1-bisalkyl- and racemic 1-alkyl-4-alken-2-ynyl carbonates with broad substitution patterns underwent C1-selective propargylation to afford enyne intermediates, and fused and spirocyclic frameworks with high structural complexity were finally furnished enantioselectively upon tandem palladium catalysis, which proceeded *via* a cascade vinylogous addition, unusual central carbon Tsuji–Trost alkylation, protonation and β-H elimination process. In contrast, 1-aryl-substituted 4-alken-2-ynyl carbonates favoured terminal substitution to generate 1,2,3-butatriene intermediates, and regiodivergent transformations gave distinct polycyclic architectures, albeit in a racemic pattern. This work exhibits the versatile reaction potential of multifunctional 4-alken-2-ynyl carbonates, which offer a platform to construct structurally complex compounds *via* auto-tandem catalysis. More results will be reported in due course.

## Data availability

The data that support the findings of this study are available in the ESI† or on request from the corresponding author.

## Author contributions

The manuscript was written through contributions of all authors. All authors have given approval to the final version of the manuscript.

## Conflicts of interest

There are no conflicts to declare.

## Supplementary Material

SC-OLF-D4SC07823C-s001

SC-OLF-D4SC07823C-s002
