# Supplementary material for: Palladium-catalysed asymmetric cascade transformations of 4-alken-2-ynyl carbonates to construct complex frameworks
Source: Chem Sci. 2024 Dec 27;16(7):3124–9. doi: 10.1039/d4sc07823c (PMC11737346; doi:10.1039/d4sc07823c)
Supplement: SC-016-D4SC07823C-s001 [file SC-016-D4SC07823C-s001.pdf]

Supplementary Information (SI) for Chemical Science.
This journal is © The Royal Society of Chemistry 2025

Palladium-catalysed asymmetric cascade transformations of 4-alken-2-ynyl carbonates to construct complex frameworks

Ze-Liang He,[a] Li Li,[a] Zhi-Chao Chen,*[a] Wei Du,[a] and Ying-Chun Chen*[ab]

[a] Key Laboratory of Drug-Targeting and Drug Delivery System of the Ministry of Education and Sichuan Research Center for Drug Precision Industrial Technology, West China School of Pharmacy, Sichuan University, Chengdu 610041, China

[b] College of Pharmacy, Third Military Medical University, Shapingba, Chongqing 400038, China

Email: chenzhichao@scu.edu.cn; ycchen@scu.edu.cn

Supplementary Information

1. General methods **S1**

2. Preparation and characterisation of substrates **S2**

3. Detailed condition optimisations **S12**

4. General procedure for the reactions of achiral 4-alken-2-ynyl carbonates **S15**

4.1 General procedure for synthesis of 3 and 5 .... **S15**

4.2 Procedure for asmmytric synthesis of 7 and 9 .... **S31**

5 General procedure for reactions of racemic 1-alkyl-substituted 4-aken-2-ynyl carbonates 10 .... **S32**

6. Transformations of 1-aryl-4-alken-2-ynyl carbonates **S49**

7. Unsuccessful substrates exploration **S52**

8. Transformations of product 12a **S53**

9. Preliminary mechanism investigation **S56**

10. Crystal data and structural refinement **S58**

11. NMR, HRMS spectra and HPLC chromatograms **S65**

1. General methods

Unless otherwise noted, the reactions were carried out under ambient atmosphere; when the reactions required heating, the heat source was oil bath. ^{1}H NMR (400 or 600 MHz), ^{13}C NMR (100 or 150 MHz) and ^{19}F NMR (375 MHz) spectra were recorded on Varian INOVA-400/54, Agilent DD2-600/54 or Bruker Ascend^{TM} 400 instruments (Chemical shifts were reported in ppm from tetramethylsilane with the solvent resonance as the internal standard in $CDCl_3$ solution, unless otherwise noted). The following abbreviations were used to explain the multiplicities: s = singlet, d = doublet, t = triplet, q = quartet, dd = double doublet, m = multiplet and coupling constants (*J*) are reported in Hertz (Hz). High resolution mass spectra (HRMS) were recorded on a Waters SYNAPT G2 or Agilent G1969-85000 using a time-of-flight mass spectrometer equipped with electrospray ionization (ESI) source. X-ray diffraction experiments were carried out on an Agilent Gemini or Burker D8 Venture and the data obtained were deposited at the Cambridge Crystallographic Data Centre (CCDC 2381131–2381135). In each case, diastereomeric ratio was determined by ^{1}H NMR analysis and enantiomeric excess was determined by HPLC (Agilent Technologies: 1220 Infinity II, 1200 Series, 1260 Infinity) analysis on a chiral column in comparison with authentic racemate, using a Daicel Chiralpak AD-H Column (250 × 4.6 mm), Chiralpak IA Column (250 × 4.6 mm), Chiralpak IB Column (250 × 4.6mm), IBN-5 Column (250 × 4.6 mm), Chiralpak ID Column (250 × 4.6 mm), Chiralpak IE Column(250 × 4.6 mm), Chiralpak IF Column (250 × 4.6 mm). UV detection was monitored at 220 nm or 254 nm. Optical rotation was measured in $CHCl_3$ solution at 25 °C. Column chromatography was performed on silica gel (200–300 mesh) eluting with EtOAc and petroleum ether. TLC was performed on glass-backed silica plates. UV light, I_2, and solution of potassium permanganate were used to visualize products or starting materials. All chemicals were used without purification as commercially available unless otherwise noted. Petroleum ether and EtOAc were re-distilled. Experiments involving moisture and/or air sensitive components were performed under a positive pressure of argon in oven-dried glassware equipped with a rubber septum inlet. Toluene was freshly distilled from CaH_2 under an atmosphere of dry argon. Dried solvents reagents were transferred by oven-dried syringe.

2. Preparation and characterization of substrates

2.1 General procedure for the preparation of 4-alken-2-ynyl carbonates[1]

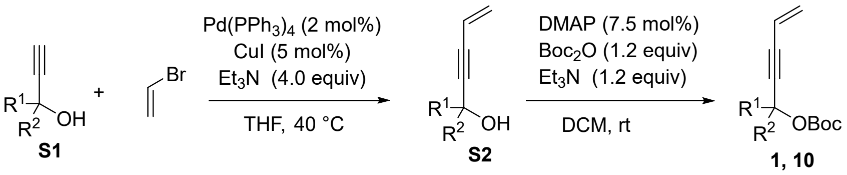

To an oven-dried flask were added CuI (57 mg, 0.3 mmol) and $Pd(PPh_3)_4$ (346 mg, 0.3 mmol). The flask was capped, purged and back-filled with argon for five times. Dry THF (20 mL), propargylic alcohol **S1** (10 mmol), vinyl bromide (1.0 M in THF, 15 mL, 15 mmol) and triethylamine (5 mL, 40 mmol) were added sequentially via syringe. The mixture was stirred at 40 °C until complete consumption of **S1**. The mixture was cooled to room temperature and filtered. The filtrate was concentrated under reduced pressure, and the residue was purified by flash column chromatography to give **S2**.

To a solution of **S2** (10 mmol) in DCM (30 mL) were added triethylamine (1.7 mL, 12 mmol) and Boc_2O (2.6 g, 12 mmol) followed by DMAP (92 mg, 0.75 mmol). The mixture was stirred at room temperature overnight and then poured into water. The phases were separated and the aqueous phase was extracted three times with DCM. The combined organic phases were washed with brine, dried over anhydrous Na_2SO_4, filtered and the filtrate was concentrated and the residue was purified by flash column chromatography to give substrate **1** or **10**.

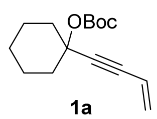

1a

1-(But-3-en-1-yn-1-yl)cyclohexyl *tert*-butyl carbonate (1a): eluent (petroleum ether/ethyl acetate = 75/1); colourless oil; 71% yield; ^{1}H NMR (400 MHz, $CDCl_3$): δ (ppm) 5.86 (dd, *J* = 17.6, 11.2 Hz, 1H), 5.64 (dd, *J* = 17.6, 2.4 Hz, 1H), 5.48 (dd, *J* = 11.2, 2.0 Hz, 1H), 2.24–2.14 (m, 2H), 1.89–1.78 (m, 2H), 1.73–1.55 (m, 5H), 1.49 (s, 9H), 1.34–1.25 (m, 1H); ^{13}C NMR (100 MHz, $CDCl_3$): δ (ppm) 151.3, 127.1, 116.8, 89.4, 85.1, 81.9, 37.1, 27.8, 25.2, 22.8; HRMS (ESI-TOF) *m/z*: $[M + Na]^+$ Calcd for $C_{15}H_{22}O_3Na^+$ 273.1461; Found 273.1459.

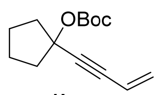

1b

1-(But-3-en-1-yn-1-yl)cyclopentyl *tert*-butyl carbonate (1b): eluent (petroleum ether/ethyl acetate = 75/1); colorless oil; 72% yield; ^{1}H NMR (400 MHz, $CDCl_3$): δ (ppm) 5.89–5.77 (m, 1H), 5.61 (dd, *J* = 17.6, 2.0 Hz, 1H), 5.46 (dd, *J* = 11.2, 2.4 Hz, 1H), 2.32–2.25 (m, 2H), 2.17–2.09 (m, 2H), 1.81–1.72 (m, 4H), 1.50 (s, 9H); ^{13}C NMR (100 MHz, $CDCl_3$): δ (ppm) 151.8, 127.1, 116.8, 90.0, 83.4, 82.5, 82.1, 40.5, 27.8, 23.4; HRMS (ESI-TOF) *m/z*:

[M + Na]+ Calcd for $C_{14}H_{20}O_3Na^+$ 259.1305; Found 259.1304.

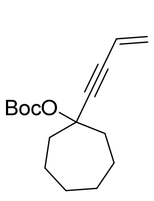

1d

1-(But-3-en-1-yn-1-yl)cycloheptyl *tert*-butyl carbonate (1d): eluent (petroleum ether/ethyl acetate = 70/1); colourless oil; 66% yield; ^{1}H NMR (400 MHz, $CDCl_3$): δ (ppm) 5.85 (dd, J = 17.6, 11.2 Hz, 1H), 5.63 (dd, J = 17.6, 2.0 Hz, 1H), 5.47 (dd, J = 11.2, 2.0 Hz, 1H), 2.31–2.24 (m, 2H), 2.15–2.06 (m, 2H), 1.66–1.55 (m, 8H), 1.49 (s, 9H); ^{13}C NMR (100 MHz, $CDCl_3$): δ (ppm) 151.4, 127.1, 116.9, 90.6, 84.2, 81.9, 80.7, 40.1, 28.2, 27.9, 22.2; HRMS (ESI-TOF) *m/z*: [M + Na]+ Calcd for $C_{16}H_{24}O_3Na^+$ 287.1618; Found 287.1624.

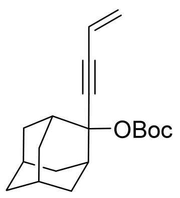

1f

2-(But-3-en-1-yn-1-yl)adamantan-2-yl *tert*-butyl carbonate (1f): eluent (petroleum ether/ethyl acetate = 75/1); colourless oil; 65% yield; ^{1}H NMR (400 MHz, $CDCl_3$): δ (ppm) 5.86 (dd, J = 17.6, 11.2 Hz, 1H), 5.62 (dd, J = 17.6, 2.4 Hz, 1H), 5.46 (dd, J = 11.2, 2.4 Hz, 1H), 2.50 (s, 2H), 2.17 (d, J = 12.4 Hz, 2H), 2.06 (d, J = 12.0 Hz, 2H), 1.83–1.70 (m, 6H), 1.63–1.57 (m, 2H), 1.50 (s, 9H); ^{13}C NMR (100 MHz, $CDCl_3$): δ (ppm) 151.4, 126.9, 116.9, 90.0, 85.6, 81.7, 81.6, 37.5, 36.1, 35.0, 31.9, 27.9, 26.8, 26.3; HRMS (ESI-TOF) *m/z*: [M + Na]+ Calcd for $C_{19}H_{26}O_3Na^+$ 325.1774; Found 325.1771.

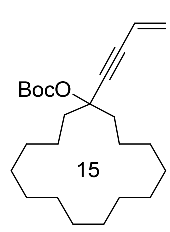

1g

1-(But-3-en-1-yn-1-yl)cyclopentadecyl *tert*-butyl carbonate (1g): eluent (petroleum ether/ethyl acetate = 70/1); colourless oil; 66% yield; ^{1}H NMR (400 MHz, $CDCl_3$): δ (ppm) 5.84 (dd, J = 17.6, 11.2 Hz, 1H), 5.62 (dd, J = 17.6, 2.0 Hz, 1H), 5.46 (dd, J = 11.2, 2.0 Hz, 1H), 2.12–2.02 (m, 2H), 1.95–1.81 (m, 2H), 1.49 (s, 9H), 1.45–1.21 (m, 24H); ^{13}C NMR (100 MHz, $CDCl_3$): δ (ppm) 150.3, 126.1, 115.8, 89.0, 83.3, 80.8, 78.8, 35.5, 26.8, 26.2, 25.9, 25.6, 25.4, 20.7; HRMS (ESI-TOF) *m/z*: [M + Na]+ Calcd for $C_{24}H_{40}O_3Na^+$ 399.2870; Found 399.2870.

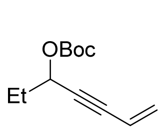

10a

***tert*-Butyl hept-6-en-4-yn-3-yl carbonate (10a):** eluent (petroleum ether/ethyl acetate = 75/1); colourless oil; 69% yield; ^{1}H NMR (400 MHz, $CDCl_3$): δ (ppm) 5.87–5.74 (m, 1H), 5.66 (dd, J = 17.6, 2.4 Hz, 1H), 5.50 (dd, J = 11.2, 2.4 Hz, 1H), 5.25 (t, J = 6.4 Hz, 1H), 1.88–1.80 (m, 2H), 1.50 (s, 9H), 1.03 (t, J = 7.6 Hz, 3H); ^{13}C NMR (150 MHz, $CDCl_3$): δ (ppm) 152.7, 127.8, 116.5, 86.6, 84.4, 82.6, 68.5, 28.2, 27.7, 9.4; HRMS (ESI-TOF) *m/z*: [M + Na]+ Calcd for $C_{12}H_{18}O_3Na^+$ 233.1148; Found 233.1141.

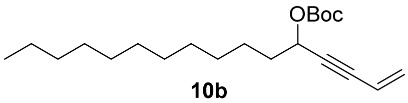

10b

***tert*-Butyl hexadec-1-en-3-yn-5-yl carbonate (10b):** eluent (petroleum ether/ethyl acetate = 80/1); colourless oil; 64% yield; ^{1}H NMR (400 MHz, $CDCl_3$): δ (ppm) 5.87–5.75 (m, 1H), 5.65 (dd, *J* = 17.6, 2.4 Hz, 1H), 5.49 (dd, *J* = 11.2, 2.4 Hz, 1H), 5.34–5.23 (m, 1H), 1.86–1.75 (m, 2H), 1.50 (s, 9H), 1.47–1.41 (m, 2H), 1.30–1.21 (m, 16H), 0.88 (t, *J* = 6.8 Hz, 3H); ^{13}C NMR (150 MHz, $CDCl_3$): δ (ppm) 152.7, 127.8, 116.5, 87.0, 84.3, 82.6, 67.5, 34.9, 31.9, 29.6, 29.52, 29.51, 29.45, 29.3, 29.1, 27.8, 25.0, 22.7, 14.1; HRMS (ESI-TOF) *m/z*: $[M + Na]^+$ Calcd for $C_{21}H_{36}O_3Na^+$ 359.2557; Found 359.2563.

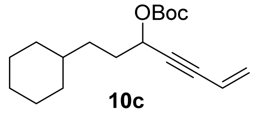

10c

***tert*-Butyl (1-cyclohexylhept-6-en-4-yn-3-yl) carbonate (10c):** eluent (petroleum ether/ethyl acetate = 75/1); colourless oil; 76% yield; ^{1}H NMR (400 MHz, $CDCl_3$): δ (ppm) 5.86–5.76 (m, 1H), 5.65 (dd, *J* = 17.6, 2.4 Hz, 1H), 5.50 (dd, *J* = 11.6, 2.4 Hz, 1H), 5.26 (t, *J* = 6.8 Hz, 1H), 1.85–1.77 (m, 2H), 1.74–1.64 (m, 5H), 1.50 (s, 9H), 1.37–1.30 (m, 2H), 1.27–1.11 (m, 4H), 0.95–0.84 (m, 2H); ^{13}C NMR (100 MHz, $CDCl_3$): δ (ppm) 152.7, 127.8, 116.5, 87.0, 84.4, 82.6, 67.8, 37.3, 33.3, 33.2, 32.5, 32.4, 27.8, 26.6, 26.3; HRMS (ESI-TOF) *m/z*: $[M + Na]^+$ Calcd for $C_{18}H_{28}O_3Na^+$ 315.1931; Found 315.1936.

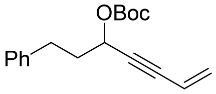

10d

***tert*-Butyl (1-phenylhept-6-en-4-yn-3-yl) carbonate (10d):** eluent (petroleum ether/ethyl acetate = 70/1); colourless oil; 73% yield; ^{1}H NMR (400 MHz, $CDCl_3$): δ (ppm) 7.31–7.26 (m, 2H), 7.22–7.17 (m, 3H), 5.89–5.77 (m, 1H), 5.67 (dd, *J* = 17.6, 2.4 Hz, 1H), 5.52 (dd, *J* = 11.2, 2.4 Hz, 1H), 5.31–5.26 (m, 1H), 2.80 (t, *J* = 8.0 Hz, 2H), 2.20–2.08 (m, 2H), 1.50 (s, 9H); ^{13}C NMR (100 MHz, $CDCl_3$): δ (ppm) 152.6, 140.7, 128.5, 128.4, 128.0, 126.1, 116.4, 86.5, 84.8, 82.7, 66.8, 36.4, 31.2, 27.8; HRMS (ESI-TOF) *m/z*: $[M + Na]^+$ Calcd for $C_{18}H_{22}O_3Na^+$ 309.1461; Found 309.1467.

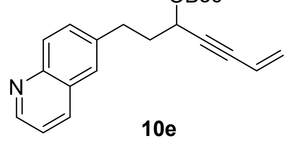

10e

***tert*-Butyl (1-(quinolin-6-yl)hept-6-en-4-yn-3-yl) carbonate (10e):** eluent (petroleum ether/ethyl acetate = 12/1); colourless oil; 62% yield; ^{1}H NMR (400 MHz, $CDCl_3$): δ (ppm) 8.91–8.82 (m, 1H), 8.11–8.01 (m, 2H), 7.63–7.56 (m, 2H), 7.40–7.34 (m, 1H), 5.90–5.77 (m, 1H), 5.72–5.64 (m, 1H), 5.56–5.49 (m, 1H), 5.37–5.31 (m, 1H), 3.00 (t, *J* = 7.6 Hz, 2H), 2.30–2.19 (m, 2H), 1.50 (s, 9H); ^{13}C NMR (100 MHz, $CDCl_3$): δ (ppm) 152.6, 149.9, 147.2, 139.2, 135.6, 130.8, 129.6, 128.3, 128.2,

126.3, 121.2, 116.4, 86.4, 85.0, 82.8, 66.7, 36.1, 31.2, 27.8; HRMS (ESI-TOF) *m/z*: $[M + H]^+$ Calcd for $C_{21}H_{24}NO_3^+$ 338.1751; Found 338.1747.

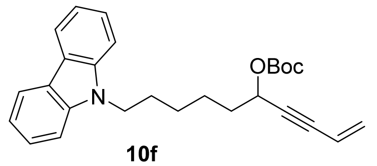

10f

10-(9*H*-carbazol-9-yl)dec-1-en-3-yn-5-yl *tert*-butyl carbonate (10f): eluent (petroleum ether/ethyl acetate = 15/1); colourless oil; 68% yield; ^{1}H NMR (400 MHz, $CDCl_3$): δ (ppm) 8.10 (d, *J* = 7.6 Hz, 2H), 7.48–7.44 (m, 2H), 7.41–7.37 (m, 2H), 7.24–7.20 (m, 2H), 5.84–5.73 (m, 1H), 5.64 (dd, *J* = 17.6, 2.0 Hz, 1H), 5.49 (dd, *J* = 10.8, 2.4 Hz, 1H), 5.27 (t, *J* = 6.4 Hz, 1H), 4.30 (t, *J* = 7.2 Hz, 2H), 1.93–1.85 (m, 2H), 1.82–1.73 (m, 2H), 1.55–1.50 (m, 2H), 1.48 (s, 9H), 1.46–1.40 (m, 2H); ^{13}C NMR (100 MHz, $CDCl_3$): δ (ppm) 152.7, 140.4, 128.0, 125.6, 122.8, 120.4, 118.8, 116.4, 108.6, 86.7, 84.6, 82.7, 67.2, 42.9, 34.7, 28.8, 27.8, 26.8, 24.7; HRMS (ESI-TOF) *m/z*: $[M + Na]^+$ Calcd for $C_{27}H_{31}NO_3Na^+$ 440.2196; Found 440.2198.

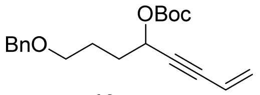

10g

1-(Benzyloxy)oct-7-en-5-yn-4-yl *tert*-butyl carbonate (10g): eluent (petroleum ether/ethyl acetate = 70/1); colourless oil; 75% yield; ^{1}H NMR (400 MHz, $CDCl_3$): δ (ppm) 7.37–7.26 (m, 5H), 5.85–5.72 (m, 1H), 5.65 (dd, *J* = 17.6, 2.0 Hz, 1H), 5.49 (dd, *J* = 11.2, 2.4 Hz, 1H), 5.36–5.31 (m, 1H), 4.50 (s, 2H), 3.52 (t, *J* = 6.4 Hz, 2H), 1.96–1.89 (m, 2H), 1.83–1.76 (m, 2H), 1.49 (s, 9H); ^{13}C NMR (100 MHz, $CDCl_3$): δ (ppm) 152.7, 138.5, 128.4, 127.9, 127.6, 127.6, 116.5, 86.7, 84.6, 82.7, 72.9, 69.6, 67.2, 31.8, 27.8, 25.3; HRMS (ESI-TOF) *m/z*: $[M + Na]^+$ Calcd for $C_{20}H_{26}O_4Na^+$ 353.1723; Found 353.1719.

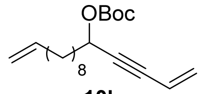

10h

***tert*-Butyl pentadeca-1,14-dien-3-yn-5-yl carbonate (10h):** eluent (petroleum ether/ethyl acetate = 80/1); colourless oil; 67% yield; ^{1}H NMR (400 MHz, $CDCl_3$): δ (ppm) 5.92–5.74 (m, 2H), 5.72–5.60 (m, 1H), 5.55–5.45 (m, 1H), 5.28 (t, *J* = 6.4 Hz, 1H), 5.06–4.86 (m, 2H), 2.10–1.98 (m, 2H), 1.87–1.74 (m, 2H), 1.50 (s, 9H), 1.46–1.25 (m, 12H); ^{13}C NMR (100 MHz, $CDCl_3$) δ (ppm) 152.7, 139.2, 127.8, 116.5, 114.1, 87.0, 84.3, 82.6, 67.5, 34.9, 33.8, 29.4, 29.3, 29.1, 28.9, 27.8, 25.0; HRMS (ESI-TOF): *m/z*: $[M + Na]^+$ Calcd for $C_{20}H_{32}O_3Na^+$ 343.2244; Found 343.2250.

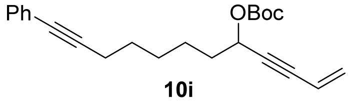

10i

***tert*-Butyl (12-phenyldodeca-1-en-3,11-diyn-5-yl) carbonate (10i):** eluent (petroleum ether/ethyl acetate = 75/1); colourless oil; 69% yield; ^{1}H NMR (400 MHz, $CDCl_3$): δ (ppm) 7.42–7.36 (m, 2H), 7.31–

7.26 (m, 3H), 5.86–5.75 (m, 1H), 5.66 (dd, J = 17.6, 2.4 Hz, 1H), 5.50 (dd, J = 11.2, 2.4 Hz, 1H), 5.31 (t, J = 6.4 Hz, 1H), 2.41 (t, J = 6.8 Hz, 2H), 1.89–1.81 (m, 2H), 1.66–1.61 (m, 2H), 1.56–1.51 (m, 4H), 1.50 (s, 9H); ^{13}C NMR (100 MHz, $CDCl_3$): δ (ppm) 152.7, 131.6, 128.2, 127.9, 127.5, 124.0, 116.5, 90.1, 86.8, 84.5, 82.6, 80.8, 67.4, 34.8, 28.6, 28.4, 27.8, 24.6, 19.3; HRMS (ESI-TOF) *m/z*: [M + Na]$^+$ Calcd for $C_{27}H_{28}O_3Na^+$ 375.1931; Found 375.1937.

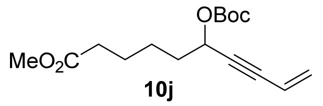

10j

Methyl 6-((*tert*-butoxycarbonyl)oxy)dec-9-en-7-ynoate (10j): eluent (petroleum ether/ethyl acetate = 30/1); colourless oil; 58% yield; ^{1}H NMR (400 MHz, $CDCl_3$): δ (ppm) 5.89–5.74 (m, 1H), 5.66 (dd, J = 17.6, 2.4 Hz, 1H), 5.51 (dd, J = 10.8, 2.4 Hz, 1H), 5.29 (t, J = 6.4 Hz, 1H), 3.67 (s, 3H), 2.33 (t, J = 7.2 Hz, 2H), 1.88–1.78 (m, 2H), 1.72–1.63 (m, 2H), 1.55–1.50 (m, 2H), 1.50 (s, 9H); ^{13}C NMR (100 MHz, $CDCl_3$): δ (ppm) 173.9, 152.6, 128.0, 116.4, 86.6, 84.5, 82.7, 67.1, 51.6, 34.5, 33.9, 27.8, 24.6, 24.5; HRMS (ESI-TOF) *m/z*: [M + Na]$^+$ Calcd for $C_{16}H_{24}O_5Na^+$ 319.1516; Found 319.1518.

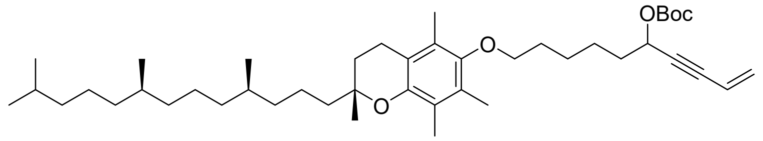

10k

***tert*-Butyl (10-(((*R*)-2,5,7,8-tetra methyl-2-((4*R*,8*R*)-4,8,12-trimethyl tridecyl)chroman-6-yl)oxy)dec-1-en-3-yn-5-yl) carbonate (10k):** eluent (petroleum ether/ethyl acetate = 20/1); colourless oil; 58% yield; ^{1}H NMR (400 MHz, $CDCl_3$): δ (ppm) 5.87–5.74 (m, 1H), 5.66 (dd, J = 17.6, 2.2 Hz, 1H), 5.50 (dd, J = 11.0, 2.2 Hz, 1H), 5.34–5.28 (m, 1H), 3.63 (t, J = 6.5 Hz, 2H), 2.56 (t, J = 6.7 Hz, 2H), 2.15 (s, 3H), 2.11 (s, 3H), 2.07 (s, 3H), 1.88–1.71 (m, 6H), 1.58–1.52 (m, 6H), 1.50 (s, 9H), 1.45–1.23 (m, 11H), 1.22 (s, 3H), 1.18–0.97 (m, 7H), 0.94–0.74 (m, 13H); ^{13}C NMR (100 MHz, $CDCl_3$): δ (ppm) 152.7, 148.3, 147.7, 127.9, 127.8, 125.8, 122.8, 117.5, 116.5, 86.9, 84.4, 82.6, 74.7, 72.8, 67.4, 40.11, 40.07, 39.4, 37.4, 37.3, 34.9, 32.80, 32.78, 32.70, 32.68, 31.30, 31.25, 30.2, 28.0, 27.8, 25.8, 24.9, 24.8, 24.4, 23.9, 22.7, 22.6, 21.1, 21.0, 20.7, 19.8, 19.7, 19.6, 12.7, 11.9, 11.8; HRMS (ESI-TOF) *m/z*: [M + Na]$^+$ Calcd for $C_{44}H_{72}O_5Na^+$ 703.5272; Found 703.5270.

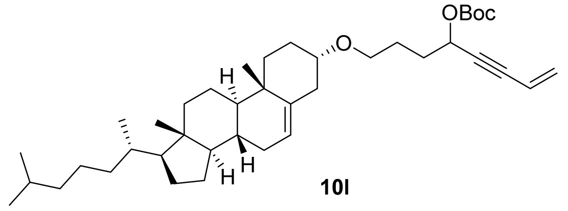

10l

***tert*-Butyl (1-(((3*S*,8*R*,9*R*,10*S*,13*S*,14*R*,17*S*)-10,13-dimethyl-17-((*S*)-6-methylheptan-2-yl)-2,3,4,7,8,9,10,11,12,13,14,15,16,17-tetradecahydro-1*H*-cyclopenta[*a*]phenanthren-3-yl)oxy)oct-7-en-5-yn-4-yl)**

carbonate (10l): eluent (petroleum ether/ethyl acetate = 20/1); colourless oil; 51% yield; ^{1}H NMR (400 MHz, $CDCl_3$): δ (ppm) 5.87–5.74 (m, 1H), 5.65 (dd, J = 17.6, 2.4 Hz, 1H), 5.49 (dd, J = 11.2, 2.4 Hz, 1H), 5.36–5.30 (m, 2H), 3.50 (t, J = 6.4 Hz, 2H), 3.17–3.08 (m, 1H), 2.38–2.32 (m, 1H), 2.18 (t, J = 11.8 Hz, 1H), 2.04–1.94 (m, 2H), 1.92–1.69 (m, 7H), 1.60–1.51 (m, 3H), 1.49 (s, 9H), 1.49–1.00 (m, 17H), 1.00 (s, 3H), 1.00–0.90 (m, 4H), 0.88–0.84 (m, 6H), 0.68 (s, 3H); ^{13}C NMR (150 MHz, $CDCl_3$): δ (ppm) 152.6, 141.0, 127.9, 121.5, 116.5, 86.7, 84.5, 82.6, 79.0, 67.22, 67.19, 56.8, 56.1, 50.2, 42.3, 39.8, 39.5, 39.1, 37.3, 36.9, 36.2, 35.8, 31.94, 31.88, 31.82, 28.4, 28.2, 28.0, 27.8, 25.7, 24.3, 23.8, 22.8, 22.6, 21.1, 19.4, 18.7, 11.8; HRMS (ESI-TOF) *m/z*: $[M + Na]^+$ Calcd for $C_{40}H_{64}O_4Na^+$ 631.4697; Found 631.4703.

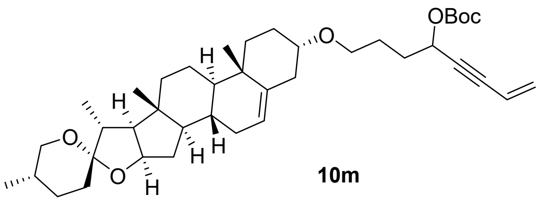

10m

***tert*-Butyl (1-(((4*S*,5'*S*,6a*S*,6b*R*,8a*R*,8b*S*,9*R*,10*S*,11a*R*,12a*R*,12b*R*)-5',6a,8a,9-tetramethyl-1,3,3',4,4',5,5',6,6a,6b,6',7,8,8a,8b,9,11a,12,12a,12b-icosahydrospiro[naphtho[2',1':4,5]indeno[2,1-*b*]furan-10,2'-pyran]-4-yl)oxy)oct-7-en-5-yn-4-yl) carbonate (10m)**: eluent (petroleum ether/ethyl acetate = 25/1); colourless oil; 56% yield; ^{1}H NMR (400 MHz, $CDCl_3$): δ (ppm) 5.89–5.74 (m, 1H), 5.65 (dd, J = 17.6, 2.4 Hz, 1H), 5.50 (dd, J = 10.8, 2.0 Hz, 1H), 5.36–5.29 (m, 2H), 4.41 (q, J = 7.2 Hz, 1H), 3.53–3.44 (m, 3H), 3.37 (t, J = 10.8 Hz, 1H), 3.17–3.07 (m, 1H), 2.40–2.32 (m, 1H), 2.22–2.13 (m, 1H), 2.03–1.95 (m, 2H), 1.92–1.82 (m, 5H), 1.80–1.52 (m, 12H), 1.49 (s, 9H), 1.47–1.08 (m, 6H), 1.01 (s, 3H), 0.99–0.93 (m, 4H), 0.81–0.76 (m, 6H); ^{13}C NMR (100 MHz, $CDCl_3$): δ (ppm) 152.7, 141.1, 127.9, 121.2, 116.5, 109.3, 86.7, 84.5, 82.6, 80.8, 79.0, 67.2, 66.9, 62.1, 56.5, 50.1, 41.6, 40.3, 39.8, 39.1, 37.2, 37.0, 32.1, 31.9, 31.8, 31.5, 31.4, 30.3, 28.8, 28.4, 27.8, 25.8, 20.9, 19.4, 17.1, 16.3, 14.5; HRMS (ESI-TOF) *m/z*: $[M + Na]^+$ Calcd for $C_{40}H_{60}O_6Na^+$ 659.4282; Found 659.4283.

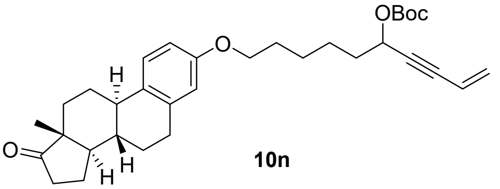

10n

***tert*-Butyl (10-(((8*S*,9*R*,13*R*,14*R*)-13-methyl-17-oxo-7,8,9,11,12,13,14,15,16,17-decahydro-6*H*-cyclopenta[*a*]phenanthren-3-yl)oxy)dec-1-en-3-yn-5-yl) carbonate (10n)**: eluent (petroleum ether/ethyl acetate = 20/1); colourless oil; 64% yield; ^{1}H NMR (400 MHz, $CDCl_3$): δ (ppm) 7.19 (d, J = 8.4 Hz, 1H), 6.73–6.67 (m, 1H), 6.66–6.60 (m, 1H), 5.80 (dd, J = 17.6, 11.2 Hz, 1H), 5.65 (d, J = 17.6 Hz, 1H), 5.50 (d, J = 11.2 Hz, 1H), 5.30 (t, J = 6.4 Hz, 1H), 3.93 (t, J = 6.4 Hz, 2H), 2.94–2.84 (m, 2H), 2.54–2.46 (m,

1H), 2.44–2.36 (m, 1H), 2.29–2.21 (m, 1H), 2.17–1.93 (m, 4H), 1.87–1.75 (m, 4H), 1.64–1.51 (m, 8H), 1.50 (s, 9H), 1.47–1.39 (m, 2H), 0.91 (s, 3H); ^{13}C NMR (150 MHz, $CDCl_3$): δ (ppm) 221.0, 157.1, 152.7, 137.7, 131.9, 127.9, 126.3, 116.5, 114.5, 112.1, 86.8, 84.5, 82.6, 67.6, 67.3, 50.4, 48.0, 44.0, 38.4, 35.9, 34.8, 31.6, 29.6, 29.1, 27.8, 26.6, 25.9, 25.7, 24.8, 21.6, 13.8; HRMS (ESI-TOF) *m/z*: $[M + Na]^+$ Calcd for $C_{33}H_{44}O_5Na^+$ 543.3081; Found 543.3077.

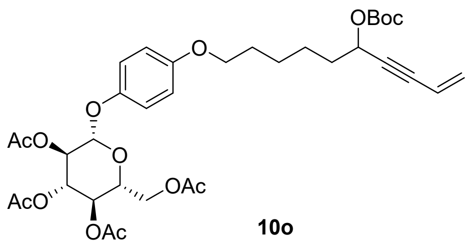

10o

(2*R*,3*R*,4*S*,5*R*,6*S*)-2-(Acetoxymethyl)-6-(4-((6-((*tert*-butoxycarbonyl)oxy)dec-9-en-7-yn-1-yl)oxy)phenoxy)tetrahydro-2*H*-pyran-3,4,5-triyl triacetate (10o): eluent (petroleum ether/ethyl acetate = 20/1); colourless oil; 57% yield; ^{1}H NMR (600 MHz, $CDCl_3$): δ (ppm) 6.92–6.90 (m, 2H), 6.79–6.77 (m, 2H), 5.84–5.75 (m, 1H), 5.63 (dd, *J* = 17.6, 2.4 Hz, 1H), 5.48 (dd, *J* = 10.8, 2.0 Hz, 1H), 5.29–5.20 (m, 3H), 5.16–5.12 (m, 1H), 4.93 (d, *J* = 7.8 Hz, 1H), 4.29–4.25 (m, 1H), 4.16–4.13 (m, 1H), 3.89 (t, *J* = 6.6 Hz, 2H), 3.80–3.77 (m, 1H), 2.06 (d, *J* = 6.0 Hz, 6H), 2.02 (d, *J* = 6.0 Hz, 6H), 1.85–1.81 (m, 2H), 1.78–1.74 (m, 2H), 1.54–1.49 (m, 4H), 1.47 (s, 9H); ^{13}C NMR (100 MHz, $CDCl_3$): δ (ppm) 170.6, 170.3, 169.4, 169.3, 155.3, 152.7, 150.8, 128.0, 118.7, 116.4, 115.2, 100.3, 86.7, 84.5, 82.7, 72.7, 71.9, 71.2, 68.3, 68.2, 67.3, 61.9, 34.8, 29.1, 27.8, 25.6, 24.8, 20.8, 20.69, 20.65, 20.6; HRMS (ESI-TOF) *m/z*: $[M + Na]^+$ Calcd for $C_{35}H_{46}O_{14}Na^+$ 713.2780; Found 713.2783.

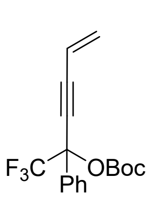

10y

***tert*-Butyl (1,1,1-trifluoro-2-phenylhex-5-en-3-yn-2-yl) carbonate (10y)**: eluent (petroleum ether/ethyl acetate = 70/1); colourless oil; 68% yield; ^{1}H NMR (400 MHz, $CDCl_3$): δ (ppm) 7.69–7.64 (m, 2H), 7.44–7.40 (m, 3H), 6.02–5.91 (m, 1H), 5.91–5.85 (m, 1H), 5.71 (dd, *J* = 10.8, 2.8 Hz, 1H), 1.41 (s, 9H); ^{13}C NMR (100 MHz, $CDCl_3$): δ (ppm) 149.3, 133.3, 130.4, 129.7, 128.4, 122.1 (q, $^1J_{FC}$ = 282.7 Hz), 115.6, 88.4, 84.2, 81.1, 78.8 (q, $^2J_{FC}$ = 32.2 Hz), 27.6; ^{19}F NMR (375 MHz, $CDCl_3$): δ (ppm) –78.4; HRMS (ESI-TOF) *m/z*: $[M + Na]^+$ Calcd for $C_{17}H_{17}F_3O_3Na^+$ 349.1022; Found 349.1026.

2.2 Procedure for preparation of *ortho*-functionalised aryl enones[2]

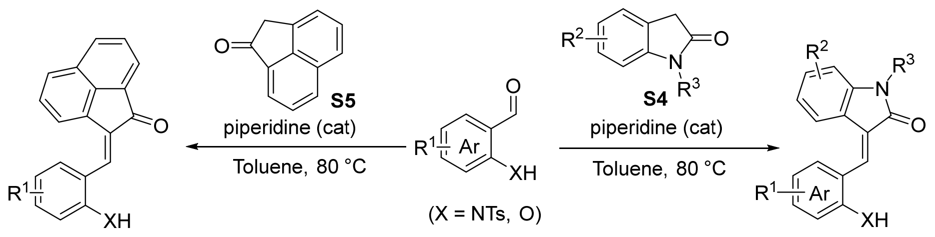

4, 13 **S3** **2, 11, 15**

To a solution of **S3** (2.0 mmol) in toluene (10 mL) were added **S4** or **S5** (2.2 mmol) and piperidine (2−3 drops). The mixture was heated at 80 °C for 3–8 h, and monitored by TLC (petroleumether/EtOAc = 5/1). After completion, the mixture was cooled to room temperature and the solid was collected by filtration and then dried in vacuum to give *ortho*-functionalised aryl enones **2**, **11** and **15**.

Characterisation for selected *ortho*-functionalised aryl enones:

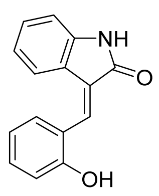

2m

Yellow solid, 71% yield; mp 233–235 °C; ^{1}H NMR (400 MHz, DMSO-d_6): δ (ppm) 10.57 (s, 1H), 10.19 (s, 1H), 7.72 (s, 1H), 7.64 (d, *J* = 6.8 Hz, 1H), 7.52 (d, *J* = 7.6 Hz, 1H), 7.37–7.29 (m, 1H), 7.26–7.18 (m, 1H), 7.00 (d, *J* = 8.0 Hz, 1H), 6.96–6.91 (m, 1H), 6.91–6.80 (m, 2H); ^{13}C NMR (100 MHz, DMSO-d_6): δ (ppm) 169.3, 157.0, 143.1, 133.0, 132.1, 130.2, 130.0, 127.0, 122.8, 121.82, 121.79, 121.5, 119.3, 116.5, 110.4; HRMS (ESI-TOF) *m/z*: $[M + Na]^+$ Calcd for $C_{15}H_{11}NO_2Na^+$ 260.0682; Found 260.0682.

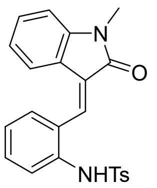

11a

Yellow solid, 75% yield; mp 247–249 °C; ^{1}H NMR (400 MHz, DMSO-d_6): δ (ppm) 10.01 (s, 1H), 7.65 (s, 1H), 7.56 (d, *J* = 7.6 Hz, 1H), 7.48–7.43 (m, 1H), 7.42–7.37 (m, 2H), 7.37–7.27 (m, 3H), 7.05 (d, *J* = 7.6 Hz, 1H), 7.00 (d, *J* = 7.6 Hz, 1H), 6.95 (d, *J* = 8.0 Hz, 2H), 6.86–6.79 (m, 1H), 3.20 (s, 3H), 2.00 (s, 3H); ^{13}C NMR (100 MHz, DMSO-d_6): δ (ppm) 167.2, 144.4, 143.3, 137.4, 135.7, 133.9, 131.8, 131.0, 130.4, 130.2, 129.8, 128.5, 128.1, 127.0, 126.9, 122.6, 121.9, 120.6, 109.1, 26.3, 21.2; HRMS (ESI-TOF) *m/z*: $[M + Na]^+$ Calcd for $C_{23}H_{20}N_2O_3SNa^+$ 427.1087; Found 427.1089.

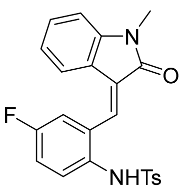

11c

Yellow solid, 77% yield; mp 272–274 °C; ^{1}H NMR (400 MHz, DMSO-d_6): δ (ppm) 9.97 (s, 1H), 7.53 (s, 1H), 7.42–7.25 (m, 6H), 7.01 (d, *J* = 8.0 Hz, 2H), 6.92 (d, *J* = 8.0 Hz, 2H), 6.89–6.81 (m, 1H), 3.20 (s, 3H), 1.98 (s, 3H); ^{13}C NMR (100 MHz, DMSO-d_6): δ (ppm) 167.0, 160.5 (d, $^{1}J_{FC}$ = 243.8 Hz), 144.5, 143.3, 137.1, 134.4 (d, $^{3}J_{FC}$ = 8.7 Hz), 132.2, 131.9 (d, $^{4}J_{FC}$ = 2.8 Hz), 131.5 (d, $^{3}J_{FC}$ = 9.0 Hz), 130.7, 129.8, 128.9, 126.9, 122.7, 122.0, 120.2, 117.7 (d, $^{1}2_{FC}$ = 22.3 Hz), 116.5 (d, $^{2}J_{FC}$ = 23.2 Hz), 109.2, 26.4, 21.2; ^{19}F NMR (375 MHz, DMSO-d_6): δ (ppm) –114.6; HRMS (ESI-TOF) *m/z*: [M + Na]$^+$ Calcd for $C_{23}H_{19}FN_2O_3SNa^+$ 445.0993; Found 445.0996.

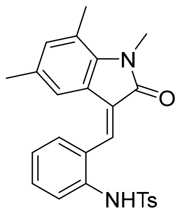

11d

Yellow solid, 74% yield; mp 248–250 °C; ^{1}H NMR (400 MHz, DMSO-d_6): δ (ppm) 9.99 (s, 1H), 7.59 (s, 1H), 7.51–7.46 (m, 1H), 7.46–7.37 (m, 3H), 7.35–7.26 (m, 2H), 6.99 (d, *J* = 7.6 Hz, 2H), 6.84 (s, 1H), 6.65 (s, 1H), 3.45 (s, 3H), 2.06 (s, 3H), 2.03 (s, 3H); ^{13}C NMR (100 MHz, DMSO-d_6): δ (ppm) 168.0, 143.3, 140.0, 137.5, 135.6, 134.5, 133.4, 131.8, 130.8, 130.3, 130.1, 129.8, 128.2, 127.9, 127.0, 126.7, 121.32, 121.28, 119.9, 29.5, 21.2, 20.9, 18.9; HRMS (ESI-TOF) *m/z*: [M + Na]$^+$ Calcd for $C_{25}H_{24}N_2O_3SNa^+$ 455.1400; Found 455.1401.

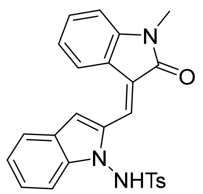

15a

Yellow solid, 62% yield; mp 286–288 °C; ^{1}H NMR (400 MHz, DMSO-d_6): δ (ppm) 11.70 (s, 1H), 7.93 (d, *J* = 7.6 Hz, 1H), 7.67 (d, *J* = 8.0 Hz, 1H), 7.47–7.32 (m, 4H), 7.32–7.25 (m, 1H), 7.22–7.11 (m, 3H), 7.10–6.99 (m, 2H), 6.88 (d, *J* = 8.0 Hz, 2H), 3.21 (s, 3H), 1.88 (s, 3H); ^{13}C NMR (100 MHz, DMSO-d_6): δ (ppm) 167.4, 144.5, 144.2, 138.1, 134.6, 133.2, 130.5, 128.0, 125.5, 125.4, 125.1, 122.9, 122.7, 122.2, 121.90, 121.86, 120.6, 111.1, 109.0, 106.5, 26.4, 21.0; HRMS (ESI-TOF) *m/z*: [M + Na]$^+$ Calcd for $C_{25}H_{21}N_3O_3SNa^+$ 466.1196; Found 466.1195.

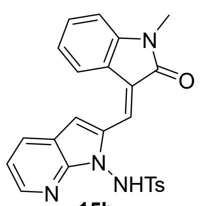

15b

Yellow solid, 67% yield; mp 297–299 °C; ^{1}H NMR (400 MHz, DMSO-d_6): δ (ppm) 11.72 (s, 1H), 8.45 (s, 1H), 8.29–8.22 (m, 1H), 8.16–8.09 (m, 1H), 7.59–7.48 (m, 2H), 7.39–7.31 (m, 2H), 7.25–7.12 (m, 4H), 7.11–7.05 (m, 1H), 7.03 (d, *J* = 8.0 Hz, 1H), 3.24 (s, 3H), 2.08 (s, 3H); ^{13}C NMR (100 MHz, DMSO-d_6): δ (ppm) 165.4, 147.9, 146.8, 144.7, 142.4, 136.0, 133.3, 130.9, 130.3, 129.9, 128.0, 125.5, 123.3, 122.1, 120.5, 119.8, 118.7, 118.3, 109.0, 108.5, 26.4, 21.3; HRMS (ESI-TOF) *m/z*: [M + Na]$^+$ Calcd for $C_{24}H_{20}N_4O_3SNa^+$ 467.1148; Found 467.1152.

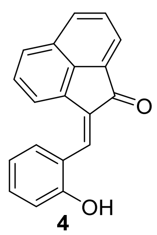

4

Yellow solid, 82% yield; mp 196–198 °C; ^{1}H NMR (400 MHz, DMSO-d_6): δ (ppm) 10.27 (s, 1H), 8.28 (d, J = 8.4 Hz, 1H), 8.07 (d, J = 6.8 Hz, 1H), 8.01 (d, J = 8.0 Hz, 1H), 7.93 (s, 1H), 7.89–7.82 (m, 2H), 7.79 (d, J = 6.8 Hz, 1H), 7.68–7.60 (m, 1H), 7.44–7.34 (m, 1H), 7.07 (d, J = 8.4 Hz, 1H), 7.04–6.97 (m, 1H); ^{13}C NMR (100 MHz, DMSO-d_6): δ (ppm) 192.2, 157.3, 139.9, 132.7, 132.4, 132.2, 131.6, 130.8, 130.3, 128.9, 128.7, 126.4, 121.9, 121.8, 120.3, 119.3, 116.5; HRMS (ESI-TOF) *m/z*: [M + H]$^+$ Calcd for $C_{19}H_{13}O_2^+$ 273.0910; Found 273.0911.

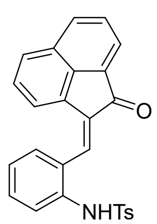

13

Yellow solid, 65% yield; mp 218–220 °C; ^{1}H NMR (400 MHz, DMSO-d_6): δ (ppm) 10.04 (s, 1H), 8.29 (d, J = 8.0 Hz, 1H), 8.10–7.97 (m, 2H), 7.91–7.83 (m, 1H), 7.74 (s, 1H), 7.71–7.65 (m, 1H), 7.59–7.49 (m, 2H), 7.45–7.38 (m, 2H), 7.38–7.23 (m, 3H), 6.68 (d, J = 7.6 Hz, 2H), 1.39 (s, 3H); ^{13}C NMR (100 MHz, DMSO-d_6): δ (ppm) 191.6, 142.9, 140.1, 137.3, 135.8, 134.6, 132.2, 131.99, 131.97, 131.90, 131.0, 130.8, 130.4, 129.6, 129.1, 128.8, 127.1, 126.9, 126.5, 121.8, 120.5, 20.4; HRMS (ESI-TOF) *m/z*: [M + H]$^+$ Calcd for $C_{26}H_{20}NO_3S^+$ 426.1158; Found 426.1157.

2.3 Procedure for the preparation of substrate 8

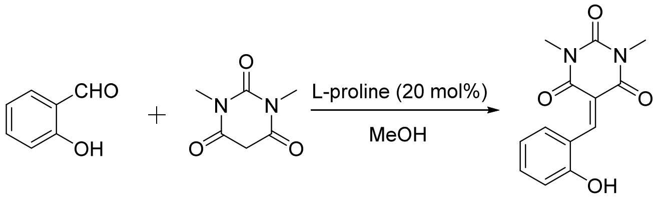

To a solution of salicylaldehyde (1.22 g, 10.0 mmol) in MeOH (30 mL) were added 1,3-dimethylbarbituric acid (1.72 g, 11.0 mmol) and L-proline (0.23 g, 2.0 mmol). The mixture was stirred at room temperature for 5 h, and monitored by TLC (petroleumether/EtOAc = 5/1). After completion, the solid was collected by filtration and then dried in vacuo to give product **8**: 1.85 g (7.11 mmol), as a yellow solid, 71% yield; mp 204–206 °C; ^{1}H NMR (400 MHz, DMSO-d_6): δ (ppm) 10.59 (s, 1H), 8.67 (s, 1H), 8.10 (d, J = 8.8 Hz, 1H), 7.41–7.34 (m, 1H), 6.94 (d, J = 8.0 Hz, 1H), 6.86–6.80 (m, 1H), 3.23 (s, 3H), 3.18 (s, 3H); ^{13}C NMR (100 MHz, DMSO-d_6): δ (ppm) 162.9, 160.9, 159.4, 151.8, 151.7, 135.1, 133.1, 120.5, 118.6, 117.4, 115.9, 29.0, 28.4; HRMS (ESI-TOF) *m/z*: [M + H]$^+$ Calcd for $C_{13}H_{13}N_2O_4^+$ 261.0807; Found 261.0814.

Other substrates, including **2a**–**2l**, **6** and **10x** are known compounds, and the spectra of these compounds were consistent with the reports.[1,3]

References

1. (*a*) C. Li, Z. Zhou, Y. Li, Y. Guo and S. Ma, *Chem. Commun.* 2023, **59**, 3727–3730; (*b*) Z.-L. Wang and R. Zhu, *J. Am. Chem. Soc.* 2024, **146**, 19377–19385.

2. S. Sambhaji Vagh, B.-J. Hou, A. Edukondalu, P.-C. Wang, Y.-R. Chen and W. Lin, *Adv. Synth. Catal.* 2021, **363**, 5429–5435.

3. J. Sun, X. Liu, Q. Sun, Y. Han and C.-G. Yan, *J. Org. Chem.* 2023, **88**, 11562–11580.

3. Detailed condition optimisations

3.1 Detailed condition optimisations for asymmetric synthesis of 3a[a]

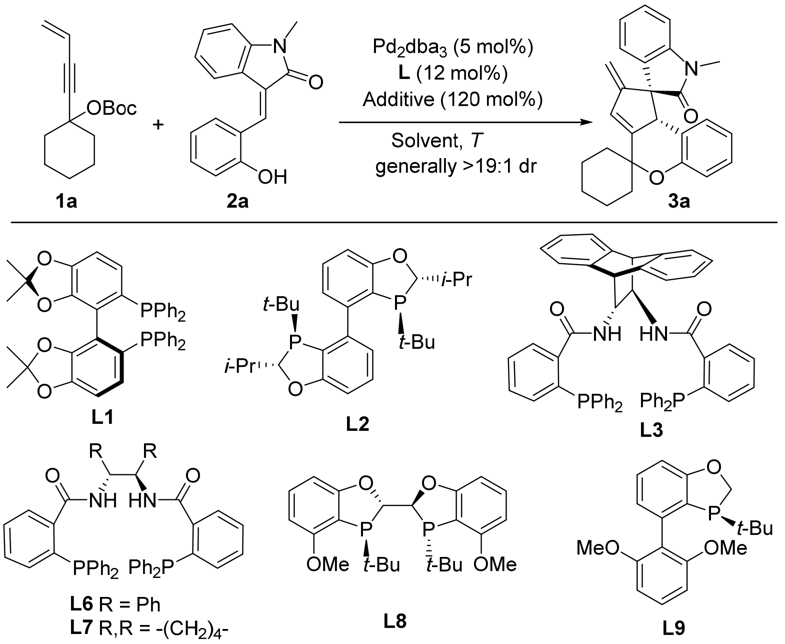

Entry	Ligand	Solvent	*T* (° C)	*t* (h)	Additive	Yield (%)[b]	ee (%)[c]
1	**L1**	MeCN	80	24	/	56	29
2	**L2**	MeCN	80	24	/	33	58
3	**L3**	MeCN	80	24	/	63	74
4	**L6**	MeCN	80	24	/	trace	/
5	**L7**	MeCN	80	24	/	trace	/
6	**L8**	MeCN	80	24	/	40	9
7[d]	**L9**	MeCN	80	24	/	48	8
8	**L3**	MeCN	50	48	/	63	85
9	**L3**	DCE	50	48	/	32	78
10	**L3**	NMP	50	48	/	58	87
11	**L3**	DMF	50	48	/	62	85

12	**L3**	DMA	50	48	/	57	88
13[e]	**L3**	DMA	50	48	/	60	88
14[e]	**L3**	DMA	50	48	$KHCO_3$	73	89
15[e]	**L3**	DMA	45	60	$KHCO_3$	64	91
16[e]	**L3**	DMA	45	60	K_2CO_3	62	91
17[e]	**L3**	DMA	45	60	K_3PO_4	61	91
18[e]	**L3**	DMA	45	60	Et_3N	trace	/
19[e]	**L3**	DMA	45	60	BzOH	trace	/
20[e,f]	**L3**	DMA	45	60	$KHCO_3$	72	91
21[e,f,g]	**L3**	DMA	45	60	$KHCO_3$	48	91

[a] Unless noted otherwise, reactions were performed with 3-vinyl propargylic carbonate **1a** (0.1 mmol), (*E*)-3-(2-hydroxybenzylidene)oxindole **2a** (0.120 mmol), Pd_2dba_3 (5 mol%), ligand **L** (12 mol%) in degassed solvent (1.0 mL) under Ar atmosphere. [b] Yield of the isolated product. [c] Determined by HPLC analysis on a chiral stationary phase, and >19:1 dr was determined by ^{1}H NMR analysis of crude product. [d] With **L9** (20 mol%). [e] With DMA (2.0 mL). [f] With **1a** (0.15 mmol) and **2a** (0.1 mmol). [g] With Pd_2dba_3 (2.5 mol%), **L3** (6 mol%). NMP = *N*-methylpyrrolidin-2-one; DMF = Dimethylformamide; DMA = Dimethylacetamide.

3.2 Detailed condition optimisations for asymmetric synthesis of 7[a]

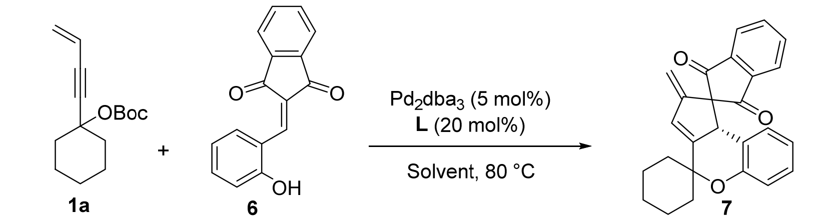

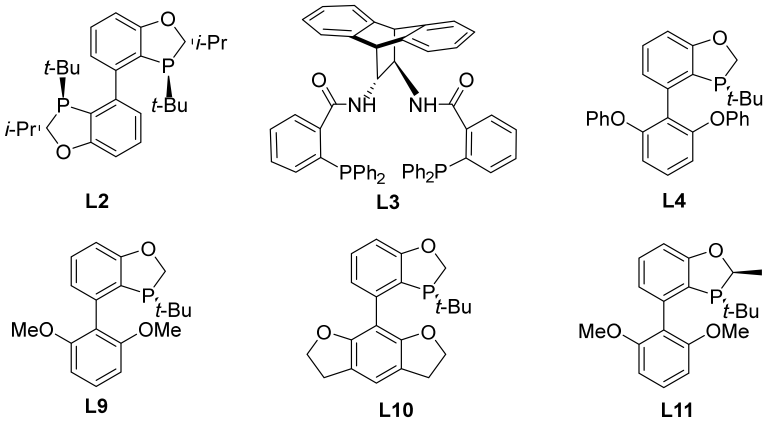

Entry	Ligand	Solvent	*T* (° C)	*t* (h)	Yield (%)[b]	ee (%)[c]
1[d]	**L2**	MeCN	80	24	trace	/
2[d]	**L3**	MeCN	80	24	trace	/
3	**L3**	toluene	80	24	trace	/
4	**L4**	MeCN	80	24	trace	/
5	**L4**	toluene	80	24	57	51

6	**L9**	toluene	80	24	65	3
7	**L10**	toluene	80	24	64	42
8	**L11**	toluene	80	24	63	10
9	**L4**	THF	80	24	53	48
10	**L4**	DMA	80	24	trace	/
11	**L4**	DCE	80	24	46	49
12	**L4**	toluene	60	36	35	52

[a] Unless noted otherwise, reactions were performed with 3-vinyl propargylic carbonate **1a** (0.15 mmol), 2-(2-hydroxybenzylidene)-1*H*-indene-1,3(2*H*)-dione **6** (0.1 mmol), Pd_2dba_3 (5 mol%) and ligand **L** (20 mol%) in degassed solvent (1.0 mL) under Ar atmosphere. [b] Yield of the isolated product. [c] Determined by HPLC analysis on a chiral stationary phase, and >19:1 dr was determined by ^{1}H NMR analysis of crude product. [d] With ligand **L** (12 mol%).

3.3 Detailed condition optimisations for asymmetric synthesis of 12a[a]

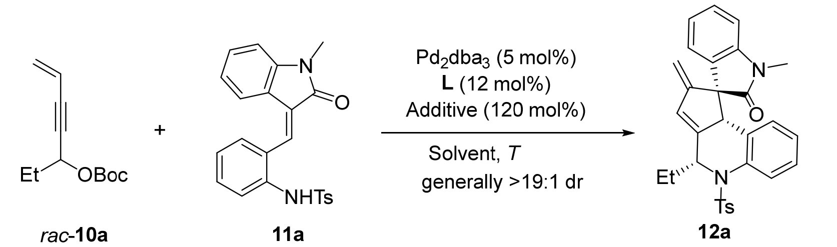

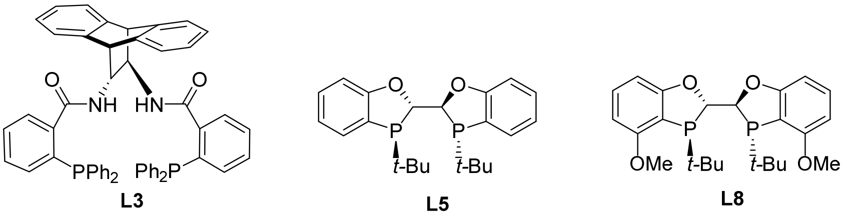

Entry	Ligand	Solvent	*T* (° C)	*t* (h)	Additive	Yield (%)[b]	ee (%)[c]
1	**L3**	MeCN	60	36	$KHCO_3$	54	36
2	**L5**	MeCN	60	36	$KHCO_3$	72	83
3	**L8**	MeCN	60	36	$KHCO_3$	52	85
4	**L5**	MeCN	50	48	$KHCO_3$	71	85
5	**L5**	MeCN	45	72	$KHCO_3$	70	93
6	**L5**	MeCN	40	72	$KHCO_3$	low conv.	/
7	**L5**	MeCN	45	72	/	low conv.	/
8[d]	**L5**	MeCN	45	72	$KHCO_3$	35	92
9[e]	**L5**	MeCN	45	72	$KHCO_3$	71	85
10	**L5**	MeCN	45	72	K_2CO_3	low conv	/
11	**L5**	toluene	45	72	$KHCO_3$	38	74
12	**L5**	THF	45	72	$KHCO_3$	71	75
13	**L5**	DMA	45	72	$KHCO_3$	low conv.	/

[a] Unless noted otherwise, reactions were performed with *tert*-butyl hept-6-en-4-yn-3-yl carbonate **10a** (0.2 mmol), (*E*)-4-methyl-*N*-(2-((1-methyl-2-oxoindolin-3-ylidene)methyl)phenyl)benzenesulfonamide **11a** (0.1 mmol), Pd_2dba_3 (5 mol%), ligand **L** (12 mol%) in degassed solvent (1.0 mL) under Ar atmosphere. [b] Yield of the isolated product. [c] Determined by HPLC analysis on a chiral stationary phase, and >19:1 dr was determined by ^{1}H NMR analysis of crude product. [d] With MeCN (0.5 mL). [e] With MeCN (2.0 mL).

4. General procedure for the reactions of achiral 4-alken-2-ynyl carbonates

4.1 General procedure for synthesis of 3 and 5

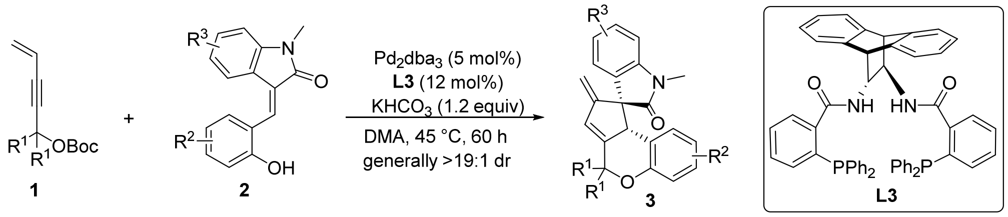

General procedure: To an oven-dried 10 mL Schlenk tube equipped with a magnetic stirring bar were added 4-alken-2-ynyl carbonate **1** (0.15 mmol, 1.5 equiv), activated alkene **2** (0.1 mmol, 1.0 equiv), Pd_2dba_3 (4.6 mg, 0.0050 mmol, 5 mol%), **L3** (9.8 mg, 0.012 mmol, 12 mol%) and $KHCO_3$ (12.0 mg, 0.120 mmol, 1.2 equiv). The tube was capped, evacuated and back-filled with argon for five times. Then degassed dry DMA (2.0 mL) was added via syringe. The mixture was stirred at 45 °C for 60 h, and monitored by TLC (EtOAc/petroleum ether). After completion, the solvent was evaporated in vacuo. The residue was purified by flash chromatography on silica gel (EtOAc/petroleum ether) to give the product.

*Note: racemic **3** was prepared from **1** and **2** through the same procedure under the catalysis of $Pd(PPh_3)_4$ (5 mol%).*

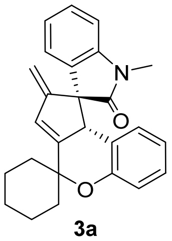

3a

Synthesis of 3a: To an oven-dried 10 mL Schlenk tube equipped with a magnetic stirring bar were added 1-(but-3-en-1-yn-1-yl)cyclohexyl *tert*-butyl carbonate **1a** (37.6 mg, 0.150 mmol, 1.5 equiv), (*E*)-3-(2-hydroxybenzylidene)-1-methylindolin-2-one **2a** (25.1 mg, 0.0999 mmol, 1.0 equiv), Pd_2dba_3 (4.6 mg, 0.0050 mmol, 5 mol%), **L3** (9.8 mg, 0.012 mmol, 12 mol%) and $KHCO_3$ (12.0 mg, 0.120 mmol, 1.2 equiv). The tube was capped, evacuated and back-filled with argon for five times. Then degassed dry DMA (2.0 mL) was added via syringe. The mixture was stirred at 45 °C for 60 h, and monitored by TLC (EtOAc/petroleum ether = 1/8). After completion, the solvent was evaporated in vacuo. The residue was purified by flash chromatography on silica gel (EtOAc/petroleum ether = 1/8) to give the product **3a**: 27.6 mg (0.0720 mmol), as a white solid, 72% yield; mp 157–159 °C; >19:1 dr, determind by ^{1}H

NMR analysis of crude product; $[\alpha_D^{25}]$ = –58.3 (c = 3.46 in $CHCl_3$); 91% ee, determined by HPLC analysis (Daicel Chiralpak AD, n-hexane/i-PrOH = 95/5, flow rate =1.0 mL/min, l = 254 nm) t_R = 6.10 min (minor), t_R = 7.36 min (major); ^1H NMR (400 MHz, $CDCl_3$): δ (ppm) 7.12–7.05 (m, 1H), 6.95–6.87 (m, 1H), 6.84–6.76 (m, 2H), 6.70–6.61 (m, 2H), 6.48–6.41 (m, 1H), 6.23 (d, J = 2.8 Hz, 1H), 6.17 (d, J = 7.6 Hz, 1H), 4.96 (s, 1H), 4.76 (s, 1H), 4.55 (s, 1H), 3.40 (s, 3H), 2.15–2.05 (m, 1H), 2.04–1.93 (m, 3H), 1.81–1.67 (m, 2H), 1.59–1.32 (m, 4H); ^{13}C NMR (100 MHz, $CDCl_3$): δ (ppm) 178.7, 155.8, 152.9, 152.5, 142.6, 132.5, 127.74, 127.68, 127.6, 124.5, 122.9, 122.7, 121.8, 120.4, 117.5, 108.0, 105.3, 76.1, 62.9, 49.9, 34.4, 32.5, 26.8, 25.6, 21.7, 21.3; HRMS (ESI-TOF) m/z: [M + Na]$^+$ Calcd for $C_{26}H_{25}NO_2Na^+$ 406.1778; Found 406.1777.

Asymmetric synthesis of 3a on a 1.0 mmol scale

To an oven-dried 50 mL Schlenk tube equipped with a magnetic stirring bar were added 1-(but-3-en-1-yn-1-yl)cyclohexyl *tert*-butyl carbonate **1a** (375.5 mg, 1.500 mmol), (*E*)-3-(2-hydroxy benzylidene) -1-methylindolin-2-one **2a** (251.3 mg, 1.000 mmol) , Pd_2dba_3 (45.8 mg, 0.0500 mmol, 5 mol%), **L3** (97.5 mg, 0.120 mmol, 12 mol%) and $KHCO_3$ (120.2 mg, 1.201 mmol). The tube was capped, evacuated and back-filled with argon for five times. Then degassed dry DMA (20 mL) was added via syringe. The mixture was stirred at 45 °C for 60 h, and monitored by TLC (EtOAc/petroleum ether = 1/8). After completion, the solvent was evaporated in vacuo. The residue was purified by flash chromatography on silica gel (EtOAc/petroleum ether = 1/8) to give the product **3a**: 268.5 mg (0.7001 mmol), as a white solid, 70% yield, >19:1 dr, 91% ee.

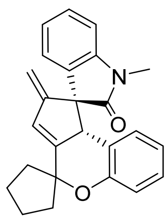
3b

Synthesis of 3b: To an oven-dried 10 mL Schlenk tube equipped with a magnetic stirring bar were added 1-(but-3-en-1-yn-1-yl)cyclopentyl *tert*-butyl carbonate **1b** (35.5 mg, 0.150 mmol, 1.5 equiv), (*E*)-3-(2-hydroxybenzylidene)-1-methylindolin-2-one **2a** (25.1 mg, 0.0999 mmol, 1.0 equiv), Pd_2dba_3 (4.6 mg, 0.0050 mmol, 5 mol%), **L3** (9.8 mg, 0.012 mmol, 12 mol%) and $KHCO_3$ (12.0 mg, 0.120 mmol, 1.2 equiv). The tube was capped, evacuated and back-filled with argon for five times. Then degassed dry DMA (2.0 mL) was added via syringe. The mixture was stirred at 45 °C for 60 h, and monitored by TLC (EtOAc/petroleum ether = 1/8). After completion, the solvent was evaporated in vacuo. The residue was purified by flash chromatography on silica gel (EtOAc/petroleum ether = 1/8) to give the product **3b**: 28.1 mg (0.0761 mmol), as a white solid, 76% yield; mp 146–148 °C; >19:1 dr, determind by ^1H

NMR analysis of crude product; [α_D^{25}] = −77.6 (c = 5.28 in $CHCl_3$); 95% ee, determined by HPLC analysis (Daicel Chiralpak AD, n-hexane/i-PrOH = 95/5, flow rate =1.0 mL/min, l = 254 nm) t_R = 7.61 min (minor), t_R = 9.69 min (major); ^{1}H NMR (400 MHz, $CDCl_3$): δ (ppm) 7.13–7.05 (m, 1H), 6.93–6.86 (m, 1H), 6.81 (d, J = 8.0 Hz, 1H), 6.75–6.70 (m, 1H), 6.70–6.62 (m, 2H), 6.48–6.41 (m, 1H), 6.23 (d, J = 2.8 Hz, 1H), 6.16 (d, J = 8.0 Hz, 1H), 4.96 (s, 1H), 4.77 (s, 1H), 4.55 (s, 1H), 3.40 (s, 3H), 2.29–2.14 (m, 2H), 2.11–1.99 (m, 2H), 1.91–1.72 (m, 4H); ^{13}C NMR (100 MHz, $CDCl_3$): δ (ppm) 178.7, 155.7, 153.5, 150.5, 142.6, 132.4, 127.8, 127.6, 123.6, 123.0, 122.7, 121.8, 120.4, 117.6, 108.0, 105.5, 86.3, 63.1, 50.7, 36.4, 36.0, 26.8, 23.8, 23.6; HRMS (ESI-TOF) m/z: [M + Na]$^+$ Calcd for $C_{25}H_{23}NO_2Na^+$ 392.1621; Found 392.1623.

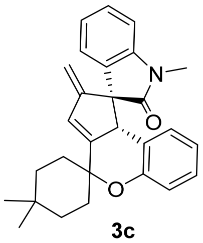
3c

Synthesis of 3c: To an oven-dried 10 mL Schlenk tube equipped with a magnetic stirring bar were added 1-(but-3-en-1-yn-1-yl)-4,4-dimethylcyclohexyl *tert*-butyl carbonate **1c** (41.7 mg, 0.150 mmol, 1.5 equiv), (E)-3-(2-hydroxybenzylidene)-1-methylindolin-2-one **2a** (25.1 mg, 0.0999 mmol, 1.0 equiv), Pd_2dba_3 (4.6 mg, 0.0050 mmol, 5 mol%), **L3** (9.8 mg, 0.012 mmol, 12 mol%) and $KHCO_3$ (12.0 mg, 0.120 mmol, 1.2 equiv). The tube was capped, evacuated and back-filled with argon for five times. Then degassed dry DMA (2.0 mL) was added via syringe. The mixture was stirred at 45 °C for 60 h, and monitored by TLC (EtOAc/petroleum ether = 1/7). After completion, the solvent was evaporated in vacuo. The residue was purified by flash chromatography on silica gel (EtOAc/petroleum ether = 1/7) to give the product **3c**: 24.7 mg (0.0600 mmol), as a white solid, 60% yield; mp 197−198 °C; >19:1 dr, determind by ^{1}H NMR analysis of crude product; [α_D^{25}] = −31.5 (c = 6.32 in $CHCl_3$); 56% ee, determined by HPLC analysis (Daicel Chiralpak IBN-5, n-hexane/i-PrOH = 95/5, flow rate =1.0 mL/min, l = 254 nm) t_R = 6.87 min (minor), t_R = 8.54 min (major); ^{1}H NMR (400 MHz, $CDCl_3$): δ (ppm) 7.12–7.02 (m, 1H), 6.95–6.87 (m, 1H), 6.84–6.73 (m, 2H), 6.70–6.57 (m, 2H), 6.48–6.41 (m, 1H), 6.25 (d, J = 2.8 Hz, 1H), 6.16 (d, J = 7.6 Hz, 1H), 4.96 (s, 1H), 4.76 (s, 1H), 4.55 (s, 1H), 3.39 (s, 3H), 2.18–2.08 (m, 1H), 2.06–1.99 (m, 1H), 1.96–1.80 (m, 2H), 1.68–1.58 (m, 1H), 1.55–1.40 (m, 2H), 1.20–1.13 (m, 1H), 1.01 (s, 3H), 0.97 (s, 3H); ^{13}C NMR (100 MHz, $CDCl_3$): δ (ppm) 178.7, 155.8, 152.9, 152.3, 142.6, 132.4, 127.8, 127.7, 127.6, 124.4, 122.9, 122.7, 121.8, 120.4, 117.5, 108.0, 105.4, 75.9, 62.9, 50.0, 34.3, 34.0, 31.2, 30.5, 29.7, 28.6, 26.8, 25.2; HRMS (ESI-TOF) m/z: [M + H]$^+$ Calcd for $C_{28}H_{30}NO_2^+$ 412.2271; Found 412.2274.

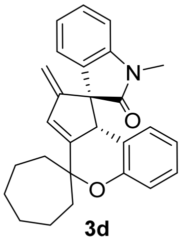

3d

Synthesis of 3d: To an oven-dried 10 mL Schlenk tube equipped with a magnetic stirring bar were added 1-(but-3-en-1-yn-1-yl)cycloheptyl *tert*-butyl carbonate **1d** (39.7 mg, 0.150 mmol, 1.5 equiv), (*E*)-3-(2-hydroxybenzylidene)-1-methylindolin-2-one **2a** (25.1 mg, 0.0999 mmol, 1.0 equiv), Pd_2dba_3 (4.6 mg, 0.0050 mmol, 5 mol%), **L3** (9.8 mg, 0.012 mmol, 12 mol%) and $KHCO_3$ (12.0 mg, 0.120 mmol, 1.2 equiv). The tube was capped, evacuated and back-filled with argon for five times. Then degassed dry DMA (2.0 mL) was added via syringe. The mixture was stirred at 45 °C for 60 h, and monitored by TLC (EtOAc/petroleum ether = 1/7). After completion, the solvent was evaporated in vacuo. The residue was purified by flash chromatography on silica gel (EtOAc/petroleum ether = 1/7) to give the product **3d**: 25.1 mg (0.0631 mmol), as a white solid, 63% yield; mp 174–176 °C; >19:1 dr, determind by ^{1}H NMR analysis of crude product; $[\alpha_D^{25}]$ = –55.6 (*c* = 4.32 in $CHCl_3$); 81% ee, determined by HPLC analysis (Daicel Chiralpak AD, *n*-hexane/*i*-PrOH = 90/10, flow rate =1.0 mL/min, l = 254 nm) t_R = 6.26 min (minor), t_R = 7.18 min (major); ^{1}H NMR (400 MHz, $CDCl_3$): δ (ppm) 7.13–7.04 (m, 1H), 6.94–6.85 (m, 1H), 6.80 (d, *J* = 8.0 Hz, 1H), 6.78–6.71 (m, 1H), 6.70–6.57 (m, 2H), 6.48–6.39 (m, 1H), 6.24 (d, *J* = 2.8 Hz, 1H), 6.15 (d, *J* = 7.6 Hz, 1H), 4.96 (s, 1H), 4.74 (s, 1H), 4.55 (s, 1H), 3.40 (s, 3H), 2.36–2.26 (m, 1H), 2.19–2.05 (m, 2H), 2.02–1.91 (m, 1H), 1.84–1.61 (m, 6H), 1.56–1.33 (m, 2H); ^{13}C NMR (100 MHz, $CDCl_3$): δ (ppm) 178.7, 155.7, 153.2, 153.0, 142.6, 132.5, 127.74, 127.67, 127.5, 124.4, 122.9, 122.7, 121.6, 120.2, 117.6, 108.0, 105.3, 80.7, 62.9, 49.8, 38.4, 35.3, 30.0, 29.8, 26.8, 22.4, 21.6; HRMS (ESI-TOF) *m/z*: $[M + H]^+$ Calcd for $C_{27}H_{28}NO_2^+$ 398.2115; Found 398.2111.

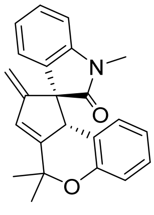

3e

Synthesis of 3e: To an oven-dried 10 mL Schlenk tube equipped with a magnetic stirring bar were added *tert*-butyl (2-methylhex-5-en-3-yn-2-yl) carbonate **1e** (31.6 mg, 0.150 mmol, 1.5 equiv), (*E*)-3-(2-hydroxybenzylidene)-1-methylindolin-2-one **2a** (25.1 mg, 0.0999 mmol, 1.0 equiv), Pd_2dba_3 (4.6 mg, 0.0050 mmol, 5 mol%), **L3** (9.8 mg, 0.012 mmol, 12 mol%) and $KHCO_3$ (12.0 mg, 0.120 mmol, 1.2 equiv). The tube was capped, evacuated and back-filled with argon for five times. Then degassed dry DMA (2.0 mL) was added via syringe. The mixture was stirred at 45 °C for 60 h, and monitored by TLC (EtOAc/petroleum ether = 1/8). After completion, the solvent was evaporated in vacuo. The residue was purified by flash chromatography on silica gel (EtOAc/petroleum ether = 1/8) to give the product **3e**: 23.1 mg (0.0672

mmol), as a white solid, 67% yield; mp 135–137 °C; >19:1 dr, determind by ^{1}H NMR analysis of crude product; $[\alpha_D^{25}]$ = –96.5 (c = 7.23 in $CHCl_3$); 92% ee, determined by HPLC analysis (Daicel Chiralpak AD, n-hexane/i-PrOH = 90/10, flow rate =1.0 mL/min, l = 254 nm) t_R = 5.07 min (minor), t_R = 6.70 min (major); ^{1}H NMR (400 MHz, $CDCl_3$): δ (ppm) 7.13–7.06 (m, 1H), 6.95–6.86 (m, 1H), 6.82 (d, J = 8.0 Hz, 1H), 6.76–6.70 (m, 1H), 6.70–6.64 (m, 1H), 6.64–6.56 (m, 1H), 6.47–6.40 (m, 1H), 6.23 (d, J = 2.8 Hz, 1H), 6.16 (d, J = 7.6 Hz, 1H), 4.97 (s, 1H), 4.78 (s, 1H), 4.56 (s, 1H), 3.40 (s, 3H), 1.74 (s, 3H), 1.48 (s, 3H); ^{13}C NMR (100 MHz, $CDCl_3$): δ (ppm) 178.6, 155.6, 153.2, 152.2, 142.6, 132.4, 127.8, 127.73, 127.70, 124.2, 122.9, 122.7, 121.0, 120.3, 117.4, 108.0, 105.5, 75.3, 63.0, 49.8, 26.8, 26.2, 24.9; HRMS (ESI-TOF) m/z: $[M + H]^+$ Calcd for $C_{23}H_{22}NO_2^+$ 344.1645; Found 344.1644.

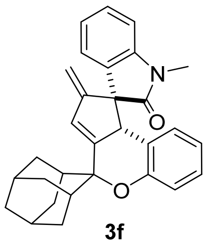

3f

Synthesis of 3f: To an oven-dried 10 mL Schlenk tube equipped with a magnetic stirring bar were added 2-(but-3-en-1-yn-1-yl)adamantan-2-yl *tert*-butyl carbonate **1f** (45.4 mg, 0.150 mmol, 1.5 equiv), (*E*)-3-(2-hydroxybenzylidene)-1-methyl indolin-2-one **2a** (25.1 mg, 0.0999 mmol, 1.0 equiv), $Pd(PPh_3)_4$ (5.8 mg, 0.0050 mmol, 5 mol%) and $KHCO_3$ (12.0 mg, 0.120 mmol, 1.2 equiv). The tube was capped, evacuated and back-filled with argon for five times. Then degassed dry DMA (2.0 mL) was added via syringe. The mixture was stirred at 60 °C for 24 h, and monitored by TLC (EtOAc/petroleum ether = 1/6). After completion, the solvent was evaporated in vacuo. The residue was purified by flash chromatography on silica gel (EtOAc/petroleum ether = 1/6) to give the product **3f**: 25.7 mg (0.0590 mmol), as a white solid, 59% yield; mp 188–190 °C; >19:1 dr, determind by ^{1}H NMR analysis of crude product; ^{1}H NMR (400 MHz, $CDCl_3$): δ (ppm) 7.11–7.02 (m, 1H), 6.95–6.87 (m, 1H), 6.85–6.74 (m, 2H), 6.69–6.61 (m, 1H), 6.59–6.53 (m, 1H), 6.48–6.39 (m, 2H), 6.15 (d, J = 8.0 Hz, 1H), 4.96 (s, 1H), 4.70 (s, 1H), 4.50 (s, 1H), 3.38 (s, 3H), 2.66–2.57 (m, 1H), 2.46–2.40 (m, 1H), 2.35–2.27 (m, 1H), 2.25–2.17 (m, 1H), 2.10–2.00 (m, 1H), 2.00–1.88 (m, 3H), 1.85–1.69 (m, 5H), 1.50–1.38 (m, 1H); ^{13}C NMR (100 MHz, $CDCl_3$): δ (ppm) 178.9, 156.0, 152.7, 151.4, 142.6, 132.8, 127.74, 127.68, 127.63, 126.7, 123.4, 123.3, 122.6, 120.5, 117.6, 107.8, 105.3, 80.9, 62.5, 51.0, 37.5, 35.4, 34.8, 33.8, 32.6, 31.8, 31.6, 27.3, 27.1, 26.7; HRMS (ESI-TOF) m/z: $[M + Na]^+$ Calcd for $C_{30}H_{29}NO_2Na^+$ 458.2091; Found 458.2101.

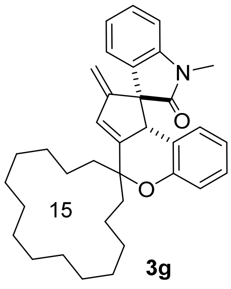

3g

Synthesis of 3g: To an oven-dried 10 mL Schlenk tube equipped with a magnetic stirring bar were added 1-(but-3-en-1-yn-1-yl)cyclopentadecyl *tert*-butyl carbonate **1g** (55.2 mg, 0.150 mmol, 1.5 equiv), (*E*)-3-(2-hydroxybenzylidene)-1-methyl indolin-2-one **2a** (25.1 mg, 0.0999 mmol, 1.0 equiv), $Pd(PPh_3)_4$ (5.8 mg, 0.0050 mmol, 5 mol%) and $KHCO_3$ (12.0 mg, 0.120 mmol, 1.2 equiv). The tube was capped, evacuated and back-filled with argon for five times. Then degassed dry DMA (2.0 mL) was added via syringe. The mixture was stirred at 60 °C for 24 h, and monitored by TLC (EtOAc/petroleum ether = 1/9). After completion, the solvent was evaporated in vacuo. The residue was purified by flash chromatography on silica gel (EtOAc/petroleum ether = 1/9) to give the product **3g**: 33.7 mg (0.0661 mmol), as a white solid, 66% yield; mp 115–117 °C; dr = 10:1, determind by ^{1}H NMR analysis of crude product; ^{1}H NMR (400 MHz, $CDCl_3$): δ (ppm) 7.10–7.05 (m, 1H), 6.92–6.87 (m, 1H), 6.80 (d, *J* = 8.0 Hz, 1H), 6.73 (d, *J* = 8.0 Hz, 1H), 6.68–6.61 (m, 2H), 6.45–6.39 (m, 1H), 6.26 (d, *J* = 2.8 Hz, 1H), 6.16 (d, *J* = 7.6 Hz, 1H), 4.96 (s, 1H), 4.69 (s, 1H), 4.55 (s, 1H), 3.39 (s, 3H), 2.06–1.72 (m, 4H), 1.52–1.25 (m, 24H); ^{13}C NMR (100 MHz, $CDCl_3$): δ (ppm) 178.6, 155.6, 153.1, 151.7, 142.6, 132.4, 127.7, 127.7, 127.5, 125.7, 122.9, 122.7, 121.4, 120.2, 117.3, 108.0, 105.2, 80.1, 62.9, 49.8, 35.2, 32.4, 27.8, 27.5, 27.0, 26.9, 26.8, 26.7, 26.6, 26.4, 26.3, 21.8, 21.0; HRMS (ESI-TOF) *m/z*: $[M + H]^+$ Calcd for $C_{35}H_{44}NO_2^+$ 510.3367; Found 510.3368.

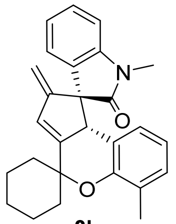
3h

Synthesis of 3h: To an oven-dried 10 mL Schlenk tube equipped with a magnetic stirring bar were added 1-(but-3-en-1-yn-1-yl)cyclohexyl *tert*-butyl carbonate **1a** (37.6 mg, 0.150 mmol, 1.5 equiv), (*E*)-3-(2-hydroxy-3-methylbenzylidene)-1-methylindolin-2-one **2b** (26.5 mg, 0.0999 mmol, 1.0 equiv), Pd_2dba_3 (4.6 mg, 0.0050 mmol, 5 mol%), **L3** (9.8 mg, 0.012 mmol, 12 mol%) and $KHCO_3$ (12.0 mg, 0.120 mmol, 1.2 equiv). The tube was capped, evacuated and back-filled with argon for five times. Then degassed dry DMA (2.0 mL) was added via syringe. The mixture was stirred at 45 °C for 60 h, and monitored by TLC (EtOAc/petroleum ether = 1/8). After completion, the solvent was evaporated in vacuo. The residue was purified by flash chromatography on silica gel (EtOAc/petroleum ether = 1/8) to give the product **3h**: 23.9 mg (0.0601 mmol), as a white solid, 60% yield; mp 151–153 °C; >19:1 dr, determind by ^{1}H NMR analysis of crude product; $[\alpha_D^{25}]$ = +96.5 (*c* = 5.22 in $CHCl_3$); 90% ee, determined by HPLC analysis (Daicel Chiralpak AD, *n*-hexane/*i*-PrOH = 90/10, flow rate =1.0

mL/min, l = 254 nm) t_R = 5.09 min (minor), t_R = 6.26 min (major); ^{1}H NMR (400 MHz, $CDCl_3$): δ (ppm) δ 7.14–7.03 (m, 1H), 6.84–6.73 (m, 2H), 6.71–6.57 (m, 2H), 6.34 (t, *J* = 7.2 Hz, 1H), 6.20 (d, *J* = 2.8 Hz, 1H), 6.01 (d, *J* = 7.6 Hz, 1H), 4.94 (s, 1H), 4.76 (s, 1H), 4.53 (s, 1H), 3.39 (s, 3H), 2.21 (s, 3H), 2.19–2.12 (m, 1H), 2.05–1.91 (m, 3H), 1.85–1.68 (m, 2H), 1.60–1.25 (m, 4H); ^{13}C NMR (100 MHz, $CDCl_3$): δ (ppm) 178.8, 155.9, 153.0, 151.0, 142.6, 132.6, 128.9, 127.7, 126.5, 125.1, 123.8, 122.9, 122.6, 121.1, 119.7, 108.0, 105.1, 75.9, 63.0, 50.0, 34.5, 32.5, 26.8, 25.7, 21.6, 16.3; HRMS (ESI-TOF) *m/z*: $[M + H]^+$ Calcd for $C_{27}H_{28}NO_2^+$ 398.2115; Found 398.2114.

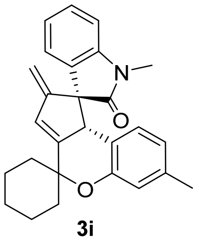

3i

Synthesis of 3i: To an oven-dried 10 mL Schlenk tube equipped with a magnetic stirring bar were added 1-(but-3-en-1-yn-1-yl)cyclohexyl *tert*-butyl carbonate **1a** (37.6 mg, 0.150 mmol, 1.5 equiv), (*E*)-3-(2-hydroxy-4-methylbenzylidene)-1-methylindolin-2-one **2c** (26.5 mg, 0.0999 mmol, 1.0 equiv), Pd_2dba_3 (4.6 mg, 0.0050 mmol, 5 mol%), **L3** (9.8 mg, 0.012 mmol, 12 mol%) and $KHCO_3$ (12.0 mg, 0.120 mmol, 1.2 equiv). The tube was capped, evacuated and back-filled with argon for five times. Then degassed dry DMA (2.0 mL) was added via syringe. The mixture was stirred at 45 °C for 60 h, and monitored by TLC (EtOAc/petroleum ether = 1/8). After completion, the solvent was evaporated in vacuo. The residue was purified by flash chromatography on silica gel (EtOAc/petroleum ether = 1/8) to give the product **3h**: 29.9 mg (0.0752 mmol), as a white solid, 75% yield; mp 155−157 °C; >19:1 dr, determind by ^{1}H NMR analysis of crude product; $[\alpha_D^{25}]$ = +84.3 (*c* = 5.87 in $CHCl_3$); 90% ee, determined by HPLC analysis (Daicel Chiralpak AD, *n*-hexane/*i*-PrOH = 90/10, flow rate =1.0 mL/min, l = 254 nm) t_R = 6.33 min (minor), t_R = 8.62 min (major); ^{1}H NMR (400 MHz, $CDCl_3$): δ (ppm) δ 7.14–7.05 (m, 1H), 6.80 (d, *J* = 7.6 Hz, 1H), 6.72–6.58 (m, 3H), 6.31–6.23 (m, 1H), 6.21 (d, *J* = 2.8 Hz, 1H), 6.04 (d, *J* = 8.0 Hz, 1H), 4.94 (s, 1H), 4.72 (s, 1H), 4.53 (s, 1H), 3.39 (s, 3H), 2.12 (s, 3H), 2.11–1.83 (m, 4H), 1.80–1.67 (m, 2H), 1.62–1.24 (m, 4H); ^{13}C NMR (100 MHz, $CDCl_3$): δ (ppm) 178.8, 155.9, 152.8, 152.7, 142.6, 137.6, 132.5, 127.7, 127.3, 124.3, 123.0, 122.7, 121.4, 118.7, 117.9, 107.9, 105.2, 76.0, 62.9, 49.7, 34.4, 32.6, 26.7, 25.6, 21.7, 21.3, 21.1; HRMS (ESI-TOF) *m/z*: $[M + H]^+$ Calcd for $C_{27}H_{28}NO_2^+$ 398.2115; Found 398.2111.

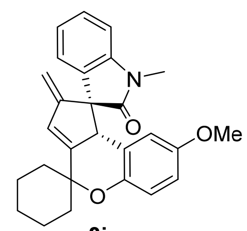

3j

Synthesis of 3j: To an oven-dried 10 mL Schlenk tube equipped with a magnetic stirring bar were added 1-(but-3-en-1-yn-1-yl)cyclohexyl *tert*-butyl carbonate **1a** (37.6 mg, 0.150 mmol, 1.5 equiv), (*E*)-3-(2-hydroxy-5-methoxy benzylidene) -1-methylindolin-2-one **2d** (28.1 mg, 0.0999 mmol, 1.0 equiv), Pd_2dba_3 (4.6 mg, 0.0050 mmol, 5 mol%), **L3** (9.8 mg, 0.012 mmol, 12 mol%) and $KHCO_3$ (12.0 mg, 0.120 mmol, 1.2 equiv). The tube was capped, evacuated and back-filled with argon for five times. Then degassed dry DMA (2.0 mL) was added via syringe. The mixture was stirred at 45 °C for 60 h, and monitored by TLC (EtOAc/petroleum ether = 1/8). After completion, the solvent was evaporated in vacuo. The residue was purified by flash chromatography on silica gel (EtOAc/petroleum ether = 1/8) to give the product **3j**: 29.8 mg (0.0721 mmol), as a white solid, 72% yield; mp 167–169 °C; >19:1 dr, determind by ^{1}H NMR analysis of crude product; $[\alpha_D^{25}]$ = +142.2 (*c* = 6.34 in $CHCl_3$); 90% ee, determined by HPLC analysis (Daicel Chiralpak IA, *n*-hexane/*i*-PrOH = 90/10, flow rate =1.0 mL/min, l = 254 nm) t_R = 8.19 min (minor), t_R = 12.70 min (major); ^{1}H NMR (400 MHz, $CDCl_3$): δ (ppm) 7.1–7.07 (m, 1H), 6.81 (d, *J* = 7.6 Hz, 1H), 6.74–6.65 (m, 3H), 6.52–6.47 (m, 1H), 6.23 (d, *J* = 2.8 Hz, 1H), 5.71 (d, *J* = 3.4 Hz, 1H), 4.97 (s, 1H), 4.71 (s, 1H), 4.56 (s, 1H), 3.39 (s, 3H), 3.36 (s, 3H), 2.15–1.89 (m, 4H), 1.79–1.64 (m, 2H), 1.57–1.33 (m, 4H); ^{13}C NMR (100 MHz, $CDCl_3$): δ (ppm) 178.7, 155.6, 153.2, 152.6, 147.0, 142.6, 132.4, 127.9, 124.5, 123.0, 122.9, 122.3, 118.2, 114.8, 111.4, 107.9, 105.4, 75.9, 62.9, 55.4, 50.4, 34.4, 32.3, 26.8, 25.6, 21.7, 21.3; HRMS (ESI-TOF) *m/z*: $[M + H]^+$ Calcd for $C_{27}H_{28}NO_3^+$ 414.2064; Found 414.2069.

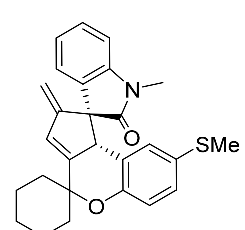

3k

Synthesis of 3k: To an oven-dried 10 mL Schlenk tube equipped with a magnetic stirring bar were added 1-(but-3-en-1-yn-1-yl)cyclohexyl *tert*-butyl carbonate **1a** (37.6 mg, 0.150 mmol, 1.5 equiv), (*E*)-3-(2-hydroxy-5-(methyl thio)benzylidene)-1-methylindolin-2-one **2e** (29.7 mg, 0.0999 mmol, 1.0 equiv), Pd_2dba_3 (4.6 mg, 0.0050 mmol, 5 mol%), **L3** (9.8 mg, 0.012 mmol, 12 mol%) and $KHCO_3$ (12.0 mg, 0.120 mmol, 1.2 equiv). The tube was capped, evacuated and back-filled with argon for five times. Then degassed dry DMA (2.0 mL) was added via syringe. The mixture was stirred at 45 °C for 60 h, and monitored by TLC (EtOAc/petroleum ether = 1/8). After completion, the solvent was evaporated in vacuo. The residue was purified by flash chromatography on silica gel (EtOAc/petroleum ether = 1/8) to give the product **3k**: 34.0 mg (0.0791 mmol), as a white solid, 79%

yield; mp 211–213 °C; >19:1 dr, determind by ^{1}H NMR analysis of crude product; $[\alpha_D^{25}]$ = −131.7 (*c* = 5.81 in $CHCl_3$); 89% ee, determined by HPLC analysis (Daicel Chiralpak IB, *n*-hexane/*i*-PrOH = 95/5, flow rate =1.0 mL/min, l = 254 nm) t_R = 6.27 min (minor), t_R = 7.28 min (major); ^{1}H NMR (400 MHz, $CDCl_3$): δ (ppm) 7.14–7.05 (m, 1H), 6.94–6.88 (m, 1H), 6.82 (d, *J* = 7.6 Hz, 1H), 6.73–6.65 (m, 2H), 6.63–6.58 (m, 1H), 6.25 (d, *J* = 2.8 Hz, 1H), 6.19–6.10 (m, 1H), 4.98 (s, 1H), 4.72 (s, 1H), 4.56 (s, 1H), 3.40 (s, 3H), 2.15–2.00 (m, 2H), 1.99 (s, 3H), 1.98–1.89 (m, 2H), 1.81–1.67 (m, 2H), 1.59–1.27 (m, 4H); ^{13}C NMR (100 MHz, $CDCl_3$): δ (ppm) 178.6, 155.4, 151.9, 151.7, 142.6, 132.3, 129.3, 128.6, 128.2, 127.9, 124.9, 123.1, 122.9, 122.6, 118.0, 108.0, 105.8, 76.4, 62.7, 50.0, 34.3, 32.6, 26.8, 25.6, 21.6, 21.3, 18.1; HRMS (ESI-TOF) *m/z*: $[M + Na]^+$ Calcd for $C_{27}H_{27}NO_2SNa^+$ 452.1655; Found 452.1648.

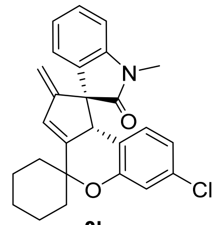

3l

Synthesis of 3l: To an oven-dried 10 mL Schlenk tube equipped with a magnetic stirring bar were added 1-(but-3-en-1-yn-1-yl)cyclohexyl *tert*-butyl carbonate **1a** (37.6 mg, 0.150 mmol, 1.5 equiv), (*E*)-3-(4-chloro-2-hydroxybenzylidene)-1-methylindolin-2-one **2f** (28.6 mg, 0.100 mmol, 1.0 equiv), Pd_2dba_3 (4.6 mg, 0.0050 mmol, 5 mol%), **L3** (9.8 mg, 0.012 mmol, 12 mol%) and $KHCO_3$ (12.0 mg, 0.120 mmol, 1.2 equiv). The tube was capped, evacuated and back-filled with argon for five times. Then degassed dry DMA (2.0 mL) was added via syringe. The mixture was stirred at 45 °C for 60 h, and monitored by TLC (EtOAc/petroleum ether = 1/8). After completion, the solvent was evaporated in vacuo. The residue was purified by flash chromatography on silica gel (EtOAc/petroleum ether = 1/8) to give the product **3l**: 30.6 mg (0.0732 mmol), as a white solid, 73% yield; mp 176–178 °C; >19:1 dr, determind by ^{1}H NMR analysis of crude product; $[\alpha_D^{25}]$ = −73.4 (*c* = 6.72 in $CHCl_3$); 88% ee, determined by HPLC analysis (Daicel Chiralpak AD, *n*-hexane/*i*-PrOH = 90/10, flow rate =1.0 mL/min, l = 254 nm) t_R = 5.75 min (minor), t_R = 6.88 min (major); ^{1}H NMR (400 MHz, $CDCl_3$): δ (ppm) 7.15–7.04 (m, 1H), 6.90–6.76 (m, 2H), 6.74–6.66 (m, 1H), 6.64–6.58 (m, 1H), 6.48–6.37 (m, 1H), 6.24 (d, *J* = 2.8 Hz, 1H), 6.07 (d, *J* = 8.4 Hz, 1H), 4.97 (s, 1H), 4.70 (s, 1H), 4.55 (s, 1H), 3.39 (s, 3H), 2.18–2.05 (m, 1H), 2.04–1.90 (m, 3H), 1.81–1.67 (m, 2H), 1.58–1.29 (m, 4H); ^{13}C NMR (100 MHz, $CDCl_3$): δ (ppm) 178.5, 155.5, 153.7, 151.5, 142.5, 132.7, 132.1, 128.5, 128.0, 124.9, 122.93, 122.91, 120.6, 120.4, 117.8, 108.1, 105.8, 62.7, 49.4, 34.3, 32.6, 26.8, 25.5, 21.6, 21.3; HRMS (ESI-TOF) *m/z*: $C_{26}H_{24}{}^{35}ClNO_2Na^+$ 440.1388; Found 440.1398; $[M + Na]^+$ Calcd for $C_{26}H_{24}{}^{37}ClNO_2Na^+$

442.1358; Found 442.1350.

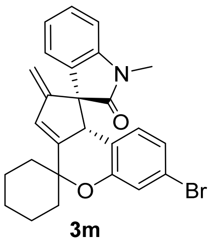

3m

Synthesis of 3m: To an oven-dried 10 mL Schlenk tube equipped with a magnetic stirring bar were added 1-(but-3-en-1-yn-1-yl)cyclohexyl *tert*-butyl carbonate **1a** (37.6 mg, 0.150 mmol, 1.5 equiv), (*E*)-3-(4-bromo-2-hydroxybenzylidene)-1-methylindolin-2-one **2g** (33.0 mg, 0.0999 mmol, 1.0 equiv), Pd_2dba_3 (4.6 mg, 0.0050 mmol, 5 mol%), **L3** (9.8 mg, 0.012 mmol, 12 mol%) and $KHCO_3$ (12.0 mg, 0.120 mmol, 1.2 equiv). The tube was capped, evacuated and back-filled with argon for five times. Then degassed dry DMA (2.0 mL) was added via syringe. The mixture was stirred at 45 °C for 60 h, and monitored by TLC (EtOAc/petroleum ether = 1/8). After completion, the solvent was evaporated in vacuo. The residue was purified by flash chromatography on silica gel (EtOAc/petroleum ether = 1/8) to give the product **3m**: 33.9 mg (0.0733 mmol), as a white solid, 73% yield; mp 144–146 °C; >19:1 dr, determind by ^{1}H NMR analysis of crude product; $[\alpha_D^{25}]$ = −174.8 (*c* = 8.32 in $CHCl_3$); 85% ee, determined by HPLC analysis (Daicel Chiralpak AD, *n*-hexane/*i*-PrOH = 90/10, flow rate =1.0 mL/min, l = 254 nm) t_R = 6.19 min (minor), t_R = 8.25 min (major); ^{1}H NMR (400 MHz, $CDCl_3$): δ (ppm) 7.16–7.08 (m, 1H), 6.98 (d, *J* = 2.0 Hz, 1H), 6.82 (d, *J* = 8.0 Hz, 1H), 6.74–6.68 (m, 1H), 6.63–6.59 (m, 1H), 6.59–6.54 (m, 1H), 6.24 (d, *J* = 2.8 Hz, 1H), 6.01 (d, *J* = 8.4 Hz, 1H), 4.97 (s, 1H), 4.68 (s, 1H), 4.55 (s, 1H), 3.39 (s, 3H), 2.13–2.06 (m, 1H), 2.03–1.90 (m, 3H), 1.78–1.68 (m, 2H), 1.61–1.35 (m, 4H); ^{13}C NMR (100 MHz, $CDCl_3$): δ (ppm) 178.5, 155.5, 153.8, 151.4, 142.5, 132.1, 128.8, 128.0, 124.9, 123.5, 123.0, 122.9, 120.9, 120.7, 120.5, 108.1, 105.8, 62.6, 49.4, 34.3, 32.7, 26.8, 25.5, 21.4, 21.3; HRMS (ESI-TOF) *m/z*: $[M + H]^+$ Calcd for $C_{26}H_{25}{}^{79}BrNO_2^+$ 462.1063; Found 462.1060; Calcd for $C_{26}H_{25}{}^{81}BrNO_2^+$ 464.1043; Found 464.1046.

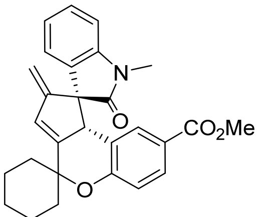

3n

Synthesis of 3n: To an oven-dried 10 mL Schlenk tube equipped with a magnetic stirring bar were added 1-(but-3-en-1-yn-1-yl)cyclohexyl *tert*-butyl carbonate **1a** (37.6 mg, 0.150 mmol, 1.5 equiv), methyl (*E*)-4-hydroxy -3-((1-methyl-2-oxoindolin-3-ylidene)methyl)benzoate **2h** (30.9 mg, 0.0999 mmol, 1.0 equiv), Pd_2dba_3 (4.6 mg, 0.0050 mmol, 5 mol%), **L3** (9.8 mg, 0.012 mmol, 12 mol%) and $KHCO_3$ (12.0 mg, 0.120 mmol, 1.2 equiv). The tube was capped, evacuated and back-filled with argon for five times. Then degassed dry DMA (2.0 mL) was added via syringe. The mixture was stirred at 45 °C for 60 h, and monitored by TLC (EtOAc/petroleum ether = 1/6). After completion,

the solvent was evaporated in vacuo. The residue was purified by flash chromatography on silica gel (EtOAc/petroleum ether = 1/6) to give the product **3n**: 23.5 mg (0.0532 mmol), as a white solid, 53% yield; mp 211–213 °C; >19:1 dr, determind by ^{1}H NMR analysis of crude product; $[\alpha_D^{25}]$ = +82.6 (*c* = 4.25 in $CHCl_3$); 91% ee, determined by HPLC analysis (Daicel Chiralpak IB, *n*-hexane/*i*-PrOH = 90/10, flow rate =1.0 mL/min, l = 254 nm) t_R = 6.85 min (minor), t_R = 8.02 min (major); ^{1}H NMR (400 MHz, $CDCl_3$): δ (ppm) 7.58 (d, *J* = 8.4 Hz, 1H), 7.07–7.03 (m, 1H), 6.93 (s, 1H), 6.85–6.76 (m, 2H), 6.67–6.55 (m, 2H), 6.29 (d, *J* = 2.0 Hz, 1H), 5.02 (s, 1H), 4.75 (s, 1H), 4.62 (s, 1H), 3.71 (s, 3H), 3.43 (s, 3H), 2.16–2.07 (m, 1H), 2.07–1.93 (m, 3H), 1.80–1.69 (m, 2H), 1.60–1.30 (m, 4H); ^{13}C NMR (100 MHz, $CDCl_3$): δ (ppm) 178.3, 166.3, 157.0, 154.9, 150.9, 142.7, 132.0, 130.0, 129.4, 128.1, 125.4, 122.7, 122.5, 122.0, 121.4, 117.3, 108.2, 106.3, 63.0, 51.6, 50.0, 34.2, 32.9, 26.8, 25.5, 21.6, 21.3; HRMS (ESI-TOF) *m/z*: $[M + Na]^+$ Calcd for $C_{28}H_{27}NO_4Na^+$ 464.1832; Found 464.1836.

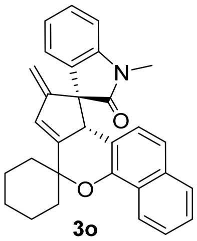
3o

Synthesis of 3o: To an oven-dried 10 mL Schlenk tube equipped with a magnetic stirring bar were added 1-(but-3-en-1-yn-1-yl)cyclohexyl *tert*-butyl carbonate **1a** (37.6 mg, 0.150 mmol, 1.5 equiv), (*E*)-3-((1-hydroxynaphthalen-2-yl)methylene)-1-methyl indolin-2-one **2i** (30.1 mg, 0.0999 mmol, 1.0 equiv), Pd_2dba_3 (4.6 mg, 0.0050 mmol, 5 mol%), **L3** (9.8 mg, 0.012 mmol, 12 mol%) and $KHCO_3$ (12.0 mg, 0.120 mmol, 1.2 equiv). The tube was capped, evacuated and back-filled with argon for five times. Then degassed dry DMA (2.0 mL) was added via syringe. The mixture was stirred at 45 °C for 60 h, and monitored by TLC (EtOAc/petroleum ether = 1/7). After completion, the solvent was evaporated in vacuo. The residue was purified by flash chromatography on silica gel (EtOAc/petroleum ether = 1/7) to give the product **3o**: 26.5 mg (0.0611 mmol), as a white solid, 61% yield; mp 193−195 °C; >19:1 dr, determind by ^{1}H NMR analysis of crude product; $[\alpha_D^{25}]$ = −212.6 (*c* = 7.32 in $CHCl_3$); 97% ee, determined by HPLC analysis (Daicel Chiralpak IF, *n*-hexane/*i*-PrOH = 90/10, flow rate =1.0 mL/min, l = 254 nm) t_R = 9.75 min (minor), t_R = 13.43 min (major); ^{1}H NMR (400 MHz, $CDCl_3$): δ (ppm) 7.49–7.44 (m, 1H), 7.42 (d, *J* = 8.8 Hz, 1H), 7.35 (d, *J* = 8.4 Hz, 1H), 7.19–7.11 (m, 1H), 7.11–7.05 (m, 1H), 7.01 (d, *J* = 8.8 Hz, 1H), 6.81–6.70 (m, 1H), 6.56–6.45 (m, 1H), 6.44–6.36 (m, 2H), 6.34 (d, *J* = 2.8 Hz, 1H), 5.17 (s, 1H), 4.96 (s, 1H), 4.56 (s, 1H), 3.27 (s, 3H), 2.26–2.14 (m, 1H), 2.14–1.96 (m, 3H), 1.86–1.65 (m, 2H), 1.62–1.35 (m, 4H); ^{13}C NMR (100 MHz, $CDCl_3$): δ (ppm) 179.7, 156.0, 153.4, 151.8, 142.2, 132.9, 131.9, 128.9, 128.6, 128.0, 127.3, 125.1, 125.0, 122.9, 122.8, 122.0, 121.9, 119.2,

114.9, 107.6, 105.2, 63.7, 48.6, 34.4, 30.7, 26.9, 25.7, 22.0, 21.5; HRMS (ESI-TOF) *m/z*: $[M + H]^+$ Calcd for $C_{30}H_{28}NO_2^+$ 434.2115; Found 434.2113.

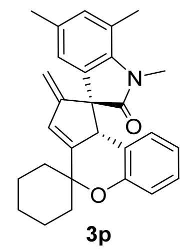
3p

Synthesis of 3p: To an oven-dried 10 mL Schlenk tube equipped with a magnetic stirring bar were added 1-(but-3-en-1-yn-1-yl)cyclohexyl *tert*-butyl carbonate **1a** (37.6 mg, 0.150 mmol, 1.5 equiv), (*E*)-3-(2-hydroxybenzylidene)-1,5,7-trimethyl indolin-2-one **2j** (27.9 mg, 0.0999 mmol, 1.0 equiv), Pd_2dba_3 (4.6 mg, 0.0050 mmol, 5 mol%), **L3** (9.8 mg, 0.012 mmol, 12 mol%) and $KHCO_3$ (12.0 mg, 0.120 mmol, 1.2 equiv). The tube was capped, evacuated and back-filled with argon for five times. Then degassed dry DMA (2.0 mL) was added via syringe. The mixture was stirred at 45 °C for 60 h, and monitored by TLC (EtOAc/petroleum ether = 1/7). After completion, the solvent was evaporated in vacuo. The residue was purified by flash chromatography on silica gel (EtOAc/petroleum ether = 1/7) to give the product **3p**: 29.4 mg (0.0714 mmol), as a white solid, 71% yield; mp 167−168 °C; >19:1 dr, determind by ^{1}H NMR analysis of crude product; $[\alpha_D^{25}]$ = −132.5 (*c* = 5.43 in $CHCl_3$); 82% ee, determined by HPLC analysis (Daicel Chiralpak AD, *n*-hexane/*i*-PrOH = 90/10, flow rate =1.0 mL/min, l = 254 nm) t_R = 5.01 min (minor), t_R = 6.70 min (major); ^{1}H NMR (400 MHz, $CDCl_3$): δ (ppm) δ 6.95–6.86 (m, 1H), 6.82–6.75 (m, 1H), 6.60 (s, 1H), 6.52–6.42 (m, 1H), 6.24 (s, 1H), 6.21 (d, *J* = 2.8 Hz, 1H), 6.18 (d, *J* = 7.6 Hz, 1H), 4.95 (s, 1H), 4.73 (s, 1H), 4.54 (s, 1H), 3.64 (s, 3H), 2.52 (s, 3H), 2.16–2.06 (m, 1H), 2.05–1.94 (m, 3H), 1.93 (s, 3H), 1.83–1.67 (m, 2H), 1.63–1.28 (m, 4H); ^{13}C NMR (100 MHz, $CDCl_3$): δ (ppm) 179.5, 156.5, 153.0, 152.5, 138.0, 133.1, 131.99, 131.96, 127.8, 127.5, 124.4, 121.9, 121.6, 120.3, 119.0, 117.3, 105.2, 76.1, 62.3, 50.2, 34.4, 32.6, 30.1, 25.7, 21.7, 21.4, 20.6, 19.0; HRMS (ESI-TOF) *m/z*: $[M + H]^+$ Calcd for $C_{28}H_{30}NO_2^+$ 412.2271; Found 412.2270.

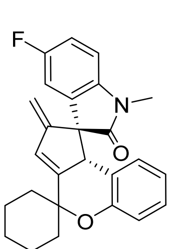
3q

Synthesis of 3q: To an oven-dried 10 mL Schlenk tube equipped with a magnetic stirring bar were added 1-(but-3-en-1-yn-1-yl)cyclohexyl *tert*-butyl carbonate **1a** (37.6 mg, 0.150 mmol, 1.5 equiv), (*E*)-5-fluoro-3-(2-hydroxybenzylidene)-1-methyl indolin-2-one **2k** (26.9 mg, 0.0999 mmol, 1.0 equiv), Pd_2dba_3 (4.6 mg, 0.0050 mmol, 5 mol%), **L3** (9.8 mg, 0.012 mmol, 12 mol%) and $KHCO_3$ (12.0 mg, 0.120 mmol, 1.2 equiv). The tube was capped, evacuated and back-filled with argon for five times. Then degassed dry DMA (2.0 mL) was added via syringe. The mixture was stirred at 45 °C for 60 h, and monitored by TLC (EtOAc/petroleum ether = 1/8). After completion, the solvent was evaporated in vacuo. The

residue was purified by flash chromatography on silica gel (EtOAc/petroleum ether = 1/8) to give the product **3q**: 29.4 mg (0.0732 mmol), as a white solid, 73% yield; mp 133−135 °C; >19:1 dr, determind by ^{1}H NMR analysis of crude product; $[\alpha_D^{25}]$ = −89.5 (*c* = 8.32 in $CHCl_3$); 92% ee, determined by HPLC analysis (Daicel Chiralpak IA, *n*-hexane/*i*-PrOH = 90/10, flow rate =1.0 mL/min, l = 254 nm) t_R = 5.90 min (minor), t_R = 6.77 min (major); ^{1}H NMR (400 MHz, $CDCl_3$): δ (ppm) 6.97–6.90 (m, 1H), 6.83–6.75 (m, 2H), 6.74–6.69 (m, 1H), 6.51–6.44 (m, 1H), 6.43–6.35 (m, 1H), 6.22 (d, *J* = 3.2 Hz, 1H), 6.18 (d, *J* = 7.6 Hz, 1H), 4.97 (s, 1H), 4.76 (s, 1H), 4.55 (s, 1H), 3.38 (s, 3H), 2.19–2.04 (m, 1H), 2.04–1.90 (m, 3H), 1.79–1.68 (m, 2H), 1.60–1.24 (m, 4H); ^{13}C NMR (100 MHz, $CDCl_3$): δ (ppm) 178.3, 159.1 (d, $^1J_{FC}$ = 239.5 Hz), 155.2, 153.0, 152.6, 138.6 (d, *J* = 1.9 Hz), 134.0 (d, $^3J_{FC}$ = 8.6 Hz), 128.0, 127.3, 124.3, 121.4, 120.5, 117.7, 114.1 (d, $^2J_{FC}$ = 23.6 Hz), 111.0 (d, $^1J_{FC}$ = 24.9 Hz), 108.4 (d, *J* = 8.2 Hz), 105.7, 76.1, 63.2, 49.8, 34.4, 32.5, 26.9, 25.6, 21.6, 21.3; ^{19}F NMR (375 MHz, $CDCl_3$): δ (ppm) −120.2; HRMS (ESI-TOF) *m/z*: $[M + H]^+$ Calcd for $C_{26}H_{25}FNO_2^+$ 402.1864; Found 402.1865.

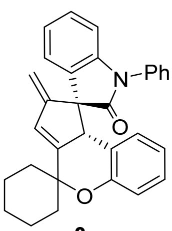

3r

Synthesis of 3r: To an oven-dried 10 mL Schlenk tube equipped with a magnetic stirring bar were added 1-(but-3-en-1-yn-1-yl)cyclohexyl *tert*-butyl carbonate **1a** (37.6 mg, 0.150 mmol, 1.5 equiv), (*E*)-3-(2-hydroxybenzylidene)-1-phenylindolin-2-one **2l** (31.4 mg, 0.0100 mmol, 1.0 equiv), Pd_2dba_3 (4.6 mg, 0.0050 mmol, 5 mol%), **L3** (9.8 mg, 0.012 mmol, 12 mol%) and $KHCO_3$ (12.0 mg, 0.120 mmol, 1.2 equiv). The tube was capped, evacuated and back-filled with argon for five times. Then degassed dry DMA (2.0 mL) was added via syringe. The mixture was stirred at 45 °C for 60 h, and monitored by TLC (EtOAc/petroleum ether = 1/8). After completion, the solvent was evaporated in vacuo. The residue was purified by flash chromatography on silica gel (EtOAc/petroleum ether = 1/8) to give the product **3r**: 27.7 mg (0.0622 mmol), as a white solid, 62% yield; mp 189−191 °C; >19:1 dr, determind by ^{1}H NMR analysis of crude product; $[\alpha_D^{25}]$ = −132.4 (*c* = 6.43 in $CHCl_3$); 91% ee, determined by HPLC analysis (Daicel Chiralpak IB, *n*-hexane/*i*-PrOH = 90/10, flow rate =1.0 mL/min, l = 254 nm) t_R = 7.09 min (minor), t_R = 8.68 min (major); ^{1}H NMR (400 MHz, $CDCl_3$): δ (ppm) 7.69–7.50 (m, 4H), 7.50–7.41 (m, 1H), 7.03–6.97 (m, 1H), 6.97–6.91 (m, 1H), 6.86–6.80 (m, 1H), 6.80–6.75 (m, 1H), 6.74–6.61 (m, 2H), 6.57–6.48 (m, 1H), 6.46–6.36 (m, 1H), 6.27 (d, *J* = 2.8 Hz, 1H), 5.04 (s, 1H), 4.86 (s, 1H), 4.75 (s, 1H), 2.20–2.09 (m, 1H), 2.08–1.92 (m, 3H), 1.84–1.67 (m, 2H), 1.58–1.30 (m, 4H); ^{13}C NMR (100 MHz, $CDCl_3$): δ (ppm) 178.0, 156.0, 153.1, 152.5, 142.5, 134.7, 132.2, 129.8, 128.2,

127.8, 127.63, 127.62, 126.5, 124.4, 123.2, 123.1, 121.8, 120.5, 117.6, 109.4, 105.6, 76.2, 63.1, 50.3, 34.4, 32.5, 25.6, 21.7, 21.3; HRMS (ESI-TOF) *m/z*: $[M + Na]^+$ Calcd for $C_{31}H_{28}NO_2^+$ 446.2115; Found 446.2112.

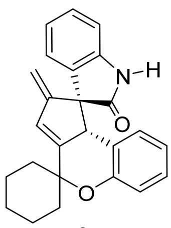
3s

Synthesis of 3s: To an oven-dried 10 mL Schlenk tube equipped with a magnetic stirring bar were added 1-(but-3-en-1-yn-1-yl)cyclohexyl *tert*-butyl carbonate **1a** (37.6 mg, 0.150 mmol, 1.5 equiv), (*E*)-3-(2-hydroxybenzylidene)indolin-2-one **2m** (23.7 mg, 0.0999 mmol, 1.0 equiv), Pd_2dba_3 (4.6 mg, 0.0050 mmol, 5 mol%), **L3** (9.8 mg, 0.012 mmol, 12 mol%) and $KHCO_3$ (12.0 mg, 0.120 mmol, 1.2 equiv). The tube was capped, evacuated and back-filled with argon for five times. Then degassed dry DMA (2.0 mL) was added via syringe. The mixture was stirred at 45 °C for 60 h, and monitored by TLC (EtOAc/petroleum ether = 1/8). After completion, the solvent was evaporated in vacuo. The residue was purified by flash chromatography on silica gel (EtOAc/petroleum ether = 1/8) to give the product **3s**: 26.3 mg (0.0712 mmol), as a white solid, 71% yield; mp 151–153 °C; >19:1 dr, determind by ^{1}H NMR analysis of crude product; $[\alpha_D^{25}]$ = –231.5 (*c* = 4.34 in $CHCl_3$); 88% ee, determined by HPLC analysis (Daicel Chiralpak AD, *n*-hexane/*i*-PrOH = 80/20, flow rate =1.0 mL/min, l = 254 nm) t_R = 5.08 min (minor), t_R = 9.23 min (major); ^{1}H NMR (400 MHz, $CDCl_3$): δ (ppm) 9.40–8.81 (m, 1H), 7.04–6.98 (m, 1H), 6.96–6.85 (m, 2H), 6.81 (d, *J* = 8.0 Hz, 1H), 6.68–6.57 (m, 2H), 6.52–6.44 (m, 1H), 6.38 (d, *J* = 7.6 Hz, 1H), 6.26 (d, *J* = 2.8 Hz, 1H), 5.02 (s, 1H), 4.80 (s, 1H), 4.73 (s, 1H), 2.19–2.08 (m, 1H), 2.06–1.93 (m, 3H), 1.77–1.39 (m, 6H); ^{13}C NMR (100 MHz, $CDCl_3$): δ (ppm) 155.7, 153.0, 152.6, 139.8, 133.0, 127.79, 127.77, 124.4, 123.3, 122.6, 121.7, 120.5, 117.5, 110.0, 105.6, 76.1, 63.4, 49.9, 34.4, 32.6, 25.6, 21.7, 21.4; HRMS (ESI-TOF) *m/z*: $[M + H]^+$ Calcd for $C_{25}H_{24}NO_2^+$ 370.1802; Found 370.1804.

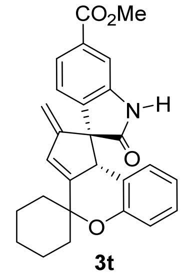
3t

Synthesis of 3t: To an oven-dried 10 mL Schlenk tube equipped with a magnetic stirring bar were added 1-(but-3-en-1-yn-1-yl)cyclohexyl *tert*-butyl carbonate **1a** (37.6 mg, 0.150 mmol, 1.5 equiv), methyl (*E*)-3-(2-hydroxybenzylidene)-2-oxo indoline-6-carboxylate **2n** (29.5 mg, 0.0999 mmol, 1.0 equiv), Pd_2dba_3 (4.6 mg, 0.0050 mmol, 5 mol%), **L3** (9.8 mg, 0.012 mmol, 12 mol%) and $KHCO_3$ (12.0 mg, 0.120 mmol, 1.2 equiv). The tube was capped, evacuated and back-filled with argon for five times. Then degassed dry DMA (2.0 mL) was added via syringe. The mixture was stirred at 45 °C for 60 h,

and monitored by TLC (EtOAc/petroleum ether = 1/5). After completion, the solvent was evaporated in vacuo. The residue was purified by flash chromatography on silica gel (EtOAc/petroleum ether = 1/5) to give the product **3t**: 27.0 mg (0.0632 mmol), as a white solid, 63% yield; mp 175−177 °C; >19:1 dr, determind by ^{1}H NMR analysis of crude product; [α_D^{25}] = −176.2 (*c* = 6.22 in $CHCl_3$); 96% ee, determined by HPLC analysis (Daicel Chiralpak IB, *n*-hexane/*i*-PrOH = 95/5, flow rate =1.0 mL/min, l = 254 nm) t_R = 15.80 min (major), t_R = 20.27 min (minor); ^{1}H NMR (400 MHz, $CDCl_3$): δ (ppm) 8.62 (s, 1H), 7.55 (s, 1H), 7.42–7.32 (m, 1H), 6.96–6.89 (m, 1H), 6.81 (d, *J* = 8.0 Hz, 1H), 6.67 (d, *J* = 7.6 Hz, 1H), 6.51–6.43 (m, 1H), 6.35 (d, *J* = 7.6 Hz, 1H), 6.25 (d, *J* = 2.4 Hz, 1H), 5.02 (s, 1H), 4.83 (s, 1H), 4.71 (s, 1H), 3.84 (s, 3H), 2.16–1.94 (m, 4H), 1.79–1.67 (m, 2H), 1.61–1.27 (m, 4H); ^{13}C NMR (150 MHz, $CDCl_3$): δ (ppm) 180.6, 166.5, 155.0, 152.9, 152.6, 139.8, 138.0, 129.8, 128.0, 127.5, 124.5, 124.2, 123.2, 121.3, 120.7, 117.6, 110.6, 106.0, 76.2, 63.3, 52.1, 49.9, 34.4, 32.5, 25.6, 21.6, 21.3; HRMS (ESI-TOF) *m/z*: [M + Na]$^{+}$ Calcd for $C_{27}H_{26}NO_4^{+}$ 428.1856; Found 428.1853.

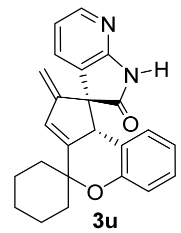

3u

Synthesis of 3u: To an oven-dried 10 mL Schlenk tube equipped with a magnetic stirring bar were added 1-(but-3-en-1-yn-1-yl)cyclohexyl *tert*-butyl carbonate **1a** (37.6 mg, 0.150 mmol, 1.5 equiv), (*E*)-3-(2-hydroxybenzylidene)-1,3-dihydro-2*H*-pyrrolo[2,3-*b*]pyridin-2-one **2o** (23.8 mg, 0.0999 mmol, 1.0 equiv), Pd_2dba_3 (4.6 mg, 0.0050 mmol, 5 mol%), **L3** (9.8 mg, 0.012 mmol, 12 mol%) and $KHCO_3$ (12.0 mg, 0.120 mmol, 1.2 equiv). The tube was capped, evacuated and back-filled with argon for five times. Then degassed dry DMA (2.0 mL) was added via syringe. The mixture was stirred at 45 °C for 60 h, and monitored by TLC (EtOAc/petroleum ether = 1/5). After completion, the solvent was evaporated in vacuo. The residue was purified by flash chromatography on silica gel (EtOAc/petroleum ether = 1/5) to give the product **3u**: 24.5 mg (0.0661 mmol), as a white solid, 66% yield; mp 193−195 °C; >19:1 dr, determind by ^{1}H NMR analysis of crude product; [α_D^{25}] = −95.3 (*c* = 6.12 in $CHCl_3$); 93% ee, determined by HPLC analysis (Daicel Chiralpak AD, *n*-hexane/*i*-PrOH = 80/20, flow rate =1.0 mL/min, l = 254 nm) t_R = 6.63 min (minor), t_R = 9.88 min (major); ^{1}H NMR (400 MHz, $CDCl_3$): δ (ppm) 10.31 (s, 1H), 8.04 (d, *J* = 5.2 Hz, 1H), 6.99–6.94 (m, 1H), 6.89–6.85 (m, 1H), 6.82 (d, *J* = 8.0 Hz, 1H), 6.62–6.58 (m, 1H), 6.57–6.53 (m, 1H), 6.44 (d, *J* = 7.6 Hz, 1H), 6.23 (d, *J* = 2.8 Hz, 1H), 5.03 (s, 1H), 4.83 (s, 1H), 4.79 (s, 1H), 2.11–1.94 (m, 4H), 1.73–1.35 (m, 6H); ^{13}C NMR (100 MHz, $CDCl_3$): δ (ppm) 179.4, 155.5, 154.8, 152.9, 152.4, 146.2, 131.1, 128.1, 127.64, 127.60, 124.2, 121.3, 120.8, 118.2,

117.7, 106.1, 76.2, 63.2, 49.5, 34.3, 32.3, 25.6, 21.7, 21.3; HRMS (ESI-TOF) *m/z*: [M + H]$^+$ Calcd for $C_{24}H_{23}N_2O_2^+$ 371.1754; Found 371.1756.

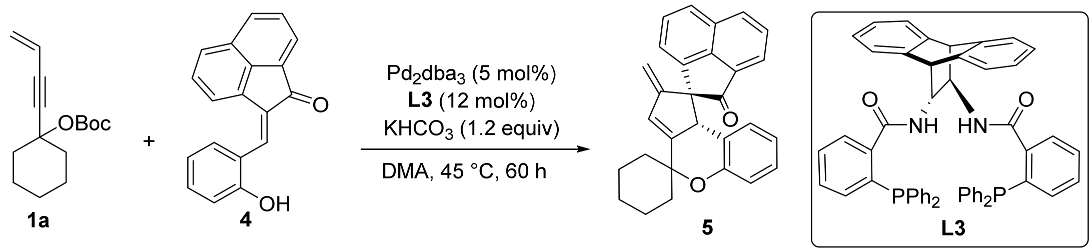

To an oven-dried 10 mL Schlenk tube equipped with a magnetic stirring bar were added 1-(but-3-en-1-yn-1-yl)cyclohexyl *tert*-butyl carbonate **1a** (37.6 mg, 0.150 mmol, 1.5 equiv), (*E*)-2-(2-hydroxy benzylidene)acenaphthylen-1(2*H*)-one **4** (27.2 mg, 0.0999 mmol, 1.0 equiv), Pd_2dba_3 (4.6 mg, 0.0050 mmol, 5 mol%), **L3** (9.8 mg, 0.012 mmol, 12 mol%) and $KHCO_3$ (12.0 mg, 0.120 mmol, 1.2 equiv). The tube was capped, evacuated and back-filled with argon for five times. Then degassed dry DMA (2.0 mL) was added via syringe. The mixture was stirred at 45 °C for 60 h, and monitored by TLC (EtOAc/petroleum ether = 1/5). After completion, the solvent was evaporated in vacuo. The residue was purified by flash chromatography on silica gel (EtOAc/petroleum ether = 1/5) to give the product **5**: 30.4 mg (0.0751 mmol), as a white solid, 75% yield; mp 163–165 °C; >19:1 dr, determind by ^{1}H NMR analysis of crude product; $[\alpha_D^{25}]$ = +154.2 (*c* = 9.43 in $CHCl_3$); 83% ee, determined by HPLC analysis (Daicel Chiralpak IE, *n*-hexane/*i*-PrOH = 95/5, flow rate =1.0 mL/min, l = 254 nm) t_R = 8.08 min (minor), t_R = 8.62 min (major); ^{1}H NMR (400 MHz, $CDCl_3$): δ (ppm) δ 8.19–8.05 (m, 2H), 7.77 (t, *J* = 7.2 Hz, 1H), 7.64 (d, *J* = 8.0 Hz, 1H), 7.25–7.21 (m, 1H), 6.90–6.75 (m, 3H), 6.25 (d, *J* = 2.4 Hz, 1H), 6.21–6.12 (m, 1H), 5.79 (d, *J* = 7.6 Hz, 1H), 4.90 (s, 2H), 4.33 (s, 1H), 2.24–2.12 (m, 1H), 2.11–1.96 (m, 3H), 1.86–1.62 (m, 3H), 1.60–1.53 (m, 1H), 1.53–1.37 (m, 2H); ^{13}C NMR (100 MHz, $CDCl_3$): δ (ppm) 206.2, 157.9, 152.8, 152.6, 141.9, 141.6, 133.6, 132.0, 130.8, 128.8, 128.0, 127.8, 127.5, 124.4, 123.7, 122.2, 122.1, 120.1, 119.7, 117.5, 105.6, 76.2, 68.1, 49.7, 34.5, 32.6, 25.7, 21.8, 21.4; HRMS (ESI-TOF) *m/z*: [M + H]$^+$ Calcd for $C_{29}H_{25}O_2^+$ 405.1849; Found 405.1851.

4.2 Procedure for asmmytric synthesis of 7 and 9

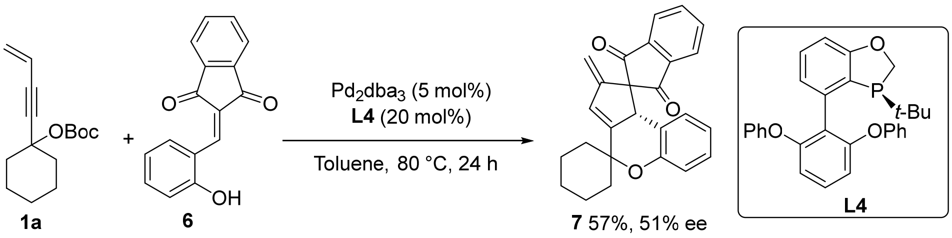

To an oven-dried 10 mL Schlenk tube equipped with a magnetic stirring bar were added 1-(but-3-en-1-yn-1-yl)cyclohexyl *tert*-butyl carbonate **1a** (37.6 mg, 0.150 mmol, 1.5 equiv), 2-(2-hydroxy benzylidene)-1*H*-indene-1,3(2*H*)-dione **6** (25.0 mg, 0.0999 mmol, 1.0 equiv), Pd_2dba_3 (4.6 mg, 0.0050 mmol, 5 mol%) and **L4** (9.1 mg, 0.020 mmol, 20 mol%). The tube was capped, evacuated and back-filled with argon for five times. Then degassed dry Tol (1.0 mL) was added via syringe. The mixture was stirred at 80 °C for 24 h, and monitored by TLC (EtOAc/petroleum ether = 1/5). After completion, the solvent was evaporated in vacuo. The residue was purified by flash chromatography on silica gel (EtOAc/petroleum ether = 1/5) to give the product **7**: 21.9 mg (0.0573 mmol), as a white solid, 57% yield; mp 234–236 °C; $[\alpha_D^{25}]$ = +175.3 (*c* = 4.23 in $CHCl_3$); 51% ee, determined by HPLC analysis (Daicel Chiralpak IB, *n*-hexane/*i*-PrOH = 90/10, flow rate =1.0 mL/min, l = 254 nm) t_R = 7.75 min (major), t_R = 8.49 min (minor); ^1H NMR (400 MHz, $CDCl_3$): δ (ppm) 8.20–8.13 (m, 1H), 7.94–7.84 (m, 3H), 7.07–6.98 (m, 1H), 6.94–6.85 (m, 1H), 6.58–6.50 (m, 1H), 6.21 (d, *J* = 7.6 Hz, 1H), 6.10 (d, *J* = 2.8 Hz, 1H), 4.97 (s, 1H), 4.86 (s, 1H), 4.28 (s, 1H), 2.17–2.10 (m, 1H), 2.03–1.88 (m, 3H), 1.78–1.68 (m, 2H), 1.60–1.23 (m, 4H); ^{13}C NMR (100 MHz, $CDCl_3$): δ (ppm) 201.3, 195.1, 154.4, 153.1, 153.0, 143.6, 142.7, 136.1, 135.6, 128.1, 126.6, 124.7, 123.71, 123.68, 121.9, 120.5, 118.1, 106.2, 76.0, 68.6, 47.2, 34.2, 32.8, 25.6, 21.6, 21.4; HRMS (ESI-TOF) *m/z*: $[M + Na]^+$ Calcd for $C_{26}H_{22}NO_3Na^+$ 405.1461; Found 405.1459.

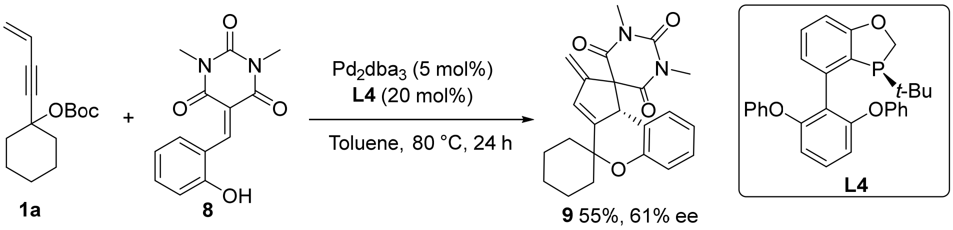

To an oven-dried 10 mL Schlenk tube equipped with a magnetic stirring bar were added 1-(but-3-en-1-yn-1-yl)cyclohexyl *tert*-butyl carbonate **1a** (37.6 mg, 0.150 mmol, 1.5 equiv), 5-(2-hydroxy benzylidene)-1,3-dimethylpyrimidine-2,4,6(1*H*,3*H*,5*H*)-trione **8** (26.0 mg, 0.0999 mmol, 1.0 equiv),

Pd_2dba_3 (4.6 mg, 0.0050 mmol, 5 mol%) and **L4** (9.1 mg, 0.020 mmol, 20 mol%). The tube was capped, evacuated and back-filled with argon for five times. Then degassed dry toluene (1.0 mL) was added via syringe. The mixture was stirred at 45 °C for 24 h, and monitored by TLC (EtOAc/petroleum ether = 1/5). After completion, the solvent was evaporated in vacuo. The residue was purified by flash chromatography on silica gel (EtOAc/petroleum ether = 1/5) to give the product **9**: 21.6 mg (0.0550 mmol), as a white solid, 55% yield; mp 148–150 °C; $[\alpha_D^{25}]$ = +98.3 (c = 5.32 in $CHCl_3$); 61% ee, determined by HPLC analysis (Daicel Chiralpak AD, n-hexane/i-PrOH = 80/20, flow rate =1.0 mL/min, l = 254 nm) t_R = 5.22 min (minor), t_R = 6.21 min (major); ^1H NMR (400 MHz, $CDCl_3$): δ (ppm) 7.14–7.07 (m, 1H), 6.93 (d, J = 8.0 Hz, 1H), 6.82–6.75 (m, 1H), 6.53 (d, J = 7.6 Hz, 1H), 6.01 (d, J = 2.8 Hz, 1H), 5.13 (s, 1H), 5.07 (s, 1H), 4.76 (s, 1H), 3.52 (s, 3H), 3.08 (s, 3H), 2.15–2.06 (m, 1H), 2.02–1.85 (m, 3H), 1.77–1.60 (m, 3H), 1.52–1.33 (m, 3H); ^{13}C NMR (100 MHz, $CDCl_3$): δ (ppm) 170.0, 165.7, 154.5, 153.7, 153.2, 151.2, 128.2, 125.3, 122.3, 121.6, 120.9, 118.2, 105.7, 76.1, 65.6, 50.4, 34.2, 32.7, 29.4, 28.8, 25.5, 21.5, 21.2; HRMS (ESI-TOF) m/z: $[M + Na]^+$ Calcd for $C_{23}H_{24}N_2O_4Na^+$ 415.1628; Found 415.1628.

5 General procedure for reactions of racemic 1-alkyl-substituted 4-aken-2-ynyl carbonates 10

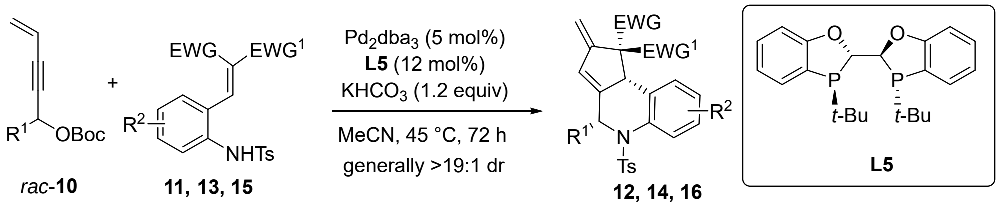

General procedure: To an oven-dried 10 mL Schlenk tube equipped with a magnetic stirring bar were added 1-alkyl-substituted 4-aken-2-ynyl carbonate **10** (0.2 mmol, 2.0 equiv), activated alkene **11**, **13** or **15** (0.1 mmol, 1.0 equiv), Pd_2dba_3 (4.6 mg, 0.0050 mmol, 5 mol%), **L5** (9.8 mg, 0.012 mmol, 12 mol%) and $KHCO_3$ (12.0 mg, 0.120 mmol, 1.2 equiv). The tube was capped, evacuated and back-filled with argon for five times. Then degassed dry MeCN (1.0 mL) was added via syringe. The mixture was stirred at 45 °C for 72 h, and monitored by TLC (EtOAc/petroleum ether). After completion, the solvent was evaporated in vacuo. The residue was purified by flash chromatography on silica gel (EtOAc/petroleum ether) to give the product.

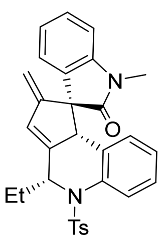
12a

Synthesis of 12a: To an oven-dried 10 mL Schlenk tube equipped with a magnetic stirring bar were added *tert*-butyl hept-6-en-4-yn-3-yl carbonate **10a** (42.1 mg, 0.200 mmol, 2.0 equiv), (*E*)-4-methyl-*N*-(2-((1-methyl-2-oxoindolin-3-ylidene)methyl)-phenyl) benzene sulfonamide **11a** (40.4 mg, 0.0999 mmol, 1.0 equiv), Pd_2dba_3 (4.6 mg, 0.0050 mmol, 5 mol%), **L5** (4.6 mg, 0.012 mmol, 12 mol%) and $KHCO_3$ (12.0 mg, 0.120 mmol, 1.2 equiv). The tube was capped, evacuated and back-filled with argon for five times. Then degassed dry MeCN (1.0 mL) was added via syringe. The mixture was stirred at 45 °C for 72 h, and monitored by TLC (EtOAc/petroleum ether = 1/5). After completion, the solvent was evaporated in vacuo. The residue was purified by flash chromatography on silica gel (EtOAc/petroleum ether = 1/5) to give the product **12a**: 34.8 mg (0.0701 mmol), as a white solid, 70% yield; mp 134–136 °C; >19:1 dr, determind by ^{1}H NMR analysis of crude product; $[\alpha_D^{25}]$ = −135.7 (*c* = 12.3 in $CHCl_3$); 93% ee, determined by HPLC analysis (Daicel Chiralpak AD, *n*-hexane/*i*-PrOH = 80/20, flow rate =1.0 mL/min, l = 254 nm) t_R = 4.92 min (minor), t_R = 7.77 min (major); ^{1}H NMR (400 MHz, $CDCl_3$): δ (ppm) 7.63 (d, *J* = 8.0 Hz, 1H), 7.34–7.27 (m, 2H), 7.21–7.14 (m, 3H), 7.14–7.08 (m, 1H), 6.89 (d, *J* = 7.6 Hz, 1H), 6.79–6.68 (m, 2H), 6.58 (d, *J* = 6.8 Hz, 1H), 6.05 (s, 1H), 5.74 (d, *J* = 7.6 Hz, 1H), 5.33–5.20 (m, 1H), 4.71 (s, 1H), 4.38 (s, 1H), 3.31 (s, 3H), 3.02 (s, 1H), 2.45 (s, 3H), 1.73–1.52 (m, 2H), 1.17 (t, *J* = 7.6 Hz, 3H); ^{13}C NMR (150 MHz, $CDCl_3$): δ (ppm) 176.7, 155.0, 153.3, 144.8, 142.5, 135.8, 134.3, 132.3, 131.6, 129.8, 129.5, 128.1, 126.7, 126.6, 126.5, 126.2, 124.4, 122.6, 122.4, 108.5, 103.3, 61.6, 58.1, 50.1, 31.5, 26.7, 21.7, 10.8; HRMS (ESI-TOF) *m/z*: $[M + Na]^+$ Calcd for $C_{30}H_{28}N_2O_3SNa^+$ 519.1713; Found 519.1720.

Recovered *S*-**10a**: 43% yield; $[\alpha_D^{25}]$ = −75.5 (*c* = 2.2 in $CHCl_3$); 68% ee, determined by HPLC analysis (Daicel Chiralpak AD, *n*-hexane/*i*-PrOH = 99/1, flow rate =0.4 mL/min, l = 254 nm) t_R = 11.24 min (minor), t_R = 13.06 min (major).

Asymmetric synthesis of 12a on a 1.0 mmol scale: To an oven-dried 50 mL Schlenk tube equipped with a magnetic stirring bar were added *tert*-butyl hept-6-en-4-yn-3-yl carbonate **10a** (420.6 mg, 2.000 mmol), (*E*)-4-methyl-*N*-(2-((1-methyl-2-oxoindolin-3-ylidene)methyl)phenyl) benzenesulfon amide **11a** (404.5 mg, 1.000 mmol), $Pd_2(dba)_3$ (45.8 mg, 0.0500 mmol, 5 mol%), **L5** (46.4 mg, 0.120 mmol, 12 mol%) and $KHCO_3$ (120.2 mg, 1.201 mmol). The tube was capped, evacuated and back-filled with argon for five times. Then degassed dry MeCN (10 mL) was added via syringe. The

mixture was stirred at 45 °C for 72 h, and monitored by TLC (EtOAc/petroleum ether = 1/5). After completion, the solvent was evaporated in vacuo. The residue was purified by flash chromatography on silica gel (EtOAc/petroleum ether = 1/5) to give the product **12a**: 322.9 mg (0.6502 mmol), as a white solid, 65% yield, >19:1 *dr*, 93% ee.

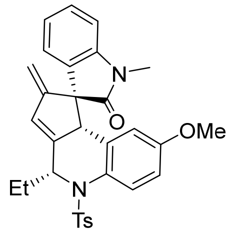

12b

Synthesis of 12b: To an oven-dried 10 mL Schlenk tube equipped with a magnetic stirring bar were added *tert*-butylhept-6-en-4-yn-3-yl carbonate **10a** (42.1 mg, 0.200 mmol, 2.0 equiv), (*E*)-*N*-(4-methoxy-2-((1-methyl-2-oxoindolin-3-ylidene) methyl)phenyl)-4-methylbenzenesulfonamide **11b** (43.4 mg, 0.0999 mmol, 1.0 equiv), Pd_2dba_3 (4.6 mg, 0.0050 mmol, 5 mol%), **L5** (4.6 mg, 0.012 mmol, 12 mol%) and $KHCO_3$ (12.0 mg, 0.120 mmol, 1.2 equiv). The tube was capped, evacuated and back-filled with argon for five times. Then degassed dry MeCN (1.0 mL) was added via syringe. The mixture was stirred at 45 °C for 72 h, and monitored by TLC (EtOAc/petroleum ether = 1/5). After completion, the solvent was evaporated in vacuo. The residue was purified by flash chromatography on silica gel (EtOAc/petroleum ether = 1/5) to give the product **12b**: 34.9 mg (0.0663 mmol), as a white solid, 66% yield; mp 154–156 °C; >19:1 dr, determind by ^{1}H NMR analysis of crude product; $[\alpha_D^{25}]$ = −114.7 (*c* = 0.30 in $CHCl_3$); 93% ee, determined by HPLC analysis (Daicel Chiralpak AD, *n*-hexane/*i*-PrOH = 80/20, flow rate =1.0 mL/min, l = 254 nm) t_R = 5.17 min (minor), t_R = 8.29 min (major); ^{1}H NMR (400 MHz, $CDCl_3$): δ (ppm) 7.51 (d, *J* = 8.8 Hz, 1H), 7.33–7.29 (m, 2H), 7.23–7.16 (m, 3H), 6.88 (d, *J* = 7.6 Hz, 1H), 6.77 (t, *J* = 7.2 Hz, 1H), 6.70–6.63 (m, 1H), 6.61 (d, *J* = 7.2 Hz, 1H), 6.04 (s, 1H), 5.29–5.16 (m, 2H), 4.72 (s, 1H), 4.37 (s, 1H), 3.31 (s, 3H), 3.28 (s, 3H), 2.93 (s, 1H), 2.47 (s, 3H), 1.70–1.62 (m, 1H), 1.59–1.49 (m, 1H), 1.15 (t, *J* = 7.2 Hz, 3H); ^{13}C NMR (100 MHz, $CDCl_3$): δ (ppm) 176.7, 157.7, 154.9, 153.2, 144.8, 142.5, 135.8, 133.4, 131.8, 130.6, 129.8, 128.2, 126.9, 126.6, 124.5, 122.9, 122.8, 112.9, 111.2, 108.3, 103.4, 61.5, 58.0, 55.0, 50.5, 31.4, 26.7, 21.7, 10.8; HRMS (ESI-TOF) *m/z*: $[M + Na]^+$ Calcd for $C_{31}H_{30}N_2O_4SNa^+$ 549.1818; Found 549.1816.

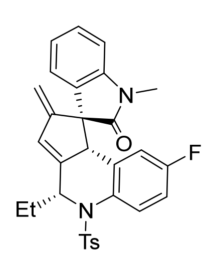

12c

Synthesis of 12c: To an oven-dried 10 mL Schlenk tube equipped with a magnetic stirring bar were added *tert*-butyl hept-6-en-4-yn-3-yl carbonate **10a** (42.1 mg, 0.200 mmol, 2.0 equiv), (*E*)-*N*-(4-fluoro-2-((1-methyl-2-oxoindolin-3-ylidene) methyl)phenyl)-4-methylbenzenesulfonamide **11c** (42.2 mg, 0.0999 mmol, 1.0 equiv), Pd_2dba_3 (4.6 mg, 0.0050 mmol, 5 mol%), **L5** (4.6 mg, 0.012 mmol, 12

mol%) and $KHCO_3$ (12.0 mg, 0.120 mmol, 1.2 equiv). The tube was capped, evacuated and back-filled with argon for five times. Then degassed dry MeCN (1.0 mL) was added via syringe. The mixture was stirred at 45 °C for 72 h, and monitored by TLC (EtOAc/petroleum ether = 1/5). After completion, the solvent was evaporated in vacuo. The residue was purified by flash chromatography on silica gel (EtOAc/petroleum ether = 1/5) to give the product **12c**: 36.6 mg (0.0711 mmol), as a white solid, 71% yield; mp 132–134 °C; >19:1 dr, determind by ^{1}H NMR analysis of crude product; $[\alpha_D^{25}]$ = −85.8 (*c* = 0.86 in $CHCl_3$); 90% ee, determined by HPLC analysis (Daicel Chiralpak AD, *n*-hexane/*i*-PrOH = 80/20, flow rate =1.0 mL/min, l = 254 nm) t_R = 5.20 min (minor), t_R = 8.58 min (major); ^{1}H NMR (400 MHz, $CDCl_3$): δ (ppm) 7.60 (dd, *J* = 8.8, 5.6 Hz, 1H), 7.34–7.28 (m, 2H), 7.25–7.15 (m, 3H), 6.90 (d, *J* = 8.0 Hz, 1H), 6.86–6.80 (m, 1H), 6.80–6.74 (m, 1H), 6.58 (d, *J* = 7.6 Hz, 1H), 6.06 (s, 1H), 5.47–5.39 (m, 1H), 5.28–5.22 (m, 1H), 4.74 (s, 1H), 4.38 (s, 1H), 3.32 (s, 3H), 2.92 (s, 1H), 2.48 (s, 3H), 1.71–1.62 (m, 1H), 1.58–1.50 (m, 1H), 1.16 (t, *J* = 7.2 Hz, 3H); ^{13}C NMR (100 MHz, $CDCl_3$): δ (ppm) 176.4, 160.7 (d, $^1J_{FC}$ = 245.1 Hz), 154.7, 152.4, 145.1, 142.5, 135.6, 134.7 (d, $^3J_{FC}$ = 8.3 Hz), 131.2, 131.14, 131.10, 130.2 (d, *J* = 2.9 Hz), 129.9, 128.5, 126.6, 124.8, 122.8, 122.5, 113.7 (d, $^2J_{FC}$ = 22.3 Hz), 113.3 (d, $^2J_{FC}$ = 24.3 Hz), 108.7, 103.8, 61.4, 58.0, 50.2, 31.4, 26.7, 21.7, 10.7; ^{19}F NMR (375 MHz, $CDCl_3$): δ (ppm) –113.6; HRMS (ESI-TOF) *m/z*: $[M + Na]^+$ Calcd for $C_{30}H_{27}FN_2O_3SNa^+$ 537.1619; Found 537.1617.

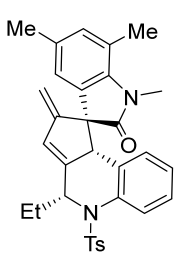

12d

Synthesis of 12d: To an oven-dried 10 mL Schlenk tube equipped with a magnetic stirring bar were added *tert*-butyl hept-6-en-4-yn-3-yl carbonate **10a** (42.1 mg, 0.200 mmol, 2.0 equiv), (*E*)-4-methyl-*N*-(2-((1,5,7-trimethyl-2-oxoindolin-3-ylidene) methyl)phenyl)benzenesulfonamide **11d** (43.2 mg, 0.0999 mmol, 1.0 equiv), Pd_2dba_3 (4.6 mg, 0.0050 mmol, 5 mol%), **L5** (4.6 mg, 0.012 mmol, 12 mol%) and $KHCO_3$ (12.0 mg, 0.120 mmol, 1.2 equiv). The tube was capped, evacuated and back-filled with argon for five times. Then degassed dry MeCN (1.0 mL) was added via syringe. The mixture was stirred at 45 °C for 72 h, and monitored by TLC (EtOAc/petroleum ether = 1/5). After completion, the solvent was evaporated in vacuo. The residue was purified by flash chromatography on silica gel (EtOAc/petroleum ether = 1/5) to give the product **12d**: 36.8 mg (0.0701 mmol), as a white solid, 70% yield; mp 160–161 °C; >19:1 dr, determind by ^{1}H NMR analysis of crude product; $[\alpha_D^{25}]$ = –65.8 (*c* = 8.23 in $CHCl_3$); 94% ee, determined by HPLC analysis (Daicel Chiralpak IA, *n*-hexane/*i*-PrOH =

90/10, flow rate =1.0 mL/min, l = 254 nm) t_R = 8.90 min (minor), t_R = 12.76 min (major); ^{1}H NMR (400 MHz, $CDCl_3$): δ (ppm) 7.64 (d, *J* = 8.0 Hz, 1H), 7.33–7.27 (m, 2H), 7.20–7.08 (m, 3H), 6.80 (t, *J* = 7.6 Hz, 1H), 6.71 (s, 1H), 6.22 (s, 1H), 6.02 (s, 1H), 5.80 (d, *J* = 8.0 Hz, 1H), 5.31–5.20 (m, 1H), 4.71 (s, 1H), 4.37 (s, 1H), 3.55 (s, 3H), 3.02 (s, 1H), 2.56 (s, 3H), 2.45 (s, 3H), 1.98 (s, 3H), 1.75–1.64 (m, 1H), 1.61–1.52 (m, 1H), 1.17 (t, *J* = 7.2 Hz, 3H); ^{13}C NMR (100 MHz, $CDCl_3$): δ (ppm) 177.6, 155.7, 153.4, 144.8, 137.8, 135.9, 134.3, 132.4, 132.39, 132.35, 131.8, 129.8, 129.4, 126.6, 126.5, 126.4, 124.4, 121.2, 119.6, 103.2, 61.1, 58.1, 50.5, 31.4, 30.0, 21.7, 20.7, 19.1, 10.8; HRMS (ESI-TOF) *m/z*: $[M + H]^+$ Calcd for $C_{32}H_{33}N_2O_3S^+$ 525.2206; Found 525.2210.

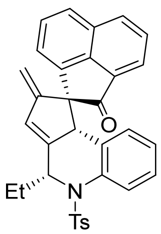

14

Synthesis of 14: To an oven-dried 10 mL Schlenk tube equipped with a magnetic stirring bar were added *tert*-butyl hept-6-en-4-yn-3-yl carbonate **10a** (42.1 mg, 0.200 mmol, 2.0 equiv), (*E*)-4-methyl-*N*-(2-((2-oxoacenaphthylen-1(2*H*)-ylidene)methyl) phenyl)benzenesulfonamide **13** (42.5 mg, 0.0999 mmol, 1.0 equiv), Pd_2dba_3 (4.6 mg, 0.0050 mmol, 5 mol%), **L5** (4.6 mg, 0.012 mmol, 12 mol%) and $KHCO_3$ (12.0 mg, 0.120 mmol, 1.2 equiv). The tube was capped, evacuated and back-filled with argon for five times. Then degassed dry MeCN (1.0 mL) was added via syringe. The mixture was stirred at 45 °C for 72 h, and monitored by TLC (EtOAc/petroleum ether = 1/6). After completion, the solvent was evaporated in vacuo. The residue was purified by flash chromatography on silica gel (EtOAc/petroleum ether = 1/6) to give the product **14**: 31.6 mg (0.0610 mmol), as a white solid, 61% yield; mp 126–128 °C; >19:1 dr, determind by ^{1}H NMR analysis of crude product; $[\alpha_D^{25}]$ = −75.3 (*c* = 0.56 in $CHCl_3$); 90% ee, determined by HPLC analysis (Daicel Chiralpak AD, *n*-hexane/*i*-PrOH = 80/20, flow rate =1.0 mL/min, l = 254 nm) t_R = 4.77 min (minor), t_R = 6.70 min (major); ^{1}H NMR (400 MHz, $CDCl_3$): δ (ppm) 8.14 (d, *J* = 8.0 Hz, 1H), 8.04 (d, *J* = 6.8 Hz, 1H), 7.81–7.77 (m, 1H), 7.75 (d, *J* = 8.4 Hz, 1H), 7.65–7.61 (m, 1H), 7.35–7.29 (m, 3H), 7.23–7.19 (m, 2H), 7.07 (t, *J* = 6.8 Hz, 1H), 6.81 (d, *J* = 6.8 Hz, 1H), 6.53 (t, *J* = 7.6 Hz, 1H), 6.04 (s, 1H), 5.39 – 5.30 (m, 2H), 4.65 (s, 1H), 4.12 (s, 1H), 3.09 (s, 1H), 2.56 (s, 3H), 1.79–1.63 (m, 2H), 1.21 (t, *J* = 7.2 Hz, 3H); ^{13}C NMR (100 MHz, $CDCl_3$): δ (ppm) 203.9, 157.3, 153.5, 144.9, 141.2, 141.1, 135.8, 134.3, 133.1, 132.9, 132.0, 131.2, 129.8, 129.7, 128.7, 128.3, 126.61, 126.59, 126.4, 126.3, 124.3, 124.1, 122.1, 119.3, 103.8, 66.8, 58.2, 49.7, 31.7, 21.8, 10.8; HRMS (ESI-TOF) *m/z*: $[M + H]^+$ Calcd for $C_{33}H_{28}NO_3S^+$ 518.1784; Found 518.1793.

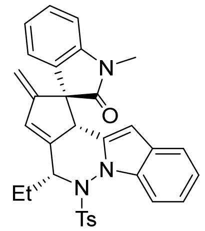

16a

Synthesis of 16a: To an oven-dried 10 mL Schlenk tube equipped with a magnetic stirring bar were added *tert*-butyl hept-6-en-4-yn-3-yl carbonate **10a** (42.1 mg, 0.200 mmol, 2.0 equiv), (*E*)-4-methyl-*N*-(2-((1-methyl-2-oxoindolin-3-ylidene)methyl)-1*H*-indol-1-yl)benzenesulfonamide **15a** (44.3 mg, 0.0999 mmol, 1.0 equiv), Pd_2dba_3 (4.6 mg, 0.0050 mmol, 5 mol%), **L5** (4.6 mg, 0.012 mmol, 12 mol%) and $KHCO_3$ (12.0 mg, 0.120 mmol, 1.2 equiv). The tube was capped, evacuated and back-filled with argon for five times. Then degassed dry MeCN (1.0 mL) was added via syringe. The mixture was stirred at 45 °C for 72 h, and monitored by TLC (EtOAc/petroleum ether = 1/5). After completion, the solvent was evaporated in vacuo. The residue was purified by flash chromatography on silica gel (EtOAc/petroleum ether = 1/5) to give the product **16a**: 39.2 mg (0.0732 mmol), as a white solid, 73% yield; mp 114–116 °C; >19:1 dr, determind by ^{1}H NMR analysis of crude product; $[\alpha_D^{25}]$ = –37.6 (*c* = 0.73 in $CHCl_3$); 85% ee, determined by HPLC analysis (Daicel Chiralpak IA, *n*-hexane/*i*-PrOH = 95/5, flow rate =1.0 mL/min, l = 254 nm) t_R = 10.67 min (minor), t_R = 13.42 min (major); ^{1}H NMR (400 MHz, $CDCl_3$): δ (ppm) 7.55 (d, *J* = 8.4 Hz, 1H), 7.50–7.42 (m, 2H), 7.27–7.25 (m, 2H), 7.25–7.22 (m, 1H), 7.22–7.18 (m, 1H), 7.18–7.12 (m, 1H), 7.06–6.98 (m, 1H), 6.91 (d, *J* = 7.6 Hz, 1H), 6.76–6.72 (m, 1H), 6.57 (d, *J* = 7.6 Hz, 1H), 6.15–6.08 (m, 1H), 5.37–5.28 (m, 1H), 4.84 (s, 1H), 4.72 (s, 1H), 4.43 (s, 1H), 3.29 (s, 3H), 3.10 (s, 1H), 2.54 (s, 3H), 1.58–1.49 (m, 1H), 1.39–1.26 (m, 1H), 1.16 (t, *J* = 7.2 Hz, 3H); ^{13}C NMR (100 MHz, $CDCl_3$): δ (ppm) 176.4, 154.5, 148.1, 146.9, 142.8, 136.8, 132.8, 132.0, 131.2, 130.3, 128.5, 128.3, 126.4, 125.3, 123.8, 122.4, 122.3, 120.8, 120.1, 110.8, 108.2, 105.6, 98.1, 61.0, 60.8, 48.7, 30.0, 26.7, 21.9, 11.1; HRMS (ESI-TOF) *m/z*: $[M + Na]^+$ Calcd for $C_{32}H_{29}N_3O_3SNa^+$ 558.1822; Found 558.1819.

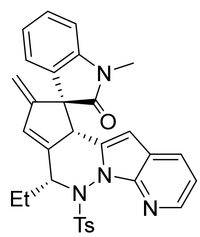

16b

Synthesis of 16b: To an oven-dried 10 mL Schlenk tube equipped with a magnetic stirring bar were added *tert*-butyl hept-6-en-4-yn-3-yl carbonate **10a** (42.1 mg, 0.200 mmol, 2.0 equiv), (*E*)-4-methyl-*N*-(2-((1-methyl-2-oxoindolin-3-ylidene) methyl)-1*H*-pyrrolo[2,3-*b*]pyridin-1-yl)benzenesulfonamide **15b** (44.4 mg, 0.0999 mmol, 1.0 equiv), Pd_2dba_3 (4.6 mg, 0.0050 mmol, 5 mol%), **L5** (4.6 mg, 0.012 mmol, 12 mol%) and $KHCO_3$ (12.0 mg, 0.120 mmol, 1.2 equiv). The tube was capped, evacuated and back-filled with argon for five times. Then degassed dry MeCN (1.0 mL) was added via syringe. The mixture was stirred at 45 °C for 72 h, and monitored by TLC (EtOAc/petroleum ether = 1/3).

After completion, the solvent was evaporated in vacuo. The residue was purified by flash chromatography on silica gel (EtOAc/petroleum ether = 1/3) to give the product **16b**: 23.2 mg (0.0432 mmol), as a white solid, 43% yield; mp 176–178 °C; >19:1 dr, determind by ^{1}H NMR analysis of crude product; $[\alpha_D^{25}]$ = −74.8 (*c* = 0.57 in $CHCl_3$); 96% ee, determined by HPLC analysis (Daicel Chiralpak AD, *n*-hexane/*i*-PrOH = 80/20, flow rate =1.0 mL/min, l = 254 nm) t_R = 8.32 min (minor), t_R = 10.30 min (major); ^{1}H NMR (400 MHz, $CDCl_3$): δ (ppm) 8.22 (d, *J* = 3.6 Hz, 1H), 7.82–7.70 (m, 2H), 7.68–7.58 (m, 1H), 7.41–7.37 (m, 1H), 7.26 (s, 2H), 7.14–7.10 (m, 1H), 7.05–6.91 (m, 3H), 6.25 (s, 1H), 5.52 (s, 1H), 5.29–5.18 (m, 1H), 4.87 (s, 1H), 4.25 (s, 1H), 3.91 (s, 1H), 3.23 (s, 3H), 2.50 (s, 3H), 1.64–1.55 (m, 2H), 1.03 (t, *J* = 7.2 Hz, 3H); ^{13}C NMR (100 MHz, $CDCl_3$): δ (ppm) 174.9, 152.7, 147.4, 147.3, 145.2, 144.2, 143.6, 134.5, 134.1, 129.8, 129.3, 129.2, 128.5, 126.5, 124.0, 122.9, 118.5, 117.1, 108.2, 105.7, 93.9, 61.7, 60.3, 49.6, 29.5, 26.4, 21.8, 10.5; HRMS (ESI-TOF) *m/z*: $[M + Na]^+$ Calcd for $C_{31}H_{28}N_4O_3SNa^+$ 559.1774; Found 559.1780.

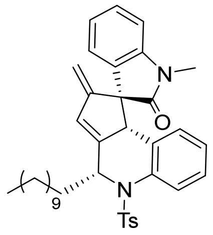

12e

Synthesis of 12e: To an oven-dried 10 mL Schlenk tube equipped with a magnetic stirring bar were added *tert*-butyl hexadec-1-en-3-yn-5-yl carbonate **10b** (67.3 mg, 0.200 mmol, 2.0 equiv), (*E*)-4-methyl-*N*-(2-((1-methyl-2-oxoindolin-3-ylidene)methyl)phenyl)benzenesulfonamide **11a** (40.4 mg, 0.0999 mmol, 1.0 equiv), Pd_2dba_3 (4.6 mg, 0.0050 mmol, 5 mol%), **L5** (4.6 mg, 0.012 mmol, 12 mol %) and $KHCO_3$ (12.0 mg, 0.120 mmol, 1.2 equiv). The tube was capped, evacuated and back-filled with argon for five times. Then degassed dry MeCN (1.0 mL) was added via syringe. The mixture was stirred at 45 °C for 72 h, and monitored by TLC (EtOAc/petroleum ether = 1/5). After completion, the solvent was evaporated in vacuo. The residue was purified by flash chromatography on silica gel (EtOAc/petroleum ether = 1/5) to give the product **12e**: 38.1 mg (0.0612 mmol), as a white solid, 61% yield; mp 112–114 °C; >19:1 dr, determind by ^{1}H NMR analysis of crude product; $[\alpha_D^{25}]$ = −32.0 (*c* = 0.15 in $CHCl_3$); 95% ee, determined by HPLC analysis (Daicel Chiralpak AD, *n*-hexane/*i*-PrOH = 80/20, flow rate =1.0 mL/min, l = 254 nm) t_R = 3.85 min (minor), t_R = 7.33 min (major); ^{1}H NMR (400 MHz, $CDCl_3$): δ (ppm) 7.65–7.59 (m, 1H), 7.33–7.27 (m, 2H), 7.22–7.10 (m, 4H), 6.88 (d, *J* = 8.0 Hz, 1H), 6.79–6.72 (m, 2H), 6.58 (d, *J* = 8.0 Hz, 1H), 6.06–6.00 (m, 1H), 5.73 (d, *J* = 7.6 Hz, 1H), 5.34 (t, *J* = 7.2 Hz, 1H), 4.71 (s, 1H), 4.37 (s, 1H), 3.32 (s, 3H), 2.99 (s, 1H), 2.46 (s, 3H), 1.64–1.59 (m, 2H), 1.36–1.17 (m, 18H), 0.89 (t, *J* = 7.2 Hz, 3H); ^{13}C NMR (100 MHz, $CDCl_3$):

δ (ppm) 176.7, 155.1, 153.6, 144.8, 142.5, 135.8, 134.3, 132.4, 131.7, 129.8, 129.6, 128.1, 126.7, 126.58, 126.56, 126.1, 124.3, 122.7, 122.5, 108.5, 103.2, 61.6, 56.7, 50.1, 38.4, 32.0, 29.68, 29.66, 29.63, 29.57, 29.4, 29.1, 26.7, 26.0, 22.7, 21.7, 14.2; HRMS (ESI-TOF) *m/z*: $[M + Na]^+$ Calcd for $C_{39}H_{46}N_2O_3SNa^+$ 645.3121; Found 645.3130.

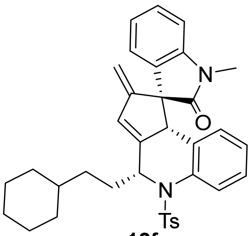
12f

Synthesis of 12f: To an oven-dried 10 mL Schlenk tube equipped with a magnetic stirring bar were added *tert*-butyl (1-cyclohexylhept-6-en-4-yn-3-yl) carbonate **10c** (58.5 mg, 0.200 mmol, 2.0 equiv), (*E*)-4-methyl-*N*-(2-((1-methyl -2-oxoindolin-3-ylidene)methyl)phenyl)benzenesulfonamide **11a** (40.4 mg, 0.0999 mmol, 1.0 equiv), Pd_2dba_3 (4.6 mg, 0.0050 mmol, 5 mol%), **L5** (4.6 mg, 0.012 mmol, 12 mol%) and $KHCO_3$ (12.0 mg, 0.120 mmol, 1.2 equiv). The tube was capped, evacuated and back-filled with argon for five times. Then degassed dry MeCN (1.0 mL) was added via syringe. The mixture was stirred at 45 °C for 72 h, and monitored by TLC (EtOAc/petroleum ether = 1/6). After completion, the solvent was evaporated in vacuo. The residue was purified by flash chromatography on silica gel (EtOAc/petroleum ether = 1/6) to give the product **12f**: 40.7 mg (0.0703 mmol), as a white solid, 70% yield; mp 116–118 °C; >19:1 dr, determind by ^{1}H NMR analysis of crude product; $[\alpha_D^{25}]$ = +78.5 (*c* = 0.43 in $CHCl_3$); 94% ee, determined by HPLC analysis (Daicel Chiralpak IA, *n*-hexane/*i*-PrOH = 60/40, flow rate =1.0 mL/min, l = 254 nm) t_R = 4.05 min (minor), t_R = 5.87 min (major); ^{1}H NMR (400 MHz, $CDCl_3$): δ (ppm) 7.61 (d, *J* = 8.0 Hz, 1H), 7.32–7.27 (m, 2H), 7.23–7.10 (m, 4H), 6.89 (d, *J* = 7.6 Hz, 1H), 6.80– 6.72 (m, 2H), 6.58 (d, *J* = 7.2 Hz, 1H), 6.03 (s, 1H), 5.73 (d, *J* = 7.6 Hz, 1H), 5.37 – 5.24 (m, 1H), 4.71 (s, 1H), 4.37 (s, 1H), 3.32 (s, 3H), 2.97 (s, 1H), 2.46 (s, 3H), 1.80–1.64 (m, 5H), 1.61–1.44 (m, 4H), 1.32–1.11 (m, 4H), 1.00–0.83 (m, 2H); ^{13}C NMR (100 MHz, $CDCl_3$): δ (ppm) 176.7, 155.1, 153.6, 144.9, 142.5, 135.8, 134.3, 132.4, 131.7, 129.8, 129.6, 128.1, 126.8, 126.60, 126.57, 126.1, 124.3, 122.7, 122.5, 108.5, 103.3, 61.6, 57.0, 50.1, 37.2, 35.8, 33.6, 33.5, 33.3, 26.7, 26.6, 26.40, 26.37, 21.7; HRMS (ESI-TOF) *m/z*: $[M + Na]^+$ Calcd for $C_{36}H_{38}N_2O_3SNa^+$ 601.2495; Found 601.2498.

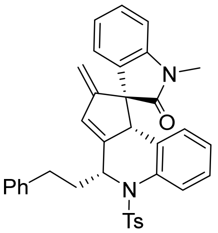
12g

Synthesis of 12g: To an oven-dried 10 mL Schlenk tube equipped with a magnetic stirring bar were added *tert*-butyl (1-phenylhept-6-en-4-yn-3-yl) carbonate **10d** (57.3 mg, 0.200 mmol, 2.0 equiv), (*E*)-4-methyl-*N*-(2-((1-methyl-2-oxoindolin-3-ylidene)methyl)phenyl)benzenesulfonamide **11a** (40.4 mg, 0.0999 mmol, 1.0

equiv), Pd_2dba_3 (4.6 mg, 0.0050 mmol, 5 mol%), **L5** (4.6 mg, 0.012 mmol, 12 mol%) and $KHCO_3$ (12.0 mg, 0.120 mmol, 1.2 equiv). The tube was capped, evacuated and back-filled with argon for five times. Then degassed dry MeCN (1.0 mL) was added via syringe. The mixture was stirred at 45 °C for 72 h, and monitored by TLC (EtOAc/petroleum ether = 1/7). After completion, the solvent was evaporated in vacuo. The residue was purified by flash chromatography on silica gel (EtOAc/petroleum ether = 1/7) to give the product **12g**: 41.3 mg (0.0721 mmol), as a white solid, 72% yield; mp 147−149 °C; >19:1 dr, determind by ^{1}H NMR analysis of crude product; $[\alpha_D^{25}]$ = +132.8 (*c* = 0.45 in $CHCl_3$); 93% ee, determined by HPLC analysis (Daicel Chiralpak IA, *n*-hexane/*i*-PrOH = 80/20, flow rate =1.0 mL/min, l = 254 nm) t_R = 6.01 min (minor), t_R = 10.28 min (major); ^{1}H NMR (400 MHz, $CDCl_3$): δ (ppm) 7.66 (d, *J* = 8.0 Hz, 1H), 7.34–7.27 (m, 4H), 7.27–7.25 (m, 1H), 7.25–7.12 (m, 6H), 6.87 (d, *J* = 8.0 Hz, 1H), 6.78 (t, *J* = 7.6 Hz, 1H), 6.71 (t, *J* = 7.6 Hz, 1H), 6.54 (d, *J* = 8.0 Hz, 1H), 6.07–6.02 (m, 1H), 5.73 (d, *J* = 8.0 Hz, 1H), 5.47–5.39 (m, 1H), 4.71 (s, 1H), 4.37 (s, 1H), 3.31 (s, 3H), 3.07–2.90 (m, 3H), 2.47 (s, 3H), 1.95–1.78 (m, 2H); ^{13}C NMR (100 MHz, $CDCl_3$): δ (ppm) 176.7, 155.0, 153.1, 145.0, 142.5, 141.4, 135.7, 134.2, 132.5, 131.6, 129.9, 129.7, 128.7, 128.5, 128.1, 126.9, 126.7, 126.6, 126.3, 126.1, 124.7, 122.7, 122.5, 108.5, 103.6, 61.6, 56.7, 50.1, 40.3, 32.6, 26.7, 21.7; HRMS (ESI-TOF) *m/z*: $[M + Na]^+$ Calcd for $C_{36}H_{32}N_2O_3SNa^+$ 595.2026; Found 595.2033.

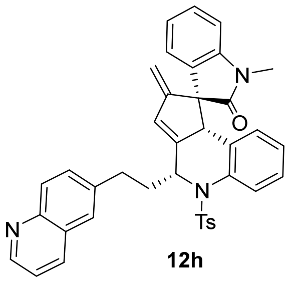

12h

Synthesis of 12h: To an oven-dried 10 mL Schlenk tube equipped with a magnetic stirring bar were added *tert*-butyl (1-(quinolin-6-yl)hept-6-en-4-yn-3-yl) carbonate **10e** (67.5 mg, 0.200 mmol, 2.0 equiv), (*E*)-4-methyl-*N*-(2-((1-methyl-2-oxoindolin-3-ylidene)methyl)phenyl)benzenesulfonamide **11a** (40.4 mg, 0.0999 mmol, 1.0 equiv), Pd_2dba_3 (4.6 mg, 0.0050 mmol, 5 mol%), **L5** (4.6 mg, 0.012 mmol, 12 mol%) and $KHCO_3$ (12.0 mg, 0.120 mmol, 1.2 equiv). The tube was capped, evacuated and back-filled with argon for five times. Then degassed dry MeCN (1.0 mL) was added via syringe. The mixture was stirred at 45 °C for 72 h, and monitored by TLC (EtOAc/petroleum ether = 1/4). After completion, the solvent was evaporated in vacuo. The residue was purified by flash chromatography on silica gel (EtOAc/petroleum ether = 1/4) to give the product **12h**: 33.8 mg (0.0542 mmol), as a white solid, 54% yield; mp 154–156 °C; >19:1 dr, determind by ^{1}H NMR analysis of crude product; $[\alpha_D^{25}]$ = +76.8 (*c* = 0.68 in $CHCl_3$); 97% ee, determined by HPLC

analysis (Daicel Chiralpak AD, *n*-hexane/*i*-PrOH = 60/40, flow rate =1.0 mL/min, l = 254 nm) t_R = 5.59 min (minor), t_R = 12.87 min (major); ^{1}H NMR (400 MHz, $CDCl_3$): δ (ppm) 8.95–8.83 (m, 1H), 8.15 (d, *J* = 8.0 Hz, 1H), 8.06 (d, *J* = 8.4 Hz, 1H), 7.74 (s, 1H), 7.69 (d, *J* = 7.6 Hz, 1H), 7.65–7.56 (m, 1H), 7.43–7.36 (m, 1H), 7.33–7.27 (m, 2H), 7.25–7.10 (m, 4H), 6.88 (d, *J* = 7.6 Hz, 1H), 6.80 (t, *J* = 7.2 Hz, 1H), 6.70 (t, *J* = 7.6 Hz, 1H), 6.54 (d, *J* = 7.2 Hz, 1H), 6.05 (s, 1H), 5.75 (d, *J* = 7.6 Hz, 1H), 5.53–5.41 (m, 1H), 4.71 (s, 1H), 4.38 (s, 1H), 3.31 (s, 3H), 3.26–3.12 (m, 2H), 2.96 (s, 1H), 2.47 (s, 3H), 2.02–1.90 (m, 2H); ^{13}C NMR (100 MHz, $CDCl_3$): δ (ppm) 176.6, 154.9, 153.0, 149.9, 147.2, 145.1, 142.5, 139.7, 135.8, 135.5, 134.1, 132.5, 131.6, 131.0, 129.9, 129.7, 129.5, 128.4, 128.2, 127.0, 126.9, 126.8, 126.6, 126.4, 124.8, 122.7, 122.5, 121.2, 108.5, 103.7, 61.6, 56.5, 50.0, 40.2, 32.6, 26.7, 21.7; HRMS (ESI-TOF) *m/z*: $[M + H]^+$ Calcd for $C_{39}H_{34}N_3O_3S^+$ 624.2315; Found 624.2323.

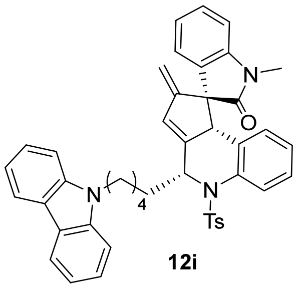

12i

Synthesis of 12i: To an oven-dried 10 mL Schlenk tube equipped with a magnetic stirring bar were added 10-(9*H*-carbazol-9-yl)dec-1-en-3-yn-5-yl *tert*-butyl carbonate **10f** (83.5 mg, 0.200 mmol, 2.0 equiv), (*E*)-4-methyl-*N*-(2-((1-methyl-2-oxoindolin-3-ylidene)methyl)phenyl)benzenesulfonamide **11a** (40.4 mg, 0.0999 mmol, 1.0 equiv), Pd_2dba_3 (4.6 mg, 0.0050 mmol, 5 mol%), **L5** (4.6 mg, 0.012 mmol, 12 mol%) and $KHCO_3$ (12.0 mg, 0.120 mmol, 1.2 equiv). The tube was capped, evacuated and back-filled with argon for five times. Then degassed dry MeCN (1.0 mL) was added via syringe. The mixture was stirred at 45 °C for 72 h, and monitored by TLC (EtOAc/petroleum ether = 1/4). After completion, the solvent was evaporated in vacuo. The residue was purified by flash chromatography on silica gel (EtOAc/petroleum ether = 1/4) to give the product **12i**: 47.3 mg (0.0672 mmol), as a white solid, 67% yield; mp 95–97 °C; >19:1 dr, determind by ^{1}H NMR analysis of crude product; $[\alpha_D^{25}]$ = −56.8 (*c* = 3.68 in $CHCl_3$); 93% ee, determined by HPLC analysis (Daicel Chiralpak IA, *n*-hexane/*i*-PrOH = 60/40, flow rate =1.0 mL/min, l = 254 nm) t_R = 5.75 min (minor), t_R = 8.94 min (major); ^{1}H NMR (400 MHz, $CDCl_3$): δ (ppm) 8.10 (d, *J* = 8.0 Hz, 2H), 7.66–7.57 (m, 1H), 7.46–7.38 (m, 4H), 7.29–7.25 (m, 2H), 7.24–7.19 (m, 2H), 7.19–7.13 (m, 3H), 7.13–7.06 (m, 1H), 6.86 (d, *J* = 7.6 Hz, 1H), 6.75 (t, *J* = 7.6 Hz, 1H), 6.67 (t, *J* = 8.4 Hz, 1H), 6.49 (d, *J* = 7.2 Hz, 1H), 5.98–5.90 (m, 1H), 5.71 (d, *J* = 7.6 Hz, 1H), 5.31 (t, *J* = 7.6 Hz, 1H), 4.68 (s, 1H), 4.40–4.29 (m, 3H), 3.30 (s, 3H), 2.96 (s, 1H), 2.45 (s, 3H), 2.01–1.89 (m, 2H), 1.76–1.64 (m, 2H), 1.57–1.33 (m, 4H); ^{13}C NMR (100 MHz, $CDCl_3$): δ (ppm) 176.7, 155.0, 153.3, 144.9, 142.5,

140.5, 135.7, 134.2, 132.4, 131.7, 129.8, 129.6, 128.1, 126.9, 126.7, 126.6, 126.3, 125.7, 124.5, 122.8, 122.7, 122.5, 120.3, 118.8, 108.7, 108.5, 103.4, 61.6, 56.6, 50.1, 42.9, 38.2, 28.9, 26.7, 26.5, 25.8, 21.7; HRMS (ESI-TOF) *m/z*: [M + Na]$^+$ Calcd for $C_{45}H_{41}N_3O_3SNa^+$ 726.2761; Found 726.2757.

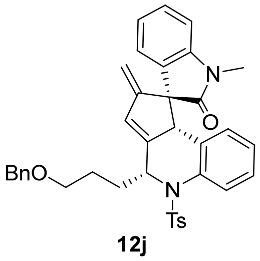

12j

Synthesis of 12j: To an oven-dried 10 mL Schlenk tube equipped with a magnetic stirring bar were added 1-(benzyloxy)oct-7-en-5-yn-4-yl *tert*-butyl carbonate **10g** (66.1 mg, 0.200 mmol, 2.0 equiv), (*E*)-4-methyl-*N*-(2-((1-methyl-2-oxoindolin-3-ylidene)methyl)phenyl)benzenesulfonamide **11a** (40.4 mg, 0.0999 mmol, 1.0 equiv), Pd_2dba_3 (4.6 mg, 0.0050 mmol, 5 mol%), **L5** (4.6 mg, 0.012 mmol, 12 mol%) and $KHCO_3$ (12.0 mg, 0.120 mmol, 1.2 equiv). The tube was capped, evacuated and back-filled with argon for five times. Then degassed dry MeCN (1.0 mL) was added via syringe. The mixture was stirred at 45 °C for 72 h, and monitored by TLC (EtOAc/petroleum ether = 1/6). After completion, the solvent was evaporated in vacuo. The residue was purified by flash chromatography on silica gel (EtOAc/petroleum ether = 1/6) to give the product **12j**: 47.6 mg (0.0772 mmol), as a white solid, 77% yield; mp 167–169 °C; >19:1 dr, determind by ^1H NMR analysis of crude product; $[\alpha_D^{25}]$ = −88.4 (*c* = 12.5 in $CHCl_3$); 95% ee, determined by HPLC analysis (Daicel Chiralpak AD, *n*-hexane/*i*-PrOH = 80/20, flow rate =1.0 mL/min, l = 254 nm) t_R = 6.18 min (minor), t_R = 9.08 min (major); ^1H NMR (400 MHz, $CDCl_3$): δ (ppm) 7.60 (d, *J* = 7.6 Hz, 1H), 7.38–7.33 (m, 3H), 7.32–7.28 (m, 2H), 7.28–7.25 (m, 2H), 7.22–7.15 (m, 3H), 7.14–7.09 (m, 1H), 6.88 (d, *J* = 8.0 Hz, 1H), 6.80–6.68 (m, 2H), 6.58 (d, *J* = 7.2 Hz, 1H), 6.04 (s, 1H), 5.73 (d, *J* = 8.0 Hz, 1H), 5.38–5.31 (m, 1H), 4.71 (s, 1H), 4.53 (s, 2H), 4.37 (s, 1H), 3.71–3.63 (m, 1H), 3.59–3.52 (m, 1H), 3.31 (s, 3H), 2.97 (s, 1H), 2.46 (s, 3H), 2.01–1.91 (m, 2H), 1.83–1.74 (m, 1H), 1.60–1.53 (m, 1H); ^{13}C NMR (100 MHz, $CDCl_3$): δ (ppm) 176.7, 155.0, 153.2, 144.9, 142.5, 138.6, 135.7, 134.2, 132.4, 131.7, 129.8, 129.7, 128.4, 128.1, 127.7, 127.6, 126.8, 126.64, 126.59, 126.2, 124.6, 122.7, 122.5, 108.5, 103.4, 72.8, 69.2, 61.6, 56.4, 50.1, 34.9, 26.7, 26.1, 21.7; HRMS (ESI-TOF) *m/z*: [M + Na]$^+$ Calcd for $C_{38}H_{36}N_2O_4SNa^+$ 639.2288; Found 639.2295.

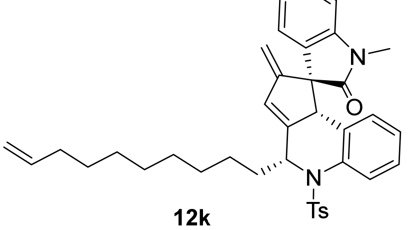

12k

Synthesis of 12k: To an oven-dried 10 mL Schlenk tube equipped with a magnetic stirring bar were added *tert*-butyl pentadeca-1,14-dien-3-yn-5-yl carbonate **10h** (64.1 mg, 0.200 mmol, 2.0 equiv), (*E*)-4-methyl-*N*-(2-((1-methyl-2-oxoindolin-3-ylidene)methyl)

phenyl)benzenesulfonamide **11a** (40.4 mg, 0.0999 mmol, 1.0 equiv), Pd_2dba_3 (4.6 mg, 0.0050 mmol, 5 mol%), **L5** (4.6 mg, 0.012 mmol, 12 mol%) and $KHCO_3$ (12.0 mg, 0.120 mmol, 1.2 equiv). The tube was capped, evacuated and back-filled with argon for five times. Then degassed dry MeCN (1.0 mL) was added via syringe. The mixture was stirred at 45 °C for 72 h, and monitored by TLC (EtOAc/petroleum ether = 1/6). After completion, the solvent was evaporated in vacuo. The residue was purified by flash chromatography on silica gel (EtOAc/petroleum ether = 1/6) to give the product **12k**: 46.3 mg (0.0763 mmol), as a white solid, 76% yield; mp 105−107 °C; >19:1 dr, determind by ^{1}H NMR analysis of crude product; $[\alpha_D^{25}]$ = −65.8 (*c* = 7.67 in $CHCl_3$); 94% ee, determined by HPLC analysis (Daicel Chiralpak AD, *n*-hexane/*i*-PrOH = 80/20, flow rate =1.0 mL/min, l = 254 nm) t_R = 4.18 min (minor), t_R = 8.45 min (major); ^{1}H NMR (400 MHz, $CDCl_3$): δ (ppm) 7.61 (d, *J* = 7.6 Hz, 1H), 7.35–7.27 (m, 2H), 7.24–7.08 (m, 4H), 6.88 (d, *J* = 7.6 Hz, 1H), 6.81–6.68 (m, 2H), 6.58 (d, *J* = 7.2 Hz, 1H), 6.03 (s, 1H), 5.88–5.77 (m, 1H), 5.73 (d, *J* = 8.0 Hz, 1H), 5.34 (t, *J* = 7.6 Hz, 1H), 5.06–4.96 (m, 1H), 4.94 (d, *J* = 10.8 Hz, 1H), 4.71 (s, 1H), 4.37 (s, 1H), 3.31 (s, 3H), 2.98 (s, 1H), 2.46 (s, 3H), 2.09–1.99 (m, 2H), 1.63–1.52 (m, 4H), 1.47–1.26 (m, 10H); ^{13}C NMR (150 MHz, $CDCl_3$): δ (ppm) 176.7, 155.1, 153.6, 144.8, 142.5, 139.2, 135.8, 134.3, 132.4, 131.7, 129.8, 129.6, 128.1, 126.7, 126.6, 126.5, 126.1, 124.3, 122.6, 122.4, 114.1, 108.4, 103.2, 61.6, 56.7, 50.1, 38.4, 33.8, 29.5, 29.4, 29.1, 29.0, 28.9, 26.6, 25.9, 21.6; HRMS (ESI-TOF) *m/z*: $[M + H]^+$ Calcd for $C_{38}H_{43}N_2O_3S^+$ 607.2989; Found 607.2994.

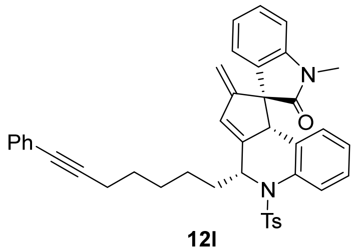

12l

Synthesis of 12l: To an oven-dried 10 mL Schlenk tube equipped with a magnetic stirring bar were added *tert*-butyl (12-phenyldodeca 1-en-3,11-diyn-5-yl) carbonate **10i** (70.5 mg, 0.200 mmol, 2.0 equiv), (*E*)-4-methyl-*N*-(2-((1-methyl-2-oxoindolin-3-ylidene)methyl) phenyl)benzenesulfonamide **11a** (40.4 mg, 0.0999 mmol, 1.0 equiv), Pd_2dba_3 (4.6 mg, 0.0050 mmol, 5 mol%), **L5** (4.6 mg, 0.012 mmol, 12 mol%) and $KHCO_3$ (12.0 mg, 0.120 mmol, 1.2 equiv). The tube was capped, evacuated and back-filled with argon for five times. Then degassed dry MeCN (1.0 mL) was added via syringe. The mixture was stirred at 45 °C for 72 h, and monitored by TLC (EtOAc/petroleum ether = 1/7). After completion, the solvent was evaporated in vacuo. The residue was purified by flash chromatography on silica gel (EtOAc/petroleum ether = 1/7) to give the product **12l**: 47.4 mg (0.0742 mmol), as a white solid, 74%

yield; mp 124–126 °C; >19:1 dr, determind by ^{1}H NMR analysis of crude product; $[\alpha_D^{25}]$ = –125.8 (*c* = 8.56 in $CHCl_3$); 94% ee, determined by HPLC analysis (Daicel Chiralpak ID, *n*-hexane/*i*-PrOH = 60/40, flow rate =1.0 mL/min, l = 254 nm) t_R = 9.01 min (minor), t_R = 12.36 min (major); ^{1}H NMR (600 MHz, $CDCl_3$): δ (ppm) 7.62 (d, *J* = 7.8 Hz, 1H), 7.44–7.36 (m, 2H), 7.35–7.26 (m, 5H), 7.21–7.15 (m, 3H), 7.14–7.10 (m, 1H), 6.88 (d, *J* = 7.8 Hz, 1H), 6.79–6.71 (m, 2H), 6.57 (d, *J* = 7.2 Hz, 1H), 6.03 (s, 1H), 5.73 (d, *J* = 7.8 Hz, 1H), 5.39–5.33 (m, 1H), 4.71 (s, 1H), 4.37 (s, 1H), 3.31 (s, 3H), 2.98 (s, 1H), 2.46 (s, 3H), 1.75–1.63 (m, 5H), 1.63–1.58 (m, 3H), 1.57–1.45 (m, 2H); ^{13}C NMR (100 MHz, $CDCl_3$): δ (ppm) 176.7, 155.0, 153.4, 144.9, 142.5, 135.8, 134.3, 132.4, 131.7, 131.6, 129.8, 129.7, 128.2, 128.1, 127.5, 126.8, 126.61, 126.58, 126.2, 124.4, 124.1, 122.7, 122.5, 108.5, 103.3, 90.7, 80.8, 61.6, 56.6, 50.1, 38.3, 28.7, 28.3, 26.7, 25.6, 21.7, 19.4; HRMS (ESI-TOF) *m/z*: $[M + Na]^+$ Calcd for $C_{41}H_{38}N_2O_3SNa^+$ 661.2495; Found 661.2496.

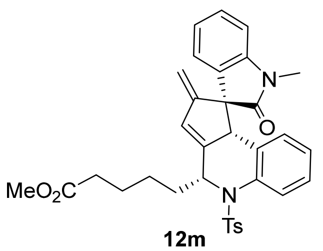
12m

Synthesis of 12m: To an oven-dried 10 mL Schlenk tube equipped with a magnetic stirring bar were added methyl 6-((*tert*-butoxycarbonyl)oxy)dec-9-en-7-ynoate **10j** (59.3 mg, 0.200 mmol, 2.0 equiv), (*E*)-4-methyl-*N*-(2-((1-methyl-2-oxoindolin-3-ylidene)methyl)phenyl)benzenesulfonamide **11a** (40.4 mg, 0.0999 mmol, 1.0 equiv), Pd_2dba_3 (4.6 mg, 0.0050 mmol, 5 mol%), **L5** (4.6 mg, 0.012 mmol, 12 mol%) and $KHCO_3$ (12.0 mg, 0.120 mmol, 1.2 equiv). The tube was capped, evacuated and back-filled with argon for five times. Then degassed dry MeCN (1.0 mL) was added via syringe. The mixture was stirred at 45 °C for 72 h, and monitored by TLC (EtOAc/petroleum ether = 1/6). After completion, the solvent was evaporated in vacuo. The residue was purified by flash chromatography on silica gel (EtOAc/petroleum ether = 1/6) to give the product **12m**: 29.9 mg (0.0513 mmol), as a white solid, 51% yield; mp 97–99 °C; >19:1 dr, determind by ^{1}H NMR analysis of crude product; $[\alpha_D^{25}]$ = –67.9 (*c* = 6.95 in $CHCl_3$); 95% ee, determined by HPLC analysis (Daicel Chiralpak IA, *n*-hexane/*i*-PrOH = 60/40, flow rate =1.0 mL/min, l = 254 nm) t_R = 6.71 min (minor), t_R = 11.76 min (major); ^{1}H NMR (400 MHz, $CDCl_3$): δ (ppm) 7.61 (d, *J* = 8.0 Hz, 1H), 7.31–7.27 (m, 2H), 7.22–7.10 (m, 4H), 6.88 (d, *J* = 7.6 Hz, 1H), 6.82–6.70 (m, 2H), 6.56 (d, *J* = 6.8 Hz, 1H), 6.07–6.00 (m, 1H), 5.73 (d, *J* = 7.6 Hz, 1H), 5.41–5.28 (m, 1H), 4.72 (s, 1H), 4.38 (s, 1H), 3.68 (s, 3H), 3.32 (s, 3H), 2.97 (s, 1H), 2.46 (s, 3H), 2.43–2.33 (m, 2H), 1.84–1.74 (m, 1H), 1.74–1.64 (m, 3H), 1.61–1.49 (m, 2H); ^{13}C NMR (100 MHz, $CDCl_3$): δ (ppm) 176.7, 174.1, 155.0,

153.2, 144.9, 142.5, 135.7, 134.1, 132.3, 131.7, 129.8, 129.6, 128.2, 126.8, 126.7, 126.6, 126.2, 124.5, 122.7, 122.5, 108.5, 103.4, 61.6, 56.4, 51.6, 50.0, 38.0, 34.0, 26.7, 25.5, 24.3, 21.7; HRMS (ESI-TOF) *m/z*: [M + Na]$^+$ Calcd for $C_{34}H_{34}N_2O_5SNa^+$ 605.2081; Found 605.2086.

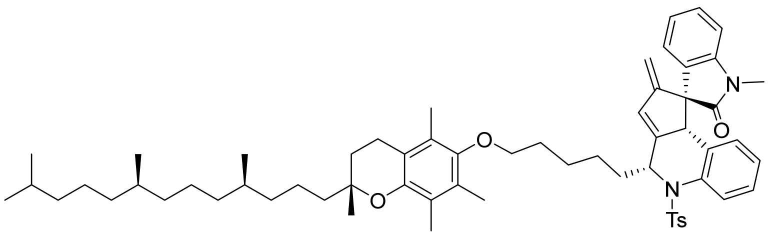

12n

Synthesis of 12n: To an oven-dried 10 mL Schlenk tube equipped with a magnetic stirring bar were added *tert*-butyl (10-(((*R*)-2,5,7,8-tetramethyl-2-((4*R*,8*R*)-4,8,12-trimethyltridecyl) chroman-6-yl)oxy)dec-1-en-3-yn-5-yl) carbonate **10k** (136.2 mg, 0.2000 mmol), (*E*)-4-methyl-*N*-(2-((1-methyl-2-oxoindolin-3-ylidene)methyl)phenyl)benzenesulfonamide **11a** (40.4 mg, 0.0999 mmol, 1.0 equiv), Pd_2dba_3 (4.6 mg, 0.0050 mmol, 5 mol%), **L5** (4.6 mg, 0.012 mmol, 12 mol%) and $KHCO_3$ (12.0 mg, 0.120 mmol, 1.2 equiv). The tube was capped, evacuated and back-filled with argon for five times. Then degassed dry MeCN (1.0 mL) was added via syringe. The mixture was stirred at 45 °C for 72 h, and monitored by TLC (EtOAc/petroleum ether = 1/6). After completion, the solvent was evaporated in vacuo. The residue was purified by flash chromatography on silica gel (EtOAc/petroleum ether = 1/6) to give the product **12n**: 59.1 mg (0.0611 mmol), as a white solid, 61% yield; mp 81–83 °C; >19:1 dr, determind by ^{1}H NMR analysis of crude product; $[\alpha_D^{25}]$ = –35.5 (*c* = 6.87 in $CHCl_3$); ^{1}H NMR (400 MHz, $CDCl_3$): δ (ppm) 7.62 (d, *J* = 8.0 Hz, 1H), 7.33–7.27 (m, 2H), 7.23–7.15 (m, 3H), 7.12 (t, *J* = 8.0 Hz, 1H), 6.88 (d, *J* = 7.6 Hz, 1H), 6.79–6.72 (m, 2H), 6.58 (d, *J* = 6.8 Hz, 1H), 6.07–6.02 (m, 1H), 5.73 (d, *J* = 7.6 Hz, 1H), 5.37 (t, *J* = 7.2 Hz, 1H), 4.72 (s, 1H), 4.38 (s, 1H), 3.66 (t, *J* = 6.4 Hz, 2H), 3.32 (s, 3H), 2.99 (s, 1H), 2.57 (t, *J* = 6.4 Hz, 2H), 2.46 (s, 3H), 2.16 (s, 3H), 2.12 (s, 3H), 2.08 (s, 3H), 1.85–1.62 (m, 8H), 1.58–1.20 (m, 20H), 1.15–1.01 (m, 6H), 0.88–0.83 (m, 12H); ^{13}C NMR (100 MHz, $CDCl_3$): δ (ppm) 176.7, 155.1, 153.5, 148.4, 147.7, 144.9, 142.5, 135.8, 134.3, 132.4, 131.7, 129.8, 129.7, 128.1, 127.8, 126.8, 126.6, 126.2, 125.8, 124.4, 122.8, 122.7, 122.5, 117.5, 108.5, 103.3, 74.8, 72.9, 61.6, 56.7, 50.1, 40.1, 39.4, 38.4, 37.6, 37.5, 37.4, 37.3, 32.8, 32.7, 31.3, 30.3, 28.0, 26.7, 26.1, 25.8, 24.84, 24.82, 24.5, 23.9, 22.8, 22.7, 21.7, 21.1, 20.7, 19.8, 19.7, 19.7, 19.6, 12.8, 12.0, 11.8; HRMS (ESI-TOF) *m/z*: [M + Na]$^+$ Calcd for $C_{62}H_{82}N_2O_5SNa^+$ 989.5837; Found 989.5841.

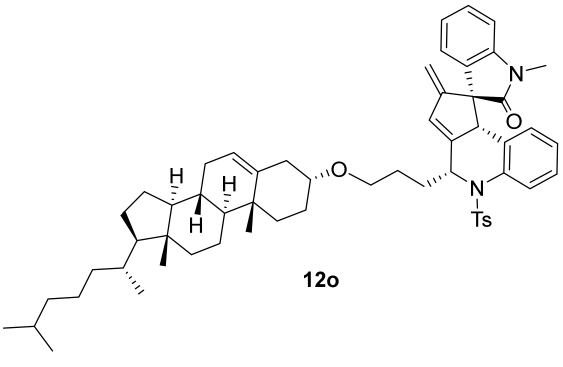

12o

Synthesis of 12o: To an oven-dried 10 mL Schlenk tube equipped with a magnetic stirring bar were added *tert*-butyl carbonate **10l** (121.8 mg, 0.2000 mmol), (*E*)-4-methyl-*N*-(2-((1-methyl-2-oxoindolin-3-ylidene) methyl)phenyl)benzenesulfonamide **11a** (40.4 mg, 0.0999 mmol, 1.0 equiv), Pd_2dba_3 (4.6 mg, 0.0050 mmol, 5 mol%), **L5** (4.6 mg, 0.012 mmol, 12 mol%) and $KHCO_3$ (12.0 mg, 0.120 mmol, 1.2 equiv). The tube was capped, evacuated and back-filled with argon for five times. Then degassed dry MeCN (1.0 mL) was added via syringe. The mixture was stirred at 45 °C for 72 h, and monitored by TLC (EtOAc/petroleum ether = 1/5). After completion, the solvent was evaporated in vacuo. The residue was purified by flash chromatography on silica gel (EtOAc/petroleum ether = 1/5) to give the product **12o**: 60.9 mg (0.0680 mmol), as a white solid, 68% yield; mp 110–112 °C; >19:1 dr, determind by ^{1}H NMR analysis of crude product; $[\alpha_D^{25}]$ = –27.9 (*c* = 8.17 in $CHCl_3$); ^{1}H NMR (400 MHz, $CDCl_3$): δ (ppm) 7.61 (d, *J* = 8.0 Hz, 1H), 7.31–7.27 (m, 2H), 7.22–7.16 (m, 3H), 7.15–7.10 (m, 1H), 6.88 (d, *J* = 7.6 Hz, 1H), 6.80–6.70 (m, 2H), 6.59 (d, *J* = 7.2 Hz, 1H), 6.06 (s, 1H), 5.73 (d, *J* = 7.6 Hz, 1H), 5.40–5.32 (m, 2H), 4.72 (s, 1H), 4.37 (s, 1H), 3.69–3.60 (m, 1H), 3.59–3.52 (m, 1H), 3.31 (s, 3H), 3.21–3.13 (m, 1H), 2.97 (s, 1H), 2.46 (s, 3H), 2.42–2.32 (m, 1H), 2.25–2.16 (m, 1H), 2.03–1.75 (m, 8H), 1.60–1.02 (m, 21H), 1.01 (s, 3H), 0.98–0.95 (m, 1H), 0.93–0.91 (m, 3H),, 0.87 (dd, *J* = 6.4, 1.6 Hz, 6H), 0.68 (s, 3H); ^{13}C NMR (100 MHz, $CDCl_3$): δ (ppm) 176.7, 155.0, 153.3, 144.9, 142.5, 141.0, 135.7, 134.2, 132.4, 131.7, 129.8, 129.7, 128.1, 126.8, 126.62, 126.59, 126.2, 124.6, 122.7, 122.6, 121.6, 121.5, 108.5, 103.4, 78.9, 66.8, 61.6, 56.8, 56.3, 56.2, 50.2, 50.1, 42.3, 39.8, 39.5, 39.2, 37.3, 36.9, 36.2, 35.8, 34.9, 32.0, 31.9, 28.55, 28.51, 28.3, 28.0, 26.7, 26.5, 24.3, 23.8, 22.8, 22.6, 21.7, 21.1, 19.4, 18.7, 11.9; HRMS (ESI-TOF) *m/z*: $[M + Na]^+$ Calcd for $C_{58}H_{74}N_2O_4SNa^+$ 917.5262; Found 917.5263.

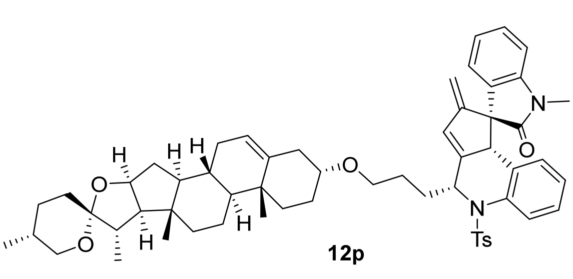

12p

Synthesis of 12p: To an oven-dried 10 mL Schlenk tube equipped with a magnetic stirring bar were added *tert*-butyl (1-(((4*S*,5'*S*,6a*S*,6b*R*,8a*R*,8b*S*, 9*R*, 10*S*,11a*R*,12a*R*,12b*R*)-5',6a,8a,9-tetramethyl-1,3,3', 4,4',5,5',6,6a,6b,6',7,8,8a,8b,9,11a,12,12a,12b-

icosahydrospiro[naphtho[2',1':4,5]indeno[2,1-*b*]furan-10,2'-pyran]-4-yl)oxy)oct-7-en-5-yn-4-yl) carbonate **10m** (127.4 mg, 0.2000 mmol), (*E*)-4-methyl-*N*-(2-((1-methyl-2-oxoindolin-3-ylidene) methyl)phenyl)benzenesulfonamide **11a** (40.4 mg, 0.0999 mmol, 1.0 equiv), Pd_2dba_3 (4.6 mg, 0.0050 mmol, 5 mol%), **L5** (4.6 mg, 0.012 mmol, 12 mol%) and $KHCO_3$ (12.0 mg, 0.120 mmol, 1.2 equiv). The tube was capped, evacuated and back-filled with argon for five times. Then degassed dry MeCN (1.0 mL) was added via syringe. The mixture was stirred at 45 °C for 72 h, and monitored by TLC (EtOAc/petroleum ether = 1/5). After completion, the solvent was evaporated in vacuo. The residue was purified by flash chromatography on silica gel (EtOAc/petroleum ether = 1/5) to give the product **12p**: 66.6 mg (0.0721 mmol), as a white solid, 72% yield; mp 107−109 °C; >19:1 dr, determind by ^{1}H NMR analysis of crude product; $[\alpha_D^{25}]$ = −76.9 (*c* = 5.84 in $CHCl_3$); ^{1}H NMR (400 MHz, $CDCl_3$): δ (ppm) 7.61 (d, *J* = 8.0 Hz, 1H), 7.30–7.27 (m, 2H), 7.20–7.15 (m, 3H), 7.15–7.10 (m, 1H), 6.88 (d, *J* = 7.6 Hz, 1H), 6.79–6.71 (m, 2H), 6.59 (d, *J* = 7.2 Hz, 1H), 6.06 (s, 1H), 5.73 (d, *J* = 7.6 Hz, 1H), 5.40–5.32 (m, 2H), 4.72 (s, 1H), 4.44–4.39 (m, 1H), 4.37 (s, 1H), 3.68–3.60 (m, 1H), 3.59–3.51 (m, 1H), 3.50–3.45 (m, 1H), 3.38 (t, *J* = 10.9 Hz, 1H), 3.32 (s, 3H), 3.21–3.13 (m, 1H), 2.97 (s, 1H), 2.46 (s, 3H), 2.42–2.33 (m, 1H), 2.24–2.15 (m, 1H), 2.06–1.66 (m, 12H), 1.61–1.40 (m, 8H), 1.34–1.05 (m, 5H), 1.03 (s, 3H), 1.00–0.94 (m, 4H), 0.81–0.76 (m, 6H); ^{13}C NMR (100 MHz, $CDCl_3$): δ (ppm) 176.7, 155.0, 153.3, 144.9, 142.5, 141.1, 135.7, 134.2, 132.4, 131.7, 129.8, 129.7, 128.1, 126.8, 126.62, 126.58, 126.2, 124.6, 122.7, 122.5, 121.30, 121.26, 109.3, 108.5, 103.4, 80.9, 78.9, 78.8, 66.9, 62.1, 61.6, 56.5, 56.3, 50.12, 50.06, 41.6, 40.3, 39.8, 39.2, 37.3, 37.1, 34.9, 32.1, 31.9, 31.5, 31.4, 30.3, 28.8, 28.52, 28.48, 26.7, 26.5, 21.7, 20.9, 19.5, 17.2, 16.3, 14.5; HRMS (ESI-TOF) *m/z*: [M + Na]$^+$ Calcd for $C_{58}H_{71}N_2O_6S^+$ 923.5027; Found 923.5032.

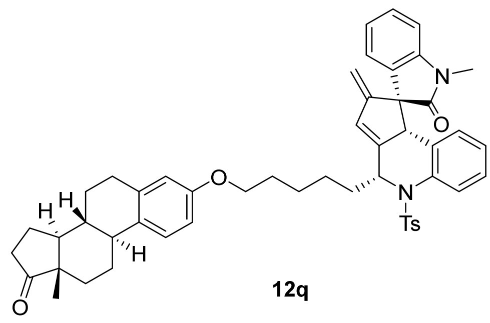

12q

Synthesis of 12q: To an oven-dried 10 mL Schlenk tube equipped with a magnetic stirring bar were added *tert*-butyl (10-(((8*S*,9*R*,13*R*,14*R*)-13-methyl-17-oxo-7,8,9,11,12,13,14,15,16,17-decahydro-6*H*-cyclopenta[*a*]phenanthrene-3-yl)oxy)dec-1-en-3-yn-5-yl) carbonate **10n** (104.2 mg, 0.2001 mmol), (*E*)-4-methyl-*N*-(2-((1-methyl-2-oxoindolin-3-ylidene)methyl)phenyl)benzenesulfonamide **11a** (40.4 mg, 0.0999 mmol, 1.0 equiv), Pd_2dba_3 (4.6 mg, 0.0050 mmol, 5 mol%), **L5** (4.6 mg, 0.012 mmol, 12 mol%) and $KHCO_3$ (12.0 mg,

0.120 mmol, 1.2 equiv). The tube was capped, evacuated and back-filled with argon for five times. Then degassed dry MeCN (1.0 mL) was added via syringe. The mixture was stirred at 45 °C for 72 h, and monitored by TLC (EtOAc/petroleum ether = 1/5). After completion, the solvent was evaporated in vacuo. The residue was purified by flash chromatography on silica gel (EtOAc/petroleum ether = 1/5) to give the product **12q**: 56.6 mg (0.0701 mmol), as a white solid, 70% yield; mp 133−134 °C; >19:1 dr, determind by ^{1}H NMR analysis of crude product; [α_D^{25}] = −88.5 (*c* = 7.54 in $CHCl_3$); ^{1}H NMR (400 MHz, $CDCl_3$): δ (ppm) 7.61 (d, *J* = 8.0 Hz, 1H), 7.31–7.27 (m, 2H), 7.23–7.09 (m, 5H), 6.88 (d, *J* = 8.0 Hz, 1H), 6.80–6.69 (m, 3H), 6.69–6.64 (m, 1H), 6.57 (d, *J* = 7.6 Hz, 1H), 6.03 (s, 1H), 5.73 (d, *J* = 7.6 Hz, 1H), 5.36 (t, *J* = 7.6 Hz, 1H), 4.71 (s, 1H), 4.37 (s, 1H), 3.97 (t, *J* = 6.0 Hz, 2H), 3.32 (s, 3H), 2.97 (s, 1H), 2.95–2.85 (m, 2H), 2.46 (s, 3H), 2.41–2.35 (m, 1H), 2.28–1.94 (m, 5H), 1.89–1.77 (m, 2H), 1.77–1.44 (m, 13H), 0.91 (s, 3H); ^{13}C NMR (100 MHz, $CDCl_3$): δ (ppm) 221.0, 176.7, 157.1, 155.0, 153.4, 144.9, 142.5, 137.7, 135.8, 134.2, 132.4, 131.9, 131.7, 129.8, 129.6, 128.1, 126.8, 126.63, 126.58, 126.3, 126.2, 124.4, 122.7, 122.5, 114.6, 112.2, 108.5, 103.4, 67.8, 61.6, 56.6, 50.4, 50.1, 48.1, 44.0, 38.4, 38.3, 35.9, 31.6, 29.7, 29.3, 26.7, 26.6, 26.0, 25.8, 25.6, 21.7, 21.6, 13.9; HRMS (ESI-TOF) *m/z*: [M + Na]$^+$ Calcd for $C_{51}H_{54}N_2O_5SNa^+$ 829.3646; Found 829.3645.

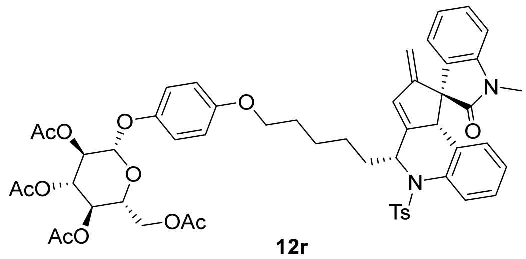

12r

Synthesis of 12r: To an oven-dried 10 mL Schlenk tube equipped with a magnetic stirring bar were added (2*R*,3*R*,4*S*,5*R*,6*S*)-2-(acetoxymethyl)-6-(4-((6-((*tert*-butoxycarbonyl)oxy)dec-9-en-7-yn-1-yl)oxy)phenoxy)tetrahydro-2*H*-pyran-3,4,5-triyl triacetate **10o** (138.2 mg, 0.2000 mmol), (*E*)-4-methyl-*N*-(2-((1-methyl-2-oxoindolin-3-ylidene)methyl)phenyl)benzenesulfonamide **11a** (40.4 mg, 0.0999 mmol, 1.0 equiv), Pd_2dba_3 (4.6 mg, 0.0050 mmol, 5 mol%), **L5** (4.6 mg, 0.012 mmol, 12 mol%) and $KHCO_3$ (12.0 mg, 0.120 mmol, 1.2 equiv). The tube was capped, evacuated and back-filled with argon for five times. Then degassed dry MeCN (1.0 mL) was added via syringe. The mixture was stirred at 45 °C for 72 h, and monitored by TLC (EtOAc/petroleum ether = 1/5). After completion, the solvent was evaporated in vacuo. The residue was purified by flash chromatography on silica gel (EtOAc/petroleum ether = 1/5) to give the product **12r**: 65.6 mg (0.0671 mmol), as a white solid, 67% yield; mp 126–128 °C; >19:1 dr, determind by ^{1}H NMR analysis of

crude product; $[\alpha_D^{25}] = -54.6$ (c = 8.65 in $CHCl_3$); ^{1}H NMR (400 MHz, $CDCl_3$): δ (ppm) 7.61 (d, J = 8.0 Hz, 1H), 7.31–7.28 (m, 1H), 7.22–7.16 (m, 3H), 7.15–7.10 (m, 1H), 6.96–6.79 (m, 6H), 6.78–6.71 (m, 2H), 6.56 (d, J = 7.6 Hz, 1H), 6.03 (s, 1H), 5.73 (d, J = 7.6 Hz, 1H), 5.37 (t, J = 7.2 Hz, 1H), 5.31–5.24 (m, 2H), 5.17 (t, J = 9.6 Hz, 1H), 4.96 (d, J = 7.2 Hz, 1H), 4.71 (s, 1H), 4.38 (s, 1H), 4.32–4.28 (m, 1H), 4.19–4.14 (m, 1H), 3.95 (t, J = 6.4 Hz, 2H), 3.84–3.79 (m, 1H), 3.32 (s, 3H), 2.96 (s, 1H), 2.46 (s, 3H), 2.09 (d, J = 2.0 Hz, 6H), 2.04 (d, J = 3.6 Hz, 6H), 1.87–1.79 (m, 2H), 1.75–1.53 (m, 6H); ^{13}C NMR (100 MHz, $CDCl_3$): δ (ppm) 176.7, 170.7, 170.3, 169.5, 169.4, 155.3, 155.0, 153.4, 150.8, 145.0, 142.5, 135.7, 134.2, 132.4, 131.6, 129.8, 129.6, 128.2, 126.8, 126.7, 126.5, 126.2, 124.4, 122.7, 122.4, 118.7, 115.2, 108.6, 103.4, 100.3, 72.8, 71.9, 71.2, 68.32, 68.29, 61.9, 61.6, 56.5, 50.0, 38.3, 29.2, 26.7, 25.7, 25.5, 21.7, 20.8, 20.73, 20.68, 20.65; HRMS (ESI-TOF) *m/z*: $[M + Na]^+$ Calcd for $C_{53}H_{56}N_2O_{14}SNa^+$ 999.3344; Found 999.3341.

6. Transformations of 1-aryl-4-alken-2-ynyl carbonates

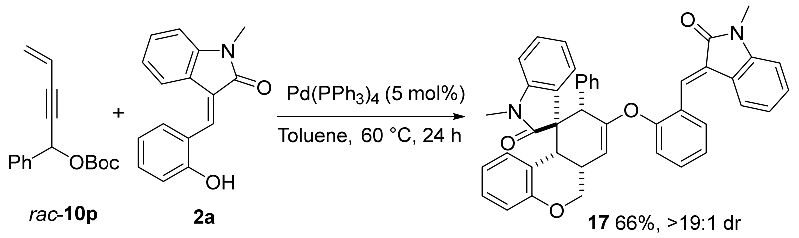

rac-**10p** **2a** **17** 66%, >19:1 dr

To an oven-dried 10 mL Schlenk tube equipped with a magnetic stirring bar were added *tert*-butyl (1-phenylpent-4-en-2-yn-1-yl) carbonate **10p** (51.7 mg, 0.200 mmol, 2.0 equiv), (*E*)-3-(2-hydroxybenzylidene)-1-methylindolin-2-one **2a** (25.1 mg, 0.0999 mmol, 1.0 equiv) and $Pd(PPh_3)_4$ (5.8 mg, 0.0050 mmol). The tube was capped, evacuated and back-filled with argon for five times. Then degassed dry toluene (1.0 mL) was added via syringe. The mixture was stirred at 60 °C for 24 h, and monitored by TLC (EtOAc/petroleum ether = 1/6). After completion, the solvent was evaporated in vacuo. The residue was purified by flash chromatography on silica gel (EtOAc/petroleum ether = 1/6) to give the product **17**: 42.5 mg (0.0661 mmol), as a white solid, 66% yield; mp 184−186 °C; >19:1 dr, determind by ^{1}H NMR analysis of crude product;^{1}H NMR (400 MHz, $CDCl_3$): δ (ppm) 7.76 (s, 1H), 7.56 (d, J = 7.6 Hz, 1H), 7.50 (d, J = 7.5 Hz, 1H), 7.45–7.40 (m, 1H), 7.29–7.26 (m, 1H), 7.25–7.23 (m, 1H), 7.21–7.13 (m, 3H), 6.97–6.78 (m, 10H), 6.67–6.62 (m, 1H), 6.57–6.53 (m, 1H), 6.23 (d, J = 7.6 Hz, 1H), 5.15–5.08 (m, 1H), 4.71 (t, J = 2.8 Hz, 1H), 4.25 (t, J = 11.4 Hz, 1H), 3.95 (d, J = 7.3 Hz, 1H), 3.80–3.70 (m, 1H), 3.33 (s, 3H), 3.15–3.07 (m, 1H),

2.86 (s, 3H); ^{13}C NMR (100 MHz, CDCl$_3$): δ (ppm) 178.7, 167.9, 154.8, 154.4, 153.3, 144.2, 144.0, 133.6, 133.2, 131.1, 130.9, 130.0, 129.9, 129.7, 128.5, 128.2, 128.1, 127.7, 127.3, 126.8, 126.6, 124.0, 122.8, 121.8, 121.2, 121.0, 120.4, 120.0, 116.9, 108.2, 107.7, 104.1, 64.9, 59.5, 50.8, 39.4, 33.7, 26.2, 26.0; HRMS (ESI-TOF) *m/z*: [M + Na]$^+$ Calcd for $C_{43}H_{34}N_2O_4Na^+$ 665.2411; Found 665.2419.

A possible mechanism for the formation of **17** was proposed. Activation of 1-phenyl-substituted propargylic carbonate *rac*-**10p** by Pd(0) led to the formation of thermally more stable allenyl-π-allyl species **Ib**, which underwent Tsuji–Trost reaction of with **2a** with distinct terminal regioselectivity. The resultant 1,2,3-butatriene intermediate **IIb** could be further HOMO-activated by Pd(0) via similar π-Lewis base catalysis, thus facilitated intramolecular vinylogous addition to generate η^3-propargylpalladium **IIIb**. Interestingly, **IIIb** was attacked by another **2a**, and subseqent protonation of **IVb** and intramolecular allylation of **Vb** provided complex **17** in a good yield with outstanding diastereo- and *E*/*Z*-selectivity.

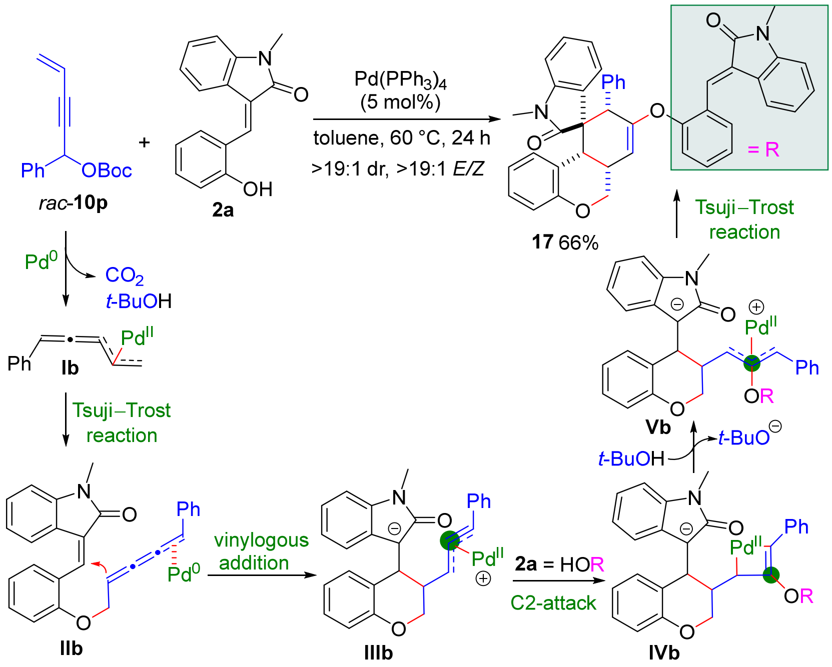

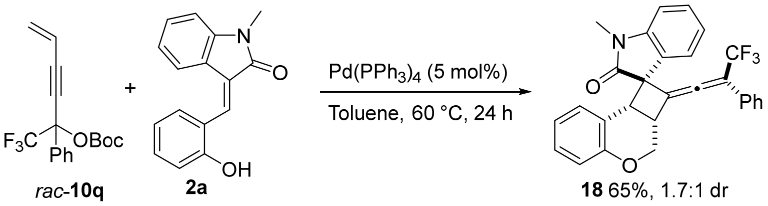

To an oven-dried 10 mL Schlenk tube equipped with a magnetic stirring bar were added *tert*-butyl (1,1,1-trifluoro-2-phenylhex-5-en-3-yn-2-yl) carbonate **10q** (65.3 mg, 0.200 mmol, 2.0 equiv), (*E*)-3-(2-hydroxybenzylidene)-1-methylindolin-2-one **2a** (25.1 mg, 0.0999 mmol, 1.0 equiv) and

Pd(PPh3)4 (5.8 mg, 0.0050 mmol). The tube was capped, evacuated and back-filled with argon for five times. Then degassed dry toluene (1.0 mL) was added via syringe. The mixture was stirred at 60 °C for 24 h, and monitored by TLC (EtOAc/petroleum ether = 1/7). After completion, the solvent was evaporated in vacuo. The residue was purified by flash chromatography on silica gel EtOAc/petroleum ether = 1/7 to 1/5) to give the products **18** and **18′** as seperable isomers.

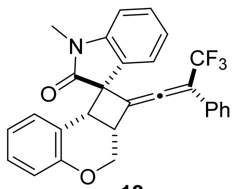

18

18: 18.9 mg (0.0411 mmol), as a white solid, 41% yield; mp 154–156 °C; ^{1}H NMR (400 MHz, $CDCl_3$): δ (ppm) 7.48–7.41 (m, 2H), 7.34–7.26 (m, 3H), 7.24–7.19 (m, 1H), 7.13–7.05 (m, 2H), 6.79–6.71 (m, 2H), 6.56–6.49 (m, 2H), 5.88 (d, *J* = 7.6 Hz, 1H), 4.56 (d, *J* = 9.2 Hz, 1H), 4.42 (d, *J* = 12.0 Hz, 1H), 4.03–3.97 (m, 2H), 3.25 (s, 3H); ^{13}C NMR (150 MHz, $CDCl_3$): δ (ppm) 195.5, 176.3, 155.9, 143.9, 132.1, 129.1, 128.9, 128.7, 128.6, 128.4, 127.1, 125.8, 125.2, 123.7, 122.2, 121.9, 121.6, 120.7, 117.7, 111.6, 107.8, 64.8, 59.7, 41.7, 41.5, 26.6; ^{19}F NMR (375 MHz, $CDCl_3$): δ (ppm) −60.1; HRMS (ESI-TOF) *m/z*: $[M + H]^+$ Calcd for $C_{28}H_{21}F_3NO_2^+$ 460.1519; Found 460.1523.

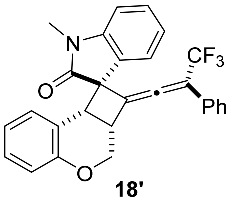

18'

18′: 11.1 mg (0.0241 mmol), as a white solid, 24% yield; mp 152–154 °C; ^{1}H NMR (400 MHz, $CDCl_3$): δ (ppm) 7.39–7.26 (m, 5H), 7.21–7.12 (m, 2H), 7.11–7.06 (m, 1H), 6.77–6.66 (m, 3H), 6.51 (d, *J* = 7.6 Hz, 1H), 6.13 (d, *J* = 7.5 Hz, 1H), 4.56–4.48 (m, 1H), 4.41 (d, *J* = 12.0 Hz, 1H), 4.06–3.94 (m, 2H), 3.24 (s, 3H); ^{13}C NMR (150 MHz, $CDCl_3$): δ (ppm) 195.3, 176.8, 156.2, 143.6, 131.7, 129.2, 128.9, 128.8, 128.6, 128.5, 127.1, 125.8, 125.6, 123.8, 122.5, 122.0, 121.4, 120.4, 117.9, 111.6, 107.7, 64.8, 59.3, 41.8, 41.5, 26.6; ^{19}F NMR (375 MHz, $CDCl_3$): δ (ppm) −59.7; HRMS (ESI-TOF) *m/z*: $[M + Na]^+$ Calcd for $C_{28}H_{20}F_3NO_2Na^+$ 482.1338; Found 482.1346.

The possible mechanism for the formation of **18** was proposed. (*E*)-3-(2-hydroxy benzylidene) oxindole **2a** underwent similar Tsuji–Trost reaction/vinylogous addition with **2a** efficiently through intermediates **Ic** and **IIc**, respectively, whereas the formed η^1-allenylpalladium moiety of **IIIc** was intramolecularly captured by enolate to provide intriguing cyclobutane-fused chromanone **18** having a tetrasubstituted exocyclic allene motif in a moderate yield with fair diastereoselectivity

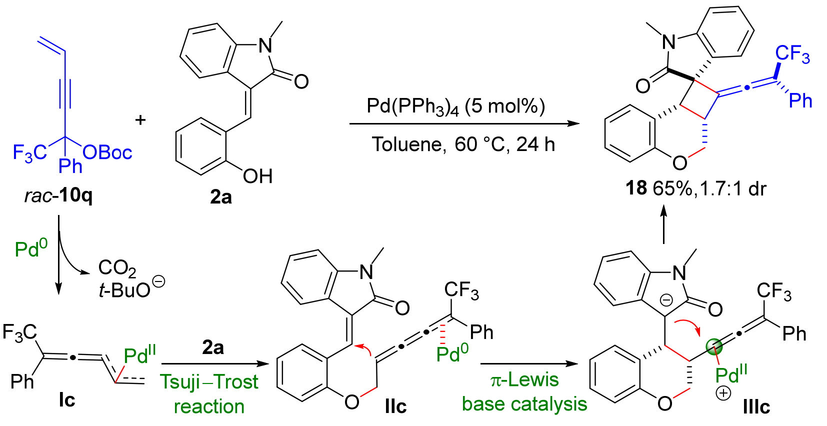

7. Unsuccessful substrates exploration

To further expand substrate scope, more *ortho*-functionalised activated alkenes derived from different backbones and primary carbonates were tested. Unfortunately, messy reaction profiles or no apparent conversions were generally observed, as summarised below.

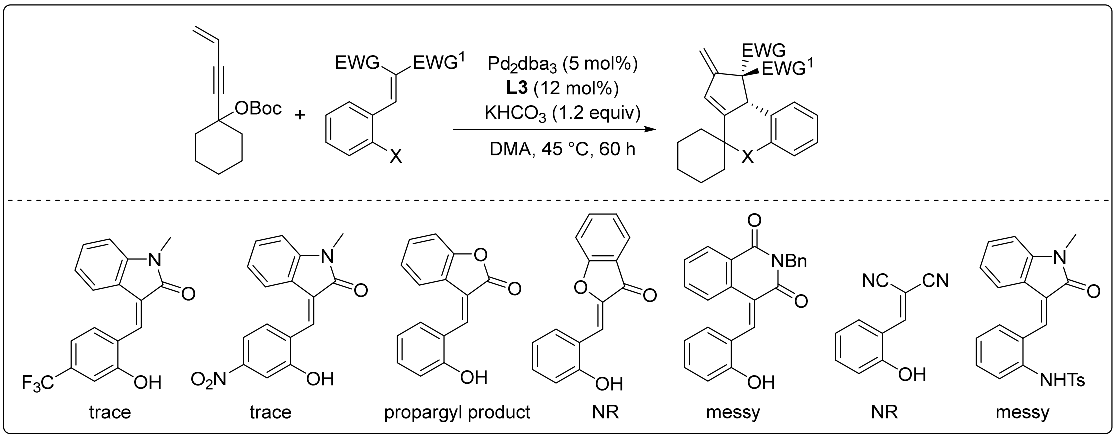

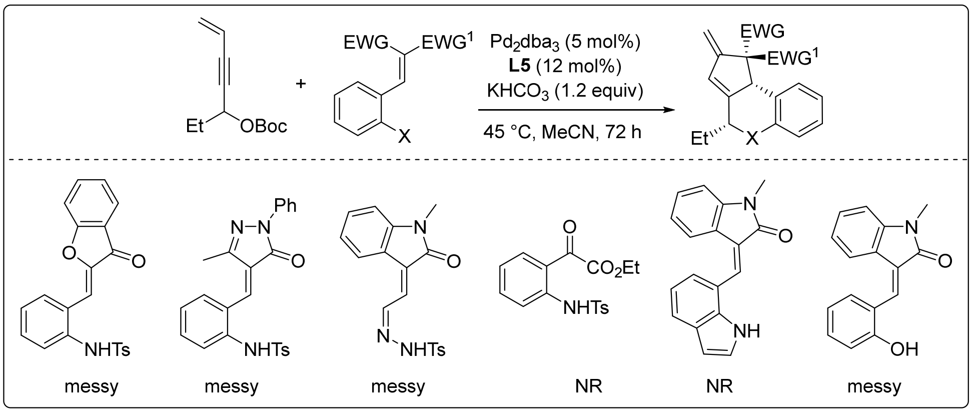

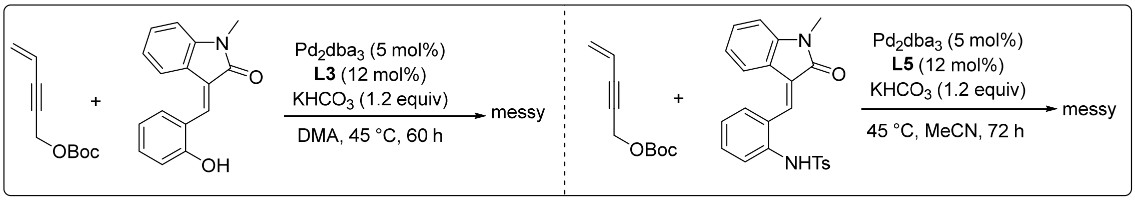

8. Transformations of product 12a

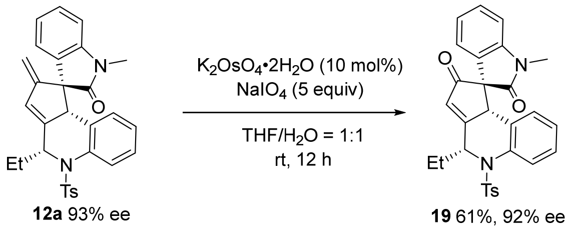

To a solution of **12a** (49.7 mg, 0.100 mmol) in THF/H_2O (0.5 mL/ 0.5 mL) was added $K_2OsO_4{\cdot}2H_2O$ (3.1 mg, 0.010 mmol) and $NaIO_4$ (108.0 mg, 0.5002 mmol). The mixture was stirred at room temperature for 10 h, and monitored by TLC (petroleumether/EtOAc = 5/1). After complete consumption of **12a**, the mixture was quenched with saturated aqueous Na_2SO_3, and extracted with ethyl acetate (3.0 mL × 2). The combined organic layers were washed with brine, dried over Na_2SO_4, filtered, and concentrated in vacuo. The residue was purified by flash chromatography on silica gel (petroleumether/EtOAc = 5/1) to afford product **19**: 30.5 mg (0.0612 mmol), as a white solid, 61% yield; mp 235–237 °C; >19:1 dr, determind by ^{1}H NMR analysis of crude product; $[\alpha_D^{25}]$ = −158.4 (*c* = 6.88 in $CHCl_3$); 92% ee, determined by HPLC analysis (Daicel Chiralpak AD, *n*-hexane/*i*-PrOH = 60/40, flow rate =1.0 mL/min, l = 254 nm) t_R = 6.85 min (minor), t_R = 11.40 min (major); ^{1}H NMR (400 MHz, $CDCl_3$): δ (ppm) 7.68 (d, *J* = 8.0 Hz, 1H), 7.35–7.29 (m, 2H), 7.26–7.14 (m, 4H), 6.91 (d, *J* = 8.0 Hz, 1H), 6.84 (t, *J* = 7.6 Hz, 1H), 6.76 (t, *J* = 7.6 Hz, 1H), 6.35 (d, *J* = 7.6 Hz, 1H), 6.01 (s, 1H), 5.78 (d, *J* = 7.6 Hz, 1H), 5.52–5.42 (m, 1H), 3.30 (s, 3H), 3.08 (s, 1H), 2.47 (s, 3H), 1.82–1.72 (m, 1H), 1.67–1.62 (m, 1H), 1.23 (t, *J* = 7.2 Hz, 3H); ^{13}C NMR (100 MHz, $CDCl_3$): δ (ppm) 198.7, 183.2, 172.7, 145.6, 144.3, 135.6, 134.4, 130.2, 129.74, 129.67, 129.0, 127.6, 127.1, 126.7, 126.4, 125.1, 123.6, 122.9, 122.4, 109.1, 66.5, 59.0, 46.6, 31.2, 26.8, 21.7, 10.9; HRMS (ESI-TOF) *m/z*: $[M + Na]^+$ Calcd for $C_{29}H_{26}N_2O_4SNa^+$ 521.1505; Found 521.1509.

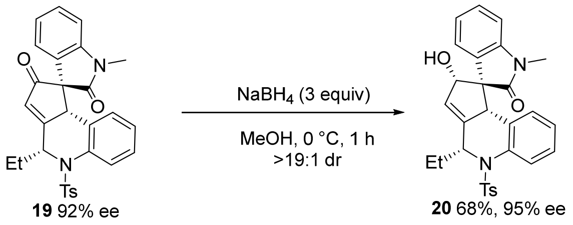

To a solution of **19** (30.5 mg, 0.0612 mmol) in MeOH (0.5 mL) was added $NaBH_4$ (7.0 mg, 0.185 mmol) at 0 °C. The mixture was stirred at 0 °C for 1 h, and monitored by TLC (petroleumether/EtOAc = 3/1). After complete consumption of **19**, the mixture was quenched with

water, and extracted with ethyl acetate (3.0 mL × 2). The combined organic layers were washed with brine, dried over Na_2SO_4, filtered, and concentrated in vacuo, The residue was purified by flash chromatography on silica gel (petroleumether/EtOAc = 3/1) to afford product **20**: 23.3 mg (0.0416 mmol), as a white solid, 68% yield; mp 178–180 °C; >19:1 dr, determind by ^{1}H NMR analysis of crude product; $[\alpha_D^{25}]$ = –190.5 (*c* = 3.54 in $CHCl_3$); 95% ee, determined by HPLC analysis (Daicel Chiralpak AD, *n*-hexane/*i*-PrOH = 60/40, flow rate =1.0 mL/min, l = 254 nm) t_R = 4.68 min (minor), t_R = 12.62 min (major); ^{1}H NMR (400 MHz, $CDCl_3$): δ (ppm) 7.56 (d, *J* = 7.6 Hz, 1H), 7.36–7.30 (m, 2H), 7.26–7.18 (m, 3H), 7.15–7.08 (m, 1H), 6.89 (d, *J* = 8.0 Hz, 1H), 6.83–6.74 (m, 3H), 5.79 (d, *J* = 8.0 Hz, 1H), 5.52 (s, 1H), 5.12–5.05 (m, 1H), 4.93 (d, *J* = 11.2 Hz, 1H), 3.28 (s, 3H), 2.62 (s, 1H), 2.52 (s, 3H), 1.60–1.45 (m, 2H), 1.40–1.33 (m, 1H), 1.15 (t, *J* = 7.2 Hz, 3H); ^{13}C NMR (100 MHz, $CDCl_3$): δ (ppm) 176.8, 147.5, 145.0, 144.7, 136.0, 134.1, 133.1, 129.9, 129.8, 129.2, 127.0, 126.8, 126.7, 125.1, 124.8, 124.5, 123.9, 122.3, 108.7, 83.7, 65.6, 57.4, 47.6, 30.8, 26.7, 21.7, 10.8; HRMS (ESI-TOF) *m/z*: $[M + Na]^+$ Calcd for $C_{29}H_{28}N_2O_4SNa^+$ 523.1662; Found 523.1672.

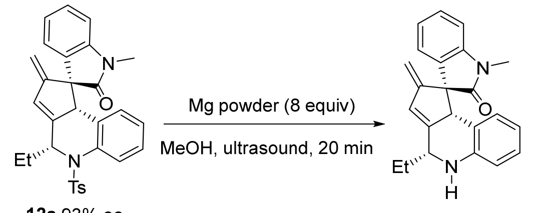

12a 93% ee

21 86%, 91% ee

To a solution of **12a** (49.7 mg, 0.100 mmol) in MeOH (1.0 mL) was added Mg powder (19.5 mg, 0.802 mmol), and the resultant mixture was sonicated for 20 minutes. After complete consumption of **12a**, the mixture was quenched with water, and extracted with ethyl acetate (3.0 mL × 2). The combined organic layers were washed with brine, dried over Na_2SO_4, filtered, and concentrated in vacuo, The residue was purified by flash chromatography on silica gel (petroleumether/EtOAc = 5/1) to afford product **21**: 29.5 mg (0.0861 mmol), as a white solid, 86% yield; mp 132–134 °C; >19:1 dr, determind by ^{1}H NMR analysis of crude product; $[\alpha_D^{25}]$ = –232.5 (*c* = 4.67 in $CHCl_3$); 91% ee, determined by HPLC analysis (Daicel Chiralpak AD, *n*-hexane/*i*-PrOH = 80/20, flow rate =1.0 mL/min, l = 254 nm) t_R = 6.84 min (minor), t_R = 8.78 min (major); ^{1}H NMR (400 MHz, $CDCl_3$): δ (ppm) 7.13–7.04 (m, 1H), 6.83–6.74 (m, 2H), 6.72–6.64 (m, 2H), 6.49 (d, *J* = 7.6 Hz, 1H), 6.29–6.23 (m, 1H), 6.17 (s, 1H), 6.08 (d, *J* = 7.6 Hz, 1H), 4.93 (s, 1H), 4.80 (s, 1H), 4.51 (s, 1H), 3.97–3.80 (m, 2H), 3.38 (s, 3H), 2.10–1.98 (m, 1H), 1.82–1.72 (m, 1H), 1.15 (t, *J* = 7.2 Hz, 3H); ^{13}C NMR (100 MHz, $CDCl_3$): δ (ppm) 179.0, 156.3, 150.8, 145.0, 142.7, 132.6, 128.0, 127.6,

127.0, 123.6, 123.0, 122.7, 120.6, 118.2, 114.9, 108.0, 104.6, 63.2, 53.9, 53.3, 26.8, 26.1, 10.4; HRMS (ESI-TOF) *m/z*: $[M + H]^+$ Calcd for $C_{23}H_{23}N_2O^+$ 343.1805; Found 343.1809.

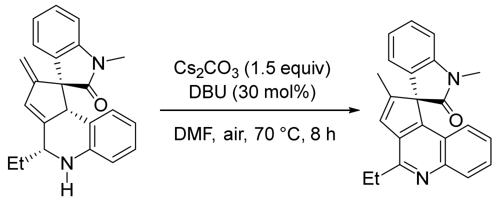

21 91% ee **22** 89%, 90% ee

To a solution of **21** (29.5 mg, 0.0861 mmol) in DMF (0.8 mL) was added Cs_2CO_3 (42.1 mg, 0.129 mmol) and DBU (4.0 mg, 0.026 mmol). The mixture was stirred at 70 °C for 8 h and monitored by TLC (petroleumether/EtOAc = 6/1). After complete consumption of **21**, the mixture was quenched with water, and extracted with ethyl acetate (3.0 mL × 3). The combined organic layers were washed with brine, dried over Na_2SO_4, filtered, and concentrated in vacuo. The residue was purified by flash chromatography on silica gel (petroleumether/EtOAc = 6/1) to afford product **22**: 26.1 mg (0.0767 mmol), as a white solid, 89% yield; mp 105–107 °C; $[\alpha_D^{25}]$ = –57.3 (*c* = 4.89 in $CHCl_3$); 90% ee, determined by HPLC analysis (Daicel Chiralpak AD, *n*-hexane/*i*-PrOH = 80/20, flow rate =1.0 mL/min, l = 254 nm) t_R = 5.98 min (minor), t_R = 13.51 min (major); ^1H NMR (400 MHz, $CDCl_3$): δ (ppm) 8.02 (d, *J* = 8.8 Hz, 1H), 7.50–7.43 (m, 1H), 7.42–7.37 (m, 1H), 7.20–7.15 (m, 1H), 7.10 (d, *J* = 8.0 Hz, 1H), 7.00–6.94 (m, 1H), 6.91–6.88 (m, 1H), 6.85 (d, *J* = 8.0 Hz, 1H), 6.65 (d, *J* = 7.6 Hz, 1H), 3.46 (s, 3H), 3.16 (q, *J* = 7.6,7.6 Hz, 2H), 1.79 (s, 3H), 1.47 (t, *J* = 7.6 Hz, 3H); ^{13}C NMR (100 MHz, $CDCl_3$): δ (ppm) 173.7, 157.3, 148.9, 148.4, 146.7, 144.7, 138.3, 129.8, 129.2, 127.8, 127.8, 127.4, 126.4, 123.6, 123.5, 123.1, 121.4, 108.9, 68.4, 29.9, 27.2, 13.7, 12.9; HRMS (ESI-TOF) *m/z*: $[M + H]^+$ Calcd for $C_{23}H_{21}N_2O^+$ 341.1648; Found 341.1652.

It should be noted that the oxindole-fused polycyclic products and their derivatives are core structural subunits of many bioactive natural products and drug candidates, as briefly exemplified below.

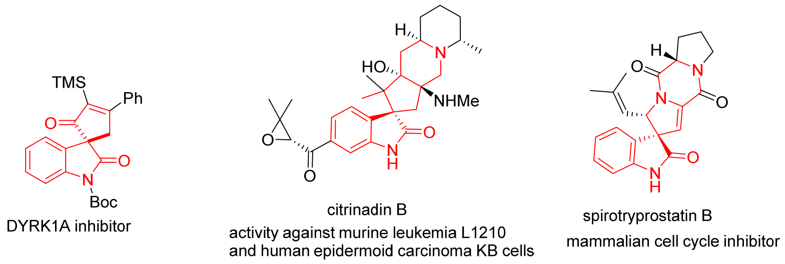

(*a*) M. Banerjee, H. Liu, O. Wiest, J. Zartman, and B. L. Ashfeld, *ChemMedChem* 2022, **17**, e202100512;
(*b*) T. Mugishima, M. Tsuda, Y. Kasai, H. Ishiyama, E. Fukushi, J. Kawabata, M. Watanabe, K. Akao, and J. Kobayashi, *J. Org. Chem.* 2005, **70**, 9430–9435;
(*c*) C.-B. Cui, H. Kakeya, and H. Osada, *J. Antibiot.* 1996, **49**, 832–835.

FigureS1. Selected bioactive spirooxindoles

9. Preliminary mechanism investigation

9.1 Kinetic studies of the reaction between 10a and 11a

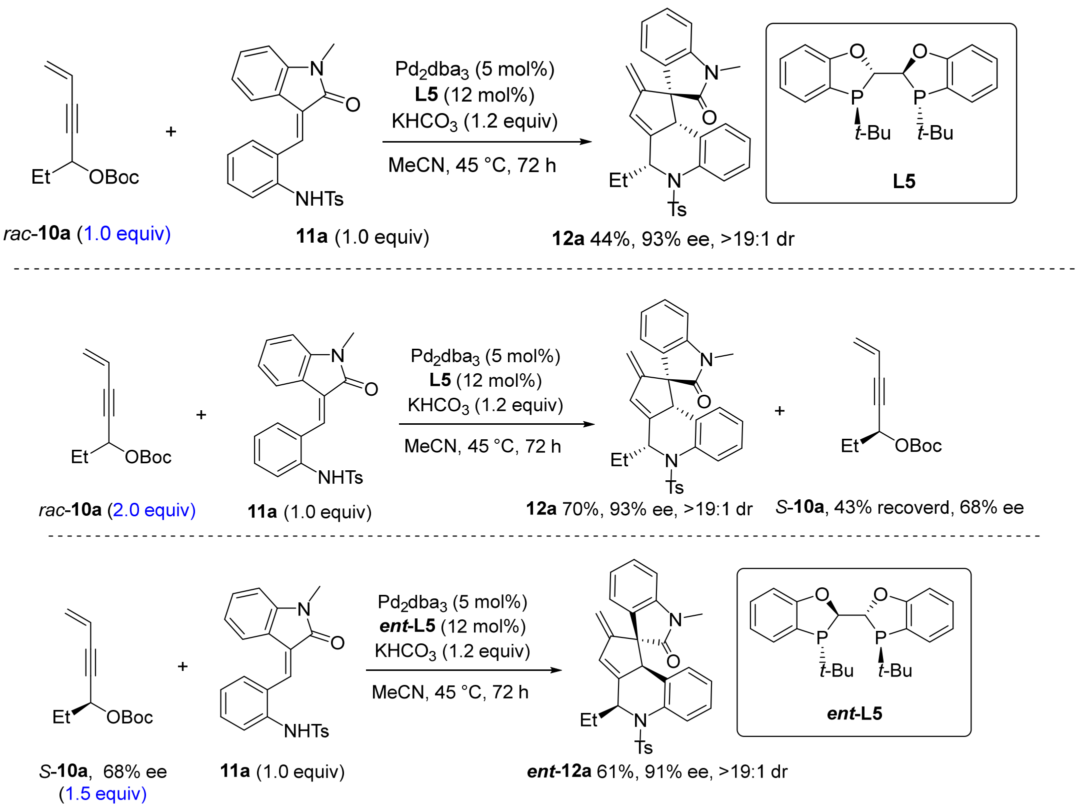

The reaction of *rac*-**10a** (1.0 equiv) with (2-aminobenzylidene)oxindole **11a** delivered product **12a** in 44% yield under standard conditions, while a significantly improved yiled was attained by increasing the amounts of *rac*-**10a** (2.0 equiv). Meanwhile, *S*-**10a** was recovered in 43% yield with 68% ee. In addition, the reaction of optically active (*S*)-**10a** under the standard conditions with *ent*-**L5** as ligand afforded product *ent*-**12a** in 61% yield with 91% ee. These results indicated that a kinetic resolution process of *rac*-**10a** was involved in current tandem transformations.

9.2 Elucidation of the origin of regioselectivity

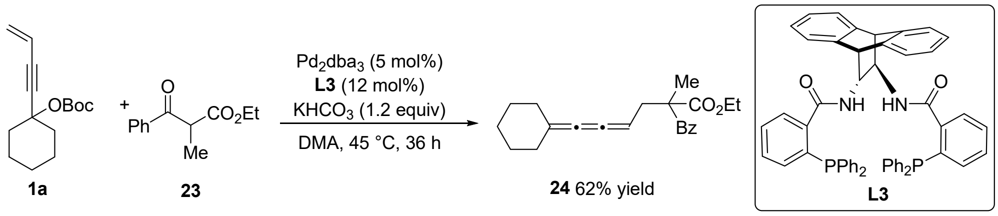

To shed some light on the origin of the unusual regioselectivity and the complicated reaction process, some control experiments were conducted. As depicted above, the assembly of 4-alken-2-ynyl carbonate **1a** with carbon-centered nucleophile β-ketoesters **23** delivered 1,2,3-butatriene product **24** under the standard conditions, which indicated that the regioselectivity for substitution was governed by the nucleophiles.

9.3 Elucidation of the reaction pathway

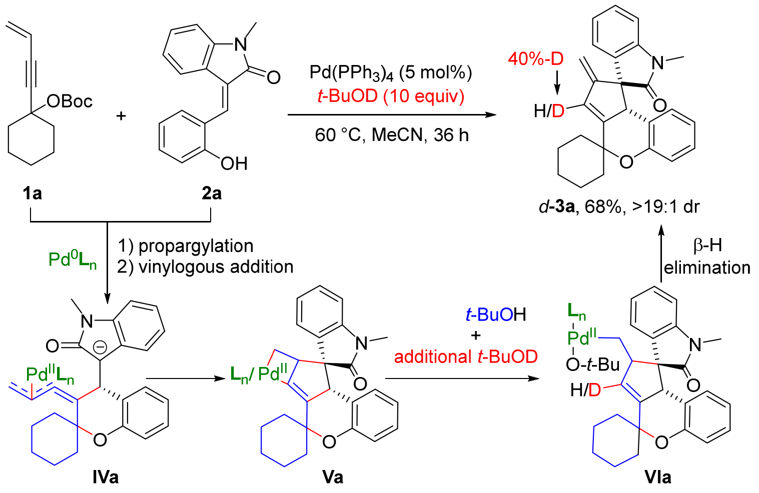

Moreover, adding *t*-BuOD to the reaction mixture led to product ***d*-3a** with 40% deuterium incorporated into *endo*-cyclic double bond, as outlined above. As nucleophilic attack on the central carbon of π-allylpalladium had been documented (*JACS* **2007**, *129*, 14866; *JACS* **2010**, *132*, 7508; *Org. Lett.* **2024**, *26*, 5782), this result further indicated that the key palladacyclobutane **Va** indeed be involved in the reaction process.

Procedure for deuterium labeling experiments

To an oven-dried 10 mL Schlenk tube equipped with a magnetic stirring bar were added 1-(but-3-en-1-yn-1-yl)cyclohexyl *tert*-butyl carbonate **1a** (37.6 mg, 0.150 mmol, 1.5 equiv), (*E*)-3-(2-hydroxybenzylidene)-1-methylindolin-2-one **2a** (25.1 mg, 0.0999 mmol, 1.0 equiv), $Pd(PPh_3)_4$ (5.8 mg, 0.0050 mmol) and *t*-BuOD (75.1 mg, 1.00 mmol). The tube was capped, evacuated and back-filled with argon for five times. Then degassed dry MeCN (2.0 mL) was added via syringe. The mixture was stirred at 60 °C for 36 h, and monitored by TLC (EtOAc/petroleum ether = 1/6). After completion, the solvent was evaporated in vacuo. The residue was purified by flash chromatography on silica gel (EtOAc/petroleum ether = 1/6) to give the product ***d*-3a**: 26.1 mg (0.0681 mmol), as a white solid, 68% yield; 40%-D; ^{1}H NMR (400 MHz, $CDCl_3$): δ (ppm) 7.12–7.05 (m, 1H), 6.94–6.88 (m, 1H), 6.84–6.75 (m, 2H), 6.69–6.60 (m, 2H), 6.48–6.41 (m, 1H), 6.24 (d, J = 2.8 Hz, 0.6H), 6.17 (d, J = 7.6 Hz, 1H), 4.96 (s, 1H), 4.76 (s, 1H), 4.55 (s, 1H), 3.40 (s, 3H), 2.13–2.05 (m, 1H), 2.05–1.93 (m, 3H), 1.81–1.68 (m, 2H), 1.59–1.31 (m, 4H).

10. Crystal data and structural refinement

Procedure for the recrystallization of 3o: To a 10 mL tube containing enantiopure **3o** (32 mg) were added *n*-hexane (0.3 mL) and EtOAc (1.4 mL). The mixture was heated until a clear solution was formed, which was kept aside at room temperature to obtain crystals. The crystals were subjected for single crystal XRD to determine the absolute configuration of **3o**. The data were collected by an Bruker D8 Venture equipped with a Cu radiation source (Kα = 1.54178 Å) at 273.0 K. CCDC 2381131 (**3o**) contains the supplementary crystallographic data for this paper. These data can be obtained free of charge via www.ccdc.cam.ac.uk/data_request/cif.

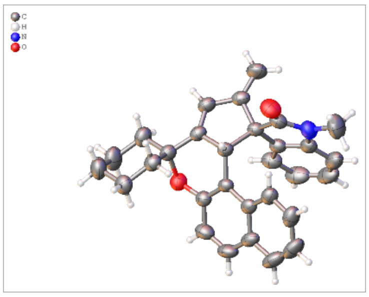

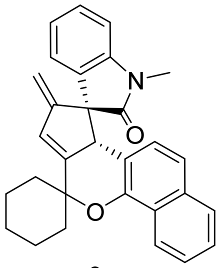

3o

Identification code	**3o**
Empirical formula	$C_{30}H_{27}NO_2$
Formula weight	433.52
Temperature/K	273.0
Crystal system	monoclinic
Space group	$P2_1$
a/Å	8.6278(4)
b/Å	12.3083(6)
c/Å	10.9519(5)
α/°	90
β/°	95.711(3)
γ/°	90
Volume/Å^3	1157.25(9)
Z	2
ρ_{calc}g/cm^3	1.244
μ/mm^{-1}	0.604
F(000)	460.0
Crystal size/mm^3	0.38 × 0.25 × 0.1
Radiation	CuKα (λ = 1.54178)
2Θ range for data collection/°	8.112 to 140.378

Index ranges	-10 ⩽ h ⩽ 10, -15 ⩽ k ⩽ 14, -13 ⩽ l ⩽ 12
Reflections collected	7672
Independent reflections	3988 [R_{int} = 0.0402, R_{sigma} = 0.0528]
Data/restraints/parameters	3988/1/300
Goodness-of-fit on F^2	1.077
Final R indexes [I>=2σ (I)]	R_1 = 0.0380, wR_2 = 0.0971
Final R indexes [all data]	R_1 = 0.0437, wR_2 = 0.1013
Largest diff. peak/hole / e Å^{-3}	0.16/-0.12
Flack parameter	-0.01(18)

Procedure for the recrystallization of 12g: To a 10 mL tube containing enantiopure **12g** (45 mg) were added *n*-hexane (0.5 mL) and EtOAc (1.5 mL). The mixture was heated until a clear solution was formed, which was kept aside at room temperature to obtain crystals. The crystals were subjected for single crystal XRD to determine the absoulte configuration of **12g**. The data were collected by an Bruker D8 Venture equipped with a Mo radiation source (Kα = 0.71073 Å) at 303.0 K. CCDC 2381132 (**12g**) contains the supplementary crystallographic data for this paper. These data can be obtained free of charge via www.ccdc.cam.ac.uk/data_request/cif.

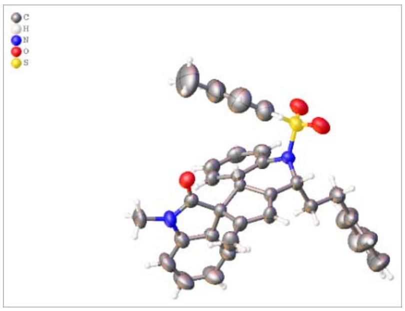

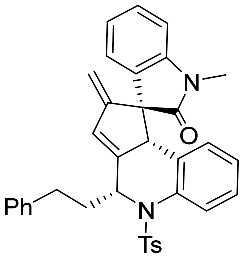

12g

Identification code	**12g**
Empirical formula	$C_{36}H_{32}N_2O_3S$
Formula weight	572.69
Temperature/K	303.0
Crystal system	tetragonal
Space group	$P4_32_12$
a/Å	12.4257(6)
b/Å	12.4257(6)
c/Å	42.988(4)
α/°	90
β/°	90

γ/°	90
Volume/Å^3	6637.3(9)
Z	8
ρ_{calc}g/cm^3	1.146
μ/mm^{-1}	0.133
F(000)	2416.0
Crystal size/mm^3	0.48 × 0.42 × 0.26
Radiation	MoKα (λ = 0.71073)
2Θ range for data collection/°	3.786 to 54.894
Index ranges	$-12 \leqslant h \leqslant 15, -16 \leqslant k \leqslant 11, -55 \leqslant l \leqslant 41$
Reflections collected	22897
Independent reflections	7524 [R_{int} = 0.0566, R_{sigma} = 0.0770]
Data/restraints/parameters	7524/7/381
Goodness-of-fit on F^2	0.985
Final R indexes [I>=2σ (I)]	R_1 = 0.0542, wR_2 = 0.1043
Final R indexes [all data]	R_1 = 0.1202, wR_2 = 0.1296
Largest diff. peak/hole / e Å^{-3}	0.14/-0.21
Flack parameter	0.00(6)

Procedure for the recrystallization of 17: To a 10 mL tube containing racemic **17** (48 mg) were added *n*-hexane (0.5 mL) and EtOAc (1.5 mL). The mixture was heated until a clear solution was formed, which was kept aside at room temperature to obtain crystals. The crystals were subjected for single crystal XRD to determine the relative configuration of **17**. The data were collected by an Bruker D8 Venture equipped with a Mo radiation source (Kα = 0.71073 Å) at 304.0 K. CCDC 2381133 (**17**) contains the supplementary crystallographic data for this paper. These data can be obtained free of charge via www.ccdc.cam.ac.uk/data_request/cif.

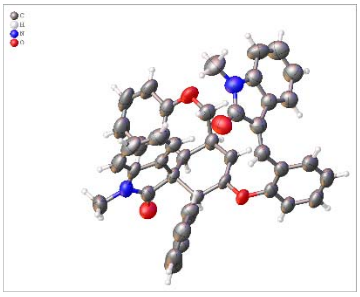

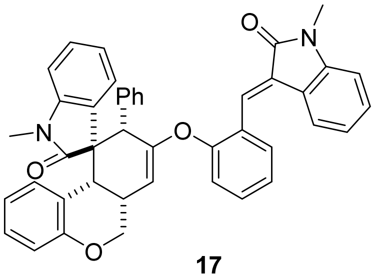

17

17

Identification code	
Empirical formula	$C_{43}H_{34}N_2O_4$
Formula weight	642.72
Temperature/K	304.0
Crystal system	monoclinic
Space group	C2/c
a/Å	21.731(4)
b/Å	9.1510(15)
c/Å	34.748(7)
α/°	90
β/°	107.204(9)
γ/°	90
Volume/Å^3	6601(2)
Z	8
ρ_{calc}g/cm^3	1.293
μ/mm^{-1}	0.083
F(000)	2704.0
Crystal size/mm^3	0.12 × 0.07 × 0.05
Radiation	MoKα (λ = 0.71073)
2Θ range for data collection/°	3.924 to 49.98
Index ranges	-25 ⩽ h ⩽ 25, -10 ⩽ k ⩽ 10, -41 ⩽ l ⩽ 41
Reflections collected	34640
Independent reflections	5737 [R_{int} = 0.1452, R_{sigma} = 0.1134]
Data/restraints/parameters	5737/0/445
Goodness-of-fit on F^2	1.025
Final R indexes [I>=2σ (I)]	R_1 = 0.0831, wR_2 = 0.1892
Final R indexes [all data]	R_1 = 0.1959, wR_2 = 0.2470
Largest diff. peak/hole / e Å^{-3}	0.24/-0.25

Procedure for the recrystallization of 18: To a 10 mL tube containing racemic **18** (42 mg) were added *n*-hexane (0.5 mL) and EtOAc (1.4 mL). The mixture was heated until a clear solution was formed, which was kept aside at room temperature to obtain crystals. The crystals were subjected for single crystal XRD to determine the relative configuration of **18**. The data were collected by an Bruker

D8 Venture equipped with a Mo radiation source (Kα = 0.71073 Å) at 227.0 K. CCDC 2381134 (**18**) contains the supplementary crystallographic data for this paper. These data can be obtained free of charge via www.ccdc.cam.ac.uk/data_request/cif.

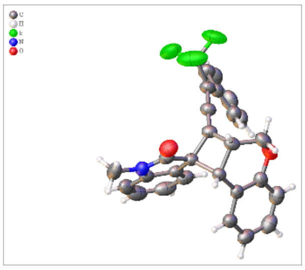

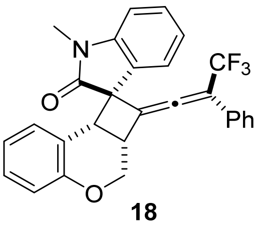

18

Identification code	**18**
Empirical formula	$C_{28}H_{20}F_3NO_2$
Formula weight	459.45
Temperature/K	227.0
Crystal system	triclinic
Space group	P-1
a/Å	9.9196(10)
b/Å	9.9973(9)
c/Å	11.8824(11)
α/°	91.791(4)
β/°	104.104(4)
γ/°	102.384(3)
Volume/Å^3	1111.91(18)
Z	2
ρ_{calc}g/cm^3	1.372
μ/mm^{-1}	0.103
F(000)	476.0
Crystal size/mm^3	0.45 × 0.37 × 0.1
Radiation	MoKα (λ = 0.71073)
2Θ range for data collection/°	4.858 to 55.012
Index ranges	-12 ≤ h ≤ 12, -11 ≤ k ≤ 12, -15 ≤ l ≤ 15
Reflections collected	18358
Independent reflections	5062 [R_{int} = 0.0935, R_{sigma} = 0.0893]
Data/restraints/parameters	5062/0/308

Goodness-of-fit on F^2	1.055
Final R indexes [I>=2σ (I)]	R_1 = 0.0703, wR_2 = 0.1888
Final R indexes [all data]	R_1 = 0.1065, wR_2 = 0.2161
Largest diff. peak/hole / e Å^{-3}	0.25/-0.28

Procedure for the recrystallization of 20: To a 10 mL tube containing racemic **20** (37 mg) were added *n*-hexane (0.4 mL) and EtOAc (1.4 mL). The mixture was heated until a clear solution was formed, which was kept aside at room temperature to obtain crystals. The crystals were subjected for single crystal XRD to determine the relative configuration of **20**. The data were collected by an Bruker D8 Venture equipped with a Mo radiation source (Kα = 0.71073 Å) at 150.0 K. CCDC 2381135 (**20**) contains the supplementary crystallographic data for this paper. These data can be obtained free of charge via www.ccdc.cam.ac.uk/data_request/cif.

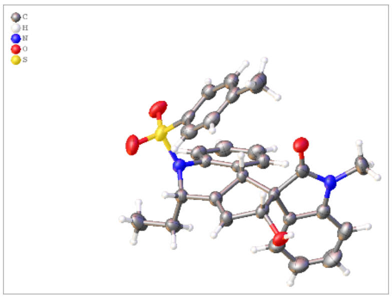

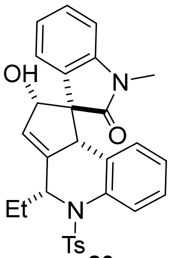

20

Identification code	**20**
Empirical formula	$C_{29}H_{28}N_2O_4S$
Formula weight	500.59
Temperature/K	150.0
Crystal system	triclinic
Space group	P-1
a/Å	7.4687(15)
b/Å	12.636(2)
c/Å	14.057(3)
α/°	86.841(7)
β/°	76.742(7)
γ/°	75.308(7)
Volume/Å^3	1249.1(4)
Z	2
ρ_{calc}g/cm^3	1.331
μ/mm^{-1}	0.168
F(000)	528.0

Crystal size/mm^3	0.24 × 0.15 × 0.1
Radiation	MoKα (λ = 0.71073)
2Θ range for data collection/°	4.462 to 49.992
Index ranges	-8 ⩽ h ⩽ 8, -15 ⩽ k ⩽ 15, -16 ⩽ l ⩽ 16
Reflections collected	17419
Independent reflections	4281 [R_{int} = 0.0923, R_{sigma} = 0.0765]
Data/restraints/parameters	4281/0/330
Goodness-of-fit on F^2	1.113
Final R indexes [I>=2σ (I)]	R_1 = 0.0965, wR_2 = 0.2626
Final R indexes [all data]	R_1 = 0.1265, wR_2 = 0.2881
Largest diff. peak/hole / e Å^{-3}	0.90/-0.44

11. NMR, HRMS spectra and HPLC chromatograms

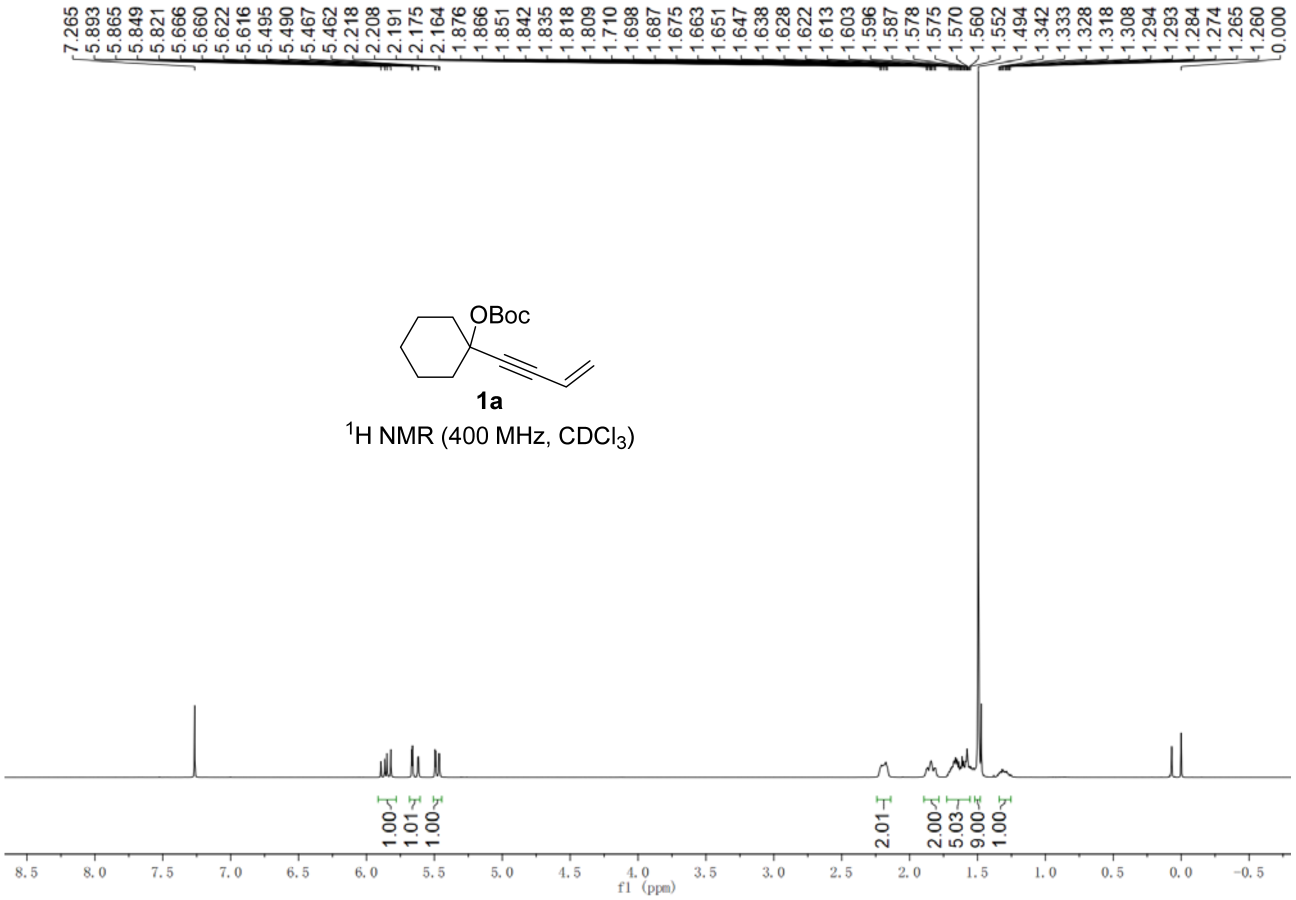

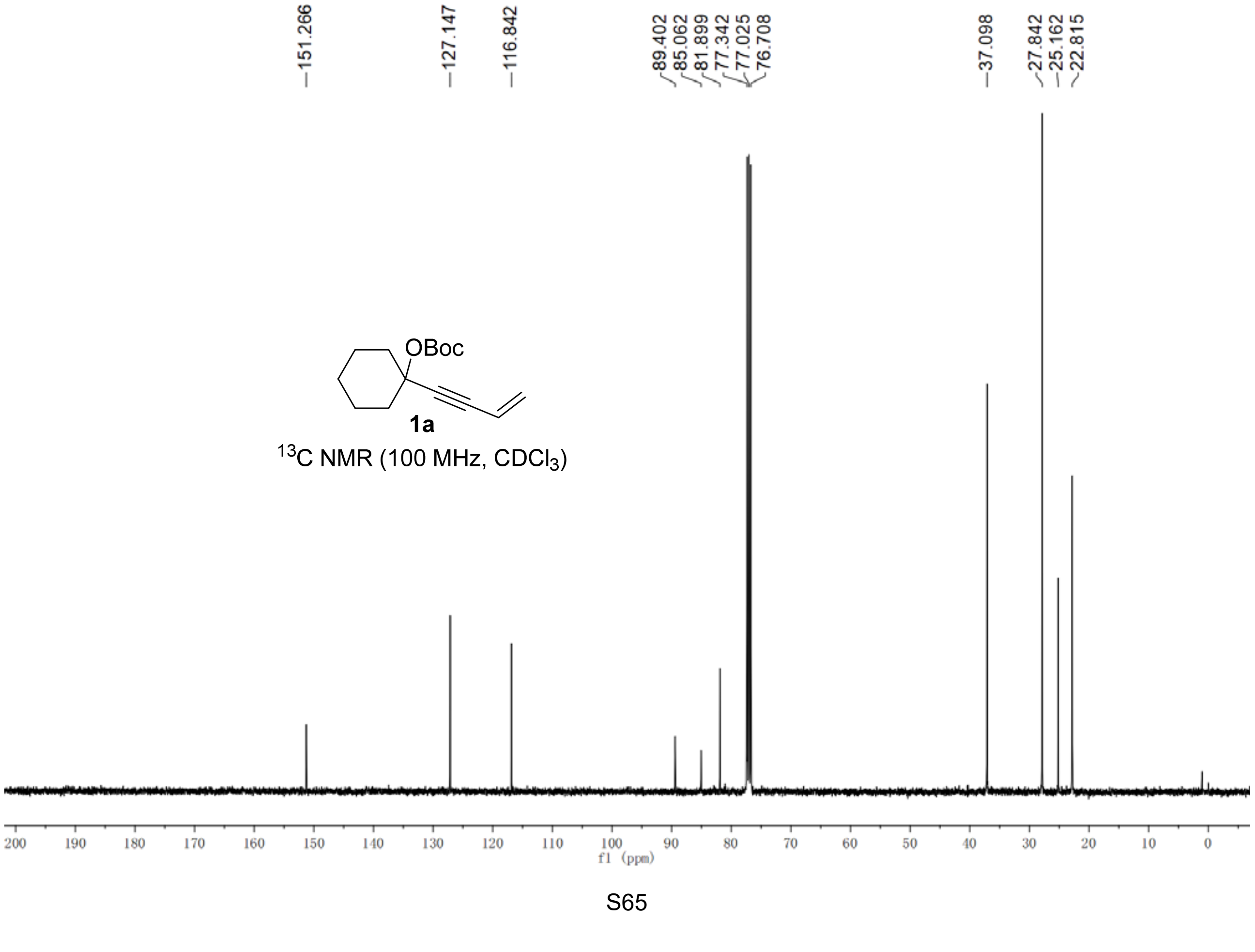

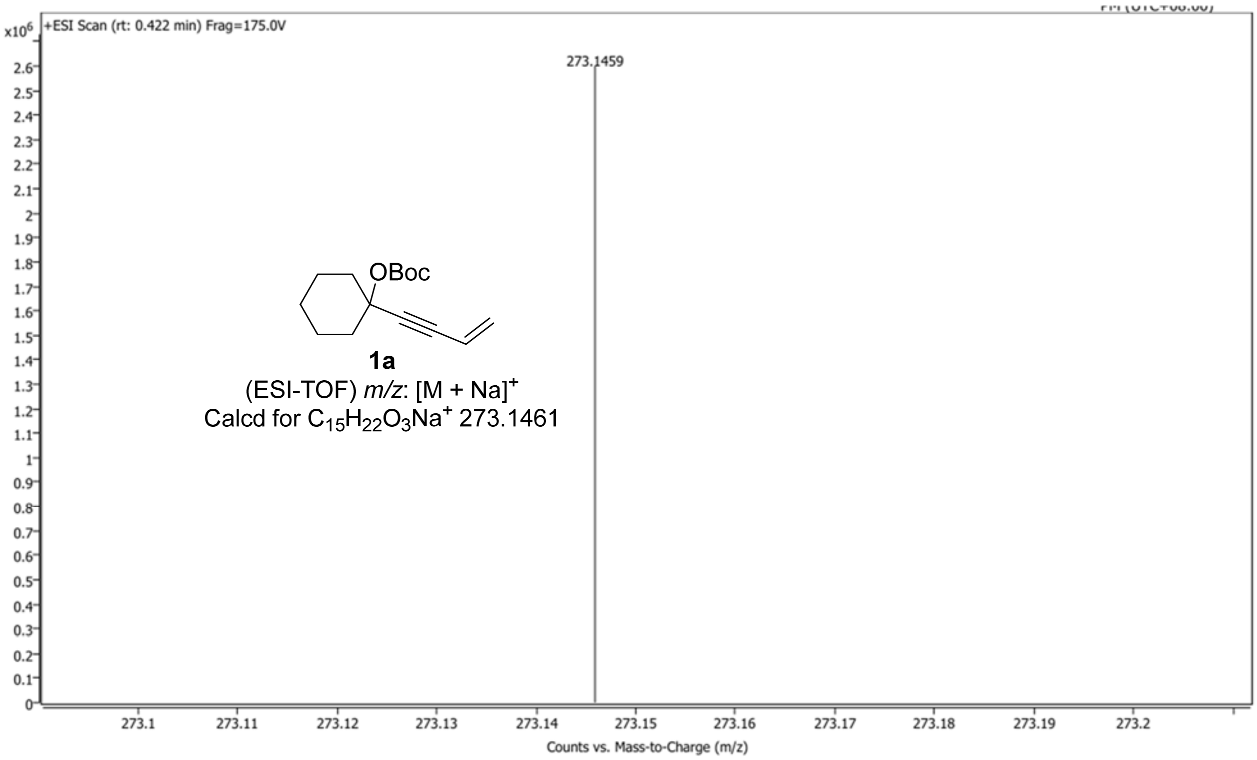
+ESI Scan (rt: 0.422 min) Frag=175.0V
x10^6
273.1459
OBoc
1a
(ESI-TOF) m/z: [M + Na]+
Calcd for C15H22O3Na+ 273.1461
Counts vs. Mass-to-Charge (m/z)

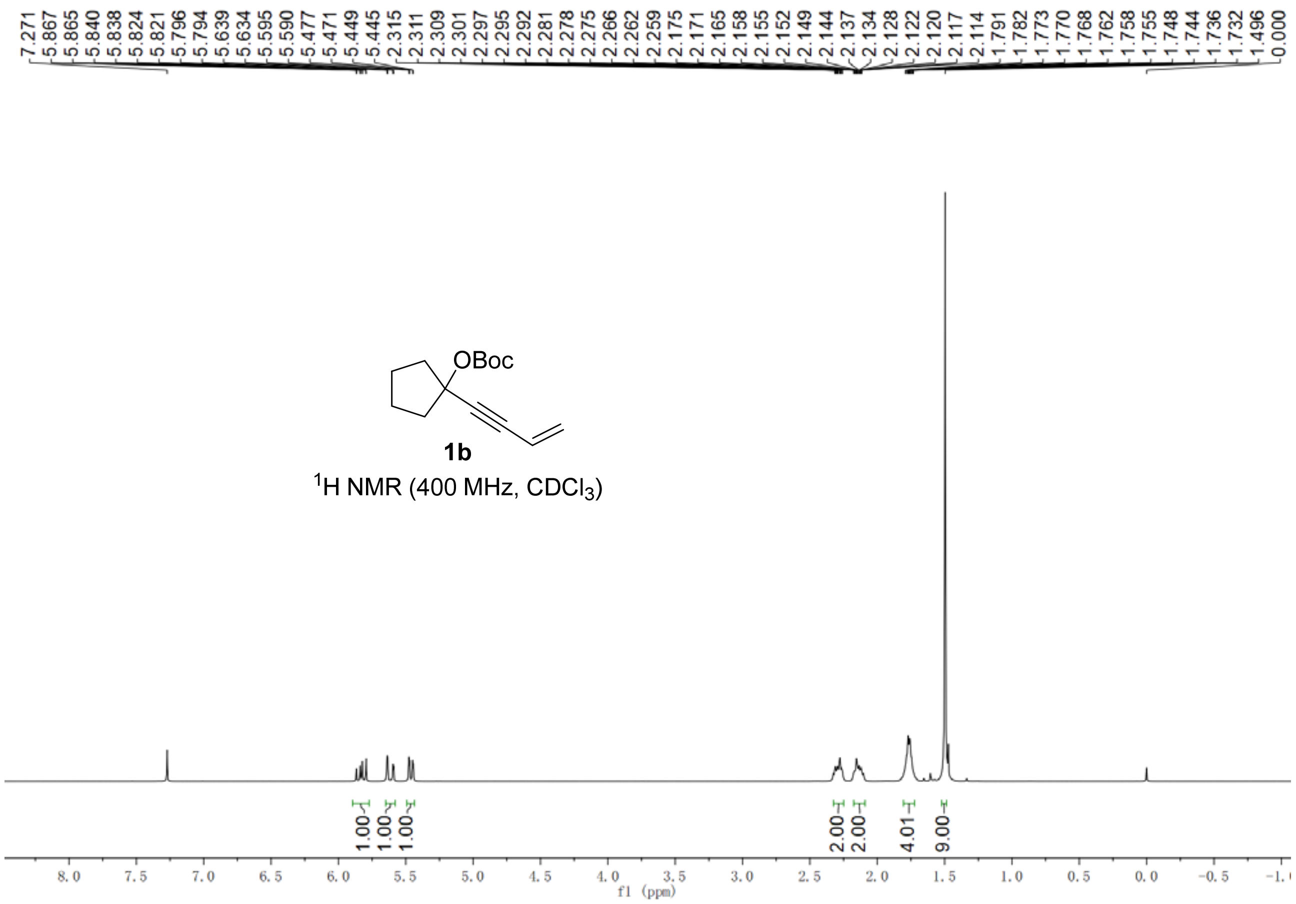
7.271
5.867
5.865
5.840
5.838
5.824
5.821
5.796
5.794
5.639
5.634
5.595
5.590
5.477
5.471
5.449
5.445
2.315
2.311
2.309
2.301
2.297
2.295
2.292
2.281
2.278
2.275
2.266
2.262
2.259
2.175
2.171
2.165
2.158
2.155
2.152
2.149
2.144
2.137
2.134
2.128
2.122
2.120
2.117
2.114
1.791
1.782
1.773
1.770
1.768
1.762
1.758
1.755
1.748
1.744
1.736
1.732
1.496
0.000
OBoc
1b
^{1}H NMR (400 MHz, CDCl$_3$)
1.00
1.00
1.00
2.00
2.00
4.01
9.00
8.0 7.5 7.0 6.5 6.0 5.5 5.0 4.5 4.0 3.5 3.0 2.5 2.0 1.5 1.0 0.5 0.0 -0.5
f1 (ppm)

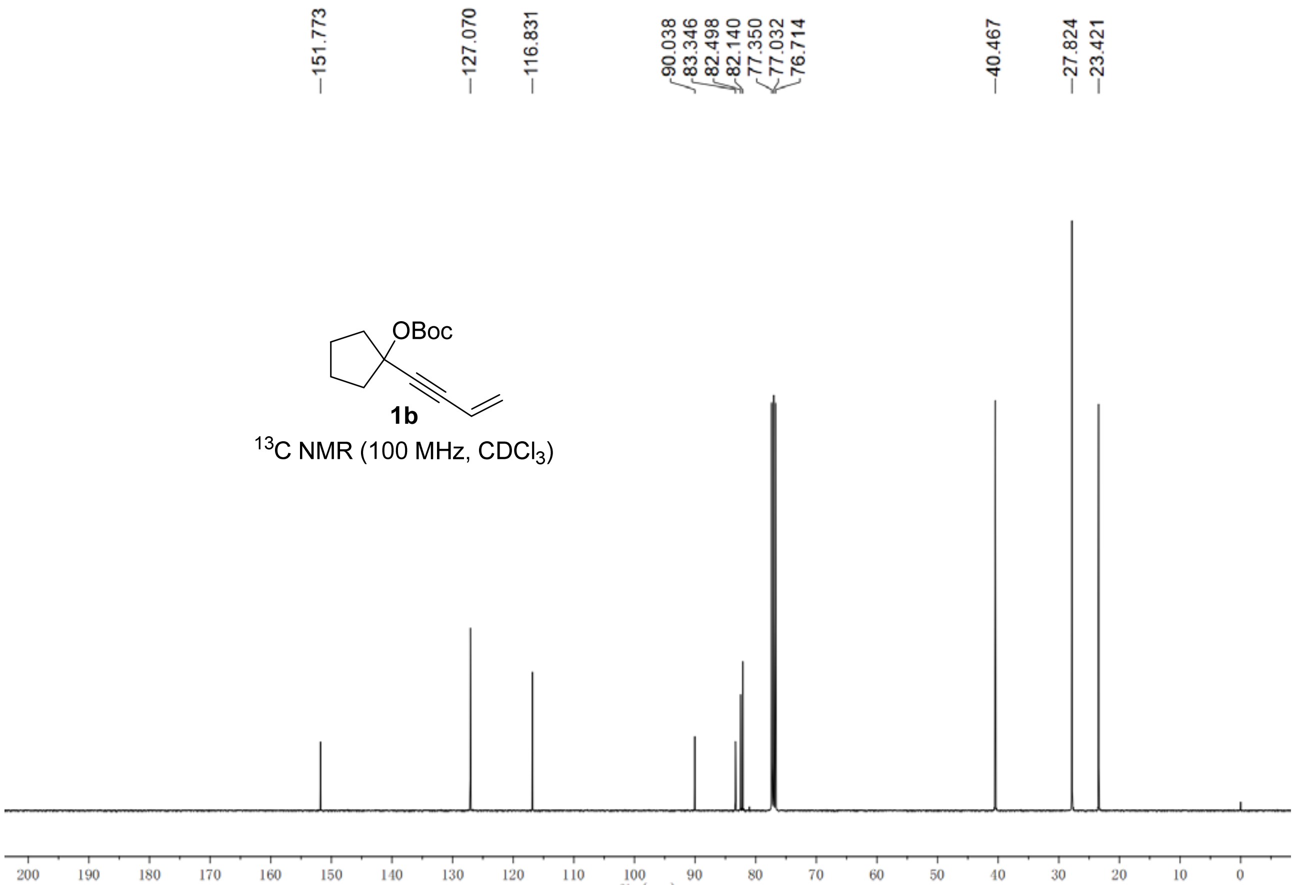
151.773
127.070
116.831
90.038
83.346
82.498
82.140
77.350
77.032
76.714
40.467
27.824
23.421
OBoc
1b
^{13}C NMR (100 MHz, CDCl$_3$)
200 190 180 170 160 150 140 130 120 110 100 90 80 70 60 50 40 30 20 10 0
f1 (ppm)

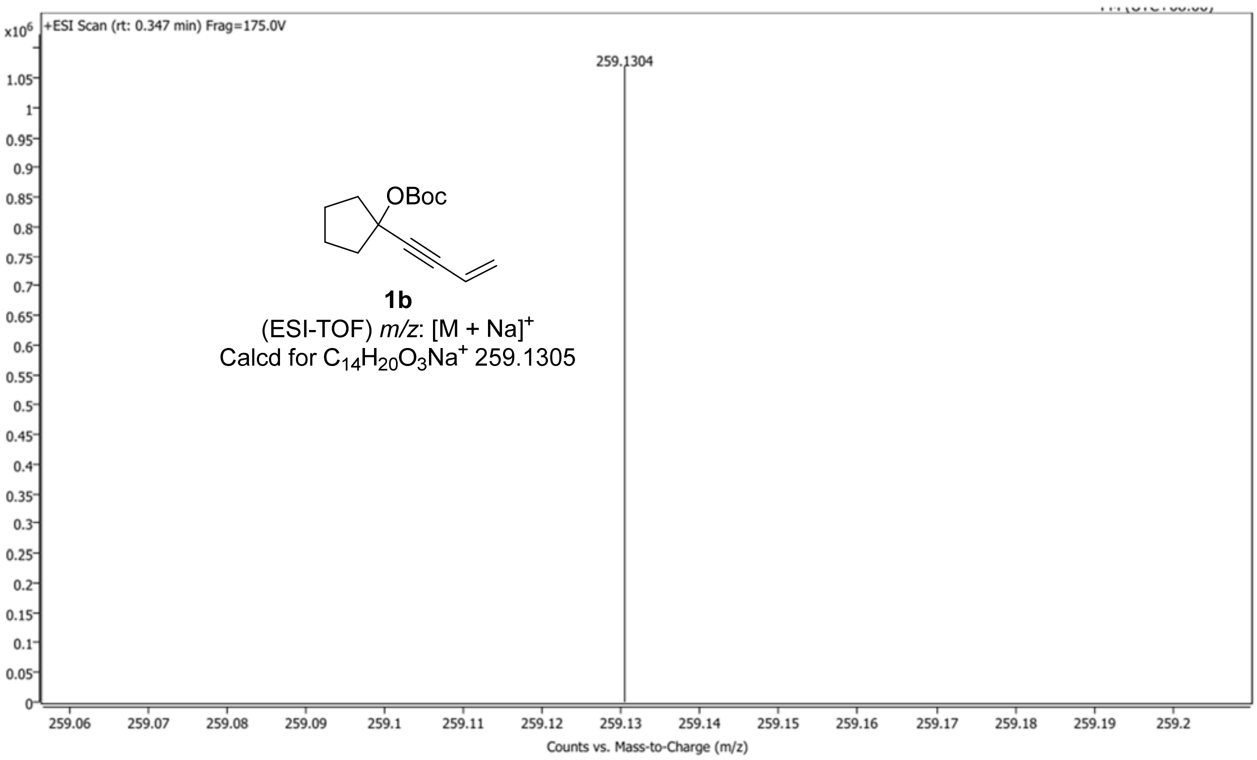
x10^6
+ESI Scan (rt: 0.347 min) Frag=175.0V
259.1304
OBoc
1b
(ESI-TOF) m/z: [M + Na]+
Calcd for C14H20O3Na+ 259.1305
Counts vs. Mass-to-Charge (m/z)

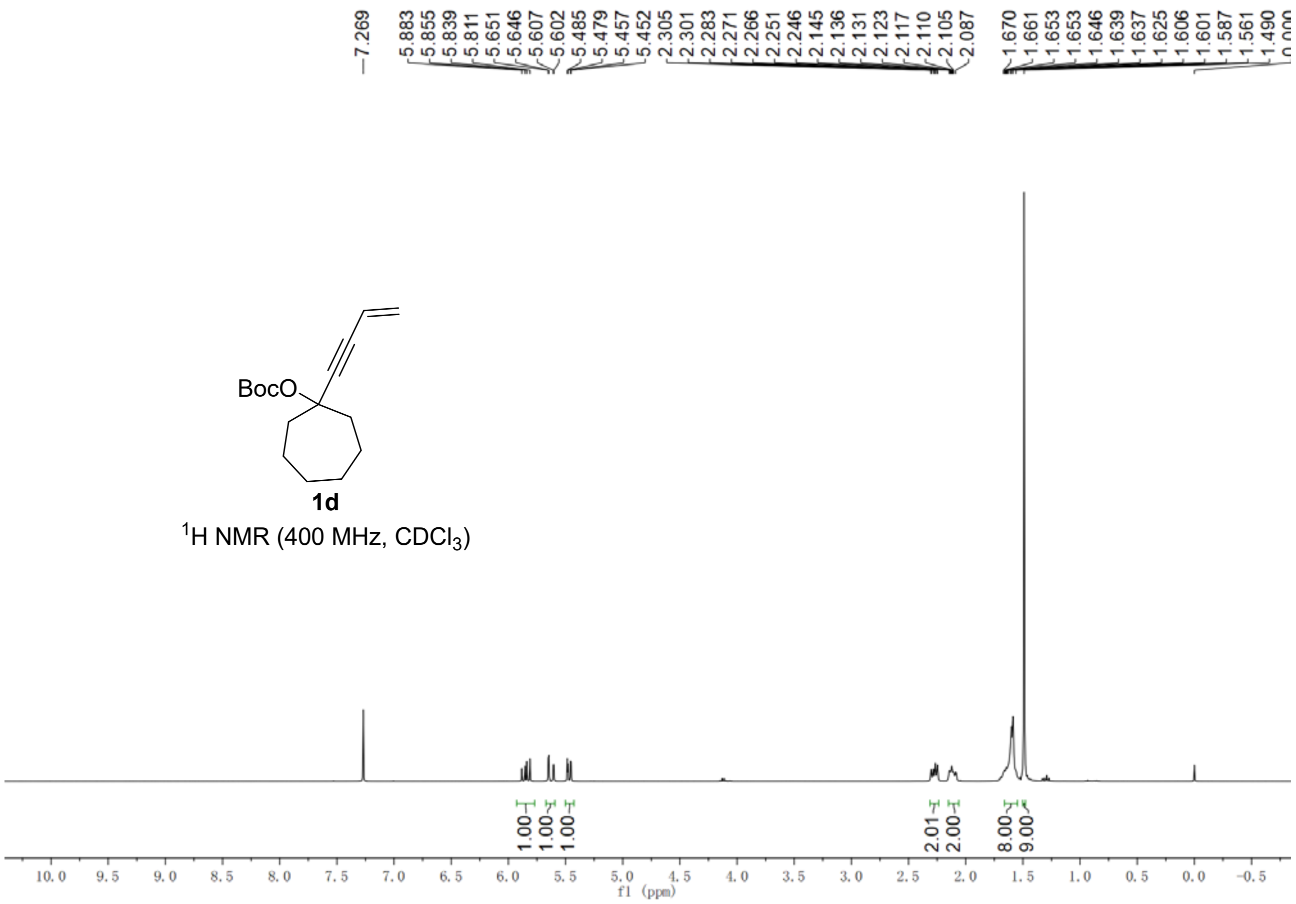

1d

^{1}H NMR (400 MHz, $CDCl_3$)

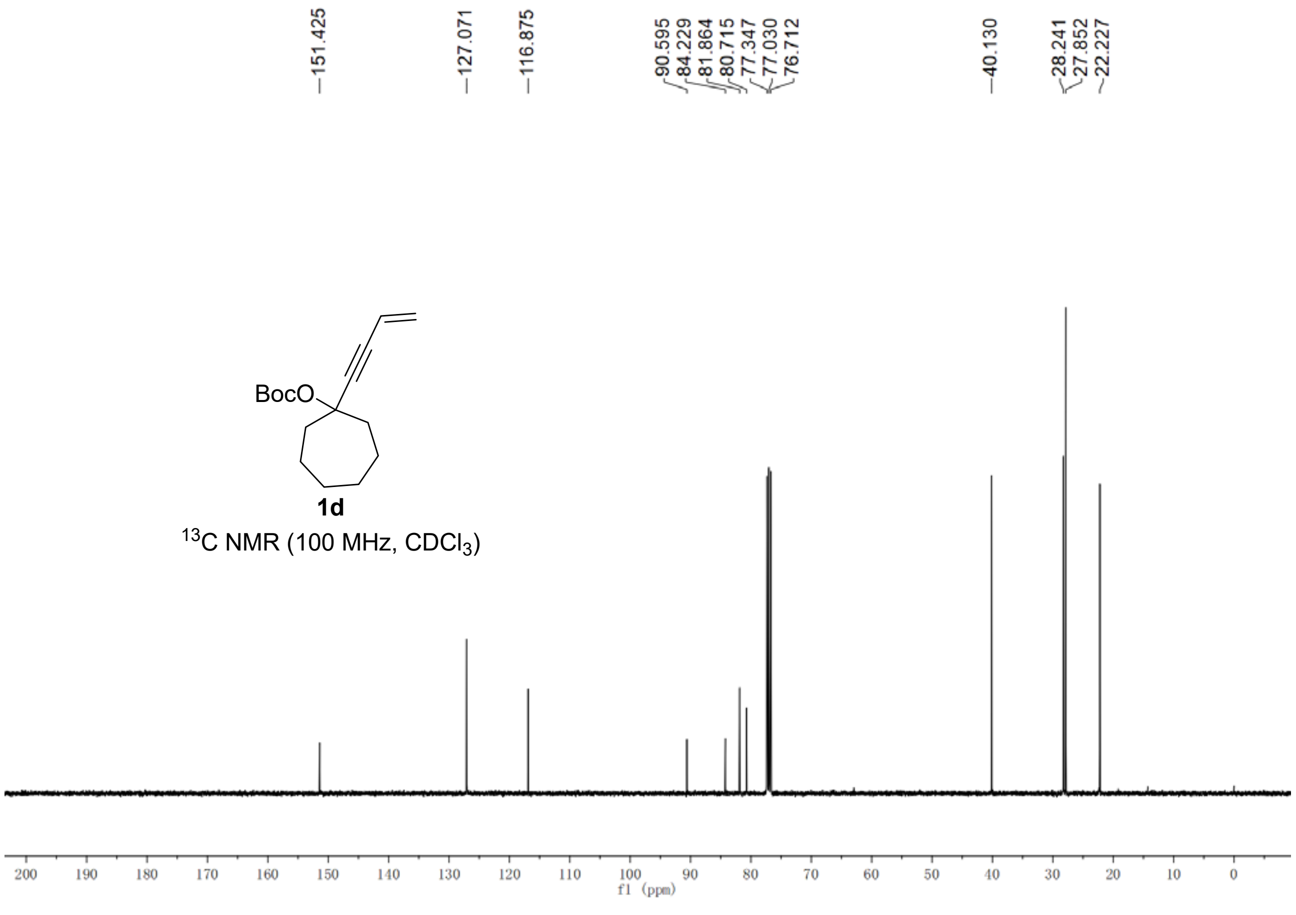

1d

^{13}C NMR (100 MHz, $CDCl_3$)

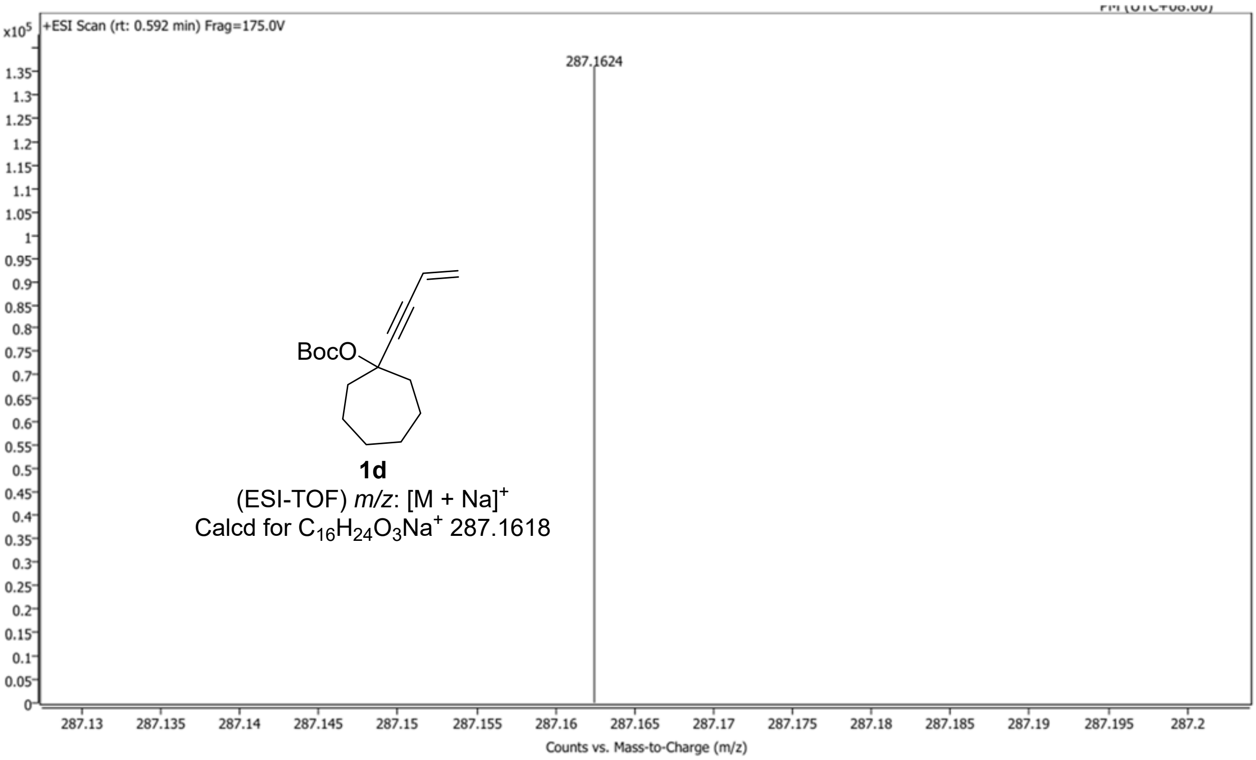

1d
(ESI-TOF) *m/z*: $[M + Na]^+$
Calcd for $C_{16}H_{24}O_3Na^+$ 287.1618

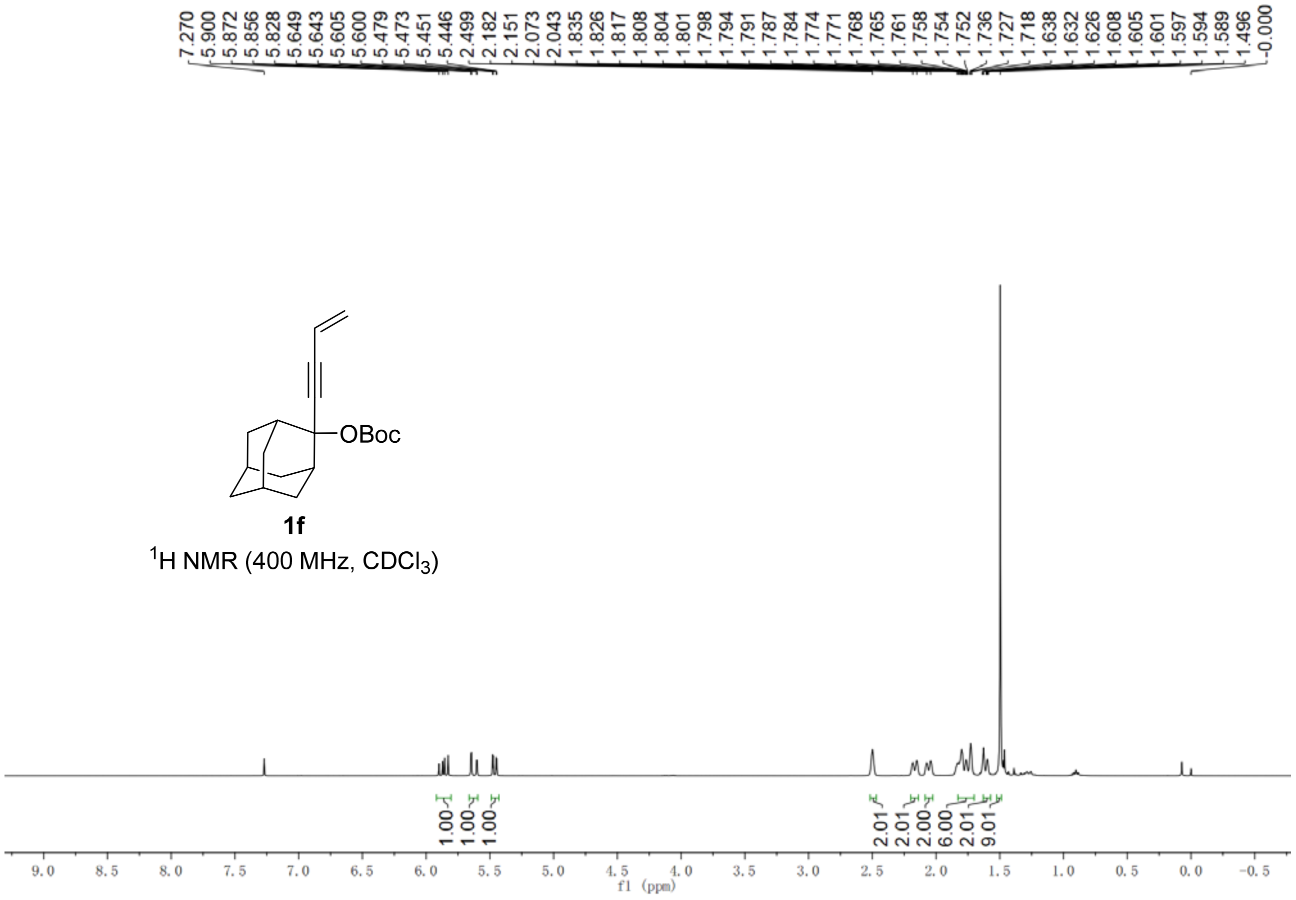
7.270
5.900
5.872
5.856
5.828
5.649
5.643
5.605
5.600
5.479
5.473
5.451
5.446
2.499
2.182
2.151
2.073
2.043
1.835
1.826
1.817
1.808
1.804
1.801
1.798
1.794
1.791
1.787
1.784
1.774
1.771
1.768
1.765
1.761
1.758
1.754
1.752
1.736
1.727
1.718
1.638
1.632
1.626
1.608
1.605
1.601
1.597
1.594
1.589
1.496
-0.000
OBoc
1f
^{1}H NMR (400 MHz, CDCl$_3$)
1.00
1.00
1.00
2.01
2.01
2.00
6.00
2.01
9.01
f1 (ppm)

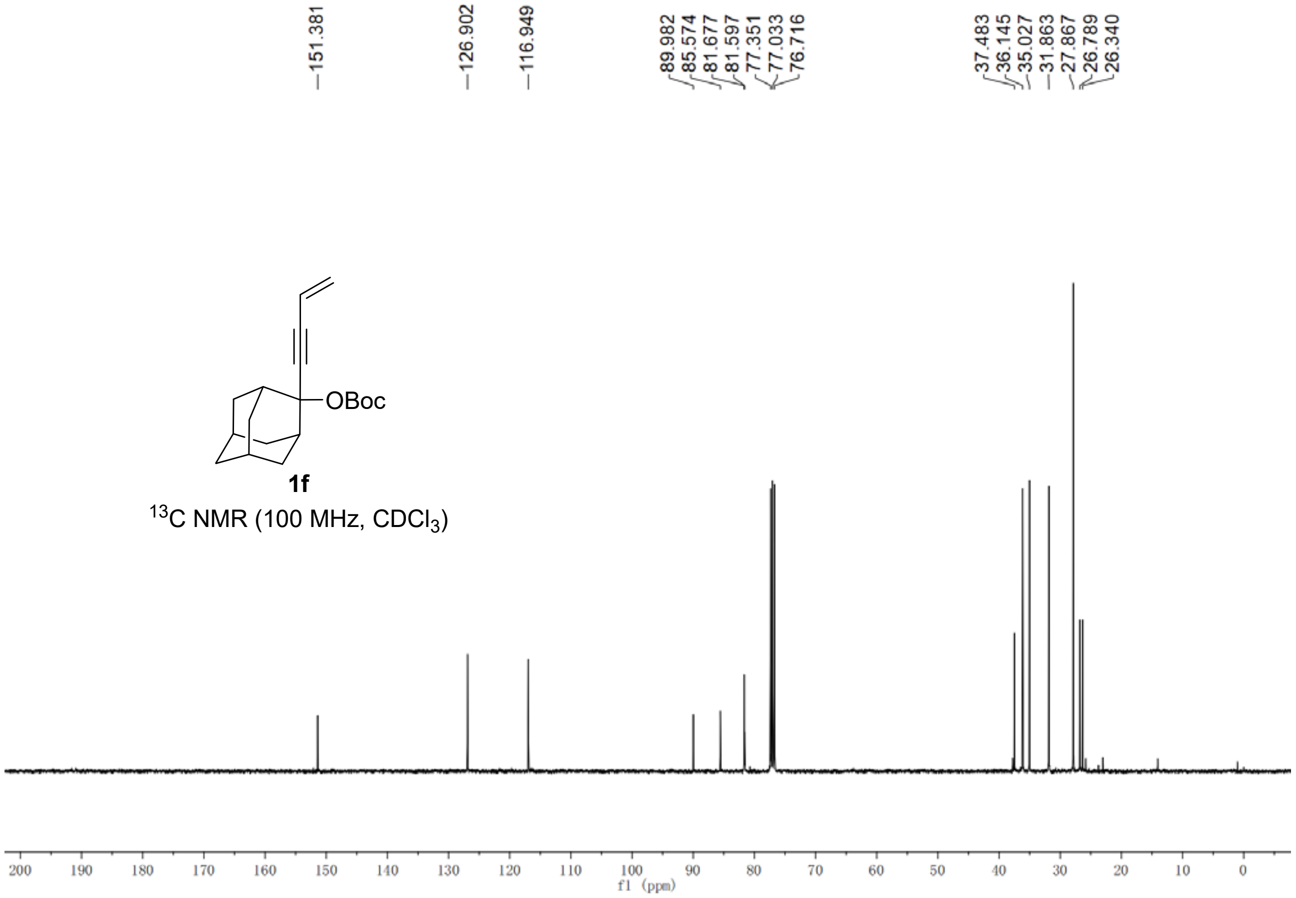
151.381
126.902
116.949
89.982
85.574
81.677
81.597
77.351
77.033
76.716
37.483
36.145
35.027
31.863
27.867
26.789
26.340
OBoc
1f
^{13}C NMR (100 MHz, CDCl$_3$)
f1 (ppm)

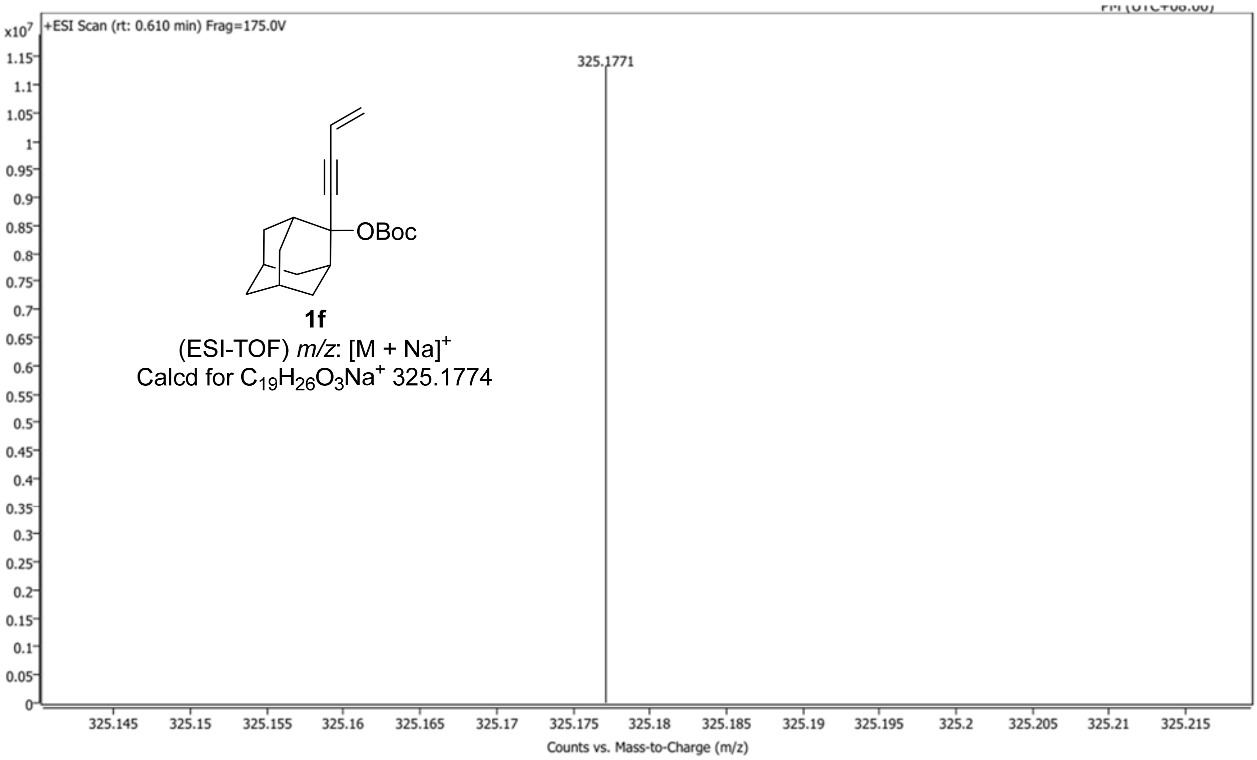
+ESI Scan (rt: 0.610 min) Frag=175.0V
x10^7
325.1771
OBoc
1f
(ESI-TOF) m/z: [M + Na]+
Calcd for C19H26O3Na+ 325.1774
Counts vs. Mass-to-Charge (m/z)

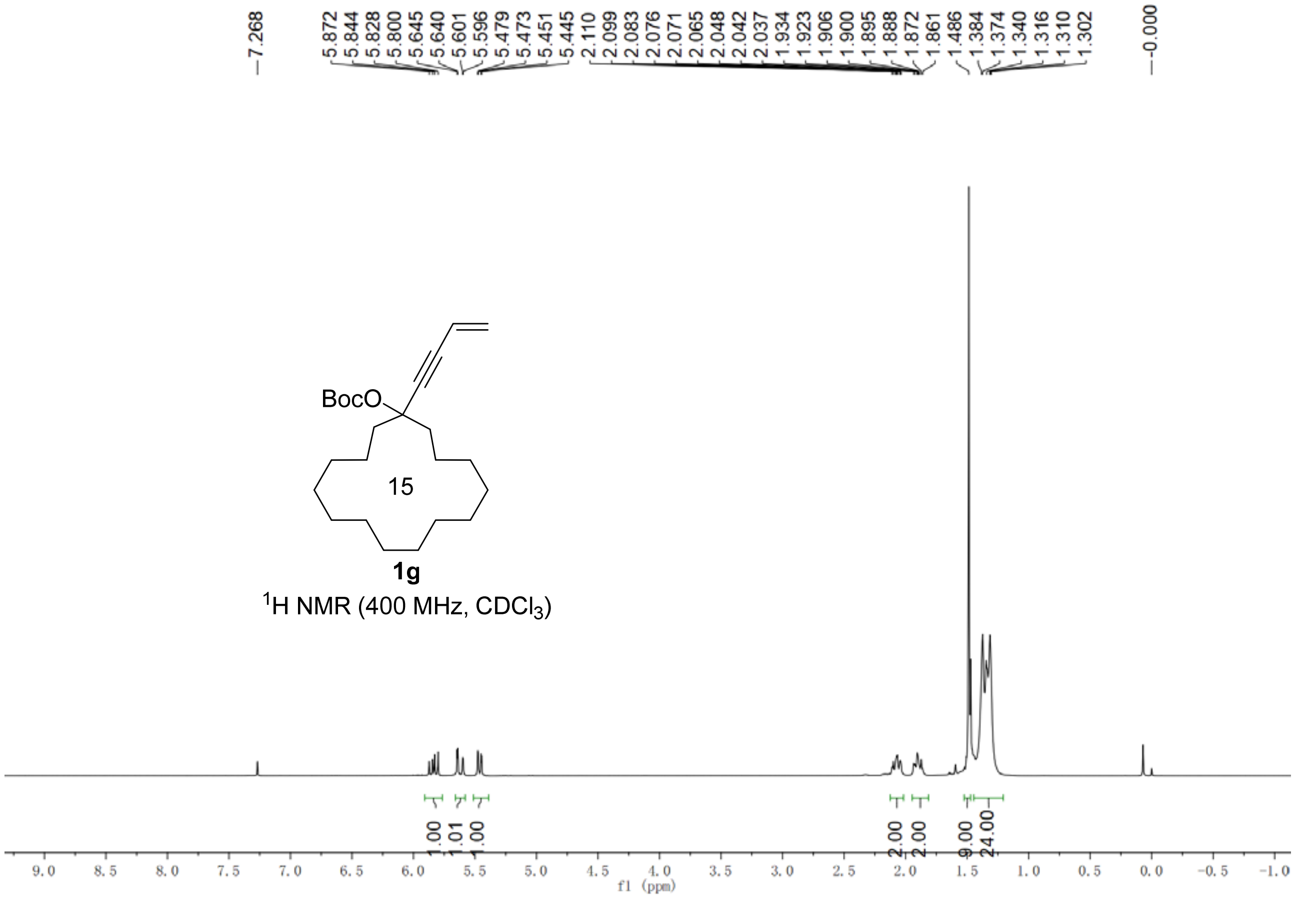
7.268
5.872 5.844 5.828 5.800 5.645 5.640 5.601 5.596 5.479 5.473 5.451 5.445 2.110 2.099 2.083 2.076 2.071 2.065 2.048 2.042 2.037 1.934 1.923 1.906 1.900 1.895 1.888 1.872 1.861 1.486 1.384 1.374 1.340 1.316 1.310 1.302
0.000
BocO
15
1g
1H NMR (400 MHz, CDCl3)
1.00 1.01 1.00 2.00 2.00 9.00 24.00
f1 (ppm)

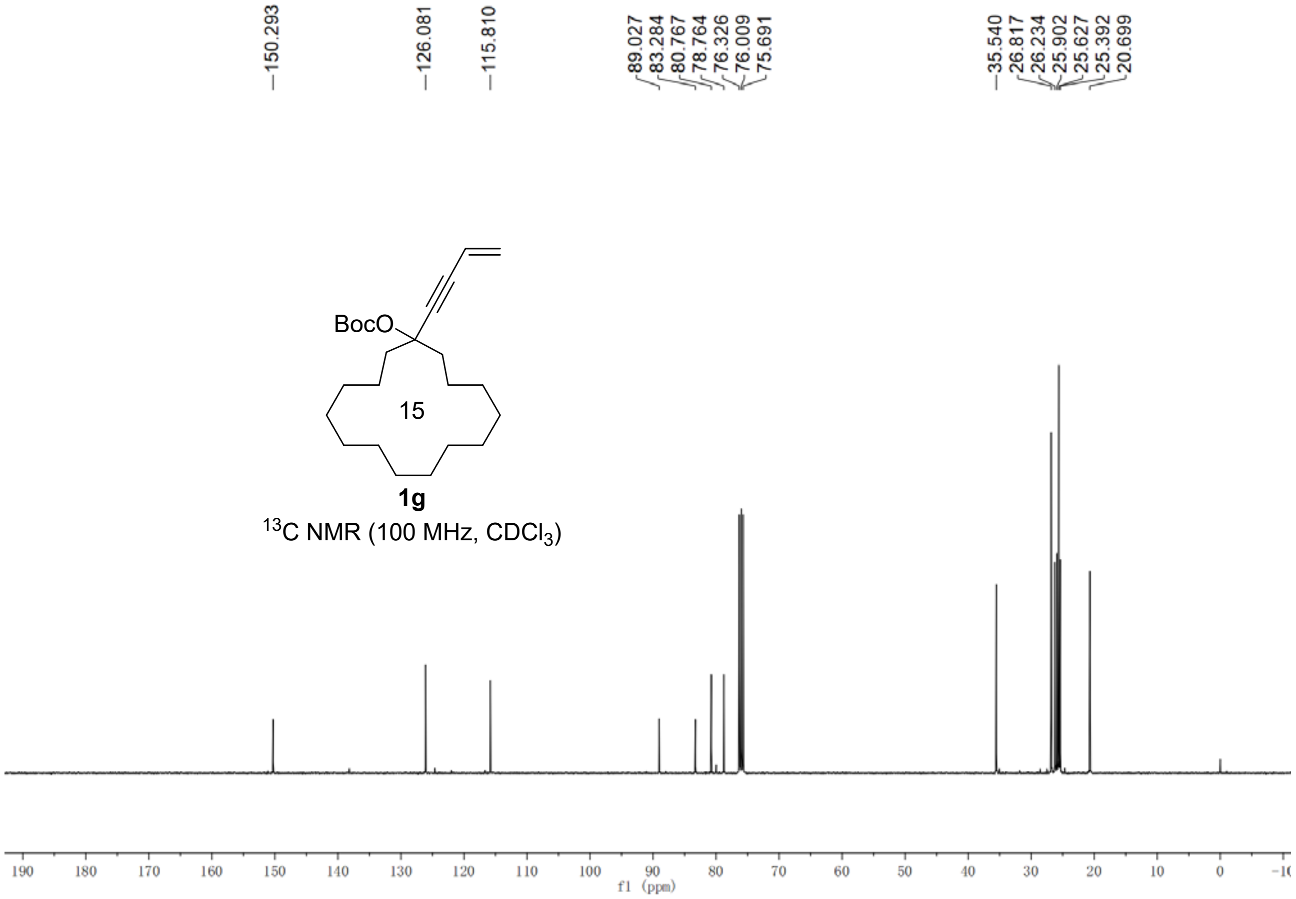
150.293
126.081
115.810
89.027 83.284 80.767 78.764 76.326 76.009 75.691
35.540 26.817 26.234 25.902 25.627 25.392 20.699
BocO
15
1g
13C NMR (100 MHz, CDCl3)
f1 (ppm)

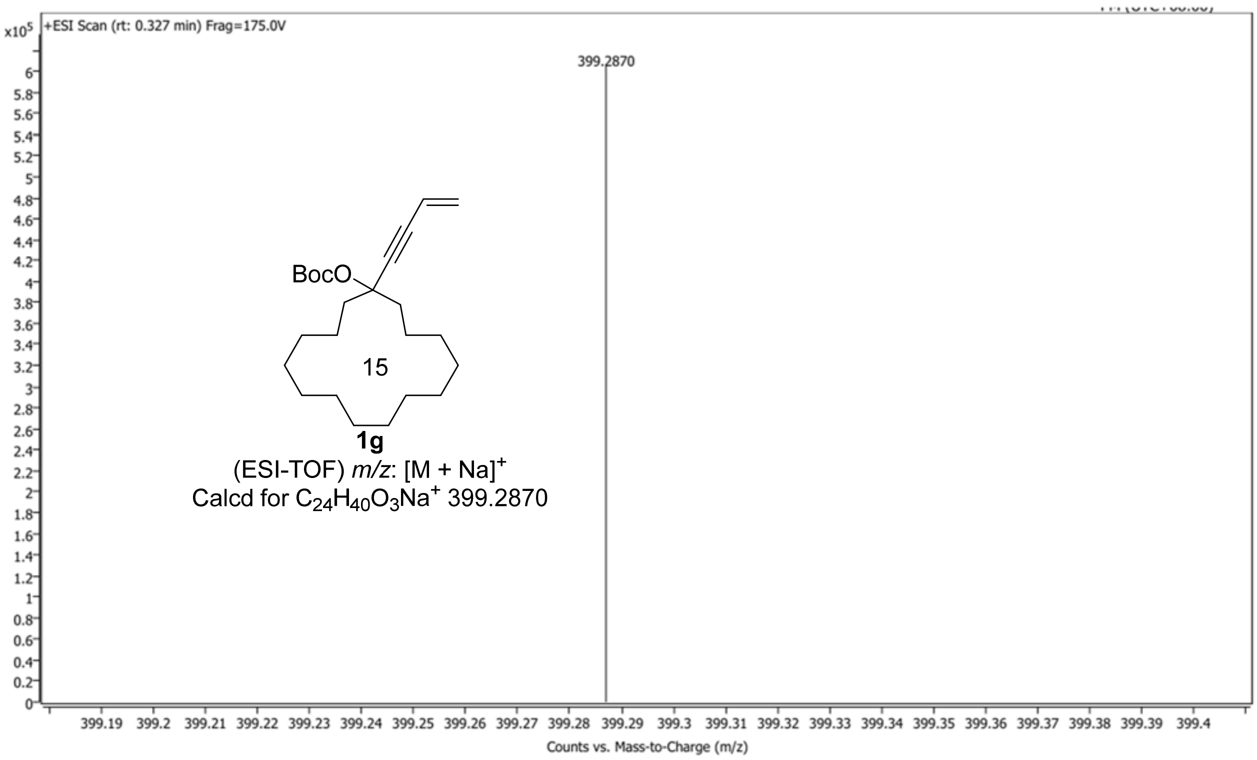

+ESI Scan (rt: 0.327 min) Frag=175.0V
x10^5
399.2870
BocO
15
1g
(ESI-TOF) m/z: [M + Na]+
Calcd for C24H40O3Na+ 399.2870
Counts vs. Mass-to-Charge (m/z)

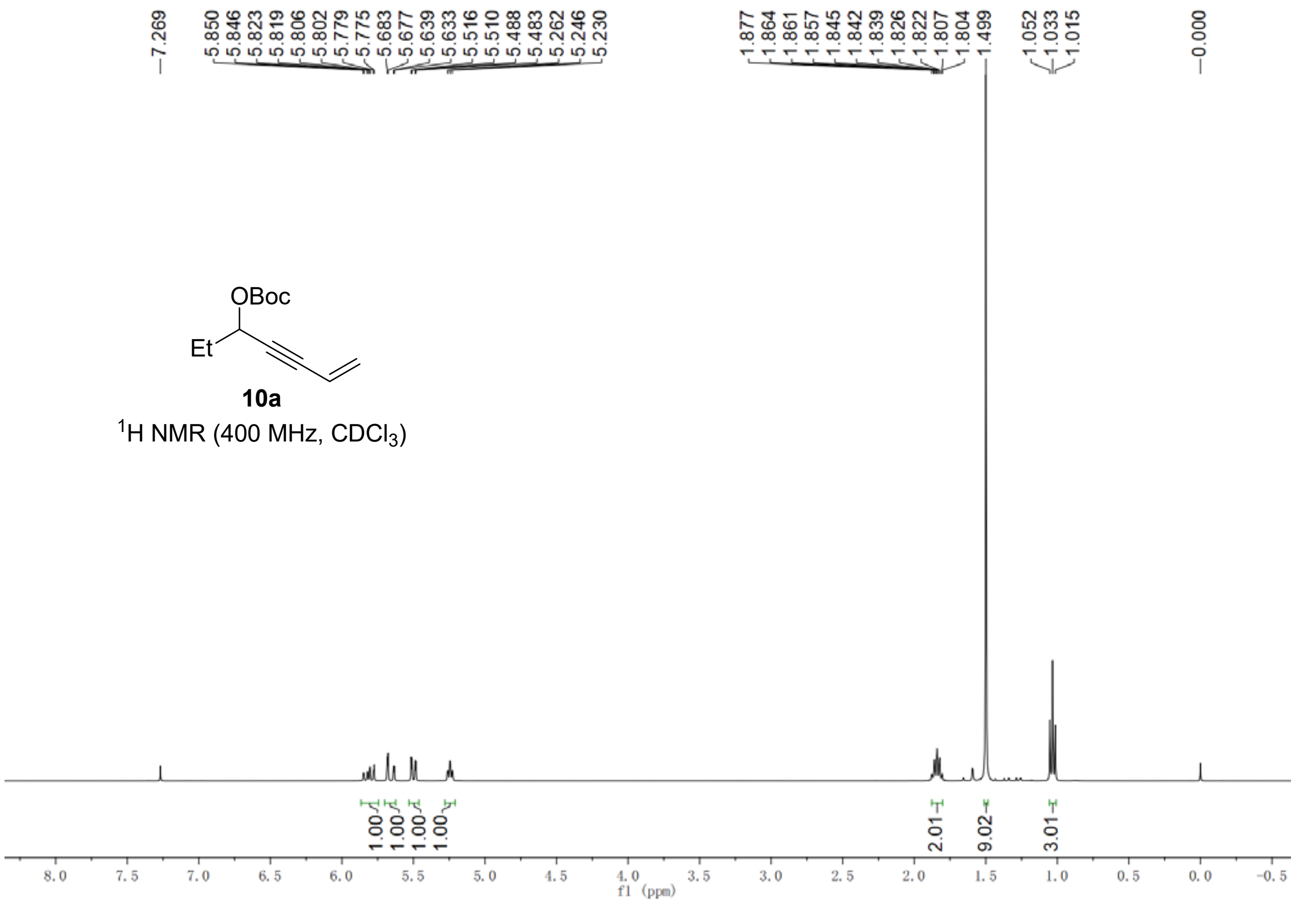

10a

^{1}H NMR (400 MHz, $CDCl_3$)

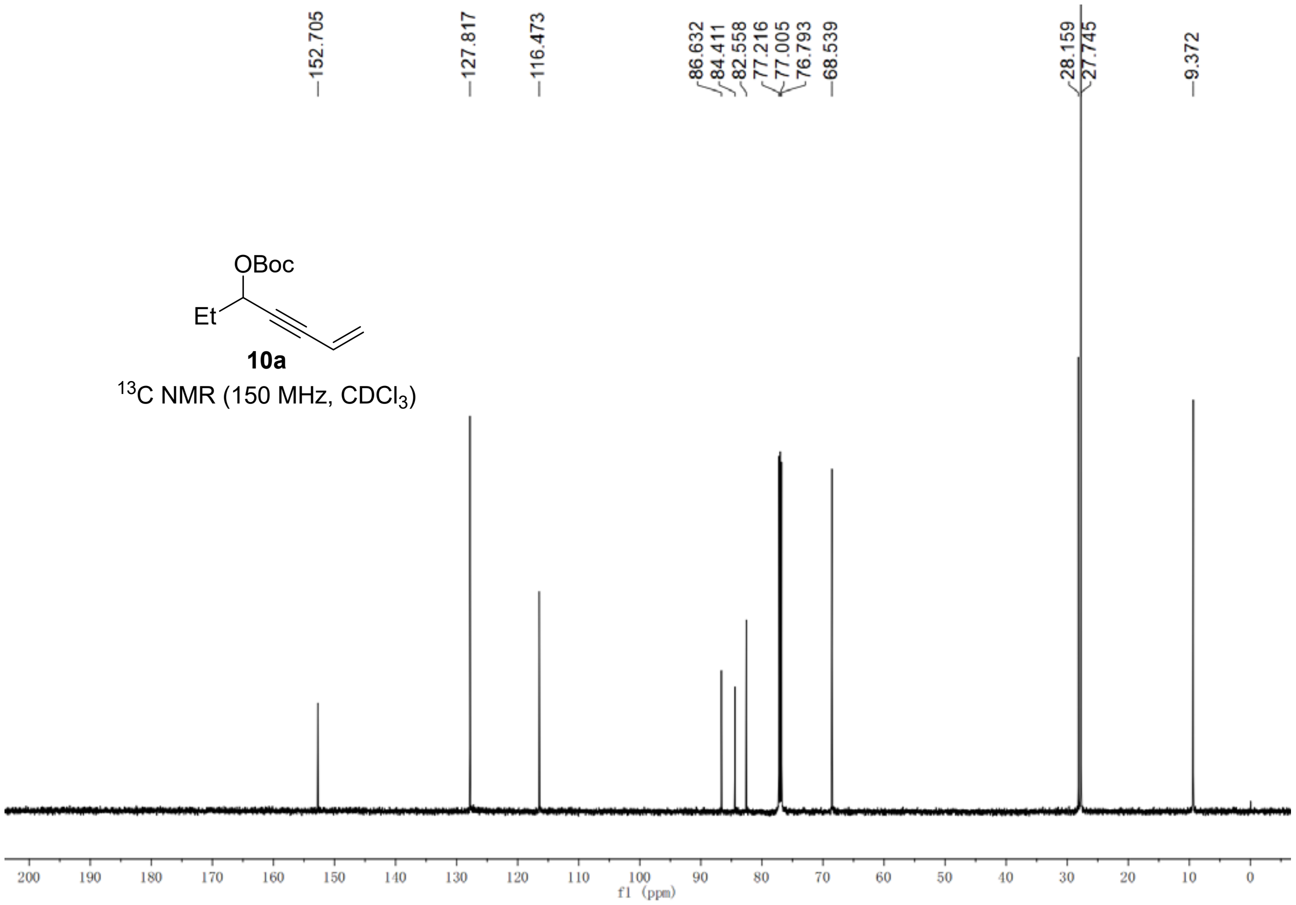

10a

^{13}C NMR (150 MHz, $CDCl_3$)

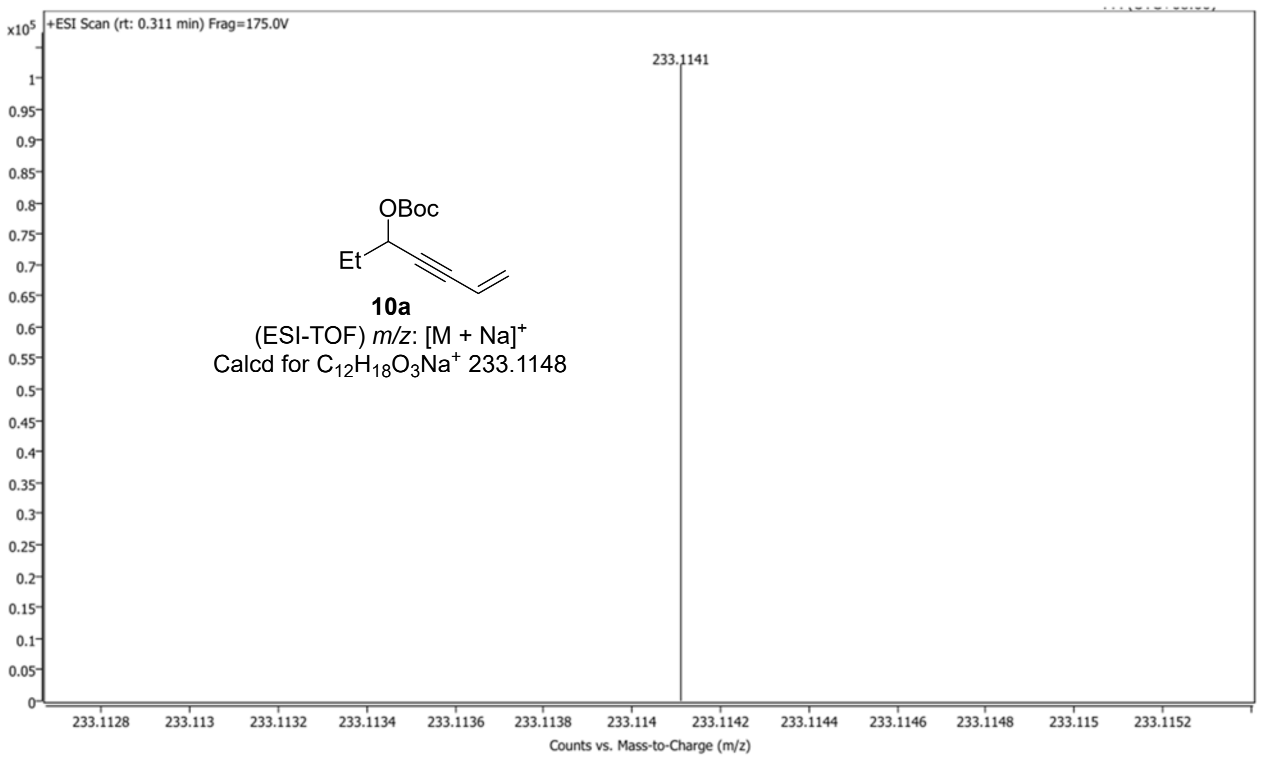

+ESI Scan (rt: 0.311 min) Frag=175.0V
x10^5
233.1141
OBoc
Et
10a
(ESI-TOF) m/z: [M + Na]+
Calcd for C12H18O3Na+ 233.1148
Counts vs. Mass-to-Charge (m/z)

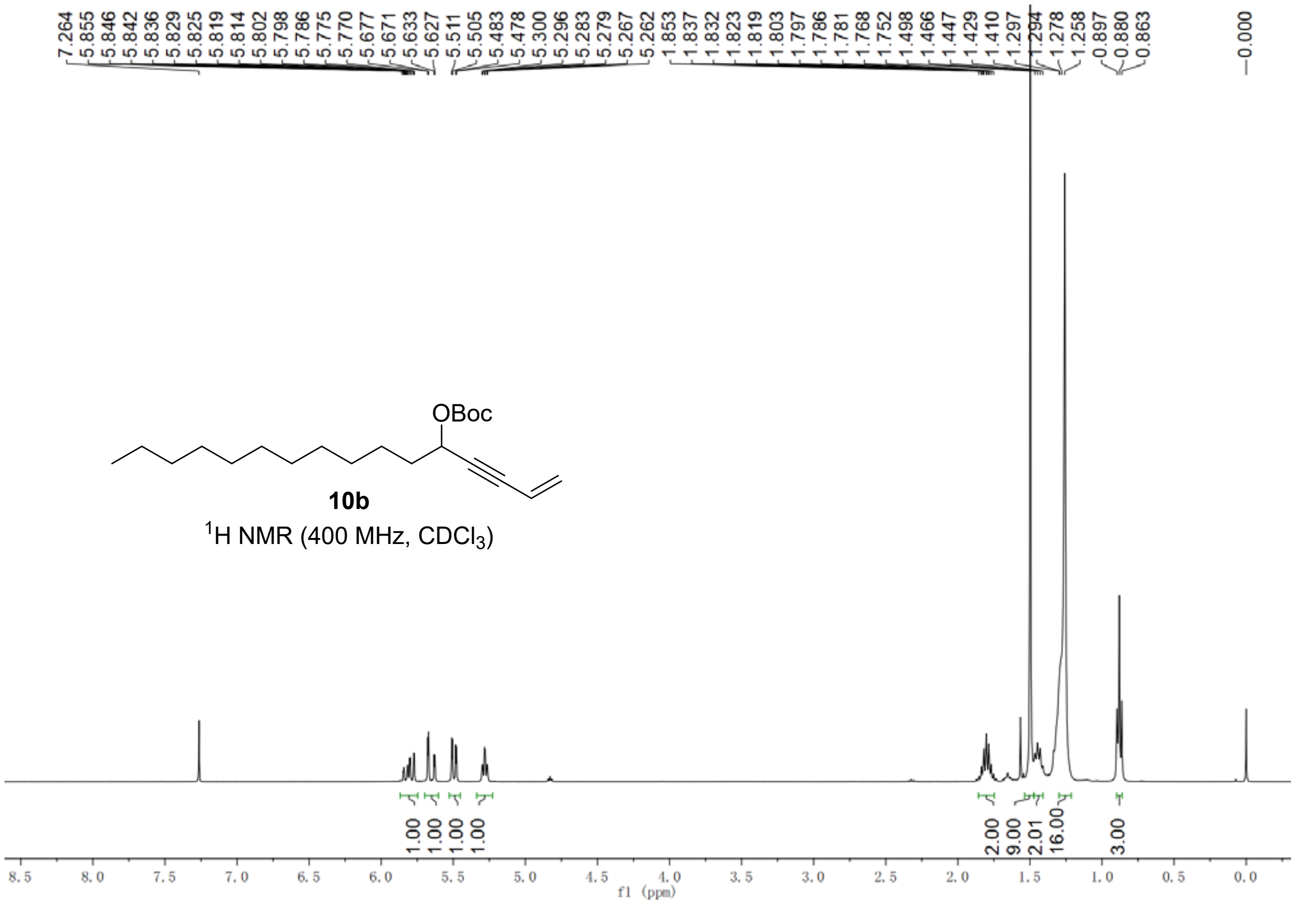

10b

^{1}H NMR (400 MHz, $CDCl_3$)

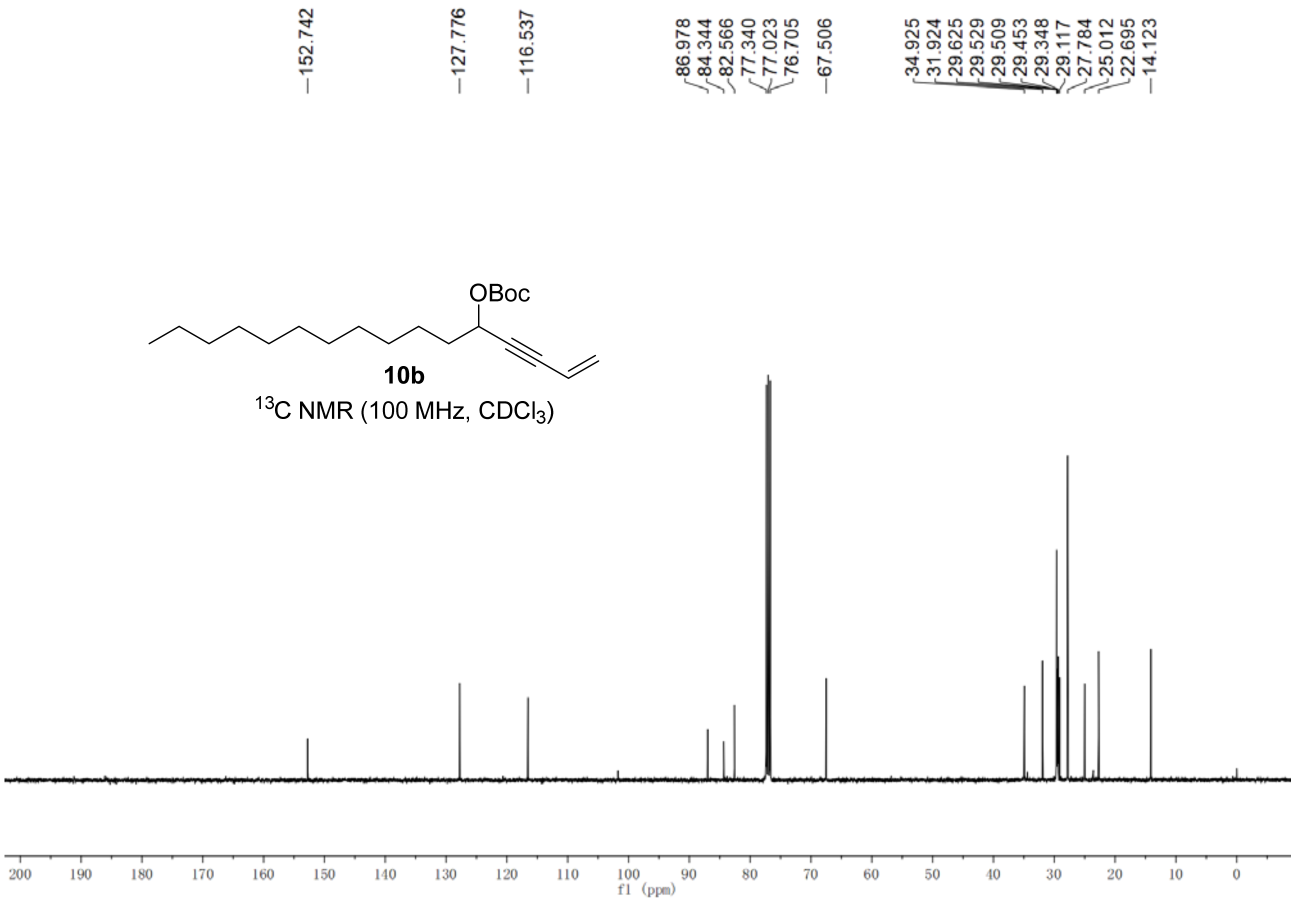

10b

^{13}C NMR (100 MHz, $CDCl_3$)

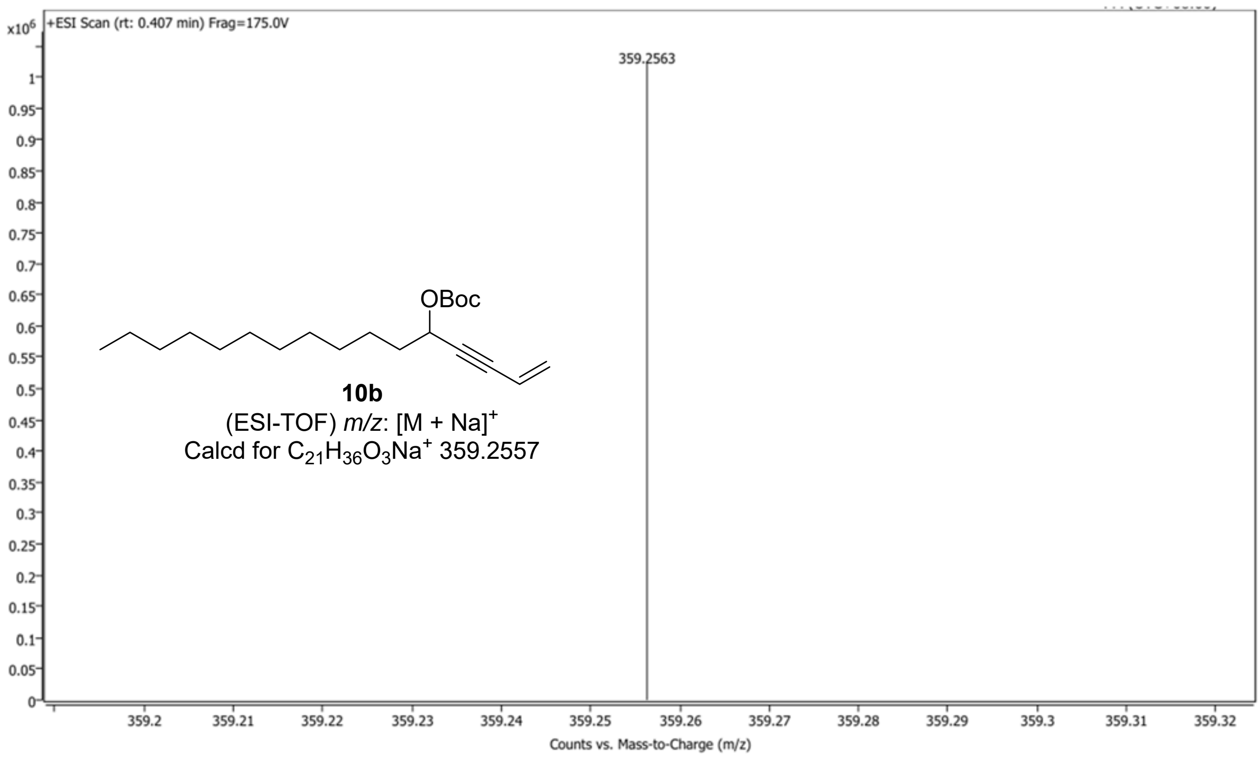
x10^6
+ESI Scan (rt: 0.407 min) Frag=175.0V
359.2563
OBoc
10b
(ESI-TOF) m/z: [M + Na]+
Calcd for C21H36O3Na+ 359.2557
Counts vs. Mass-to-Charge (m/z)

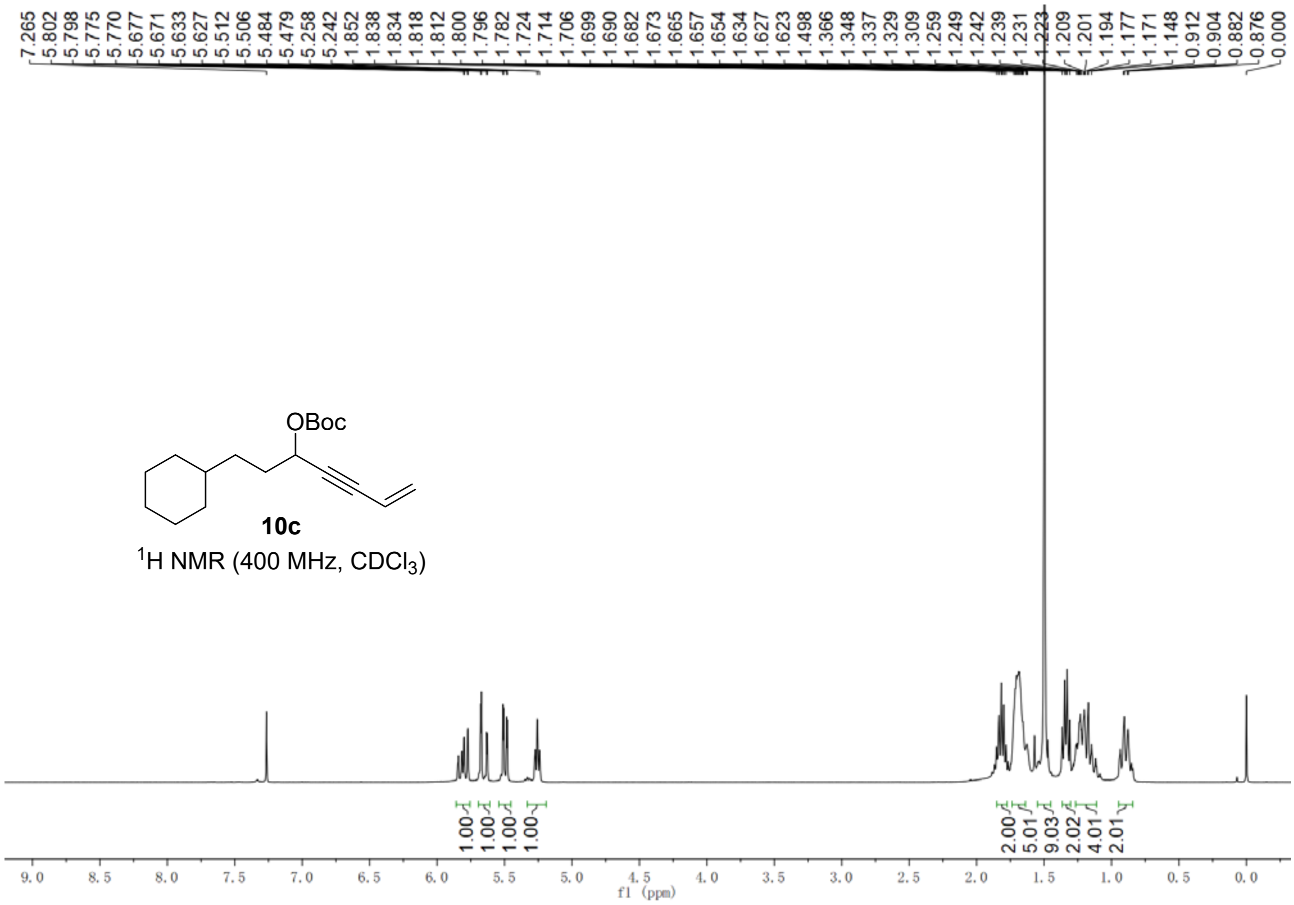

10c

^{1}H NMR (400 MHz, $CDCl_3$)

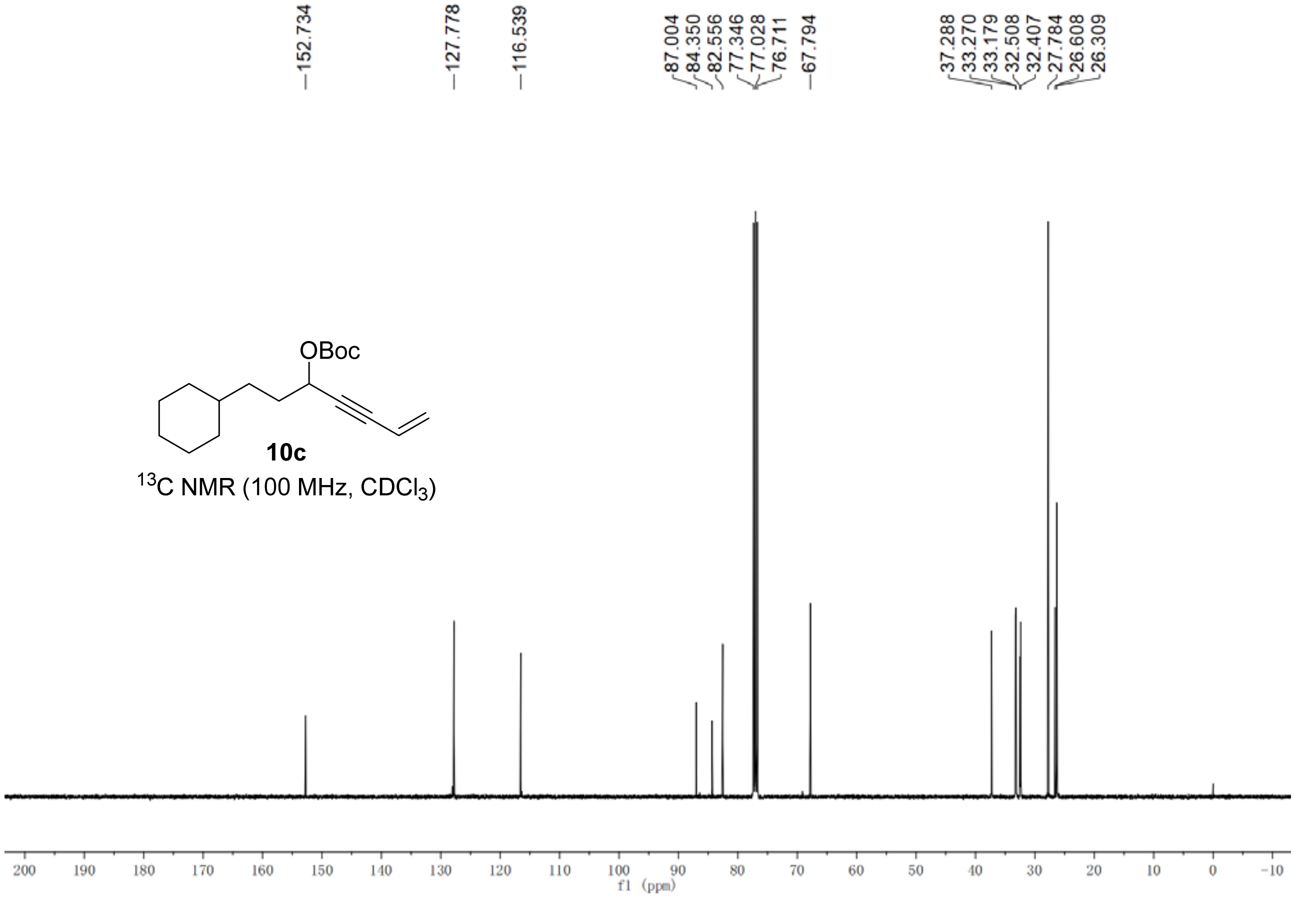

10c

^{13}C NMR (100 MHz, $CDCl_3$)

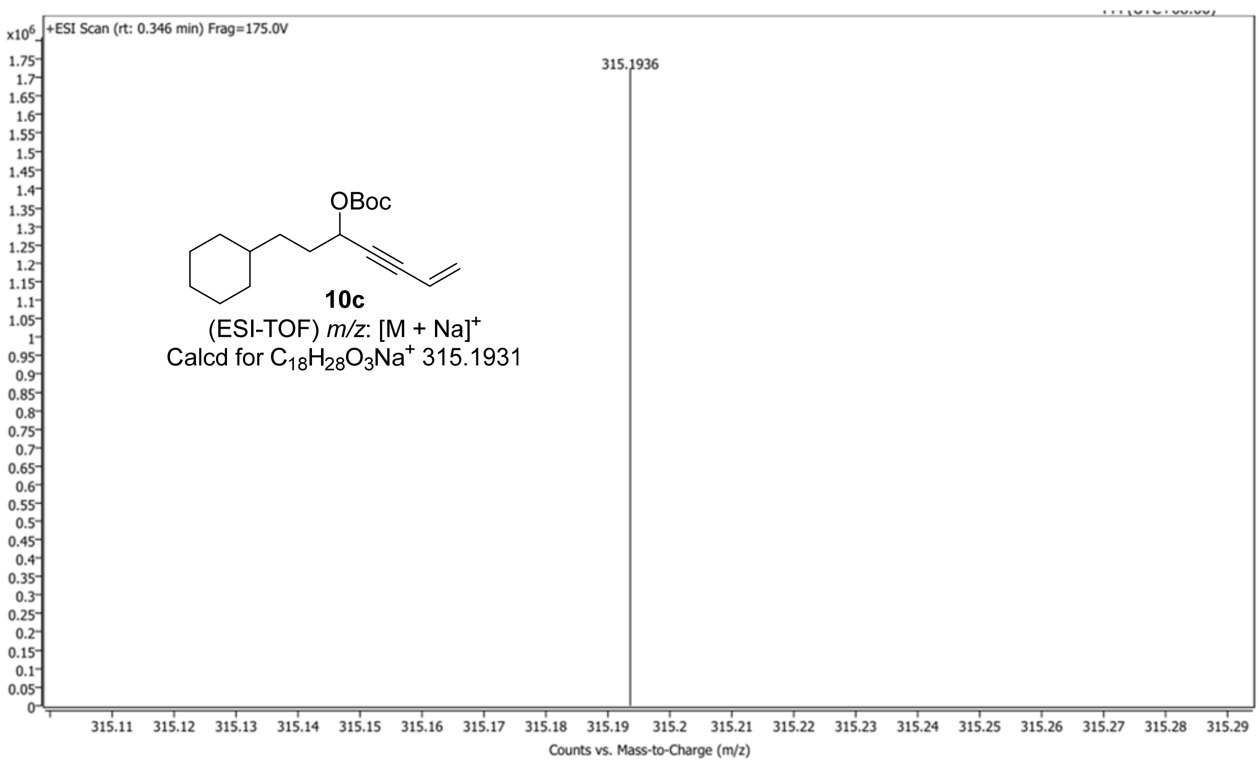
+ESI Scan (rt: 0.346 min) Frag=175.0V
x10^6
315.1936
OBoc
10c
(ESI-TOF) m/z: [M + Na]+
Calcd for C18H28O3Na+ 315.1931
Counts vs. Mass-to-Charge (m/z)

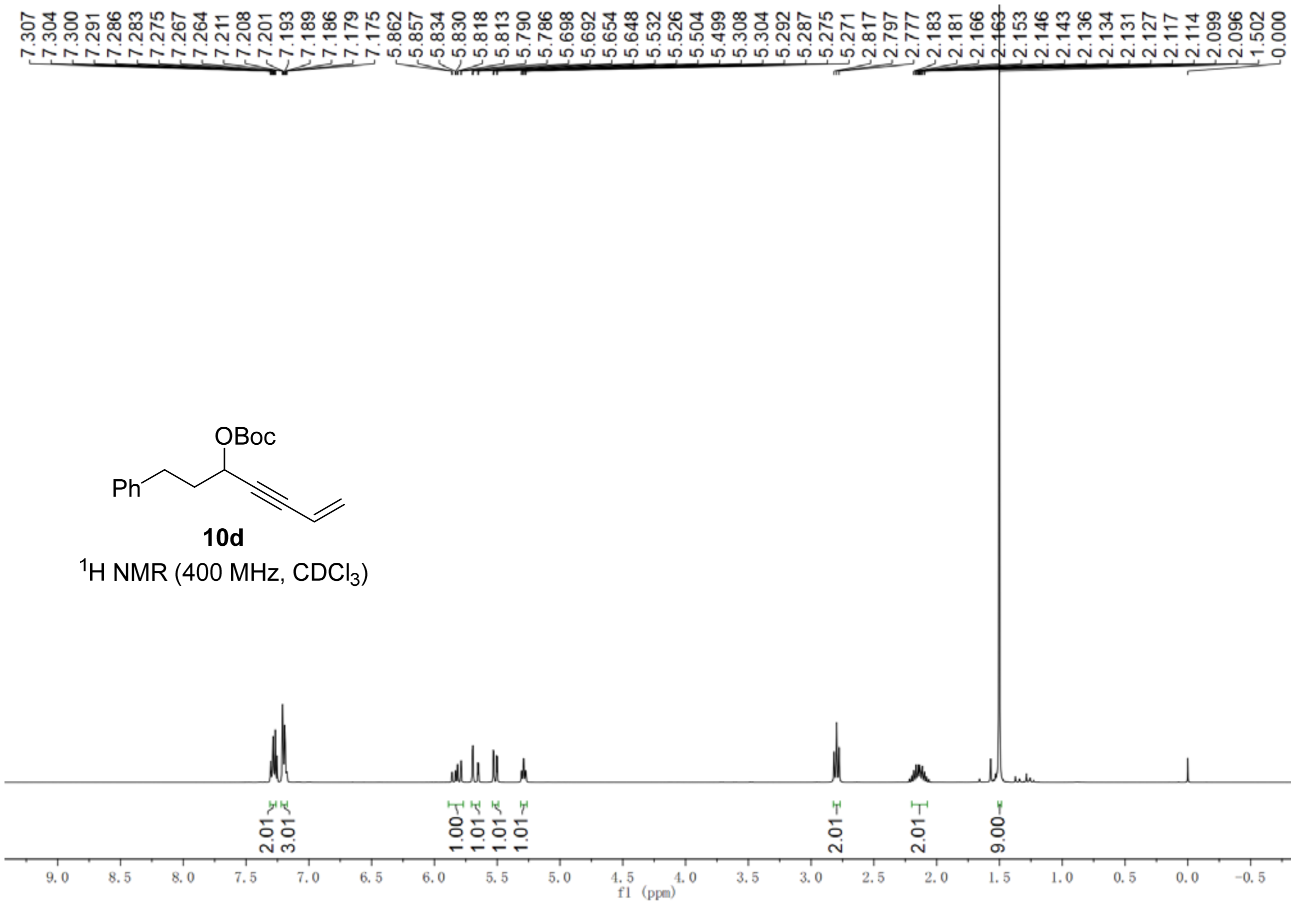

10d

^{1}H NMR (400 MHz, $CDCl_3$)

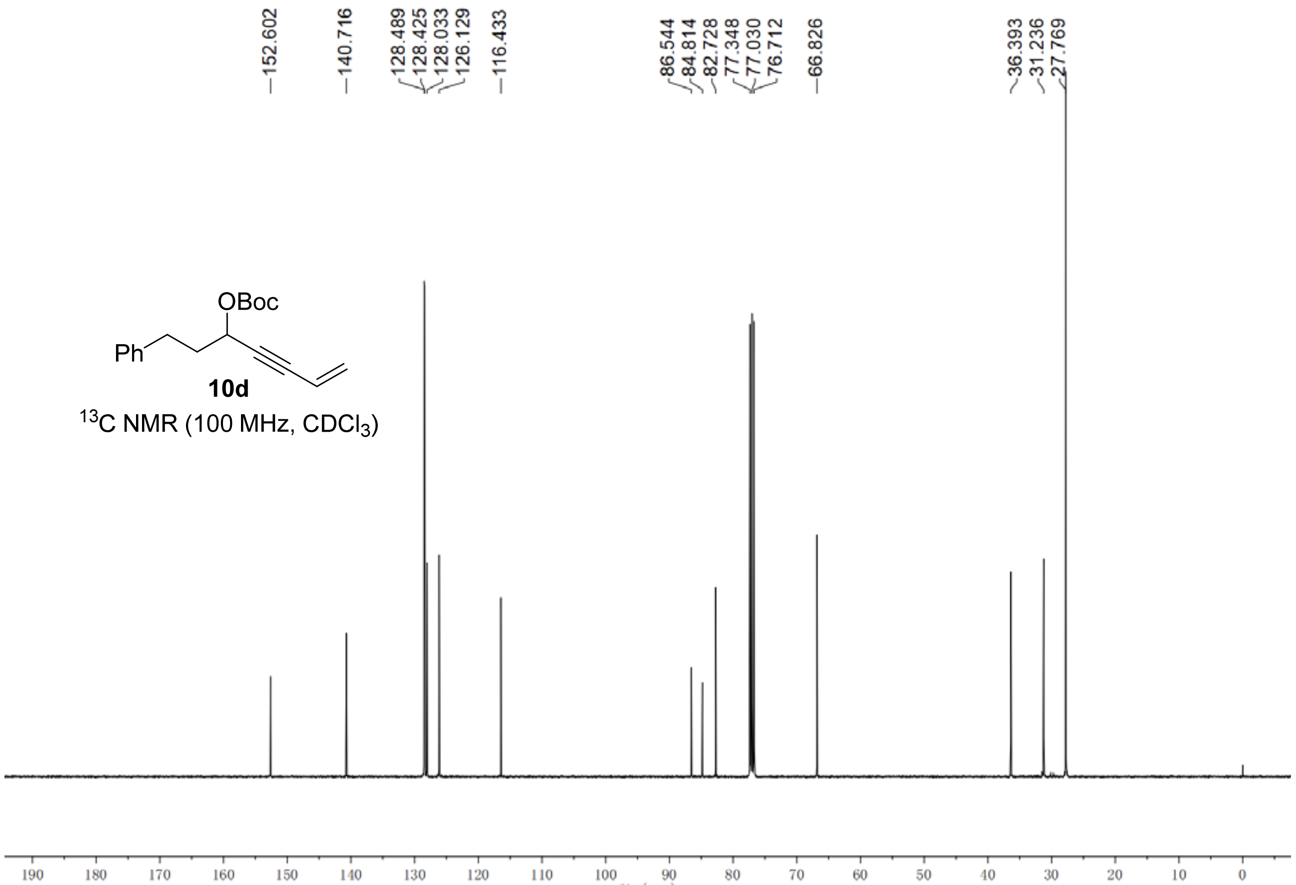

10d

^{13}C NMR (100 MHz, $CDCl_3$)

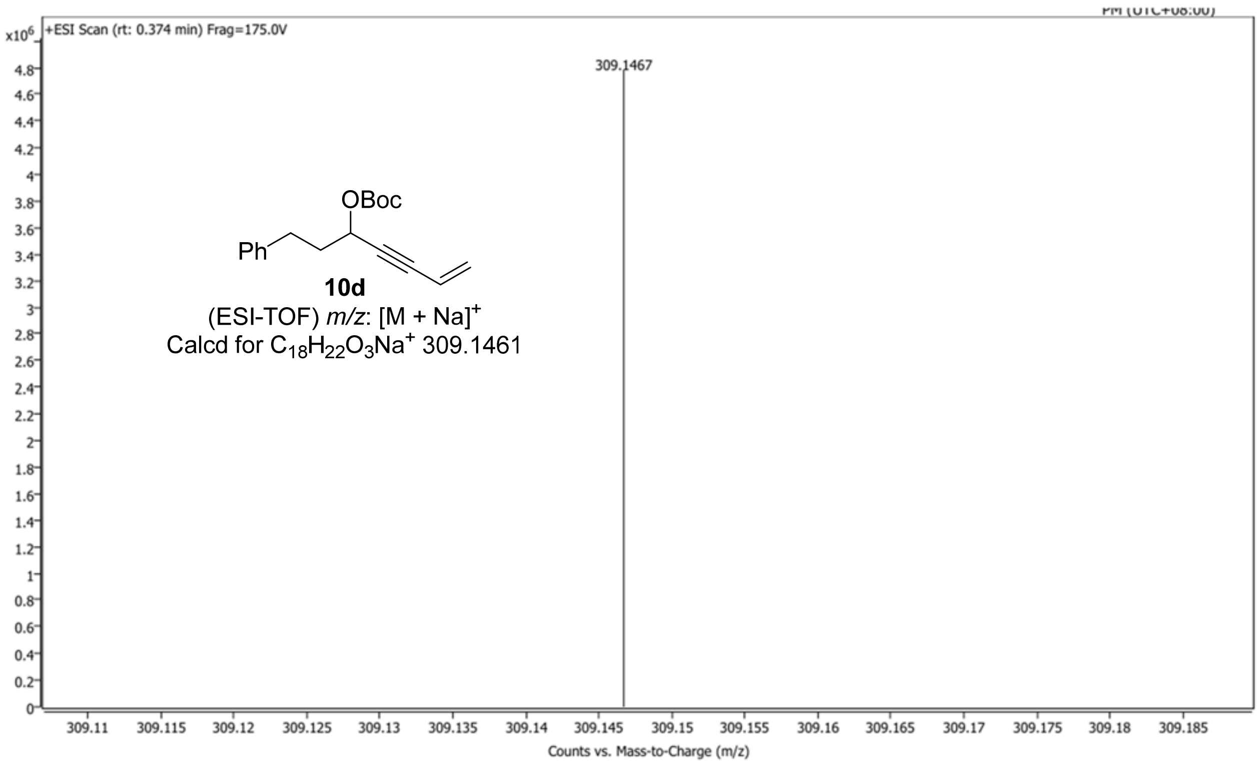
PM (UTC+08:00)
x10^6
+ESI Scan (rt: 0.374 min) Frag=175.0V
309.1467
OBoc
Ph
10d
(ESI-TOF) m/z: [M + Na]+
Calcd for C18H22O3Na+ 309.1461
Counts vs. Mass-to-Charge (m/z)

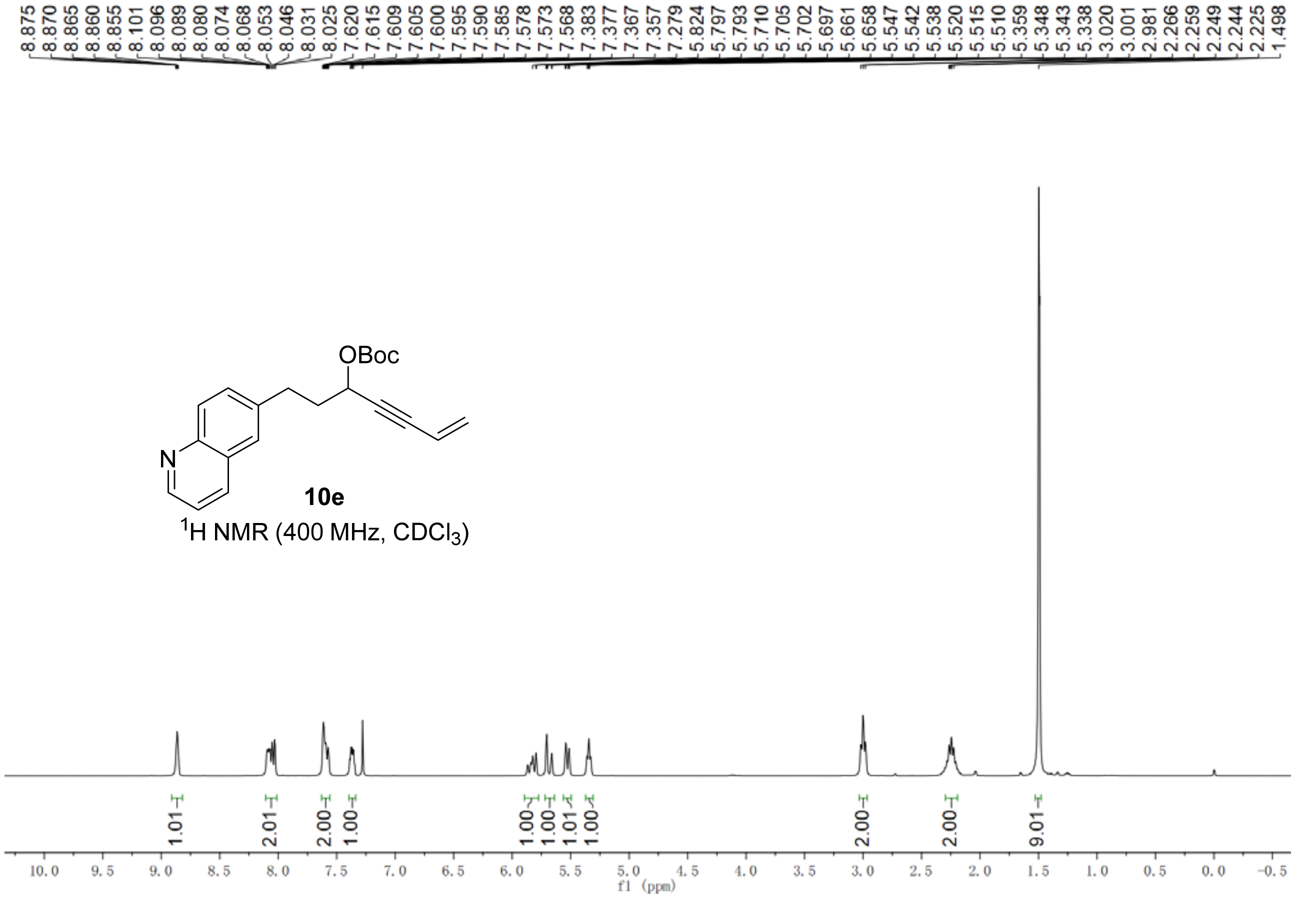
8.875
8.870
8.865
8.860
8.855
8.101
8.096
8.089
8.080
8.074
8.068
8.053
8.046
8.031
8.025
7.620
7.615
7.609
7.605
7.600
7.595
7.590
7.585
7.578
7.573
7.568
7.383
7.377
7.367
7.357
7.279
5.824
5.797
5.793
5.710
5.705
5.702
5.697
5.661
5.658
5.547
5.542
5.538
5.520
5.515
5.510
5.359
5.348
5.343
5.338
3.020
3.001
2.981
2.266
2.259
2.249
2.244
2.225
1.498
OBoc
N
10e
1H NMR (400 MHz, CDCl3)
1.01
2.01
2.00
1.00
1.00
1.00
1.01
1.00
2.00
2.00
9.01
f1 (ppm)

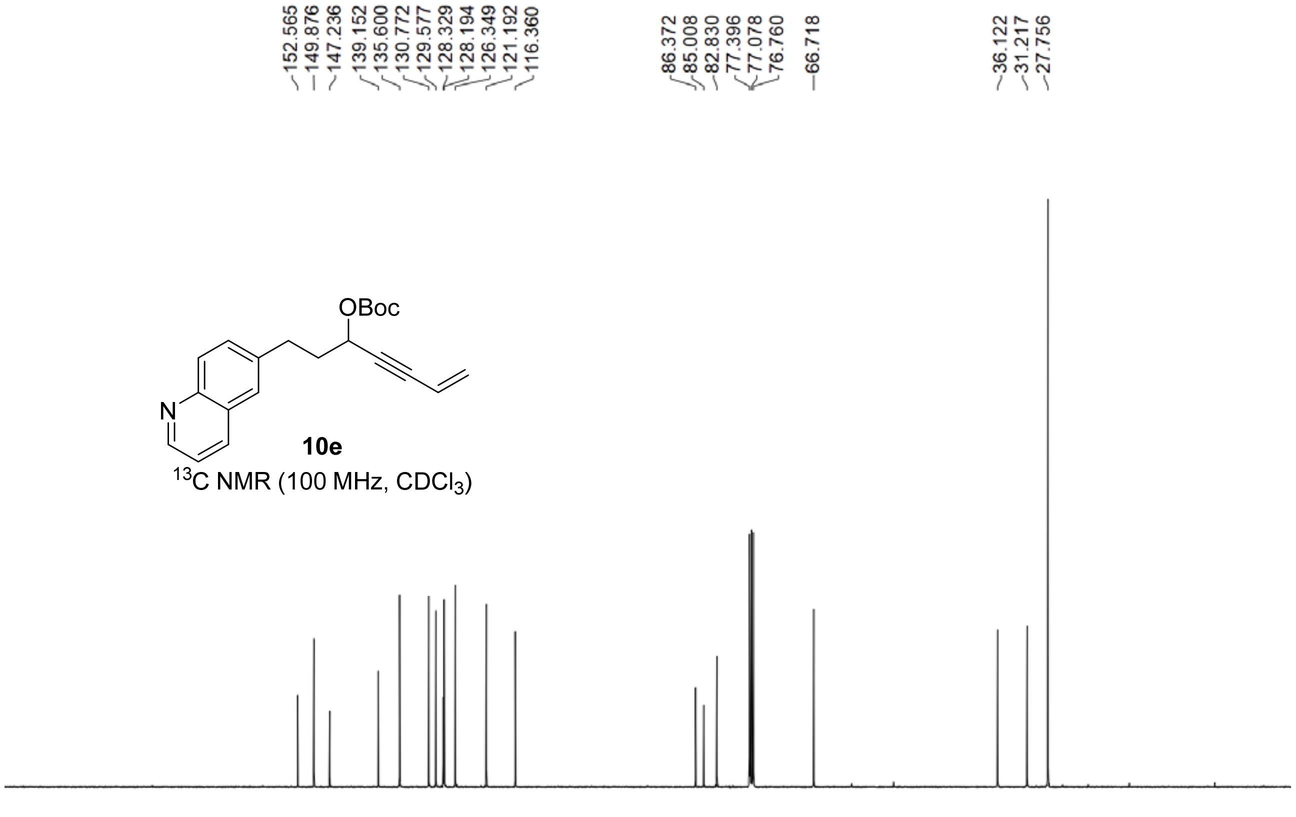
152.565
149.876
147.236
139.152
135.600
130.772
129.577
128.329
128.194
126.349
121.192
116.360
86.372
85.008
82.830
77.396
77.078
76.760
66.718
36.122
31.217
27.756
OBoc
N
10e
13C NMR (100 MHz, CDCl3)
f1 (ppm)

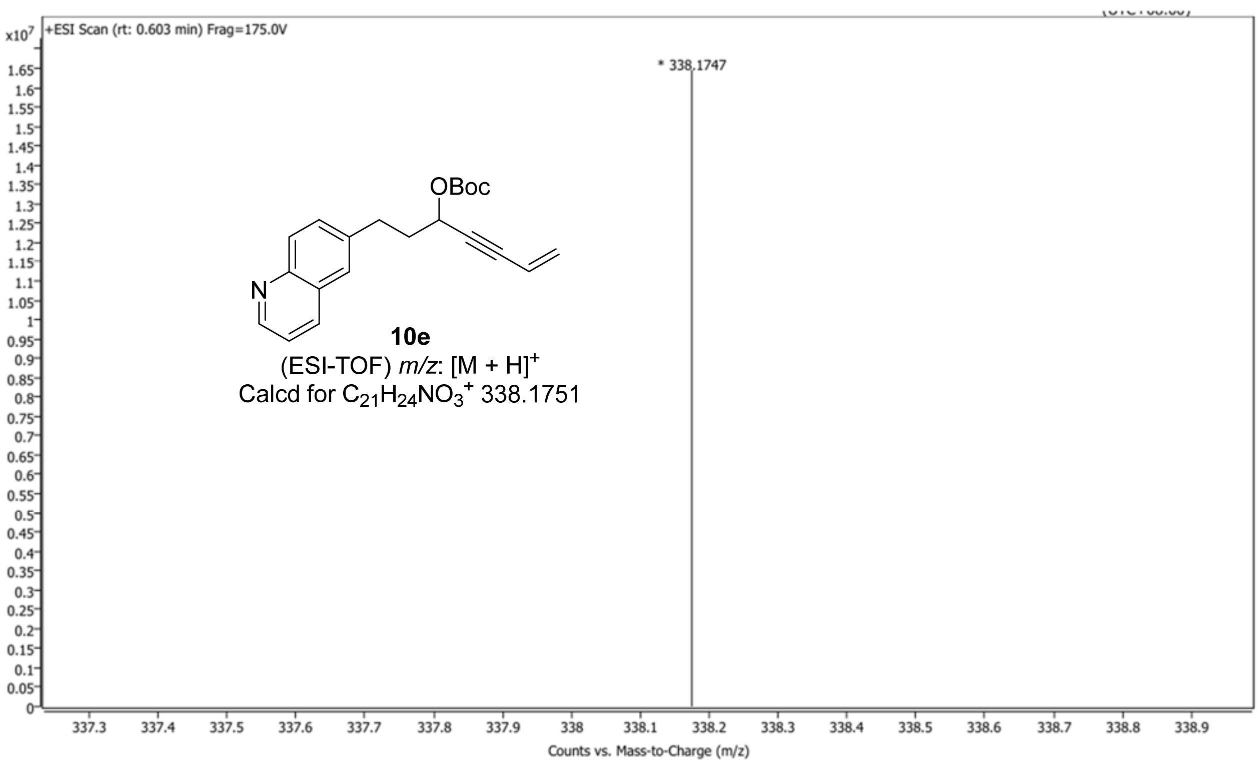

+ESI Scan (rt: 0.603 min) Frag=175.0V
x10^7
* 338.1747
OBoc
N
10e
(ESI-TOF) m/z: [M + H]+
Calcd for C21H24NO3+ 338.1751
Counts vs. Mass-to-Charge (m/z)

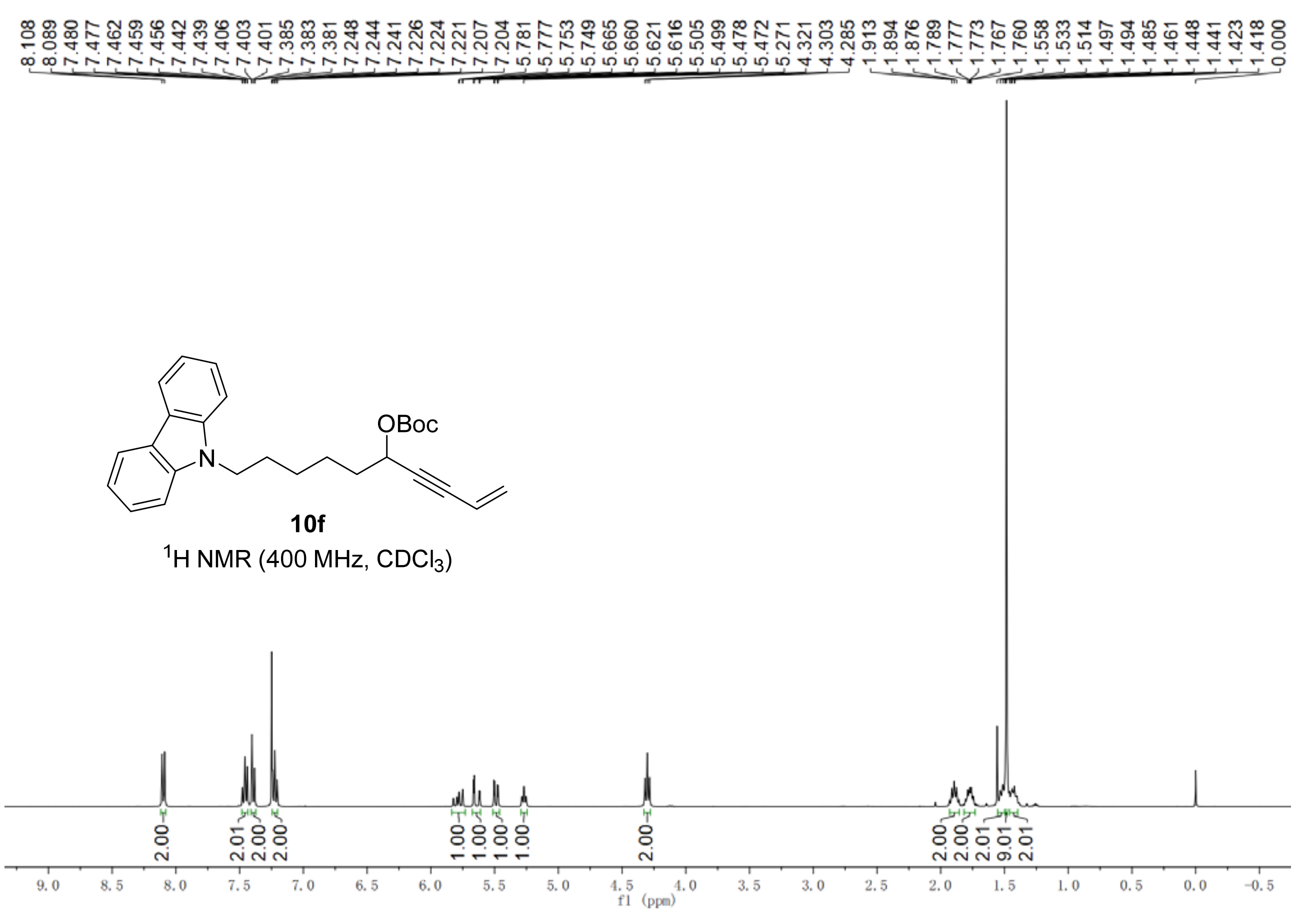

10f

^{1}H NMR (400 MHz, $CDCl_3$)

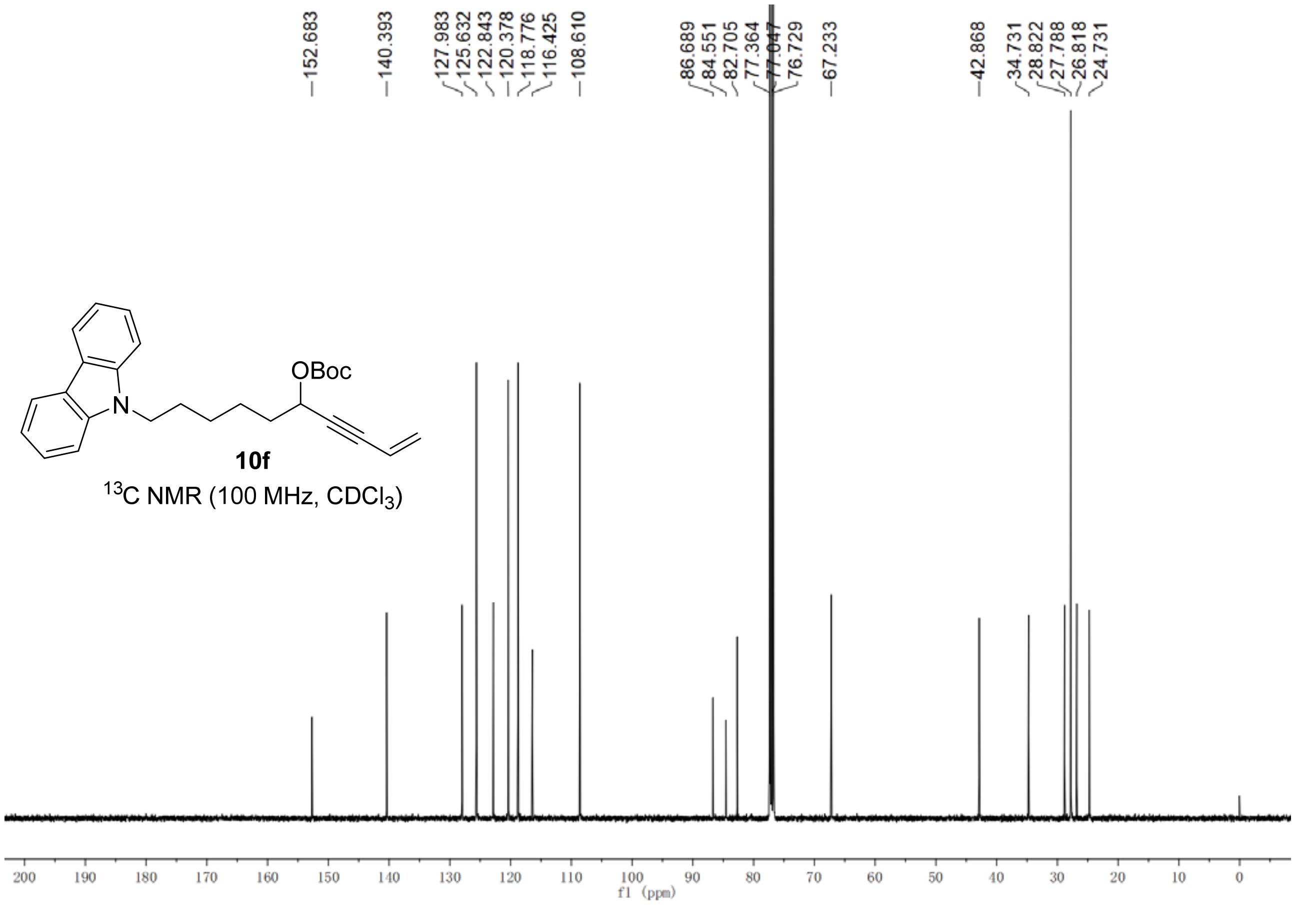

10f

^{13}C NMR (100 MHz, $CDCl_3$)

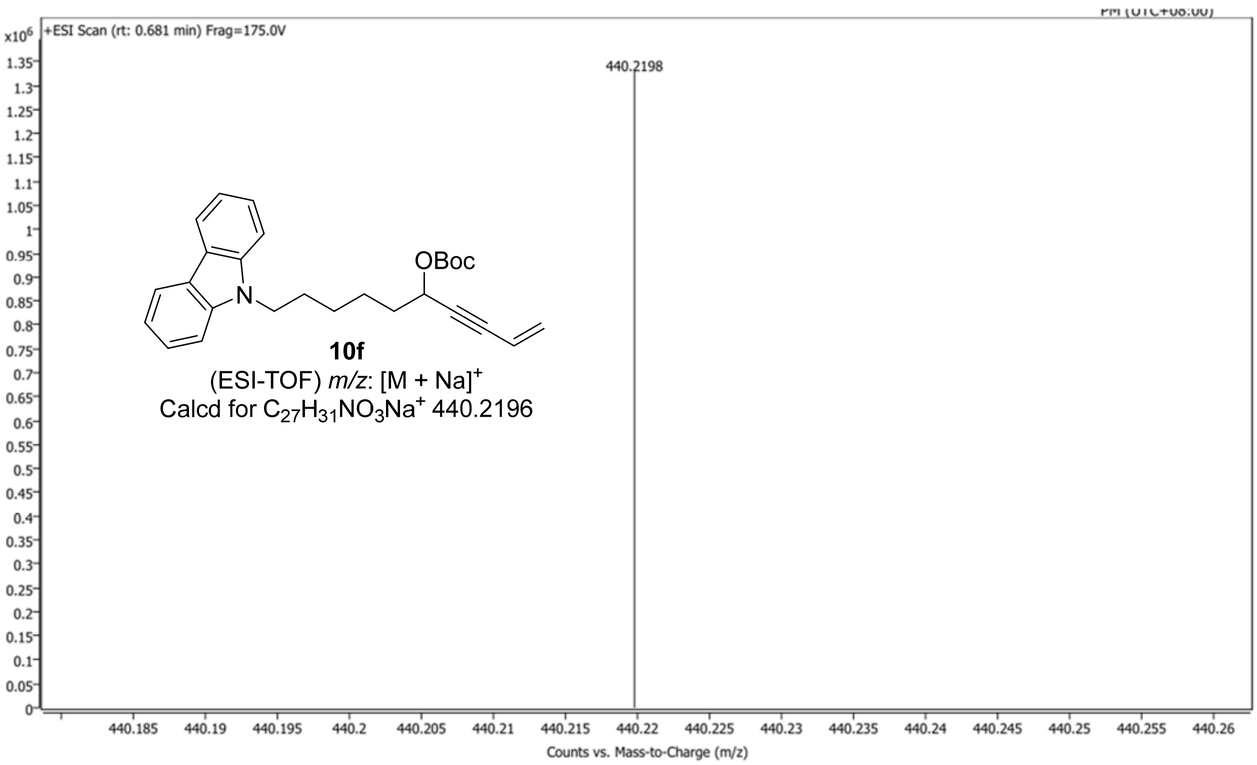

PM (UTC+08:00)
x10^6
+ESI Scan (rt: 0.681 min) Frag=175.0V
440.2198
OBoc
N
10f
(ESI-TOF) m/z: [M + Na]+
Calcd for C27H31NO3Na+ 440.2196
Counts vs. Mass-to-Charge (m/z)

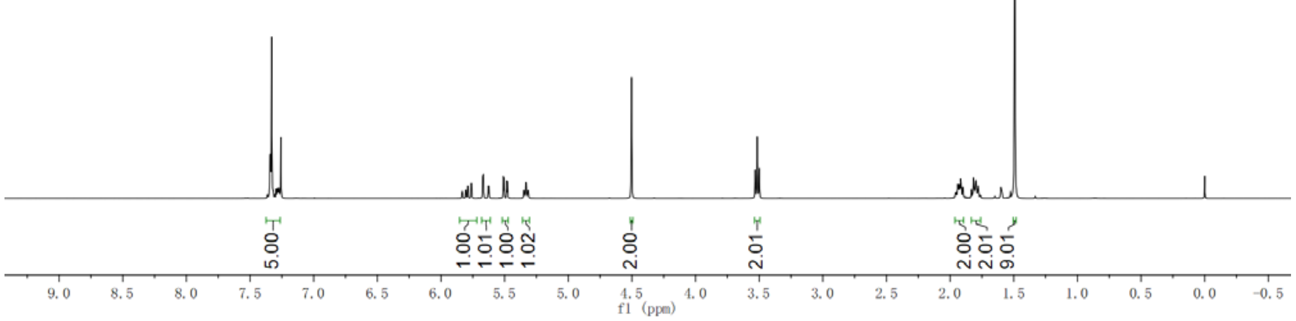

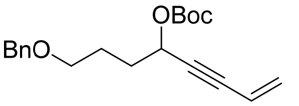

10g

^{1}H NMR (400 MHz, $CDCl_3$)

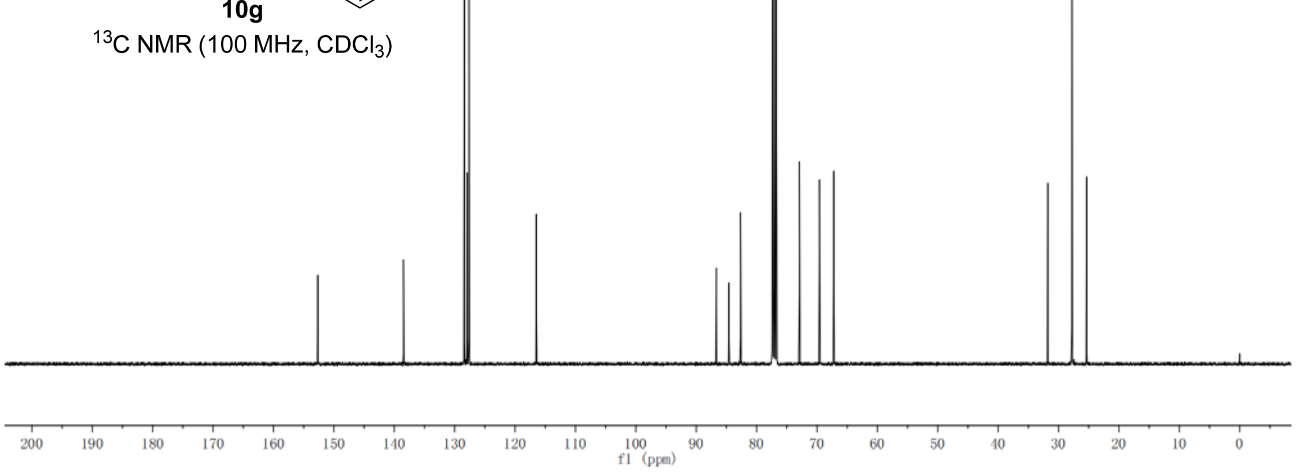

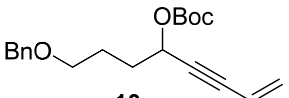

10g

^{13}C NMR (100 MHz, $CDCl_3$)

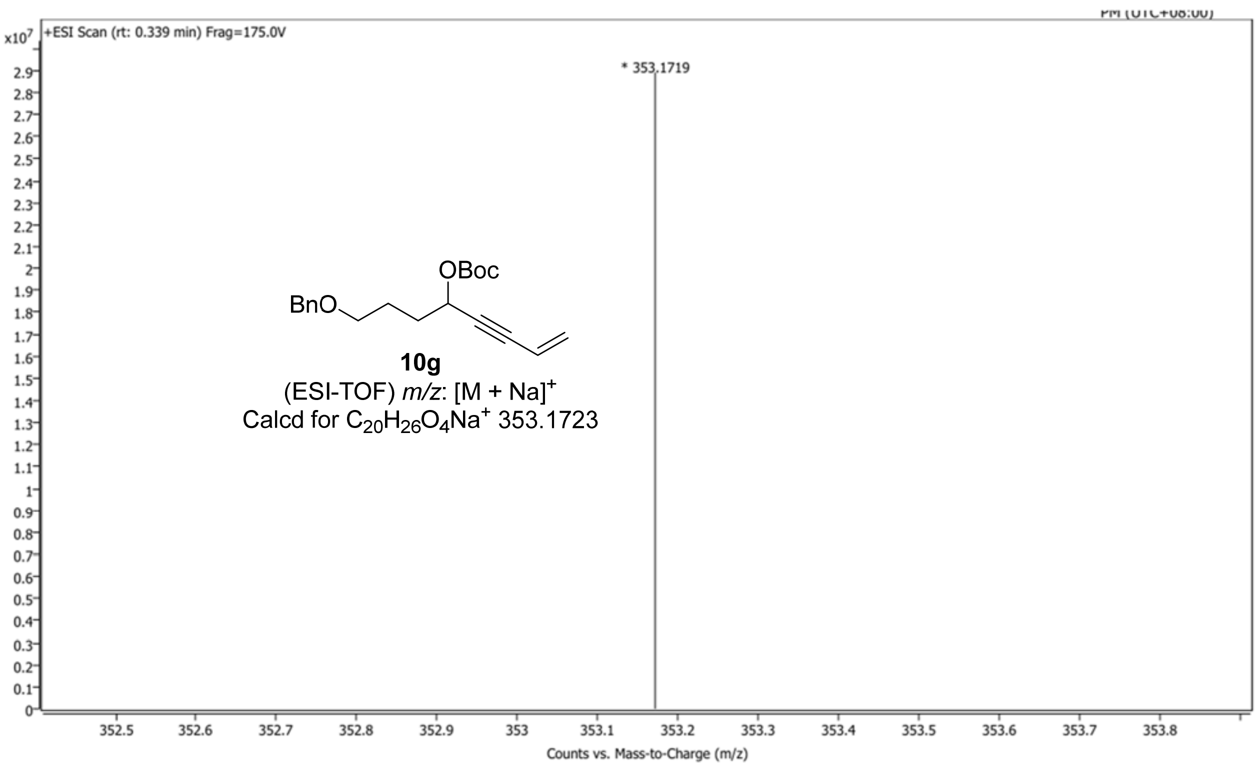

+ESI Scan (rt: 0.339 min) Frag=175.0V
x10^7
* 353.1719
OBoc
BnO
10g
(ESI-TOF) m/z: [M + Na]+
Calcd for C20H26O4Na+ 353.1723
Counts vs. Mass-to-Charge (m/z)

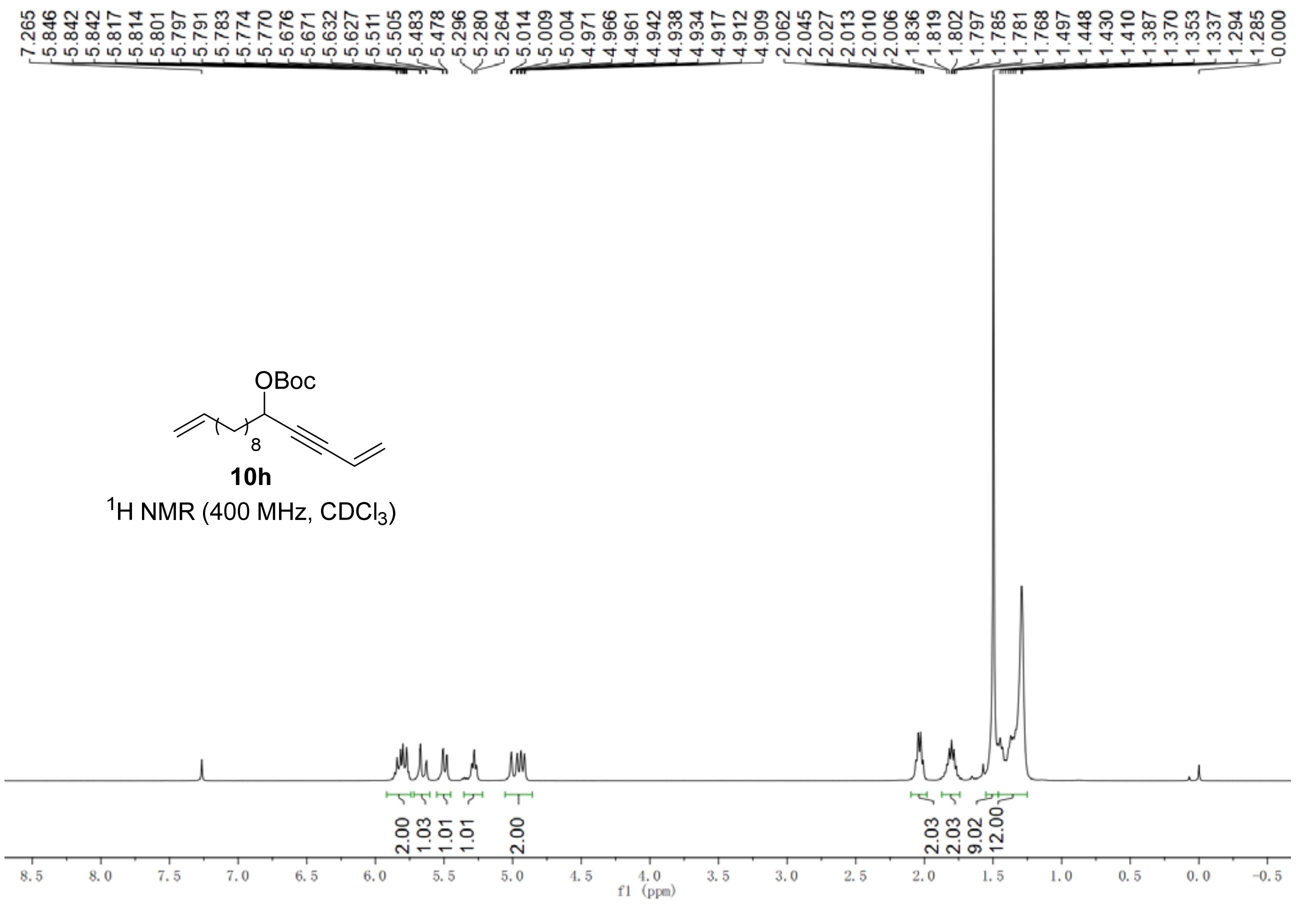
OBoc
8
10h
^{1}H NMR (400 MHz, CDCl3)
2.00
1.03
1.01
1.01
2.00
2.03
2.03
9.02
12.00
f1 (ppm)

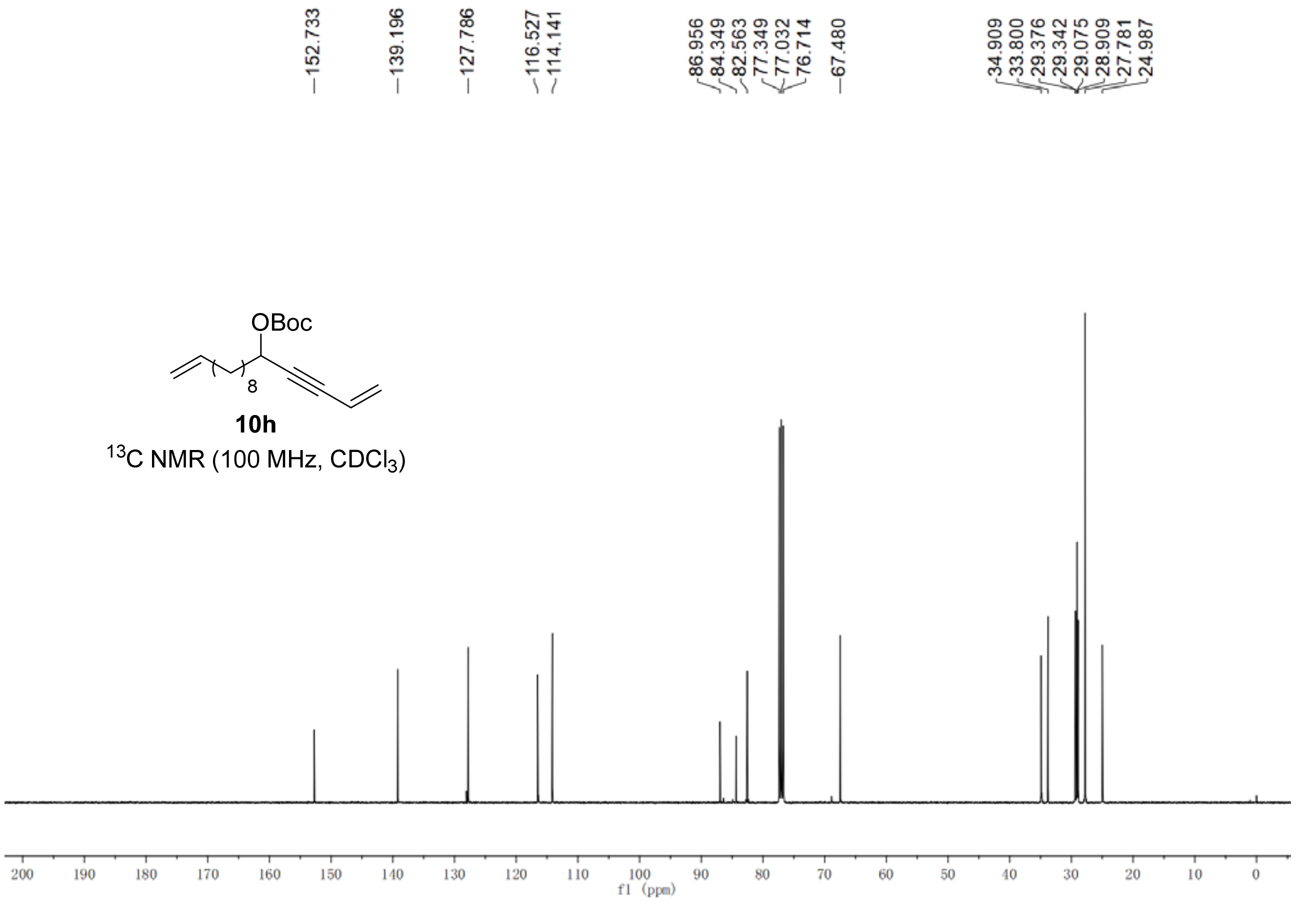
152.733
139.196
127.786
116.527
114.141
86.956
84.349
82.563
77.349
77.032
76.714
67.480
34.909
33.800
29.376
29.342
29.075
28.909
27.781
24.987
OBoc
8
10h
^{13}C NMR (100 MHz, CDCl3)
f1 (ppm)

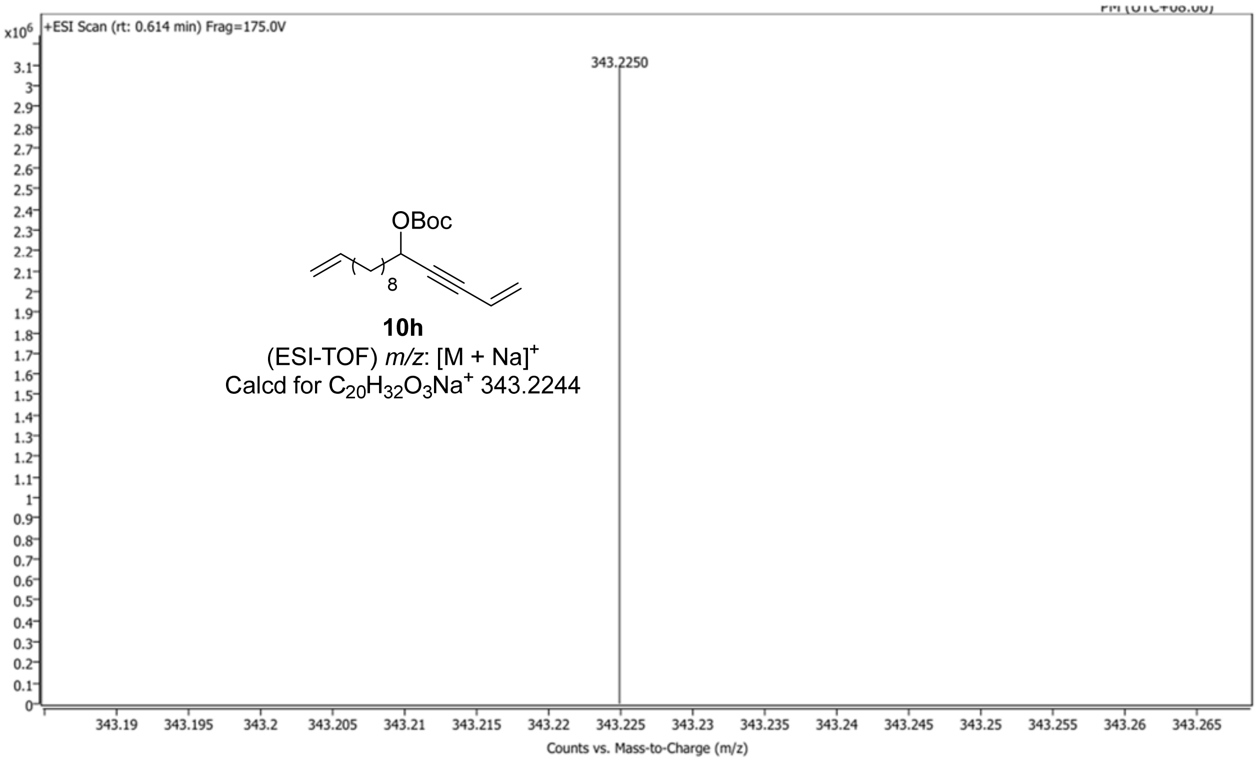
+ESI Scan (rt: 0.614 min) Frag=175.0V
x10^6
343.2250
OBoc
8
10h
(ESI-TOF) m/z: [M + Na]+
Calcd for C20H32O3Na+ 343.2244
Counts vs. Mass-to-Charge (m/z)

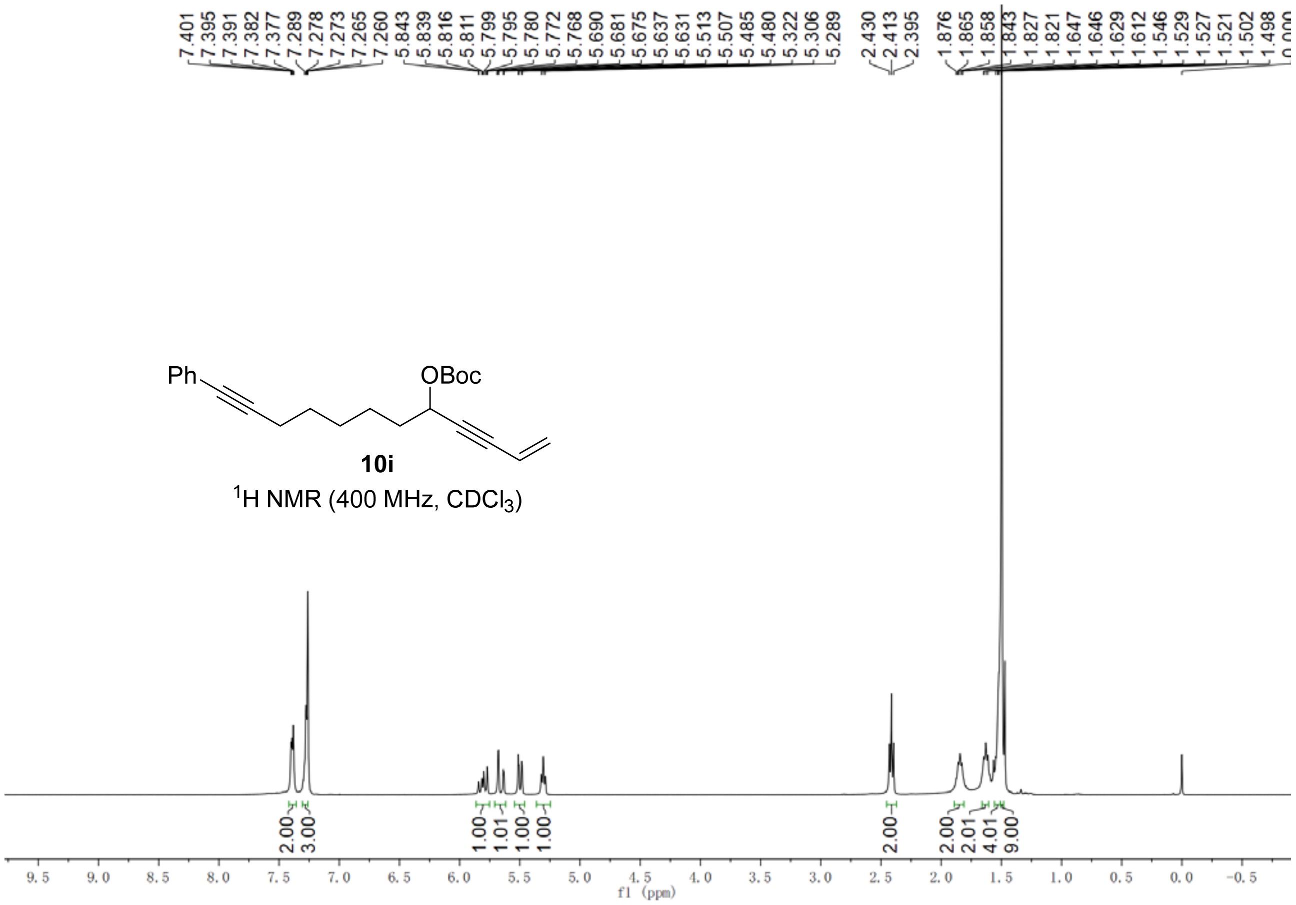
7.401
7.395
7.391
7.382
7.377
7.289
7.278
7.273
7.265
7.260
5.843
5.839
5.816
5.811
5.799
5.795
5.780
5.772
5.768
5.690
5.681
5.675
5.637
5.631
5.513
5.507
5.485
5.480
5.322
5.306
5.289
2.430
2.413
2.395
1.876
1.865
1.858
1.843
1.827
1.821
1.647
1.646
1.629
1.612
1.546
1.529
1.527
1.521
1.502
1.498
Ph
OBoc
10i
1H NMR (400 MHz, CDCl3)
2.00
3.00
1.00
1.01
1.00
1.00
2.00
2.00
2.01
4.01
9.00
9.5
9.0
8.5
8.0
7.5
7.0
6.5
6.0
5.5
5.0
4.5
4.0
3.5
3.0
2.5
2.0
1.5
1.0
0.5
0.0
-0.5
f1 (ppm)

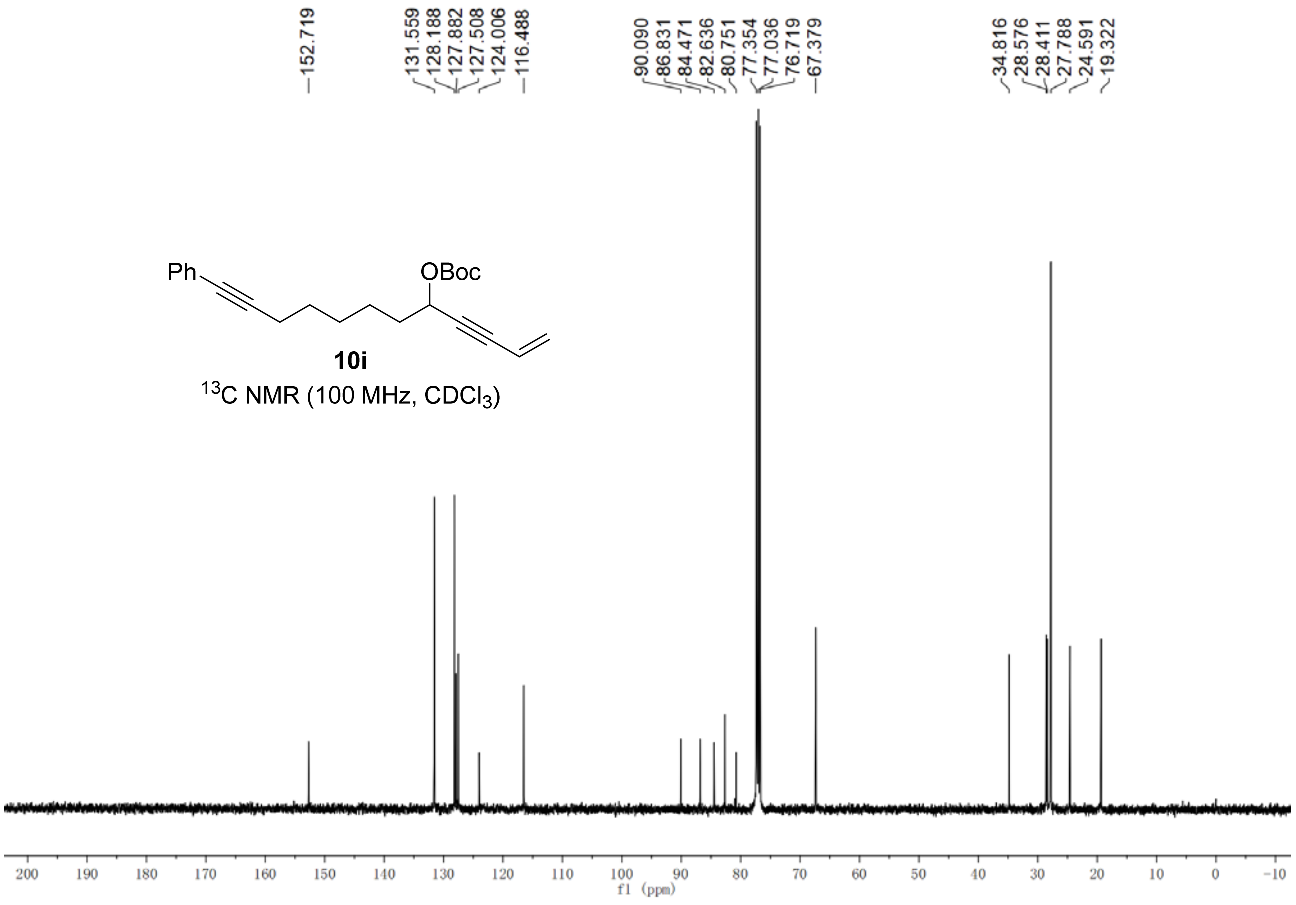
152.719
131.559
128.188
127.882
127.508
124.006
116.488
90.090
86.831
84.471
82.636
80.751
77.354
77.036
76.719
67.379
34.816
28.576
28.411
27.788
24.591
19.322
Ph
OBoc
10i
13C NMR (100 MHz, CDCl3)
200
190
180
170
160
150
140
130
120
110
100
90
80
70
60
50
40
30
20
10
0
-10
f1 (ppm)

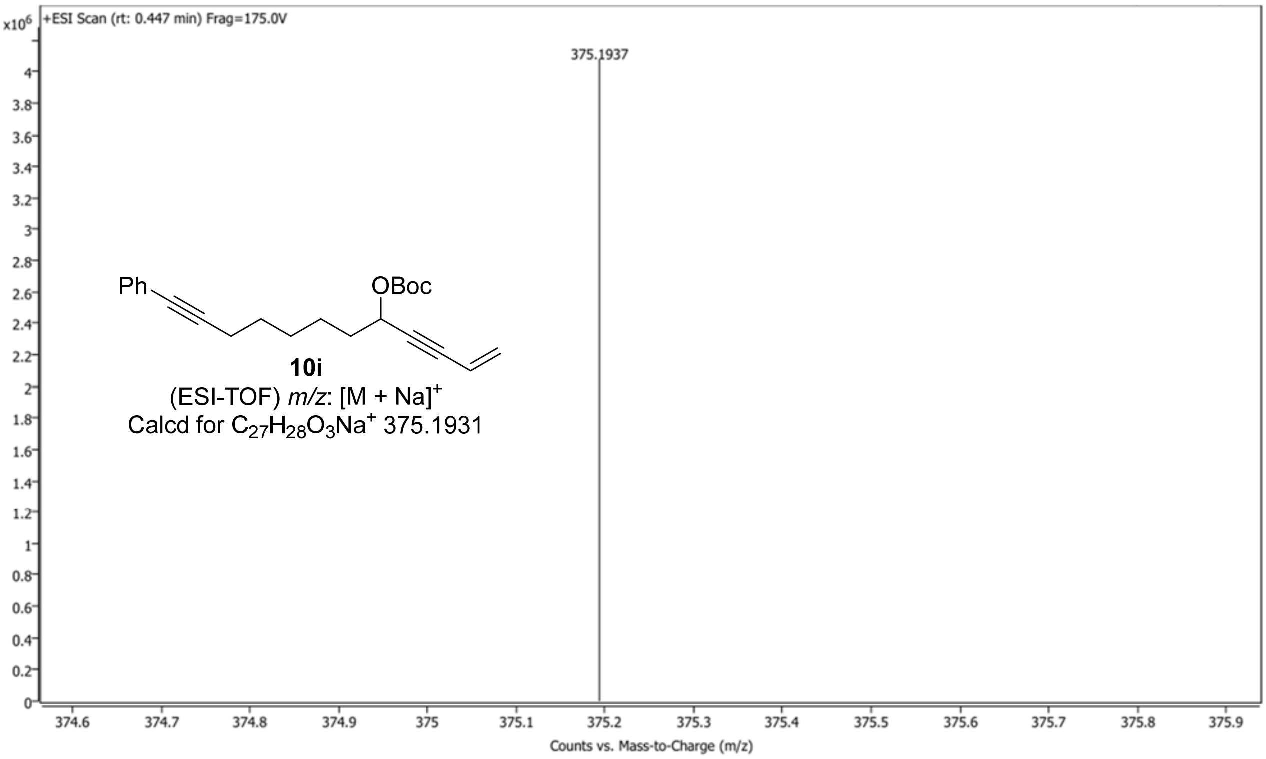

+ESI Scan (rt: 0.447 min) Frag=175.0V
x10^6
375.1937
Ph
OBoc
10i
(ESI-TOF) m/z: [M + Na]+
Calcd for C27H28O3Na+ 375.1931
Counts vs. Mass-to-Charge (m/z)

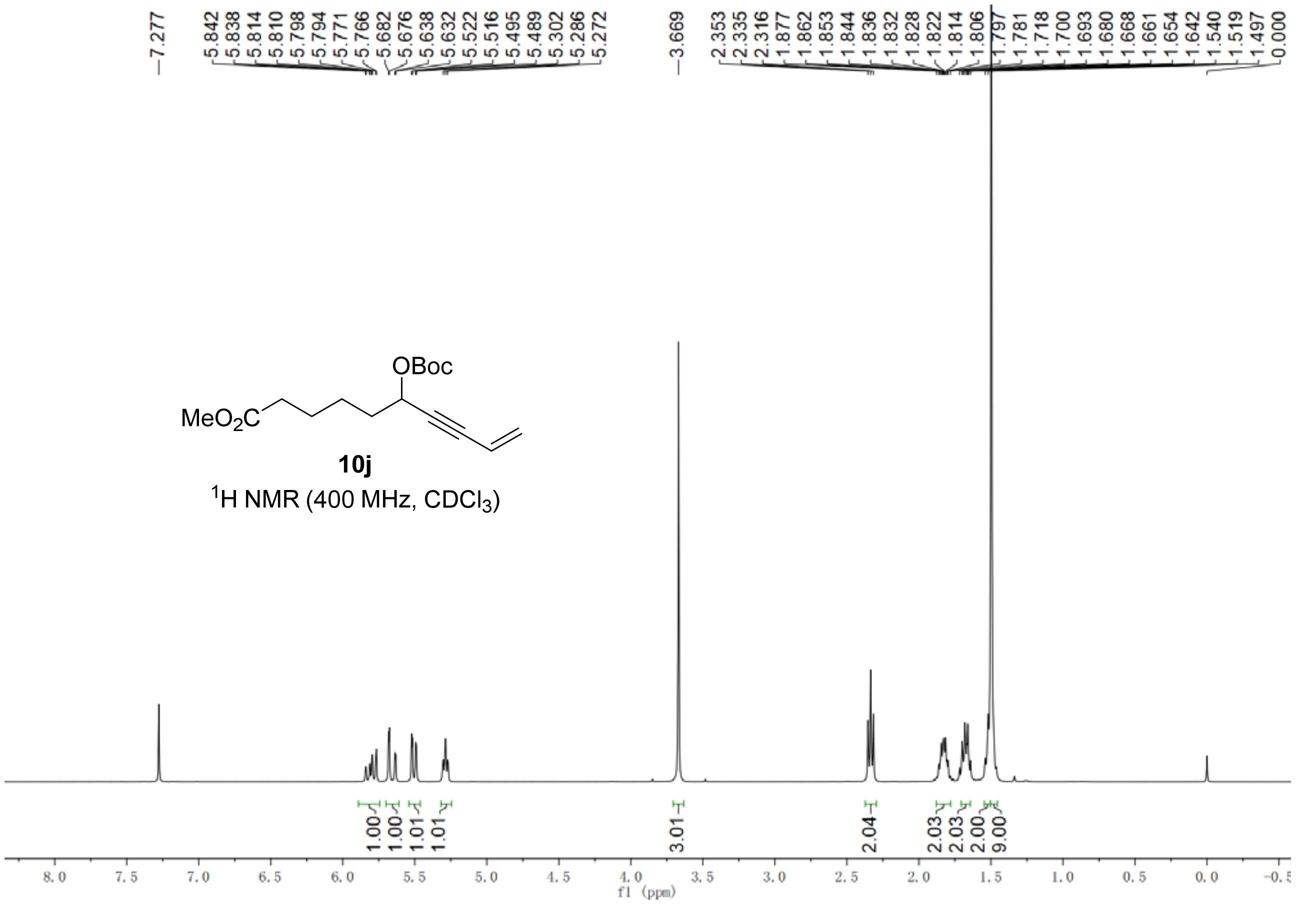

OBoc
MeO2C
10j
1H NMR (400 MHz, CDCl3)
f1 (ppm)

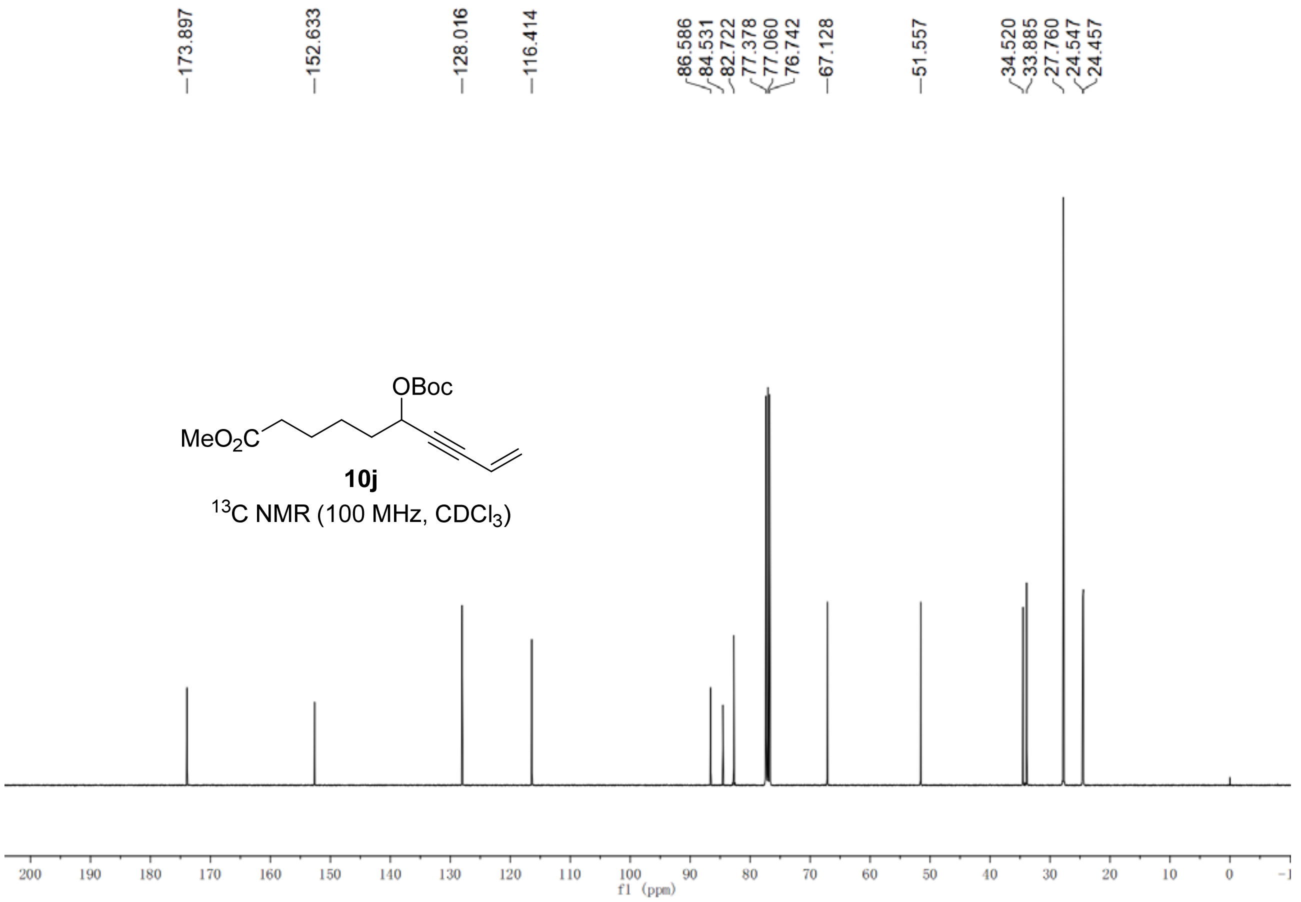

OBoc
MeO2C
10j
13C NMR (100 MHz, CDCl3)
f1 (ppm)

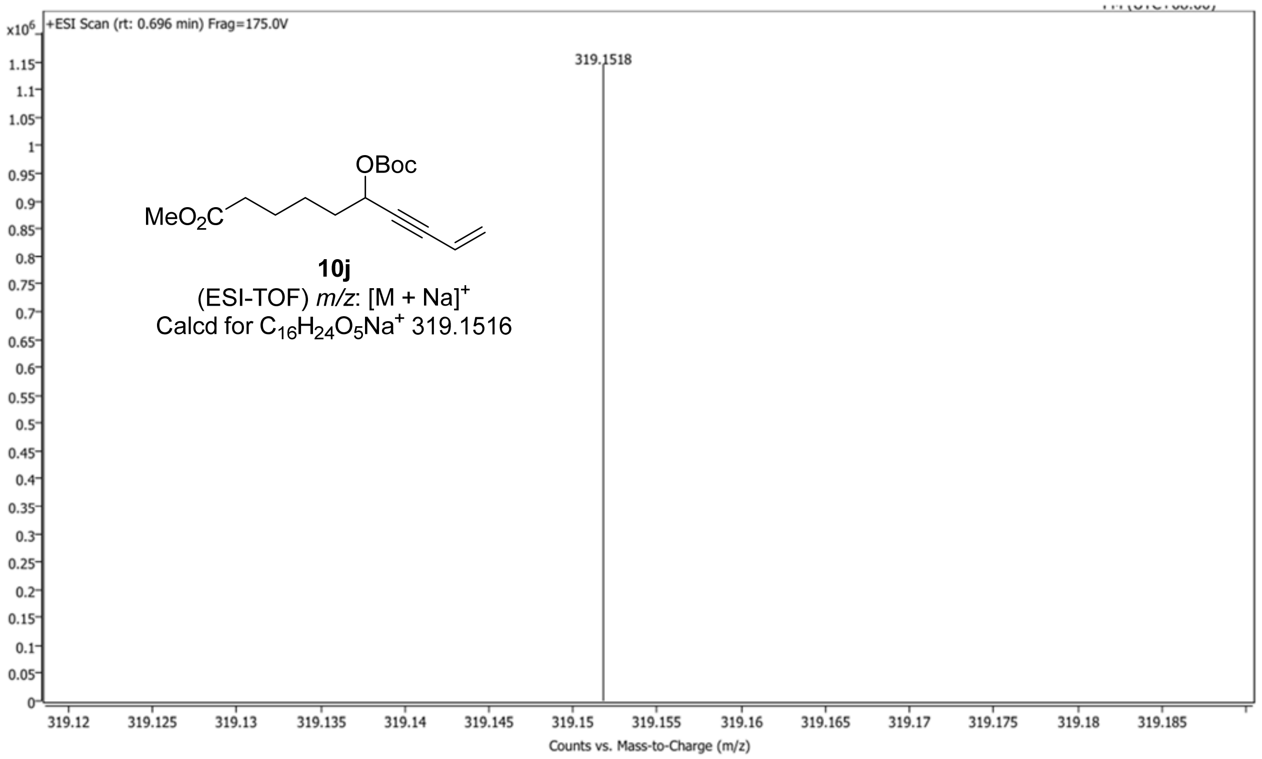
+ESI Scan (rt: 0.696 min) Frag=175.0V
x10^6
319.1518
OBoc
MeO2C
10j
(ESI-TOF) m/z: [M + Na]+
Calcd for C16H24O5Na+ 319.1516
Counts vs. Mass-to-Charge (m/z)

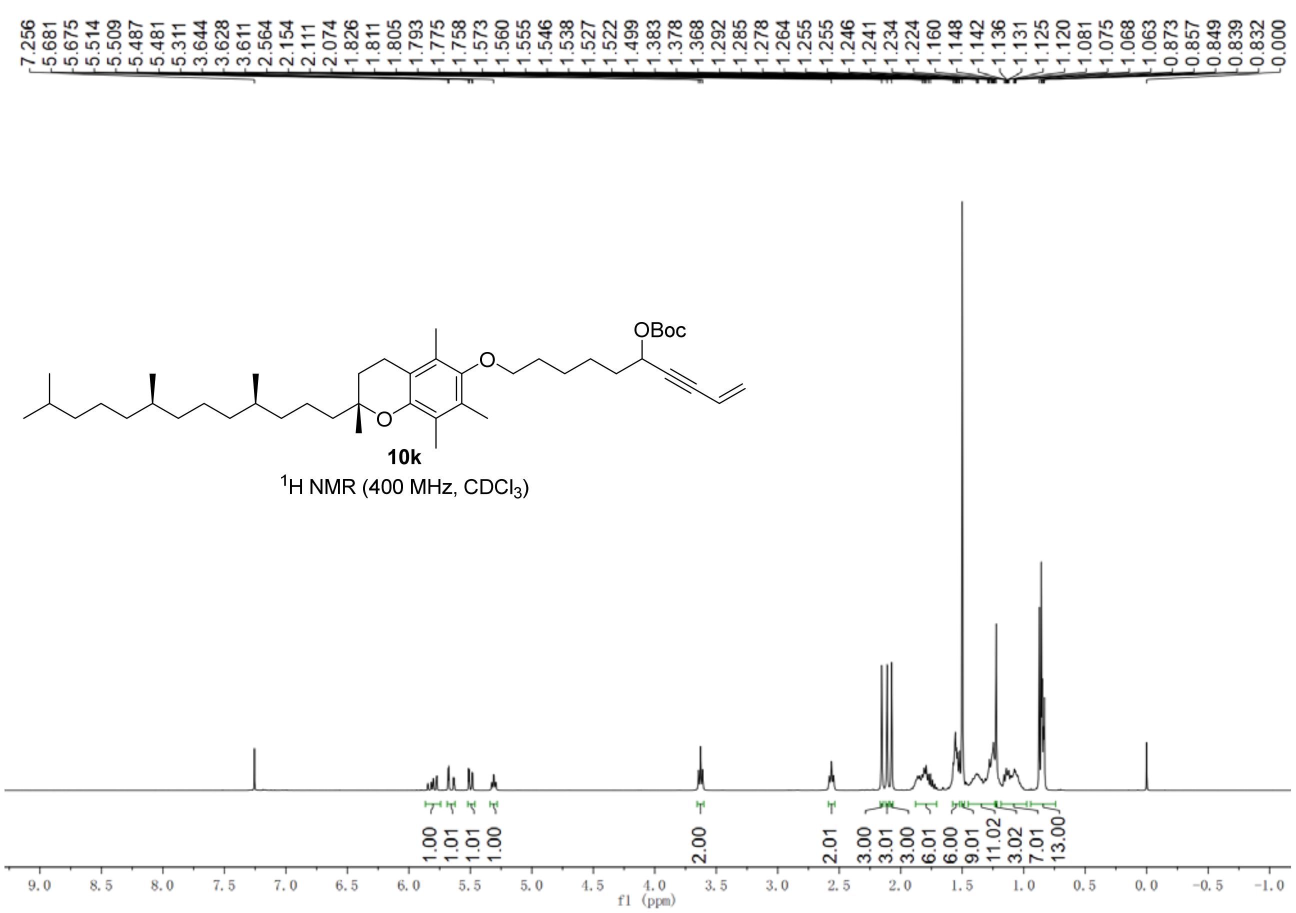

10k

^{1}H NMR (400 MHz, $CDCl_3$)

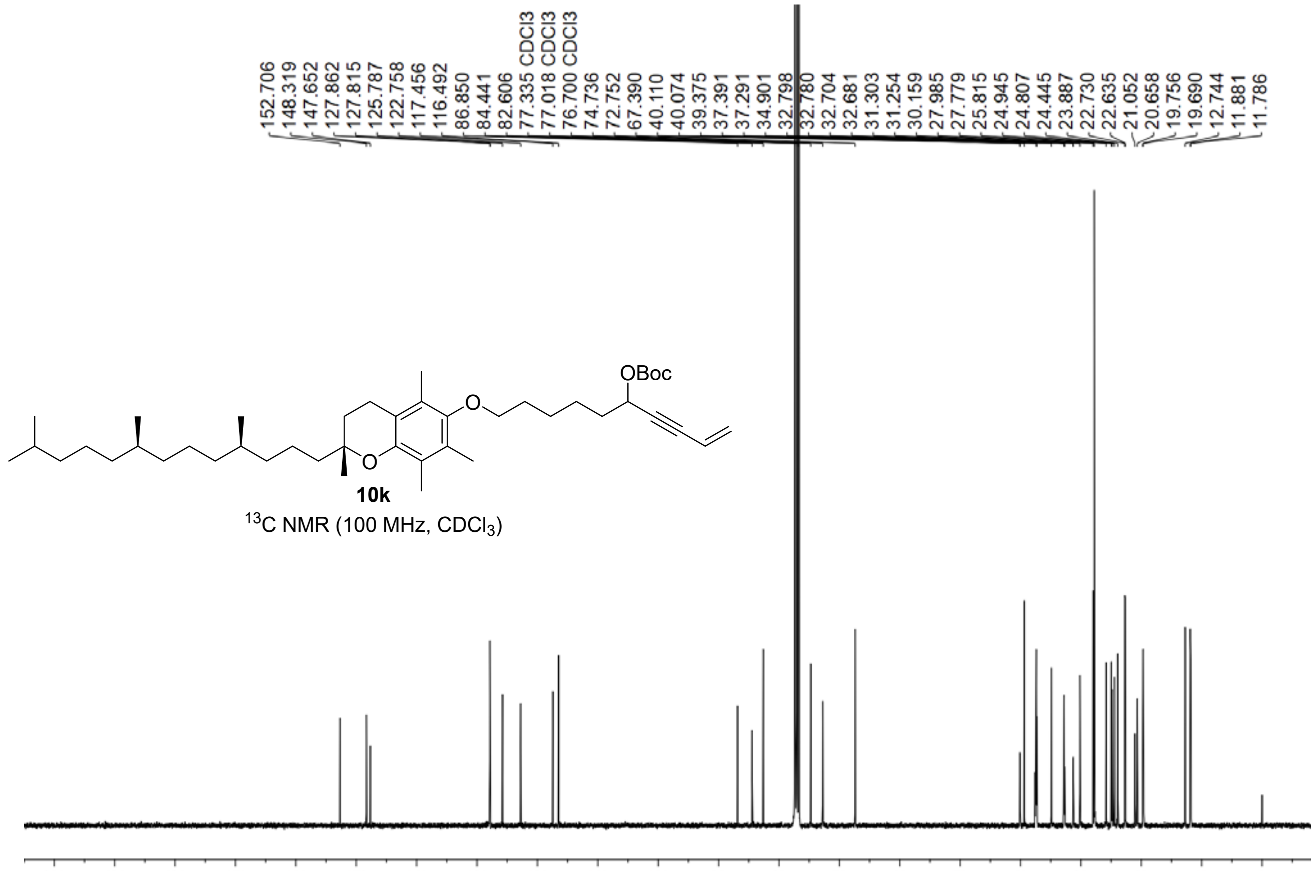

10k

^{13}C NMR (100 MHz, $CDCl_3$)

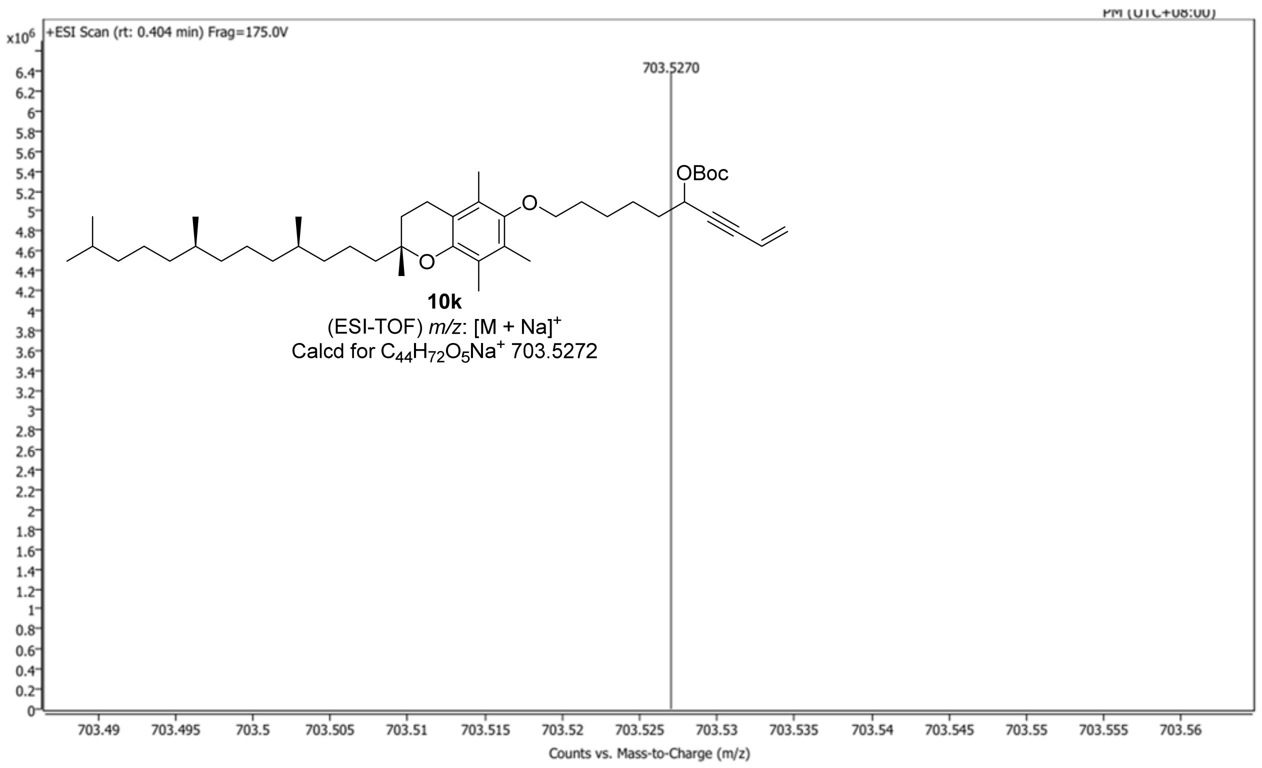
PM (UTC+08:00)
x10^6
+ESI Scan (rt: 0.404 min) Frag=175.0V
703.5270
OBoc
O
O
10k
(ESI-TOF) m/z: [M + Na]+
Calcd for C44H72O5Na+ 703.5272
Counts vs. Mass-to-Charge (m/z)

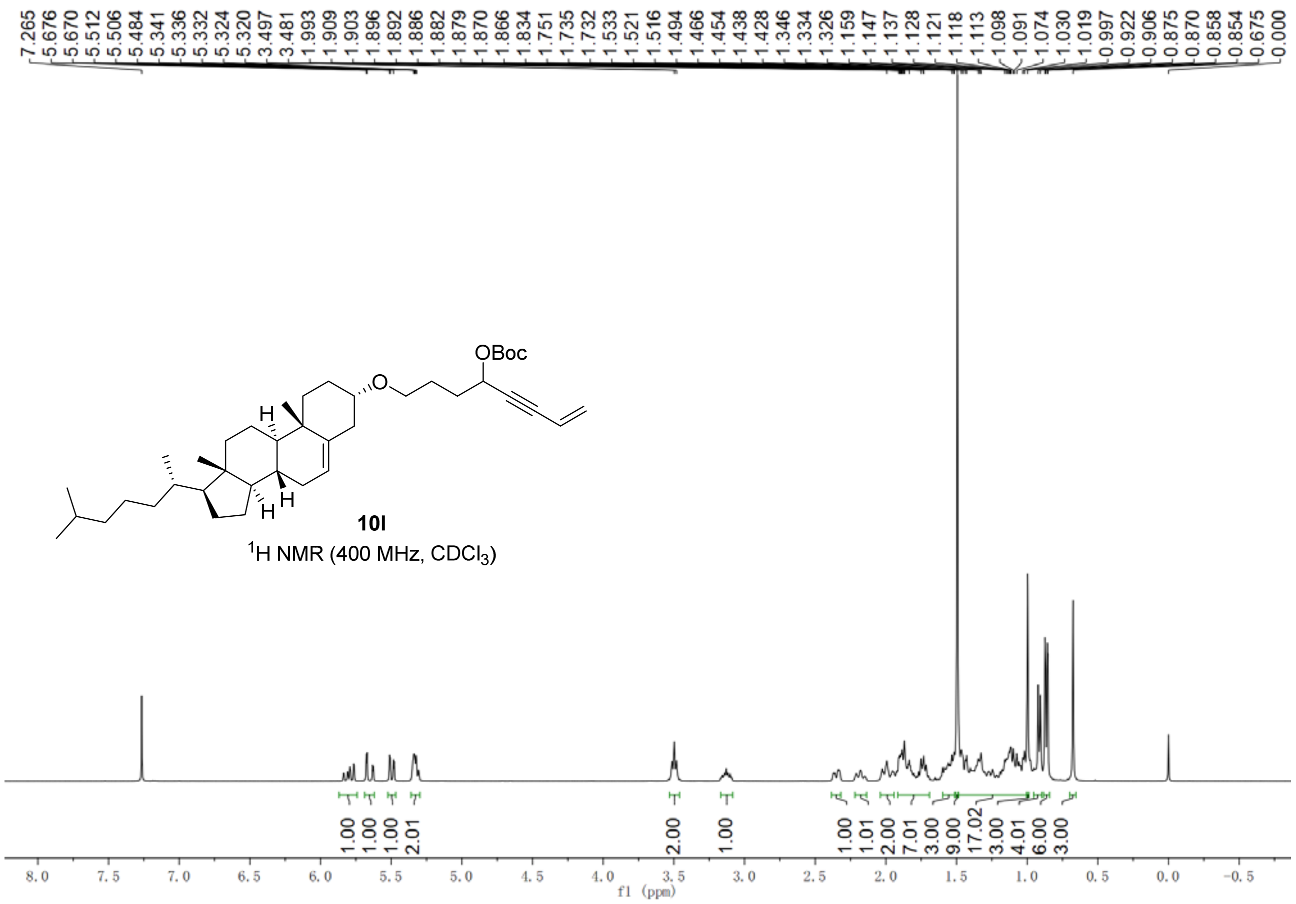

10l

^{1}H NMR (400 MHz, $CDCl_3$)

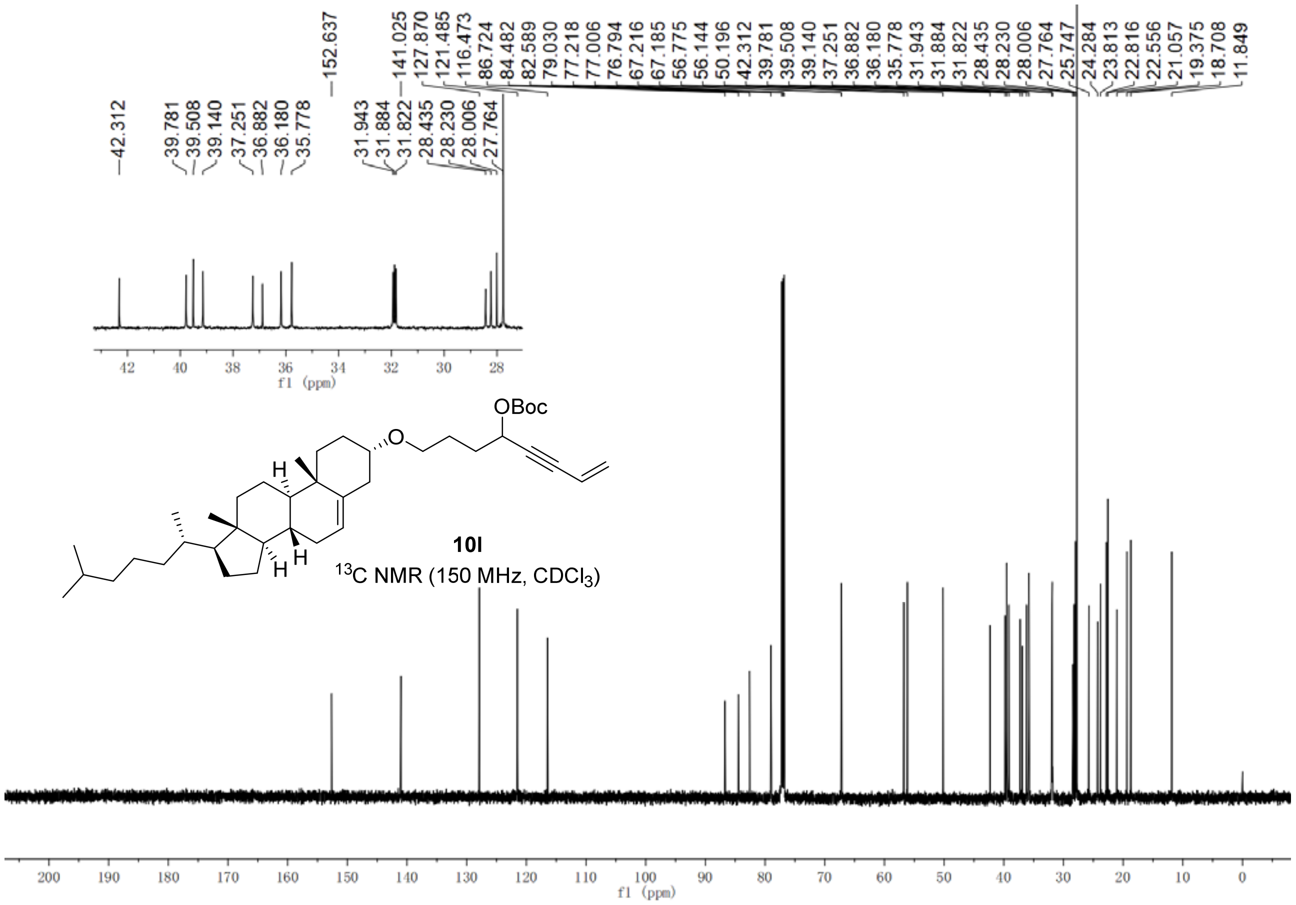

10l

^{13}C NMR (150 MHz, $CDCl_3$)

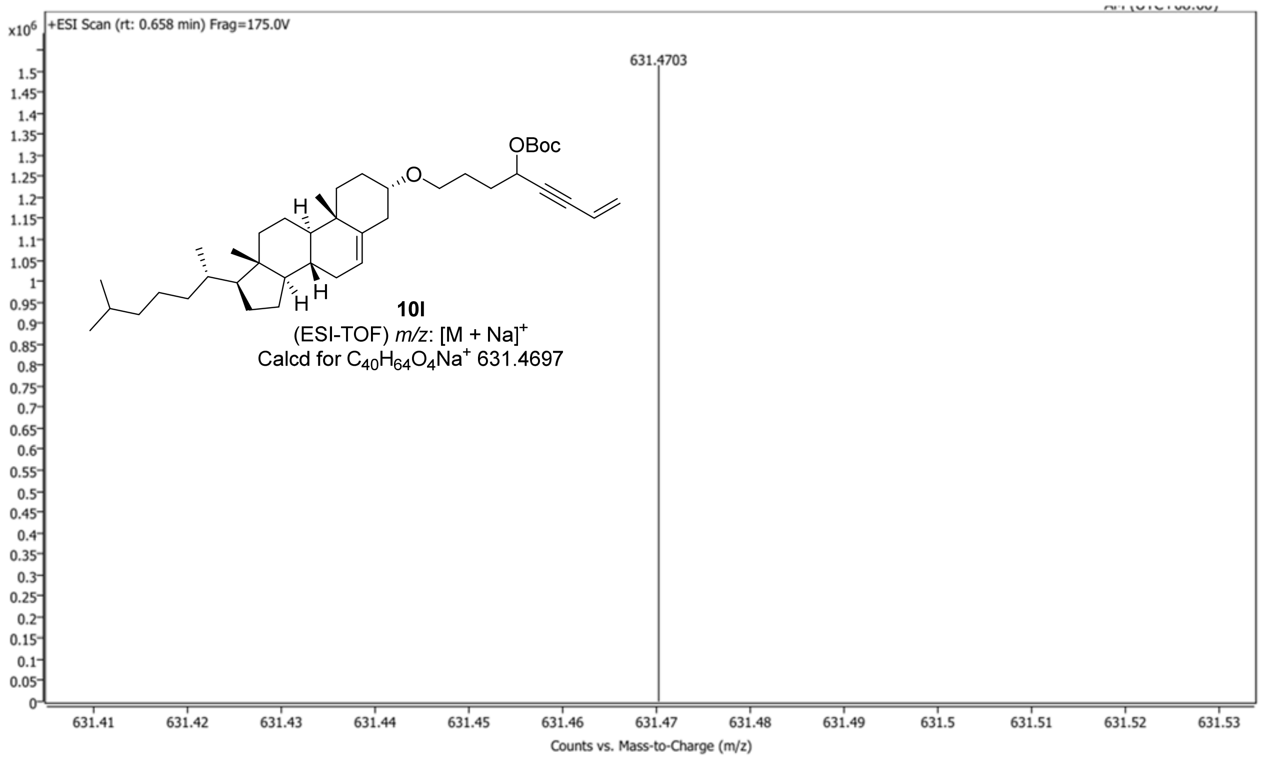
+ESI Scan (rt: 0.658 min) Frag=175.0V
x10^6
631.4703
OBoc
10l
(ESI-TOF) m/z: [M + Na]+
Calcd for C40H64O4Na+ 631.4697
Counts vs. Mass-to-Charge (m/z)

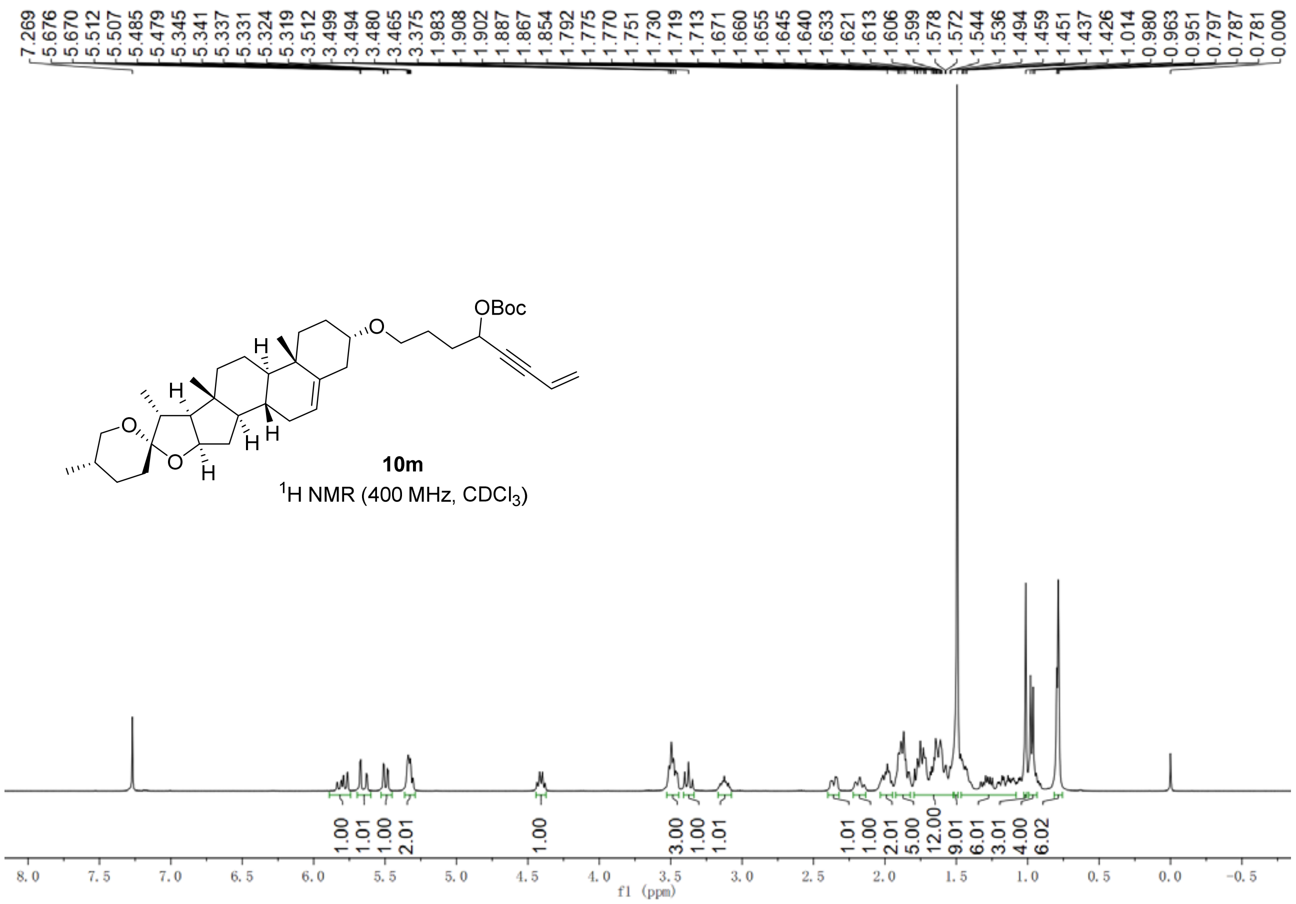
7.269 5.676 5.670 5.512 5.507 5.485 5.479 5.345 5.341 5.337 5.331 5.324 5.319 3.512 3.499 3.494 3.480 3.465 3.375 1.983 1.908 1.902 1.887 1.867 1.854 1.792 1.775 1.770 1.751 1.730 1.719 1.713 1.671 1.660 1.655 1.645 1.640 1.633 1.621 1.613 1.606 1.599 1.578 1.572 1.544 1.536 1.494 1.459 1.451 1.437 1.426 1.014 0.980 0.963 0.951 0.797 0.787 0.781 0.000
OBoc
O
H
H
H
H
O
O
H
10m
^{1}H NMR (400 MHz, $CDCl_3$)
1.00 1.01 1.00 2.01 1.00 3.00 1.00 1.01 1.01 1.00 2.01 5.00 12.00 9.01 6.01 3.01 4.00 6.02
f1 (ppm)

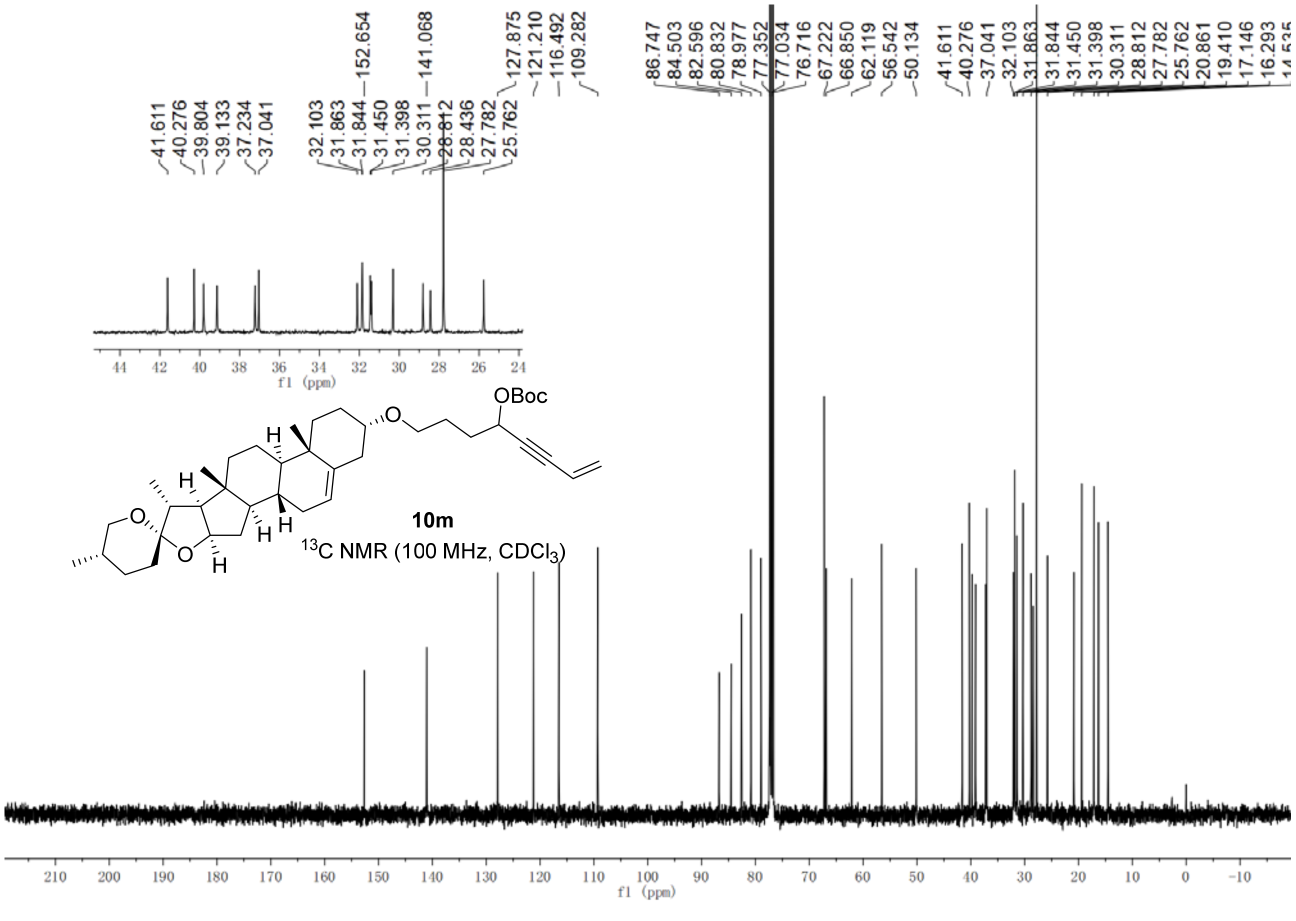
86.747 84.503 82.596 80.832 78.977 77.352 77.034 76.716 67.222 66.850 62.119 56.542 50.134 41.611 40.276 37.041 32.103 31.863 31.844 31.450 31.398 30.311 28.812 27.782 25.762 20.861 19.410 17.146 16.293
152.654 141.068 127.875 121.210 116.492 109.282
41.611 40.276 39.804 39.133 37.234 37.041 32.103 31.863 31.844 31.450 31.398 30.311 28.812 28.436 27.782 25.762
f1 (ppm)
OBoc
O
H
H
H
H
O
O
H
10m
^{13}C NMR (100 MHz, $CDCl_3$)
f1 (ppm)

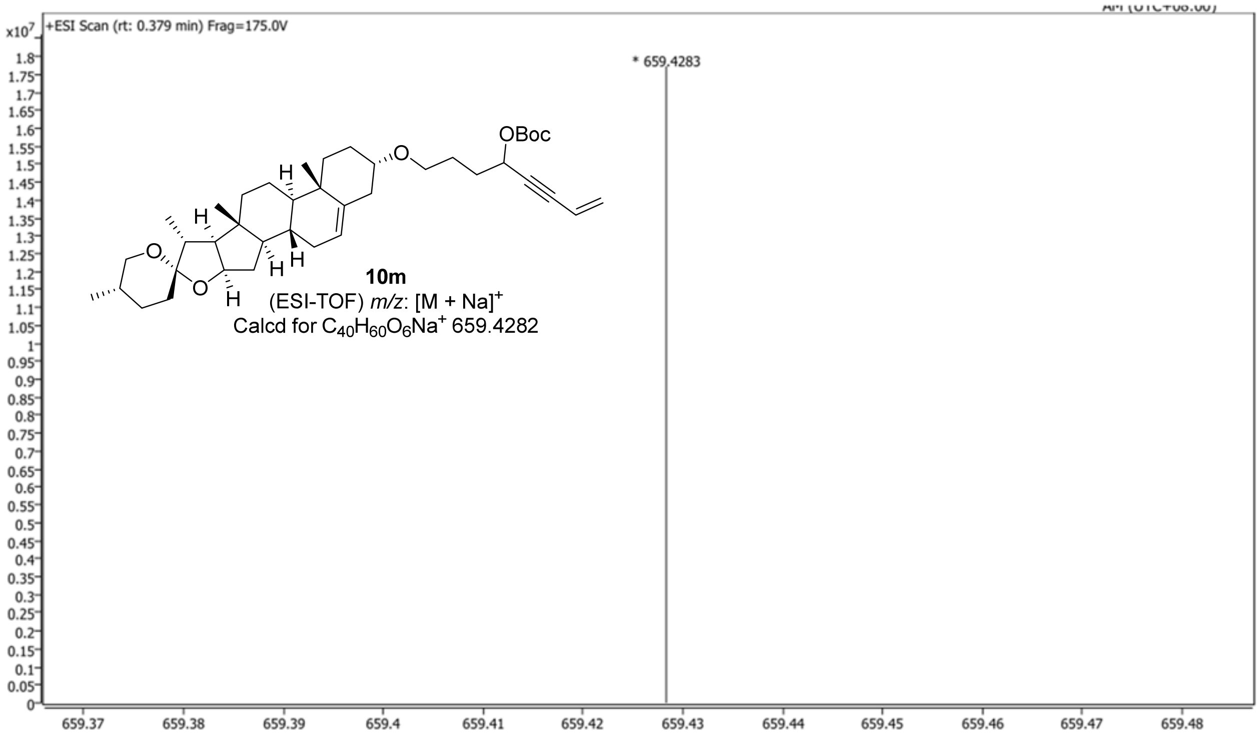
+ESI Scan (rt: 0.379 min) Frag=175.0V
x10^7
* 659.4283
OBoc
10m
(ESI-TOF) m/z: [M + Na]+
Calcd for C40H60O6Na+ 659.4282

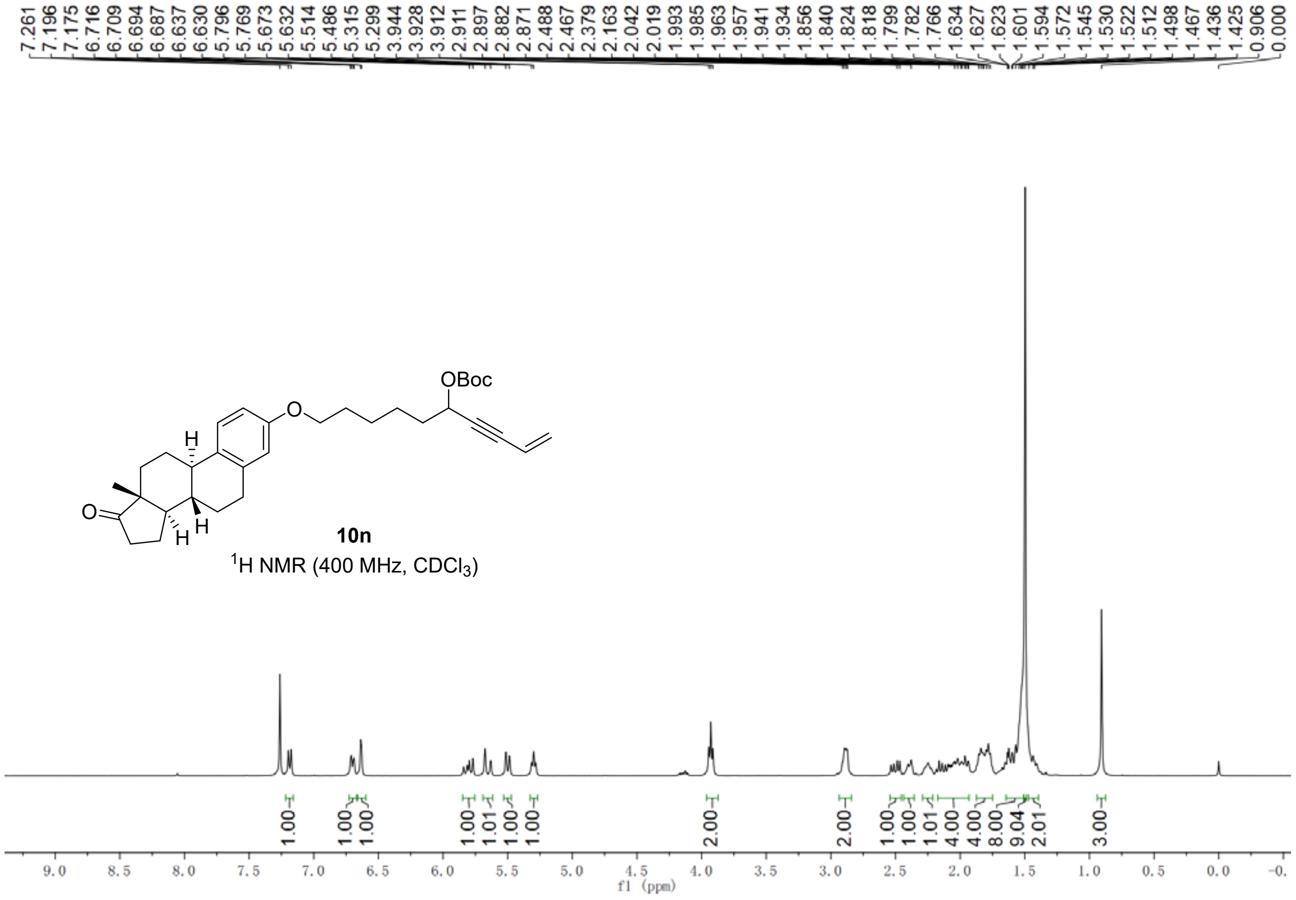
7.261 7.196 7.175 6.716 6.709 6.694 6.687 6.637 6.630 5.796 5.769 5.673 5.632 5.514 5.486 5.315 5.299 3.944 3.928 3.912 2.911 2.897 2.882 2.871 2.488 2.467 2.379 2.163 2.042 2.019 1.993 1.985 1.963 1.957 1.941 1.934 1.856 1.840 1.824 1.818 1.799 1.782 1.766 1.634 1.627 1.623 1.601 1.594 1.572 1.545 1.530 1.522 1.512 1.498 1.467 1.436 1.425 0.906 0.000
OBoc
O
H
H
H
O
10n
^{1}H NMR (400 MHz, CDCl$_3$)
1.00 1.00 1.00 1.00 1.01 1.00 1.00 2.00 2.00 1.00 1.00 1.01 4.00 4.00 8.00 9.04 2.01 3.00
f1 (ppm)

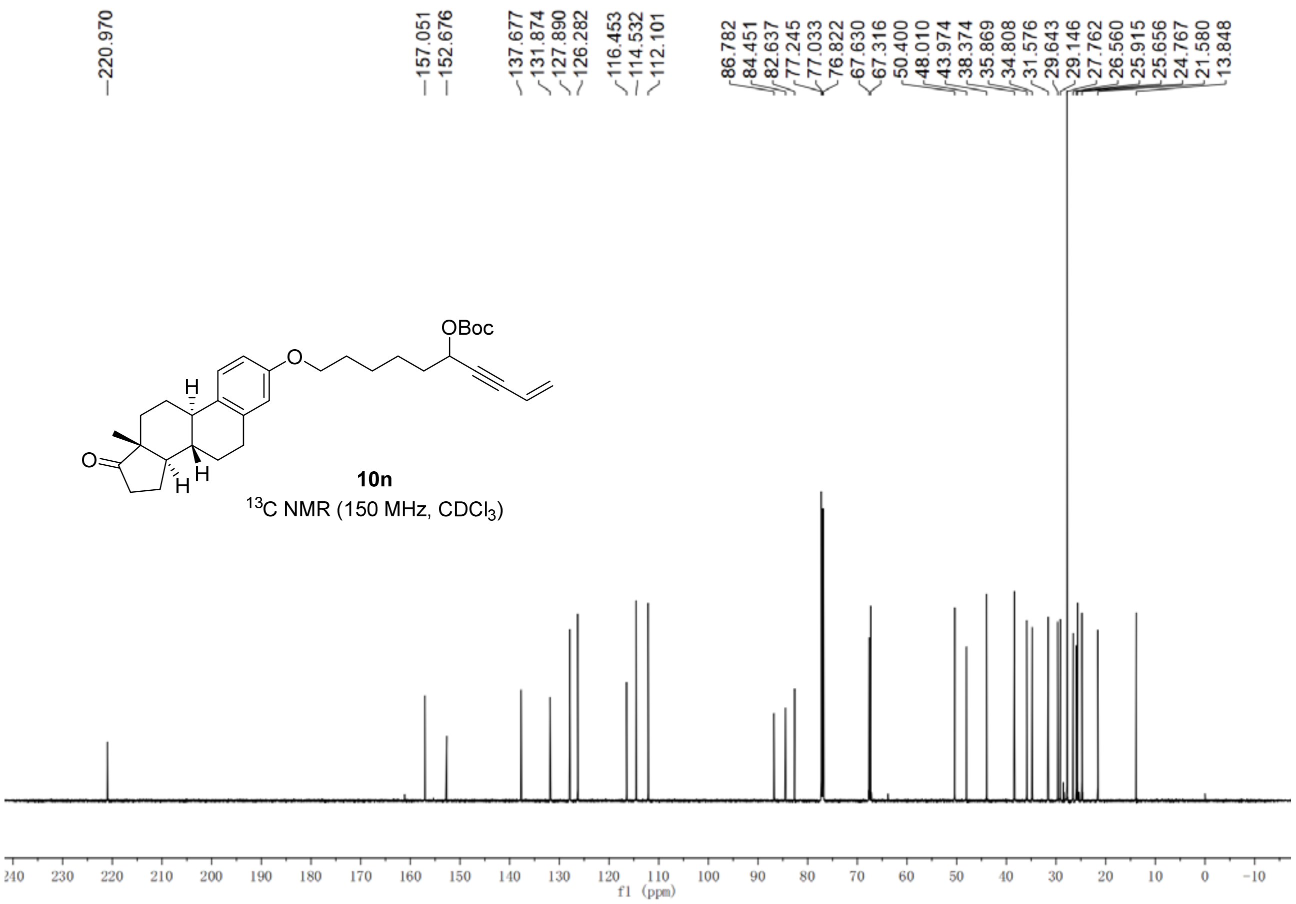
220.970 157.051 152.676 137.677 131.874 127.890 126.282 116.453 114.532 112.101 86.782 84.451 82.637 77.245 77.033 76.822 67.630 67.316 50.400 48.010 43.974 38.374 35.869 34.808 31.576 29.643 29.146 27.762 26.560 25.915 25.656 24.767 21.580 13.848
OBoc
O
H
H
H
O
10n
^{13}C NMR (150 MHz, CDCl$_3$)
f1 (ppm)

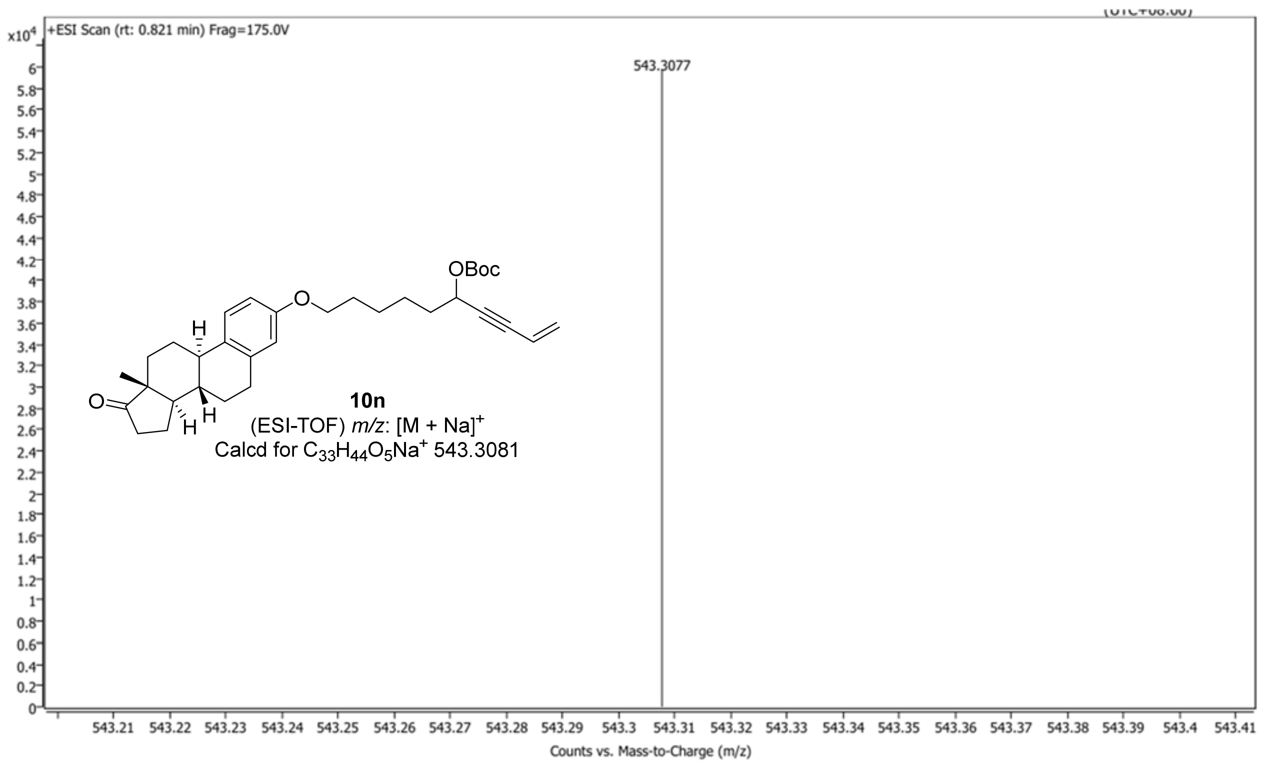

(UTC+08:00)
x10^4
+ESI Scan (rt: 0.821 min) Frag=175.0V
543.3077
OBoc
O
H
H
H
O
10n
(ESI-TOF) m/z: [M + Na]+
Calcd for C33H44O5Na+ 543.3081
Counts vs. Mass-to-Charge (m/z)

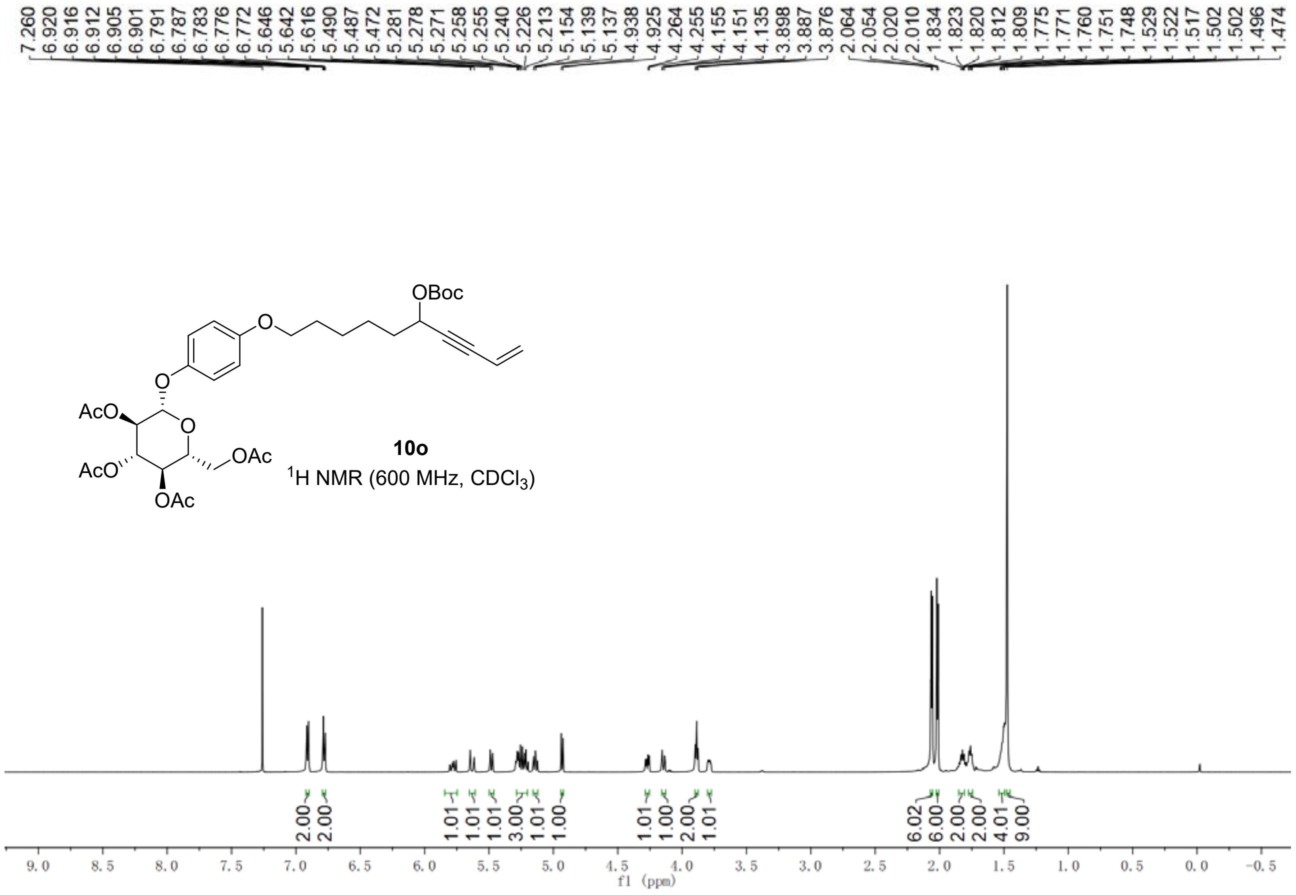
7.260 6.920 6.916 6.912 6.905 6.901 6.791 6.787 6.783 6.776 6.772 5.646 5.642 5.616 5.490 5.487 5.472 5.281 5.278 5.271 5.258 5.255 5.240 5.226 5.213 5.154 5.139 5.137 4.938 4.925 4.264 4.255 4.155 4.151 4.135 3.898 3.887 3.876 2.064 2.054 2.020 2.010 1.834 1.823 1.820 1.812 1.809 1.775 1.771 1.760 1.751 1.748 1.529 1.522 1.517 1.502 1.496 1.474
OBoc
O
O
AcO
O
OAc
AcO
OAc
10o
^{1}H NMR (600 MHz, CDCl$_3$)
2.00 2.00 1.01 1.01 1.01 3.00 1.01 1.00 1.01 1.00 2.00 1.01 6.02 6.00 2.00 2.00 4.01 9.00
f1 (ppm)

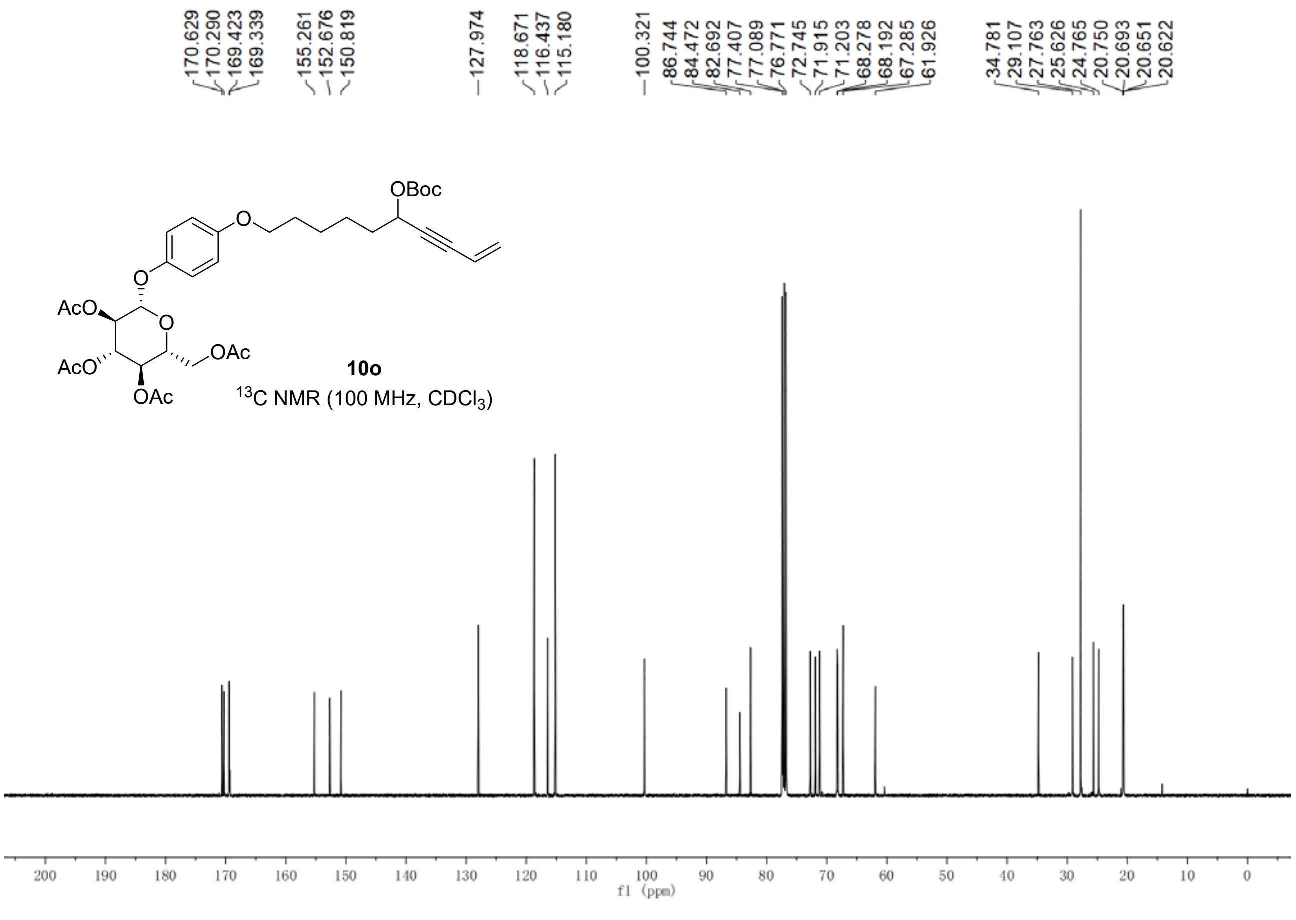
170.629 170.290 169.423 169.339 155.261 152.676 150.819 127.974 118.671 116.437 115.180 100.321 86.744 84.472 82.692 77.407 77.089 76.771 72.745 71.915 71.203 68.278 68.192 67.285 61.926 34.781 29.107 27.763 25.626 24.765 20.750 20.693 20.651 20.622
OBoc
O
O
AcO
O
OAc
AcO
OAc
10o
^{13}C NMR (100 MHz, CDCl$_3$)
f1 (ppm)

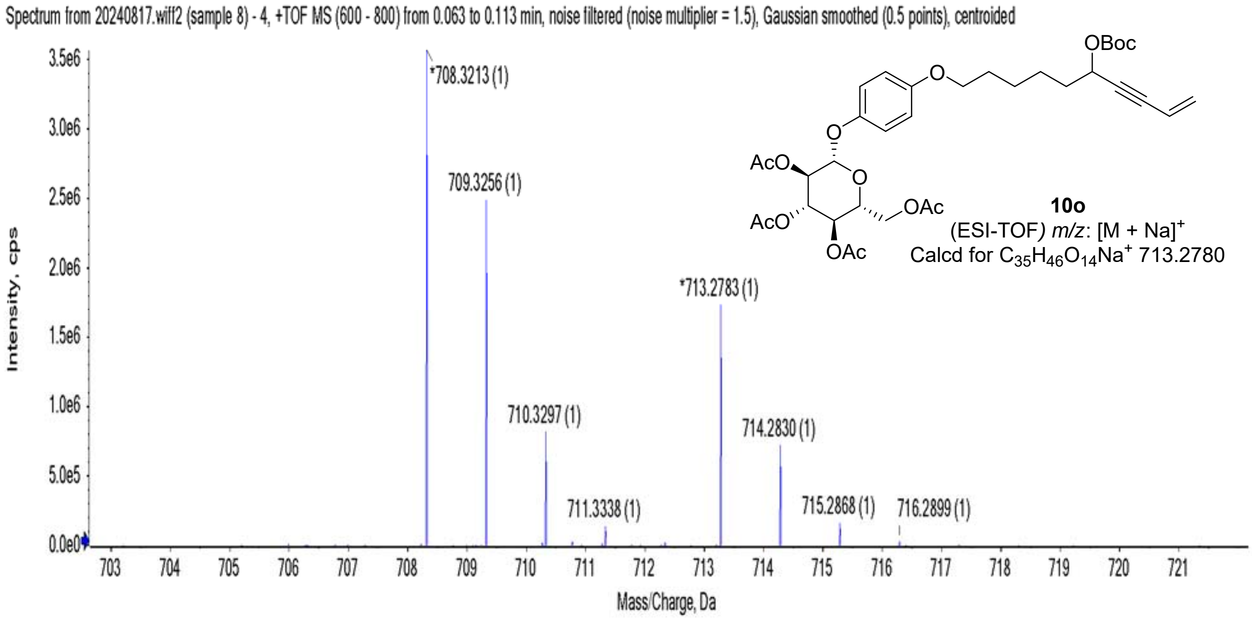
Spectrum from 20240817.wiff2 (sample 8) - 4, +TOF MS (600 - 800) from 0.063 to 0.113 min, noise filtered (noise multiplier = 1.5), Gaussian smoothed (0.5 points), centroided
Intensity, cps
Mass/Charge, Da
*708.3213 (1)
709.3256 (1)
710.3297 (1)
711.3338 (1)
*713.2783 (1)
714.2830 (1)
715.2868 (1)
716.2899 (1)
OBoc
AcO
AcO
OAc
OAc
10o
(ESI-TOF) m/z: [M + Na]+
Calcd for C35H46O14Na+ 713.2780

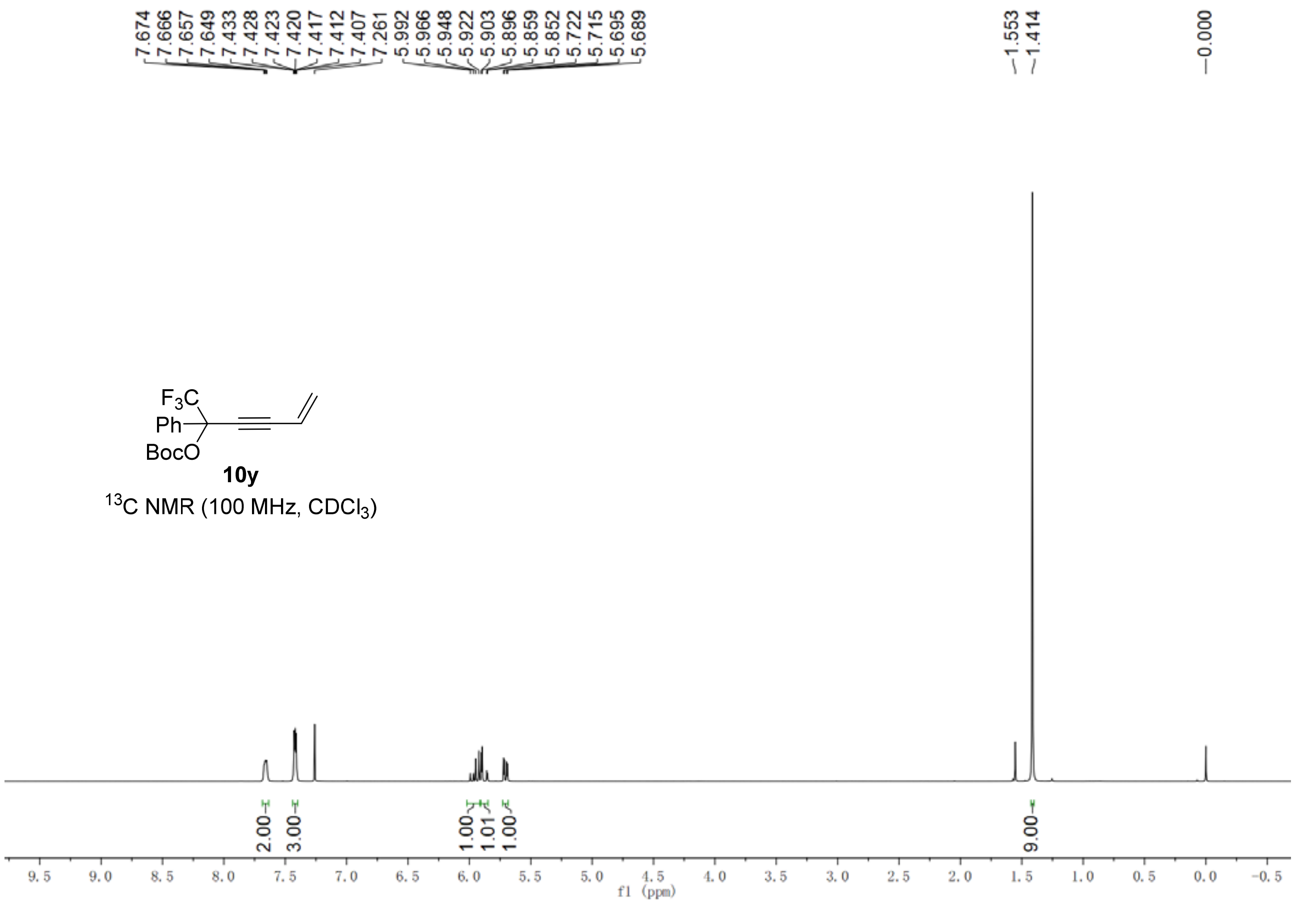
7.674
7.666
7.657
7.649
7.433
7.428
7.423
7.420
7.417
7.412
7.407
7.261
5.992
5.966
5.948
5.922
5.903
5.896
5.859
5.852
5.722
5.715
5.695
5.689
1.553
1.414
0.000
F_3C
Ph
BocO
10y
^{13}C NMR (100 MHz, $CDCl_3$)
2.00
3.00
1.00
1.01
1.00
9.00
9.5 9.0 8.5 8.0 7.5 7.0 6.5 6.0 5.5 5.0 4.5 4.0 3.5 3.0 2.5 2.0 1.5 1.0 0.5 0.0 -0.5
f1 (ppm)

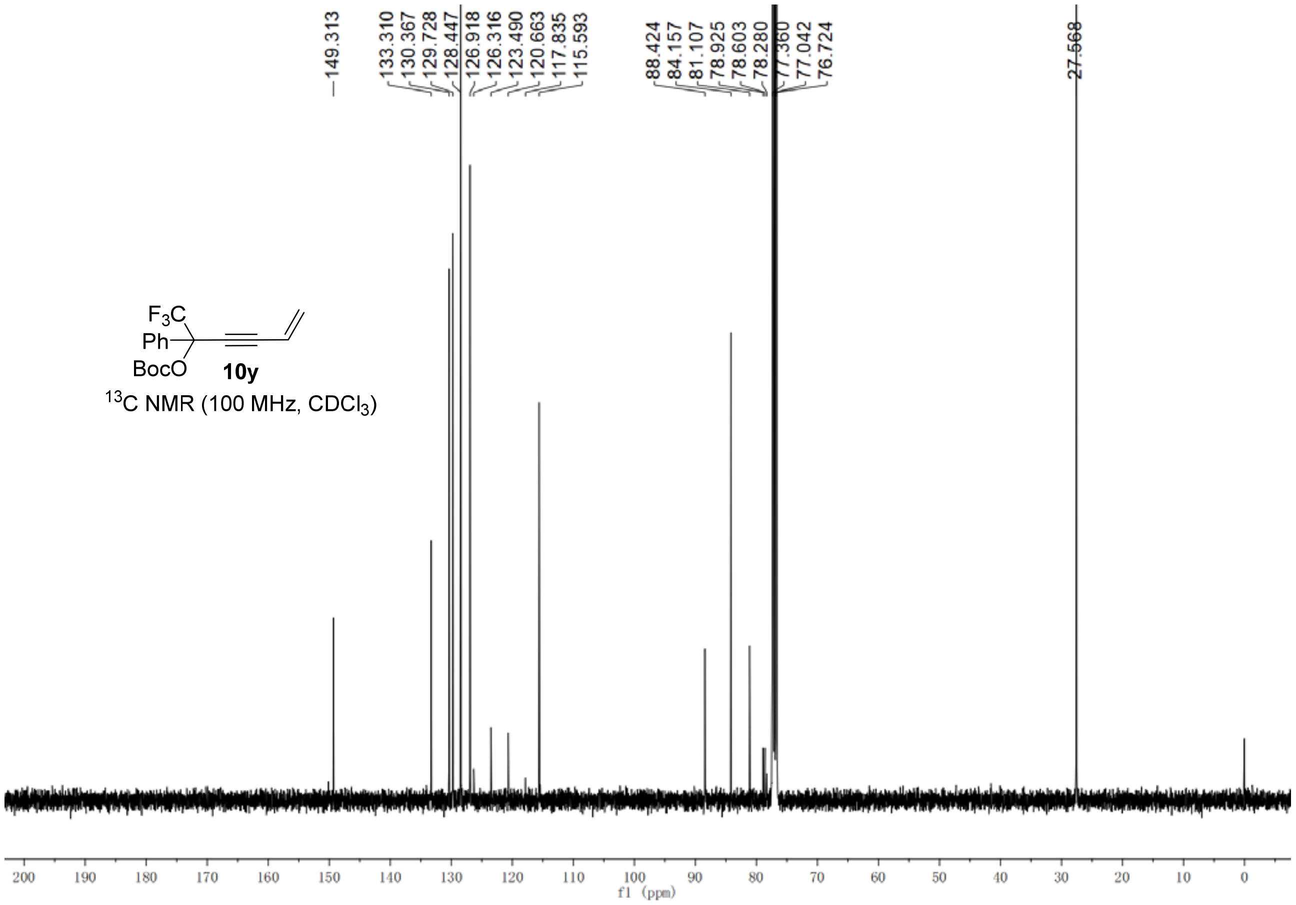
149.313
133.310
130.367
129.728
128.447
126.918
126.316
123.490
120.663
117.835
115.593
88.424
84.157
81.107
78.925
78.603
78.280
77.360
77.042
76.724
27.568
F_3C
Ph
BocO
10y
^{13}C NMR (100 MHz, $CDCl_3$)
200 190 180 170 160 150 140 130 120 110 100 90 80 70 60 50 40 30 20 10 0
f1 (ppm)

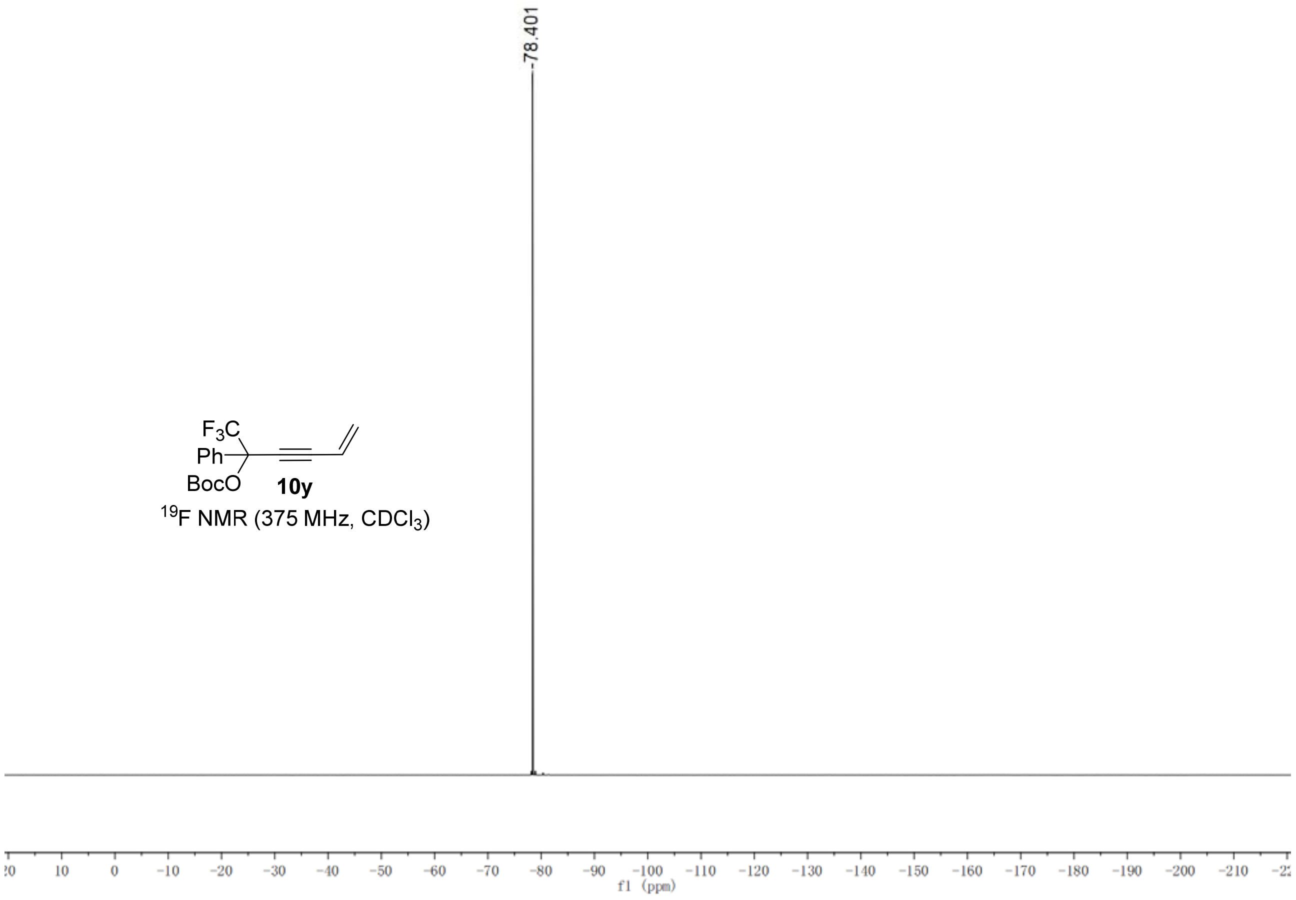
-78.401
F3C
Ph
BocO
10y
19F NMR (375 MHz, CDCl3)
10 0 -10 -20 -30 -40 -50 -60 -70 -80 -90 -100 -110 -120 -130 -140 -150 -160 -170 -180 -190 -200 -210
f1 (ppm)

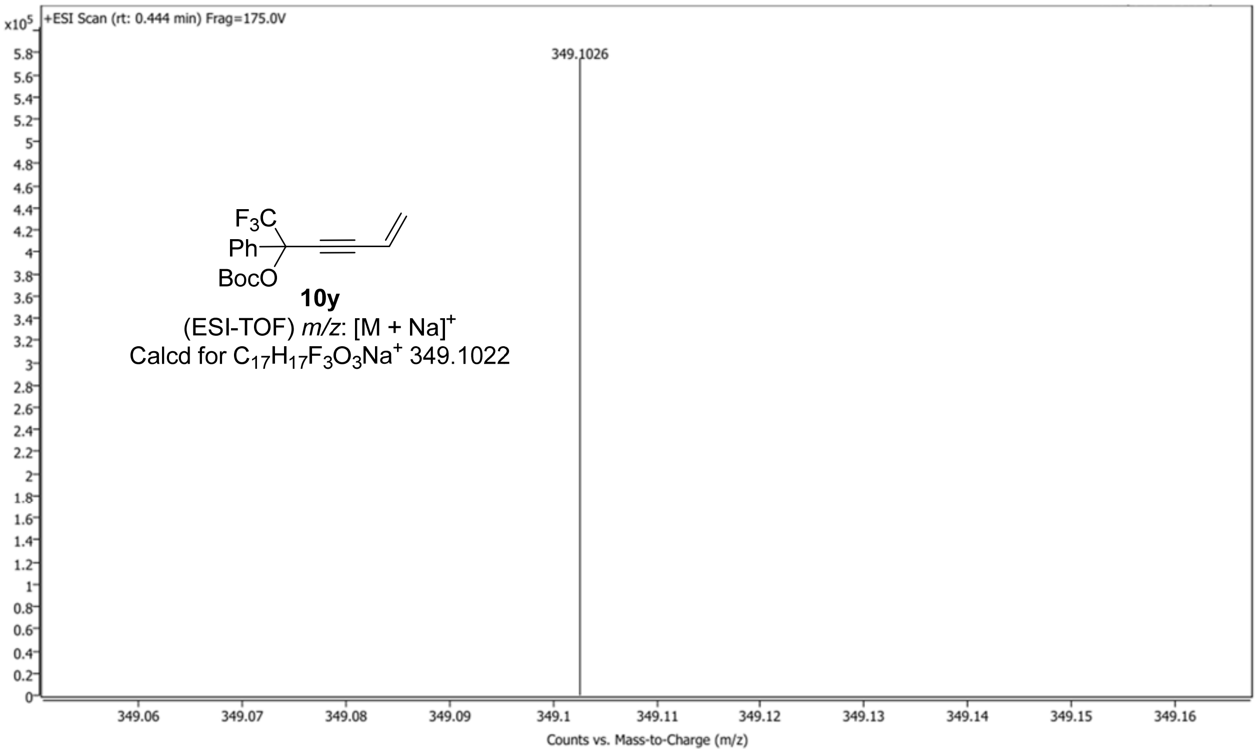
x10^5
+ESI Scan (rt: 0.444 min) Frag=175.0V
349.1026
F3C
Ph
BocO
10y
(ESI-TOF) m/z: [M + Na]+
Calcd for C17H17F3O3Na+ 349.1022
349.06 349.07 349.08 349.09 349.1 349.11 349.12 349.13 349.14 349.15 349.16
Counts vs. Mass-to-Charge (m/z)

2m

^{1}H NMR (400 MHz, DMSO-d_6)

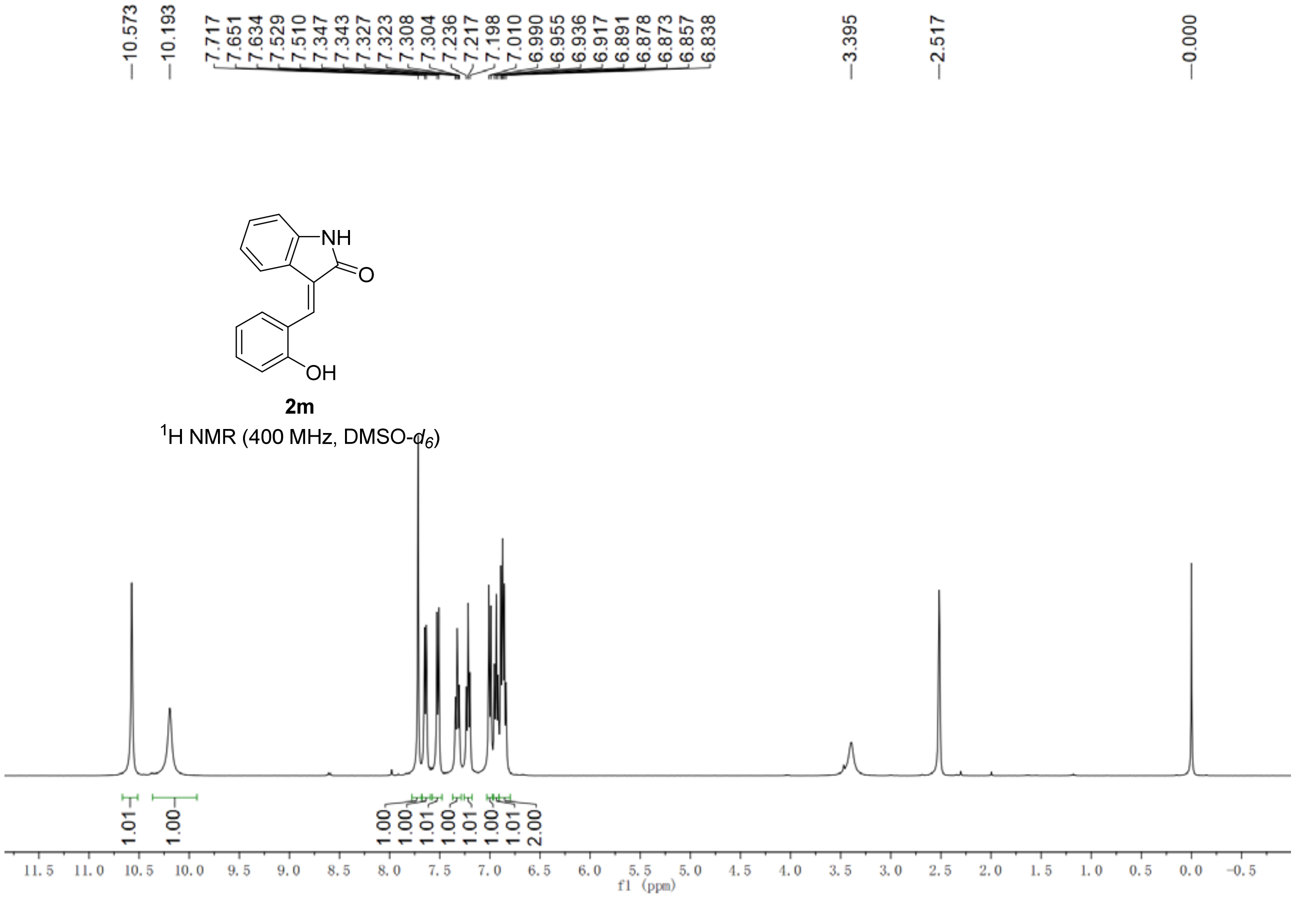

2m

^{13}C NMR (100 MHz, DMSO-d_6)

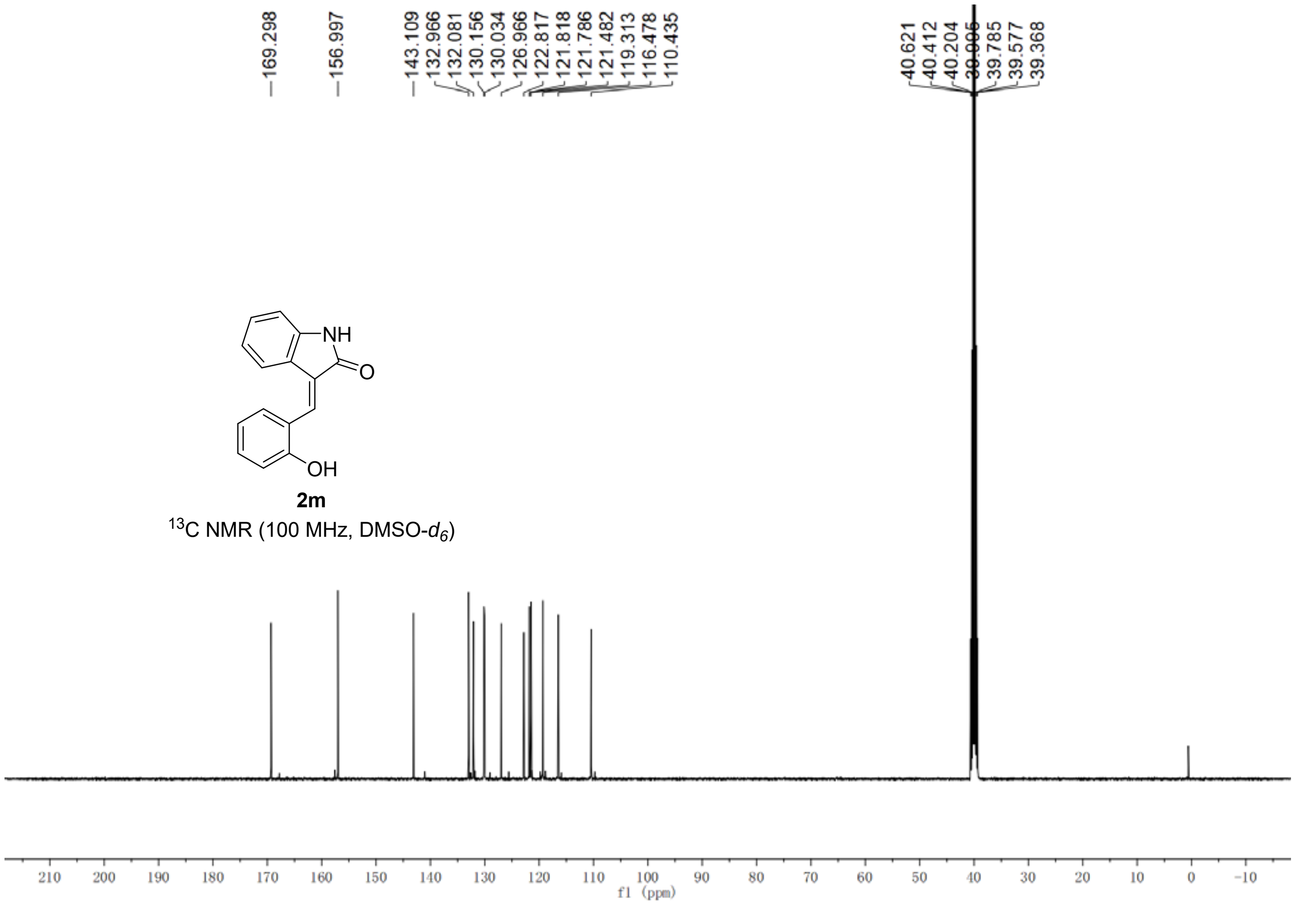

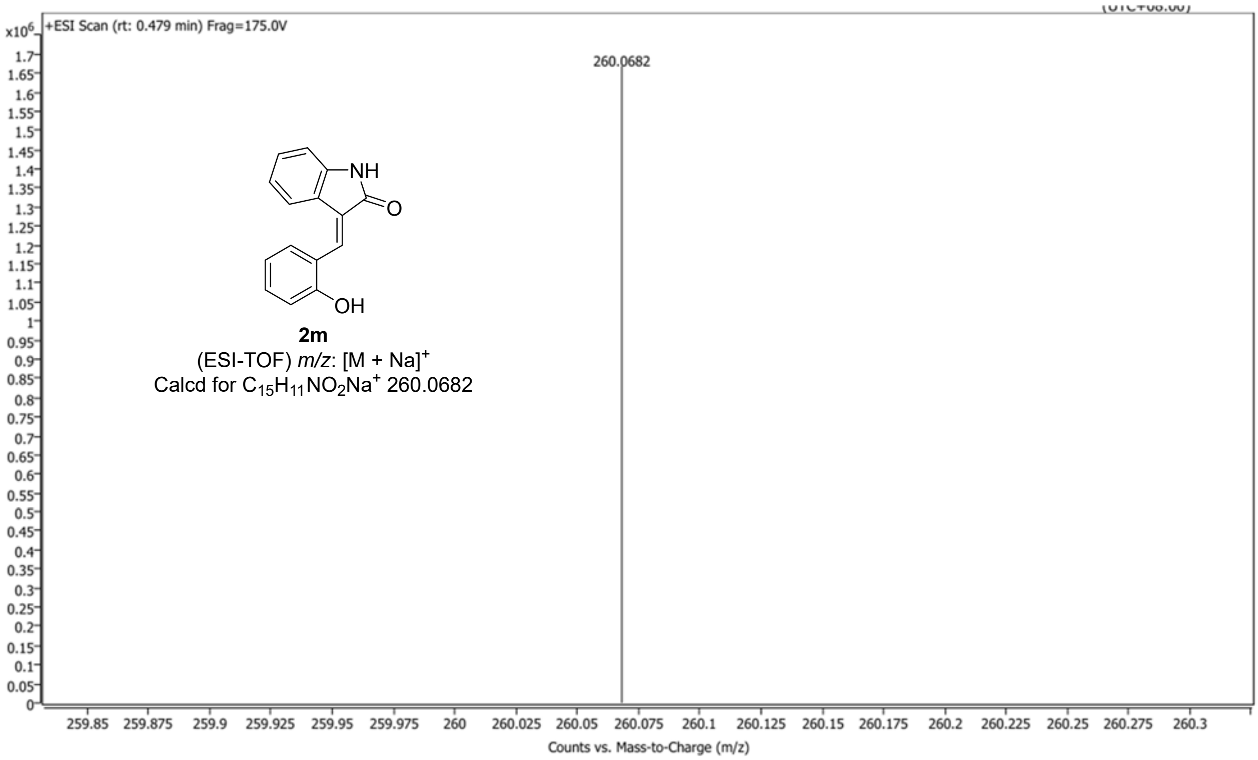
+ESI Scan (rt: 0.479 min) Frag=175.0V
x10^6
260.0682
NH
O
OH
2m
(ESI-TOF) m/z: [M + Na]+
Calcd for C15H11NO2Na+ 260.0682
Counts vs. Mass-to-Charge (m/z)

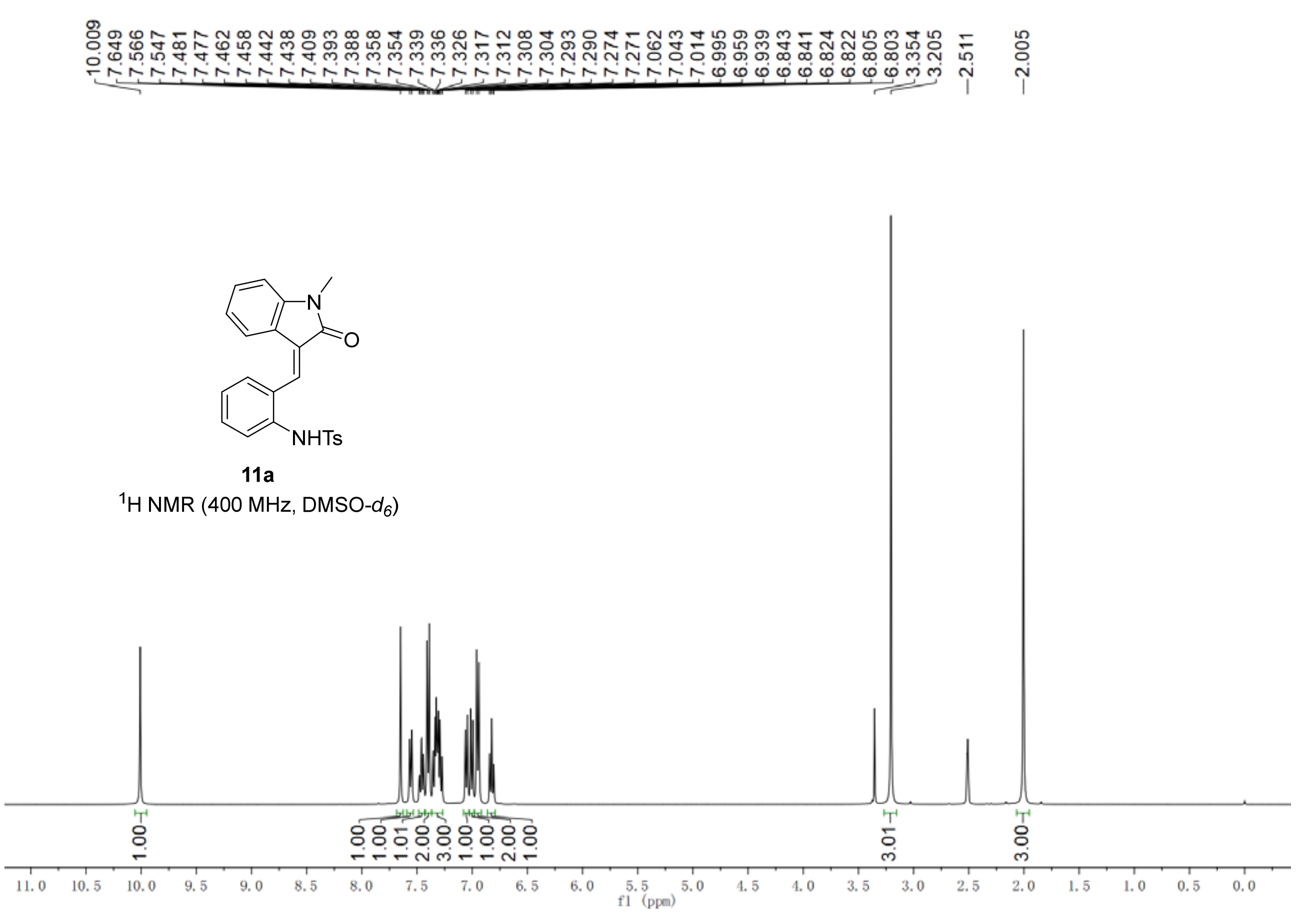

11a

^{1}H NMR (400 MHz, DMSO-d_6)

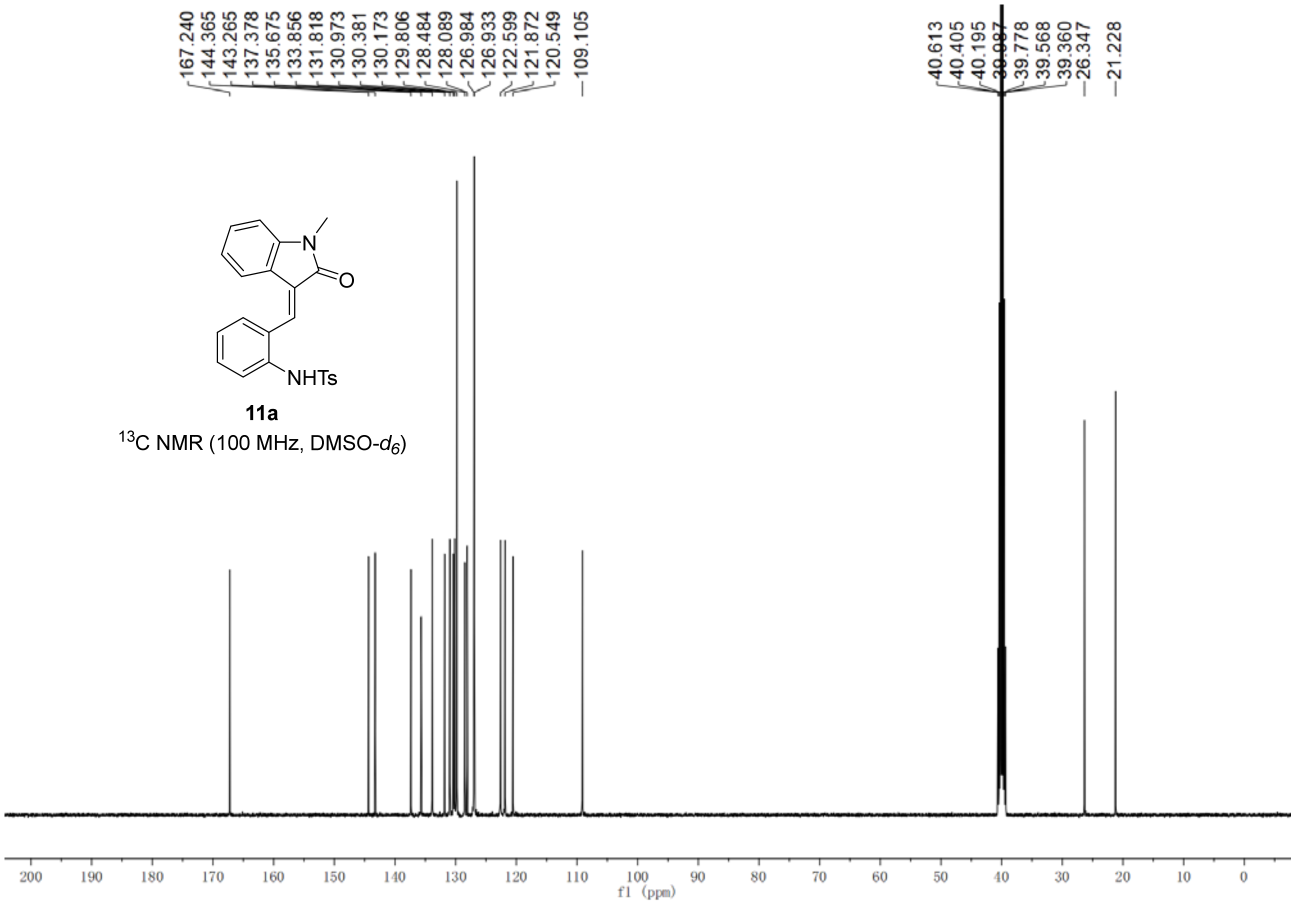

11a

^{13}C NMR (100 MHz, DMSO-d_6)

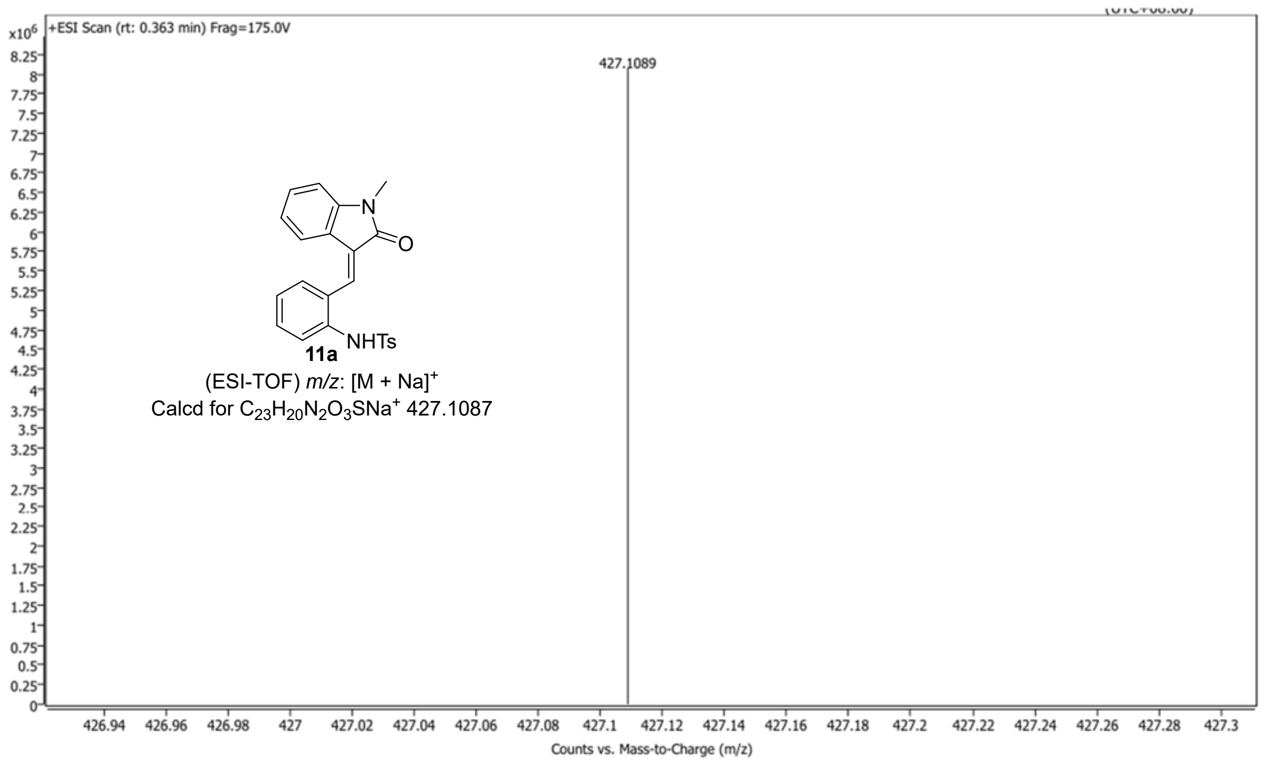
x10^6
+ESI Scan (rt: 0.363 min) Frag=175.0V
427.1089
NHTs
11a
(ESI-TOF) m/z: [M + Na]+
Calcd for C23H20N2O3SNa+ 427.1087
Counts vs. Mass-to-Charge (m/z)

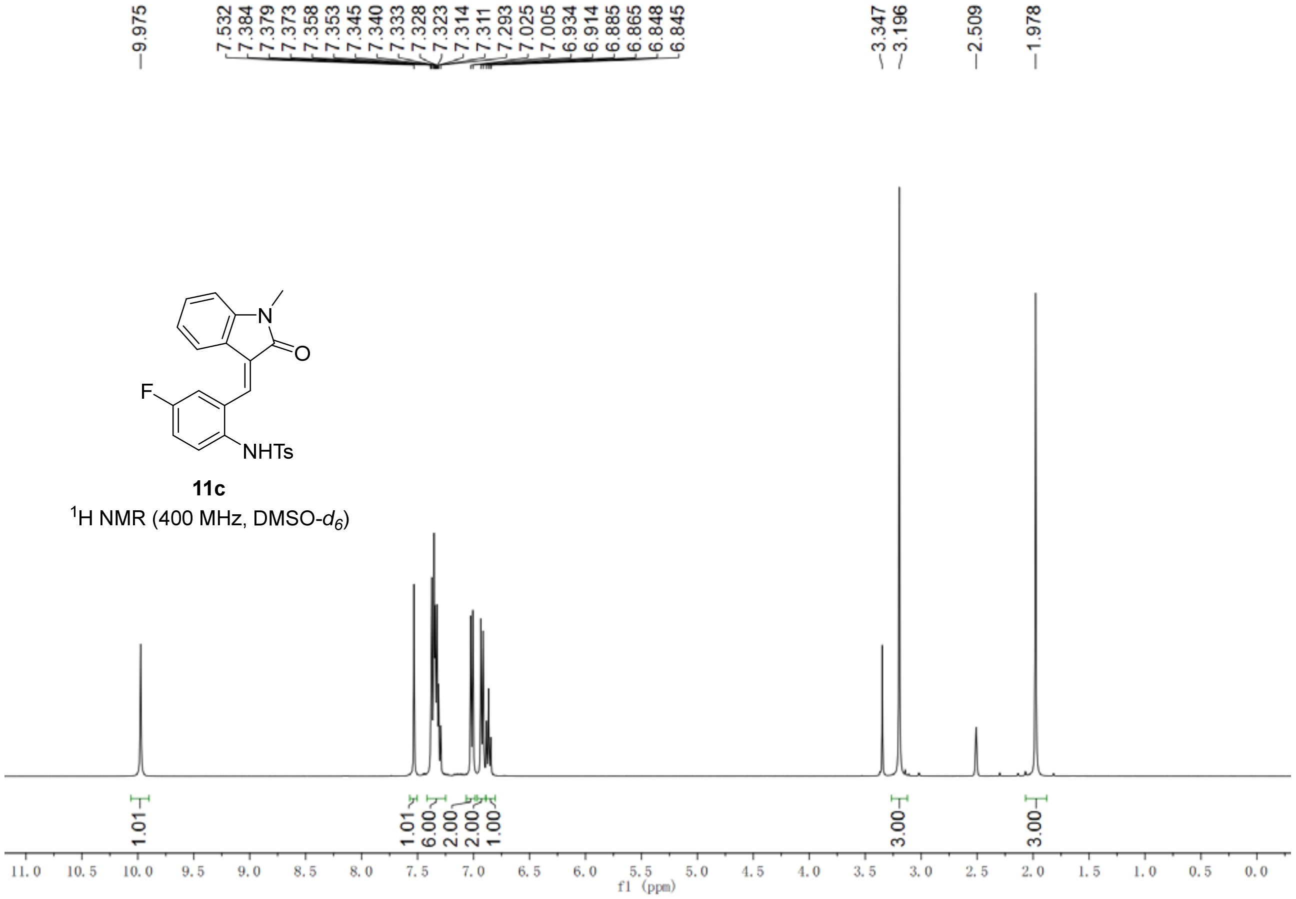

11c

^{1}H NMR (400 MHz, DMSO-d_6)

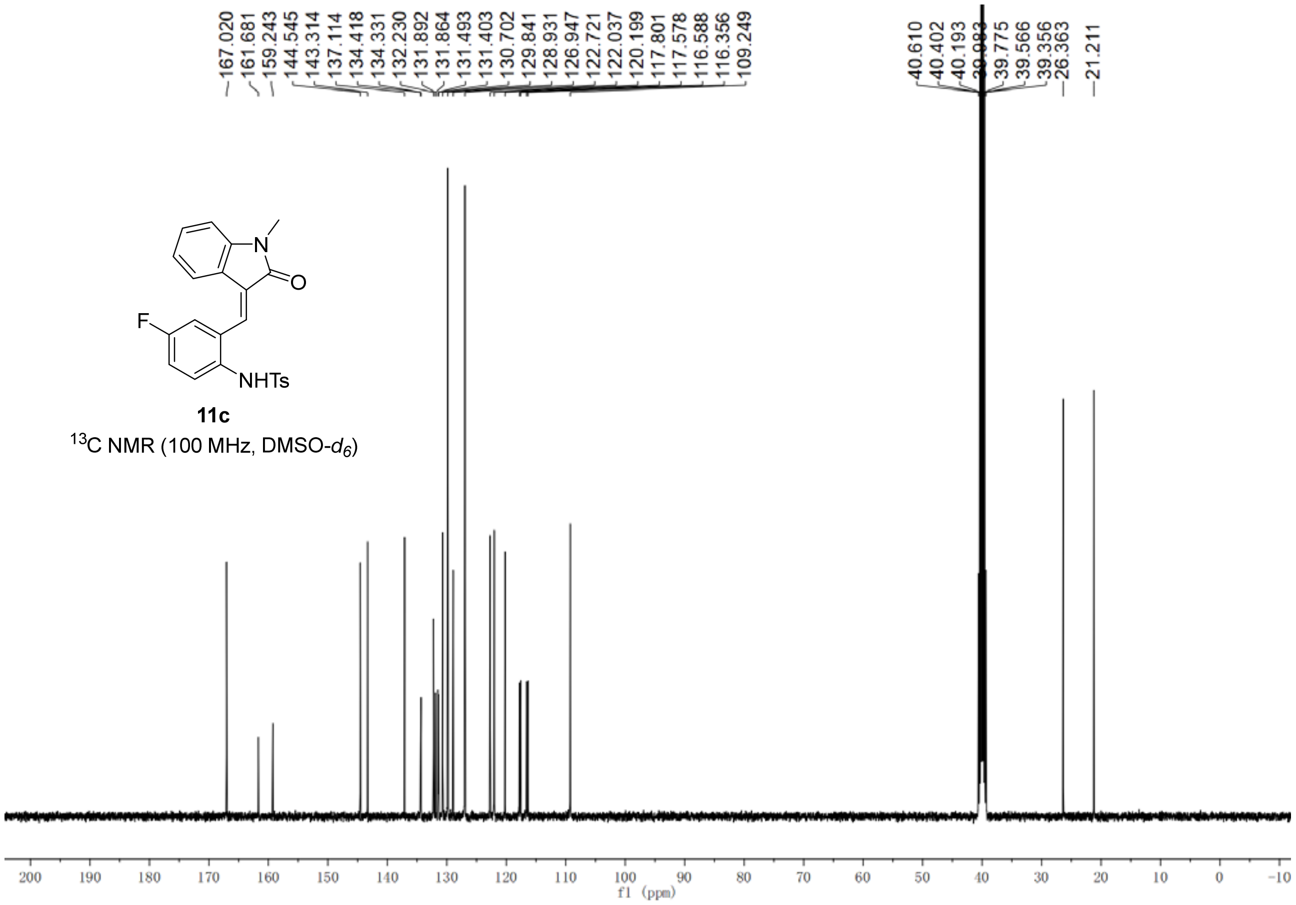

11c

^{13}C NMR (100 MHz, DMSO-d_6)

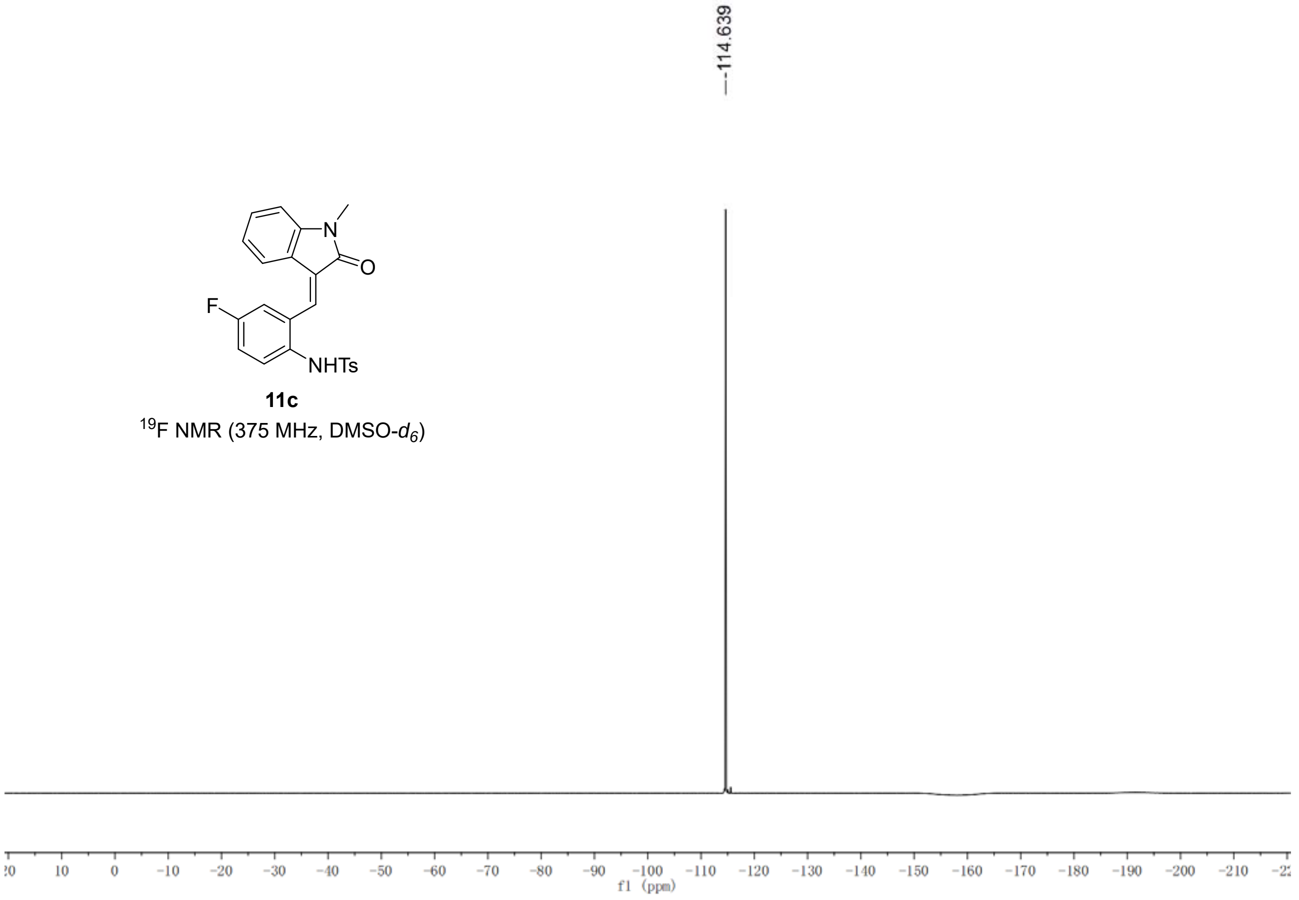
-114.639
F
N
O
NHTs
11c
19F NMR (375 MHz, DMSO-d6)
f1 (ppm)

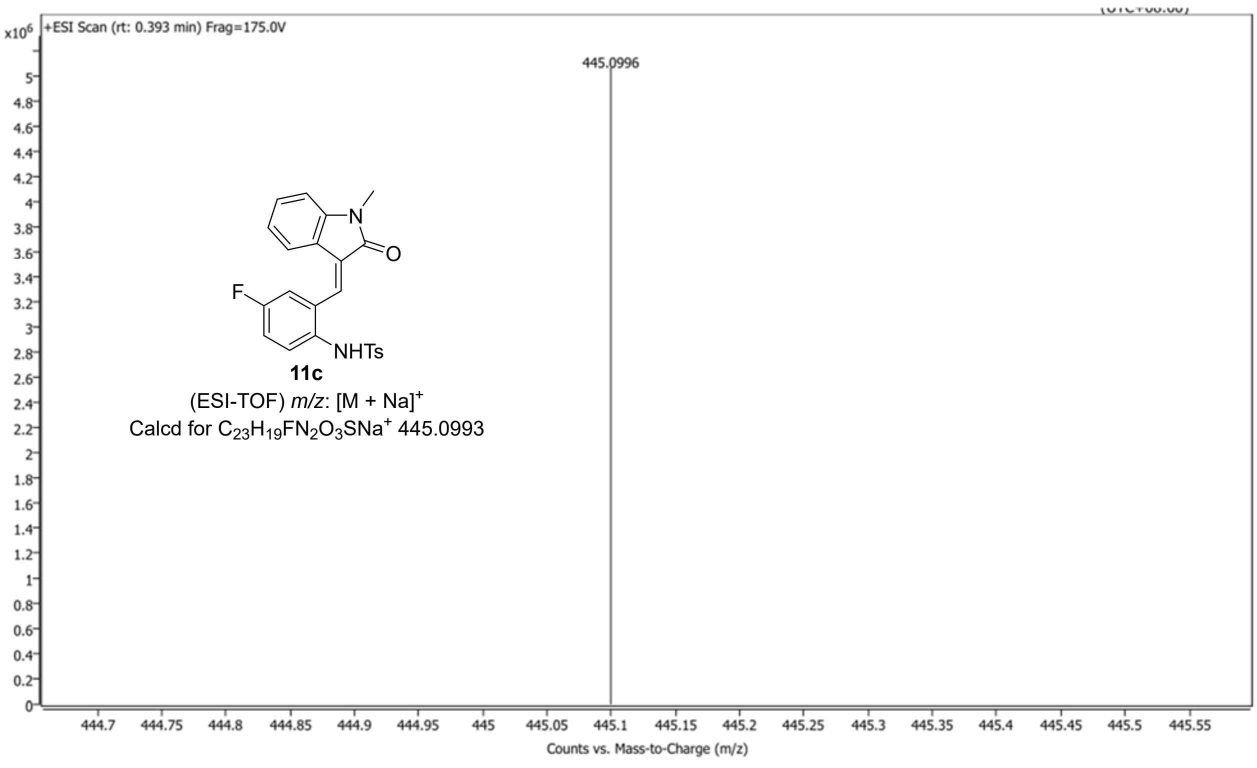
+ESI Scan (rt: 0.393 min) Frag=175.0V
445.0996
F
N
O
NHTs
11c
(ESI-TOF) m/z: [M + Na]+
Calcd for C23H19FN2O3SNa+ 445.0993
Counts vs. Mass-to-Charge (m/z)

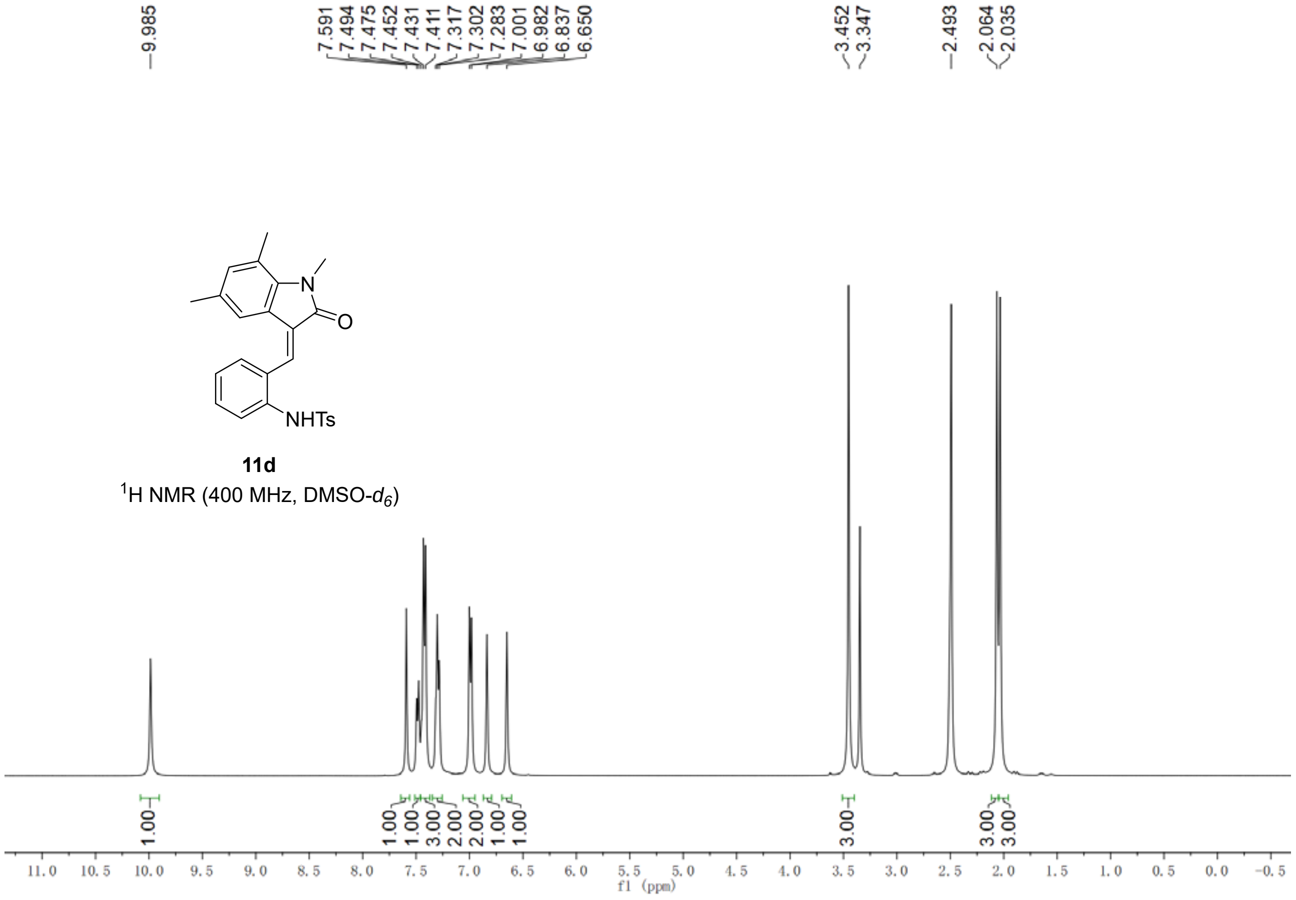
9.985
7.591
7.494
7.475
7.452
7.431
7.411
7.317
7.302
7.283
7.001
6.982
6.837
6.650
3.452
3.347
2.493
2.064
2.035
NHTs
11d
^{1}H NMR (400 MHz, DMSO-d_6)
1.00
1.00
1.00
3.00
2.00
1.00
1.00
3.00
3.00
3.00
f1 (ppm)

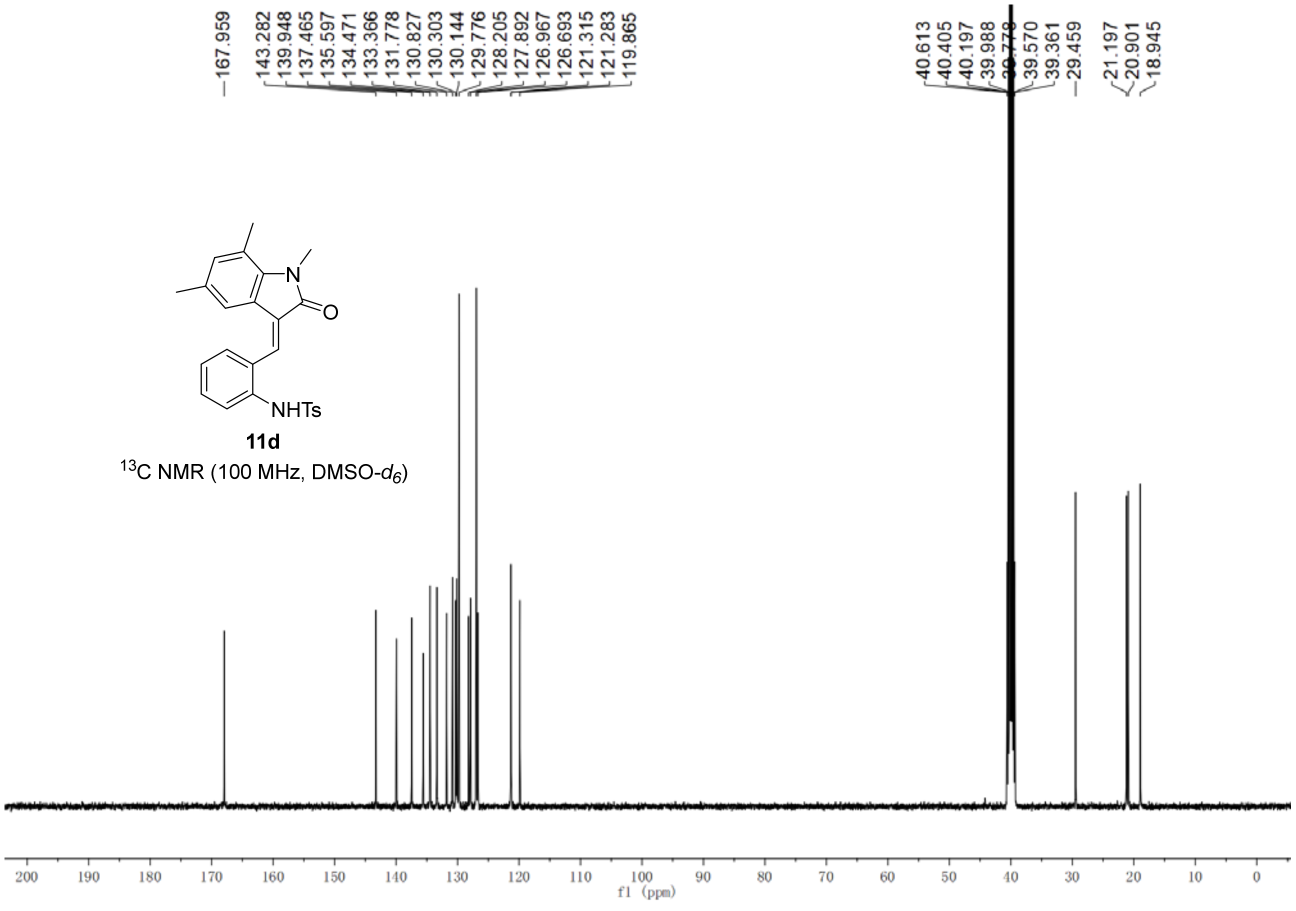
167.959
143.282
139.948
137.465
135.597
134.471
133.366
131.778
130.827
130.303
130.144
129.776
128.205
127.892
126.967
126.693
121.315
121.283
119.865
40.613
40.405
40.197
39.988
39.570
39.361
29.459
21.197
20.901
18.945
NHTs
11d
^{13}C NMR (100 MHz, DMSO-d_6)
f1 (ppm)

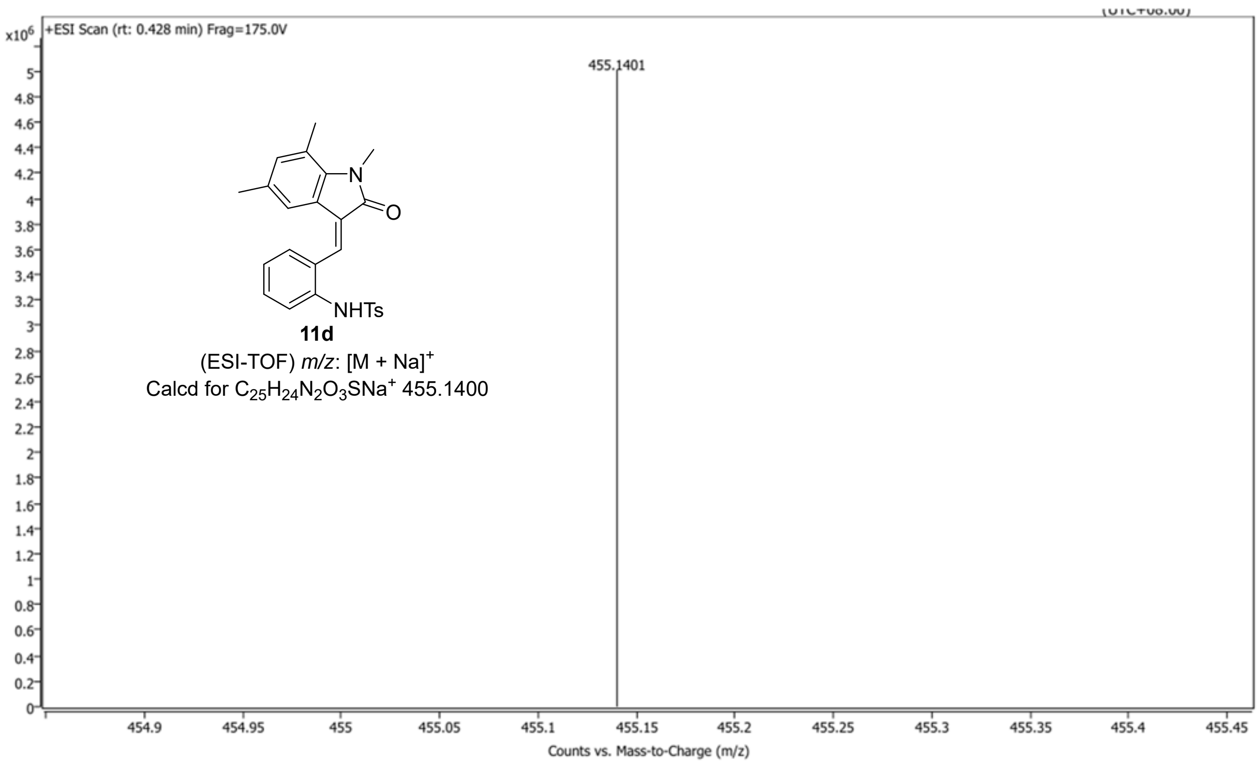
+ESI Scan (rt: 0.428 min) Frag=175.0V
x10^6
455.1401
N
O
NHTs
11d
(ESI-TOF) m/z: [M + Na]+
Calcd for C25H24N2O3SNa+ 455.1400
5
4.8
4.6
4.4
4.2
4
3.8
3.6
3.4
3.2
3
2.8
2.6
2.4
2.2
2
1.8
1.6
1.4
1.2
1
0.8
0.6
0.4
0.2
0
454.9
454.95
455
455.05
455.1
455.15
455.2
455.25
455.3
455.35
455.4
455.45
Counts vs. Mass-to-Charge (m/z)

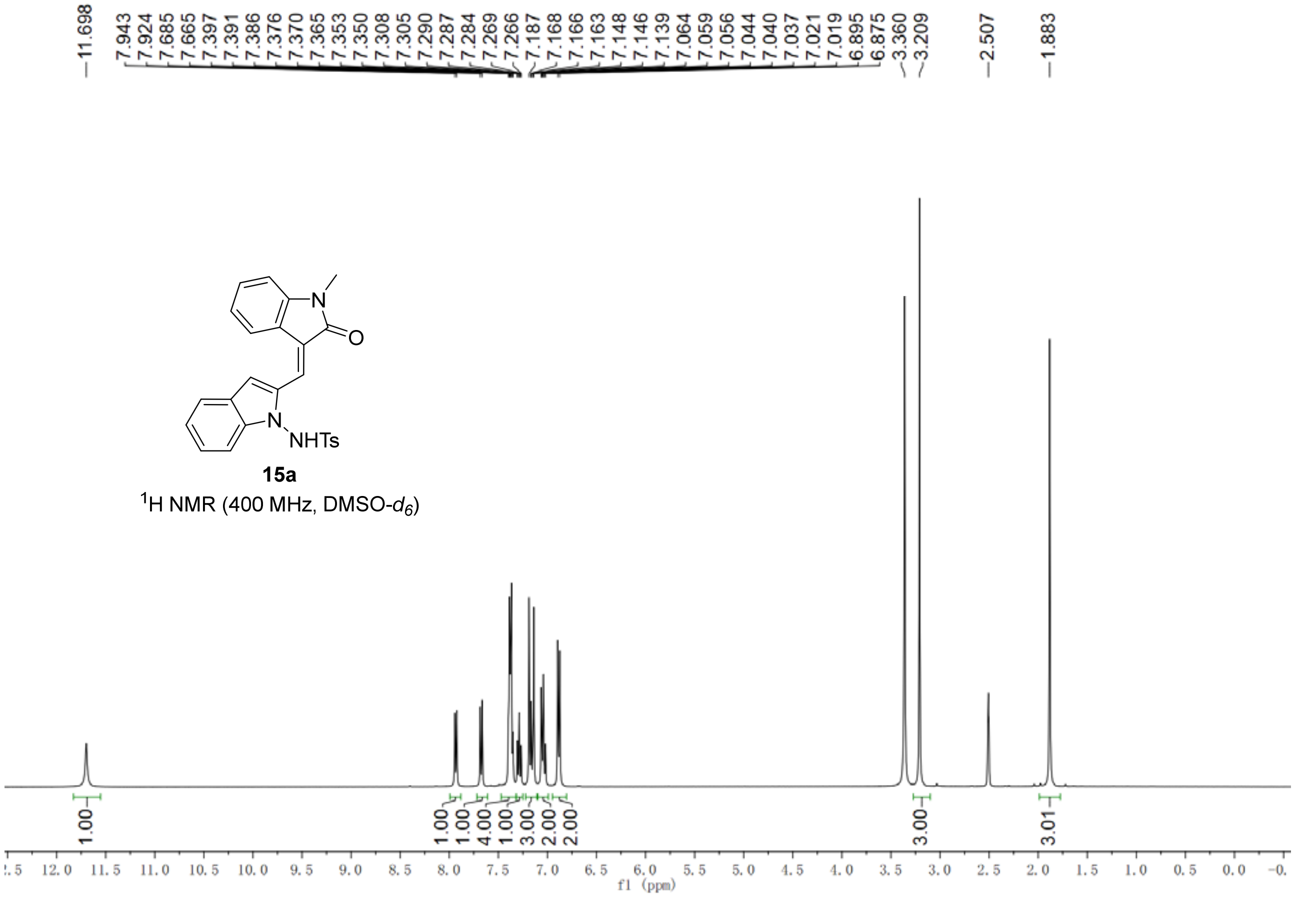

11.698
7.943
7.924
7.685
7.665
7.397
7.391
7.386
7.376
7.370
7.365
7.353
7.350
7.308
7.305
7.290
7.287
7.284
7.269
7.266
7.187
7.168
7.166
7.163
7.148
7.146
7.139
7.064
7.059
7.056
7.044
7.040
7.037
7.021
7.019
6.895
6.875
3.360
3.209
2.507
1.883
NHTs
15a
1H NMR (400 MHz, DMSO-d6)
1.00
1.00
1.00
4.00
1.00
3.00
2.00
2.00
3.00
3.01
f1 (ppm)

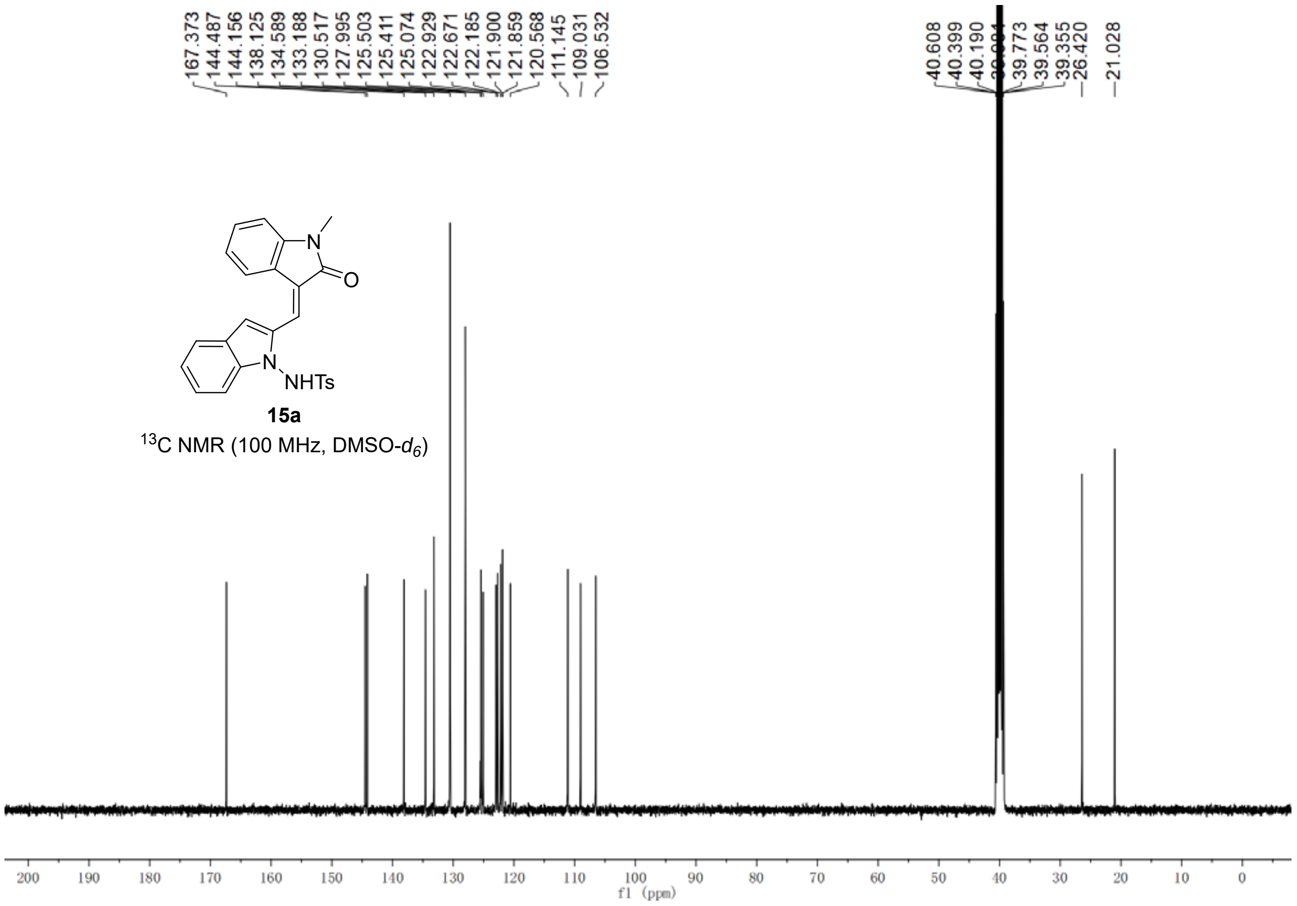

167.373
144.487
144.156
138.125
134.589
133.188
130.517
127.995
125.503
125.411
125.074
122.929
122.671
122.185
121.900
121.859
120.568
111.145
109.031
106.532
40.608
40.399
40.190
39.773
39.564
39.355
26.420
21.028
NHTs
15a
13C NMR (100 MHz, DMSO-d6)
f1 (ppm)

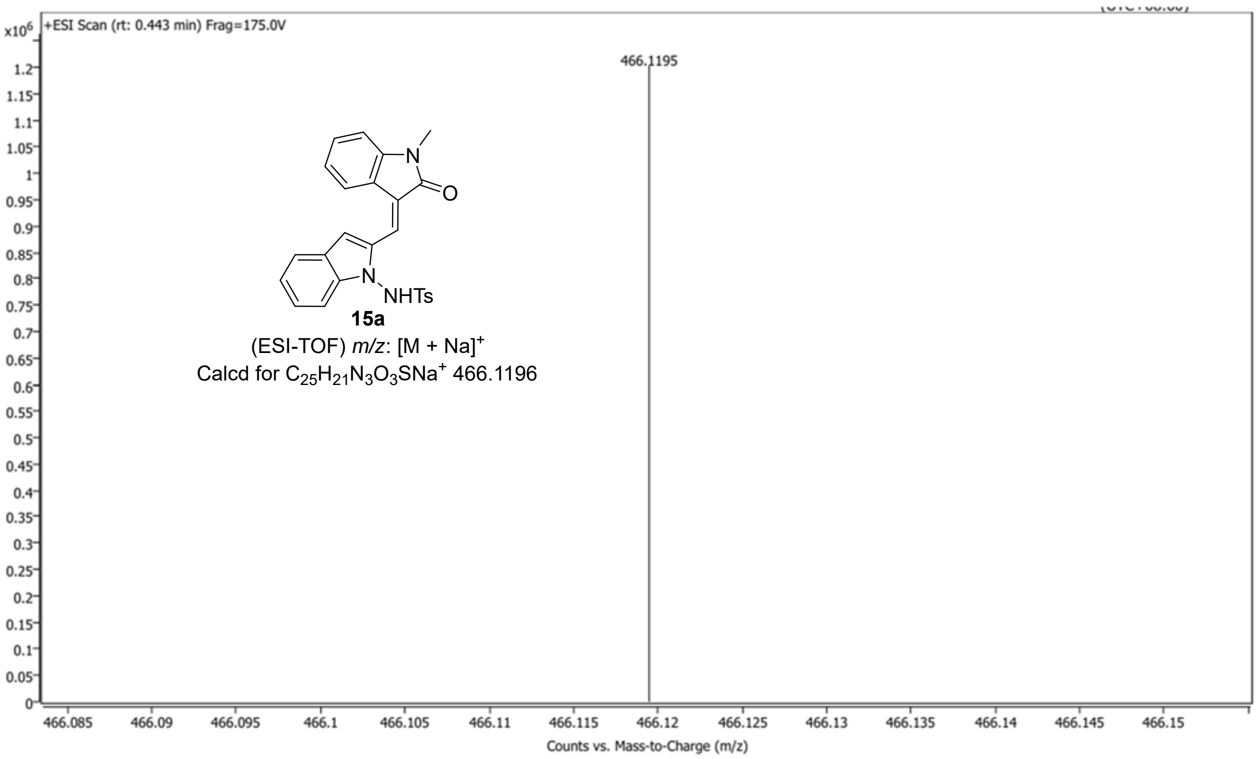
+ESI Scan (rt: 0.443 min) Frag=175.0V
x10^6
466.1195
15a
(ESI-TOF) m/z: [M + Na]+
Calcd for C25H21N3O3SNa+ 466.1196
Counts vs. Mass-to-Charge (m/z)

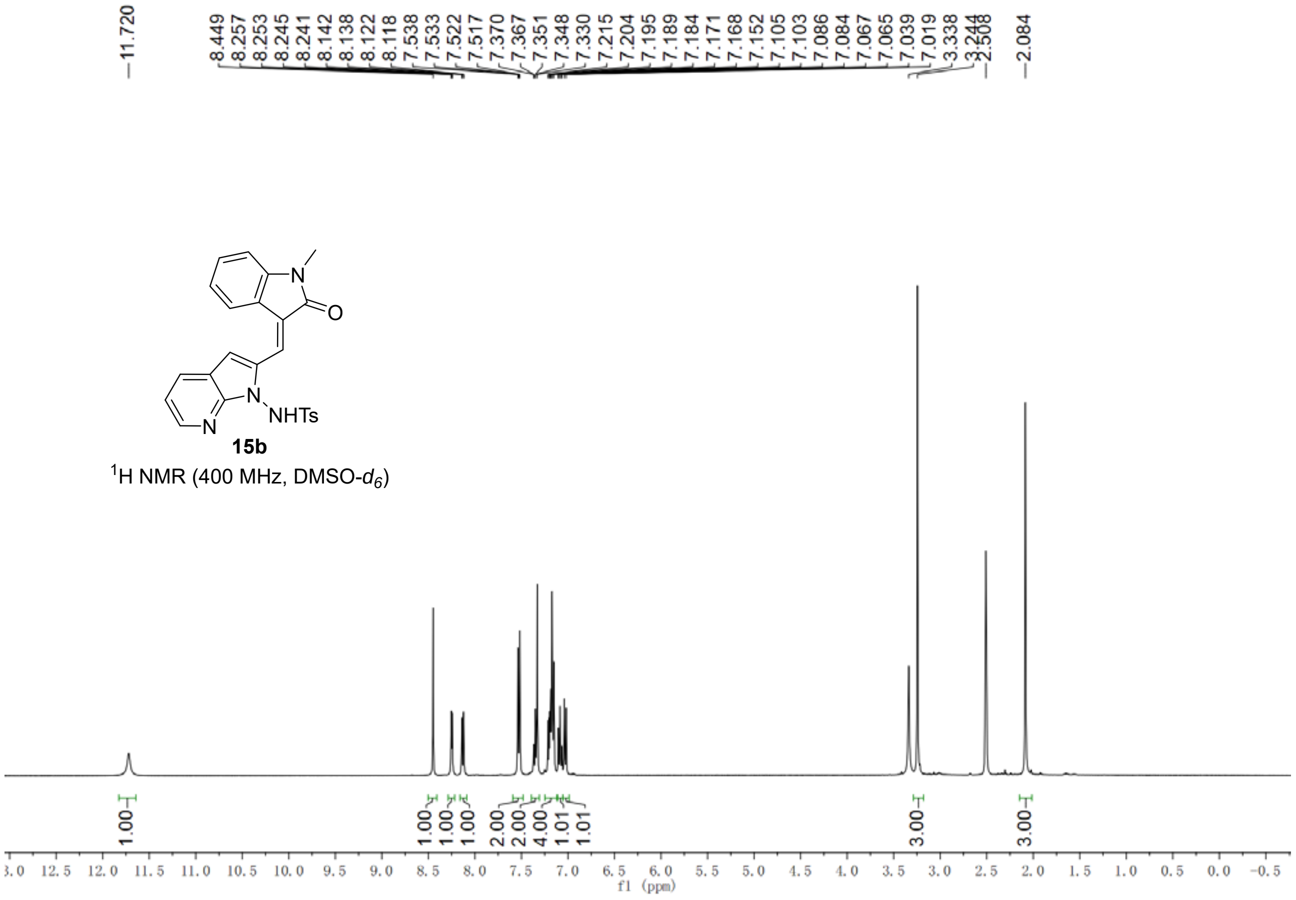
11.720
8.449
8.257
8.253
8.245
8.241
8.142
8.138
8.122
8.118
7.538
7.533
7.522
7.517
7.370
7.367
7.351
7.348
7.330
7.215
7.204
7.195
7.189
7.184
7.171
7.168
7.152
7.105
7.103
7.086
7.084
7.067
7.065
7.039
7.019
3.338
3.244
2.508
2.084
N
O
N
N
NHTs
15b
^{1}H NMR (400 MHz, DMSO-d_6)
1.00
1.00
1.00
1.00
2.00
2.00
4.00
1.01
1.01
3.00
3.00
f1 (ppm)

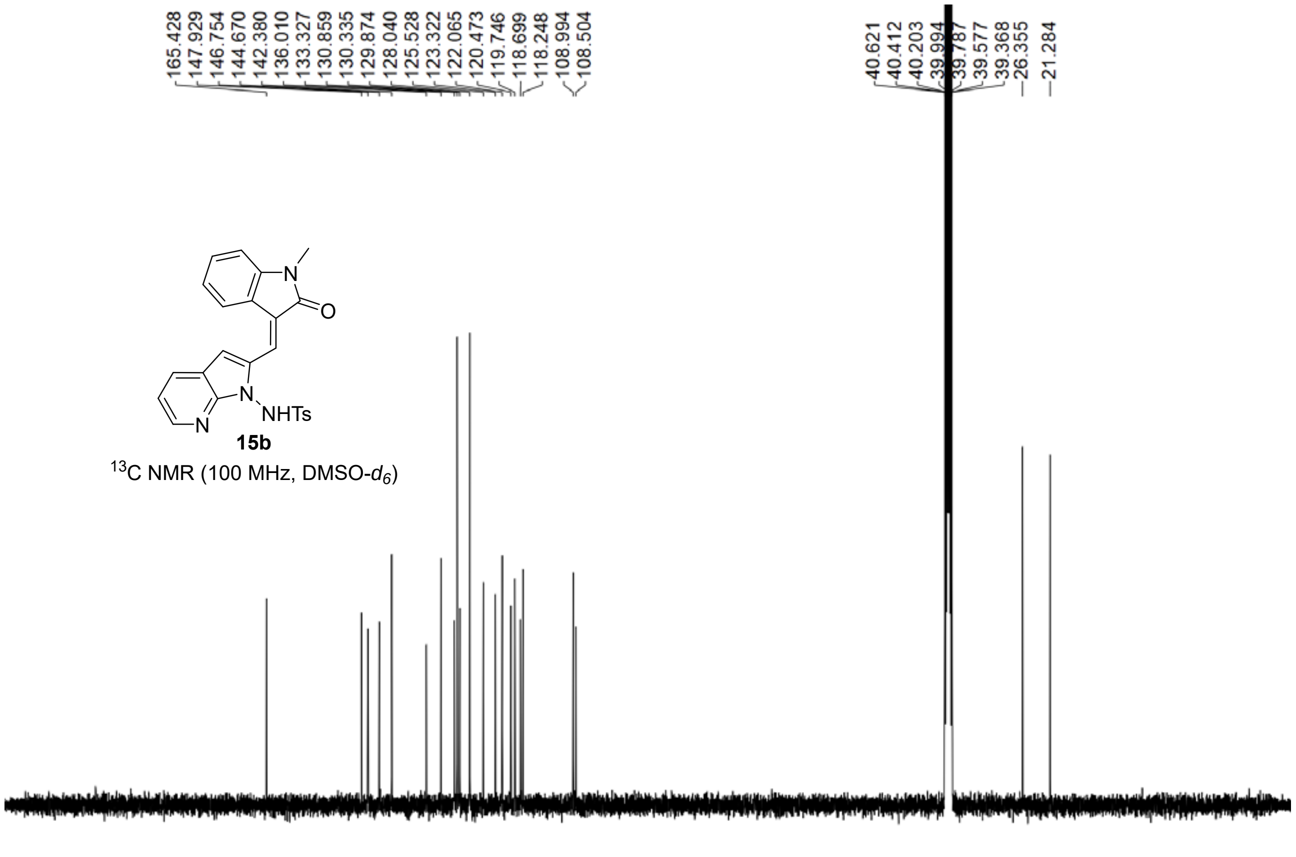
165.428
147.929
146.754
144.670
142.380
136.010
133.327
130.859
130.335
129.874
128.040
125.528
123.322
122.065
120.473
119.746
118.699
118.248
108.994
108.504
40.621
40.412
40.203
39.994
39.787
39.577
39.368
26.355
21.284
N
O
N
N
NHTs
15b
^{13}C NMR (100 MHz, DMSO-d_6)
f1 (ppm)

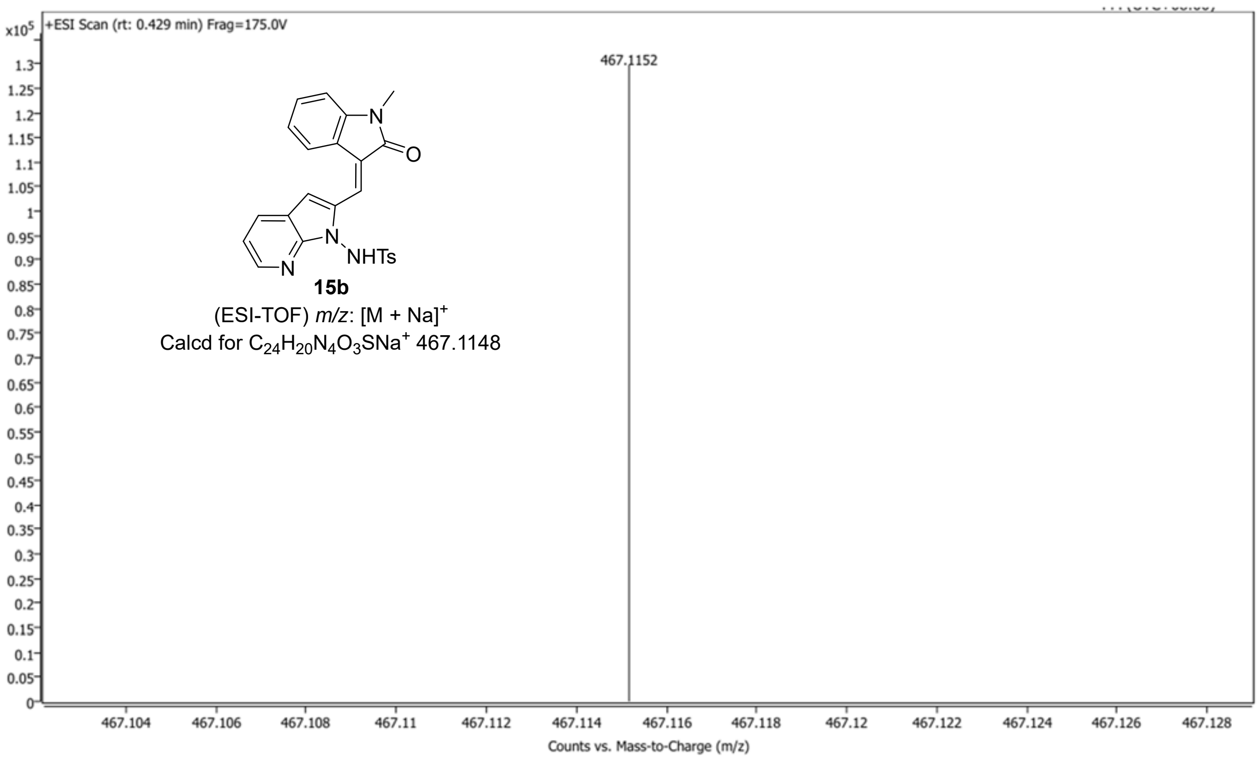

+ESI Scan (rt: 0.429 min) Frag=175.0V
x10^5
467.1152
N
O
N
N
NHTs
15b
(ESI-TOF) m/z: [M + Na]+
Calcd for C24H20N4O3SNa+ 467.1148
Counts vs. Mass-to-Charge (m/z)

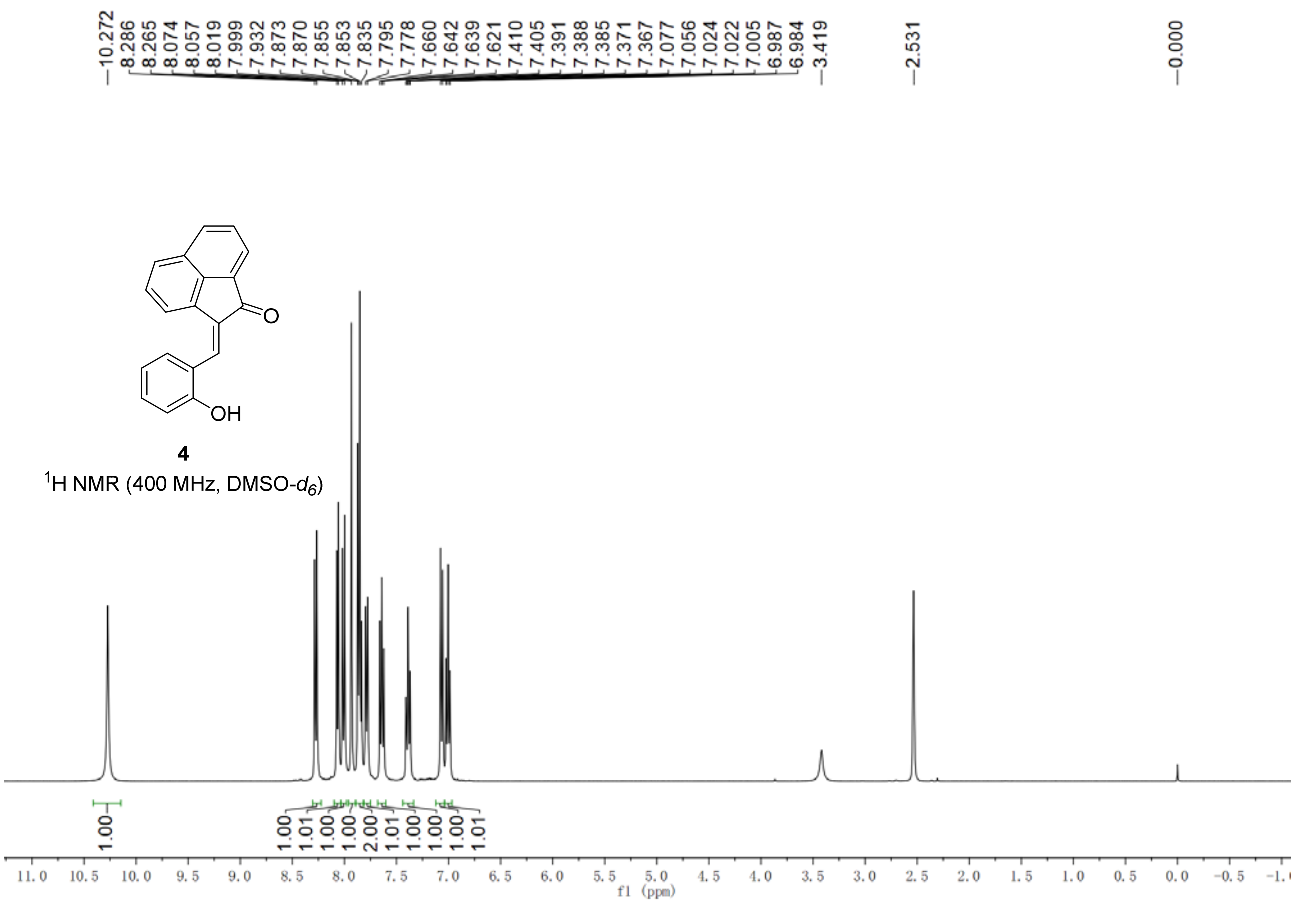

4

^{1}H NMR (400 MHz, DMSO-d_6)

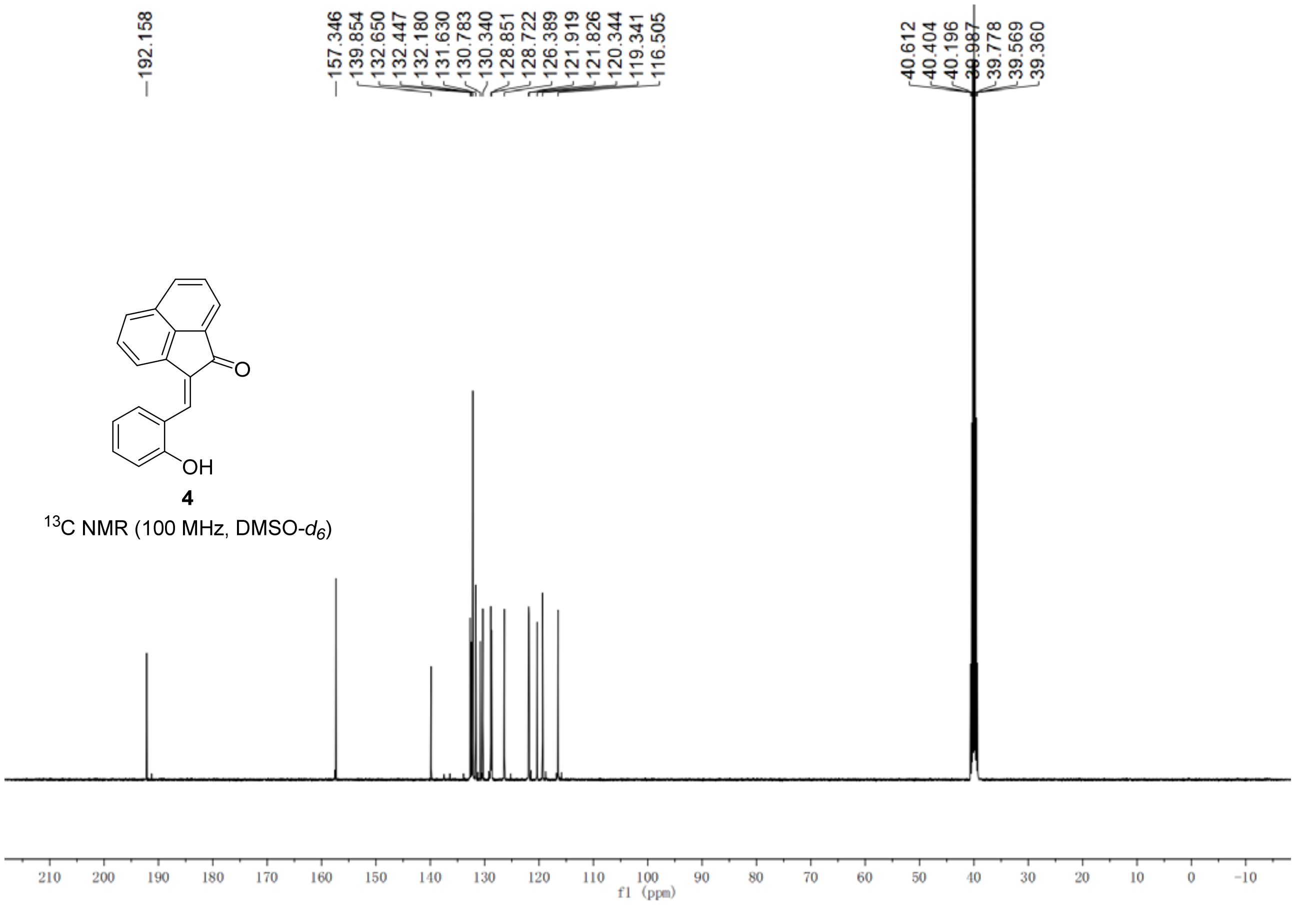

4

^{13}C NMR (100 MHz, DMSO-d_6)

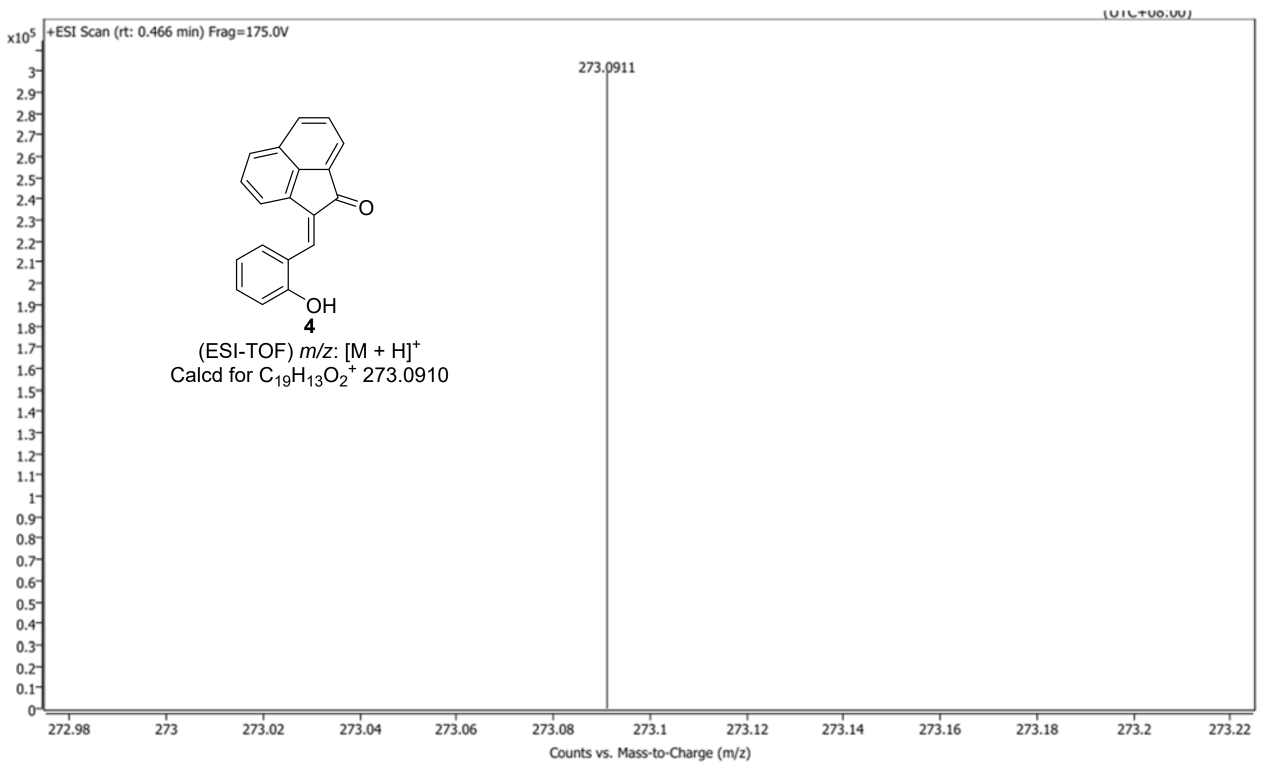

(UTC+08:00)
x10^5
+ESI Scan (rt: 0.466 min) Frag=175.0V
273.0911
OH
4
(ESI-TOF) m/z: [M + H]+
Calcd for C19H13O2+ 273.0910
Counts vs. Mass-to-Charge (m/z)

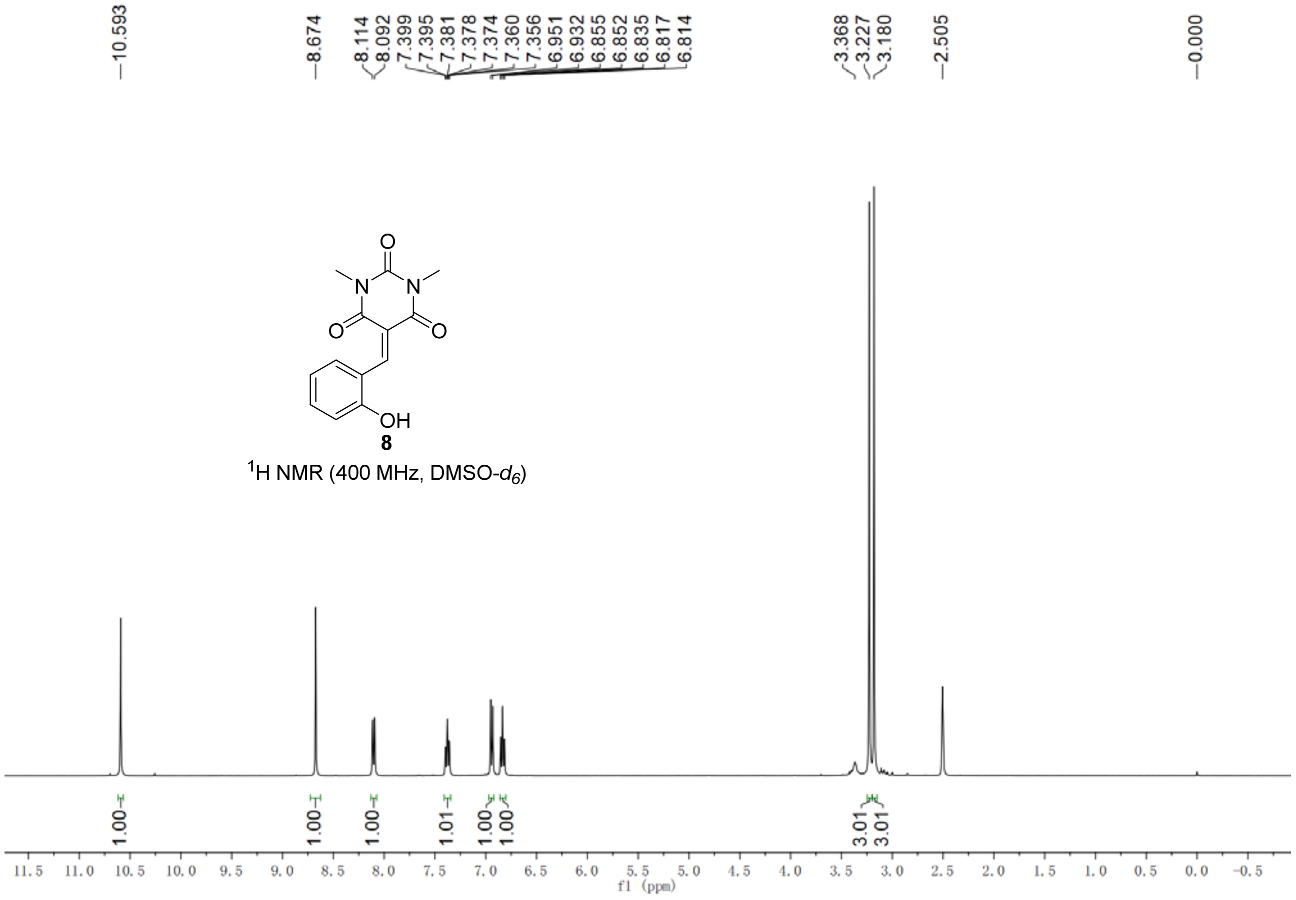
10.593
8.674
8.114
8.092
7.399
7.395
7.381
7.378
7.374
7.360
7.356
6.951
6.932
6.855
6.852
6.835
6.817
6.814
3.368
3.227
3.180
2.505
0.000
O
N
N
O
O
OH
8
^{1}H NMR (400 MHz, DMSO-d_6)
1.00
1.00
1.00
1.01
1.00
1.00
3.01
3.01
11.5 11.0 10.5 10.0 9.5 9.0 8.5 8.0 7.5 7.0 6.5 6.0 5.5 5.0 4.5 4.0 3.5 3.0 2.5 2.0 1.5 1.0 0.5 0.0 -0.5
f1 (ppm)

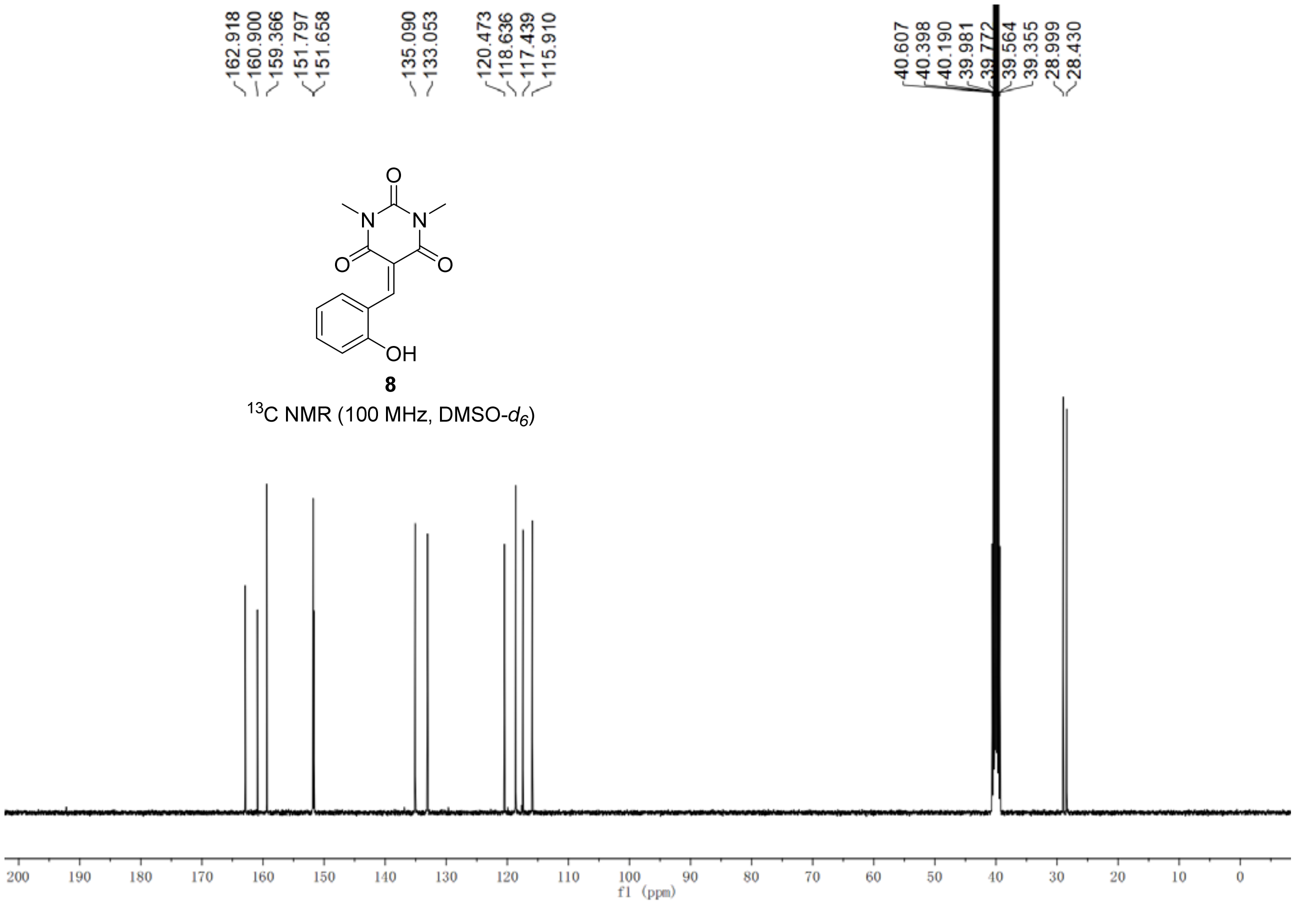
162.918
160.900
159.366
151.797
151.658
135.090
133.053
120.473
118.636
117.439
115.910
40.607
40.398
40.190
39.981
39.772
39.564
39.355
28.999
28.430
O
N
N
O
O
OH
8
^{13}C NMR (100 MHz, DMSO-d_6)
200 190 180 170 160 150 140 130 120 110 100 90 80 70 60 50 40 30 20 10 0
f1 (ppm)

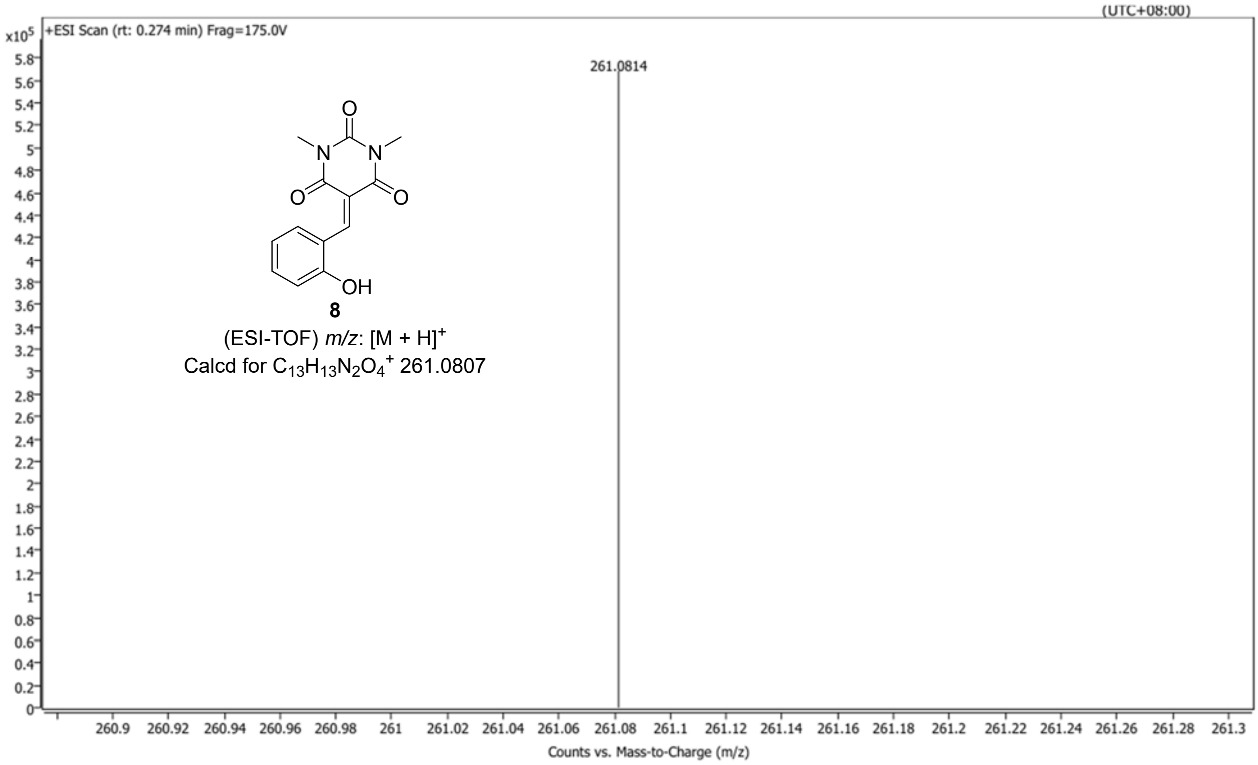
(UTC+08:00)
+ESI Scan (rt: 0.274 min) Frag=175.0V
x10^5
261.0814
8
(ESI-TOF) m/z: [M + H]+
Calcd for C13H13N2O4+ 261.0807
Counts vs. Mass-to-Charge (m/z)

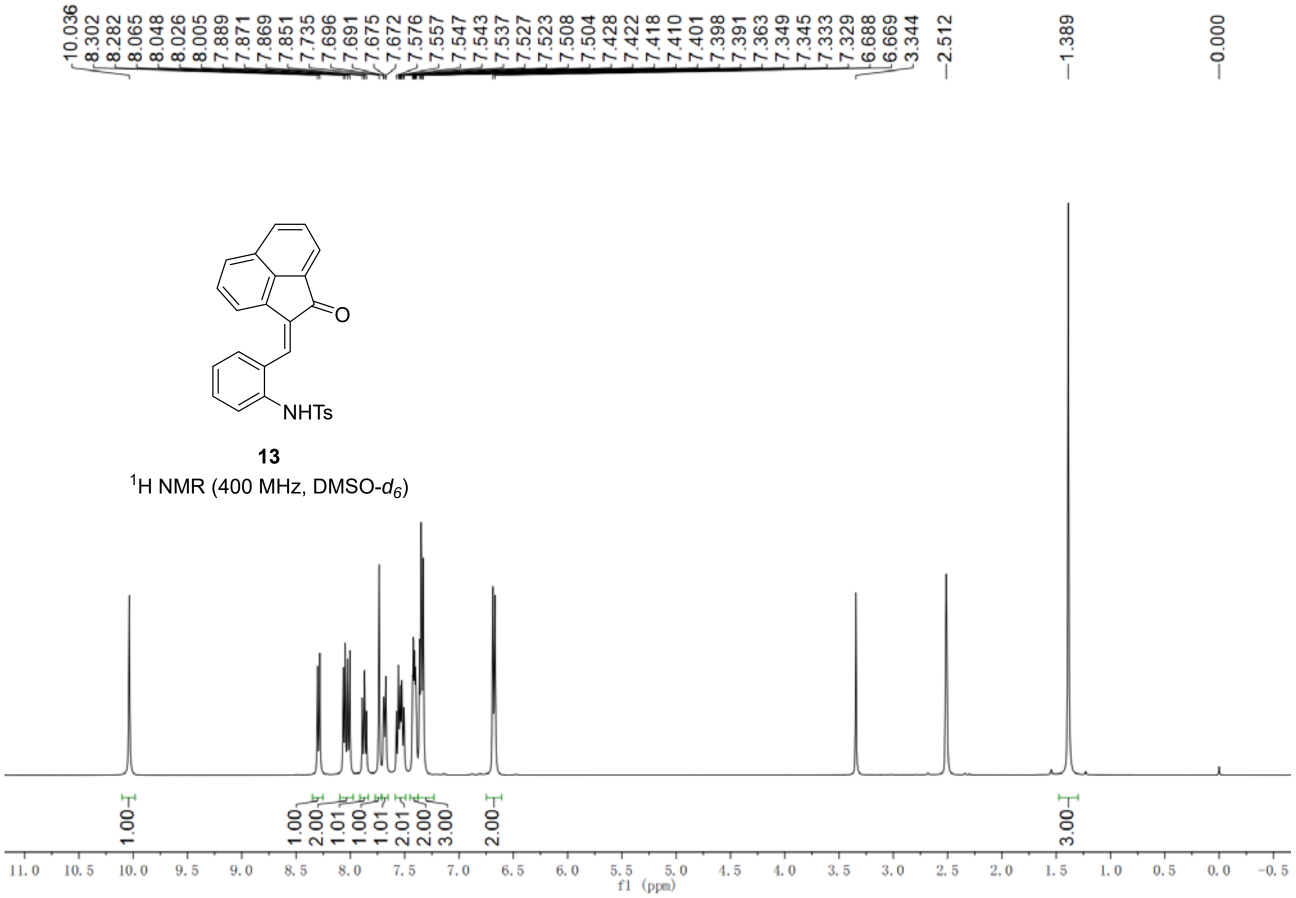
10.036
8.302
8.282
8.065
8.048
8.026
8.005
7.889
7.871
7.869
7.851
7.735
7.696
7.691
7.675
7.672
7.576
7.557
7.547
7.543
7.537
7.527
7.523
7.508
7.504
7.428
7.422
7.418
7.410
7.401
7.398
7.391
7.363
7.349
7.345
7.333
7.329
6.688
6.669
3.344
2.512
1.389
0.000
O
NHTs
13
1H NMR (400 MHz, DMSO-d6)
1.00
1.00
2.00
1.01
1.00
1.01
2.01
2.00
3.00
2.00
3.00
11.0
10.5
10.0
9.5
9.0
8.5
8.0
7.5
7.0
6.5
6.0
5.5
5.0
4.5
4.0
3.5
3.0
2.5
2.0
1.5
1.0
0.5
0.0
-0.5
f1 (ppm)

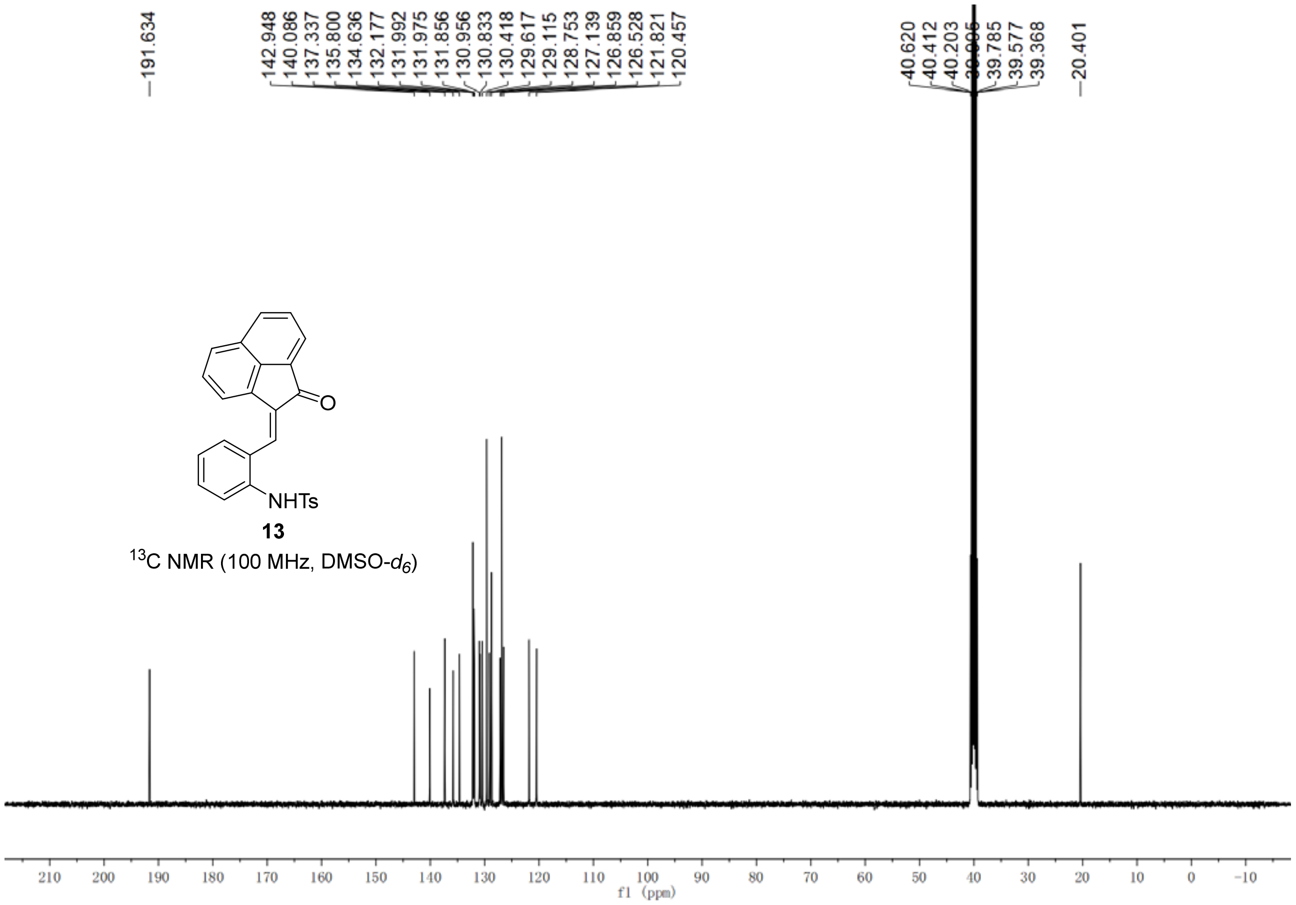
191.634
142.948
140.086
137.337
135.800
134.636
132.177
131.992
131.975
131.856
130.956
130.833
130.418
129.617
129.115
128.753
127.139
126.859
126.528
121.821
120.457
40.620
40.412
40.203
39.785
39.577
39.368
20.401
O
NHTs
13
13C NMR (100 MHz, DMSO-d6)
210
200
190
180
170
160
150
140
130
120
110
100
90
80
70
60
50
40
30
20
10
0
-10
f1 (ppm)

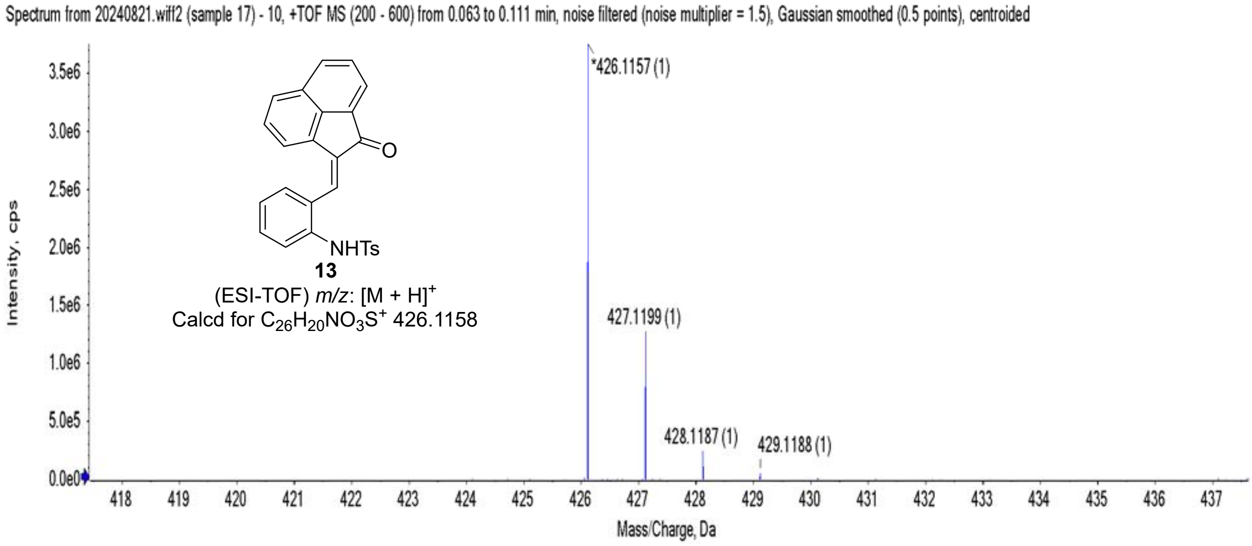

Spectrum from 20240821.wiff2 (sample 17) - 10, +TOF MS (200 - 600) from 0.063 to 0.111 min, noise filtered (noise multiplier = 1.5), Gaussian smoothed (0.5 points), centroided
Intensity, cps
3.5e6
3.0e6
2.5e6
2.0e6
1.5e6
1.0e6
5.0e5
0.0e0
*426.1157 (1)
427.1199 (1)
428.1187 (1)
429.1188 (1)
Mass/Charge, Da
O
NHTs
13
(ESI-TOF) m/z: [M + H]+
Calcd for C26H20NO3S+ 426.1158

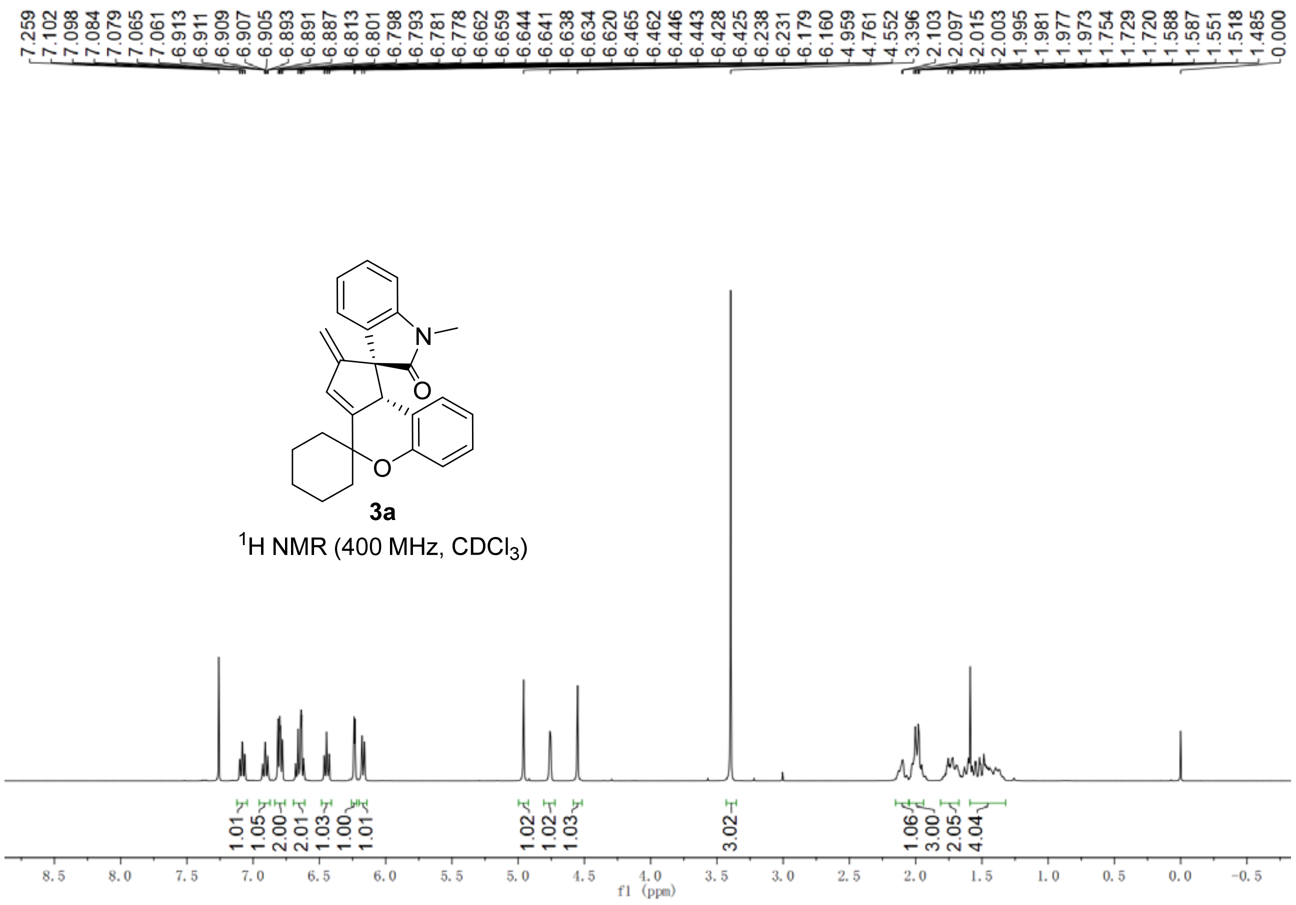
7.259 7.102 7.098 7.084 7.079 7.065 7.061 6.913 6.911 6.909 6.907 6.905 6.893 6.891 6.887 6.813 6.801 6.798 6.793 6.781 6.778 6.662 6.659 6.644 6.641 6.638 6.634 6.620 6.465 6.462 6.446 6.443 6.428 6.425 6.238 6.231 6.179 6.160 4.959 4.761 4.552 3.396 2.103 2.097 2.015 2.003 1.995 1.981 1.977 1.973 1.754 1.729 1.720 1.588 1.587 1.551 1.518 1.485 0.000
N
O
O
3a
^{1}H NMR (400 MHz, $CDCl_3$)
1.01 1.05 2.00 2.01 1.03 1.00 1.01 1.02 1.02 1.03 3.02 1.06 3.00 2.05 4.04
8.5 8.0 7.5 7.0 6.5 6.0 5.5 5.0 4.5 4.0 3.5 3.0 2.5 2.0 1.5 1.0 0.5 0.0 -0.5
f1 (ppm)

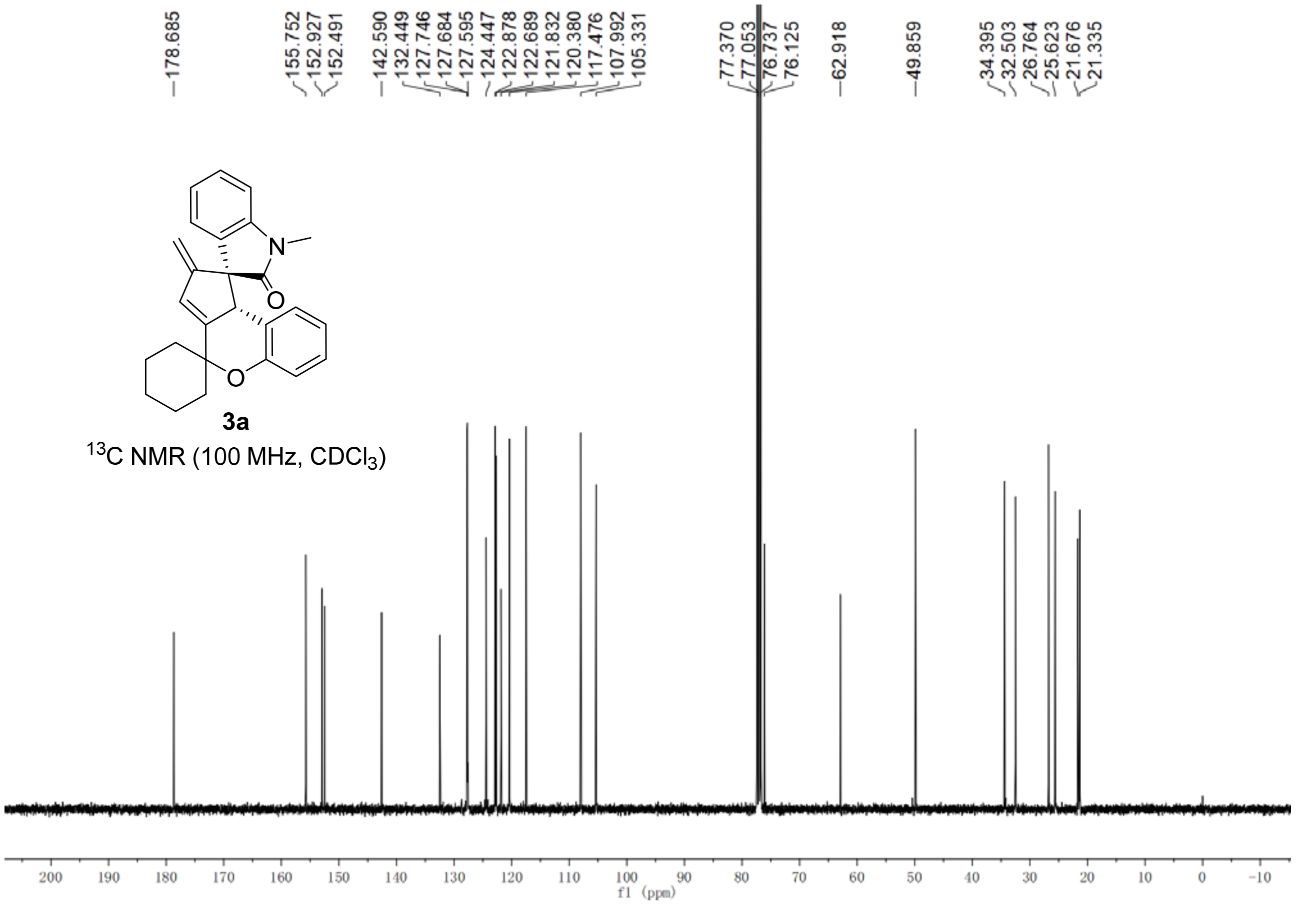
178.685 155.752 152.927 152.491 142.590 132.449 127.746 127.684 127.595 124.447 122.878 122.689 121.832 120.380 117.476 107.992 105.331 77.370 77.053 76.737 76.125 62.918 49.859 34.395 32.503 26.764 25.623 21.676 21.335
N
O
O
3a
^{13}C NMR (100 MHz, $CDCl_3$)
200 190 180 170 160 150 140 130 120 110 100 90 80 70 60 50 40 30 20 10 0 -10
f1 (ppm)

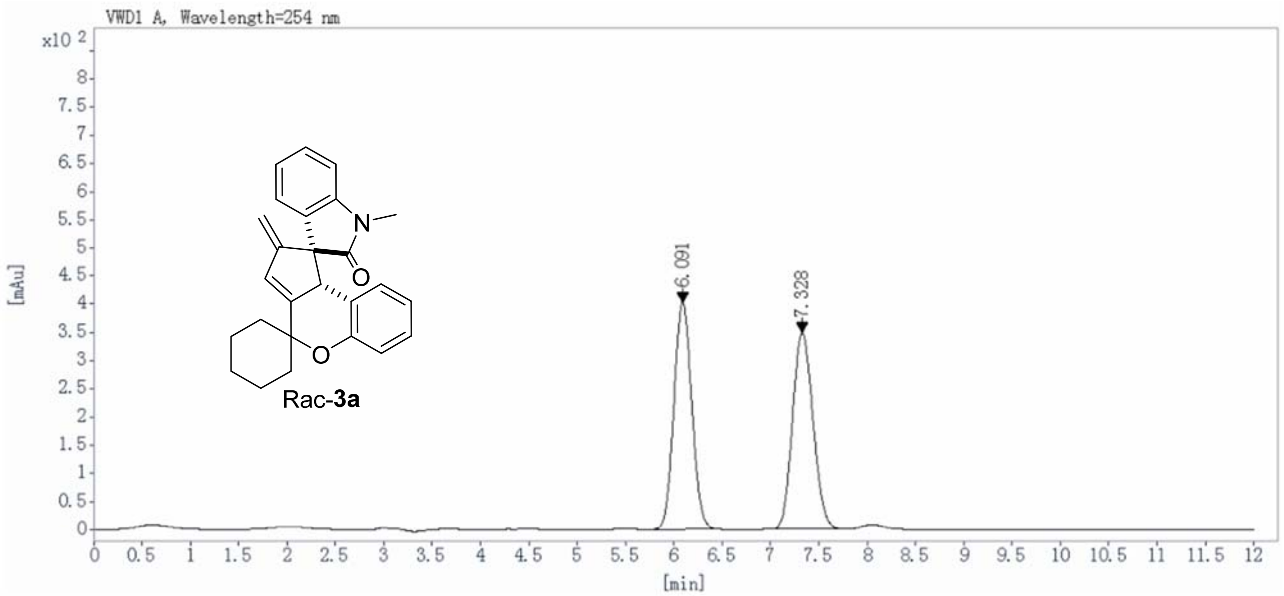

Ret Time [min]	Peak Type	Width [min]	Height [mAU]	Area [mAU*s]	Area [%]
6.091	BBA	0.20	402.5508	5172.2280	50.0206
7.328	BBA	0.23	347.8087	5167.9780	49.9794
			Totals:	10340.2061	100.0000

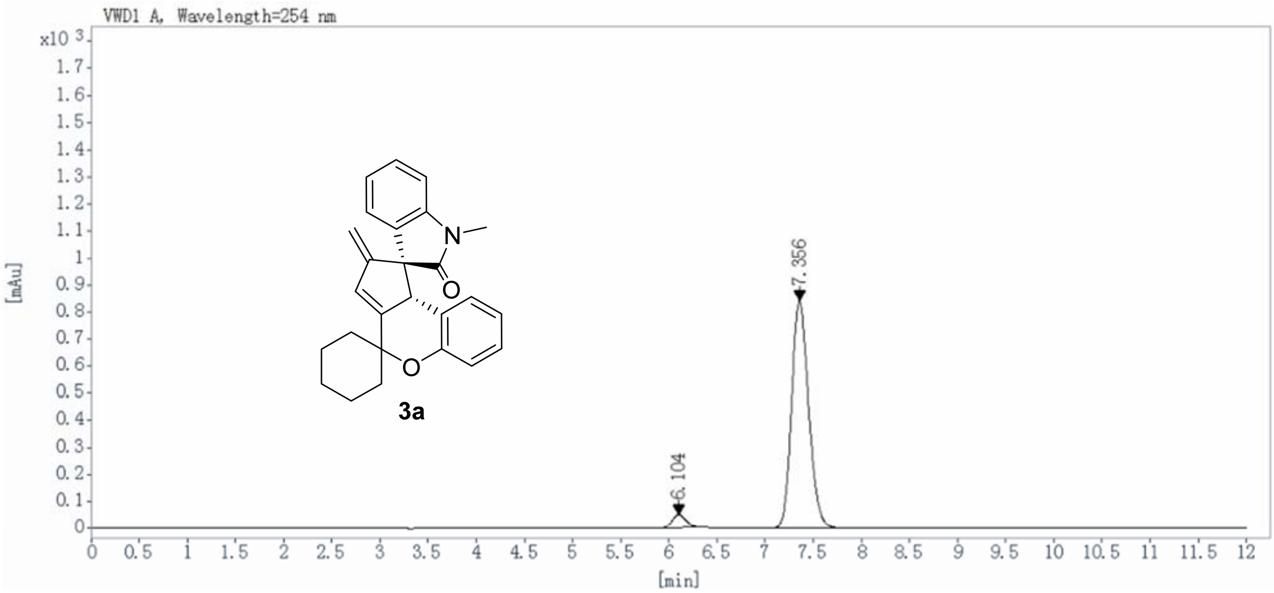

Ret Time [min]	Peak Type	Width [min]	Height [mAU]	Area [mAU*s]	Area [%]
6.104	BBA	0.15	49.1703	488.3062	4.5169
7.356	BBA	0.19	841.7538	10322.3574	95.4831
			Totals:	10810.6636	100.0000

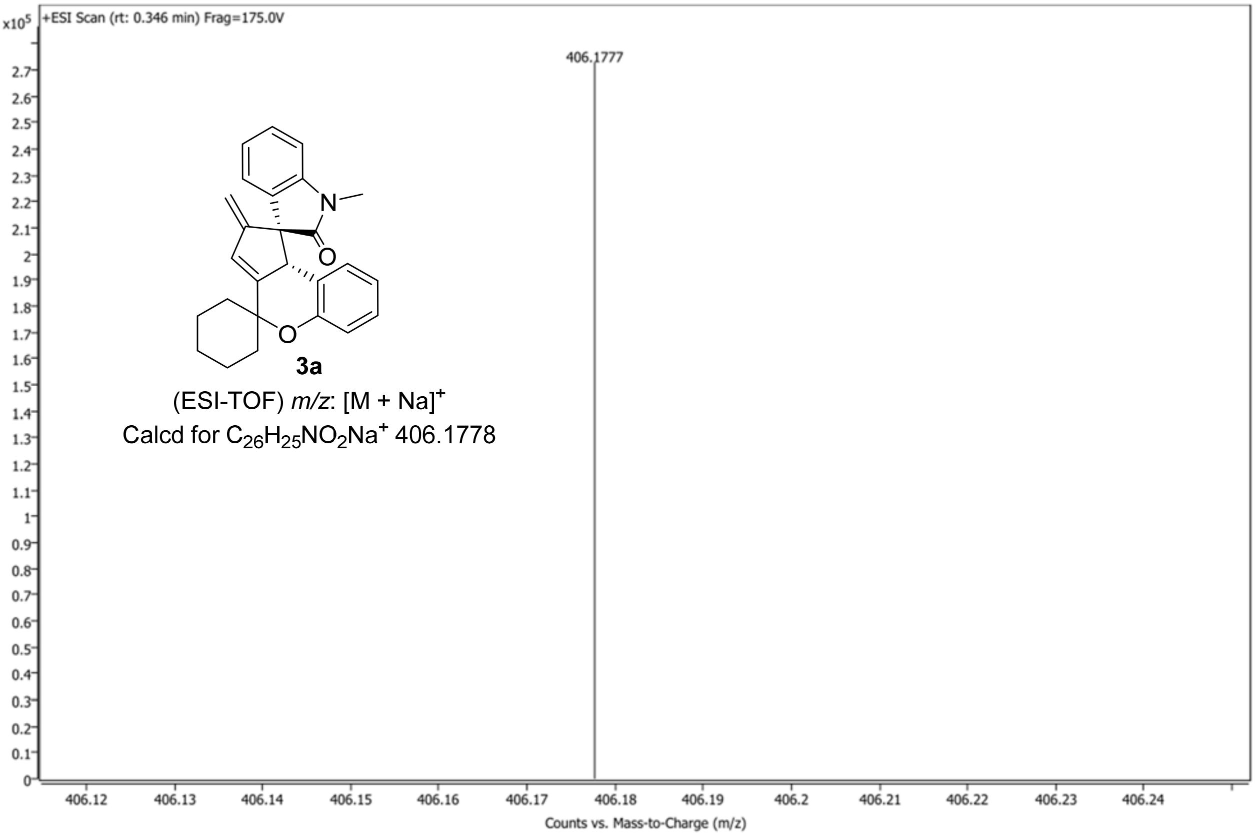
+ESI Scan (rt: 0.346 min) Frag=175.0V
x10^5
406.1777
3a
(ESI-TOF) m/z: [M + Na]+
Calcd for C26H25NO2Na+ 406.1778
N
O
O
Counts vs. Mass-to-Charge (m/z)

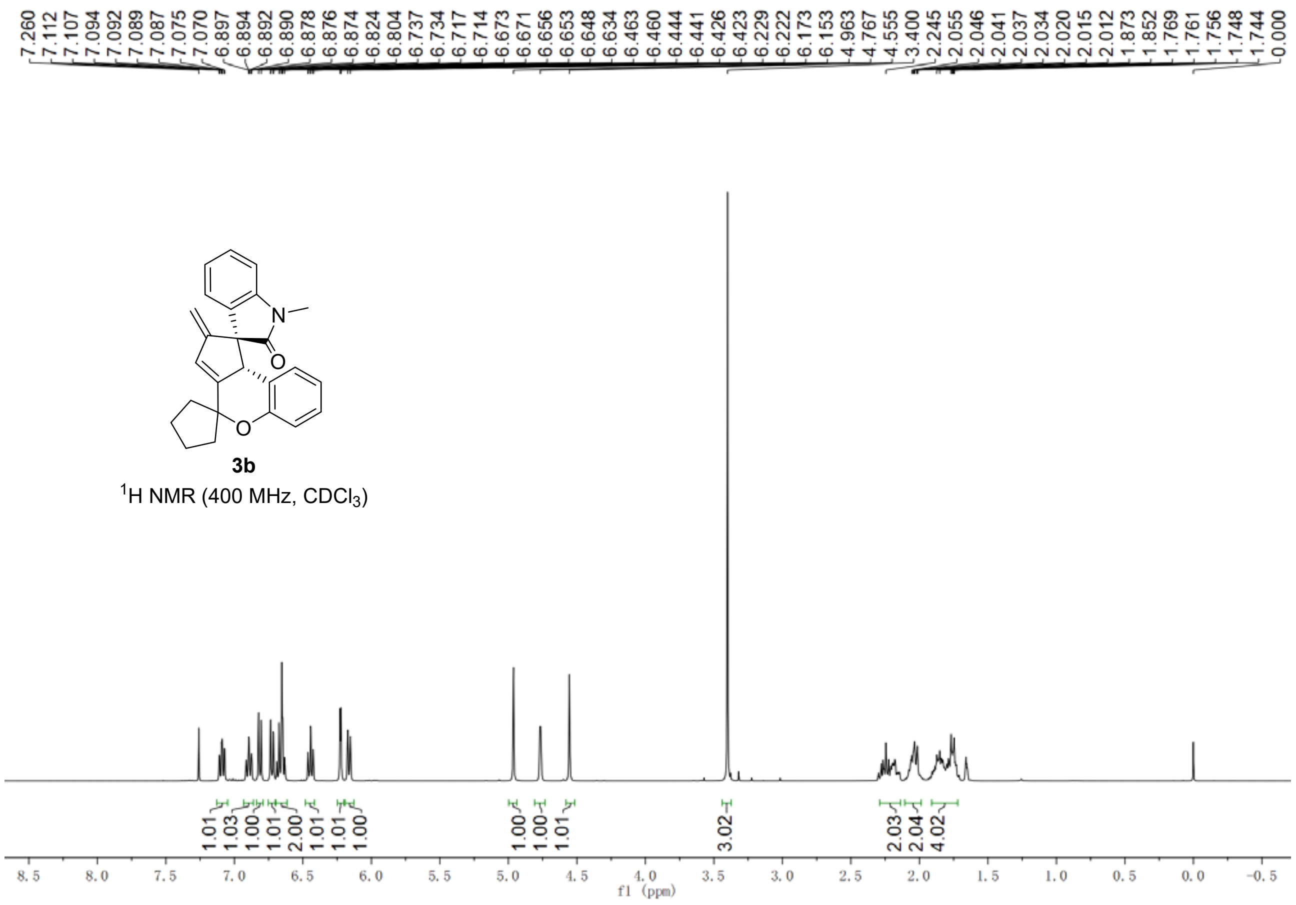
7.260 7.112 7.107 7.094 7.092 7.089 7.087 7.075 7.070 6.897 6.894 6.892 6.890 6.878 6.876 6.874 6.824 6.804 6.737 6.734 6.717 6.714 6.673 6.671 6.656 6.653 6.648 6.634 6.463 6.460 6.444 6.441 6.426 6.423 6.229 6.222 6.173 6.153 4.963 4.767 4.555 3.400 2.245 2.055 2.046 2.041 2.037 2.034 2.020 2.015 2.012 1.873 1.852 1.769 1.761 1.756 1.748 1.744 0.000
N
O
O
3b
^{1}H NMR (400 MHz, CDCl$_3$)
1.01 1.03 1.00 1.01 2.00 1.01 1.01 1.00 1.00 1.00 1.01 3.02 2.03 2.04 4.02
f1 (ppm)

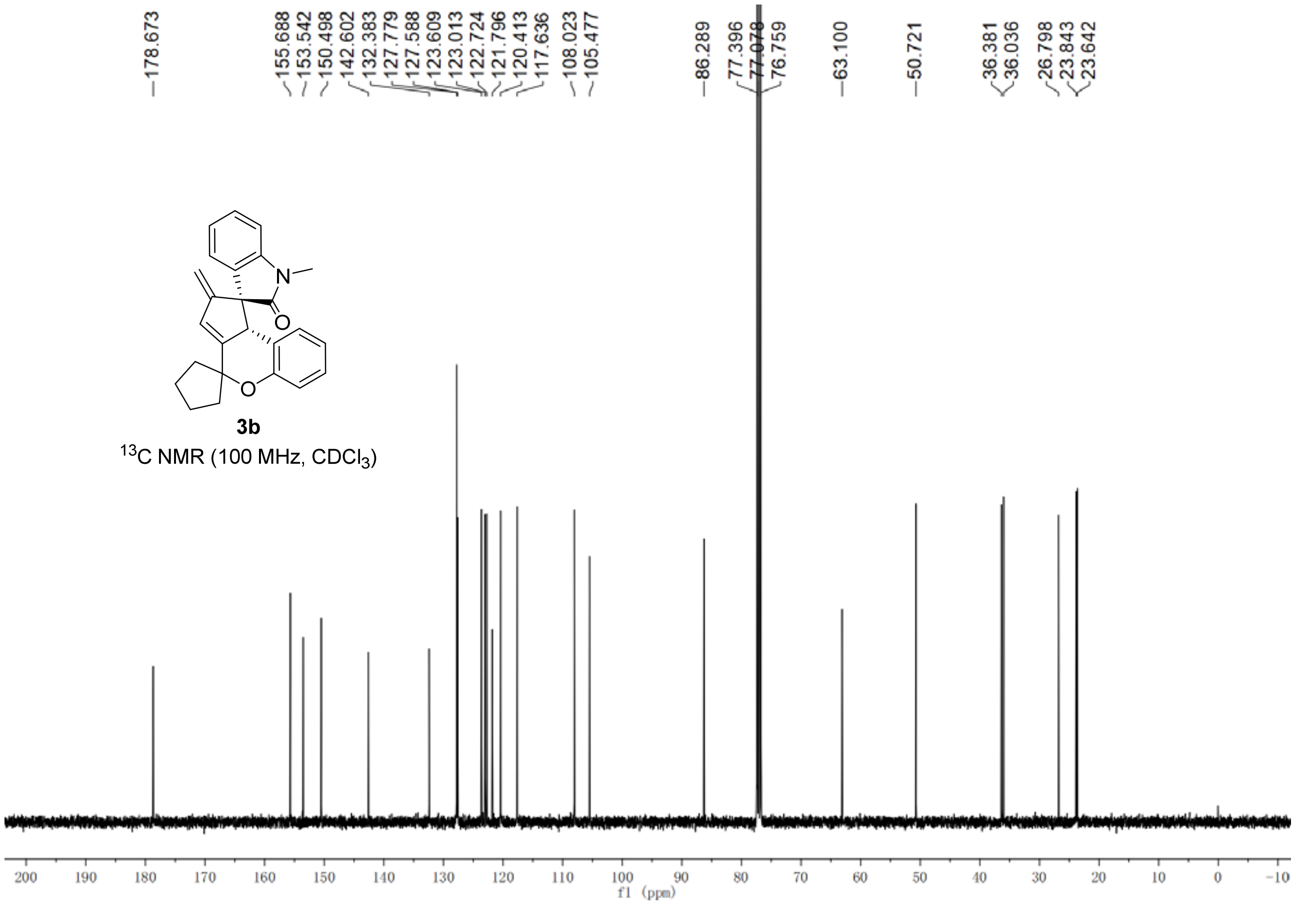
178.673 155.688 153.542 150.498 142.602 132.383 127.779 127.588 123.609 123.013 122.724 121.796 120.413 117.636 108.023 105.477 86.289 77.396 77.078 76.759 63.100 50.721 36.381 36.036 26.798 23.843 23.642
N
O
O
3b
^{13}C NMR (100 MHz, CDCl$_3$)
f1 (ppm)

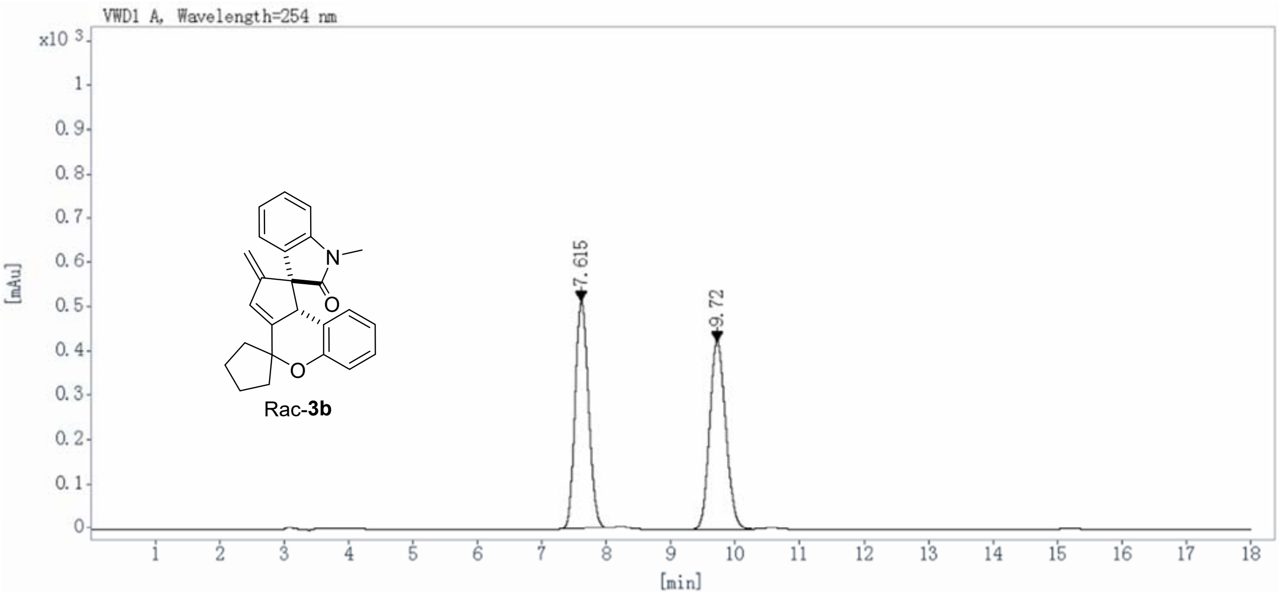

Ret Time [min]	Peak Type	Width [min]	Height [mAU]	Area [mAU*s]	Area [%]
7.615	BB	0.23	513.2851	7421.3164	50.0775
9.720	BB	0.27	423.6620	7398.3569	49.9225
			Totals:	14819.6733	100.0000

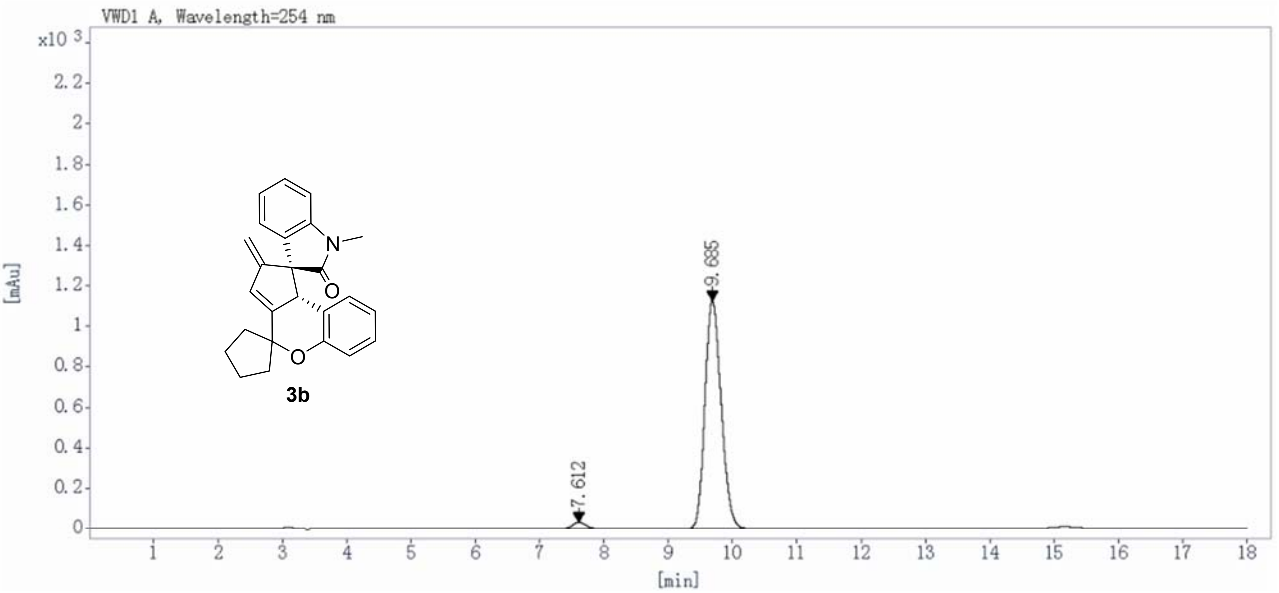

Ret Time [min]	Peak Type	Width [min]	Height [mAU]	Area [mAU*s]	Area [%]
7.612	BBA	0.22	32.6851	465.2503	2.3032
9.685	BB	0.27	1125.0988	19734.8965	97.6968
			Totals:	20200.1468	100.0000

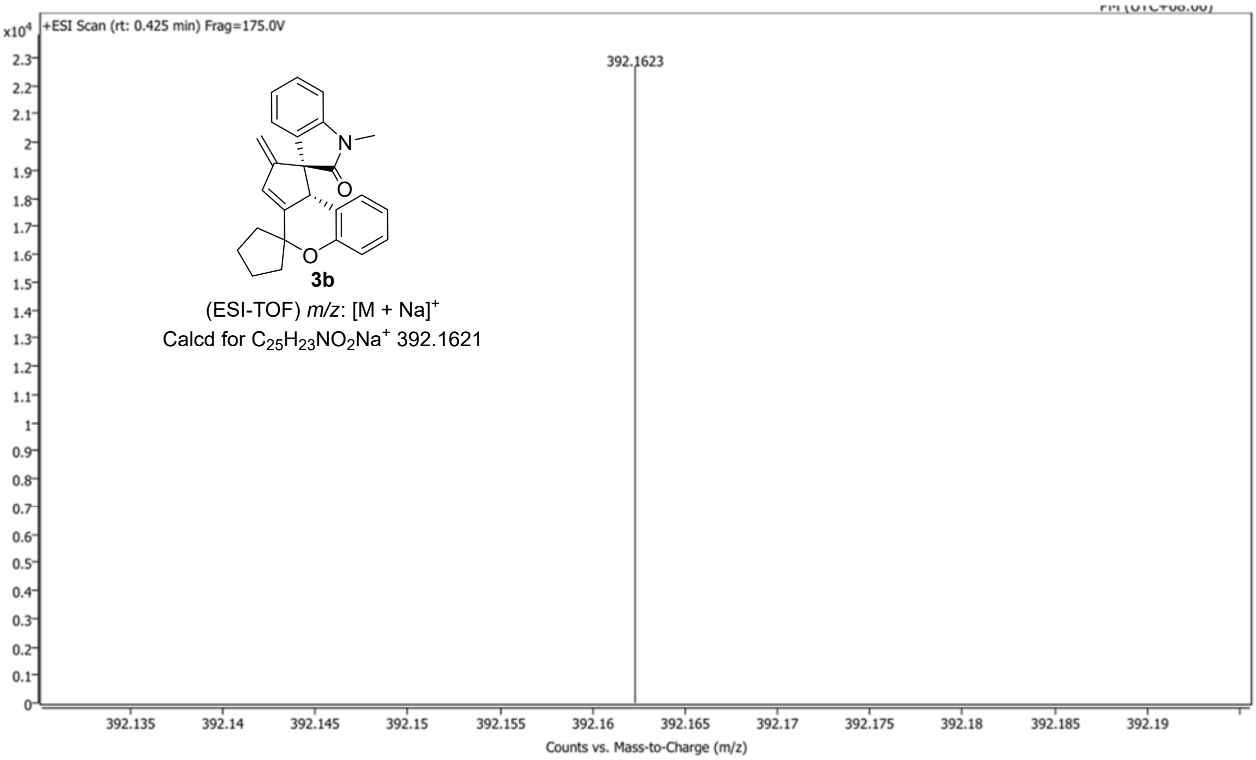
+ESI Scan (rt: 0.425 min) Frag=175.0V
x10^4
392.1623
N
O
O
3b
(ESI-TOF) m/z: [M + Na]+
Calcd for C25H23NO2Na+ 392.1621
Counts vs. Mass-to-Charge (m/z)

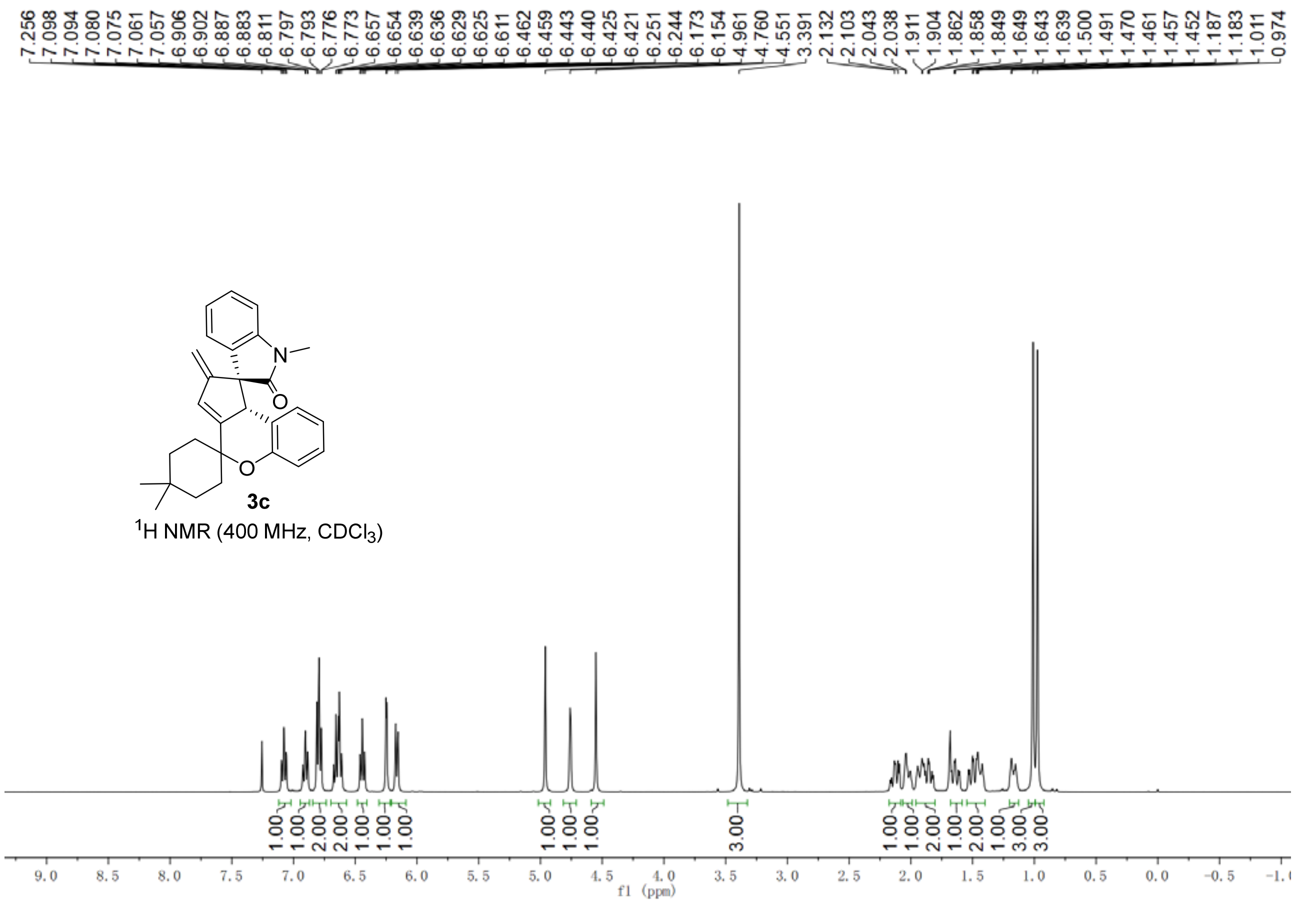
7.256
7.098
7.094
7.080
7.075
7.061
7.057
6.906
6.902
6.887
6.883
6.811
6.797
6.793
6.776
6.773
6.657
6.654
6.639
6.636
6.629
6.625
6.611
6.462
6.459
6.443
6.440
6.425
6.421
6.251
6.244
6.173
6.154
4.961
4.760
4.551
3.391
2.132
2.103
2.043
2.038
1.911
1.904
1.862
1.858
1.849
1.649
1.643
1.639
1.500
1.491
1.470
1.461
1.457
1.452
1.187
1.183
1.011
0.974
3c
1H NMR (400 MHz, CDCl3)
1.00
1.00
2.00
2.00
1.00
1.00
1.00
1.00
1.00
1.00
3.00
1.00
1.00
2.00
1.00
2.00
1.00
3.00
3.00
f1 (ppm)

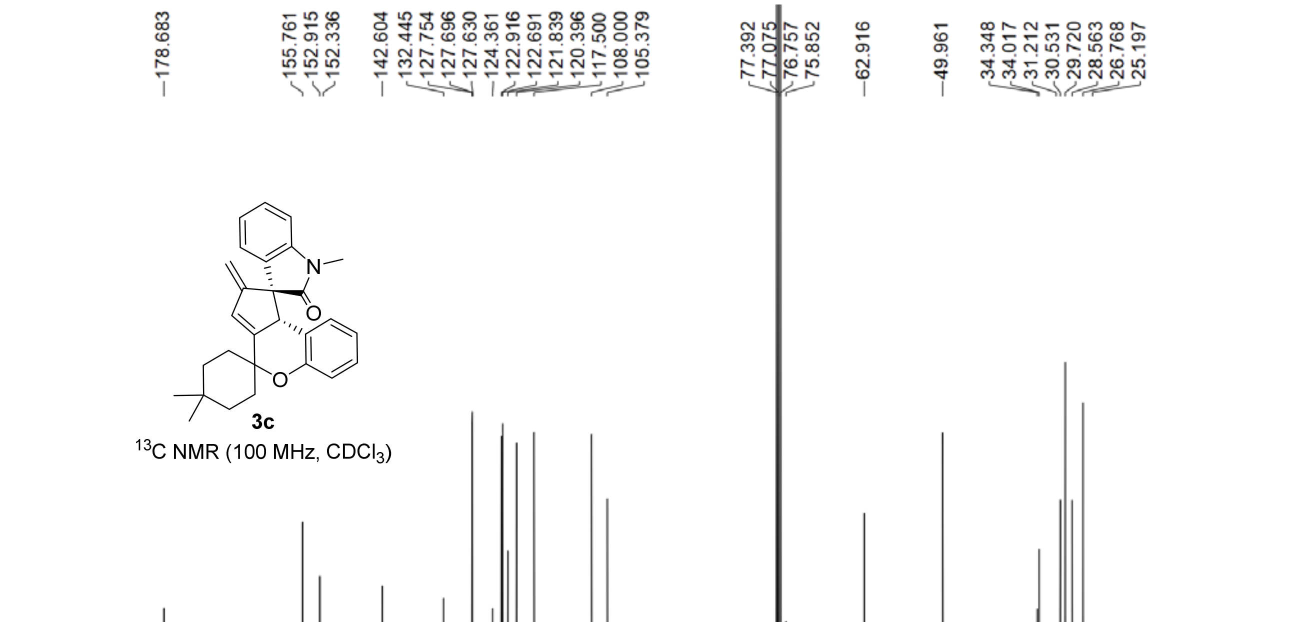
178.683
155.761
152.915
152.336
142.604
132.445
127.754
127.696
127.630
124.361
122.916
122.691
121.839
120.396
117.500
108.000
105.379
77.392
77.075
76.757
75.852
62.916
49.961
34.348
34.017
31.212
30.531
29.720
28.563
26.768
25.197
3c
13C NMR (100 MHz, CDCl3)
f1 (ppm)

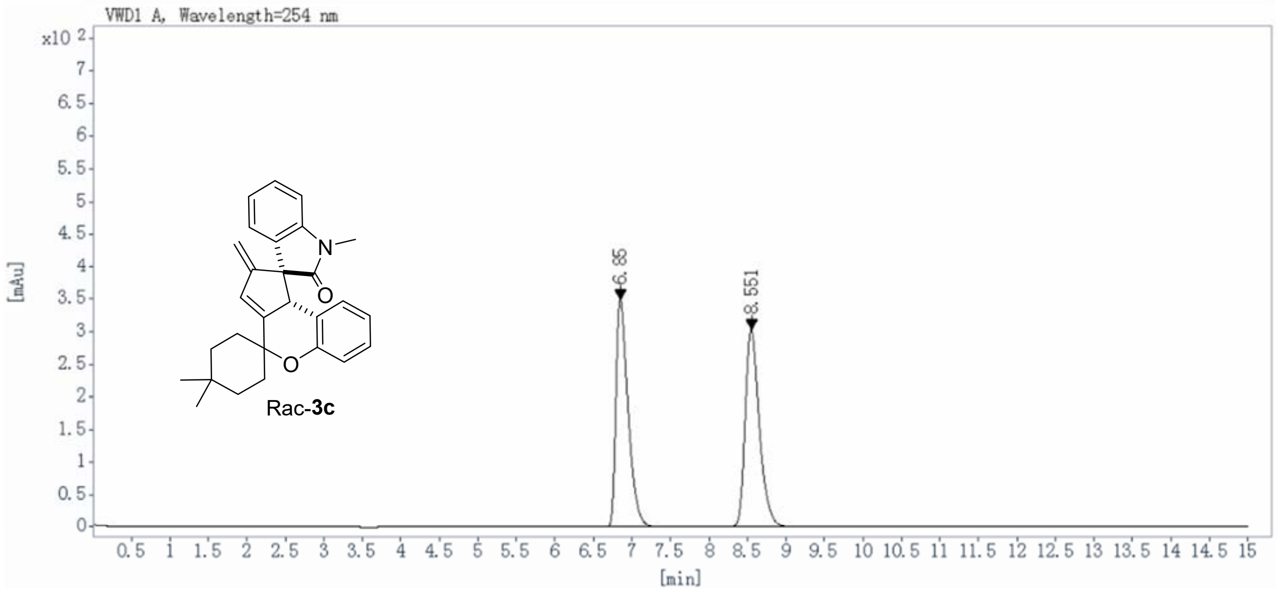

Ret Time [min]	Peak Type	Width [min]	Height [mAU]	Area [mAU*s]	Area [%]
6.850	BBA	0.17	349.2123	3864.9224	49.8809
8.551	BBA	0.20	302.6716	3883.3809	50.1191
			Totals:	7748.3032	100.0000

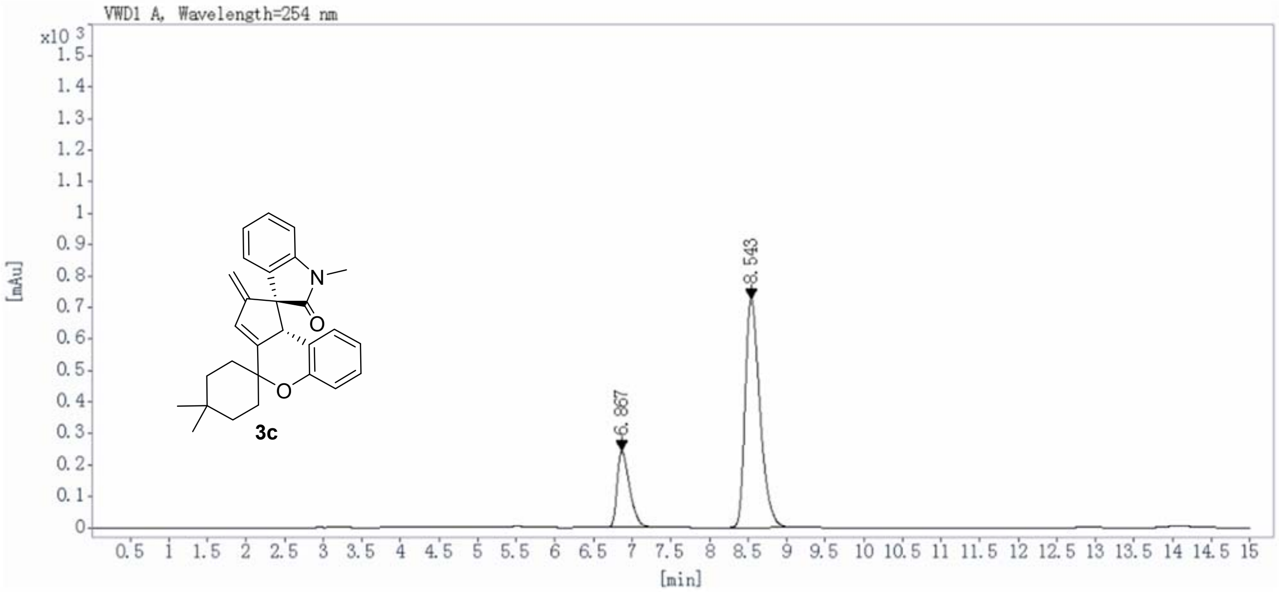

Ret Time [min]	Peak Type	Width [min]	Height [mAU]	Area [mAU*s]	Area [%]
6.867	BBA	0.18	243.2090	2777.2302	22.1981
8.543	BBA	0.20	727.0814	9733.8750	77.8019
			Totals:	12511.1052	100.0000

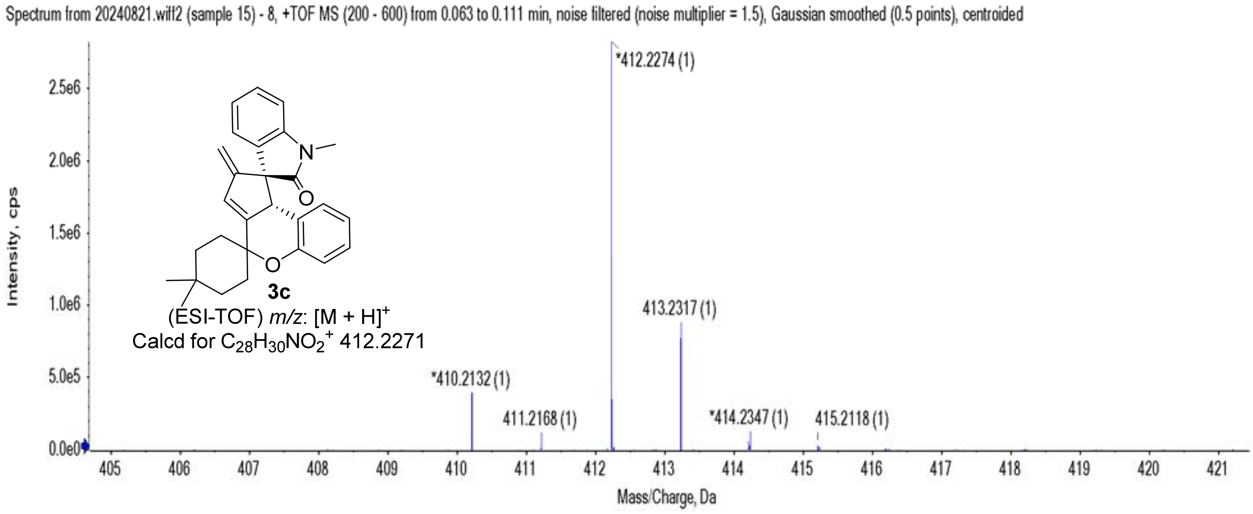

Spectrum from 20240821.wiff2 (sample 15) - 8, +TOF MS (200 - 600) from 0.063 to 0.111 min, noise filtered (noise multiplier = 1.5), Gaussian smoothed (0.5 points), centroided
*412.2274 (1)
413.2317 (1)
*410.2132 (1)
411.2168 (1)
*414.2347 (1)
415.2118 (1)
Intensity, cps
Mass/Charge, Da
N
O
O
3c
(ESI-TOF) m/z: [M + H]+
Calcd for C28H30NO2+ 412.2271

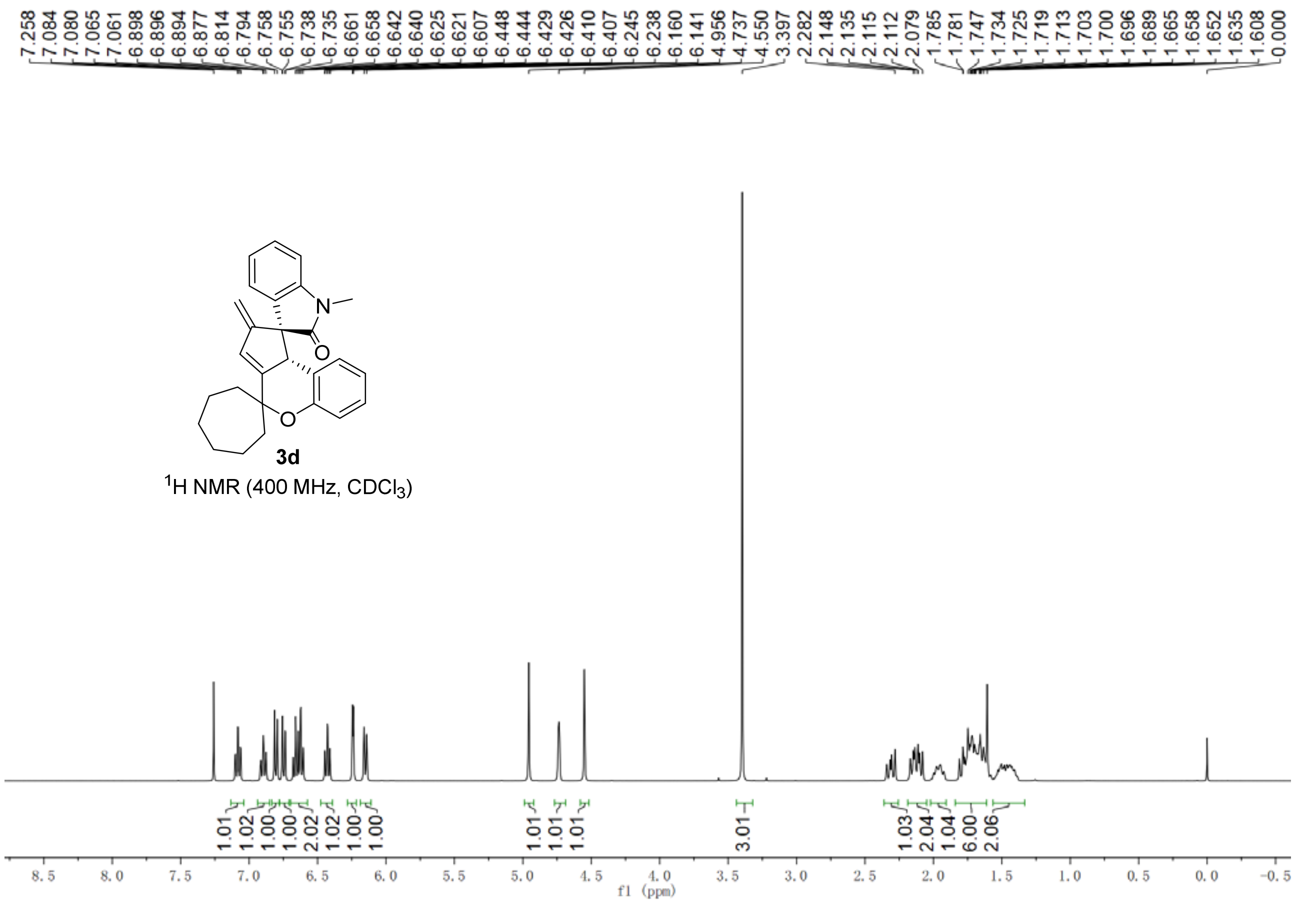

3d

^{1}H NMR (400 MHz, $CDCl_3$)

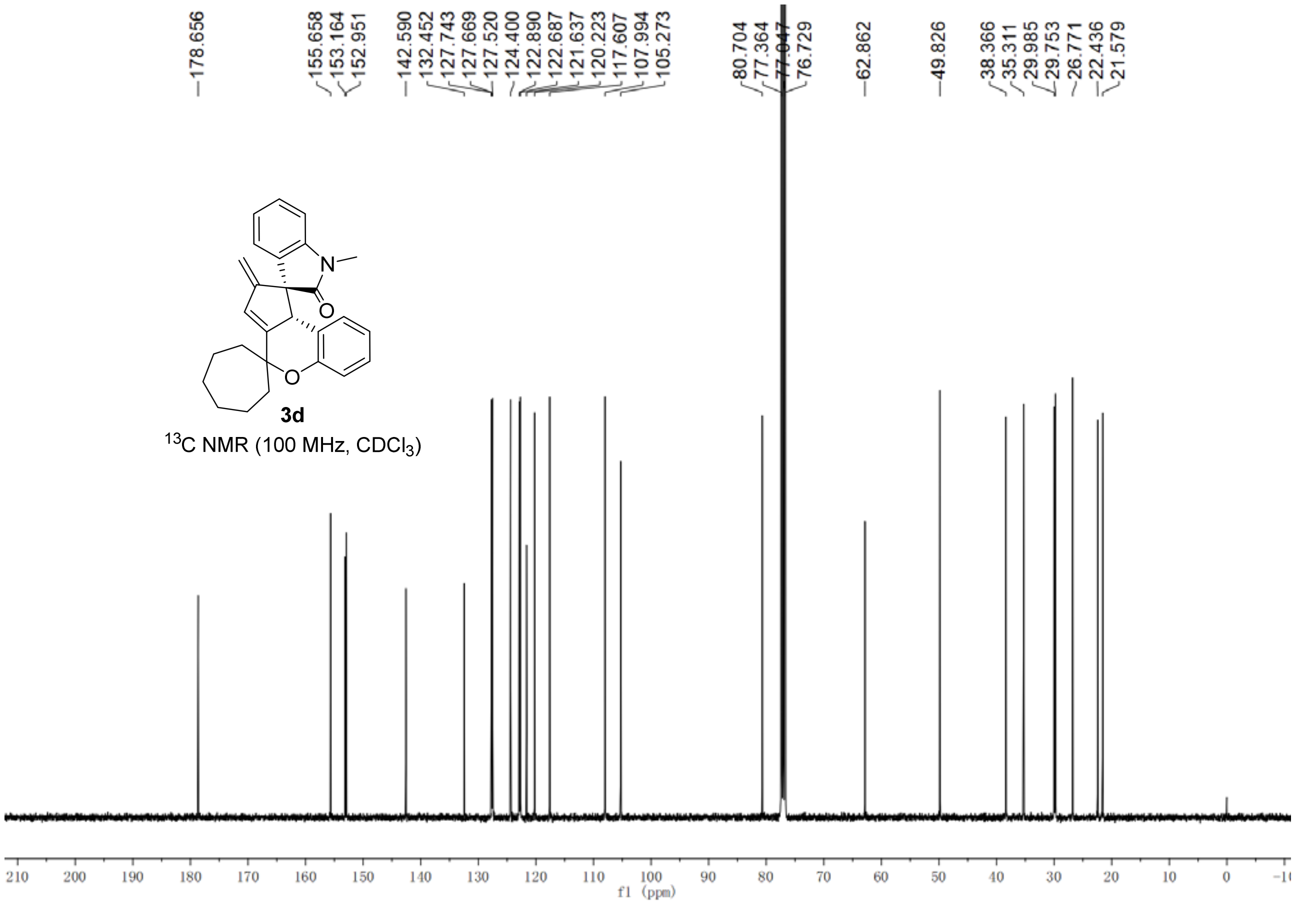

3d

^{13}C NMR (100 MHz, $CDCl_3$)

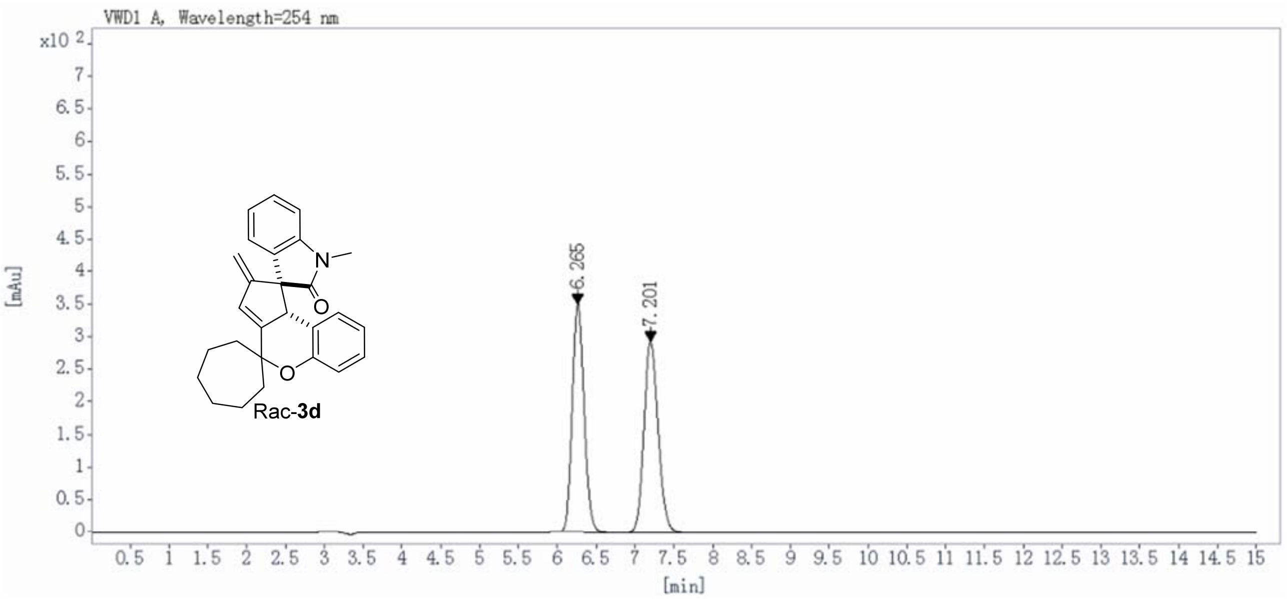

Ret Time [min]	Peak Type	Width [min]	Height [mAU]	Area [mAU*s]	Area [%]
6.265	BBA	0.16	349.9322	3710.8174	49.8768
7.201	BBA	0.20	291.8110	3729.1545	50.1232
			Totals:	7439.9719	100.0000

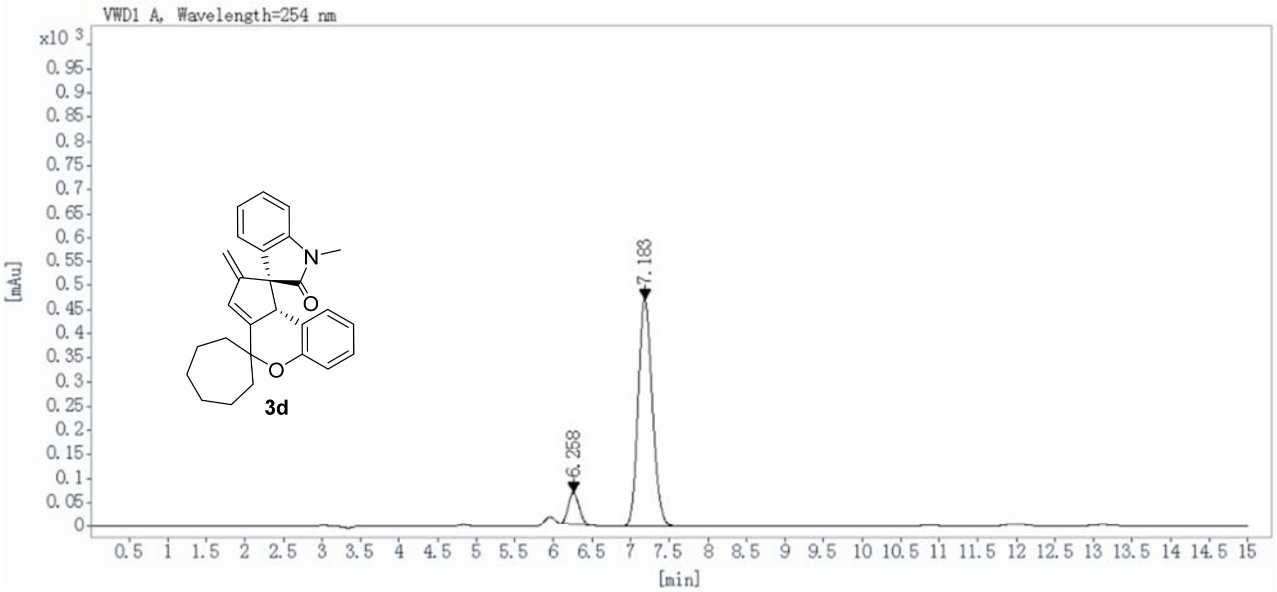

Ret Time [min]	Peak Type	Width [min]	Height [mAU]	Area [mAU*s]	Area [%]
6.258	BBA	0.15	65.9337	642.6335	9.5210
7.183	BBA	0.20	471.5378	6107.0034	90.4790
			Totals:	6749.6369	100.0000

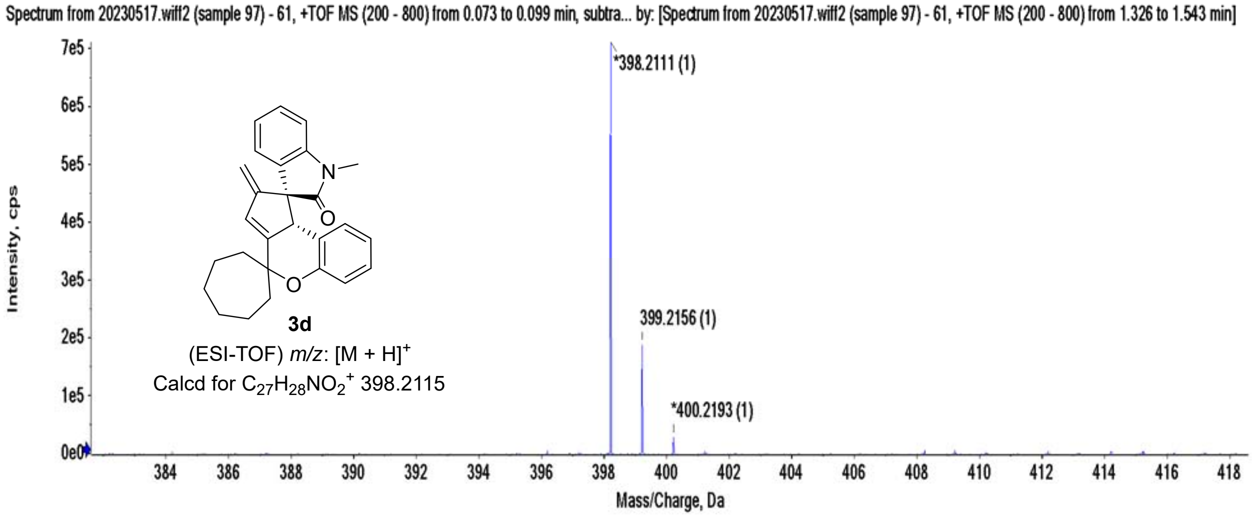
Spectrum from 20230517.wiff2 (sample 97) - 61, +TOF MS (200 - 800) from 0.073 to 0.099 min, subtra... by: [Spectrum from 20230517.wiff2 (sample 97) - 61, +TOF MS (200 - 800) from 1.326 to 1.543 min]
Intensity, cps
Mass/Charge, Da
*398.2111 (1)
399.2156 (1)
*400.2193 (1)
N
O
O
3d
(ESI-TOF) m/z: [M + H]+
Calcd for C27H28NO2+ 398.2115

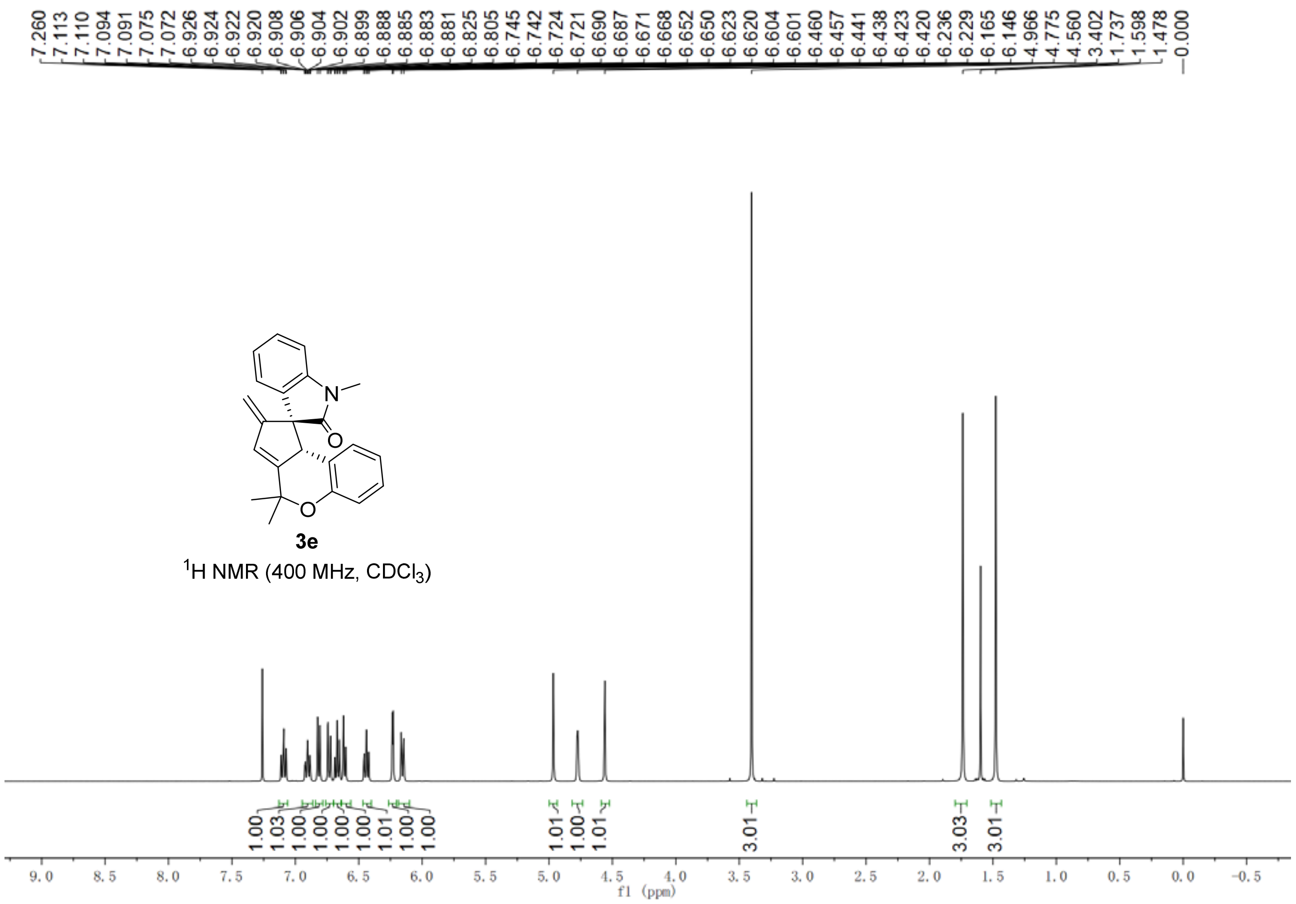

3e

^{1}H NMR (400 MHz, $CDCl_3$)

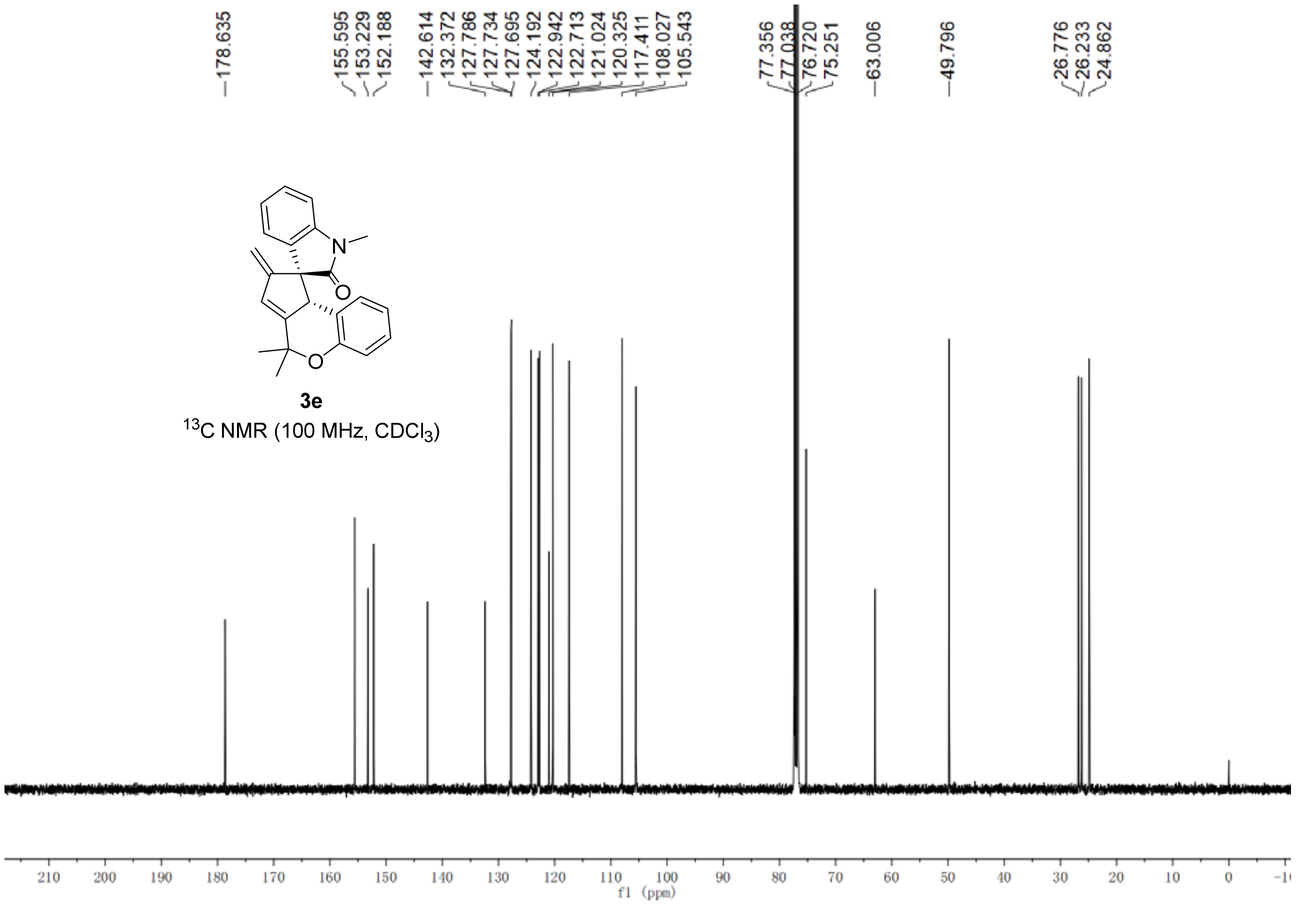

3e

^{13}C NMR (100 MHz, $CDCl_3$)

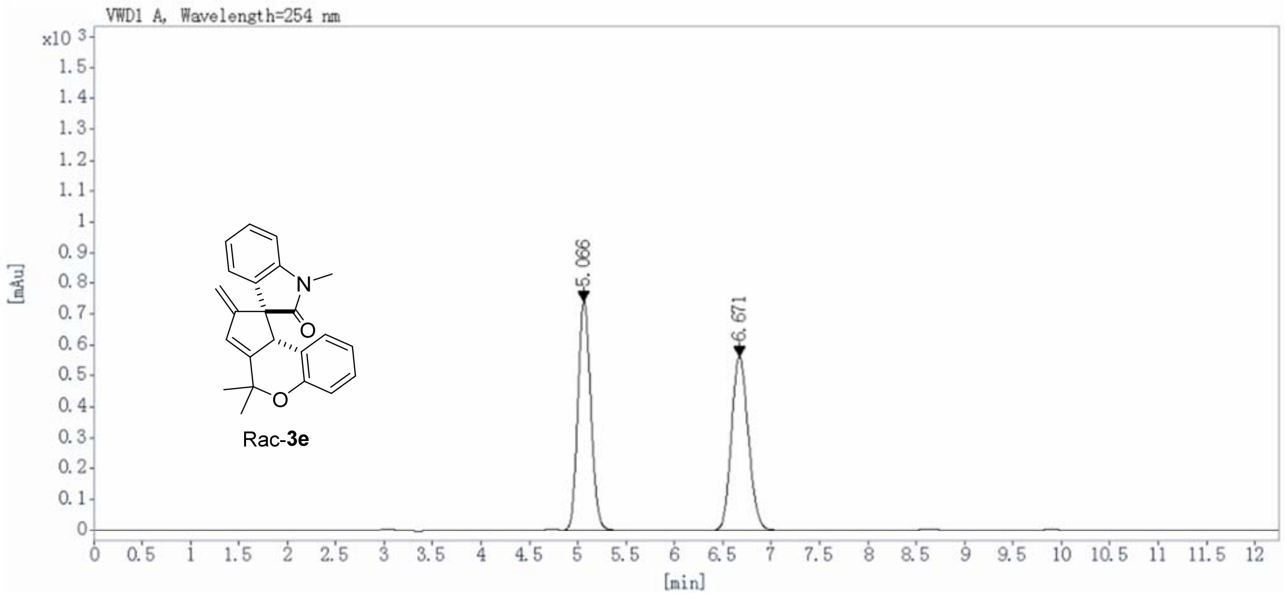

Ret Time [min]	Peak Type	Width [min]	Height [mAU]	Area [mAU*s]	Area [%]
5.066	BBA	0.15	741.8915	6873.9385	50.0958
6.671	BBA	0.19	561.2289	6847.6372	49.9042
			Totals:	13721.5757	100.0000

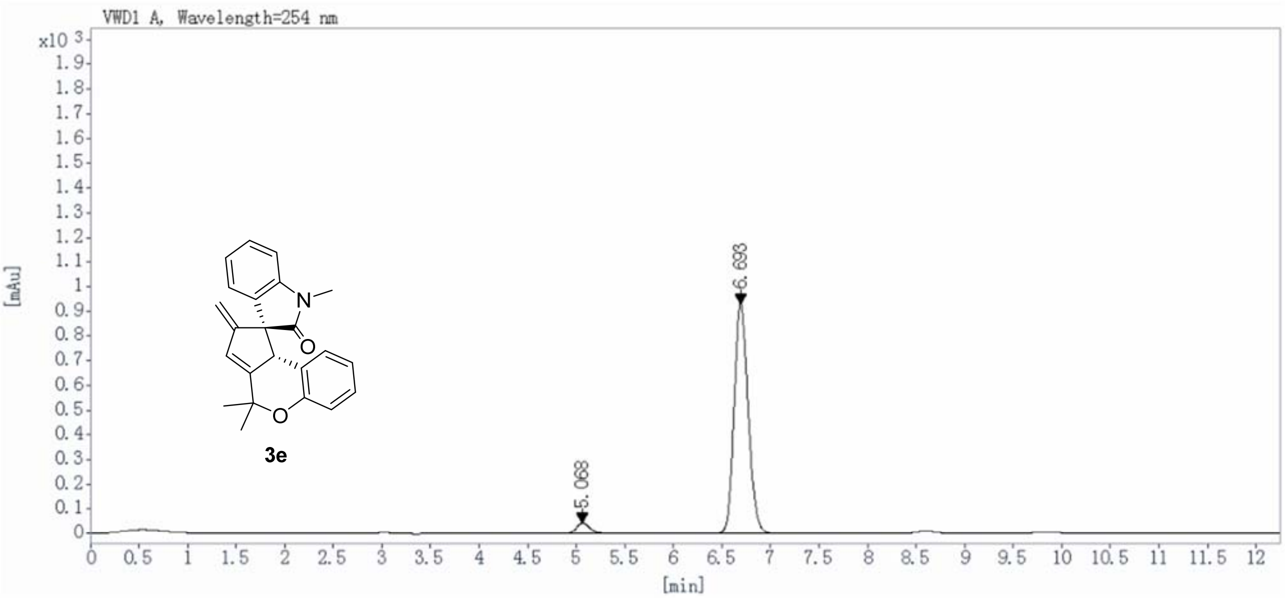

Ret Time [min]	Peak Type	Width [min]	Height [mAU]	Area [mAU*s]	Area [%]
5.068	BBA	0.15	41.3812	384.0868	3.9358
6.693	BBA	0.16	929.2313	9374.7246	96.0642
			Totals:	9758.8114	100.0000

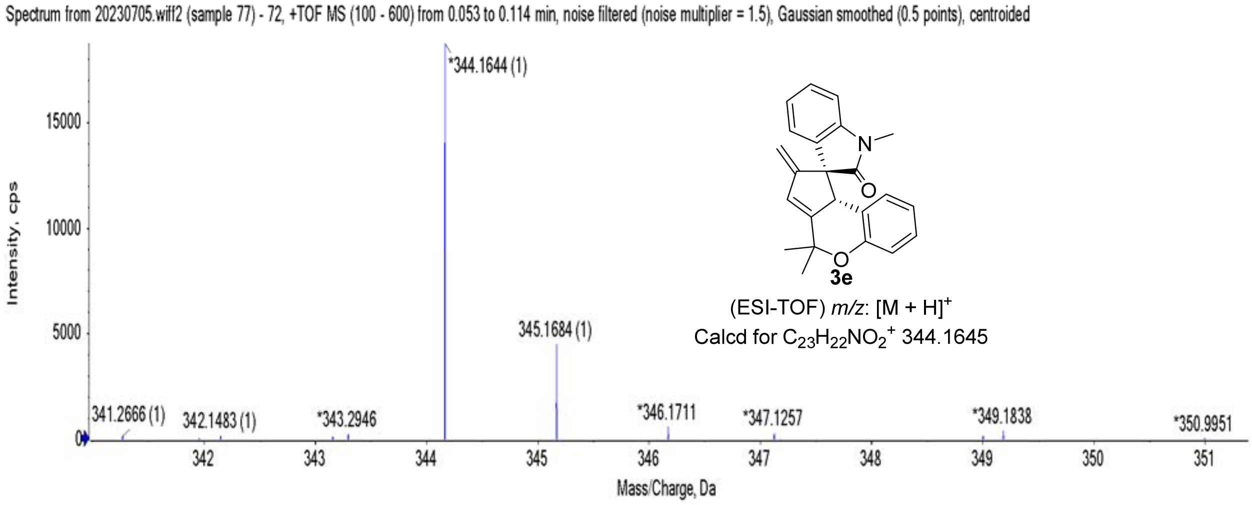
Spectrum from 20230705.wiff2 (sample 77) - 72, +TOF MS (100 - 600) from 0.053 to 0.114 min, noise filtered (noise multiplier = 1.5), Gaussian smoothed (0.5 points), centroided
*344.1644 (1)
15000
10000
5000
0
Intensity, cps
345.1684 (1)
341.2666 (1)
342.1483 (1)
*343.2946
*346.1711
*347.1257
*349.1838
*350.9951
342
343
344
345
346
347
348
349
350
351
Mass/Charge, Da
N
O
O
3e
(ESI-TOF) m/z: [M + H]+
Calcd for C23H22NO2+ 344.1645

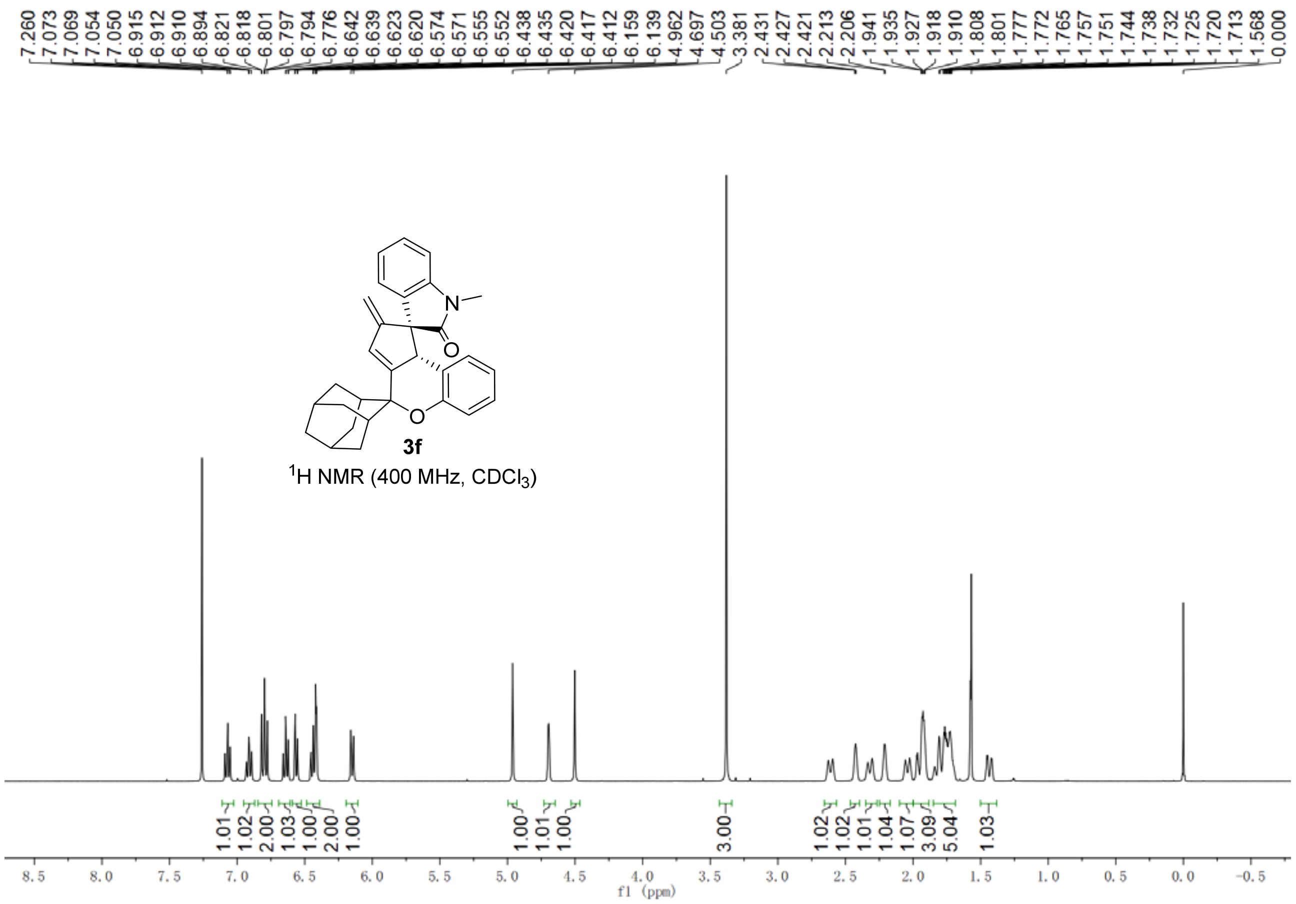
7.260 7.073 7.069 7.054 7.050 6.915 6.912 6.910 6.894 6.821 6.818 6.801 6.797 6.794 6.776 6.642 6.639 6.623 6.620 6.574 6.571 6.555 6.552 6.438 6.435 6.420 6.417 6.412 6.159 6.139 4.962 4.697 4.503 3.381 2.431 2.427 2.421 2.213 2.206 1.941 1.935 1.927 1.918 1.910 1.808 1.801 1.777 1.772 1.765 1.757 1.751 1.744 1.738 1.732 1.725 1.720 1.713 1.568 0.000
N
O
O
3f
1H NMR (400 MHz, CDCl3)
1.01 1.02 2.00 1.03 1.00 2.00 1.00 1.00 1.01 1.00 3.00 1.02 1.02 1.01 1.04 1.07 3.09 5.04 1.03
8.5 8.0 7.5 7.0 6.5 6.0 5.5 5.0 4.5 4.0 3.5 3.0 2.5 2.0 1.5 1.0 0.5 0.0 -0.5
f1 (ppm)

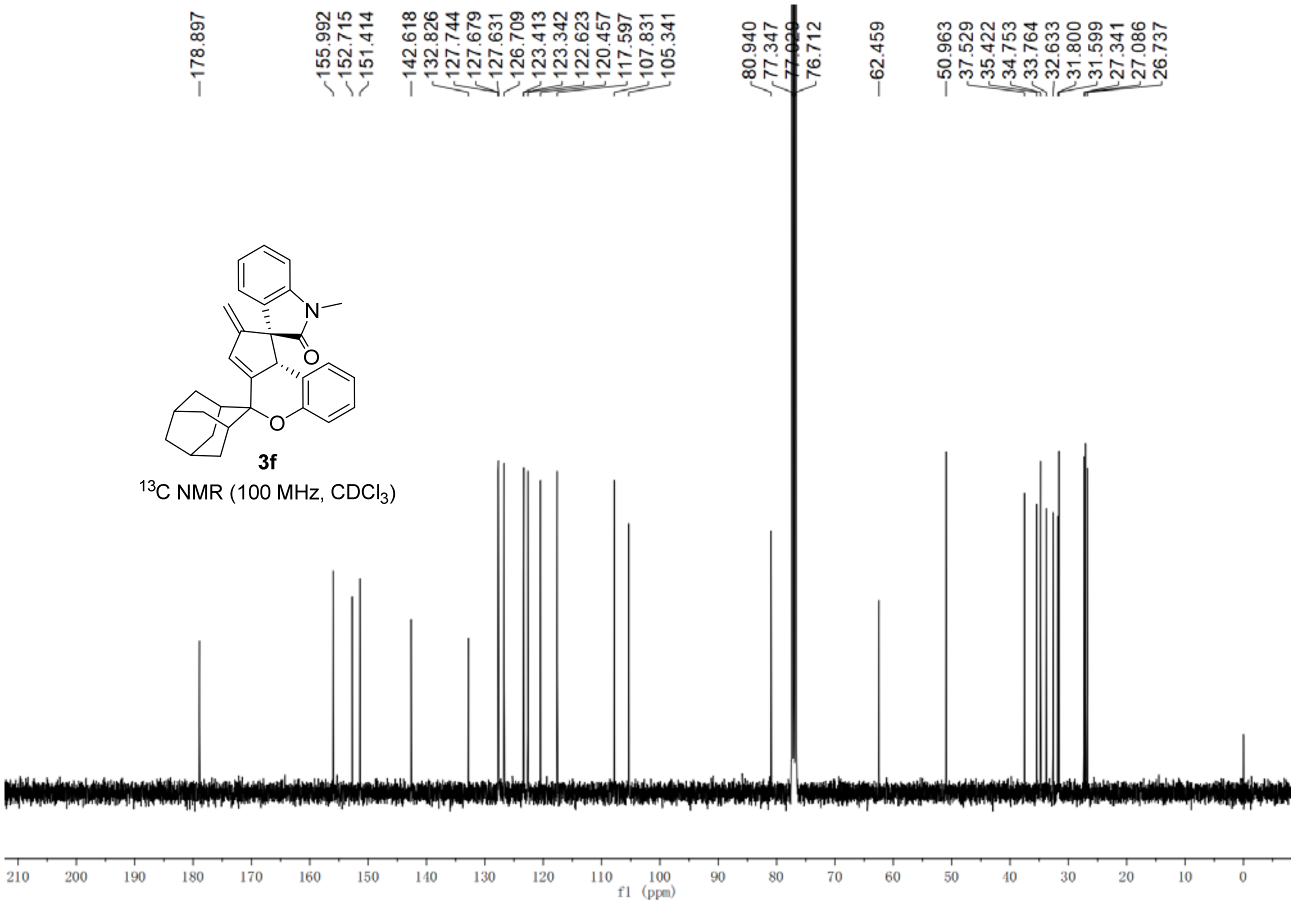
178.897 155.992 152.715 151.414 142.618 132.826 127.744 127.679 127.631 126.709 123.413 123.342 122.623 120.457 117.597 107.831 105.341 80.940 77.347 77.0[illegible] 76.712 62.459 50.963 37.529 35.422 34.753 33.764 32.633 31.800 31.599 27.341 27.086 26.737
N
O
O
3f
13C NMR (100 MHz, CDCl3)
210 200 190 180 170 160 150 140 130 120 110 100 90 80 70 60 50 40 30 20 10 0
f1 (ppm)

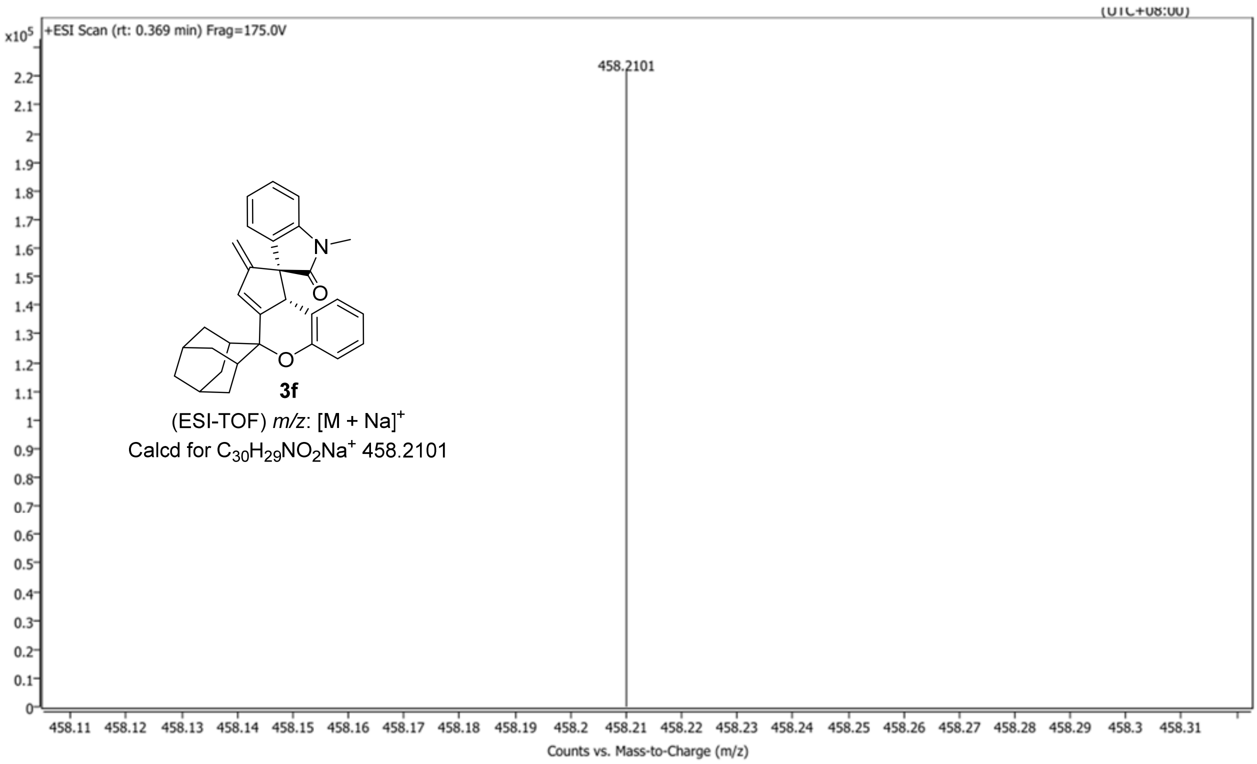
(UTC+08:00)
x10^5
+ESI Scan (rt: 0.369 min) Frag=175.0V
458.2101
N
O
O
3f
(ESI-TOF) m/z: [M + Na]+
Calcd for C30H29NO2Na+ 458.2101
Counts vs. Mass-to-Charge (m/z)

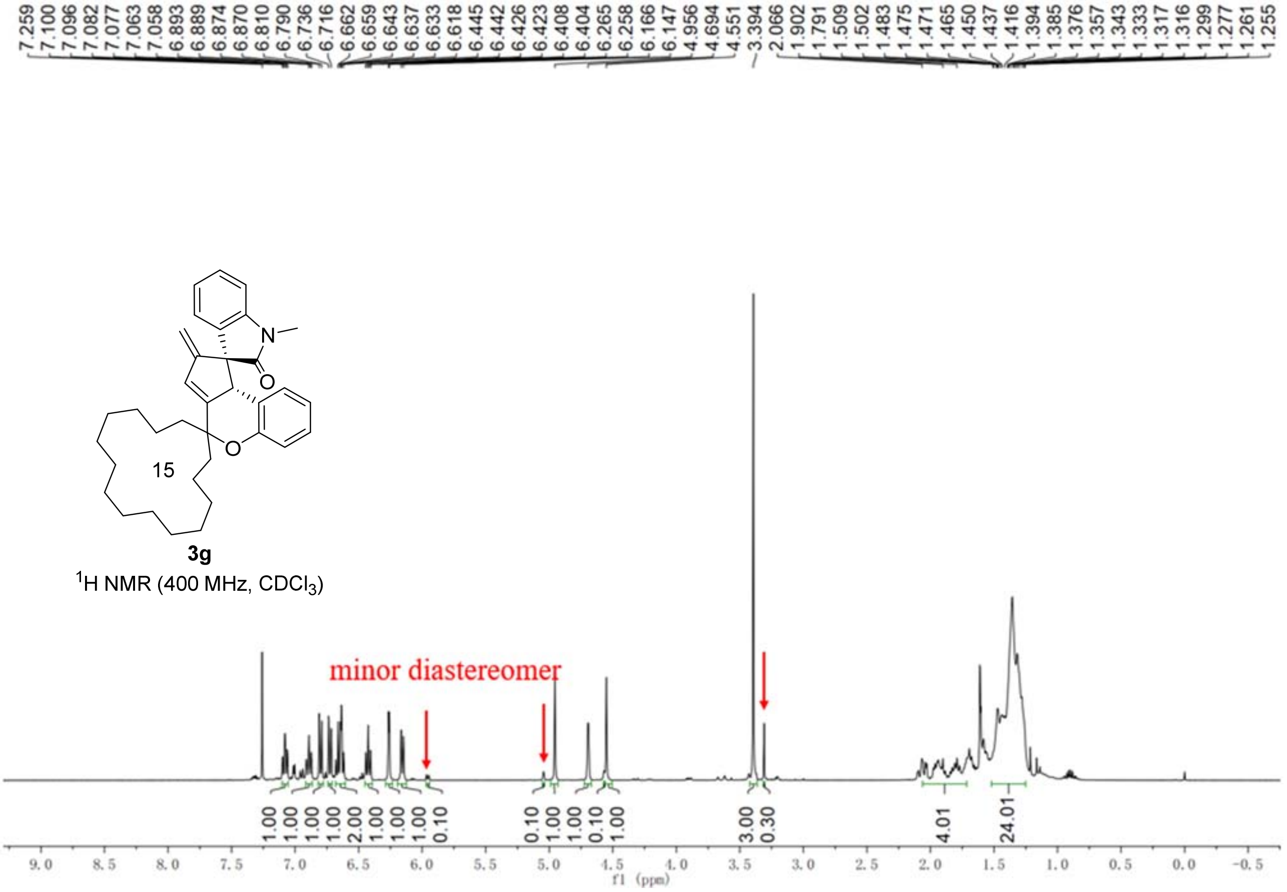

3g

^{1}H NMR (400 MHz, $CDCl_3$)

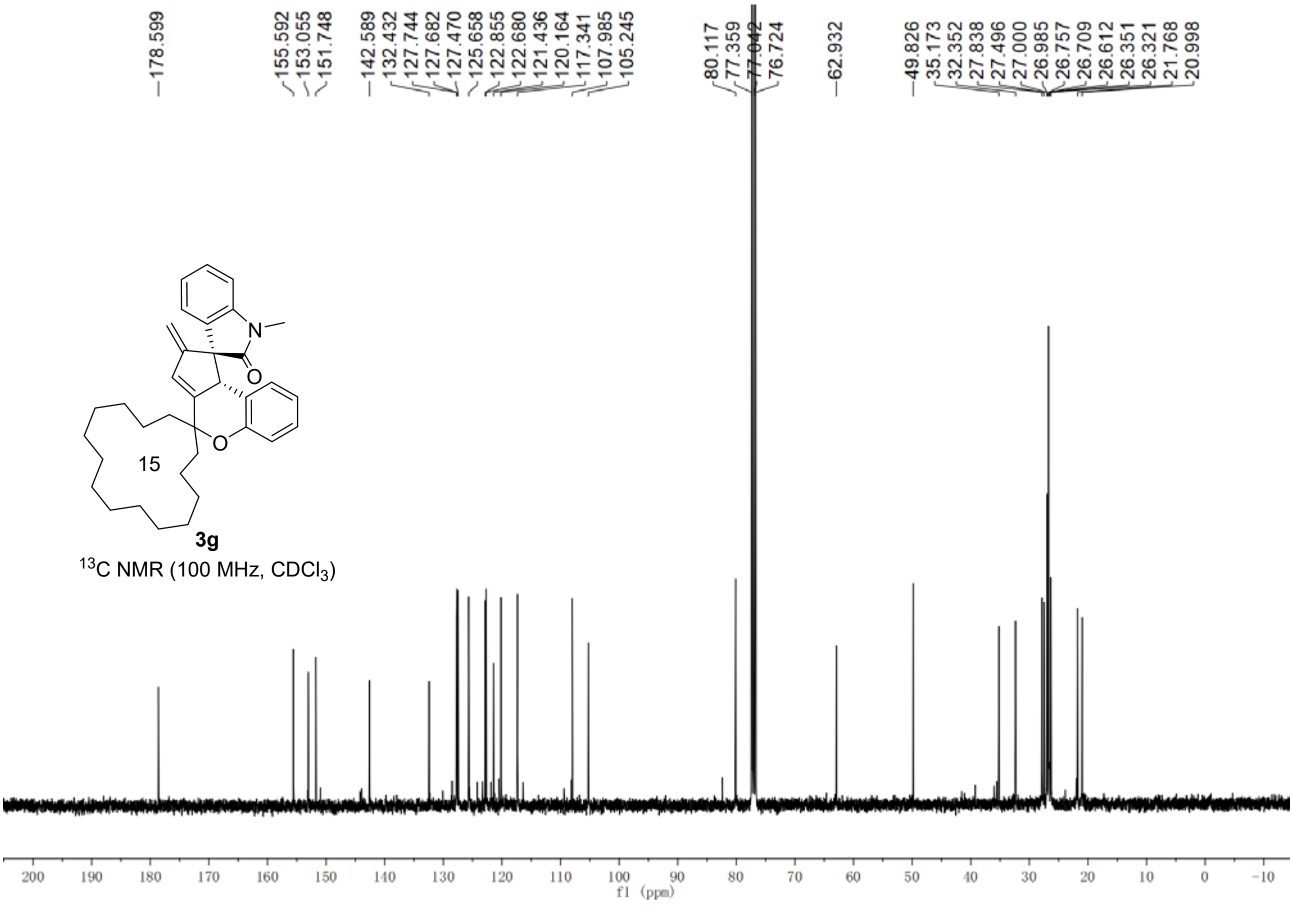

3g

^{13}C NMR (100 MHz, $CDCl_3$)

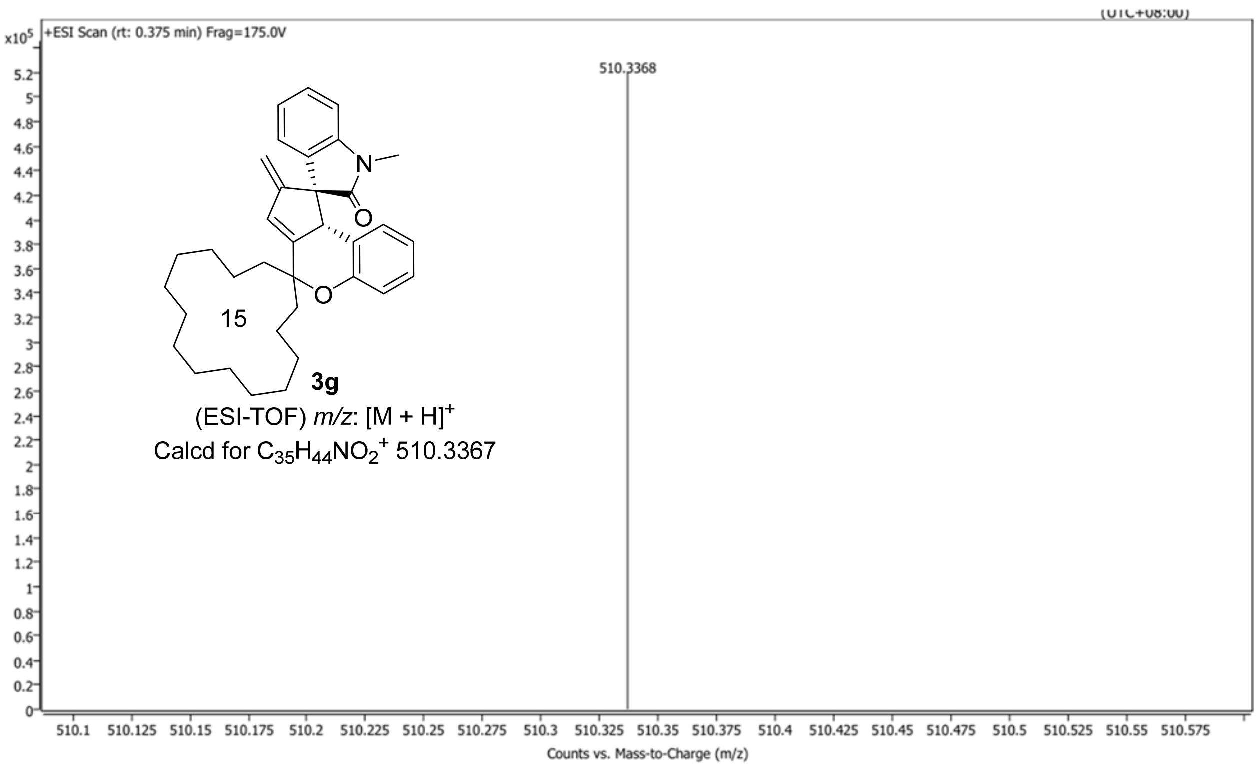
(UTC+08:00)
x10^5
+ESI Scan (rt: 0.375 min) Frag=175.0V
510.3368
N
O
O
15
3g
(ESI-TOF) m/z: [M + H]+
Calcd for C35H44NO2+ 510.3367
Counts vs. Mass-to-Charge (m/z)

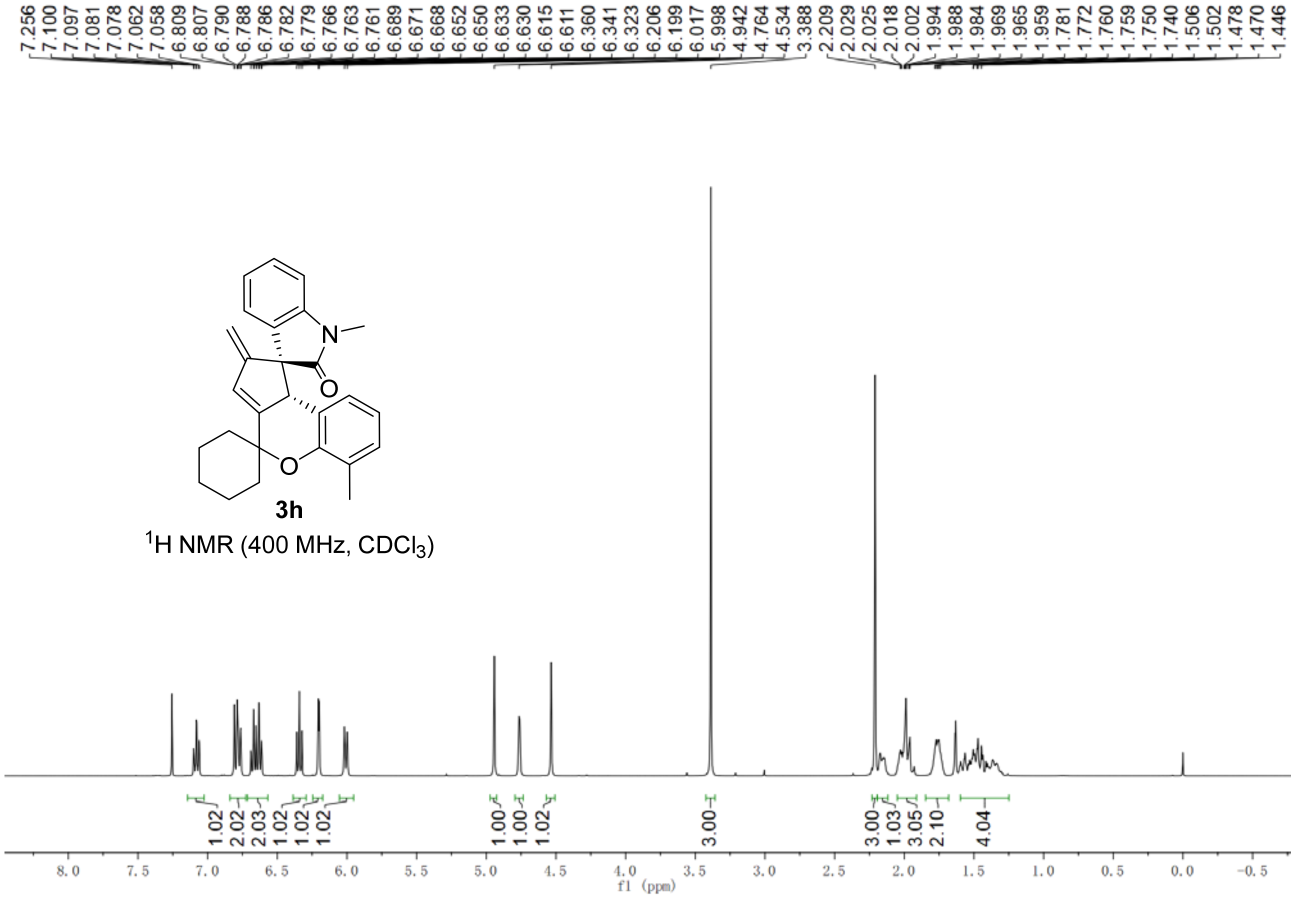

3h

^1H NMR (400 MHz, $CDCl_3$)

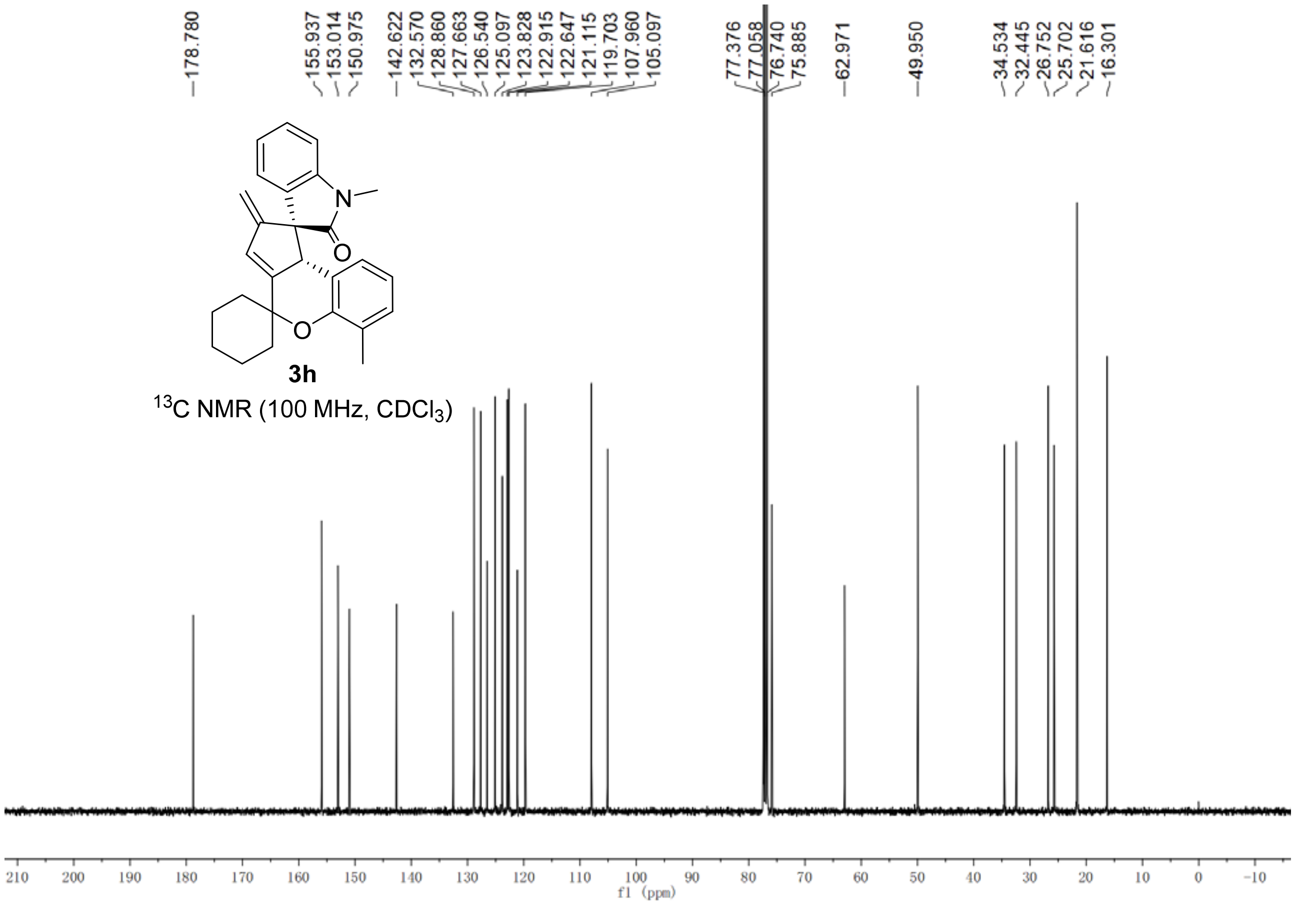

3h

^{13}C NMR (100 MHz, $CDCl_3$)

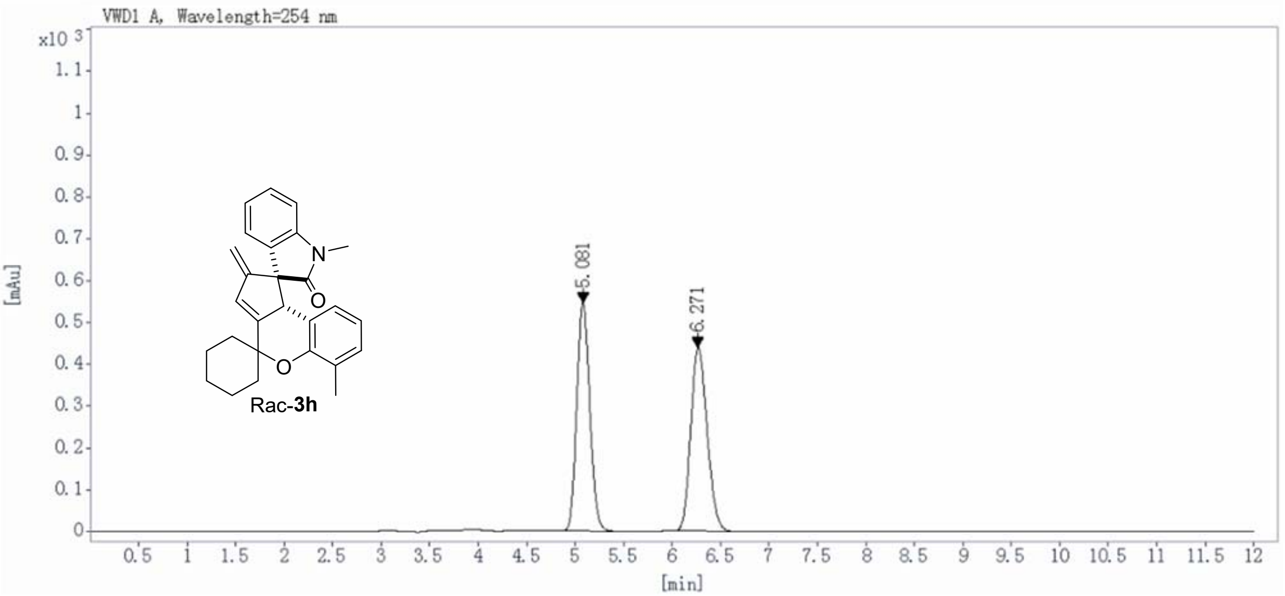

Ret Time [min]	Peak Type	Width [min]	Height [mAU]	Area [mAU*s]	Area [%]
5.081	BBA	0.15	546.0235	5105.6519	49.6539
6.271	BBA	0.18	438.4892	5176.8340	50.3461
			Totals:	10282.4858	100.0000

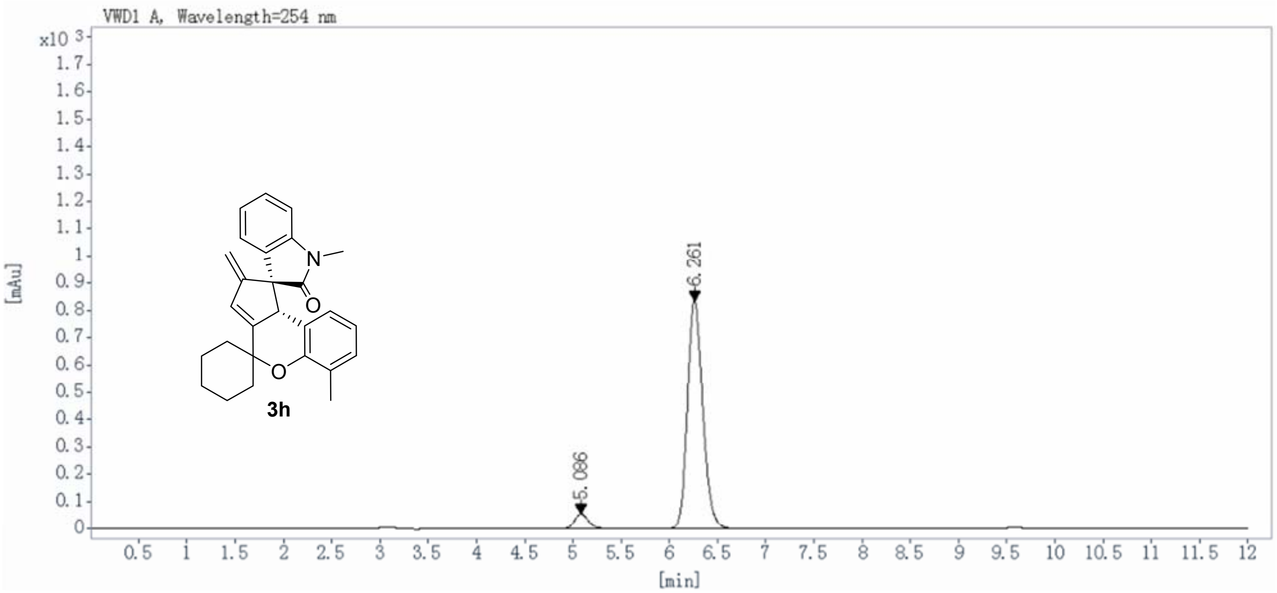

Ret Time [min]	Peak Type	Width [min]	Height [mAU]	Area [mAU*s]	Area [%]
5.086	BBA	0.15	52.6610	507.6499	5.2306
6.261	BBA	0.17	833.6223	9197.7656	94.7694
			Totals:	9705.4156	100.0000

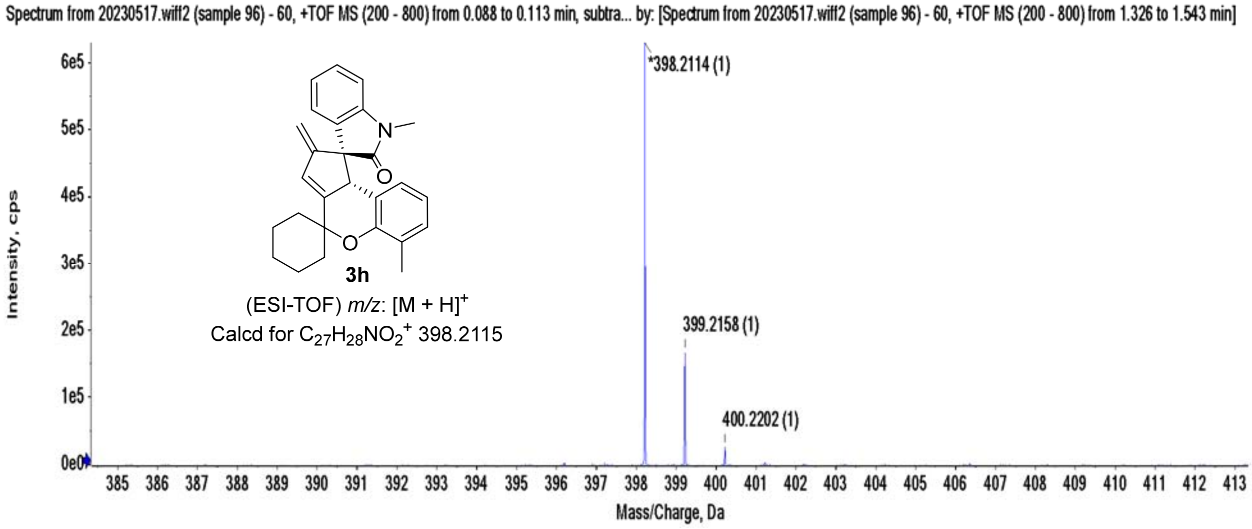
Spectrum from 20230517.wiff2 (sample 96) - 60, +TOF MS (200 - 800) from 0.088 to 0.113 min, subtra... by: [Spectrum from 20230517.wiff2 (sample 96) - 60, +TOF MS (200 - 800) from 1.326 to 1.543 min]
*398.2114 (1)
399.2158 (1)
400.2202 (1)
Intensity, cps
Mass/Charge, Da
3h
(ESI-TOF) m/z: [M + H]+
Calcd for C27H28NO2+ 398.2115

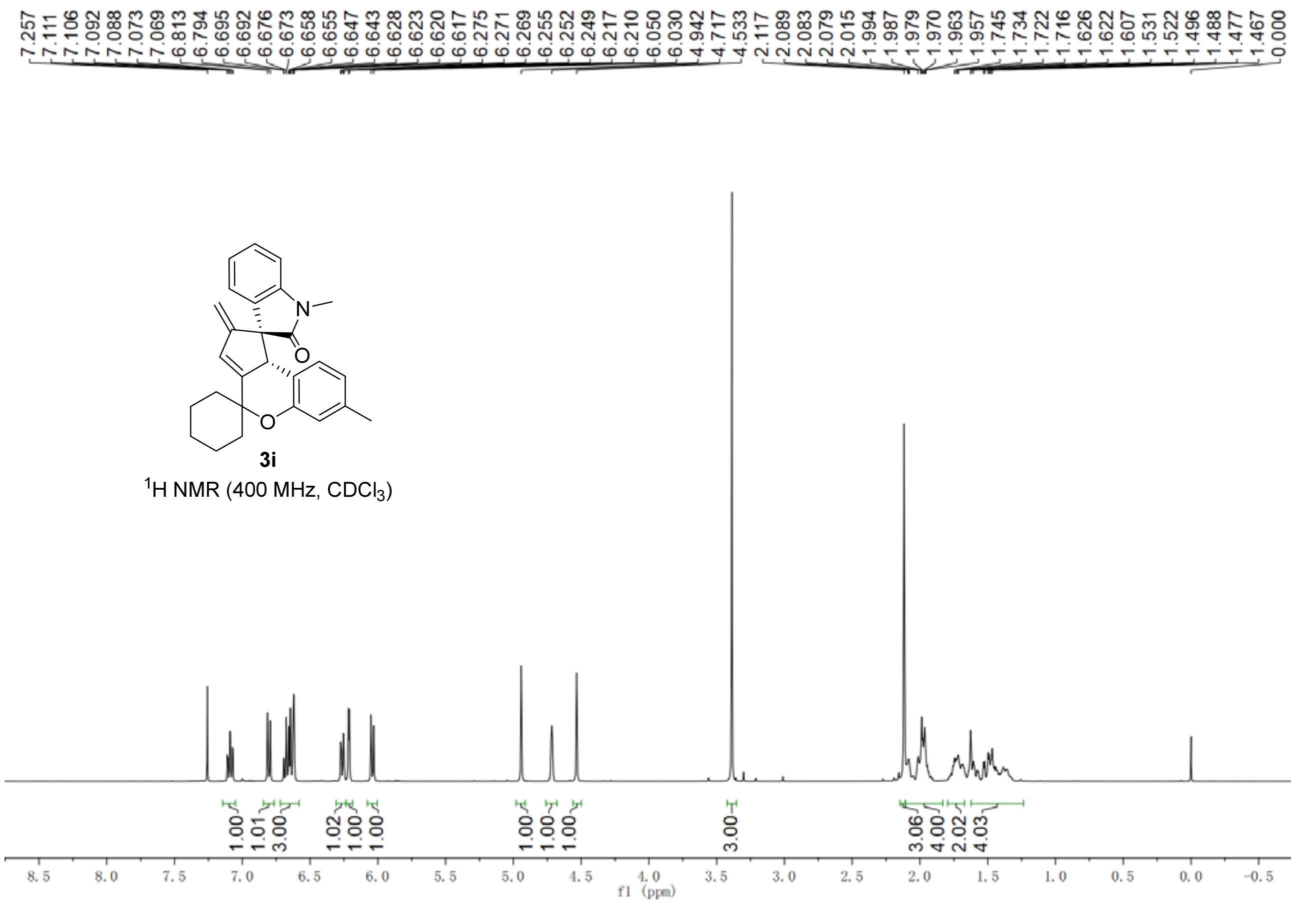
7.257 7.111 7.106 7.092 7.088 7.073 7.069 6.813 6.794 6.695 6.692 6.676 6.673 6.658 6.655 6.647 6.643 6.628 6.623 6.620 6.617 6.275 6.271 6.269 6.255 6.252 6.249 6.217 6.210 6.050 6.030 4.942 4.717 4.533 2.117 2.089 2.083 2.079 2.015 1.994 1.987 1.979 1.970 1.963 1.957 1.745 1.734 1.722 1.716 1.626 1.622 1.607 1.531 1.522 1.496 1.488 1.477 1.467 0.000
N
O
O
3i
^{1}H NMR (400 MHz, $CDCl_3$)
1.00 1.01 3.00 1.02 1.00 1.00 1.00 1.00 1.00 3.00 3.06 4.00 2.02 4.03
f1 (ppm)

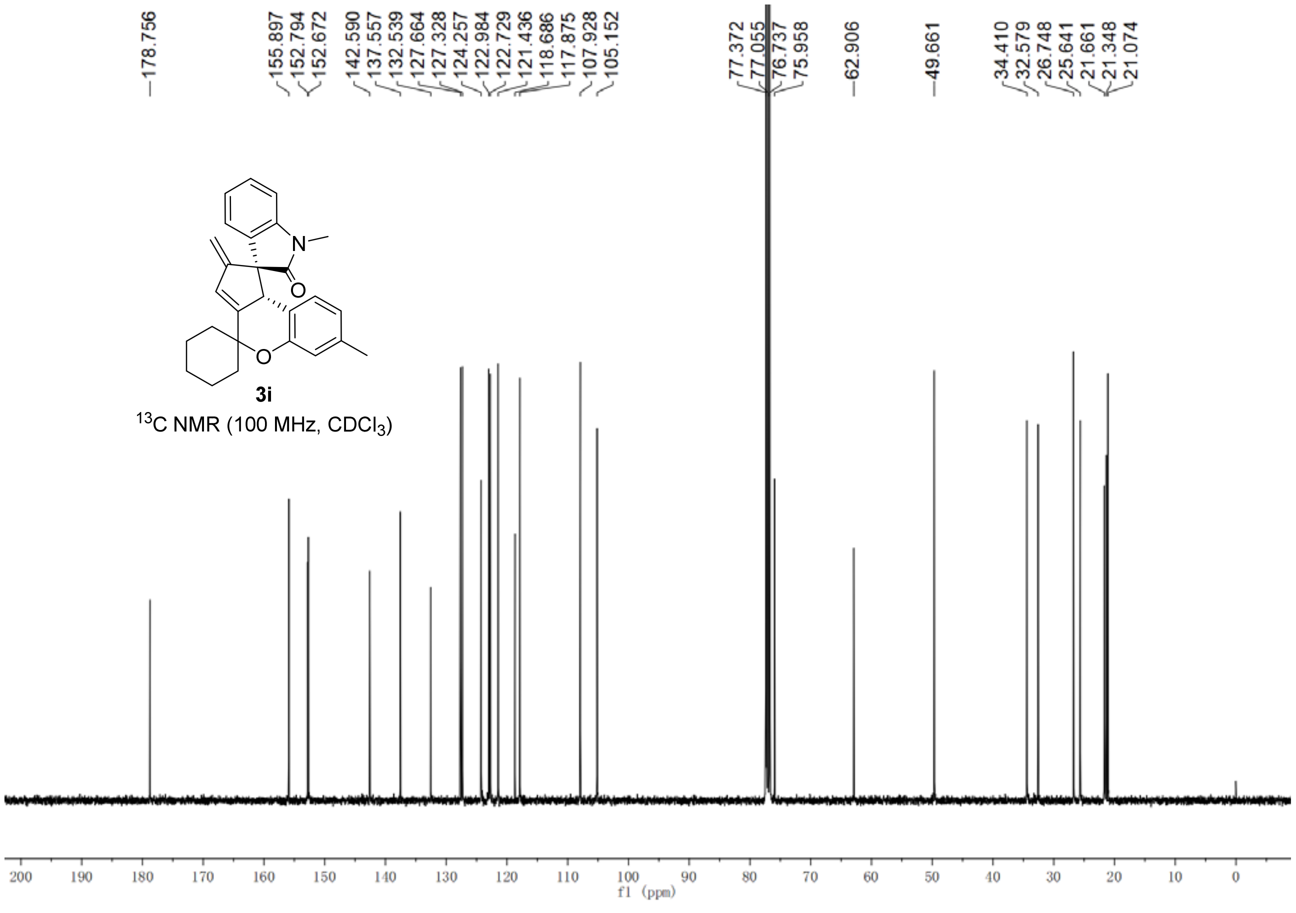
178.756
155.897 152.794 152.672 142.590 137.557 132.539 127.664 127.328 124.257 122.984 122.729 121.436 118.686 117.875 107.928 105.152
77.372 77.055 76.737 75.958 62.906 49.661 34.410 32.579 26.748 25.641 21.661 21.348 21.074
N
O
O
3i
^{13}C NMR (100 MHz, $CDCl_3$)
f1 (ppm)

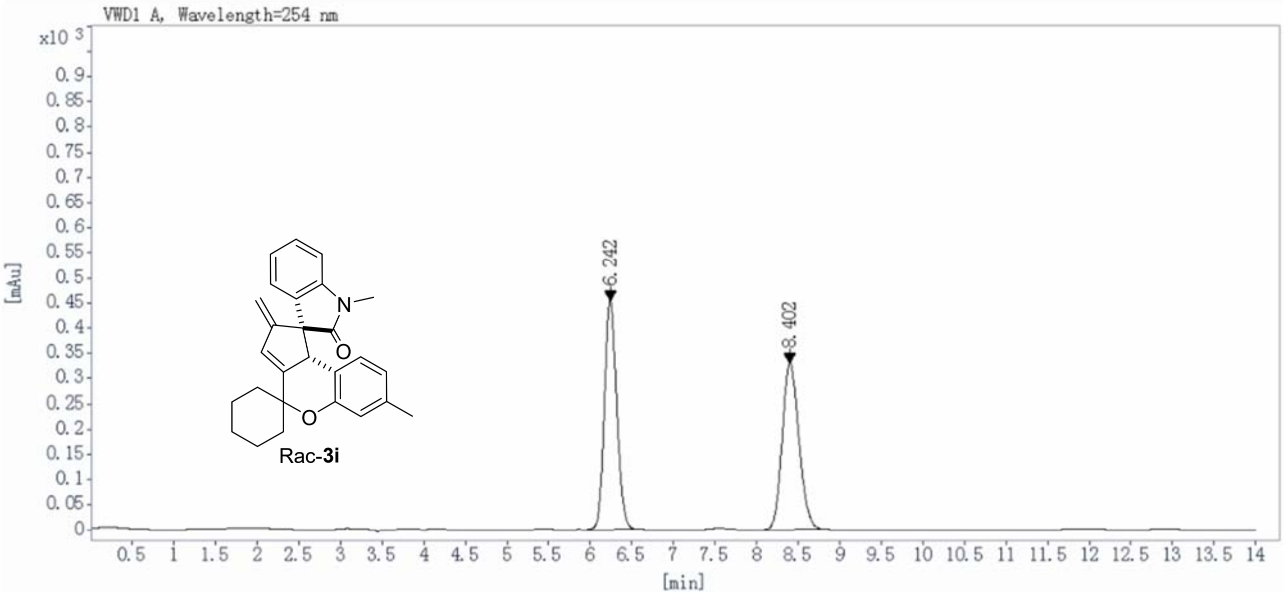

Ret Time [min]	Peak Type	Width [min]	Height [mAU]	Area [mAU*s]	Area [%]
6.242	BBA	0.16	454.1411	4702.7432	50.2738
8.402	BBA	0.22	330.2756	4651.5200	49.7262
			Totals:	9354.2632	100.0000

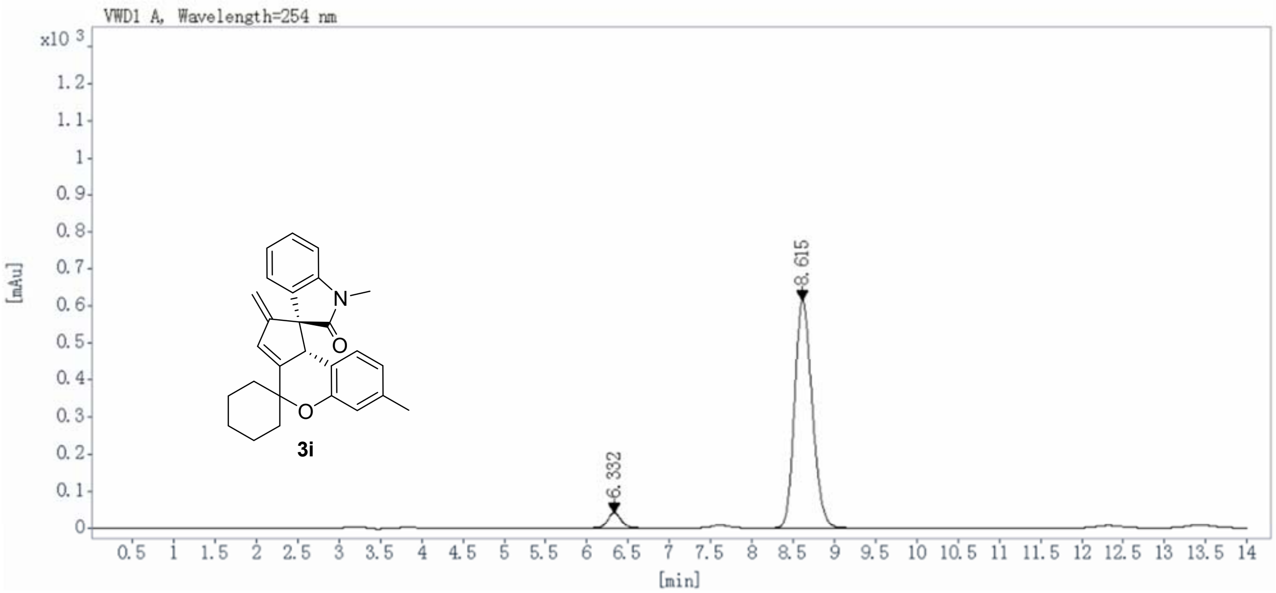

Ret Time [min]	Peak Type	Width [min]	Height [mAU]	Area [mAU*s]	Area [%]
6.332	BBA	0.17	41.2327	461.6652	4.8121
8.615	BBA	0.23	614.2065	9132.0762	95.1879
			Totals:	9593.7414	100.0000

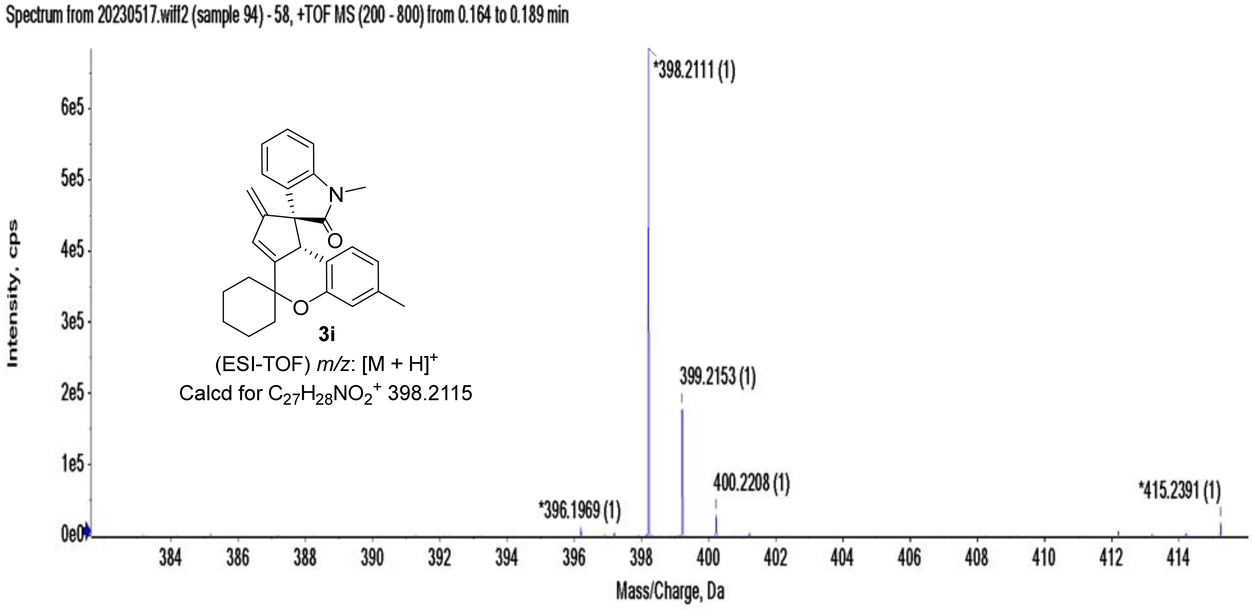
Spectrum from 20230517.wiff2 (sample 94) - 58, +TOF MS (200 - 800) from 0.164 to 0.189 min
Intensity, cps
6e5
5e5
4e5
3e5
2e5
1e5
0e0
*398.2111 (1)
399.2153 (1)
400.2208 (1)
*396.1969 (1)
*415.2391 (1)
384
386
388
390
392
394
396
398
400
402
404
406
408
410
412
414
Mass/Charge, Da
N
O
O
3i
(ESI-TOF) m/z: [M + H]+
Calcd for C27H28NO2+ 398.2115

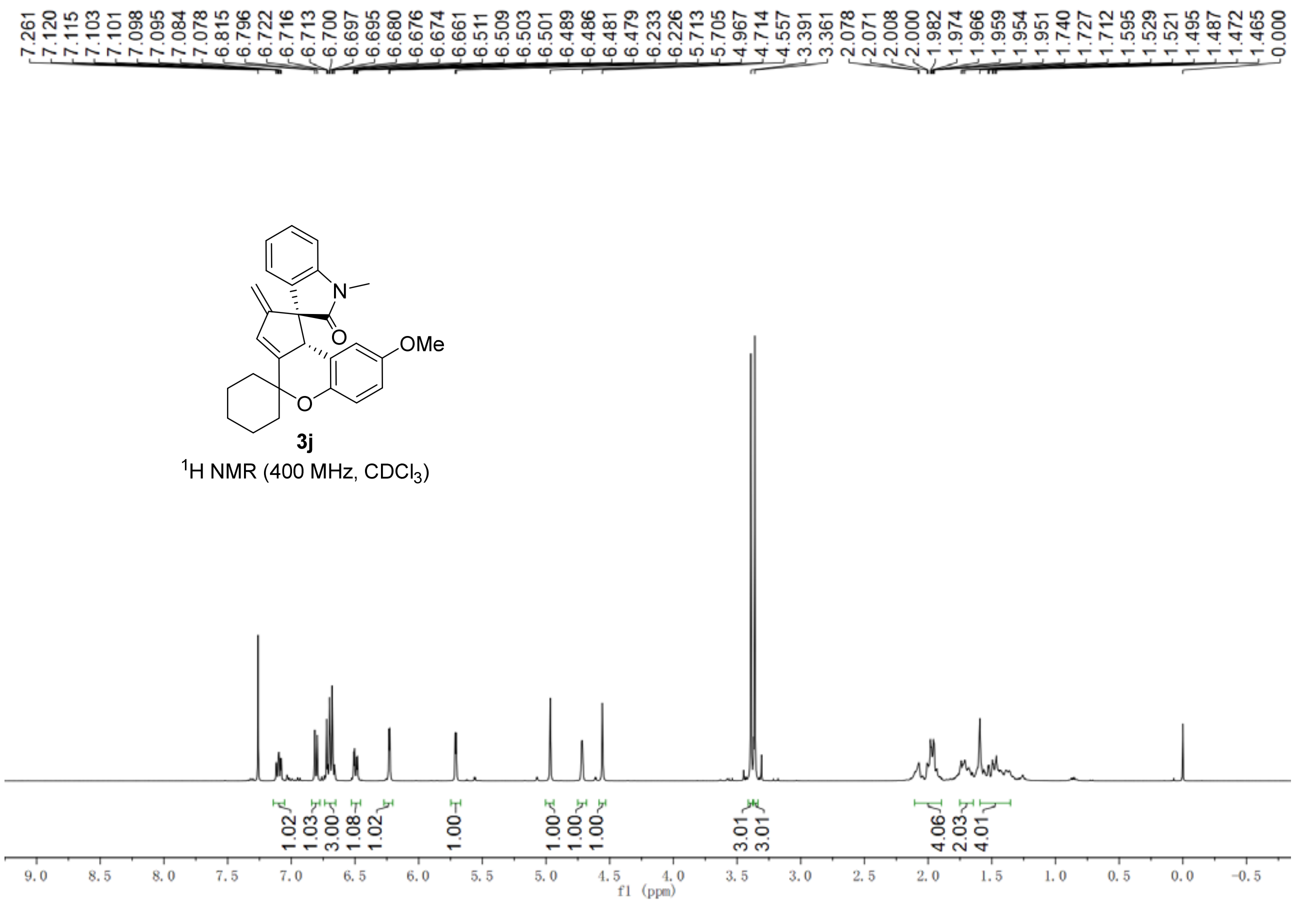

3j

^{1}H NMR (400 MHz, $CDCl_3$)

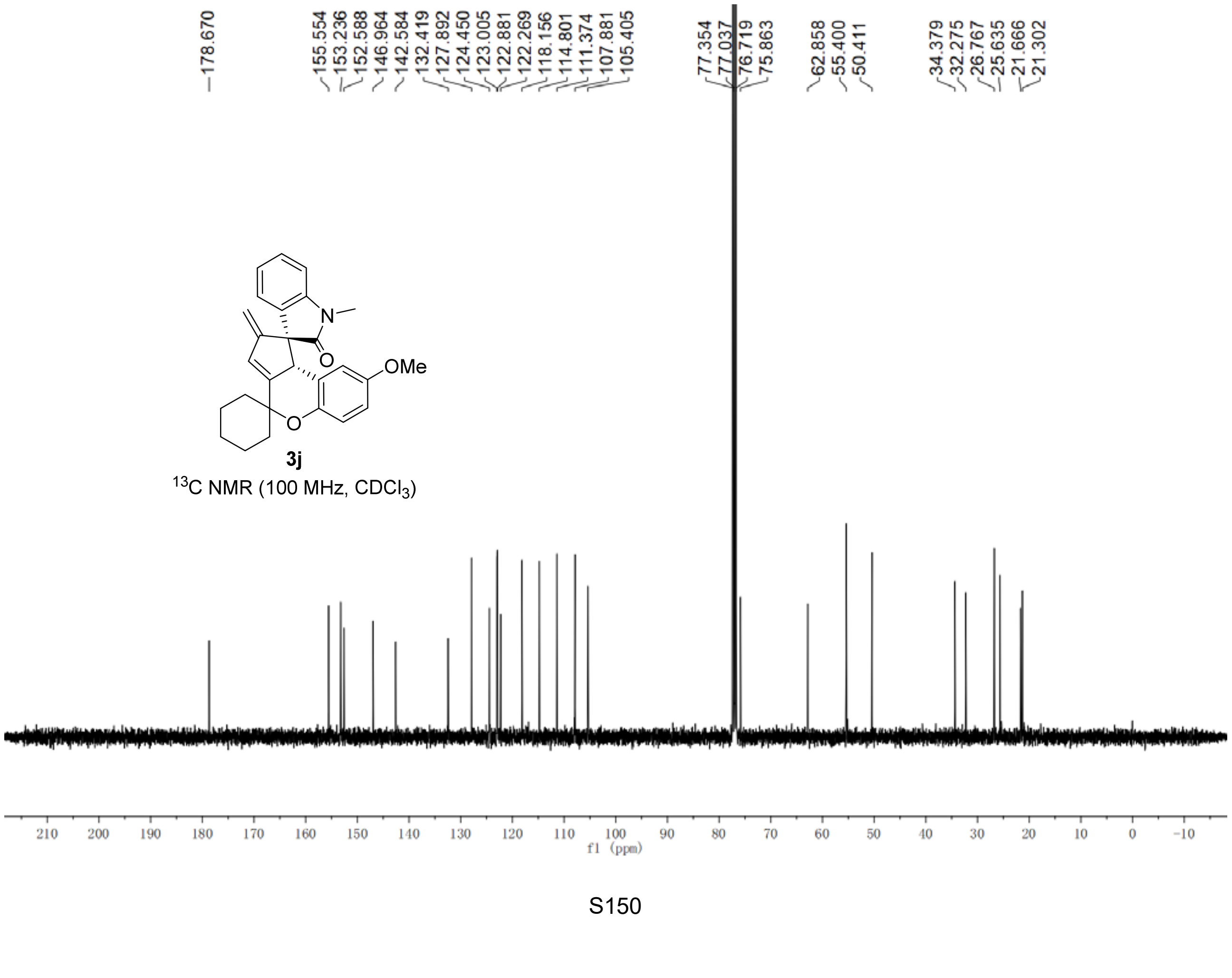

3j

^{13}C NMR (100 MHz, $CDCl_3$)

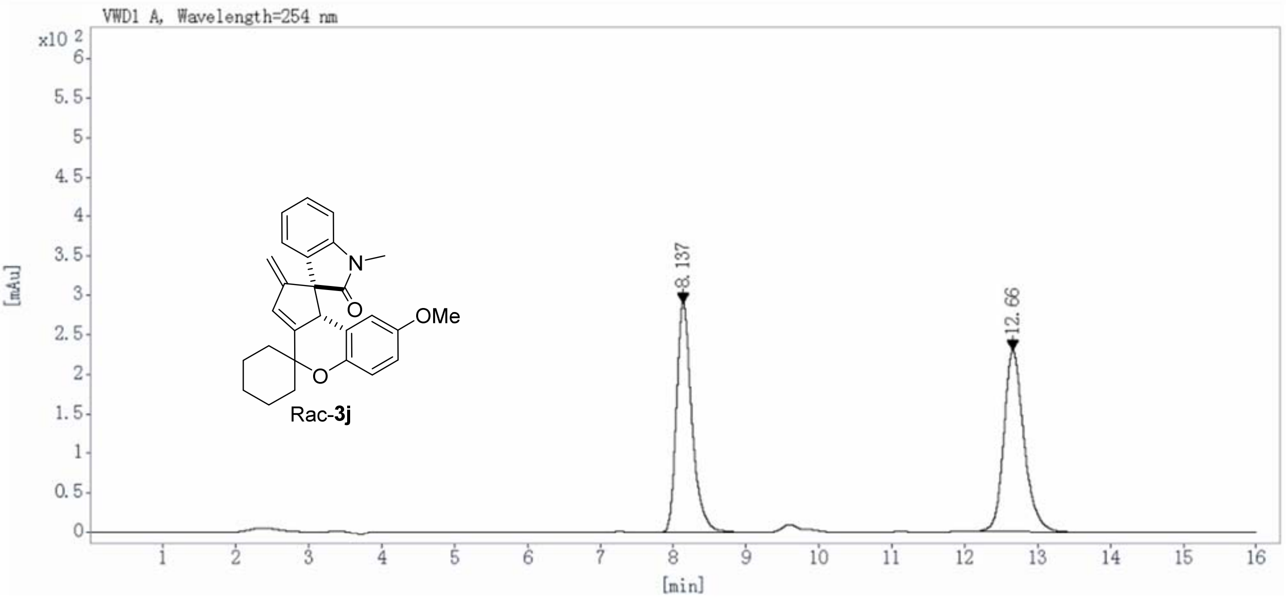

Ret Time [min]	Peak Type	Width [min]	Height [mAU]	Area [mAU*s]	Area [%]
8.137	BBA	0.22	289.9394	4266.7715	49.1870
12.660	BBA	0.29	229.3670	4407.8218	50.8130
			Totals:	8674.5933	100.0000

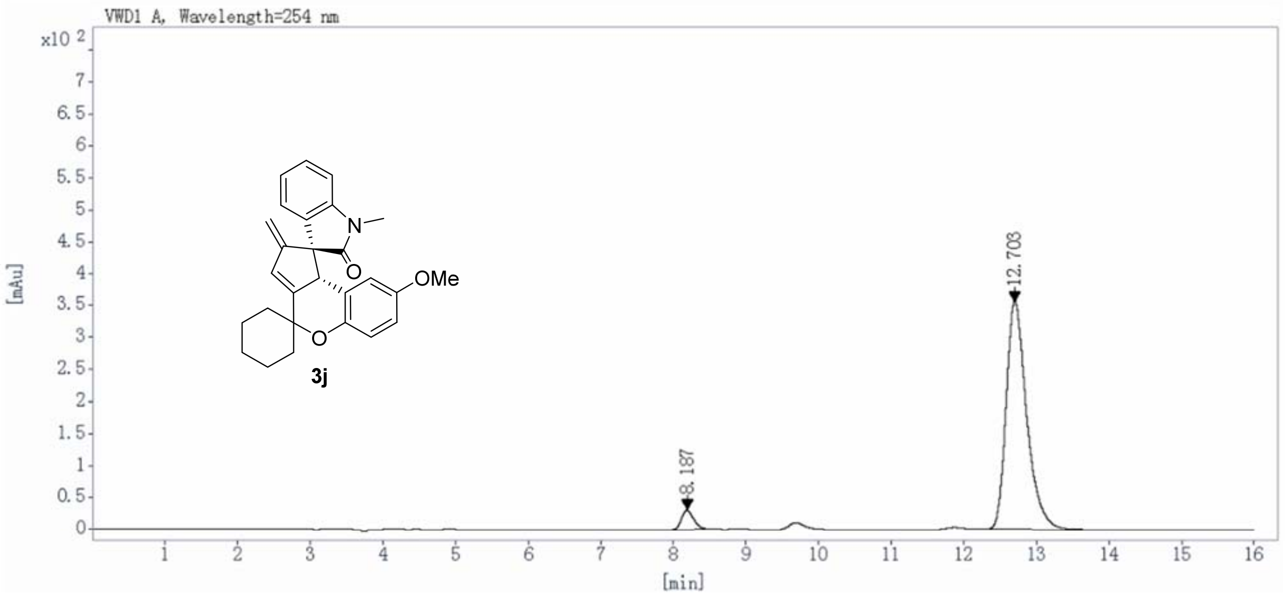

Ret Time [min]	Peak Type	Width [min]	Height [mAU]	Area [mAU*s]	Area [%]
8.187	BBA	0.18	31.2825	376.2105	5.0857
12.703	BBA	0.30	356.7078	7021.1616	94.9143
			Totals:	7397.3722	100.0000

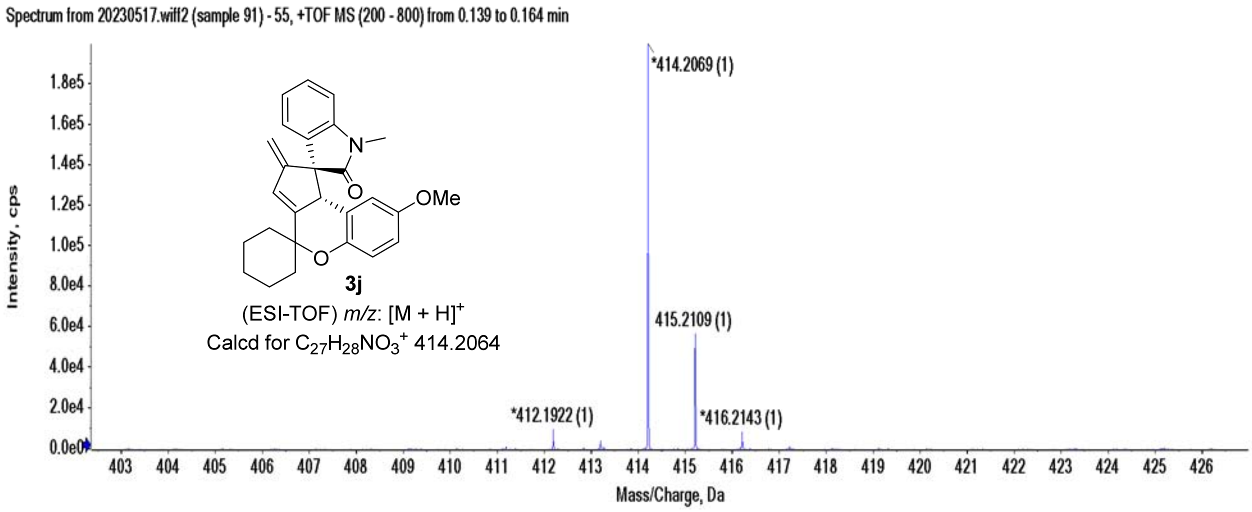
Spectrum from 20230517.wiff2 (sample 91) - 55, +TOF MS (200 - 800) from 0.139 to 0.164 min
Intensity, cps
Mass/Charge, Da
*414.2069 (1)
415.2109 (1)
*412.1922 (1)
*416.2143 (1)
OMe
N
O
O
3j
(ESI-TOF) m/z: [M + H]+
Calcd for C27H28NO3+ 414.2064

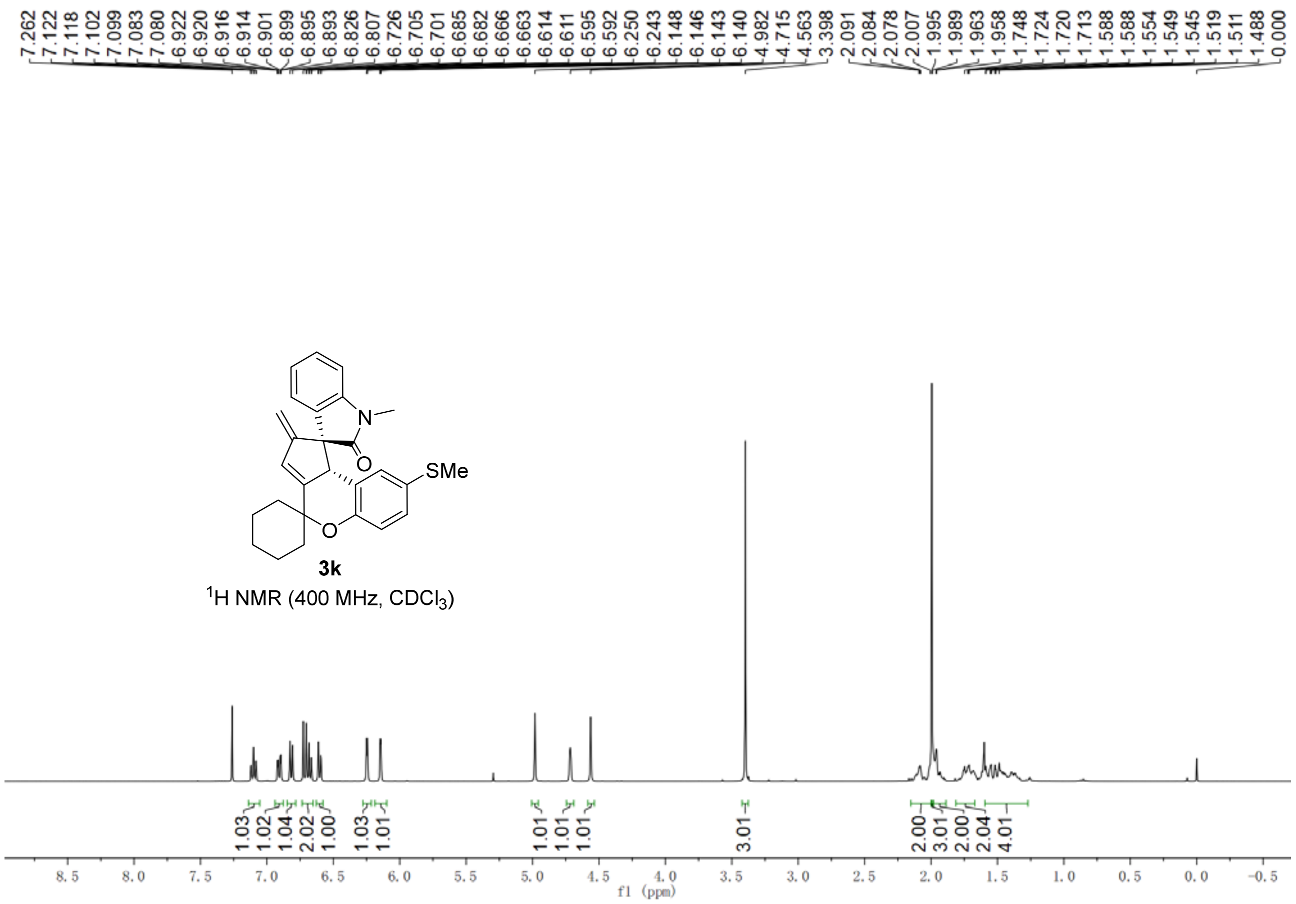
7.262
7.122
7.118
7.102
7.099
7.083
7.080
6.922
6.920
6.916
6.914
6.901
6.899
6.895
6.893
6.826
6.807
6.726
6.705
6.701
6.685
6.682
6.666
6.663
6.614
6.611
6.595
6.592
6.250
6.243
6.148
6.146
6.143
6.140
4.982
4.715
4.563
3.398
2.091
2.084
2.078
2.007
1.995
1.989
1.963
1.958
1.748
1.724
1.720
1.713
1.588
1.554
1.549
1.545
1.519
1.511
1.488
0.000
SMe
N
O
O
3k
^{1}H NMR (400 MHz, CDCl$_3$)
1.03
1.02
1.04
2.02
1.00
1.03
1.01
1.01
1.01
1.01
3.01
2.00
3.01
2.00
2.04
4.01
f1 (ppm)

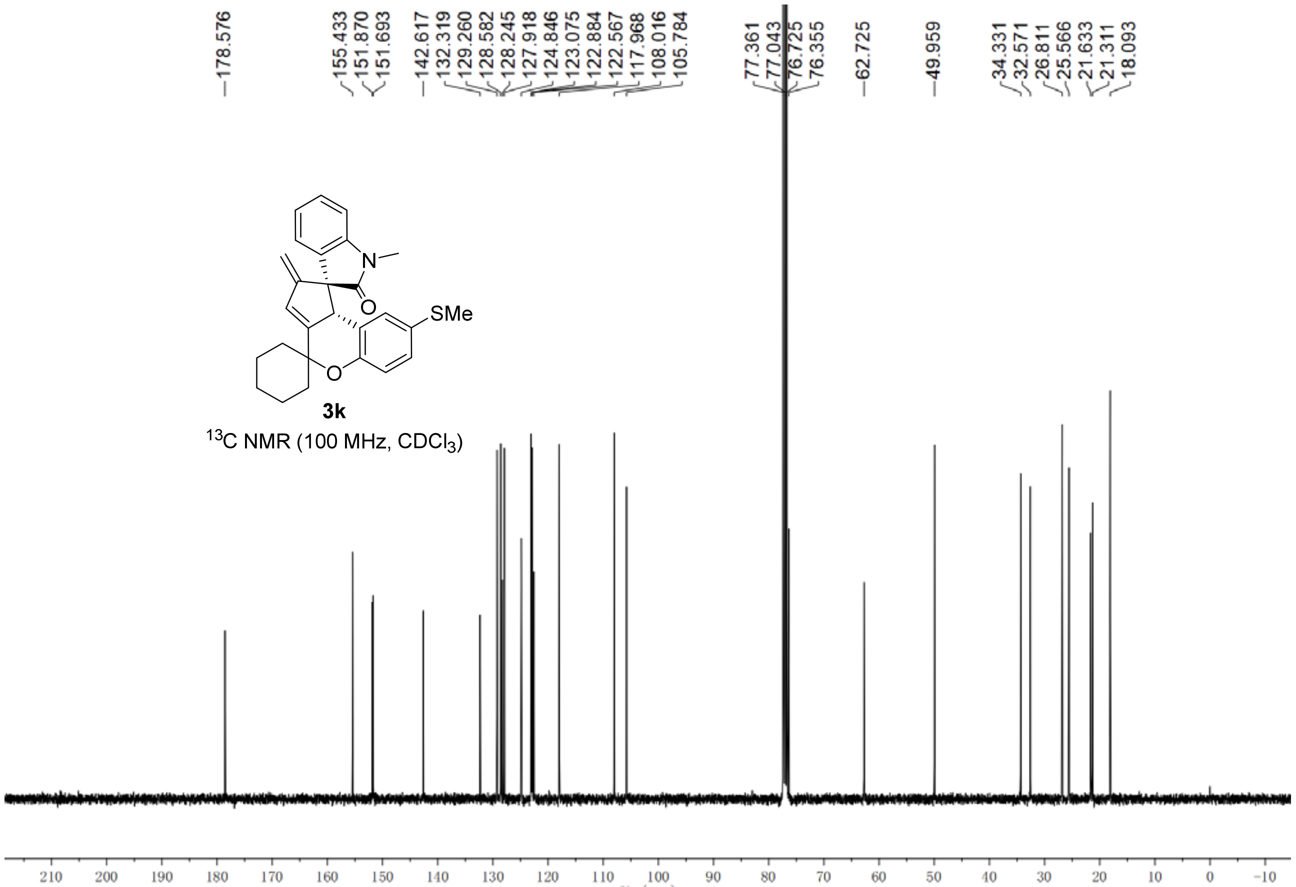
178.576
155.433
151.870
151.693
142.617
132.319
129.260
128.582
128.245
127.918
124.846
123.075
122.884
122.567
117.968
108.016
105.784
77.361
77.043
76.725
76.355
62.725
49.959
34.331
32.571
26.811
25.566
21.633
21.311
18.093
SMe
N
O
O
3k
^{13}C NMR (100 MHz, CDCl$_3$)
f1 (ppm)

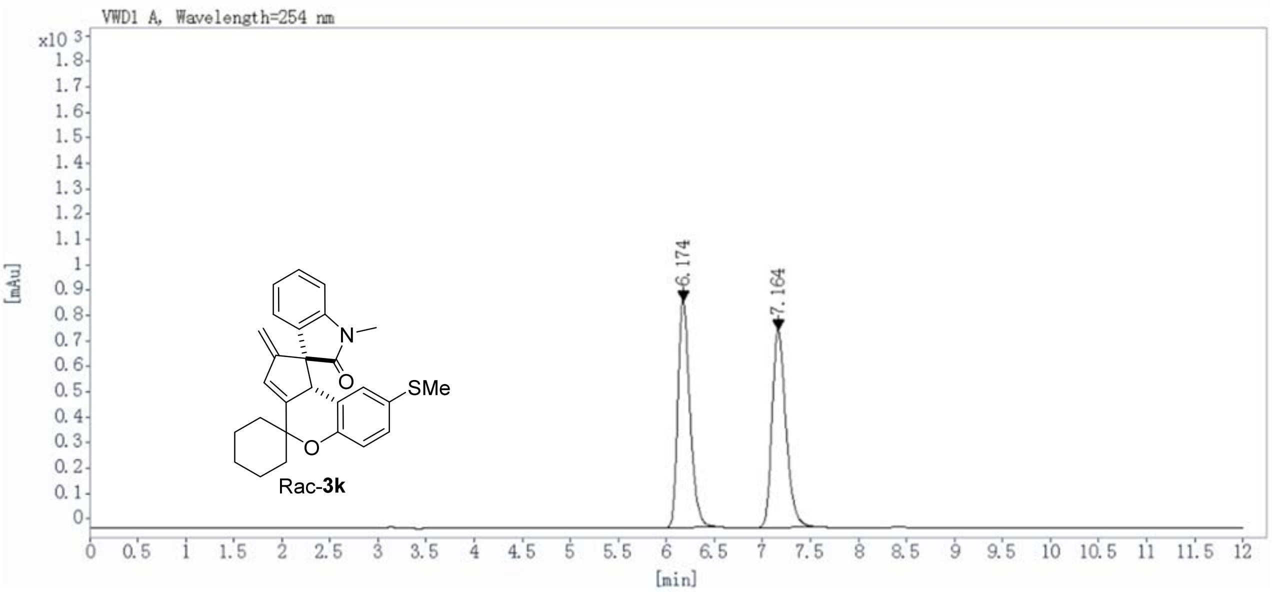

Ret Time [min]	Peak Type	Width [min]	Height [mAU]	Area [mAU*s]	Area [%]
6.174	BBA	0.13	892.4514	7819.1255	49.9049
7.164	BBA	0.15	777.7509	7848.9243	50.0951
			Totals:	15668.0498	100.0000

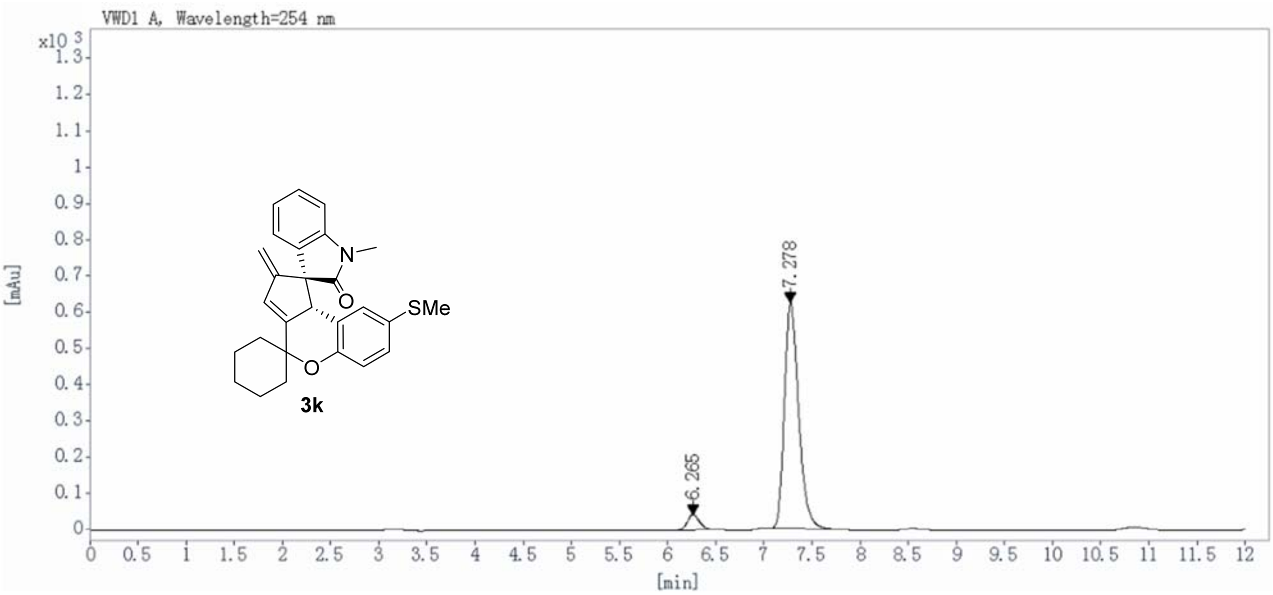

Ret Time [min]	Peak Type	Width [min]	Height [mAU]	Area [mAU*s]	Area [%]
6.265	BBA	0.14	42.4012	373.2176	5.4791
7.278	BBA	0.16	623.3853	6438.4448	94.5209
			Totals:	6811.6624	100.0000

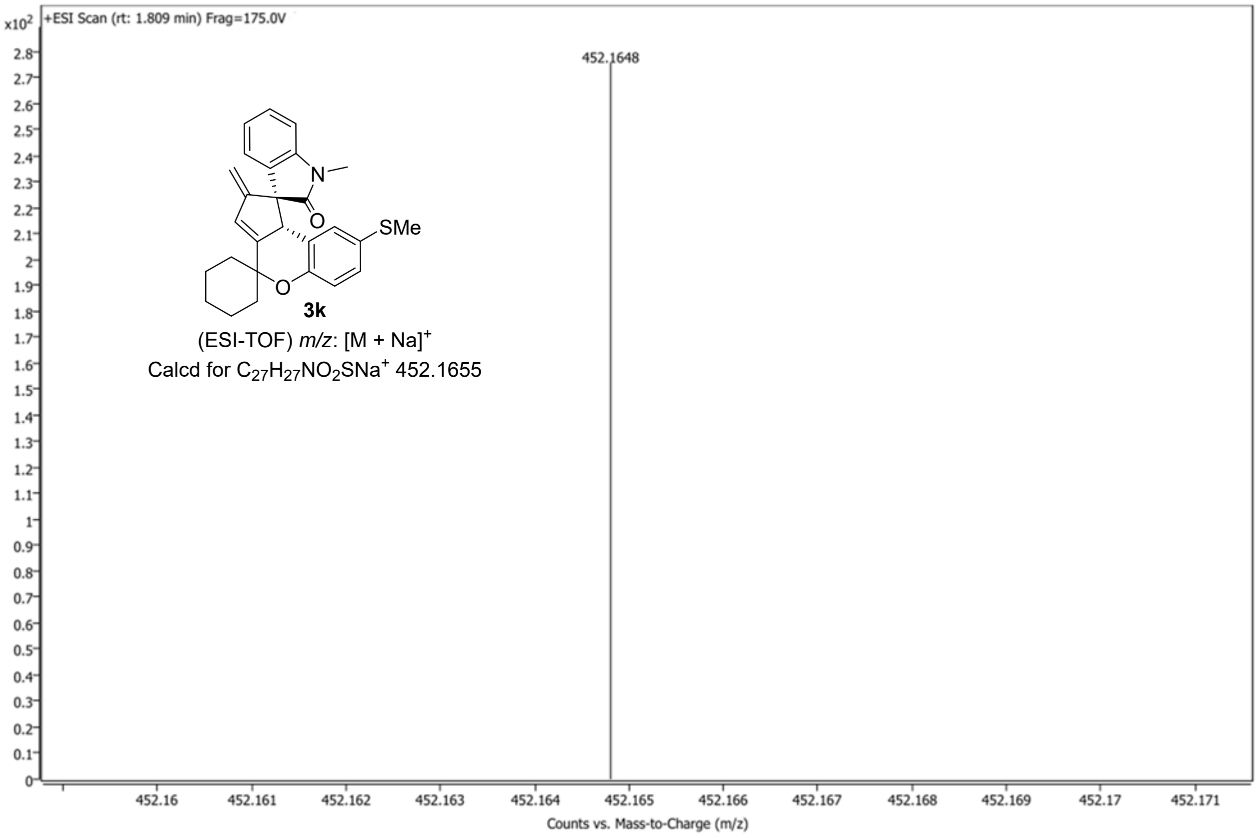
+ESI Scan (rt: 1.809 min) Frag=175.0V
x10^2
452.1648
SMe
N
O
O
3k
(ESI-TOF) m/z: [M + Na]+
Calcd for C27H27NO2SNa+ 452.1655
Counts vs. Mass-to-Charge (m/z)

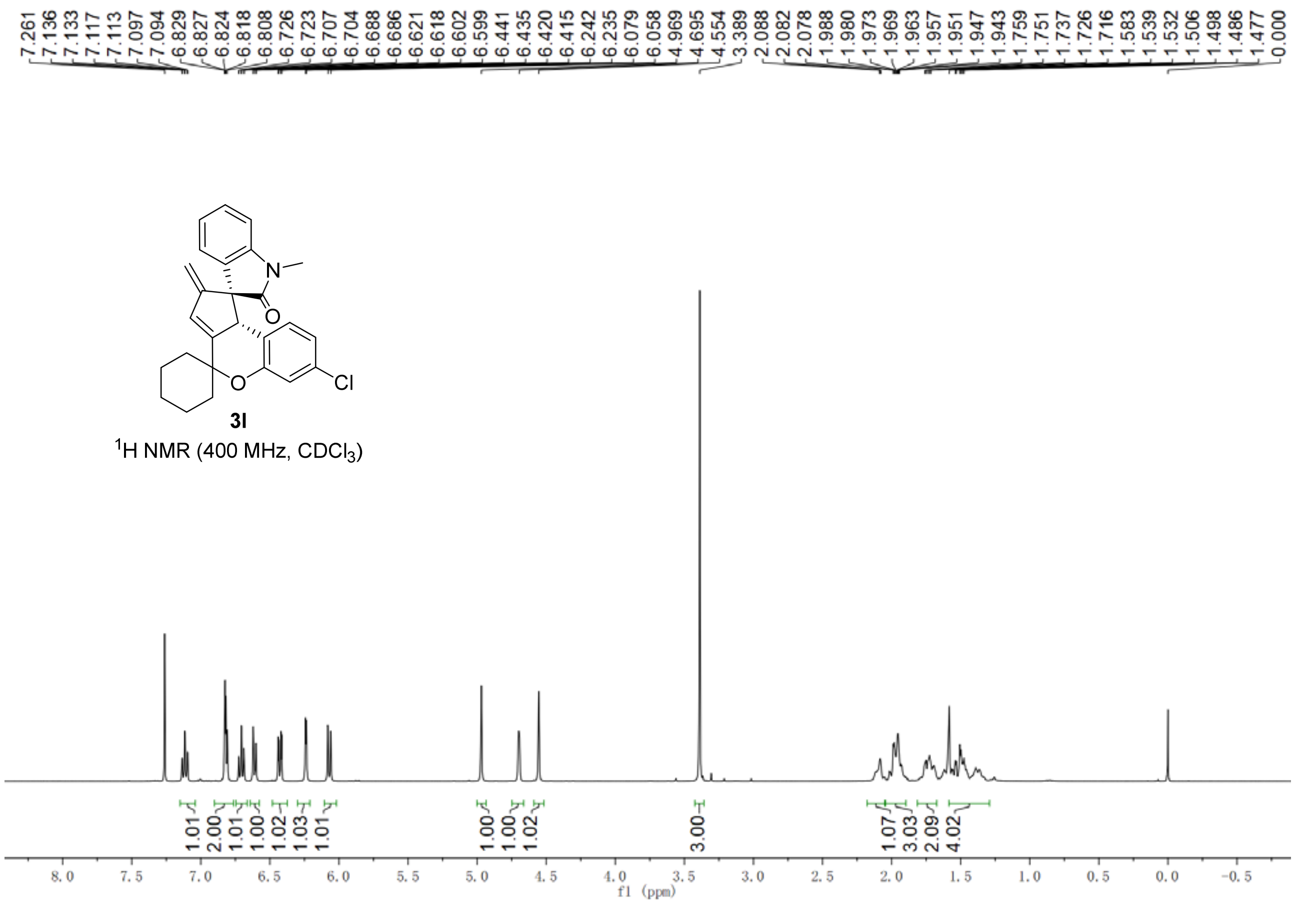

3l

^{1}H NMR (400 MHz, $CDCl_3$)

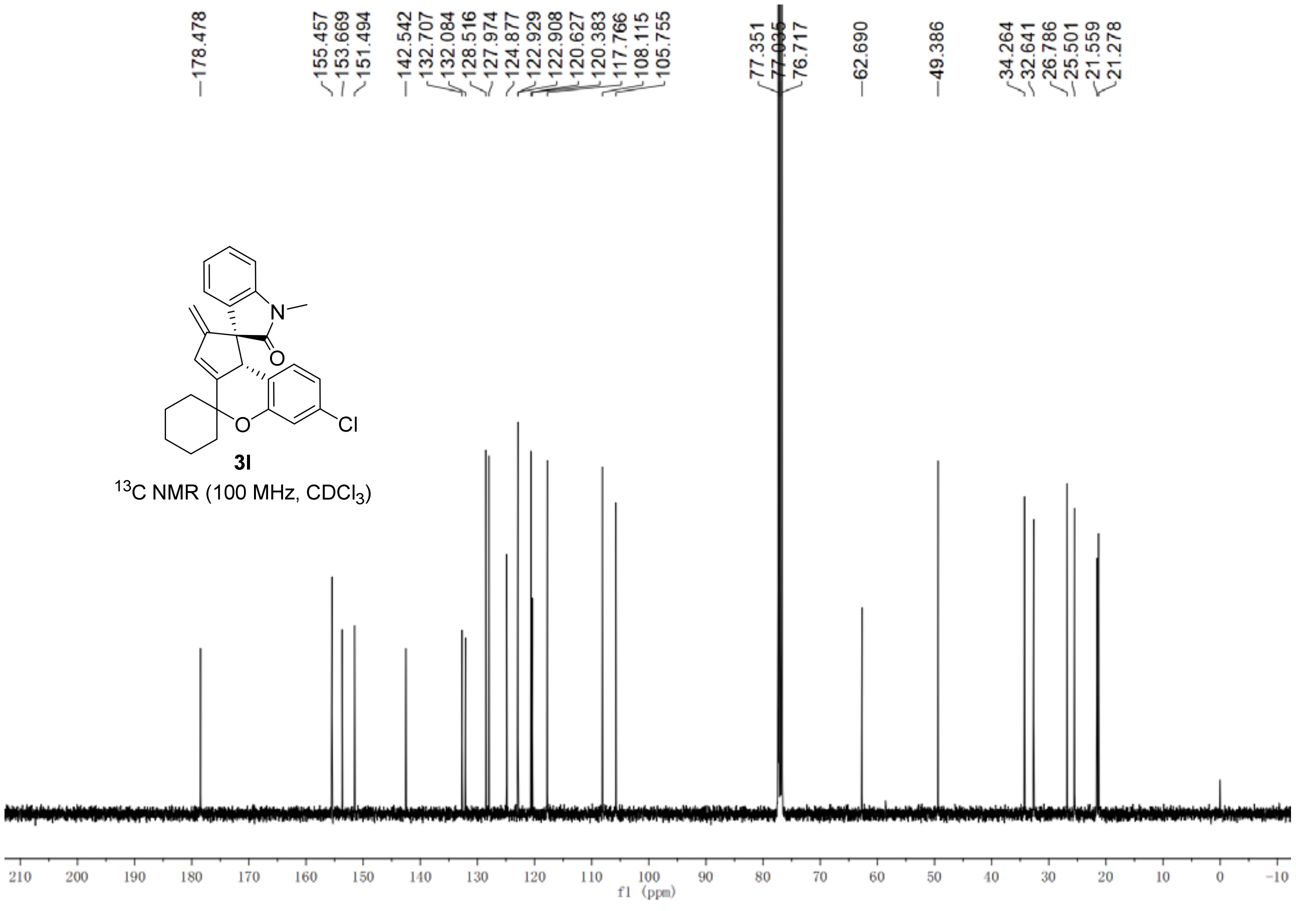

3l

^{13}C NMR (100 MHz, $CDCl_3$)

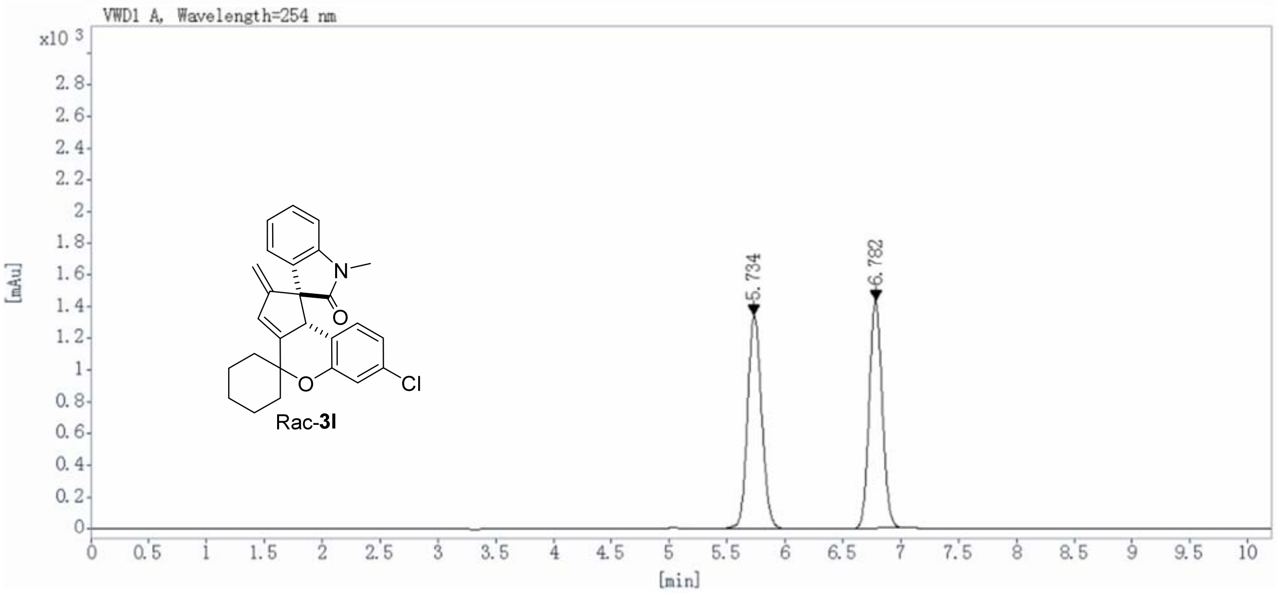

Ret Time [min]	Peak Type	Width [min]	Height [mAU]	Area [mAU*s]	Area [%]
5.734	BBA	0.13	1342.9154	11408.9932	50.4141
6.782	BB	0.12	1436.9625	11221.5811	49.5859
			Totals:	22630.5742	100.0000

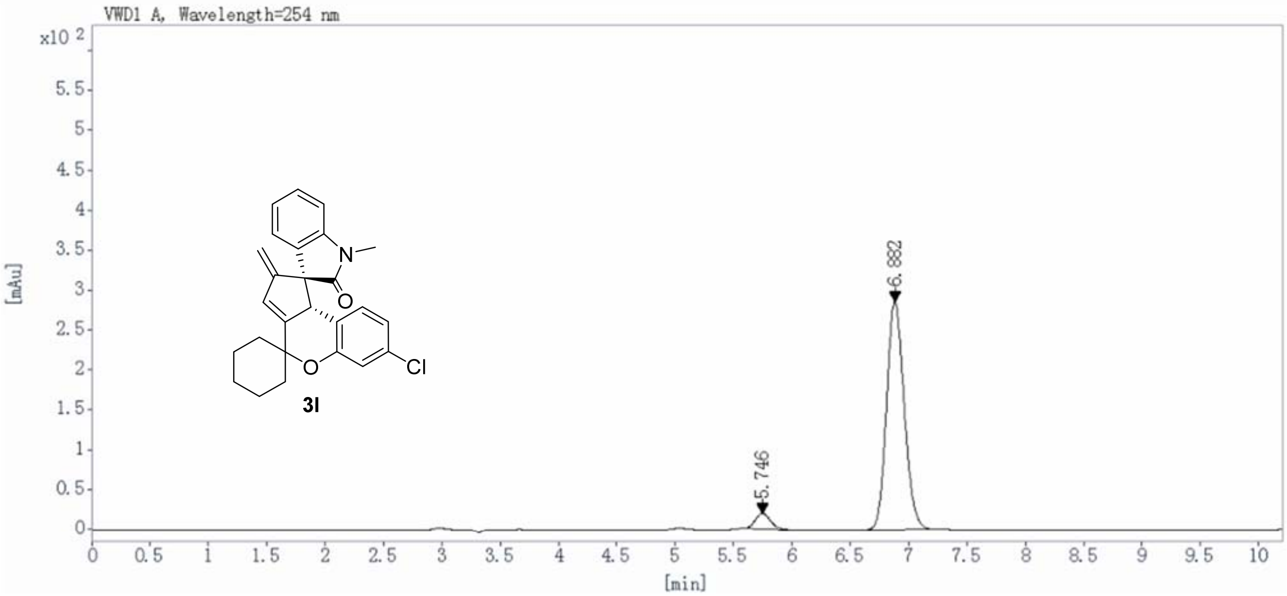

Ret Time [min]	Peak Type	Width [min]	Height [mAU]	Area [mAU*s]	Area [%]
5.746	BBA	0.14	19.8359	184.5714	5.7821
6.882	BBA	0.16	285.8464	3007.5542	94.2179
			Totals:	3192.1256	100.0000

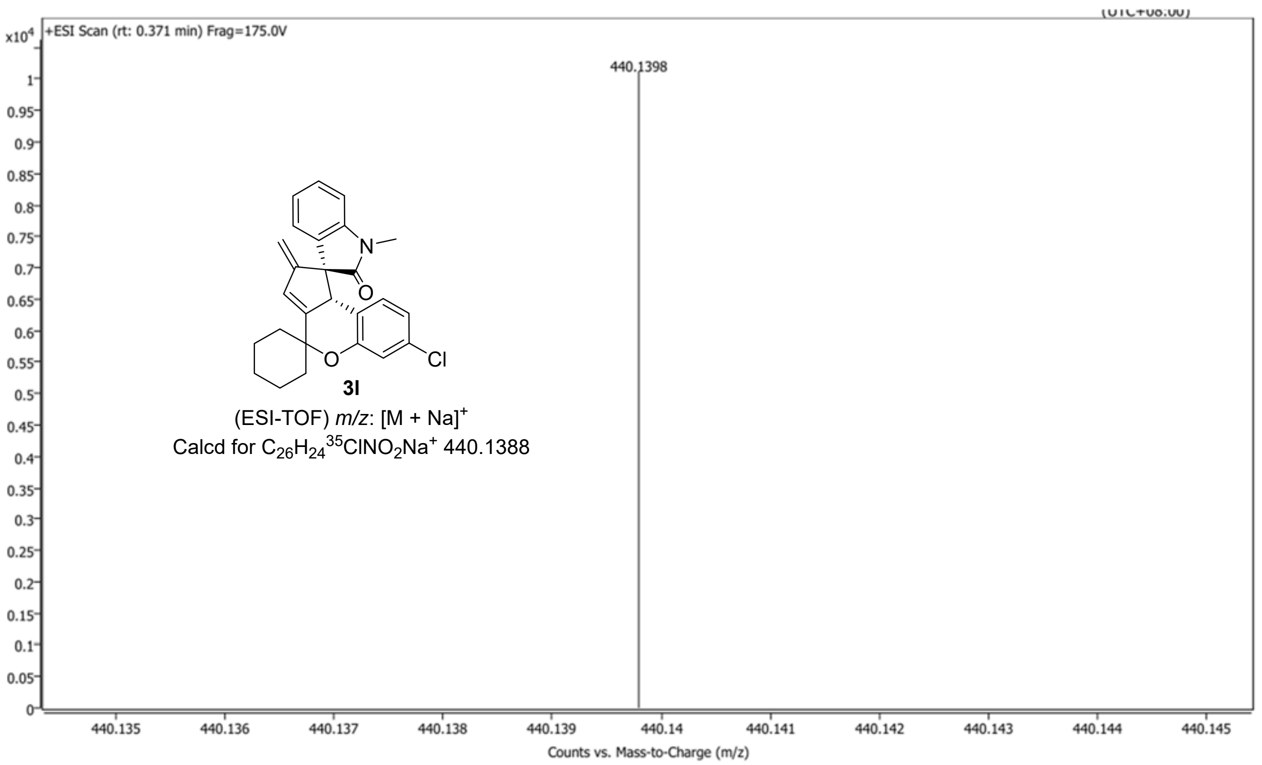
(UTC+08:00)
x10^4 +ESI Scan (rt: 0.371 min) Frag=175.0V
440.1398
N
O
O
Cl
3l
(ESI-TOF) m/z: [M + Na]+
Calcd for $C_{26}H_{24}{}^{35}ClNO_2Na^+$ 440.1388
Counts vs. Mass-to-Charge (m/z)

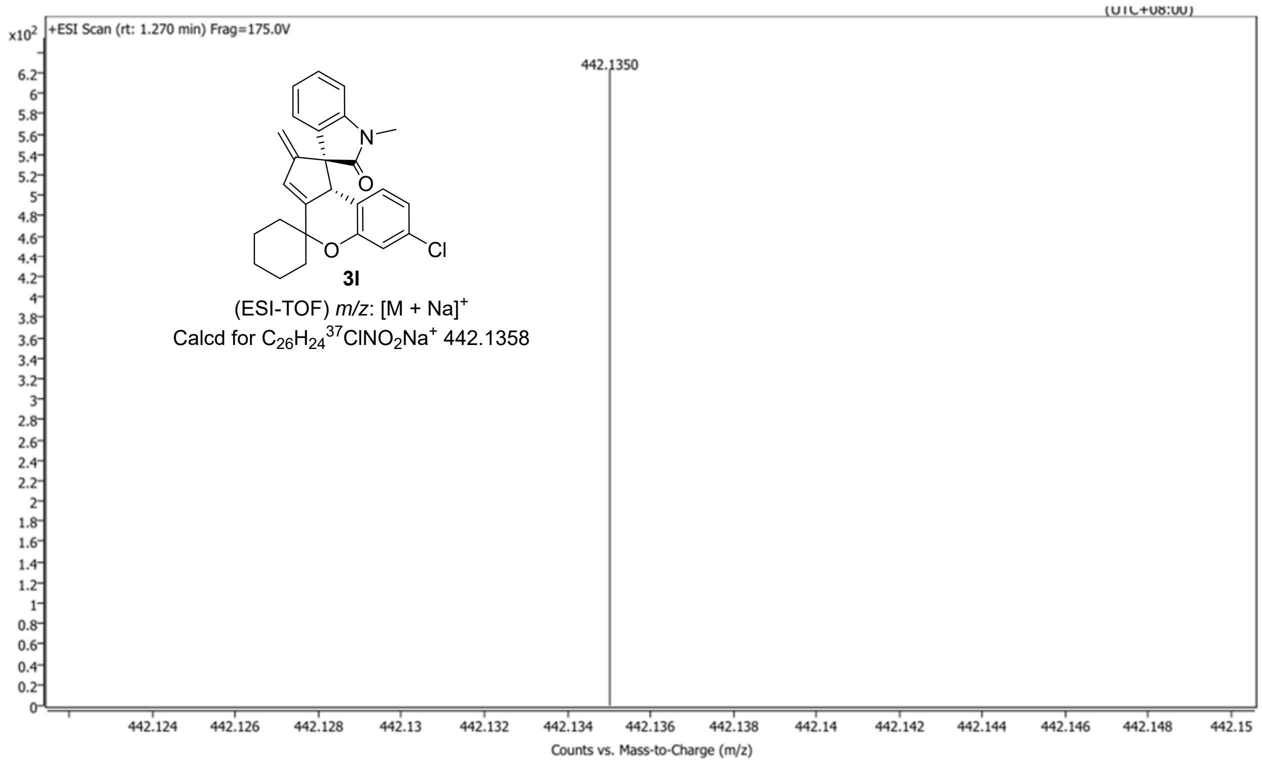
(UTC+08:00)
x10^2 +ESI Scan (rt: 1.270 min) Frag=175.0V
442.1350
N
O
O
Cl
3l
(ESI-TOF) m/z: [M + Na]+
Calcd for $C_{26}H_{24}{}^{37}ClNO_2Na^+$ 442.1358
Counts vs. Mass-to-Charge (m/z)

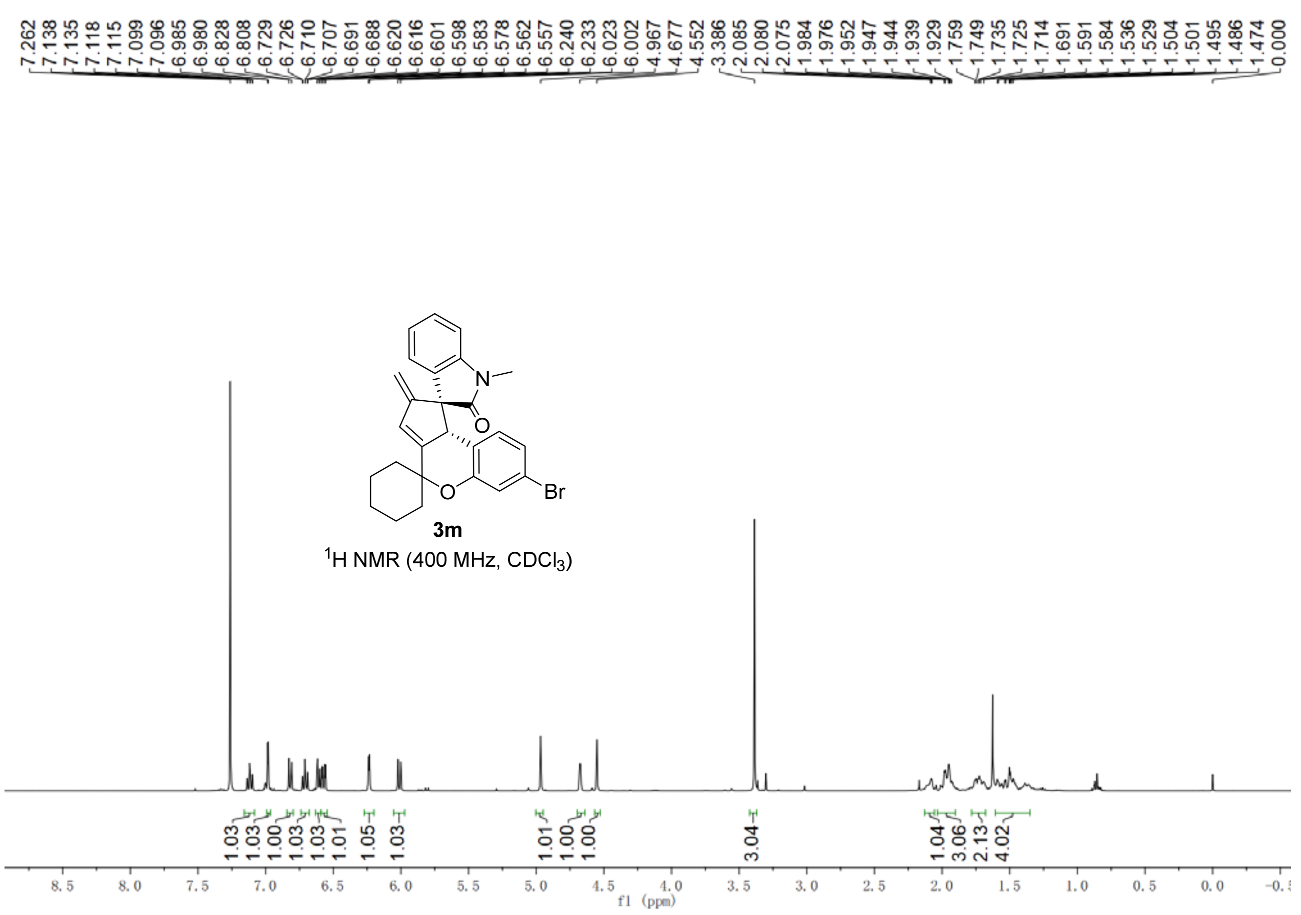
7.262
7.138
7.135
7.118
7.115
7.099
7.096
6.985
6.980
6.828
6.808
6.729
6.726
6.710
6.707
6.691
6.688
6.620
6.616
6.601
6.598
6.583
6.578
6.562
6.557
6.240
6.233
6.023
6.002
4.967
4.677
4.552
3.386
2.085
2.080
2.075
1.984
1.976
1.952
1.947
1.944
1.939
1.929
1.759
1.749
1.735
1.725
1.714
1.691
1.591
1.584
1.536
1.529
1.504
1.501
1.495
1.486
1.474
0.000
N
O
O
Br
3m
1H NMR (400 MHz, CDCl3)
1.03
1.03
1.00
1.03
1.03
1.01
1.05
1.03
1.01
1.00
1.00
3.04
1.04
3.06
2.13
4.02
8.5 8.0 7.5 7.0 6.5 6.0 5.5 5.0 4.5 4.0 3.5 3.0 2.5 2.0 1.5 1.0 0.5 0.0 -0.5
f1 (ppm)

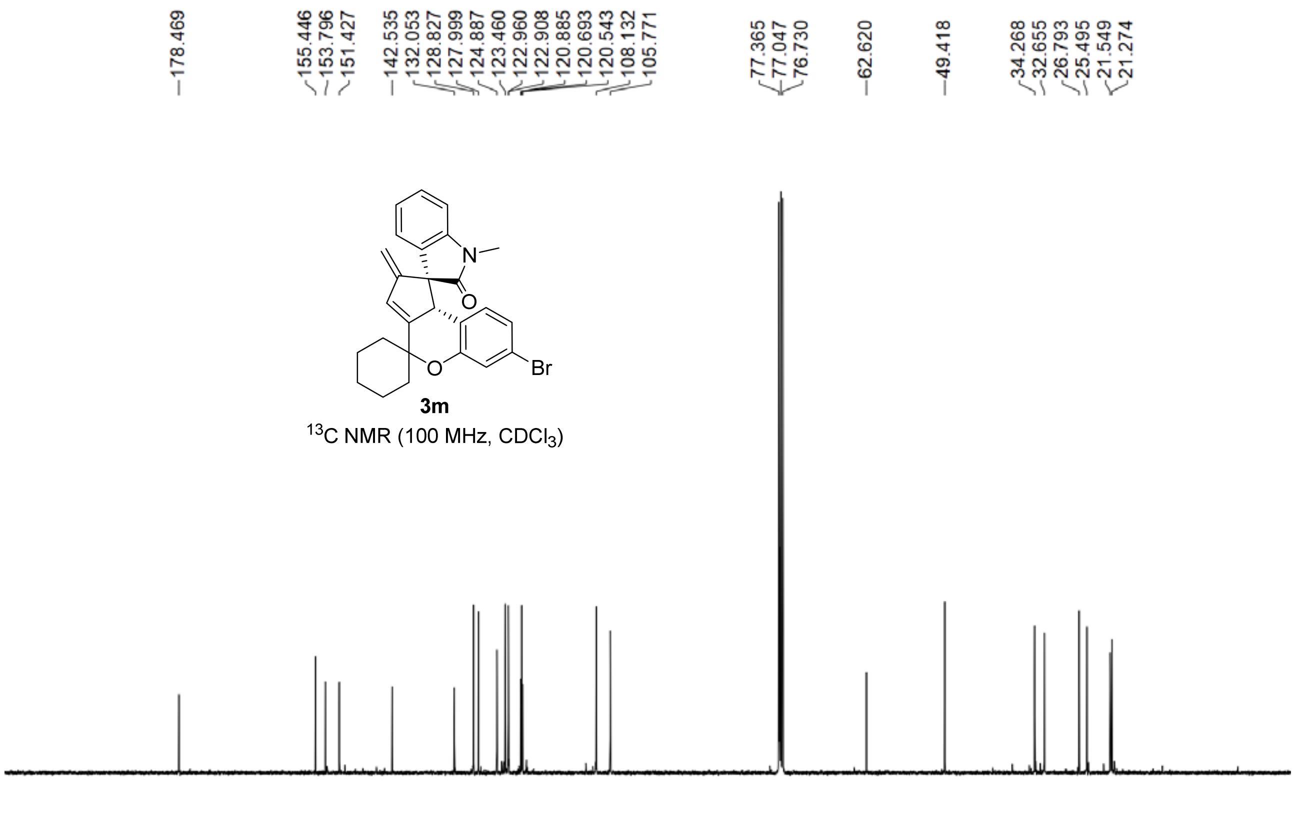
178.469
155.446
153.796
151.427
142.535
132.053
128.827
127.999
124.887
123.460
122.960
122.908
120.885
120.693
120.543
108.132
105.771
77.365
77.047
76.730
62.620
49.418
34.268
32.655
26.793
25.495
21.549
21.274
N
O
O
Br
3m
13C NMR (100 MHz, CDCl3)
200 190 180 170 160 150 140 130 120 110 100 90 80 70 60 50 40 30 20 10 0
f1 (ppm)

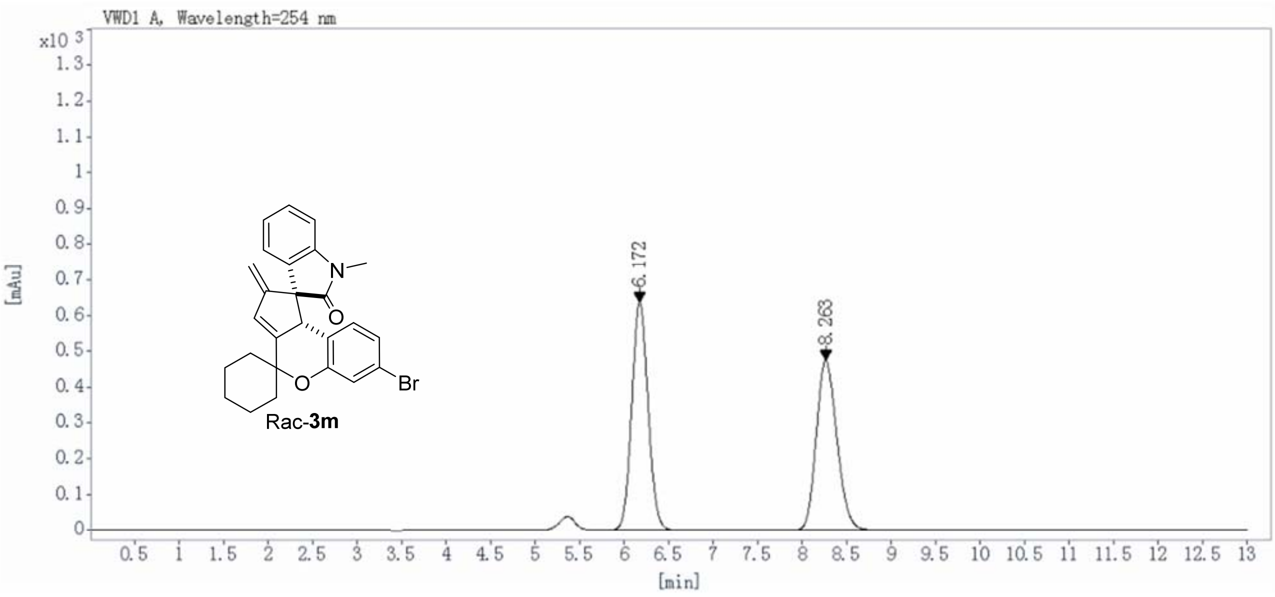

Ret Time [min]	Peak Type	Width [min]	Height [mAU]	Area [mAU*s]	Area [%]
6.172	BBA	0.20	636.8077	8103.1338	51.3770
8.263	BB	0.25	474.3646	7668.7861	48.6230
			Totals:	15771.9199	100.0000

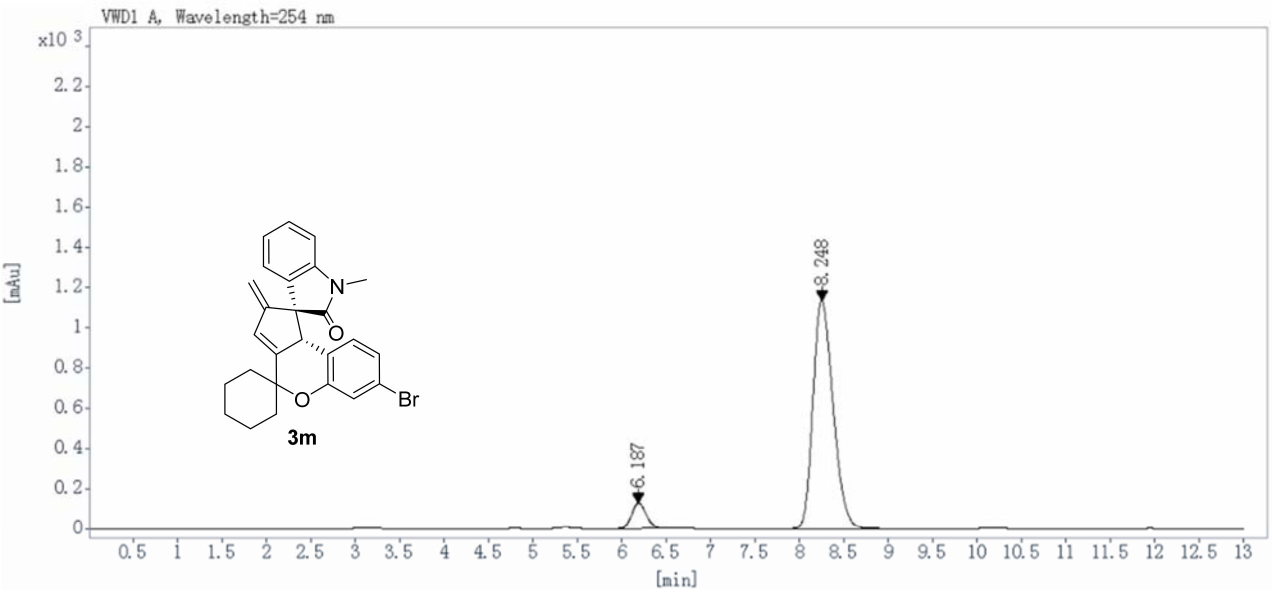

Ret Time [min]	Peak Type	Width [min]	Height [mAU]	Area [mAU*s]	Area [%]
6.187	BB	0.17	126.9802	1423.3439	7.4052
8.248	BBA	0.24	1134.6816	17797.6406	92.5948
			Totals:	19220.9845	100.0000

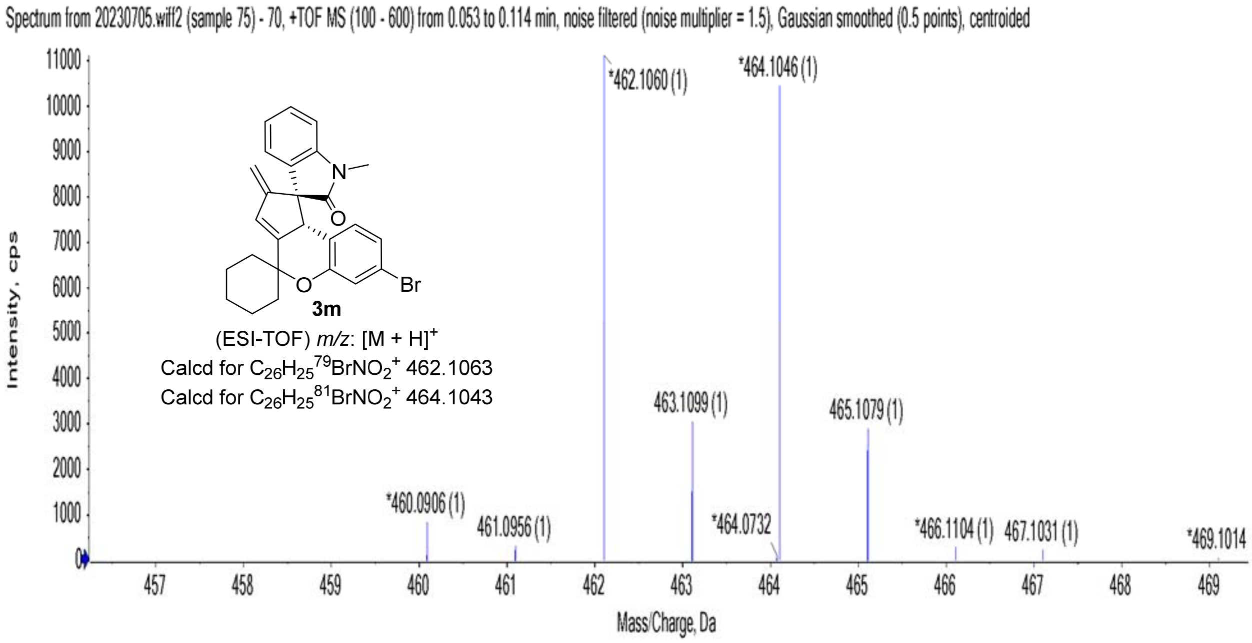

Spectrum from 20230705.wiff2 (sample 75) - 70, +TOF MS (100 - 600) from 0.053 to 0.114 min, noise filtered (noise multiplier = 1.5), Gaussian smoothed (0.5 points), centroided
Intensity, cps
Mass/Charge, Da
N
O
O
Br
3m
(ESI-TOF) m/z: [M + H]+
Calcd for C26H25 79BrNO2+ 462.1063
Calcd for C26H25 81BrNO2+ 464.1043
*462.1060 (1)
*464.1046 (1)
463.1099 (1)
465.1079 (1)
*460.0906 (1)
461.0956 (1)
*464.0732
*466.1104 (1)
467.1031 (1)
*469.1014

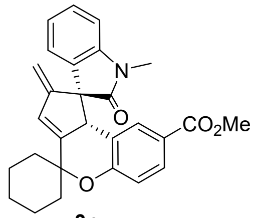

3n

^{1}H NMR (400 MHz, $CDCl_3$)

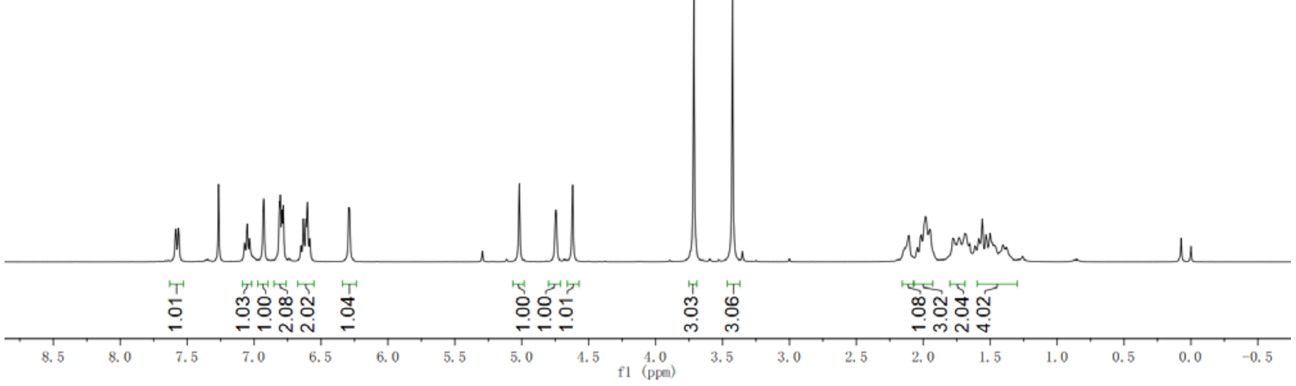

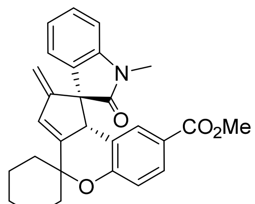

3n

^{13}C NMR (100 MHz, $CDCl_3$)

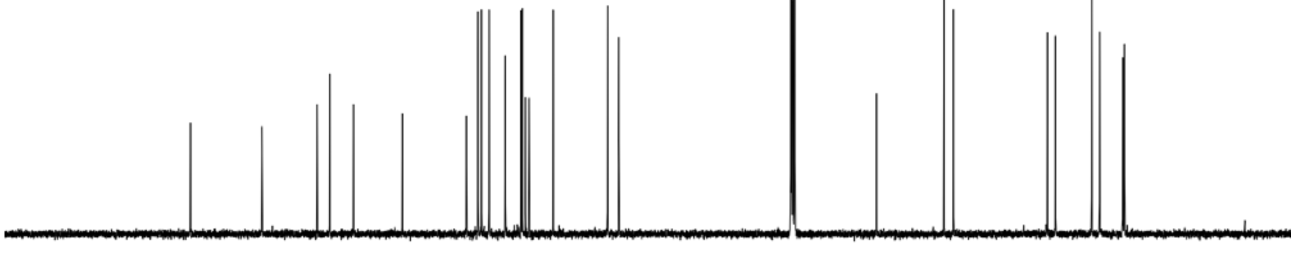

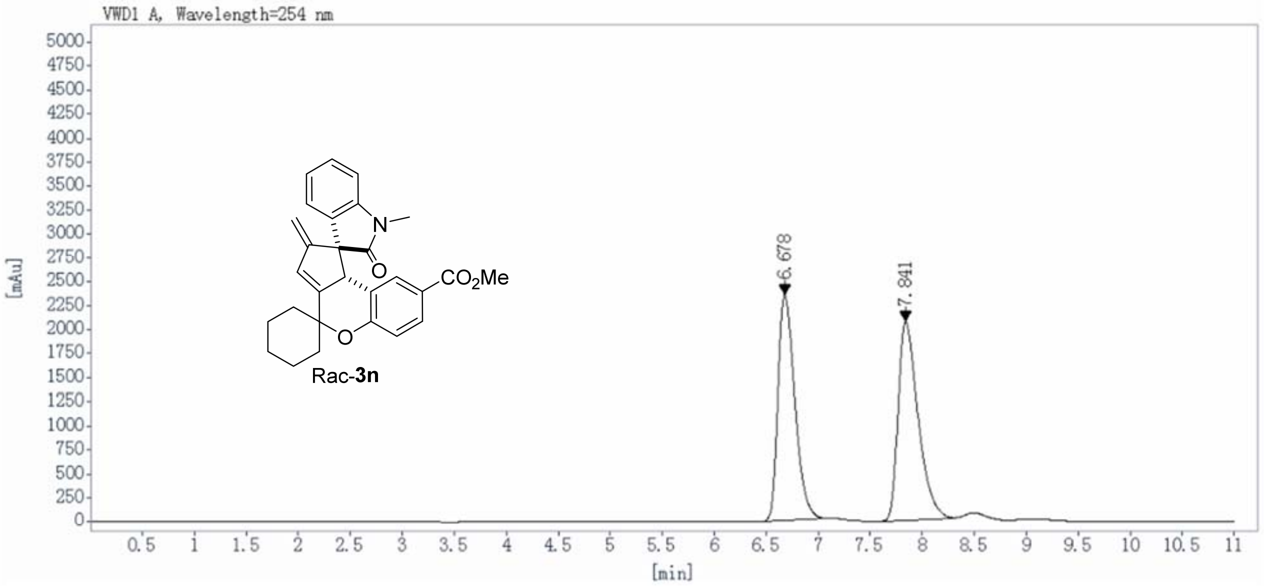

Ret Time [min]	Peak Type	Width [min]	Height [mAU]	Area [mAU*s]	Area [%]
6.678	BB	0.17	2350.3589	26511.5176	49.0665
7.841	BB	0.20	2061.8716	27520.2773	50.9335
			Totals:	54031.7949	100.0000

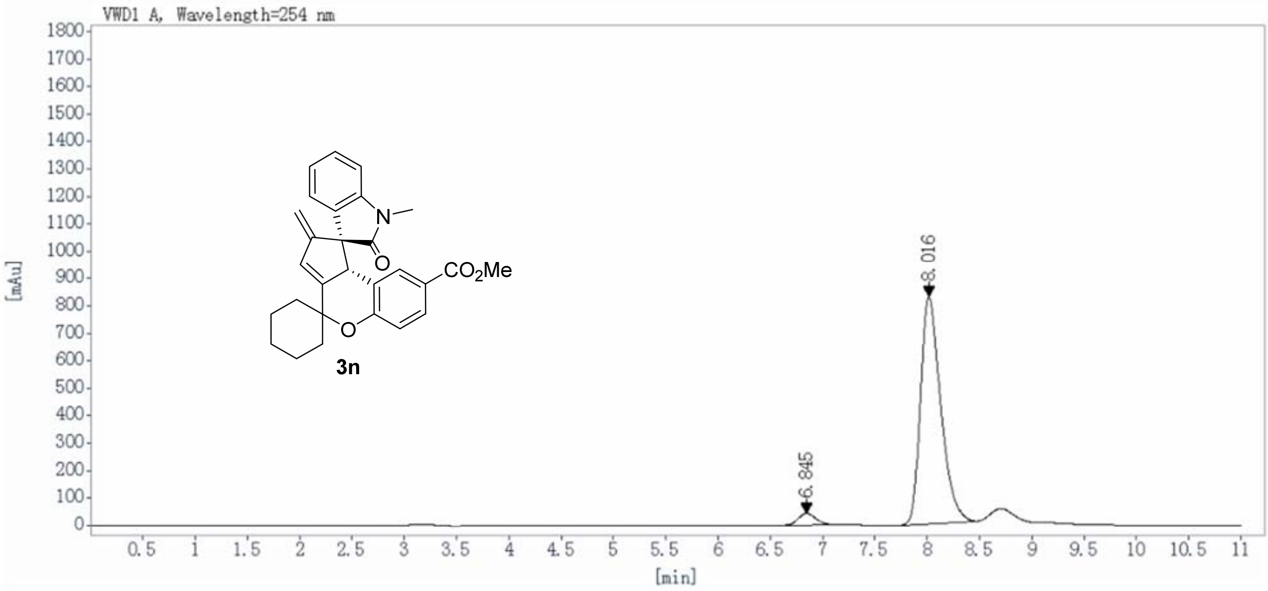

Ret Time [min]	Peak Type	Width [min]	Height [mAU]	Area [mAU*s]	Area [%]
6.845	BBA	0.18	42.4669	508.9646	4.3805
8.016	BB	0.21	825.9418	11109.8457	95.6195
			Totals:	11618.8103	100.0000

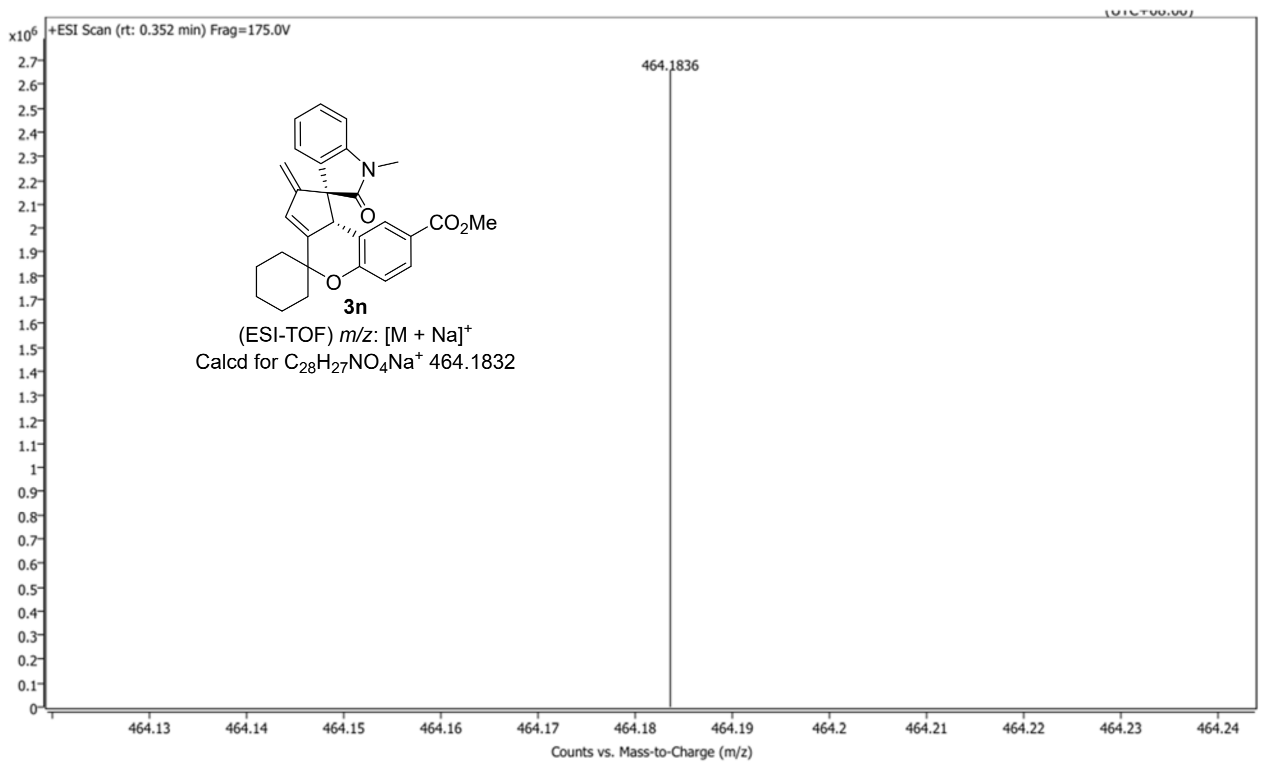

+ESI Scan (rt: 0.352 min) Frag=175.0V
x10^6
464.1836
CO2Me
N
O
O
3n
(ESI-TOF) m/z: [M + Na]+
Calcd for C28H27NO4Na+ 464.1832
Counts vs. Mass-to-Charge (m/z)

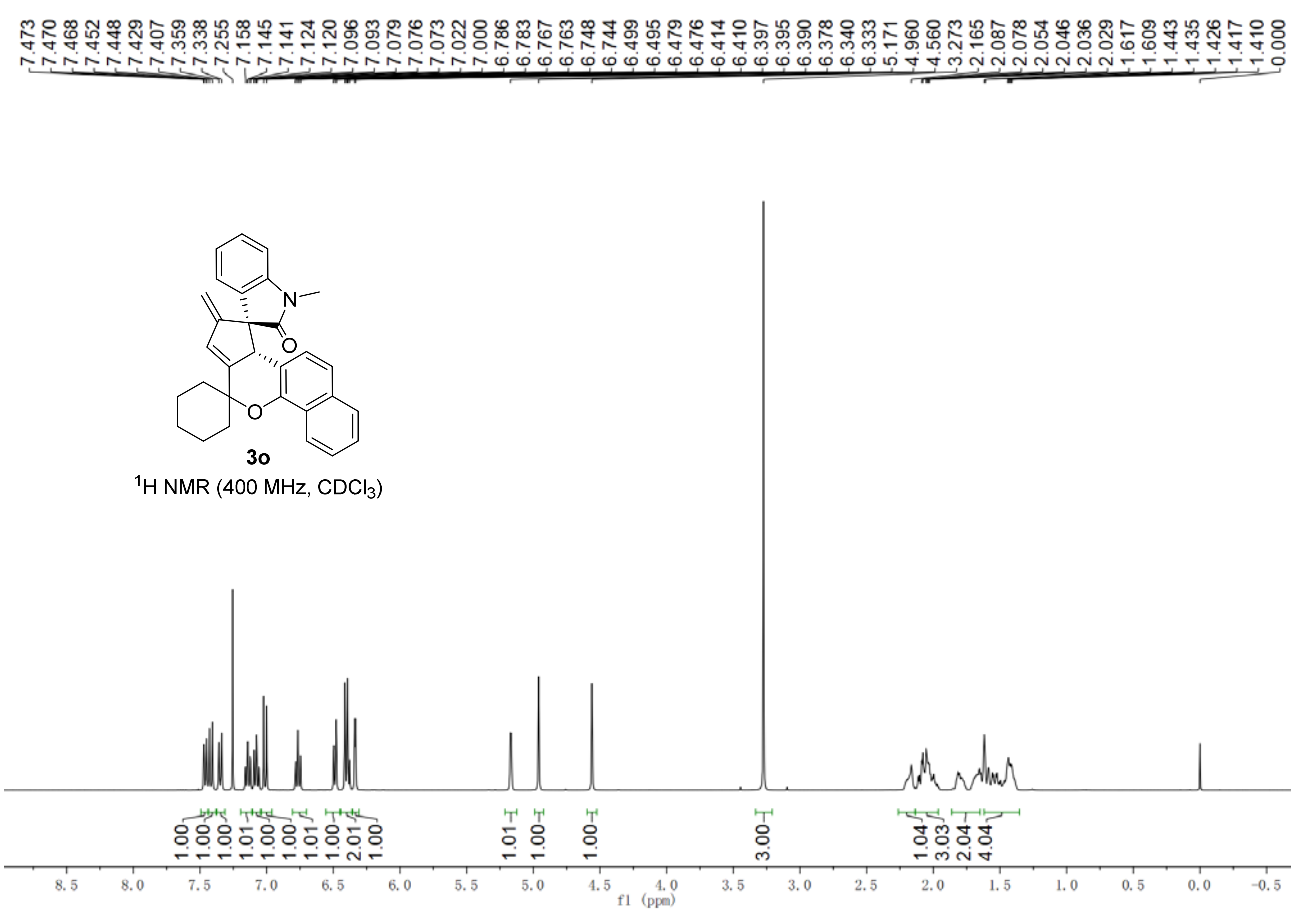

3o

^{1}H NMR (400 MHz, $CDCl_3$)

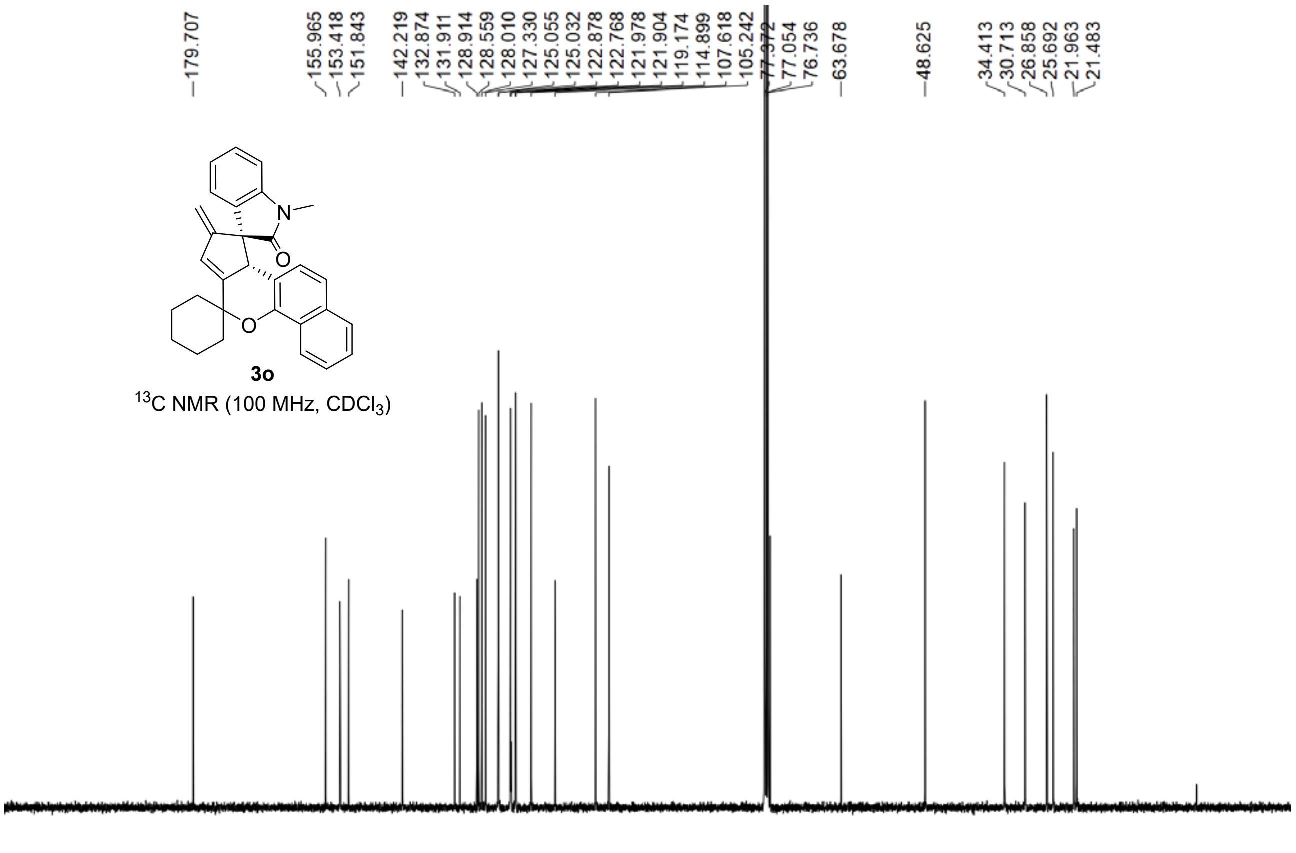

3o

^{13}C NMR (100 MHz, $CDCl_3$)

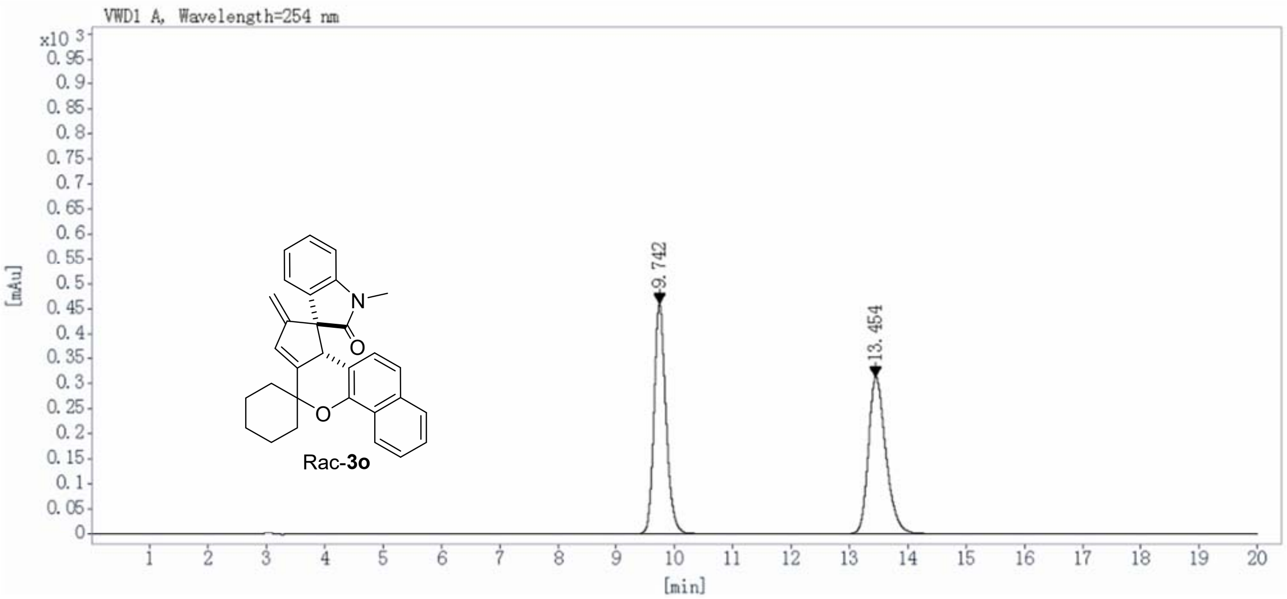

Ret Time [min]	Peak Type	Width [min]	Height [mAU]	Area [mAU*s]	Area [%]
9.742	BBA	0.22	460.1235	6490.4111	49.9937
13.454	BBA	0.32	313.6683	6492.0356	50.0063
			Totals:	12982.4468	100.0000

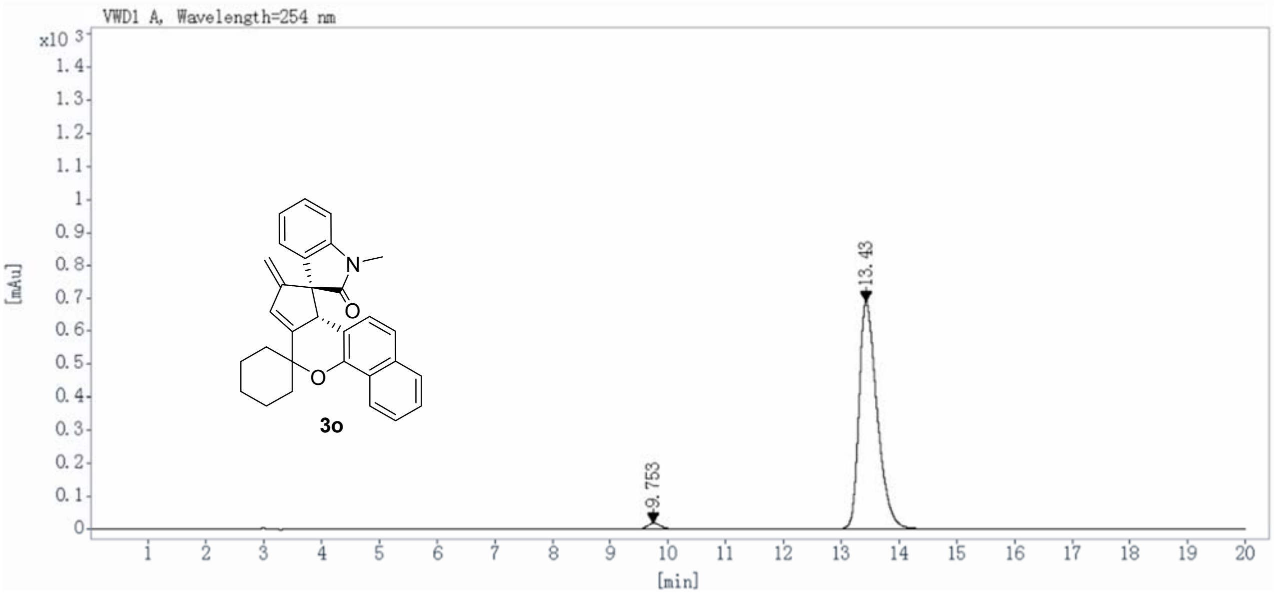

Ret Time [min]	Peak Type	Width [min]	Height [mAU]	Area [mAU*s]	Area [%]
9.753	BBA	0.21	19.0634	265.1130	1.7354
13.430	BBA	0.33	690.0911	15012.0088	98.2646
			Totals:	15277.1218	100.0000

Spectrum from 20230705.wiff2 (sample 79) - 74, +TOF MS (100 - 600) from 0.059 to 0.120 min, noise filtered (noise multiplier = 1.5), Gaussian smoothed (0.5 points), centroided

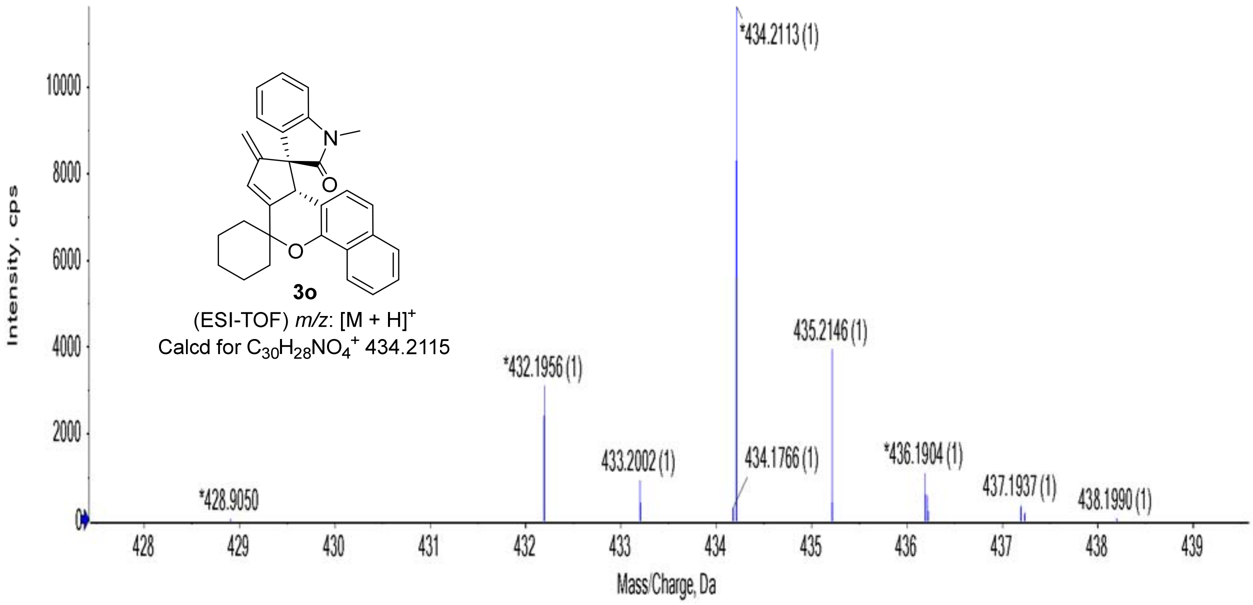

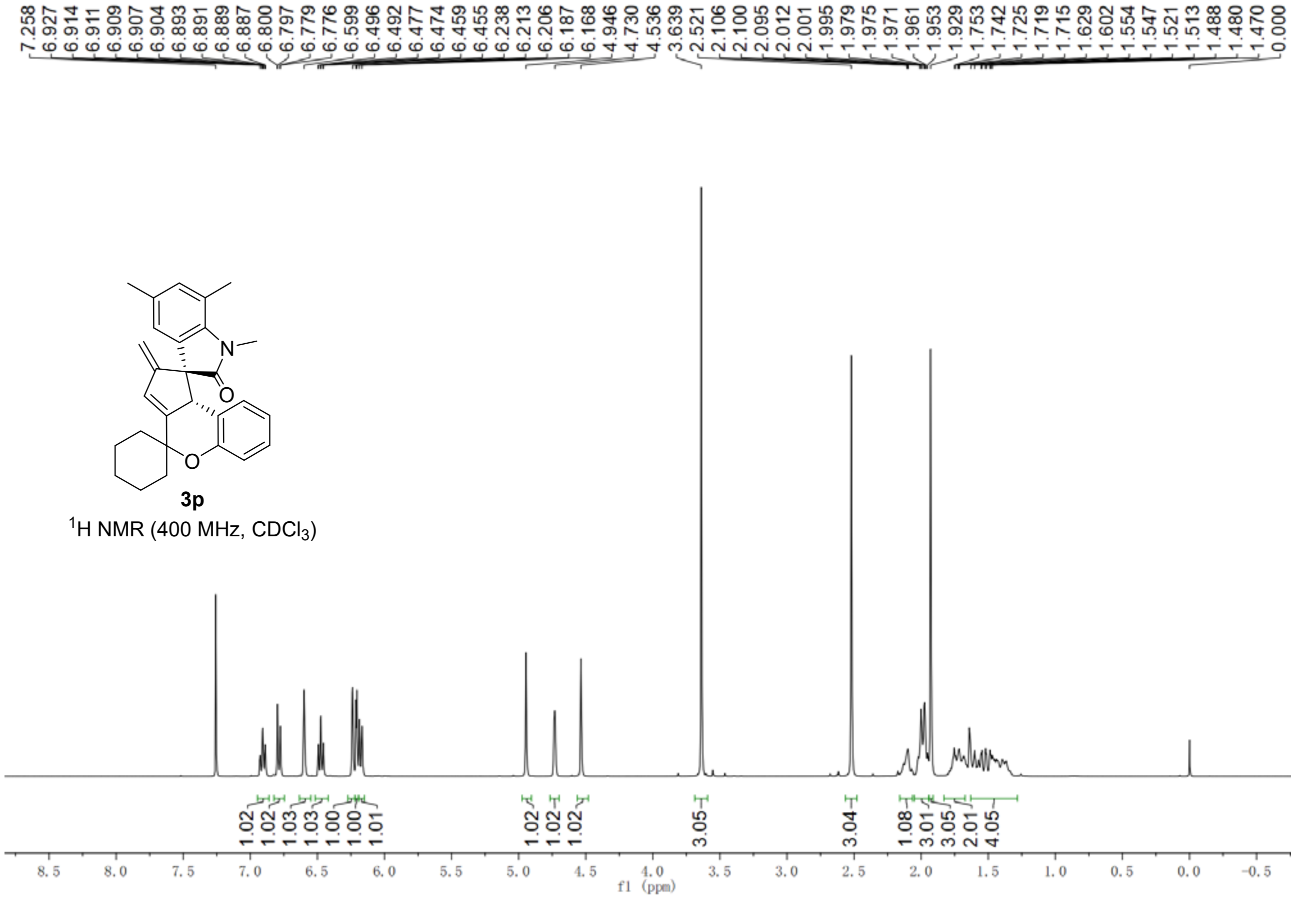

3p

^{1}H NMR (400 MHz, $CDCl_3$)

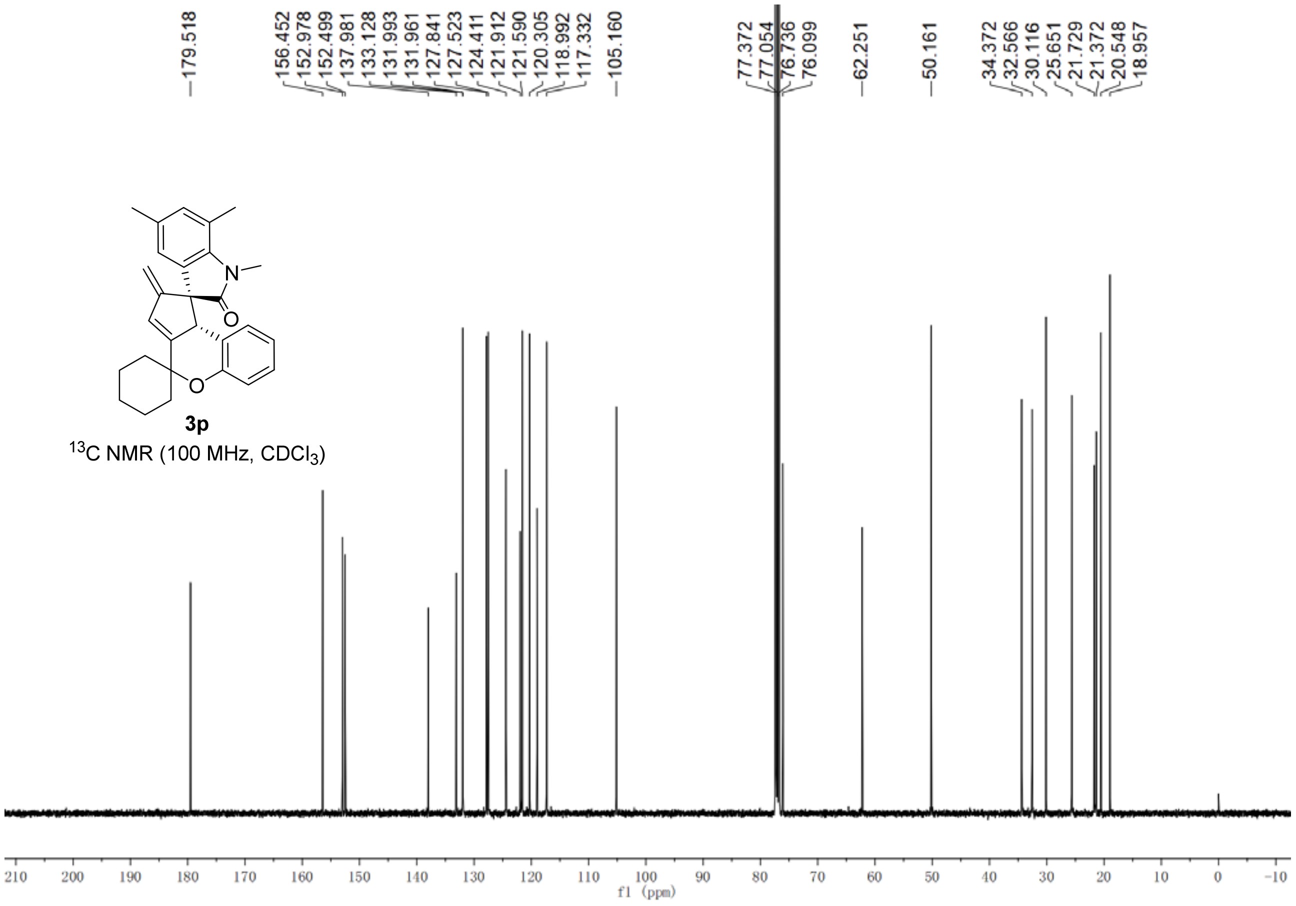

3p

^{13}C NMR (100 MHz, $CDCl_3$)

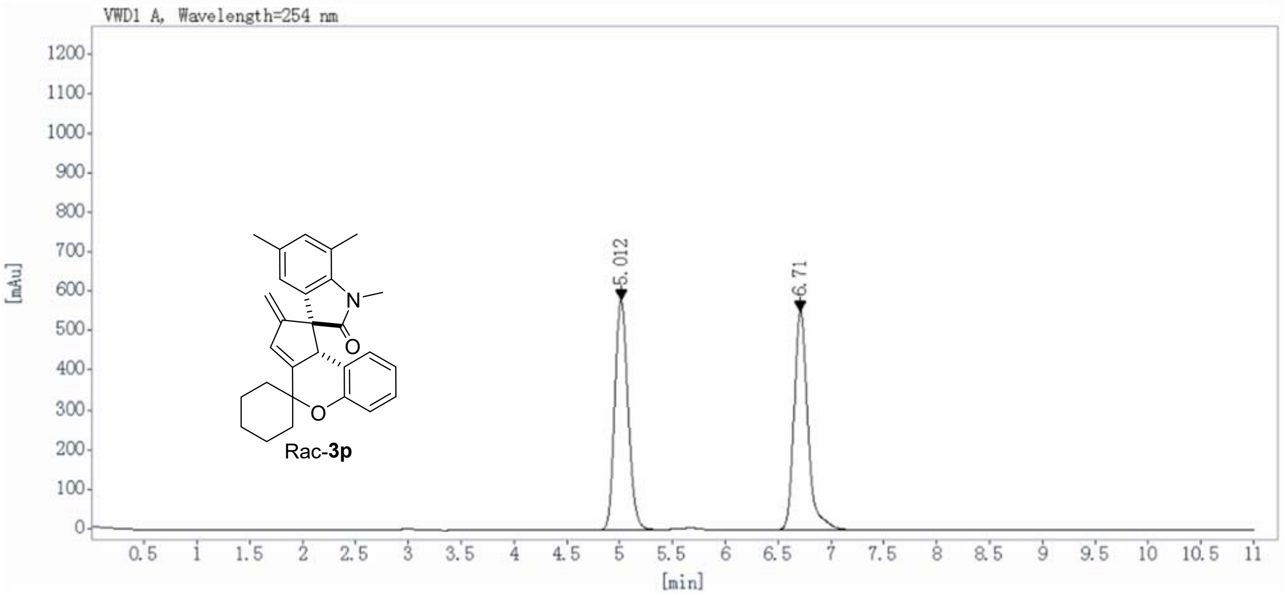

Ret Time [min]	Peak Type	Width [min]	Height [mAU]	Area [mAU*s]	Area [%]
5.012	BBA	0.13	578.7755	5017.1655	50.0194
6.710	BBA	0.14	550.9875	5013.2646	49.9806
			Totals:	10030.4302	100.0000

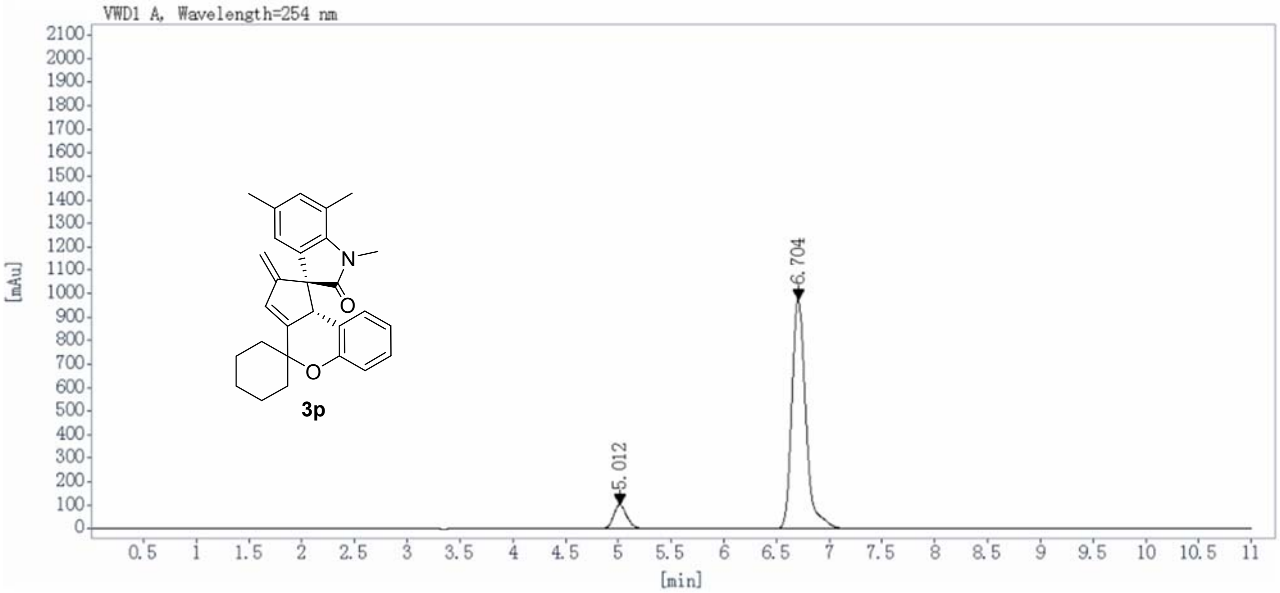

Ret Time [min]	Peak Type	Width [min]	Height [mAU]	Area [mAU*s]	Area [%]
5.012	BB	0.13	102.0049	887.6588	9.0235
6.704	BBA	0.14	976.2433	8949.5518	90.9765
			Totals:	9837.2106	100.0000

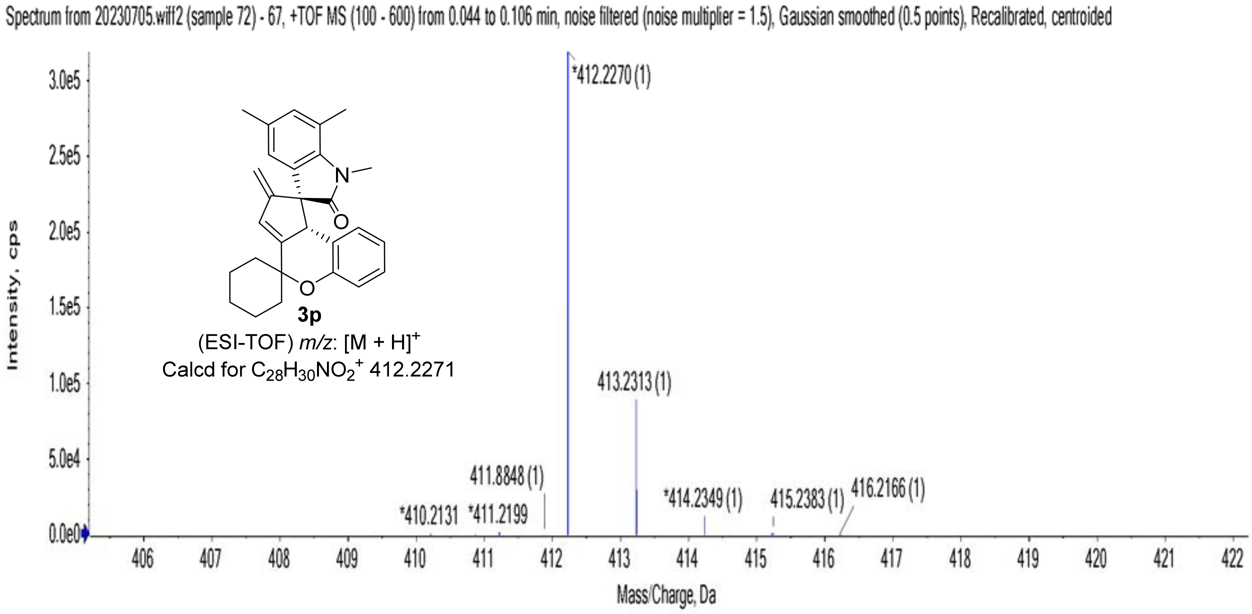
Spectrum from 20230705.wiff2 (sample 72) - 67, +TOF MS (100 - 600) from 0.044 to 0.106 min, noise filtered (noise multiplier = 1.5), Gaussian smoothed (0.5 points), Recalibrated, centroided
3p
(ESI-TOF) m/z: [M + H]+
Calcd for C28H30NO2+ 412.2271
*412.2270 (1)
413.2313 (1)
411.8848 (1)
*410.2131
*411.2199
*414.2349 (1)
415.2383 (1)
416.2166 (1)
Intensity, cps
Mass/Charge, Da

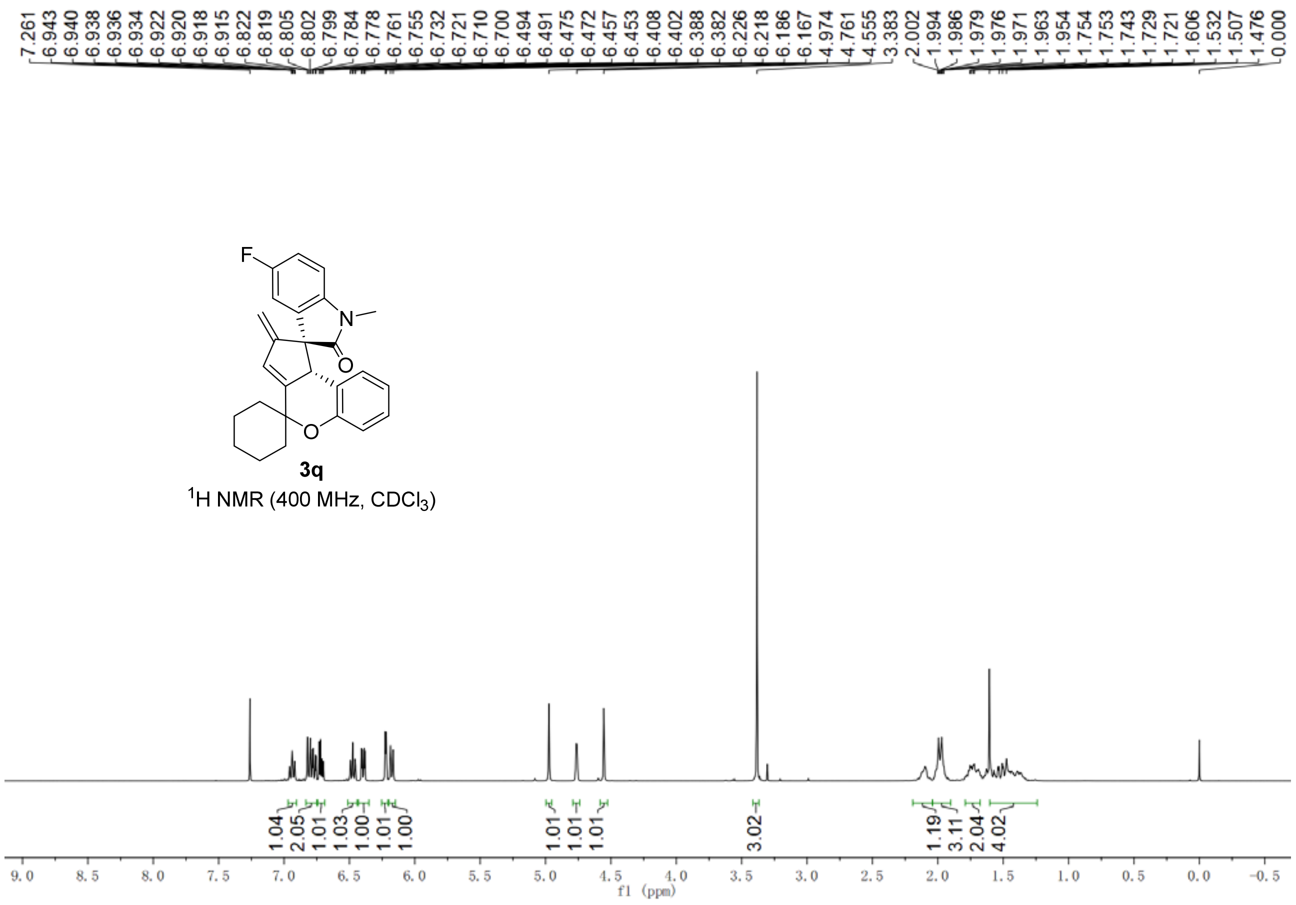
7.261
6.943
6.940
6.938
6.936
6.934
6.922
6.920
6.918
6.915
6.822
6.819
6.805
6.802
6.799
6.784
6.778
6.761
6.755
6.732
6.721
6.710
6.700
6.494
6.491
6.475
6.472
6.457
6.453
6.408
6.402
6.388
6.382
6.226
6.218
6.186
6.167
4.974
4.761
4.555
3.383
2.002
1.994
1.986
1.979
1.976
1.971
1.963
1.954
1.754
1.753
1.743
1.729
1.721
1.606
1.532
1.507
1.476
0.000
F
N
O
O
3q
1H NMR (400 MHz, CDCl3)
1.04
2.05
1.01
1.03
1.00
1.01
1.00
1.01
1.01
1.01
3.02
1.19
3.11
2.04
4.02
f1 (ppm)

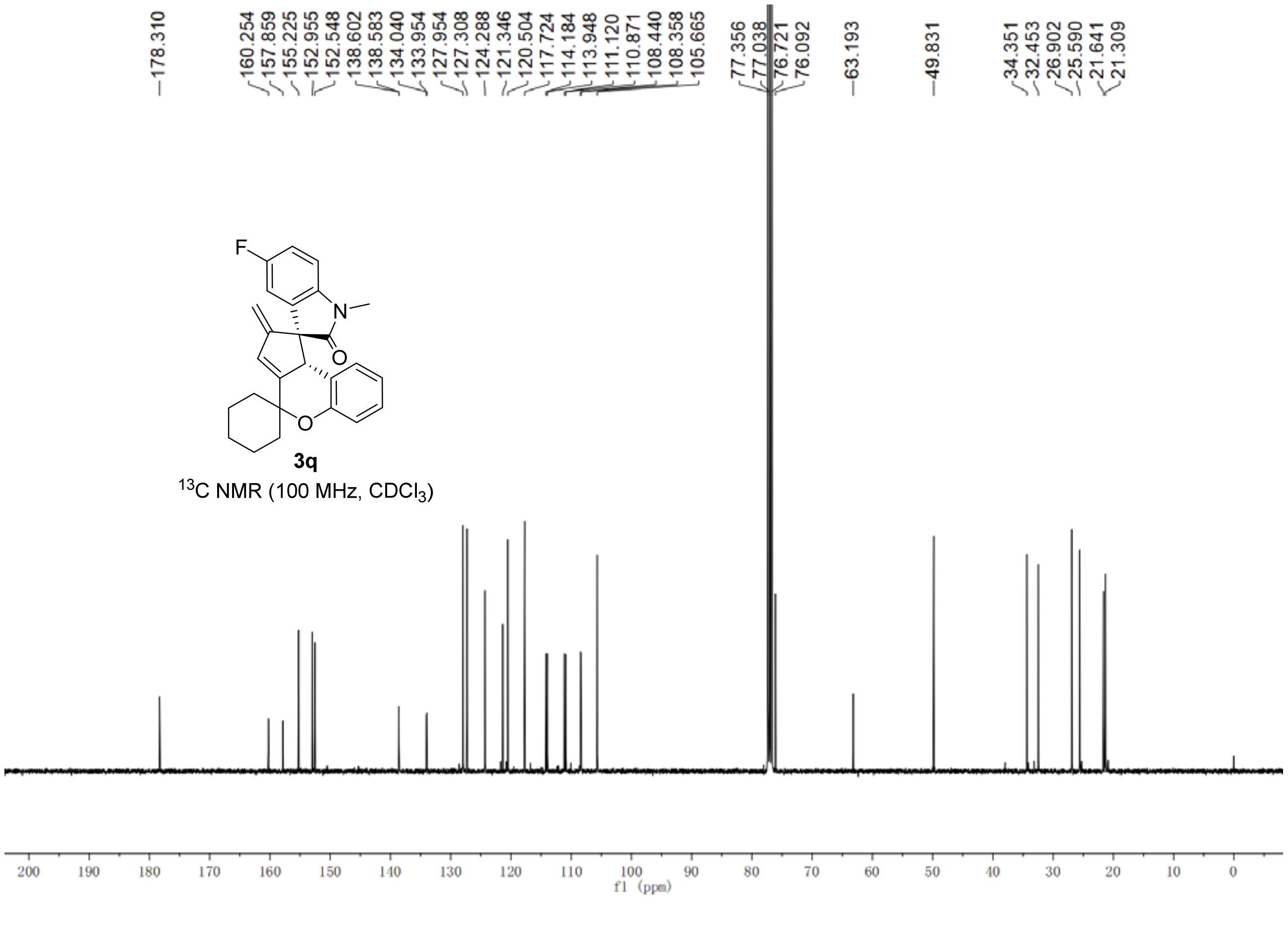
178.310
160.254
157.859
155.225
152.955
152.548
138.602
138.583
134.040
133.954
127.954
127.308
124.288
121.346
120.504
117.724
114.184
113.948
111.120
110.871
108.440
108.358
105.665
77.356
77.038
76.721
76.092
63.193
49.831
34.351
32.453
26.902
25.590
21.641
21.309
F
N
O
O
3q
13C NMR (100 MHz, CDCl3)
f1 (ppm)

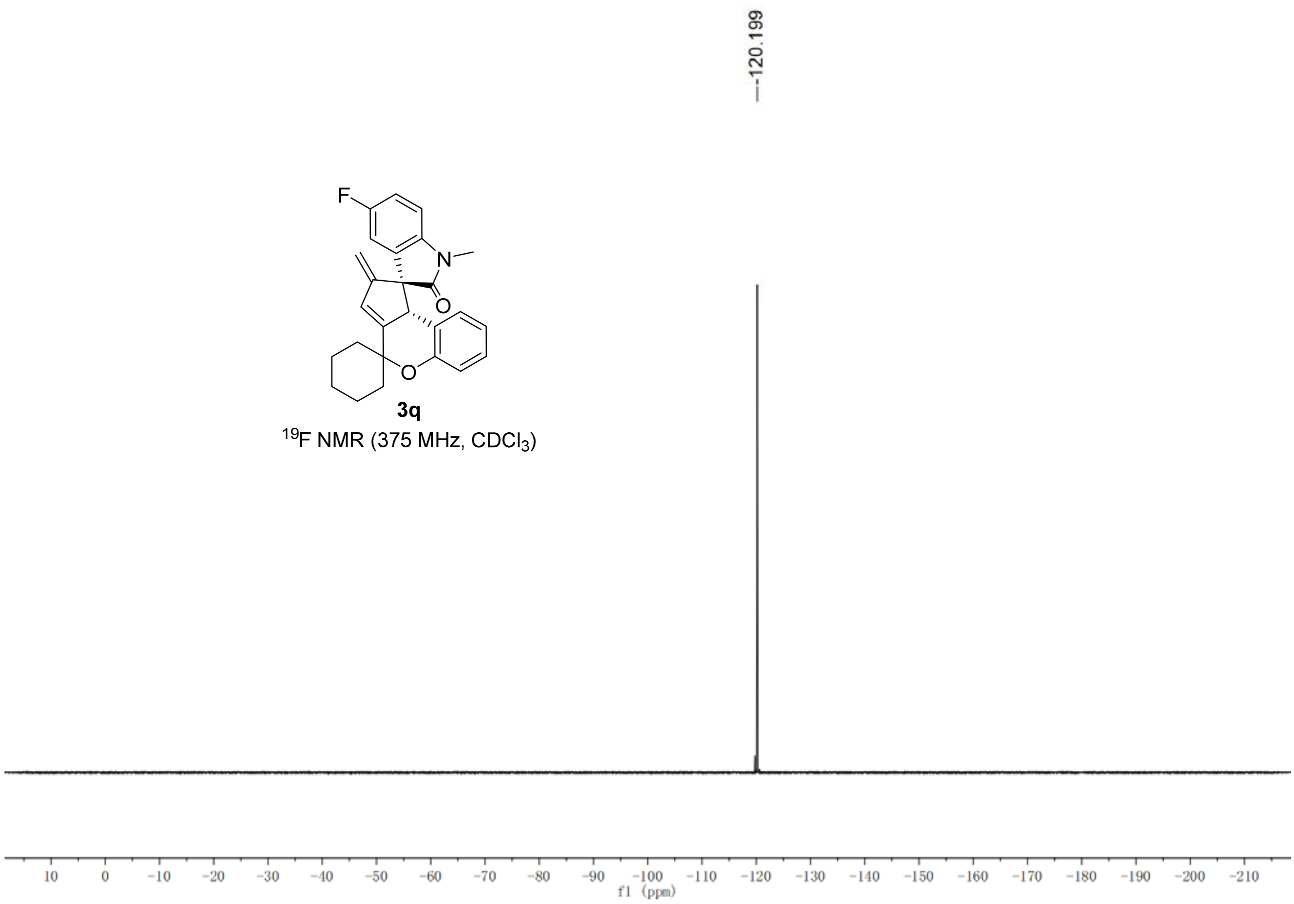

3q

^{19}F NMR (375 MHz, $CDCl_3$)

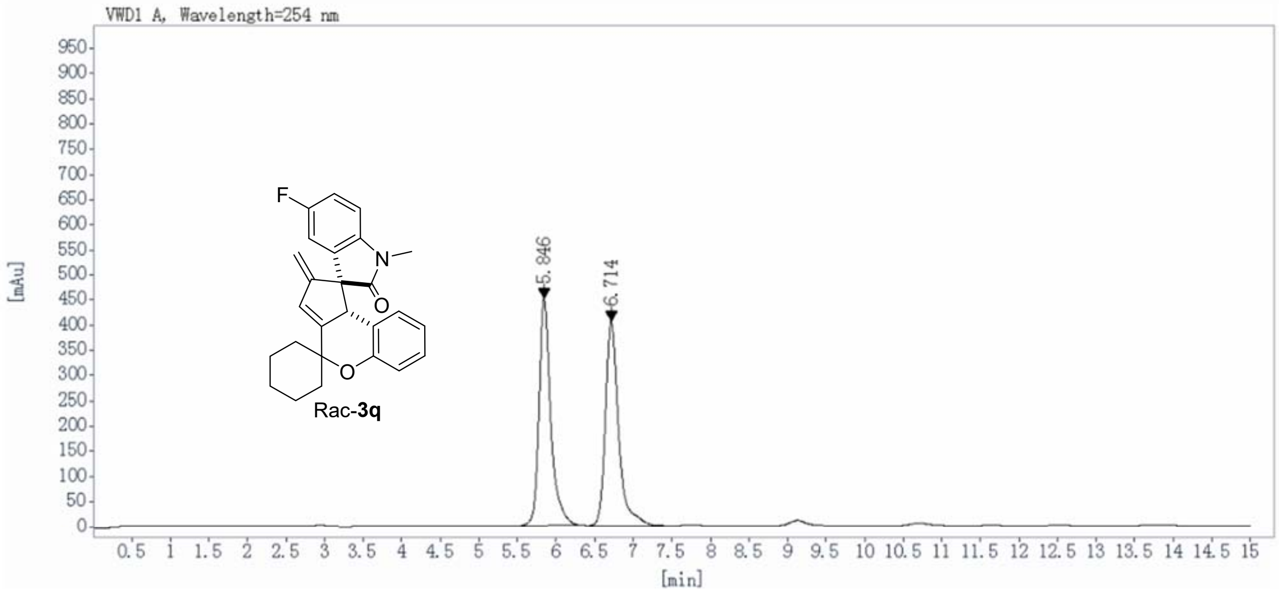

Rac-**3q**

Ret Time [min]	Peak Type	Width [min]	Height [mAU]	Area [mAU*s]	Area [%]
5.846	BBA	0.16	451.7264	4895.7183	50.3676
6.714	BBA	0.18	405.9886	4824.2539	49.6324
			Totals:	9719.9722	100.0000

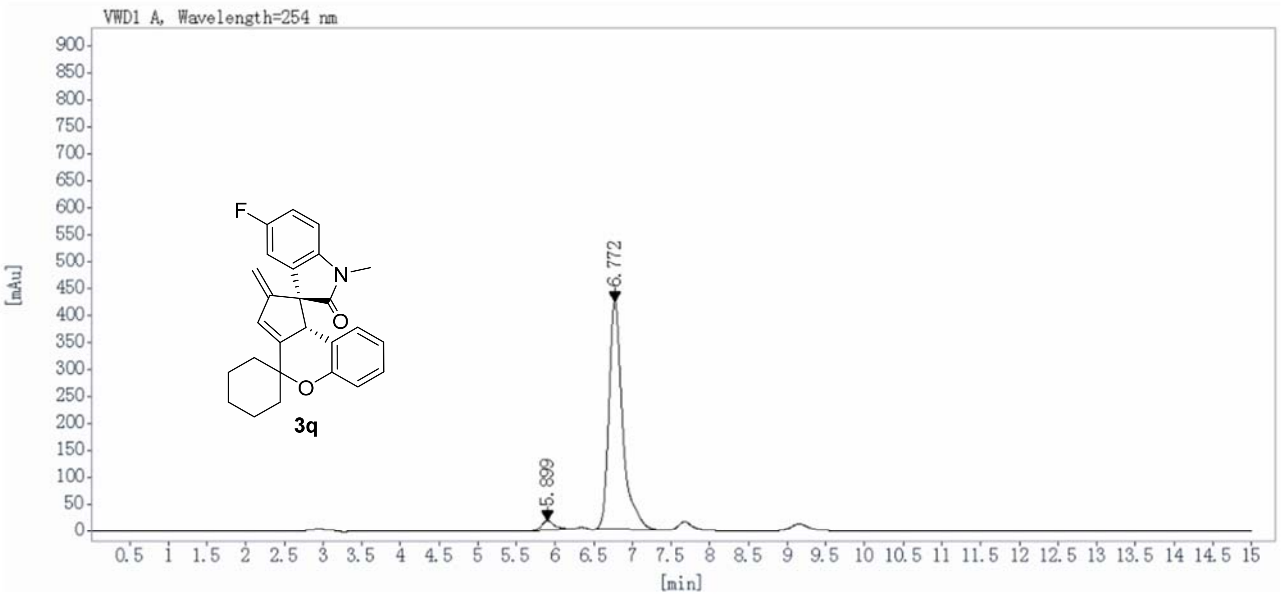

Ret Time [min]	Peak Type	Width [min]	Height [mAU]	Area [mAU*s]	Area [%]
5.899	BBA	0.17	18.3742	204.3898	3.7710
6.772	BB	0.18	423.1734	5215.7002	96.2290
			Totals:	5420.0900	100.0000

Spectrum from 20230705.wiff2 (sample 73) - 68, +TOF MS (100 - 600) from 0.053 to 0.114 min, noise filtere...2 min, noise filtered (noise multiplier = 1.5), Gaussian smoothed (0.5 points)], Recalibrated, centroided

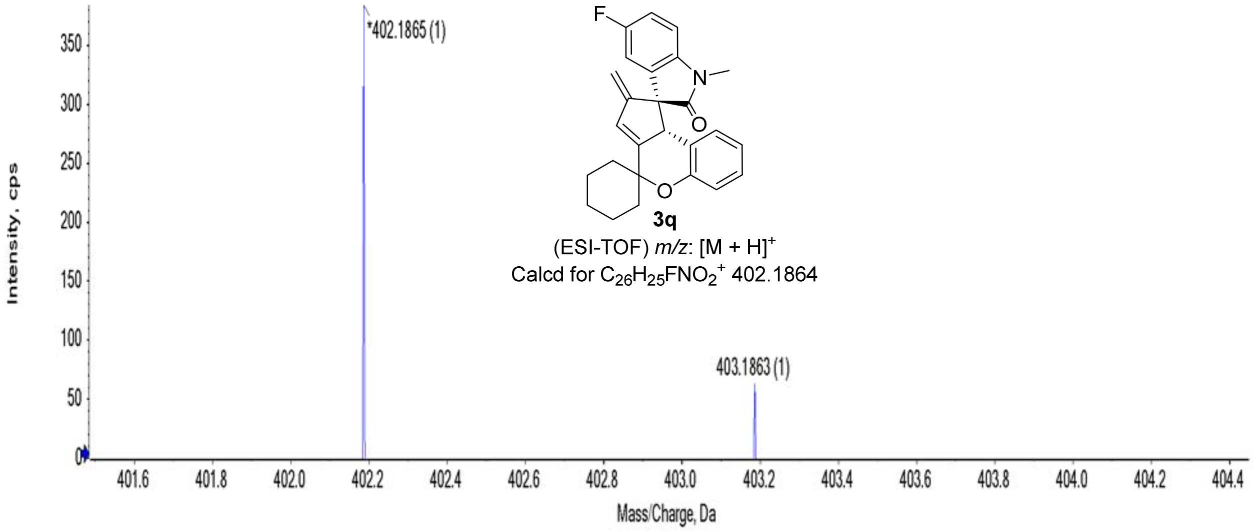

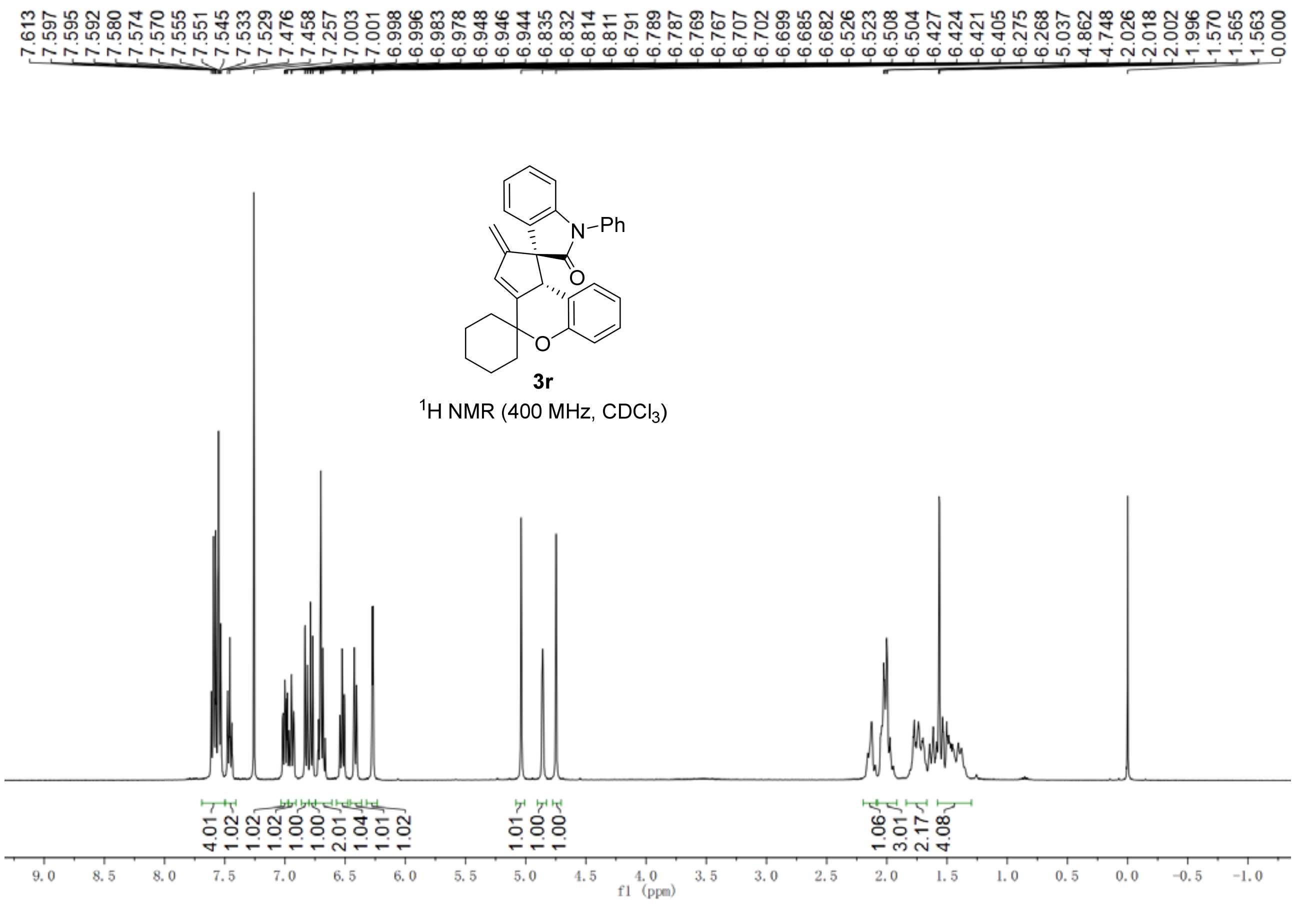

3r
^{1}H NMR (400 MHz, $CDCl_3$)

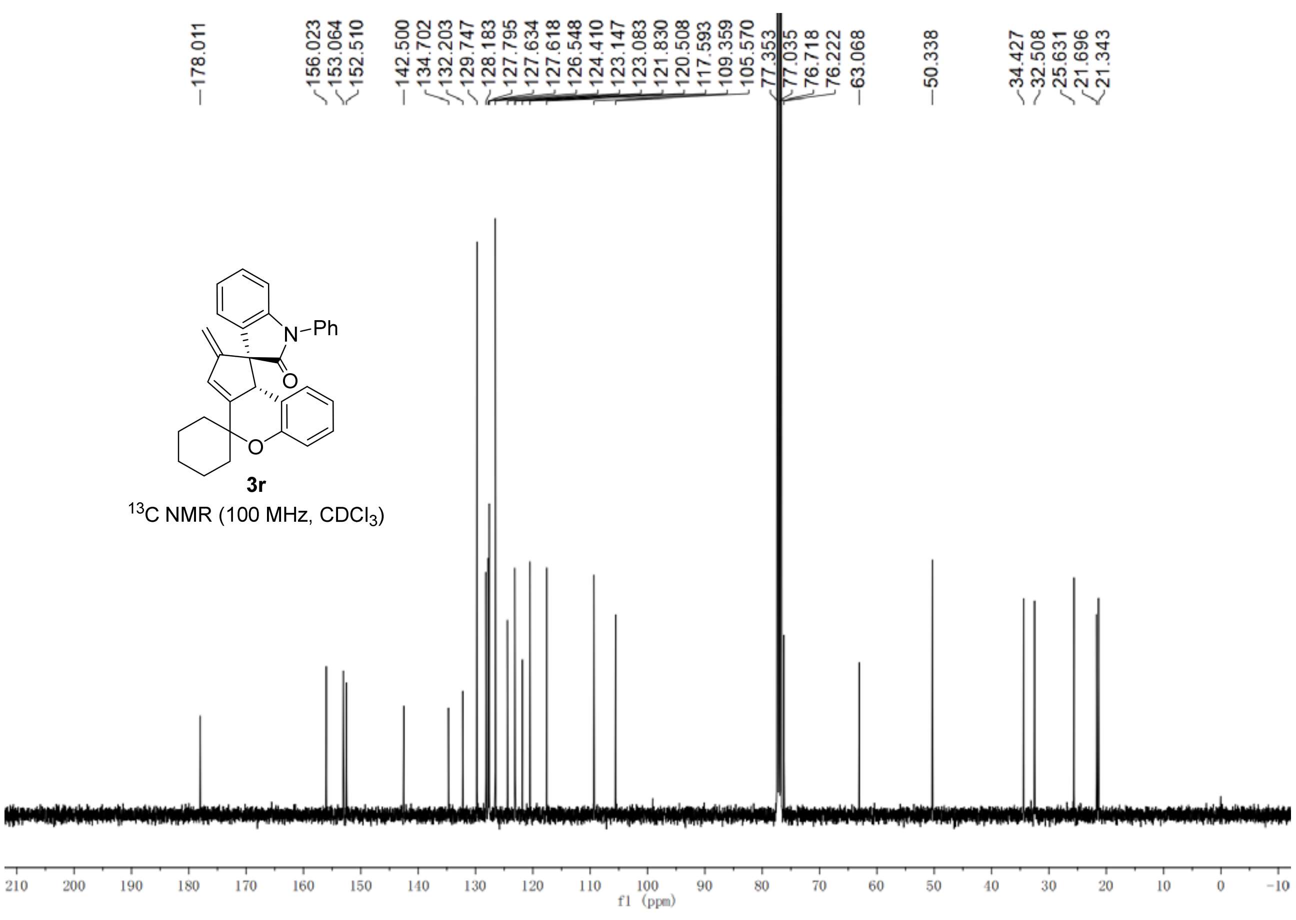

3r
^{13}C NMR (100 MHz, $CDCl_3$)

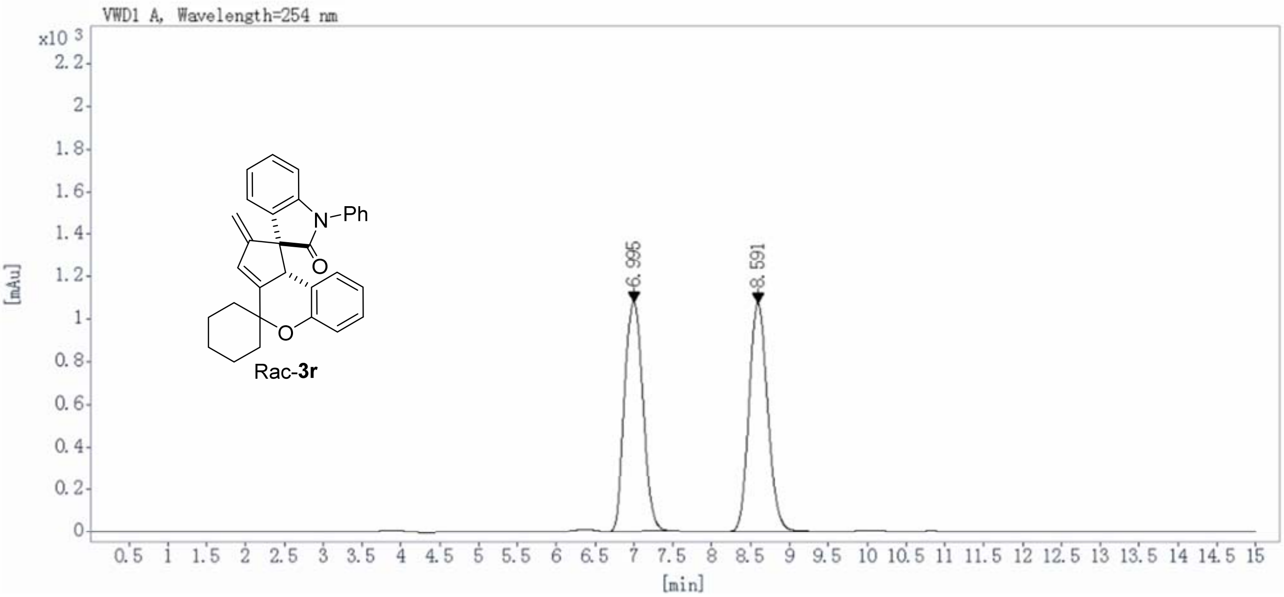

Ret Time [min]	Peak Type	Width [min]	Height [mAU]	Area [mAU*s]	Area [%]
6.995	BBA	0.27	1077.7903	17849.4648	49.7720
8.591	BBA	0.26	1073.0360	18012.9883	50.2280
			Totals:	35862.4531	100.0000

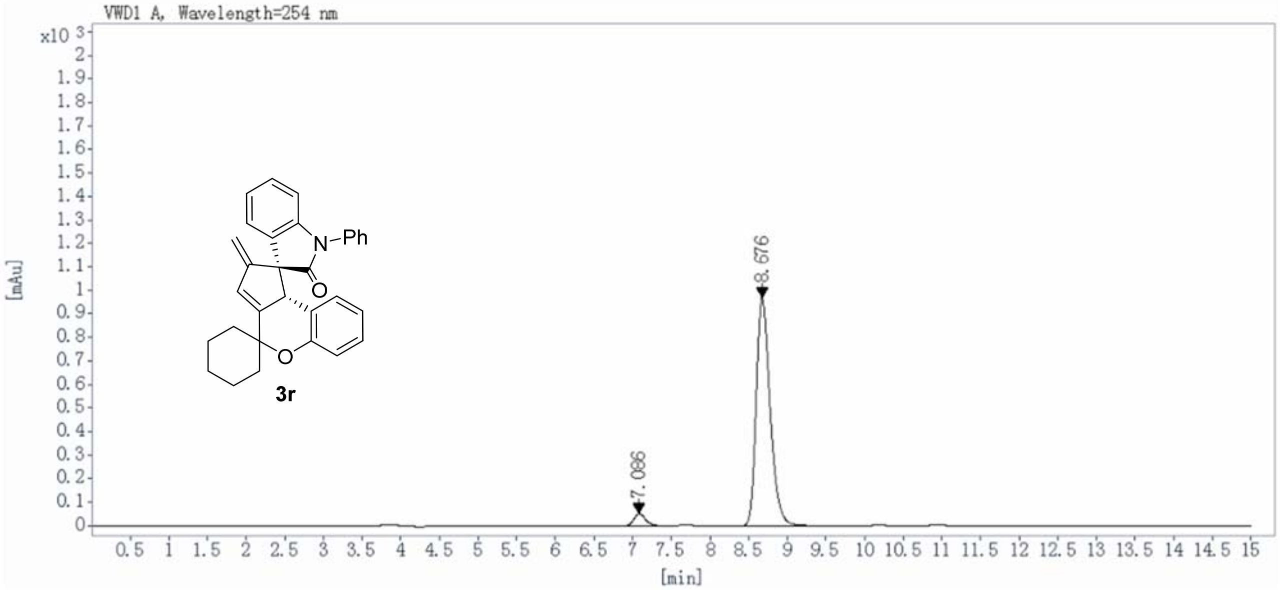

Ret Time [min]	Peak Type	Width [min]	Height [mAU]	Area [mAU*s]	Area [%]
7.086	BB	0.16	51.1723	542.4835	4.3210
8.676	BBA	0.19	969.4561	12012.2246	95.6790
			Totals:	12554.7081	100.0000

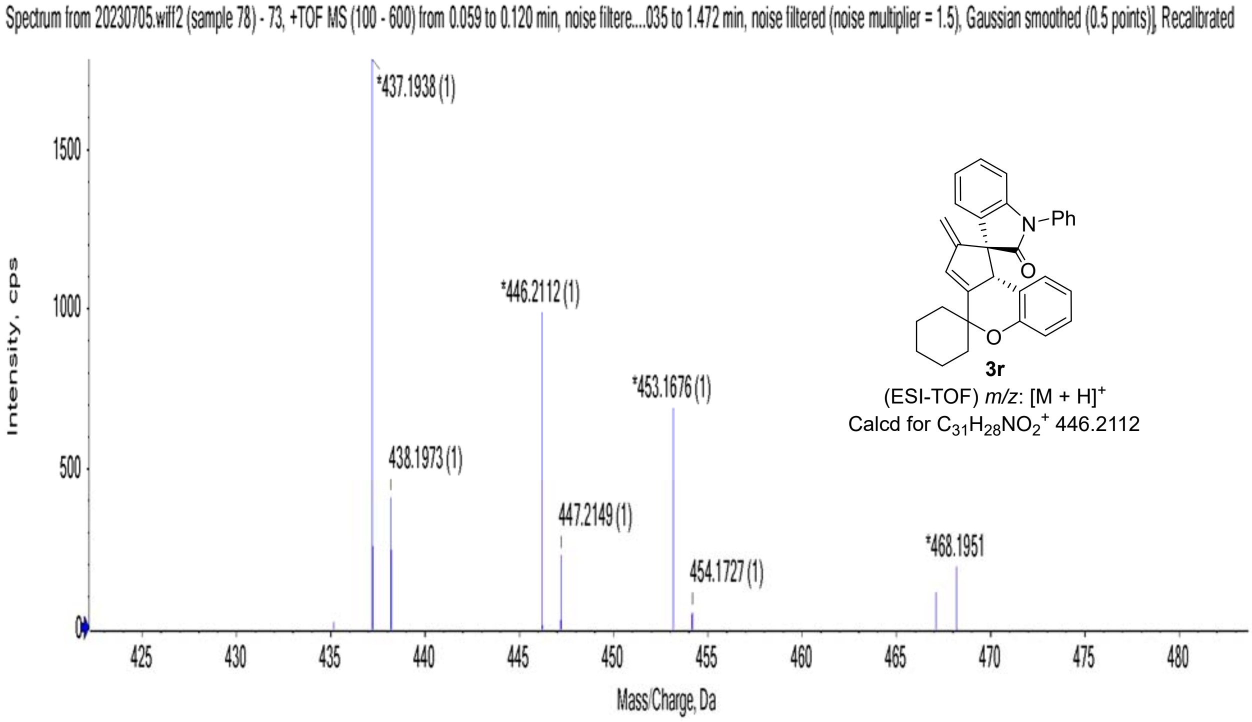
Spectrum from 20230705.wiff2 (sample 78) - 73, +TOF MS (100 - 600) from 0.059 to 0.120 min, noise filtere....035 to 1.472 min, noise filtered (noise multiplier = 1.5), Gaussian smoothed (0.5 points)], Recalibrated
Intensity, cps
1500
1000
500
0
*437.1938 (1)
438.1973 (1)
*446.2112 (1)
447.2149 (1)
*453.1676 (1)
454.1727 (1)
*468.1951
425
430
435
440
445
450
455
460
465
470
475
480
Mass/Charge, Da
N–Ph
O
O
3r
(ESI-TOF) m/z: [M + H]+
Calcd for C31H28NO2+ 446.2112

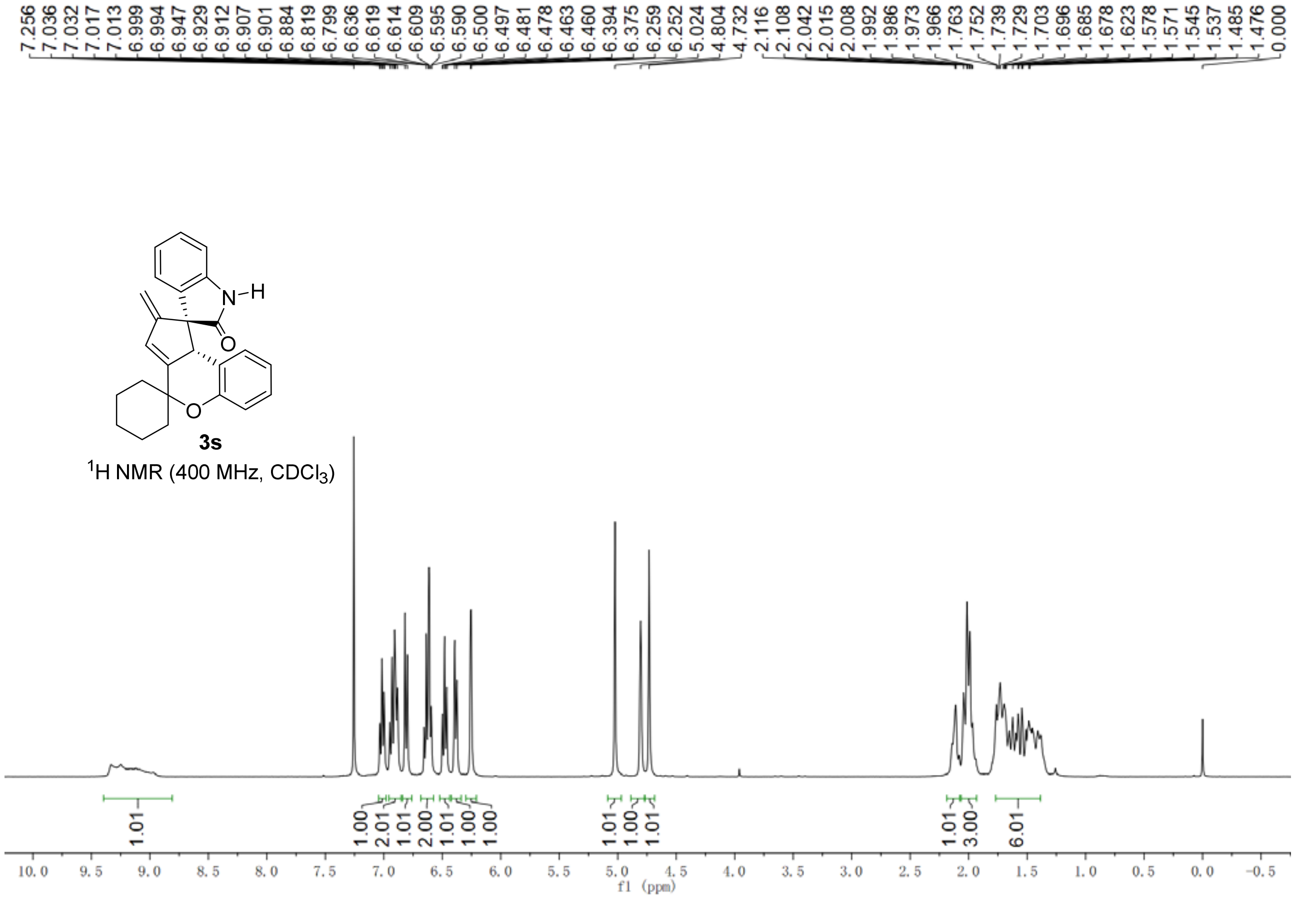

3s

^{1}H NMR (400 MHz, $CDCl_3$)

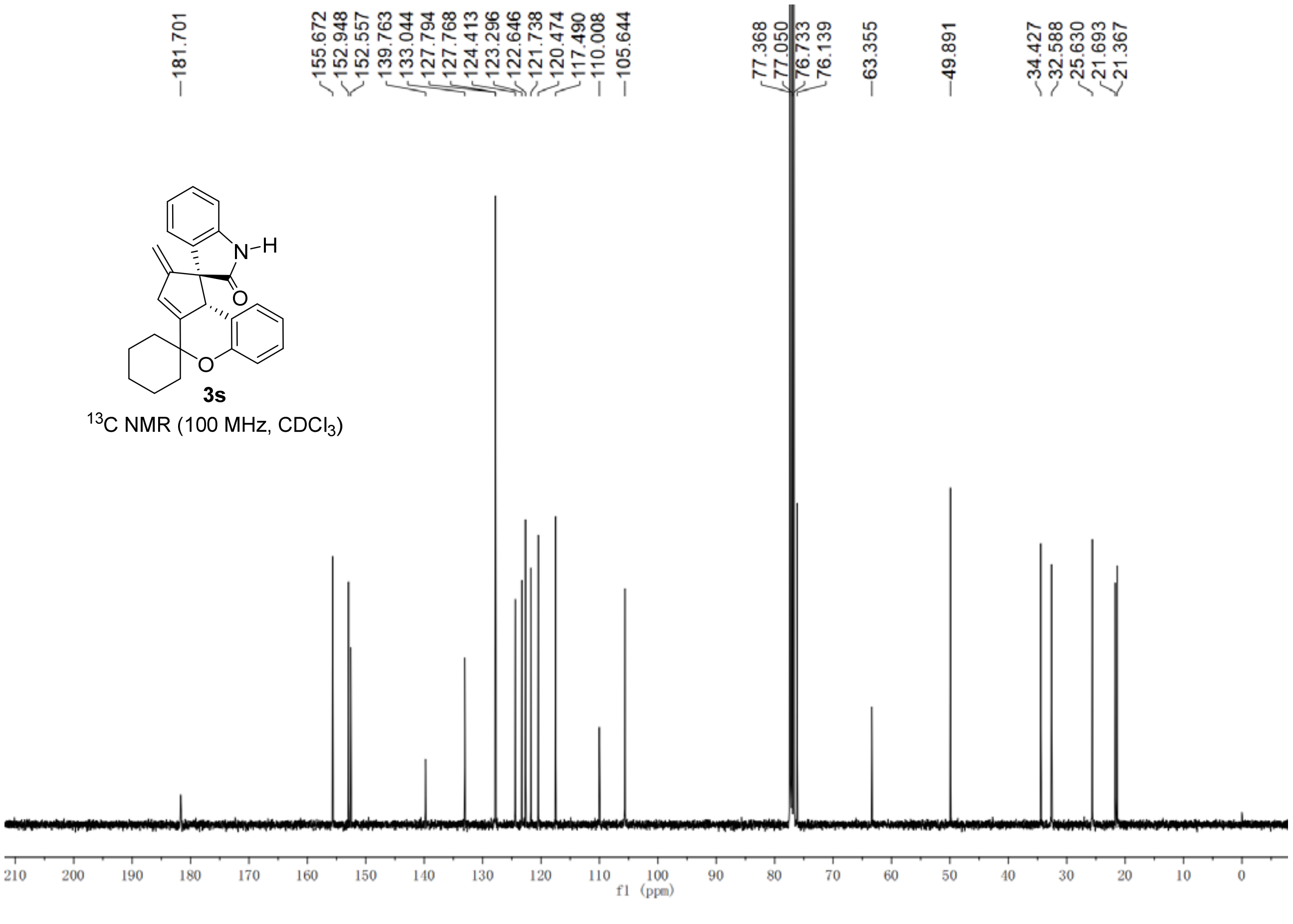

3s

^{13}C NMR (100 MHz, $CDCl_3$)

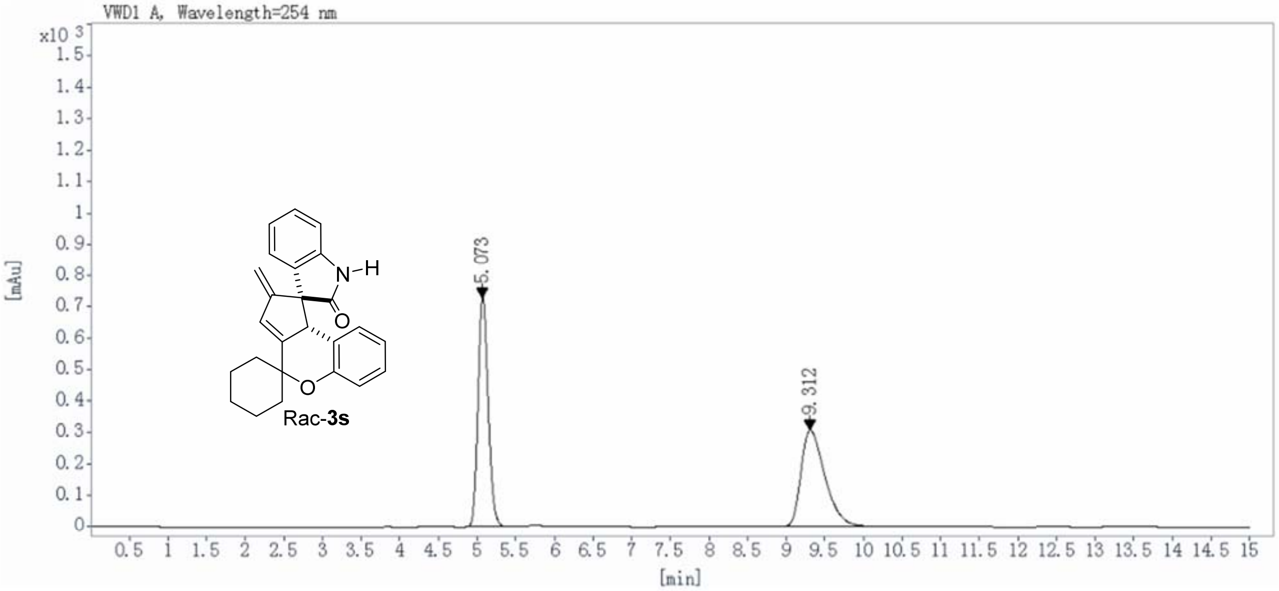

Ret Time [min]	Peak Type	Width [min]	Height [mAU]	Area [mAU*s]	Area [%]
5.073	BBA	0.14	726.8414	6615.3110	49.9077
9.312	BBA	0.33	308.0536	6639.7852	50.0923
			Totals:	13255.0962	100.0000

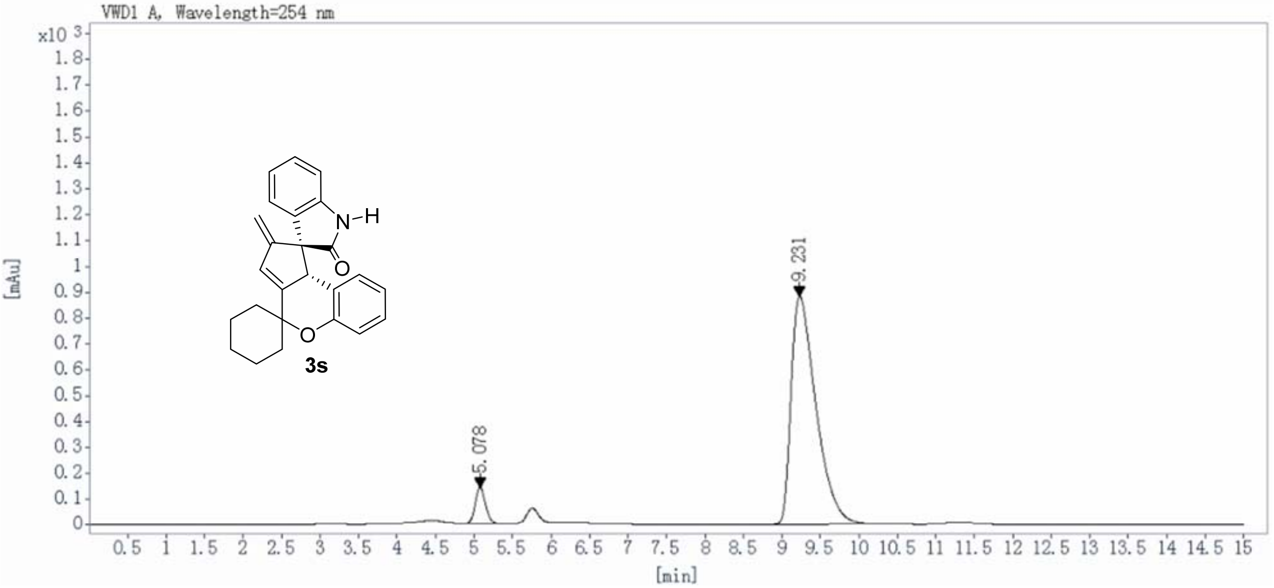

Ret Time [min]	Peak Type	Width [min]	Height [mAU]	Area [mAU*s]	Area [%]
5.078	BBA	0.14	140.4959	1284.6090	6.1001
9.231	BBA	0.34	882.8427	19774.2598	93.8999
			Totals:	21058.8688	100.0000

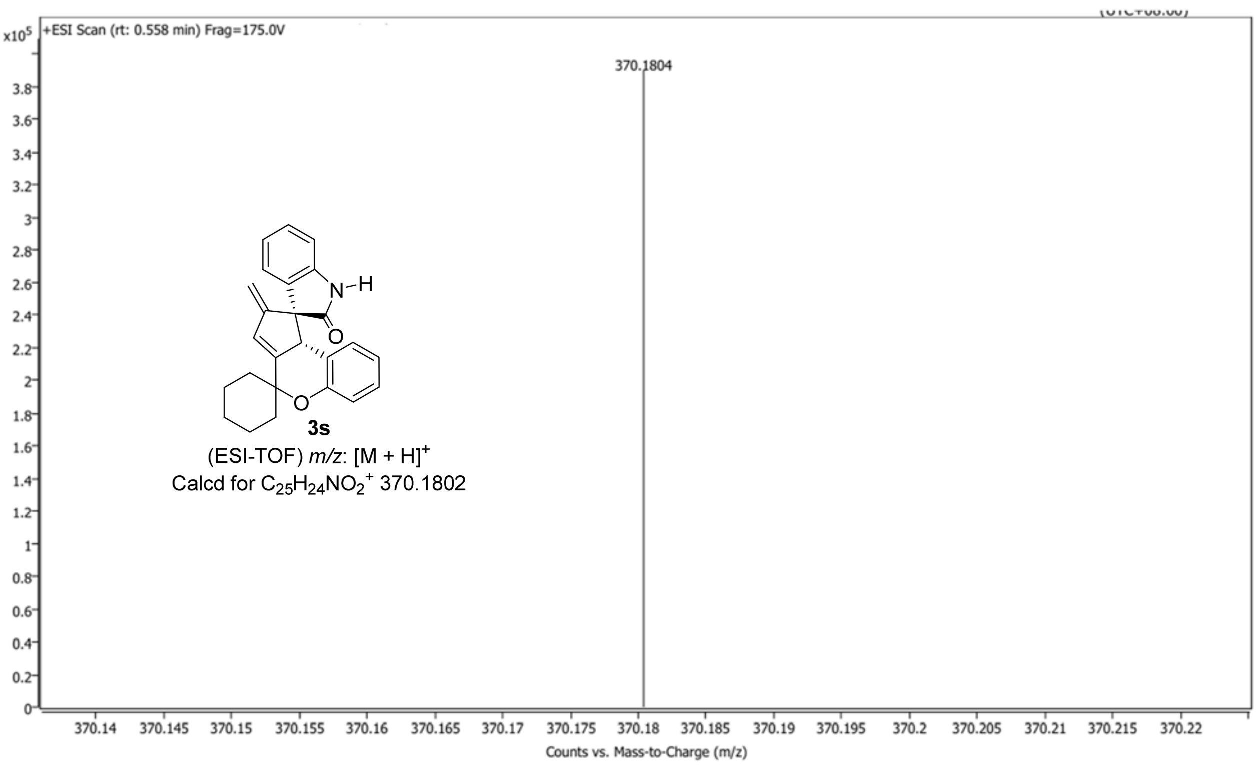
+ESI Scan (rt: 0.558 min) Frag=175.0V
x10^5
370.1804
3s
(ESI-TOF) m/z: [M + H]+
Calcd for C25H24NO2+ 370.1802
Counts vs. Mass-to-Charge (m/z)

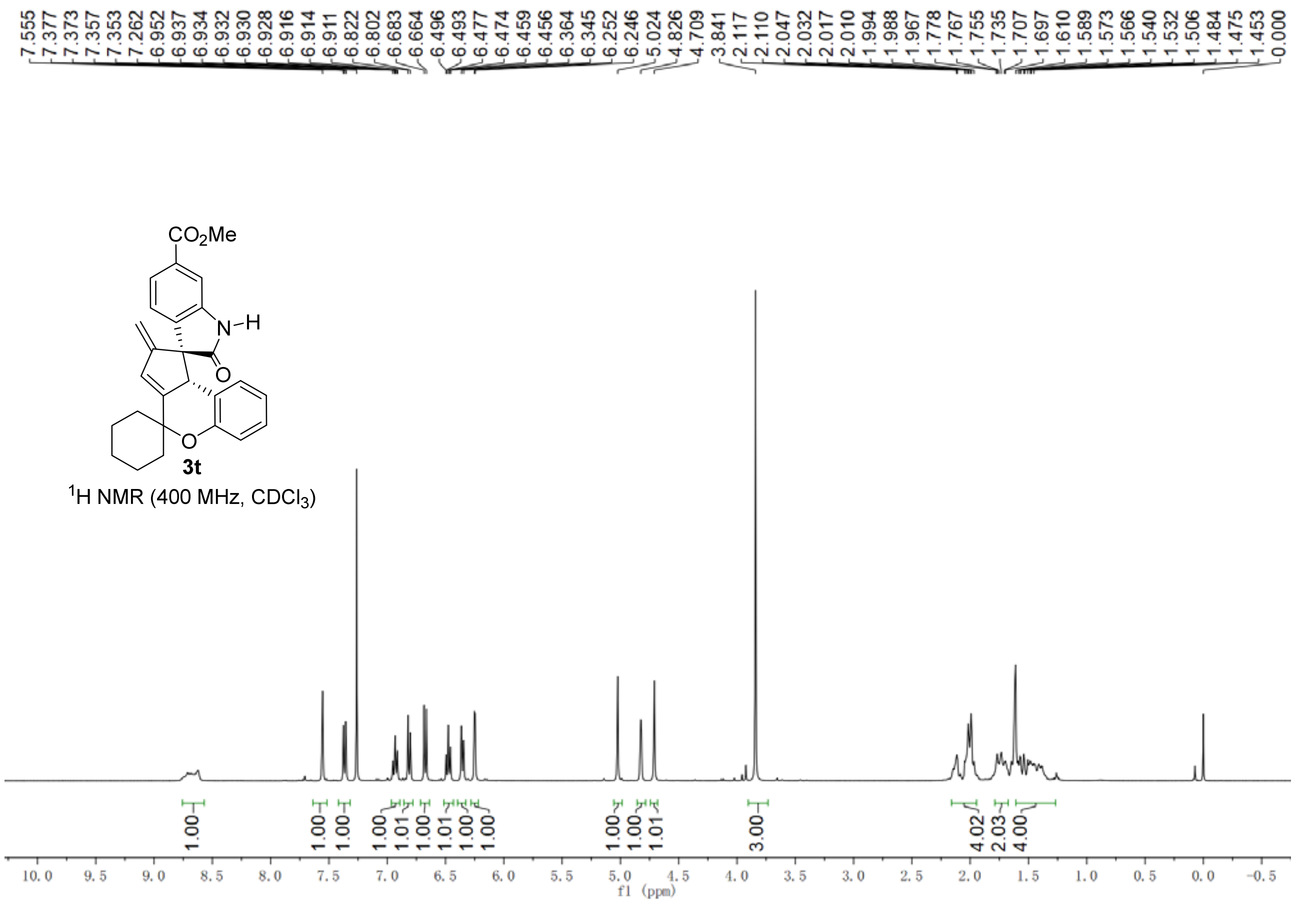
7.555 7.377 7.373 7.357 7.353 7.262 6.952 6.937 6.934 6.932 6.930 6.928 6.916 6.914 6.911 6.822 6.802 6.683 6.664 6.496 6.493 6.477 6.474 6.459 6.456 6.364 6.345 6.252 6.246 5.024 4.826 4.709 3.841 2.117 2.110 2.047 2.032 2.017 2.010 1.994 1.988 1.967 1.778 1.767 1.755 1.735 1.707 1.697 1.610 1.589 1.573 1.566 1.540 1.532 1.506 1.484 1.475 1.453 0.000
CO2Me
N–H
O
3t
1H NMR (400 MHz, CDCl3)
1.00 1.00 1.00 1.00 1.01 1.00 1.01 1.00 1.00 1.00 1.00 1.01 3.00 4.02 2.03 4.00
f1 (ppm)

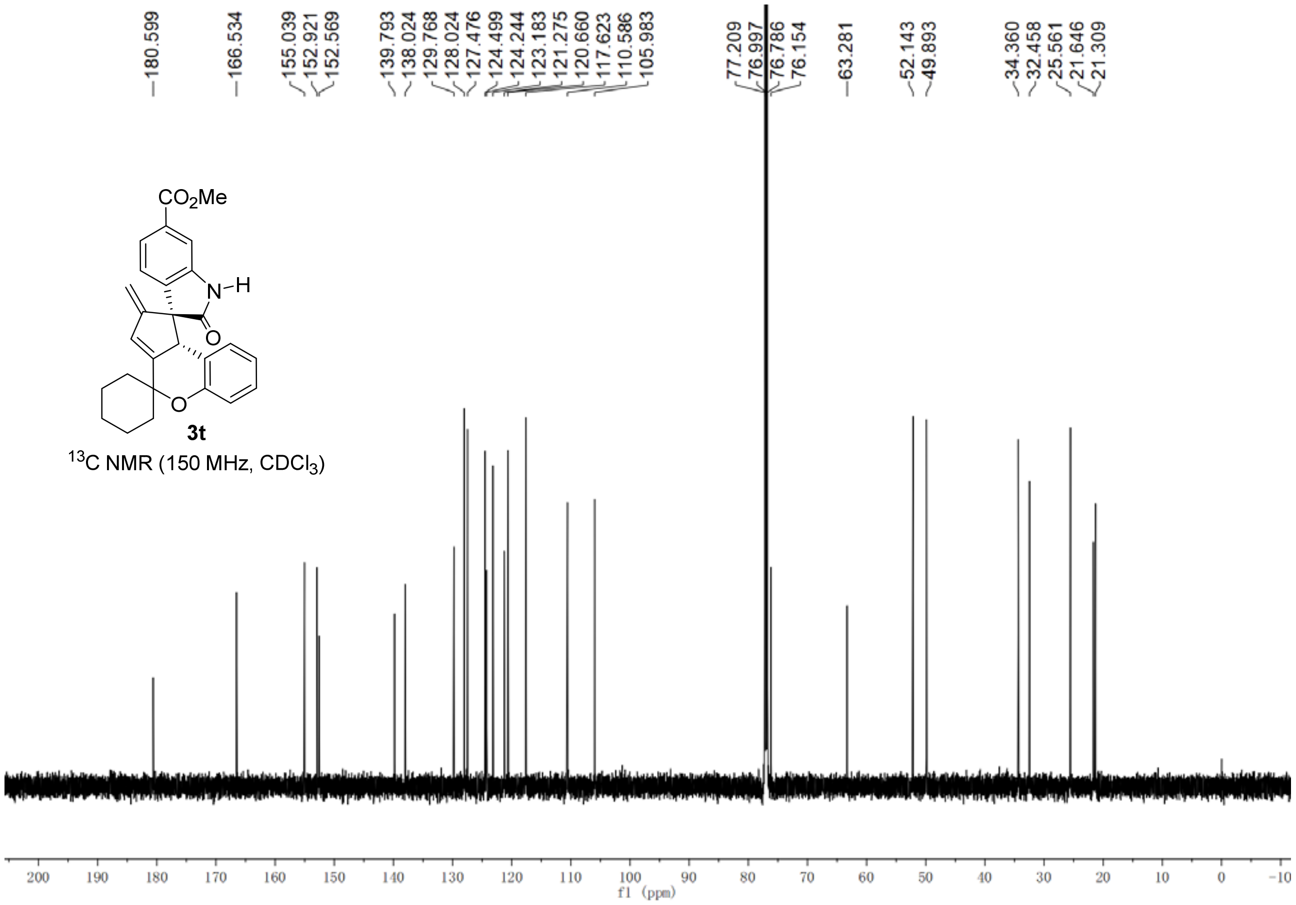
180.599 166.534 155.039 152.921 152.569 139.793 138.024 129.768 128.024 127.476 124.499 124.244 123.183 121.275 120.660 117.623 110.586 105.983 77.209 76.997 76.786 76.154 63.281 52.143 49.893 34.360 32.458 25.561 21.646 21.309
CO2Me
N–H
O
3t
13C NMR (150 MHz, CDCl3)
f1 (ppm)

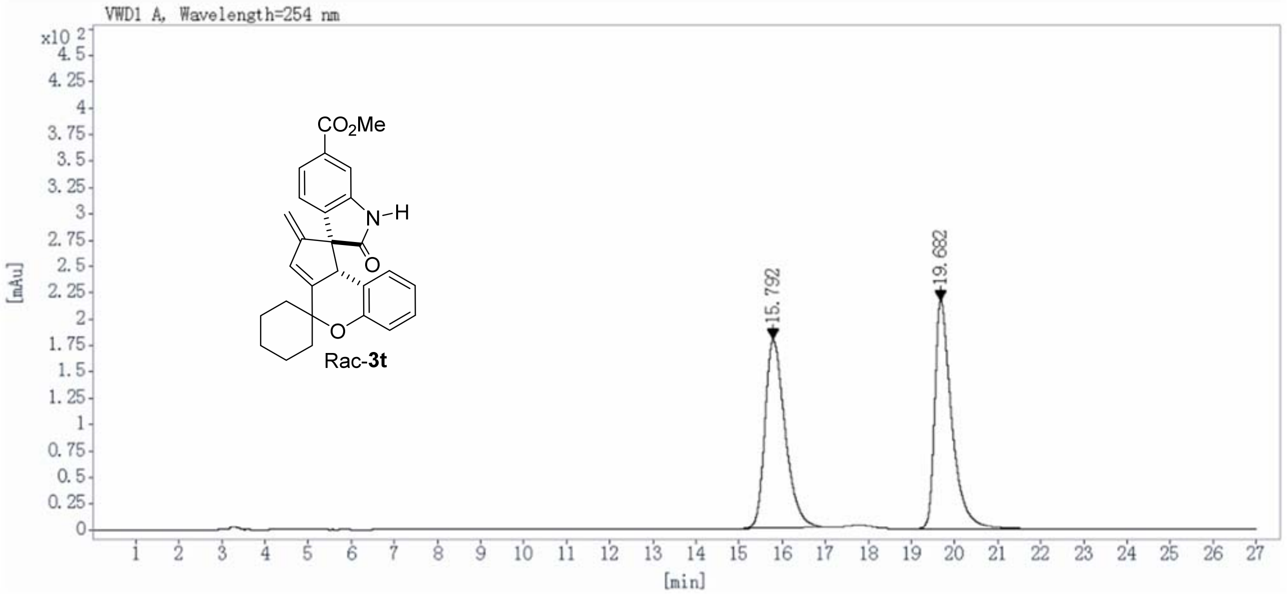

Ret Time [min]	Peak Type	Width [min]	Height [mAU]	Area [mAU*s]	Area [%]
15.792	BB	0.50	179.2602	5878.5820	49.5607
19.682	BBA	0.41	217.1251	5982.7925	50.4393
			Totals:	11861.3745	100.0000

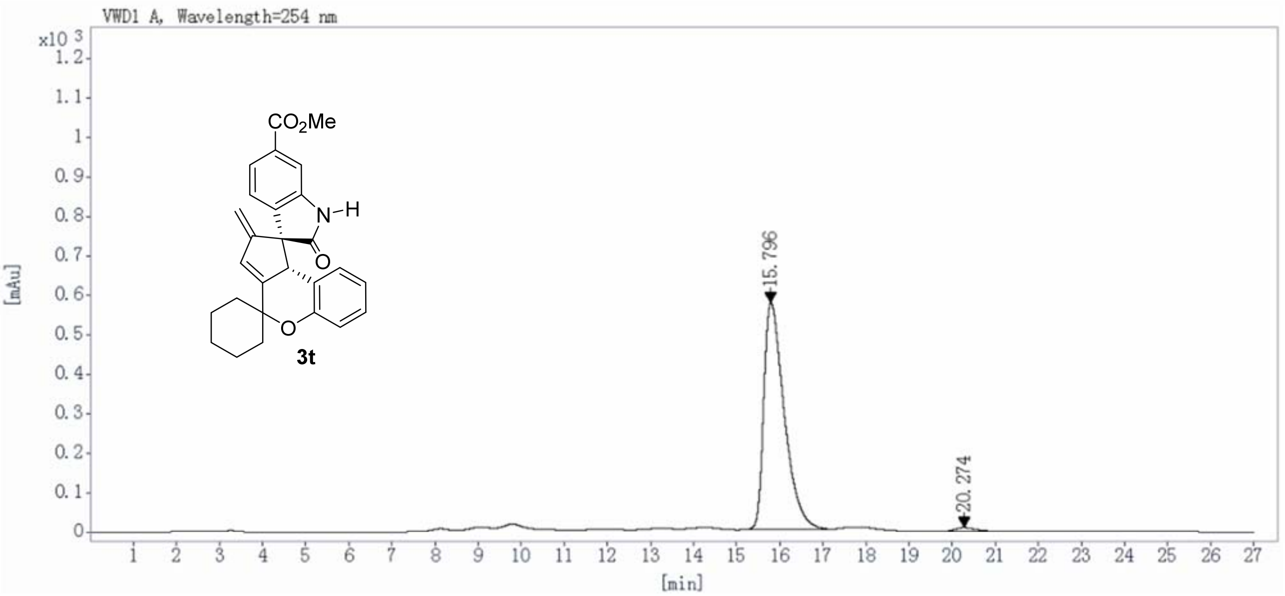

Ret Time [min]	Peak Type	Width [min]	Height [mAU]	Area [mAU*s]	Area [%]
15.796	BBA	0.50	575.8636	18961.9141	98.2026
20.274	BBA	0.52	10.0001	347.0662	1.7974
			Totals:	19308.9803	100.0000

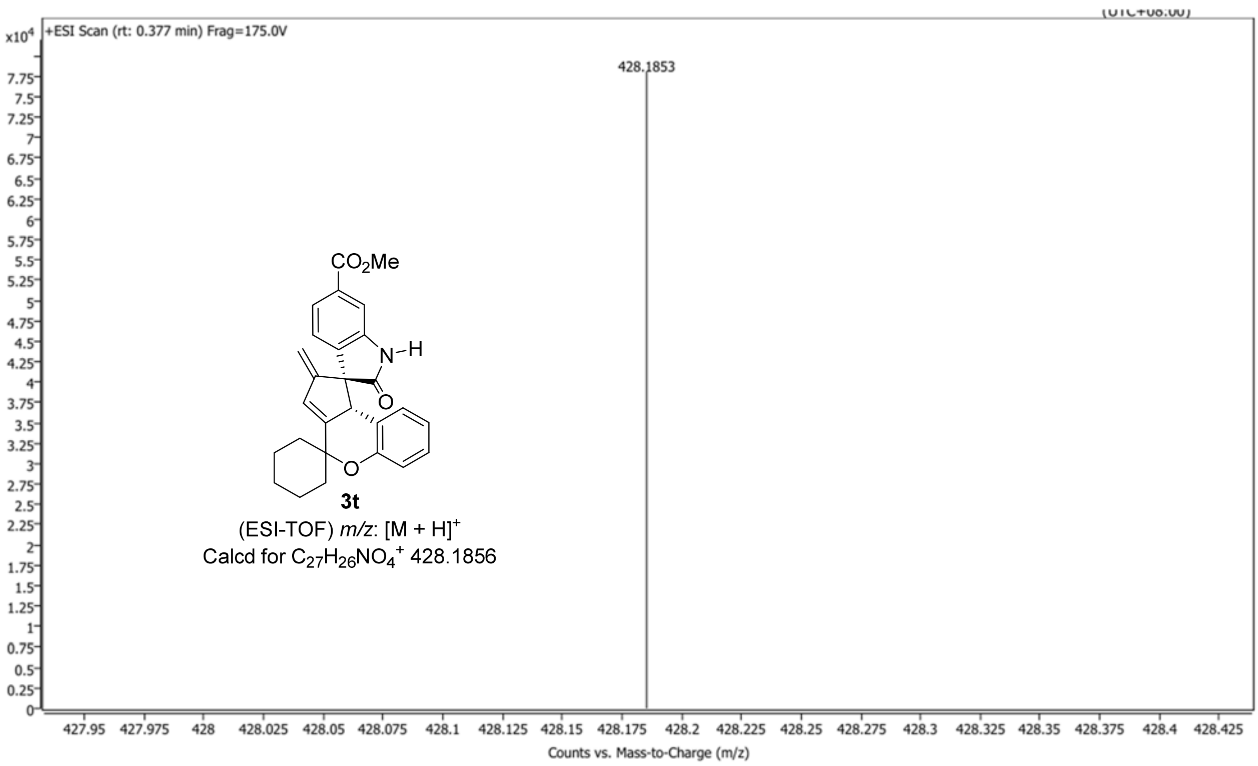
(UTC+08:00)
+ESI Scan (rt: 0.377 min) Frag=175.0V
x10^4
428.1853
CO2Me
N–H
O
O
3t
(ESI-TOF) m/z: [M + H]+
Calcd for C27H26NO4+ 428.1856
Counts vs. Mass-to-Charge (m/z)

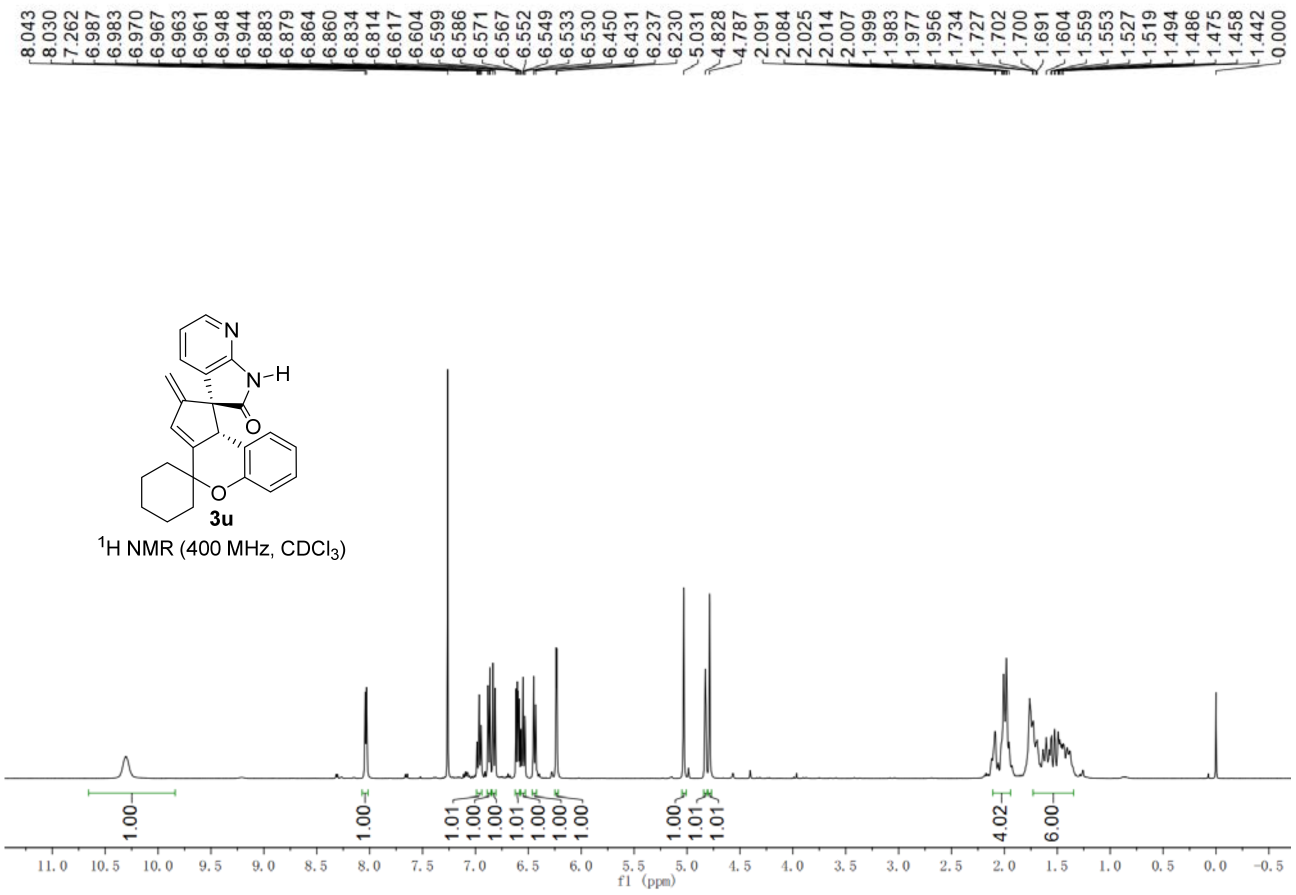

3u
^{1}H NMR (400 MHz, $CDCl_3$)

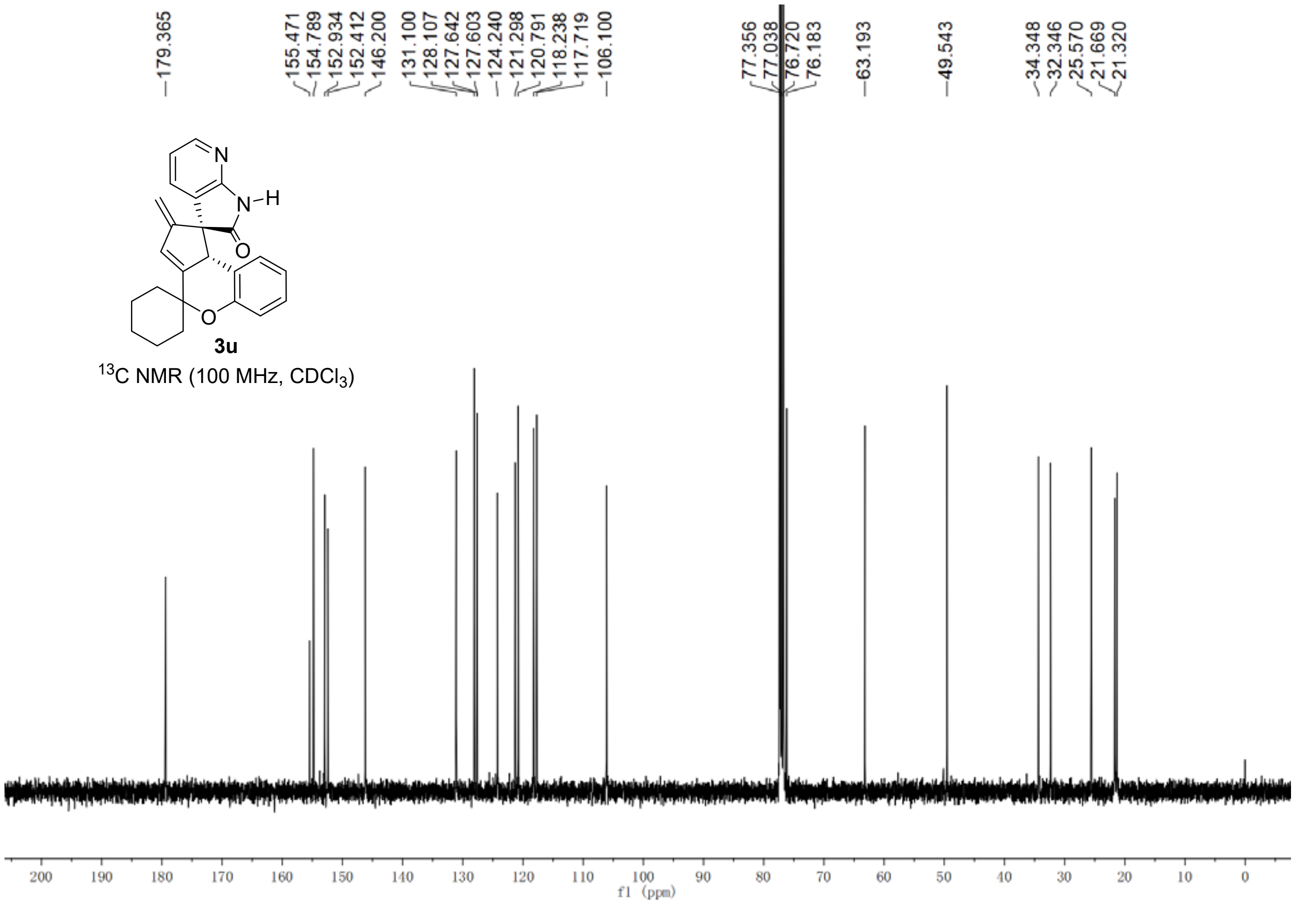

3u
^{13}C NMR (100 MHz, $CDCl_3$)

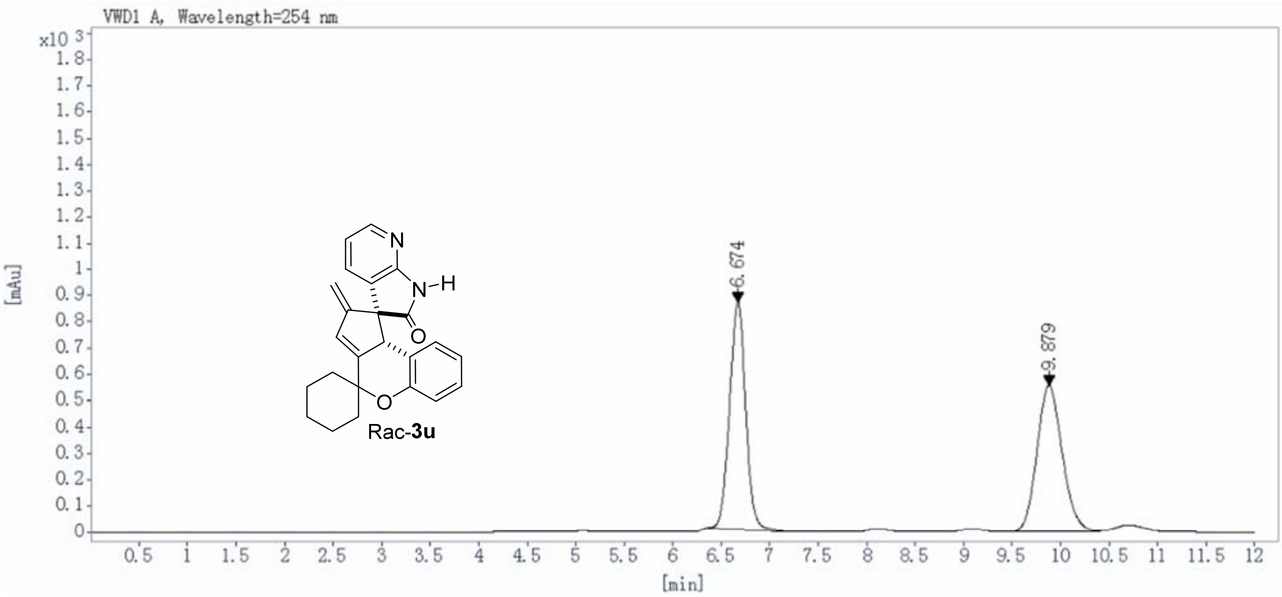

Ret Time [min]	Peak Type	Width [min]	Height [mAU]	Area [mAU*s]	Area [%]
6.674	BBA	0.18	864.6375	10019.2031	49.9140
9.879	BB	0.28	553.8257	10053.7139	50.0860
			Totals:	20072.9170	100.0000

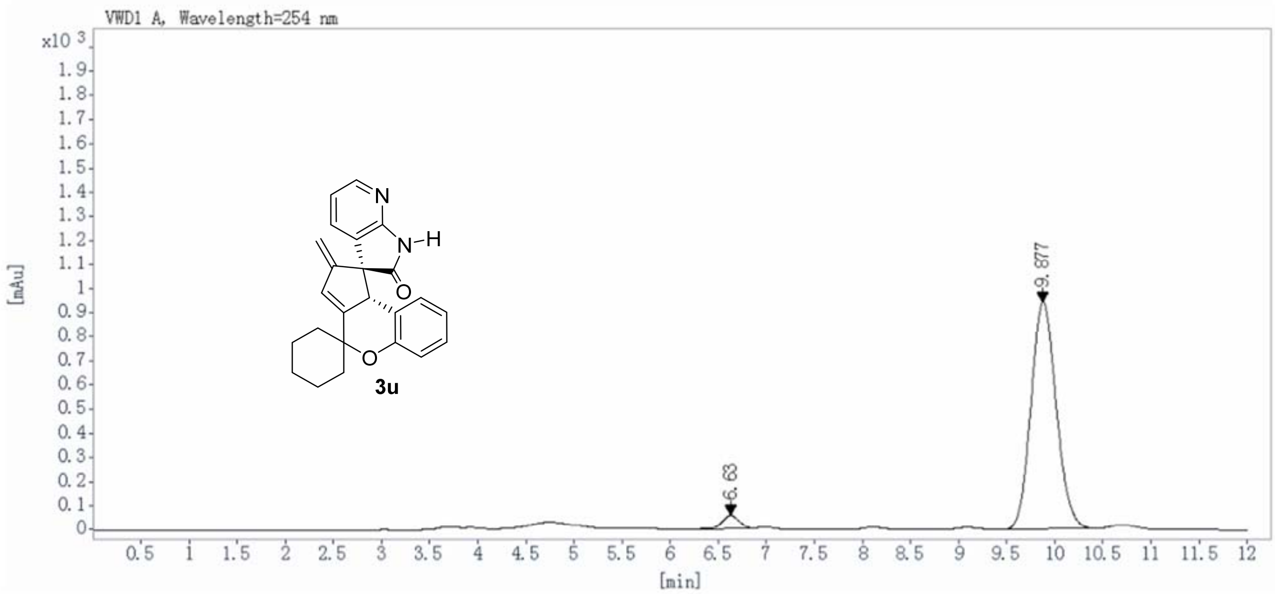

Ret Time [min]	Peak Type	Width [min]	Height [mAU]	Area [mAU*s]	Area [%]
6.630	BB	0.18	54.6443	641.9444	3.5939
9.877	BB	0.29	940.9167	17220.1152	96.4061
			Totals:	17862.0596	100.0000

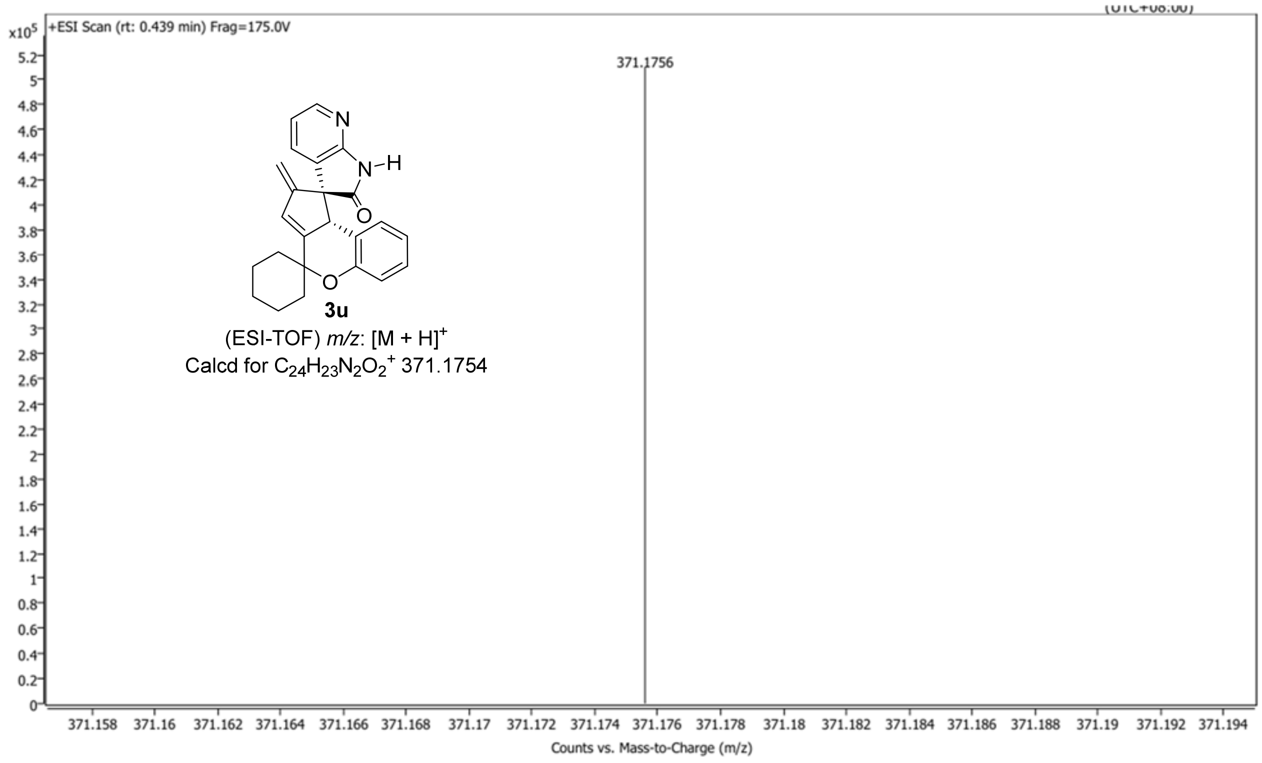
+ESI Scan (rt: 0.439 min) Frag=175.0V
x10^5
371.1756
3u
(ESI-TOF) m/z: [M + H]+
Calcd for C24H23N2O2+ 371.1754
Counts vs. Mass-to-Charge (m/z)

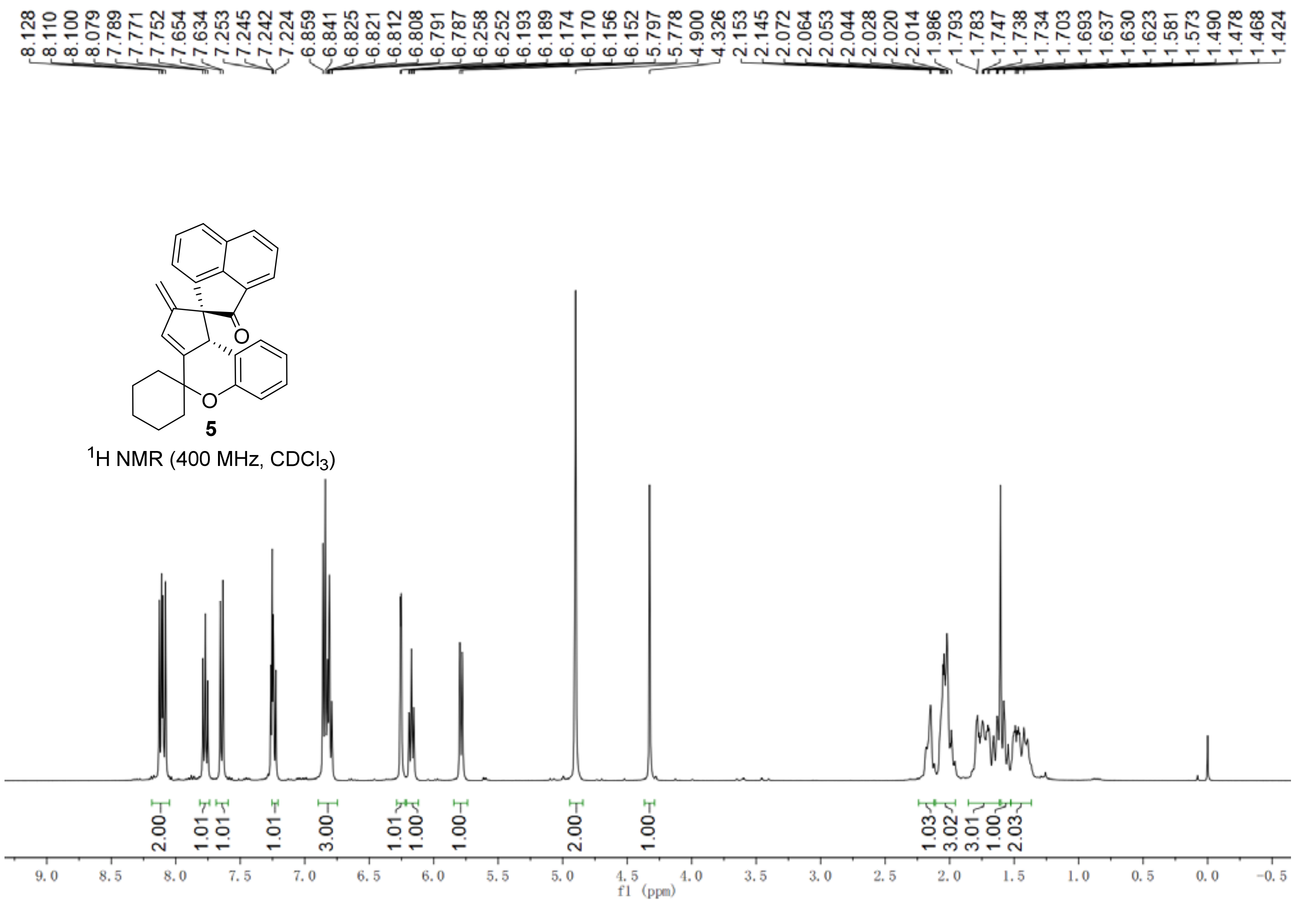
8.128
8.110
8.100
8.079
7.789
7.771
7.752
7.654
7.634
7.253
7.245
7.242
7.224
6.859
6.841
6.825
6.821
6.812
6.808
6.791
6.787
6.258
6.252
6.193
6.189
6.174
6.170
6.156
6.152
5.797
5.778
4.900
4.326
2.153
2.145
2.072
2.064
2.053
2.044
2.028
2.020
2.014
1.986
1.793
1.783
1.747
1.738
1.734
1.703
1.693
1.637
1.630
1.623
1.581
1.573
1.490
1.478
1.468
1.424
O
O
5
^{1}H NMR (400 MHz, $CDCl_3$)
2.00
1.01
1.01
1.01
3.00
1.01
1.00
1.00
2.00
1.00
1.03
3.02
3.01
1.00
2.03
9.0 8.5 8.0 7.5 7.0 6.5 6.0 5.5 5.0 4.5 4.0 3.5 3.0 2.5 2.0 1.5 1.0 0.5 0.0 -0.5
f1 (ppm)

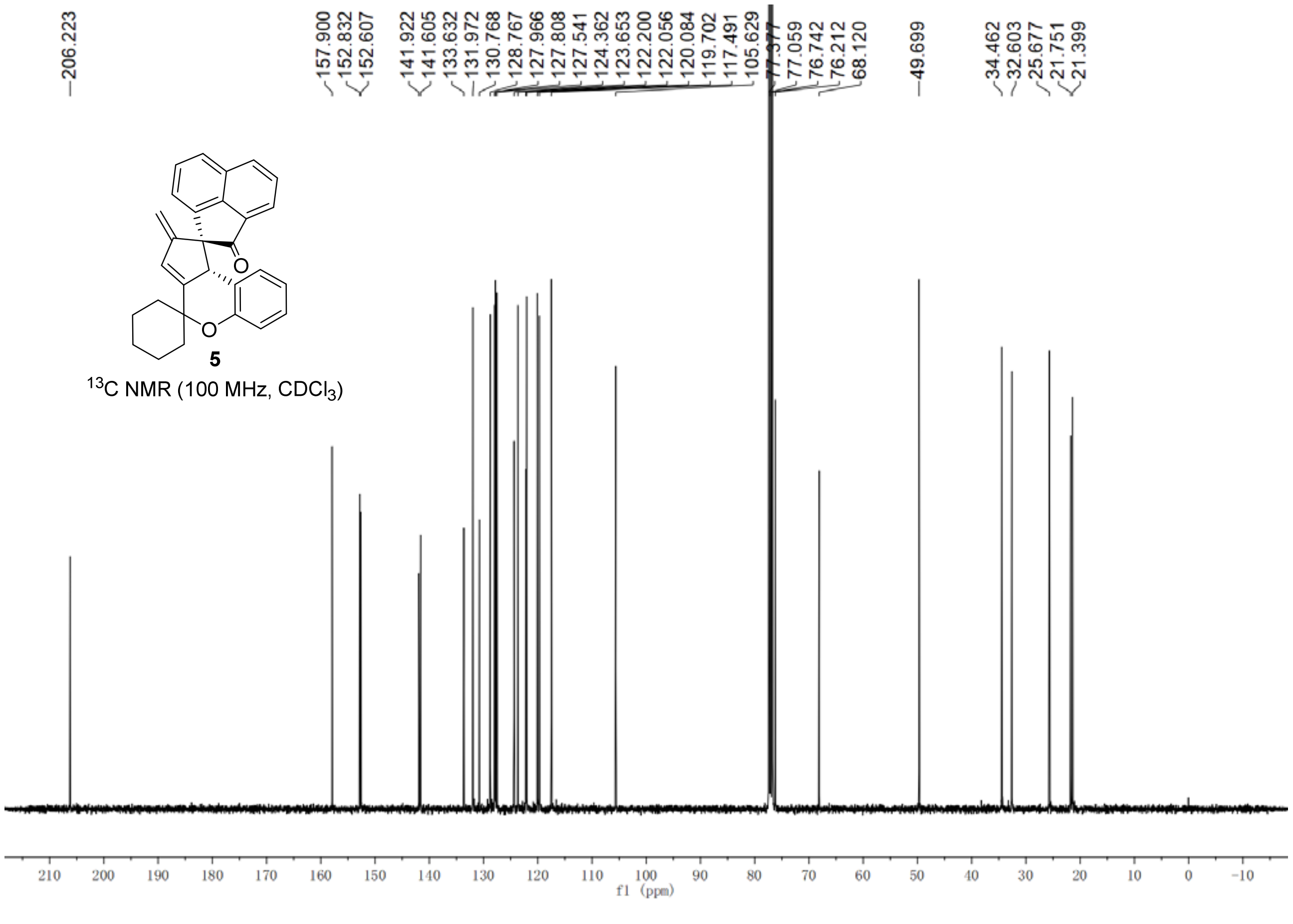
206.223
157.900
152.832
152.607
141.922
141.605
133.632
131.972
130.768
128.767
127.966
127.808
127.541
124.362
123.653
122.200
122.056
120.084
119.702
117.491
105.629
77.377
77.059
76.742
76.212
68.120
49.699
34.462
32.603
25.677
21.751
21.399
O
O
5
^{13}C NMR (100 MHz, $CDCl_3$)
210 200 190 180 170 160 150 140 130 120 110 100 90 80 70 60 50 40 30 20 10 0 -10
f1 (ppm)

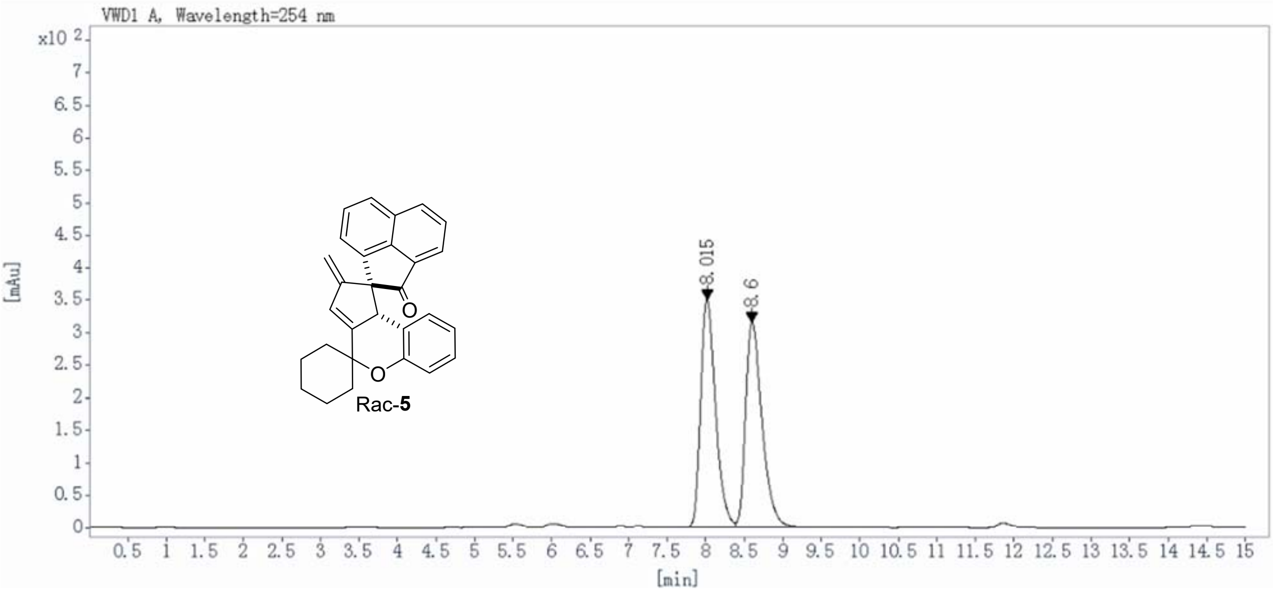

Ret Time [min]	Peak Type	Width [min]	Height [mAU]	Area [mAU*s]	Area [%]
8.015	BV	0.21	350.7008	4718.6255	49.9323
8.600	VBA	0.23	315.7699	4731.4258	50.0677
			Totals:	9450.0513	100.0000

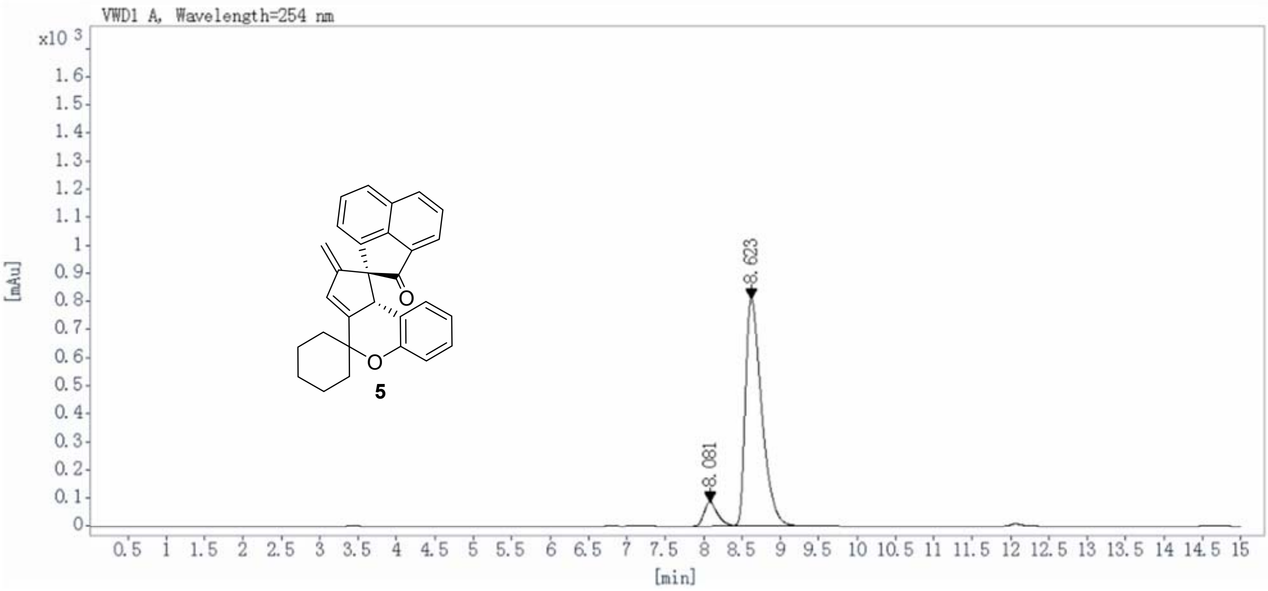

Ret Time [min]	Peak Type	Width [min]	Height [mAU]	Area [mAU*s]	Area [%]
8.081	BV	0.19	86.4970	1072.3313	8.3051
8.623	VBA	0.22	809.1938	11839.3779	91.6949
			Totals:	12911.7092	100.0000

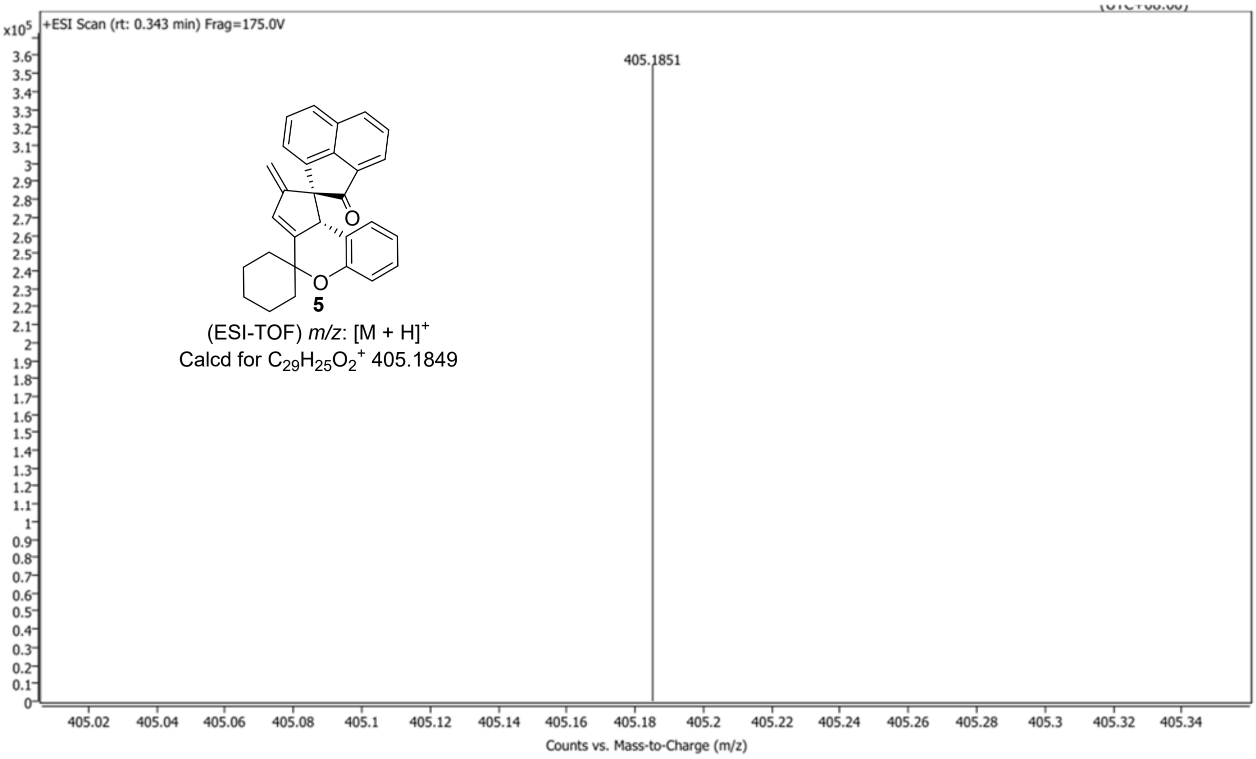
+ESI Scan (rt: 0.343 min) Frag=175.0V
x10^5
405.1851
5
(ESI-TOF) m/z: [M + H]+
Calcd for C29H25O2+ 405.1849
Counts vs. Mass-to-Charge (m/z)

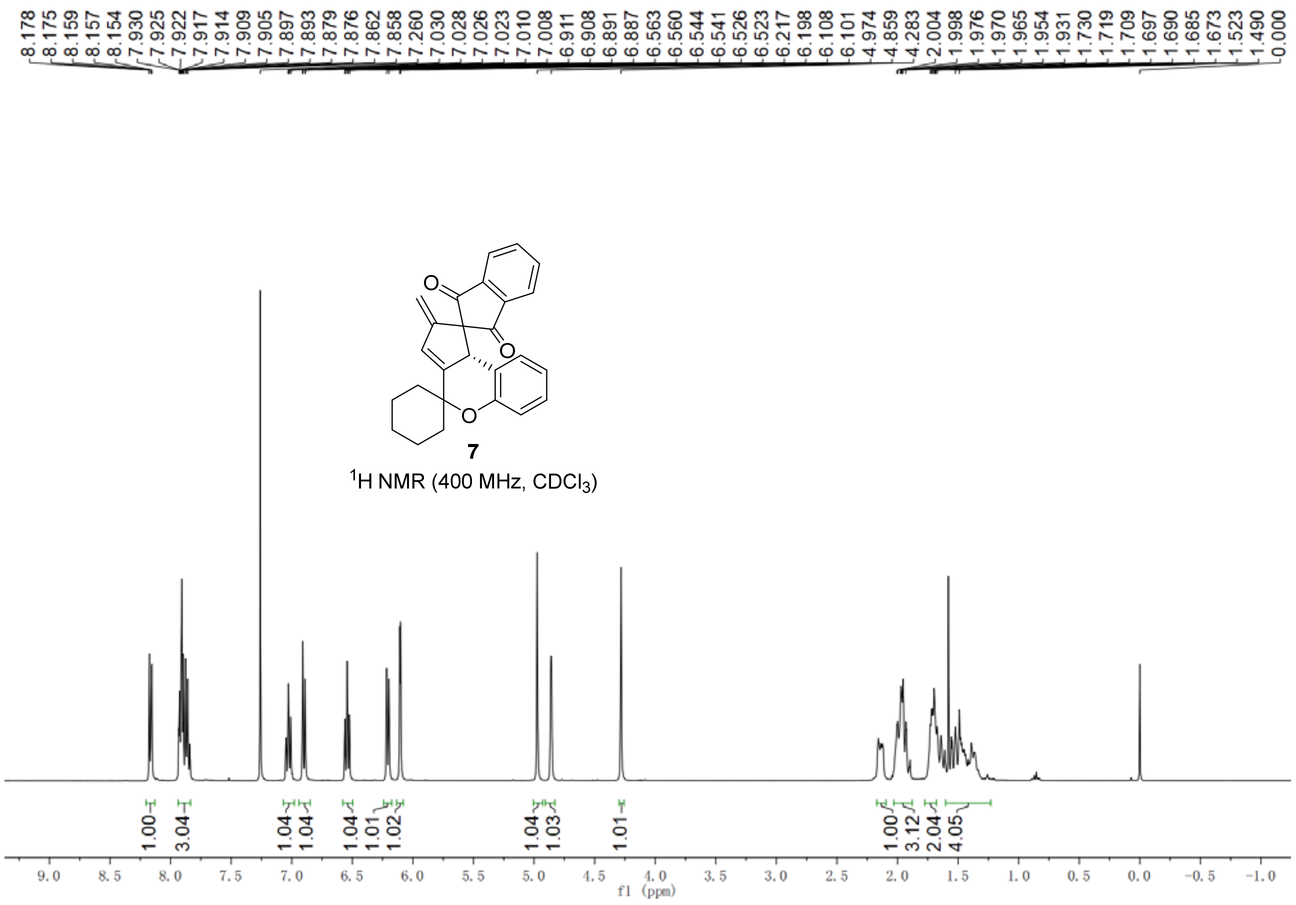
8.178
8.175
8.159
8.157
8.154
7.930
7.925
7.922
7.917
7.914
7.909
7.905
7.897
7.893
7.879
7.876
7.862
7.858
7.260
7.030
7.028
7.026
7.023
7.010
7.008
6.911
6.908
6.891
6.887
6.563
6.560
6.544
6.541
6.526
6.523
6.217
6.198
6.108
6.101
4.974
4.859
4.283
2.004
1.998
1.976
1.970
1.965
1.954
1.931
1.730
1.719
1.709
1.697
1.690
1.685
1.673
1.523
1.490
0.000
O
O
O
7
^{1}H NMR (400 MHz, CDCl$_3$)
1.00
3.04
1.04
1.04
1.04
1.01
1.02
1.04
1.03
1.01
1.00
3.12
2.04
4.05
9.0 8.5 8.0 7.5 7.0 6.5 6.0 5.5 5.0 4.5 4.0 3.5 3.0 2.5 2.0 1.5 1.0 0.5 0.0 −0.5 −1.0
f1 (ppm)

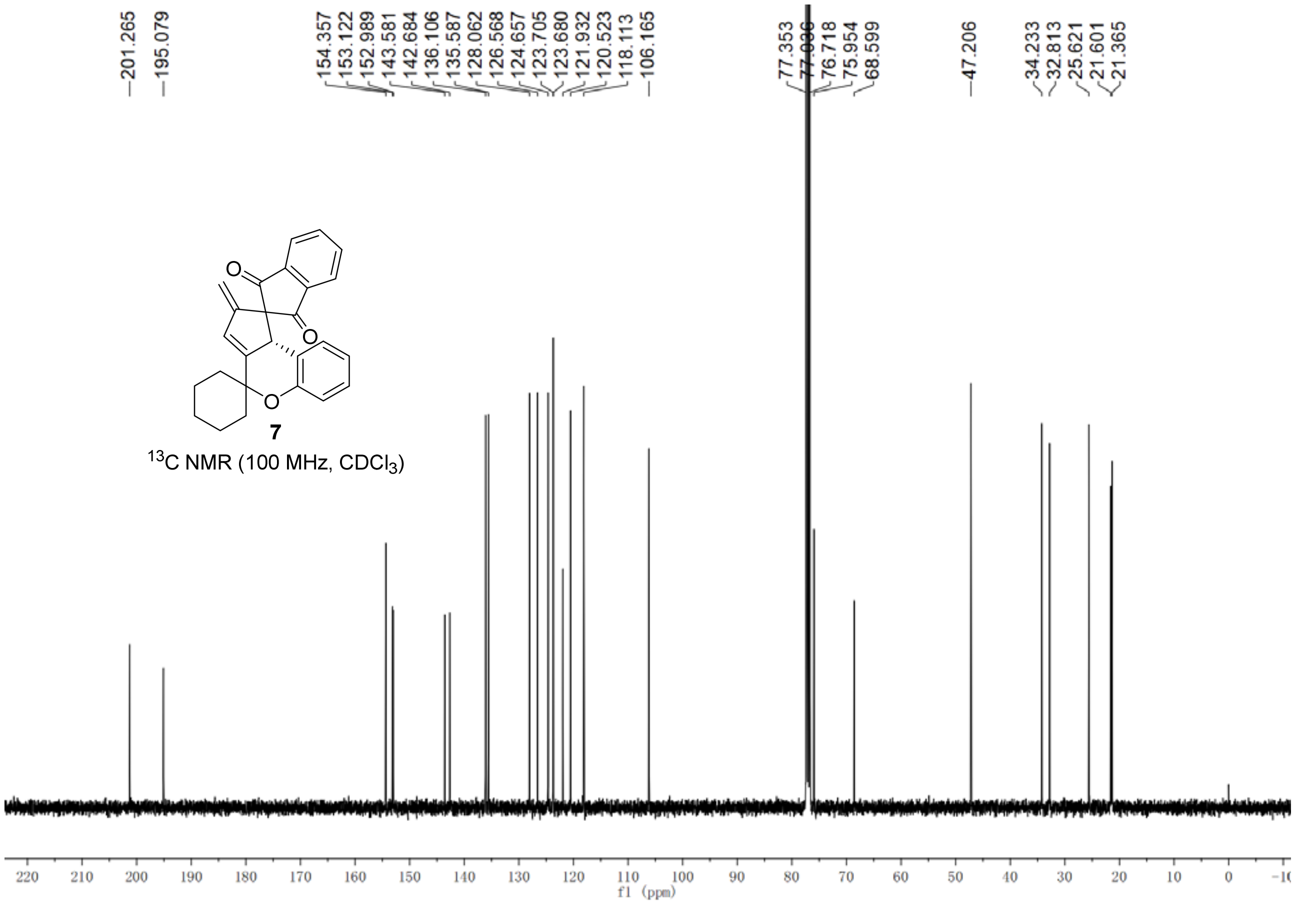
201.265
195.079
154.357
153.122
152.989
143.581
142.684
136.106
135.587
128.062
126.568
124.657
123.705
123.680
121.932
120.523
118.113
106.165
77.353
77.036
76.718
75.954
68.599
47.206
34.233
32.813
25.621
21.601
21.365
O
O
O
7
^{13}C NMR (100 MHz, CDCl$_3$)
220 210 200 190 180 170 160 150 140 130 120 110 100 90 80 70 60 50 40 30 20 10 0 −10
f1 (ppm)

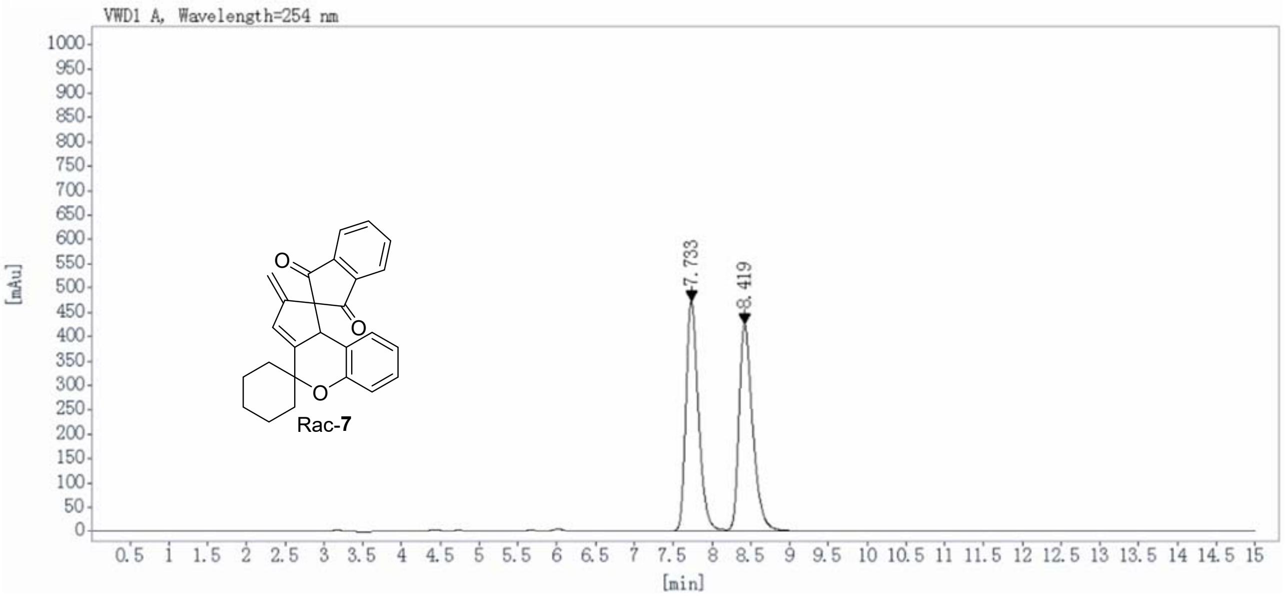

Ret Time [min]	Peak Type	Width [min]	Height [mAU]	Area [mAU*s]	Area [%]
7.733	BV	0.17	471.3006	5264.4287	50.0551
8.419	VBA	0.19	425.1812	5252.8433	49.9449
			Totals:	10517.2720	100.0000

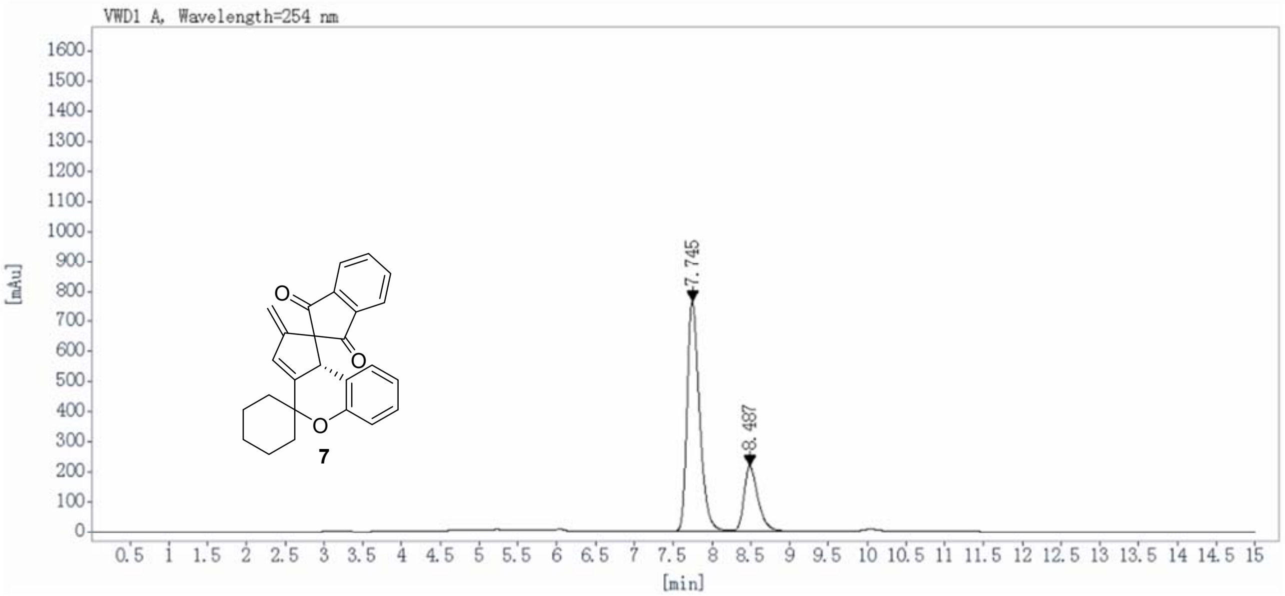

Ret Time [min]	Peak Type	Width [min]	Height [mAU]	Area [mAU*s]	Area [%]
7.745	BV	0.17	764.2188	8462.1875	75.5785
8.487	VBA	0.19	217.0580	2734.3687	24.4215
			Totals:	11196.5562	100.0000

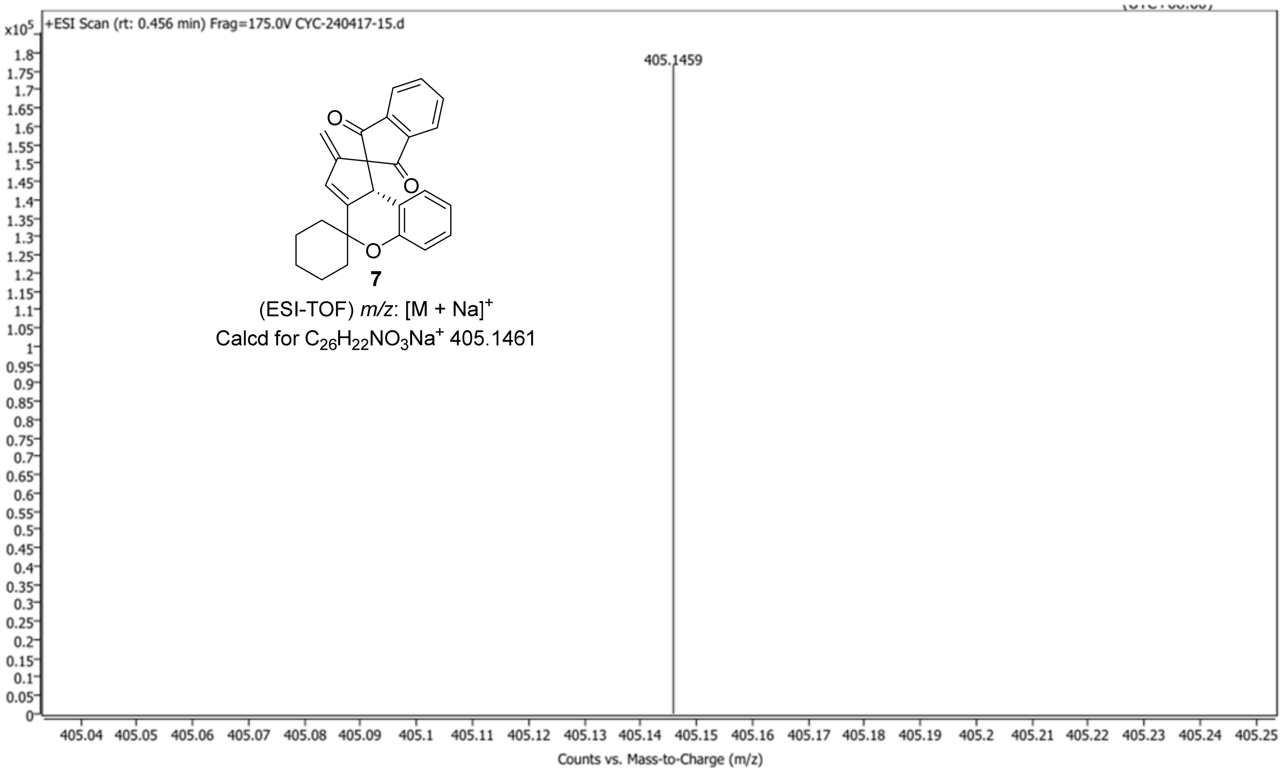

+ESI Scan (rt: 0.456 min) Frag=175.0V CYC-240417-15.d
x10^5
405.1459
7
(ESI-TOF) m/z: [M + Na]+
Calcd for C26H22NO3Na+ 405.1461
Counts vs. Mass-to-Charge (m/z)

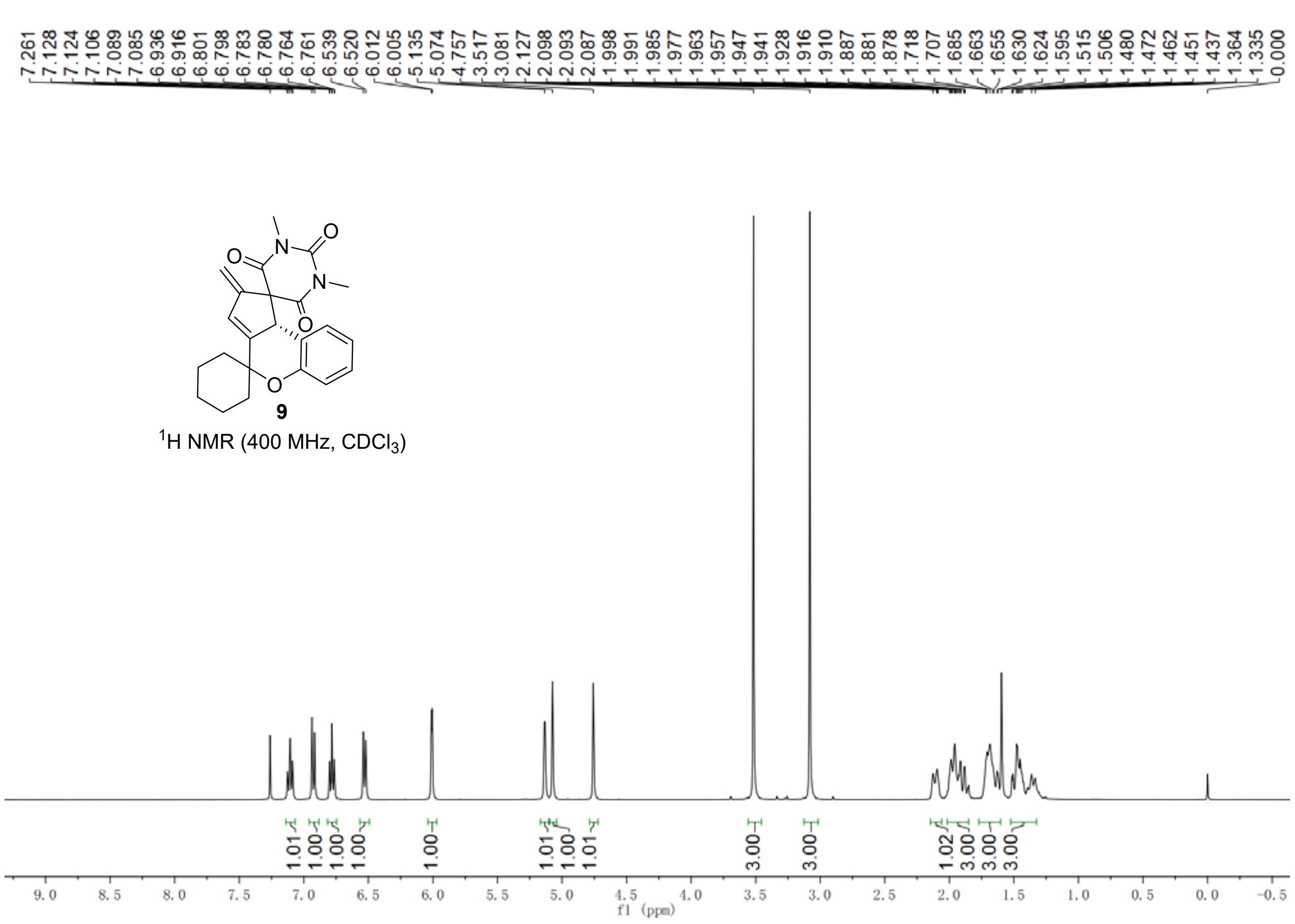
9
1H NMR (400 MHz, CDCl3)
f1 (ppm)

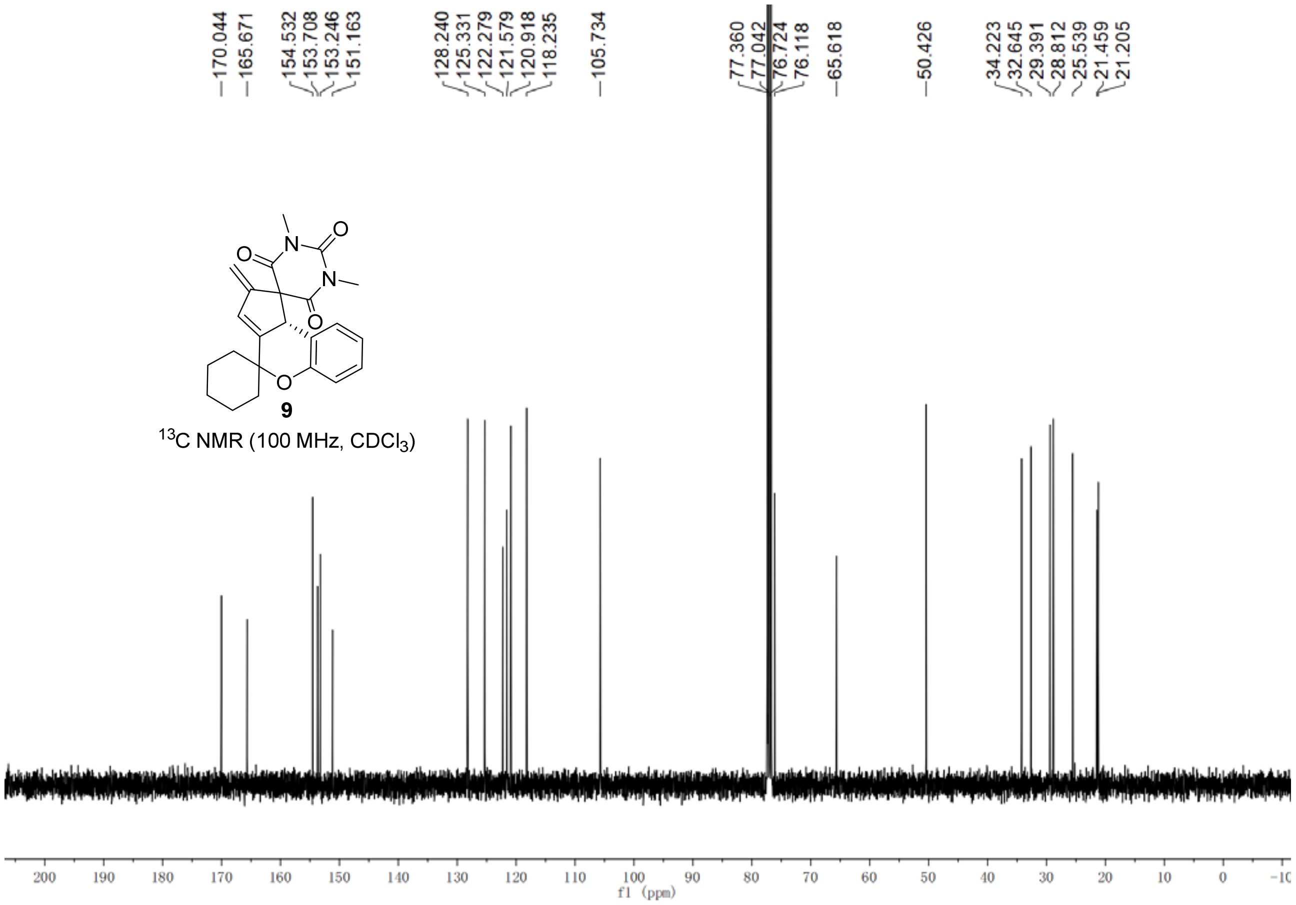
9
13C NMR (100 MHz, CDCl3)
f1 (ppm)

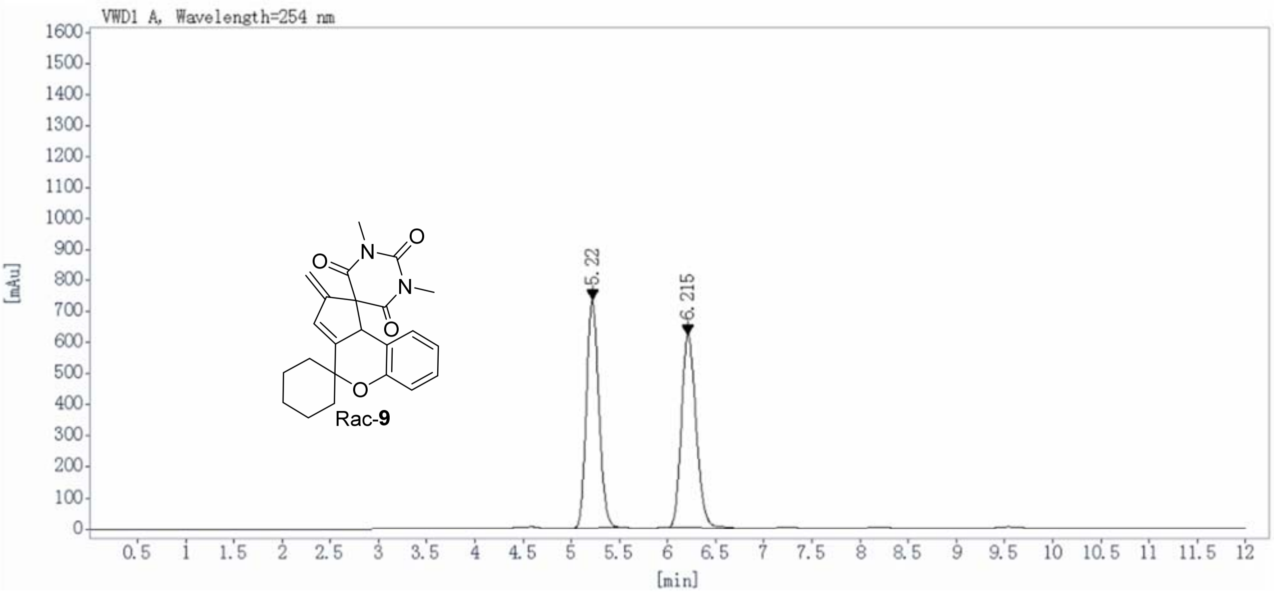

Ret Time [min]	Peak Type	Width [min]	Height [mAU]	Area [mAU*s]	Area [%]
5.220	BBA	0.14	733.8380	6613.3145	49.8825
6.215	BBA	0.17	622.2565	6644.4639	50.1175
			Totals:	13257.7783	100.0000

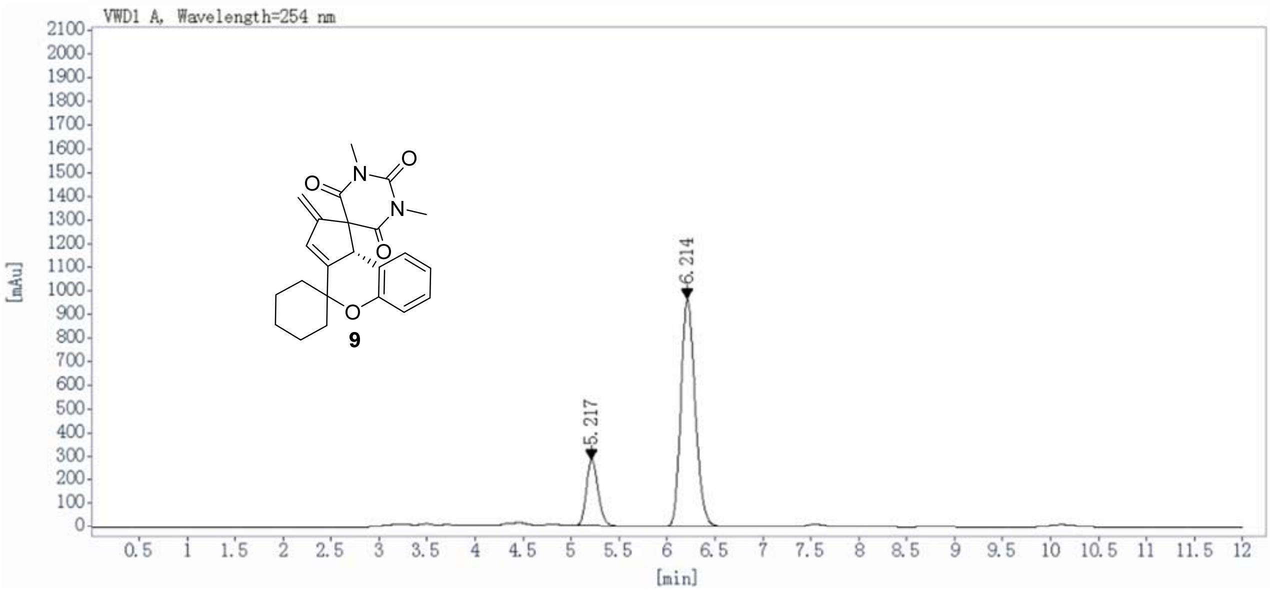

Ret Time [min]	Peak Type	Width [min]	Height [mAU]	Area [mAU*s]	Area [%]
5.217	BBA	0.13	283.1979	2408.3604	19.3641
6.214	BBA	0.16	961.2004	10028.8535	80.6359
			Totals:	12437.2139	100.0000

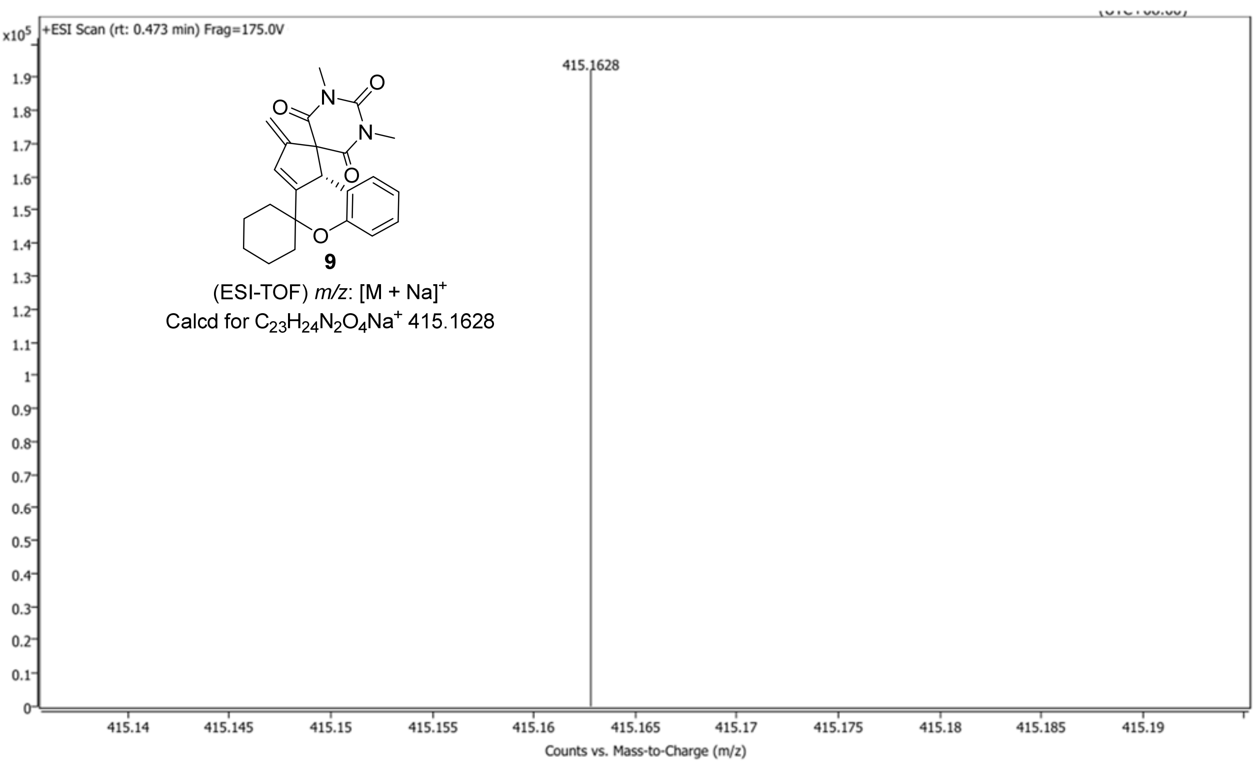
+ESI Scan (rt: 0.473 min) Frag=175.0V
x10^5
415.1628
9
(ESI-TOF) m/z: [M + Na]+
Calcd for C23H24N2O4Na+ 415.1628
Counts vs. Mass-to-Charge (m/z)

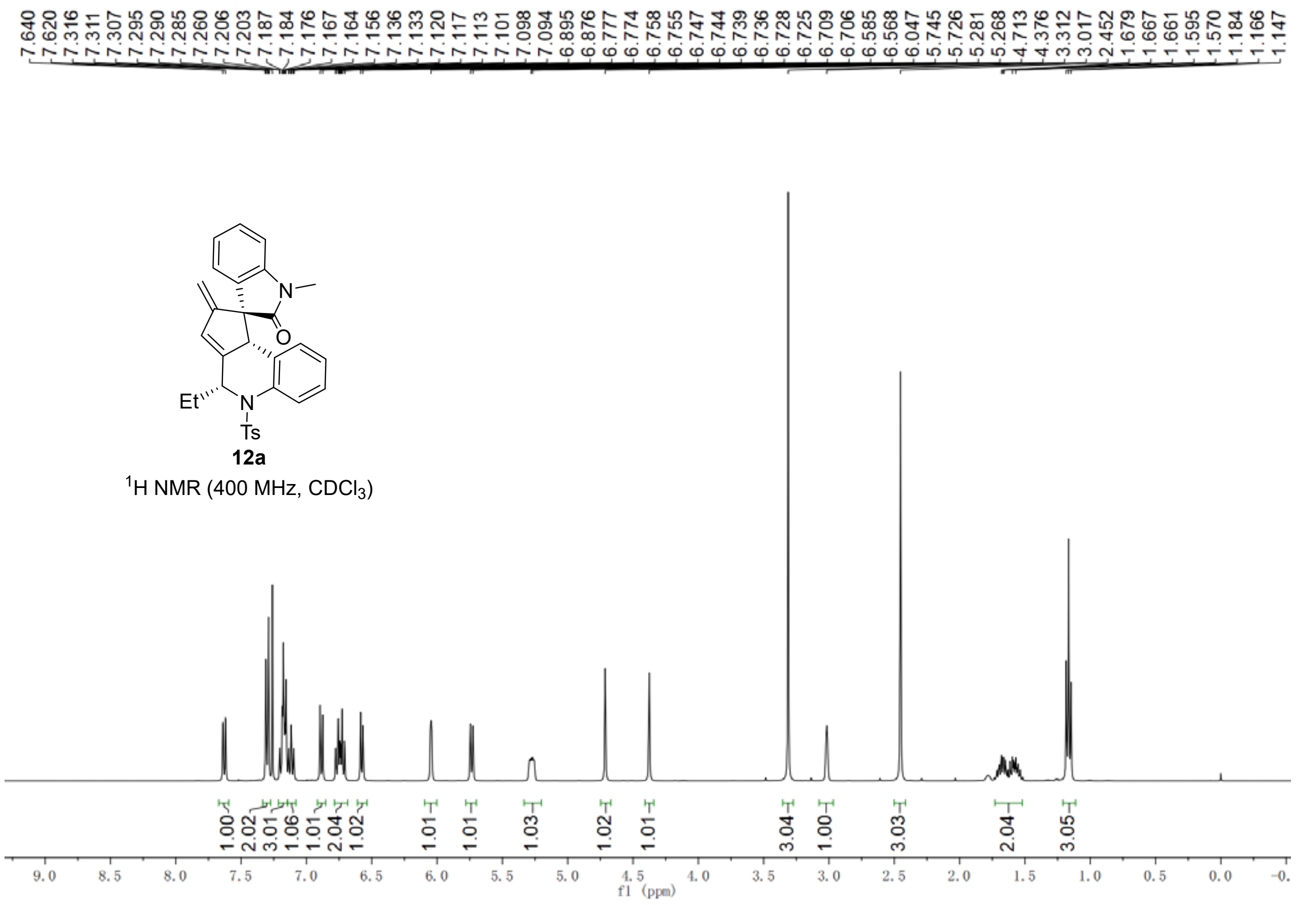
7.640
7.620
7.316
7.311
7.307
7.295
7.290
7.285
7.260
7.206
7.203
7.187
7.184
7.176
7.167
7.164
7.156
7.136
7.133
7.120
7.117
7.113
7.101
7.098
7.094
6.895
6.876
6.777
6.774
6.758
6.755
6.747
6.744
6.739
6.736
6.728
6.725
6.709
6.706
6.585
6.568
6.047
5.745
5.726
5.281
5.268
4.713
4.376
3.312
3.017
2.452
1.679
1.667
1.661
1.595
1.570
1.184
1.166
1.147
Et
N
Ts
O
12a
^{1}H NMR (400 MHz, $CDCl_3$)
1.00
2.02
3.01
1.06
1.01
2.04
1.02
1.01
1.01
1.03
1.02
1.01
3.04
1.00
3.03
2.04
3.05
f1 (ppm)

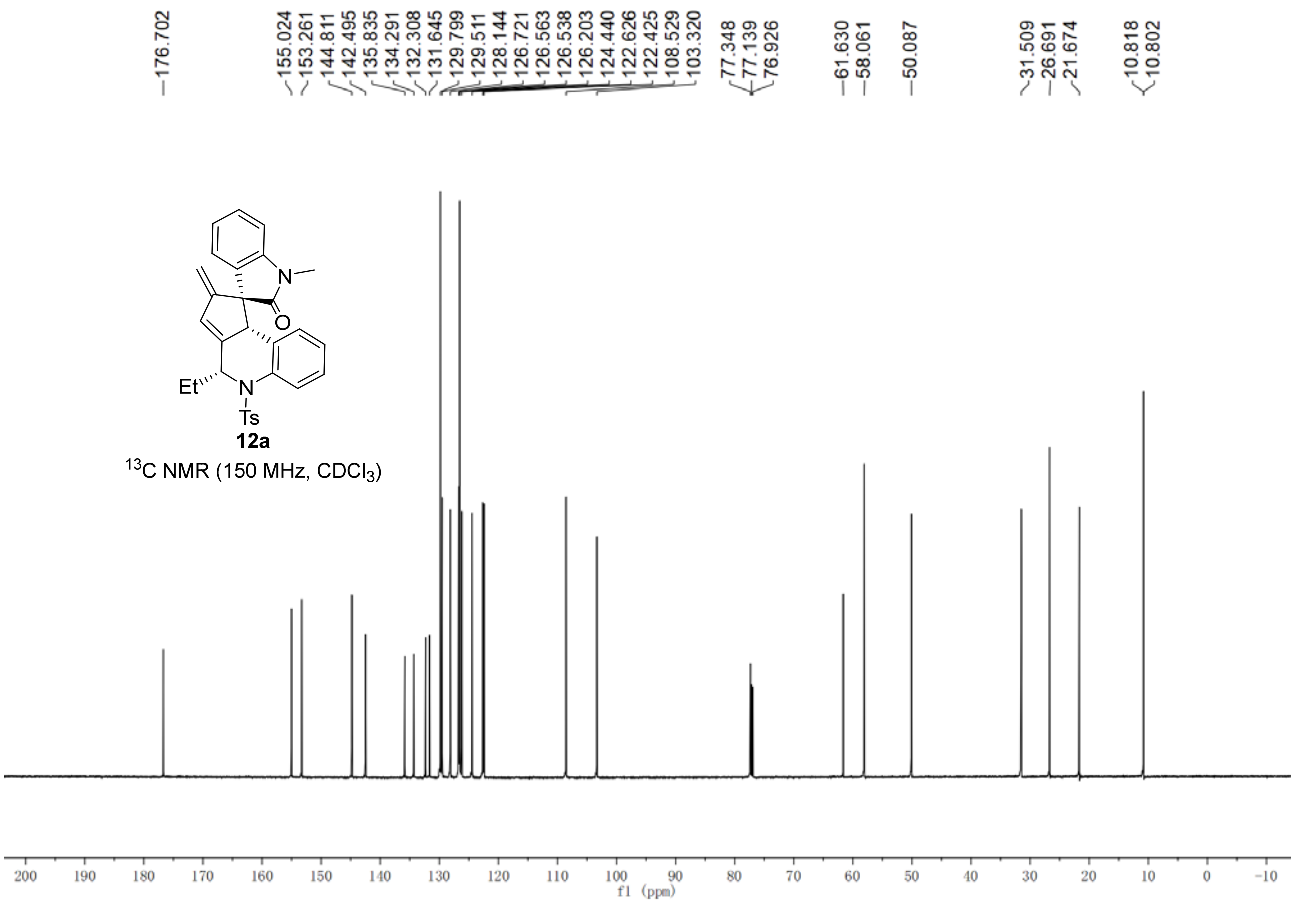
176.702
155.024
153.261
144.811
142.495
135.835
134.291
132.308
131.645
129.799
129.511
128.144
126.721
126.563
126.538
126.203
124.440
122.626
122.425
108.529
103.320
77.348
77.139
76.926
61.630
58.061
50.087
31.509
26.691
21.674
10.818
10.802
Et
N
Ts
O
12a
^{13}C NMR (150 MHz, $CDCl_3$)
f1 (ppm)

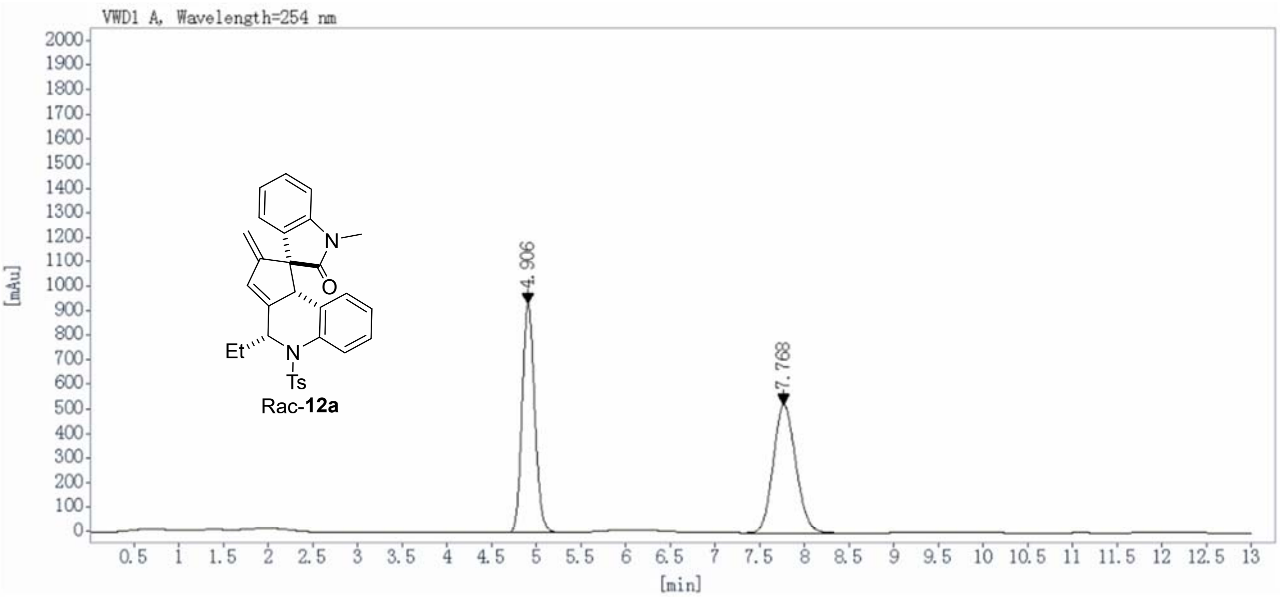

Ret Time [min]	Peak Type	Width [min]	Height [mAU]	Area [mAU*s]	Area [%]
4.906	BBA	0.15	935.4191	9221.7646	49.9052
7.768	BBA	0.28	524.1456	9256.7979	50.0948
			Totals:	18478.5625	100.0000

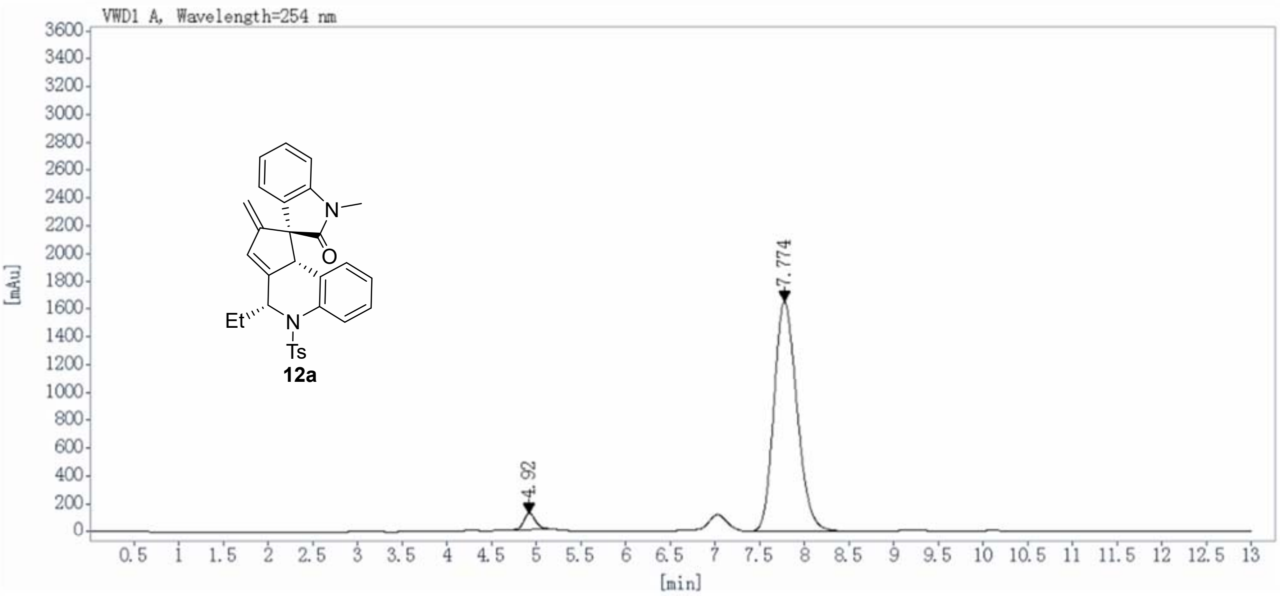

Ret Time [min]	Peak Type	Width [min]	Height [mAU]	Area [mAU*s]	Area [%]
4.920	BBA	0.14	117.5743	1106.3406	3.5979
7.774	BBA	0.28	1648.6512	29643.4590	96.4021
			Totals:	30749.7996	100.0000

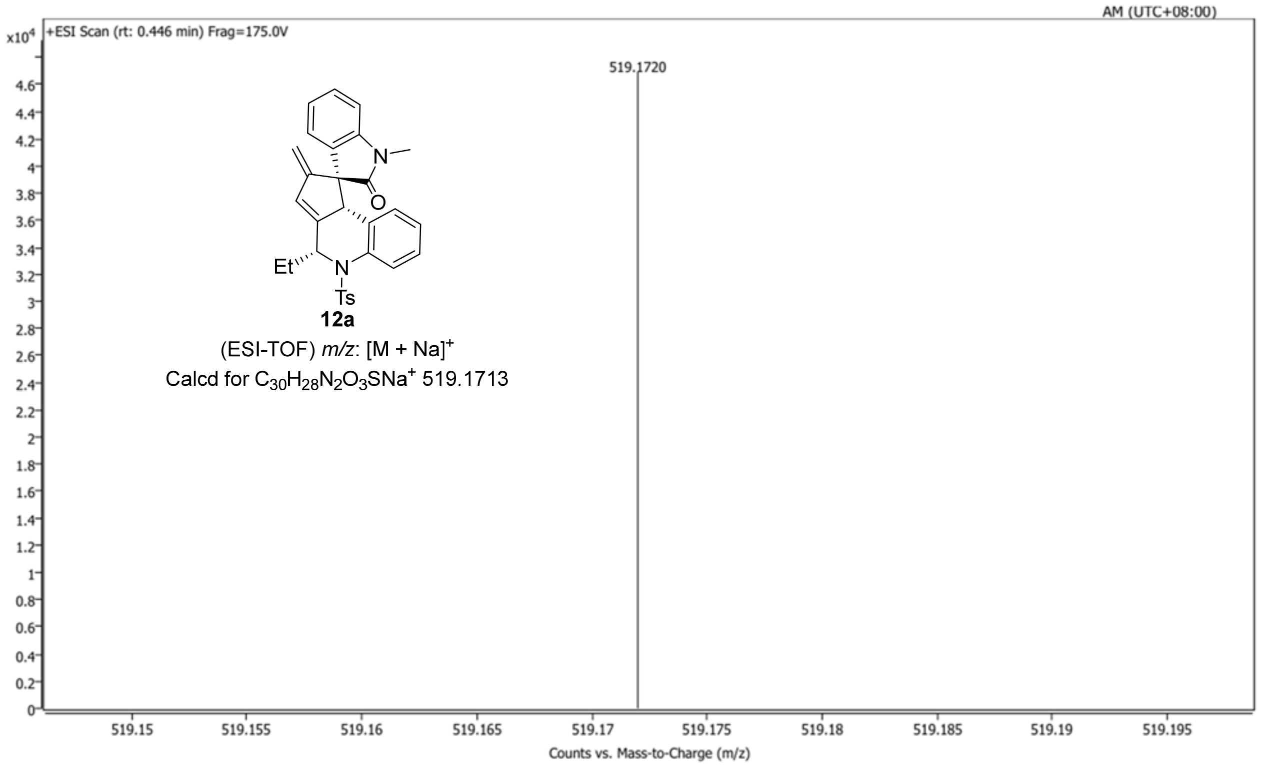
AM (UTC+08:00)
x10^4 +ESI Scan (rt: 0.446 min) Frag=175.0V
519.1720
Et
N
Ts
O
12a
(ESI-TOF) m/z: [M + Na]+
Calcd for C30H28N2O3SNa+ 519.1713
Counts vs. Mass-to-Charge (m/z)

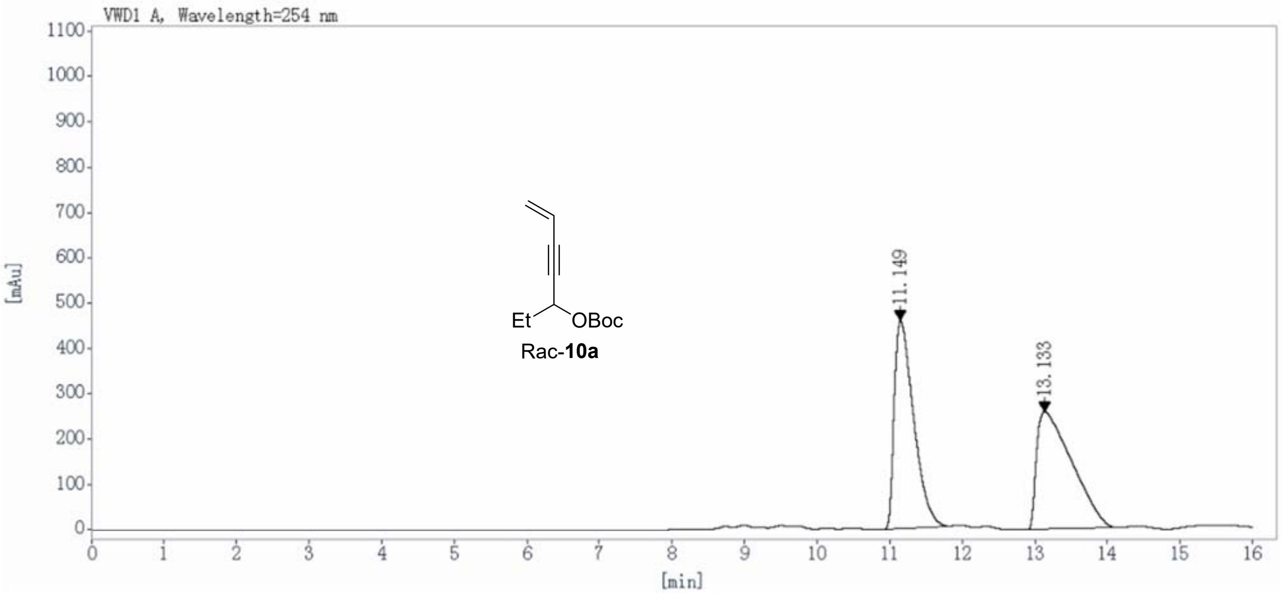

Ret Time [min]	Peak Type	Width [min]	Height [mAU]	Area [mAU*s]	Area [%]
11.149	BB	0.28	458.2355	8487.8613	49.3090
13.133	BB	0.51	258.5074	8725.7383	50.6910
			Totals:	17213.5996	100.0000

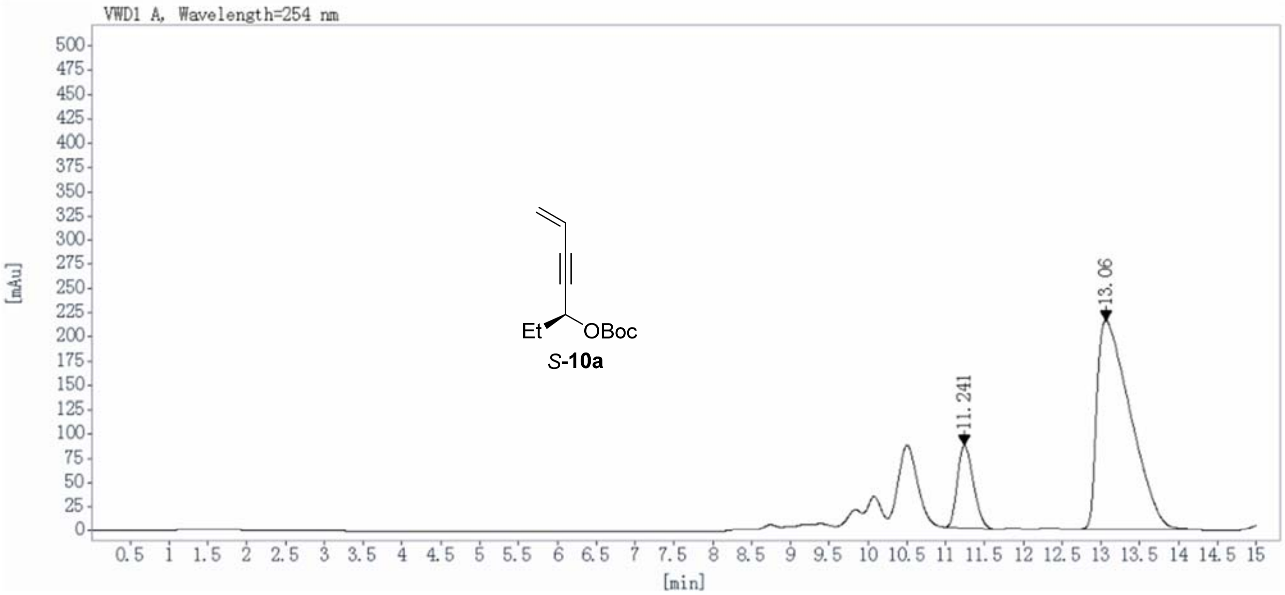

Ret Time [min]	Peak Type	Width [min]	Height [mAU]	Area [mAU*s]	Area [%]
11.241	BB	0.23	85.3246	1229.3444	15.9744
13.060	BBA	0.47	215.0339	6466.3936	84.0256
			Totals:	7695.7379	100.0000

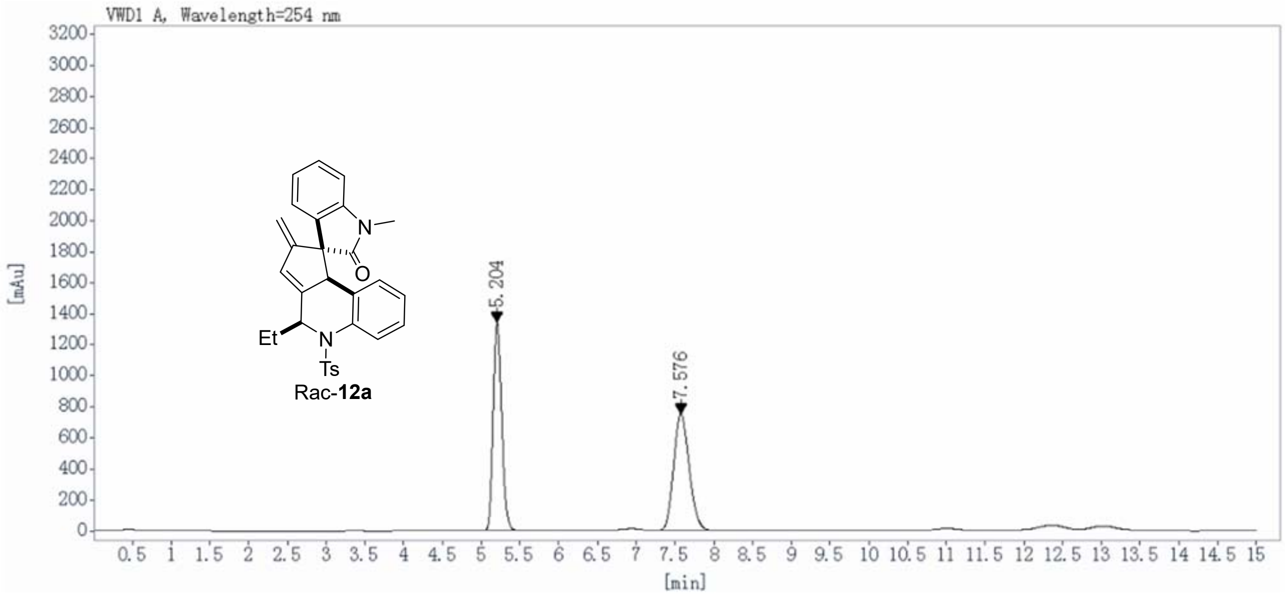

Ret Time [min]	Peak Type	Width [min]	Height [mAU]	Area [mAU*s]	Area [%]
5.204	BBA	0.12	1345.2666	10414.7266	49.8143
7.576	BBA	0.22	752.3749	10492.3945	50.1857
			Totals:	20907.1211	100.0000

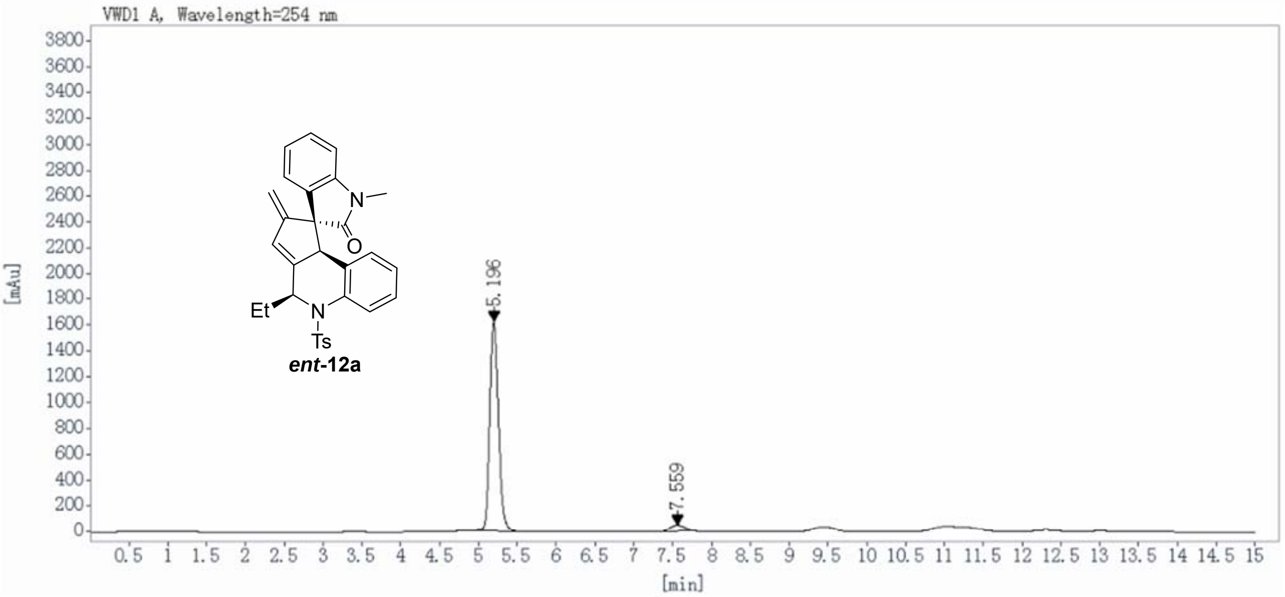

Ret Time [min]	Peak Type	Width [min]	Height [mAU]	Area [mAU*s]	Area [%]
5.196	BBA	0.12	1618.7483	12679.8691	95.4738
7.559	BBA	0.20	46.7730	601.1260	4.5262
			Totals:	13280.9952	100.0000

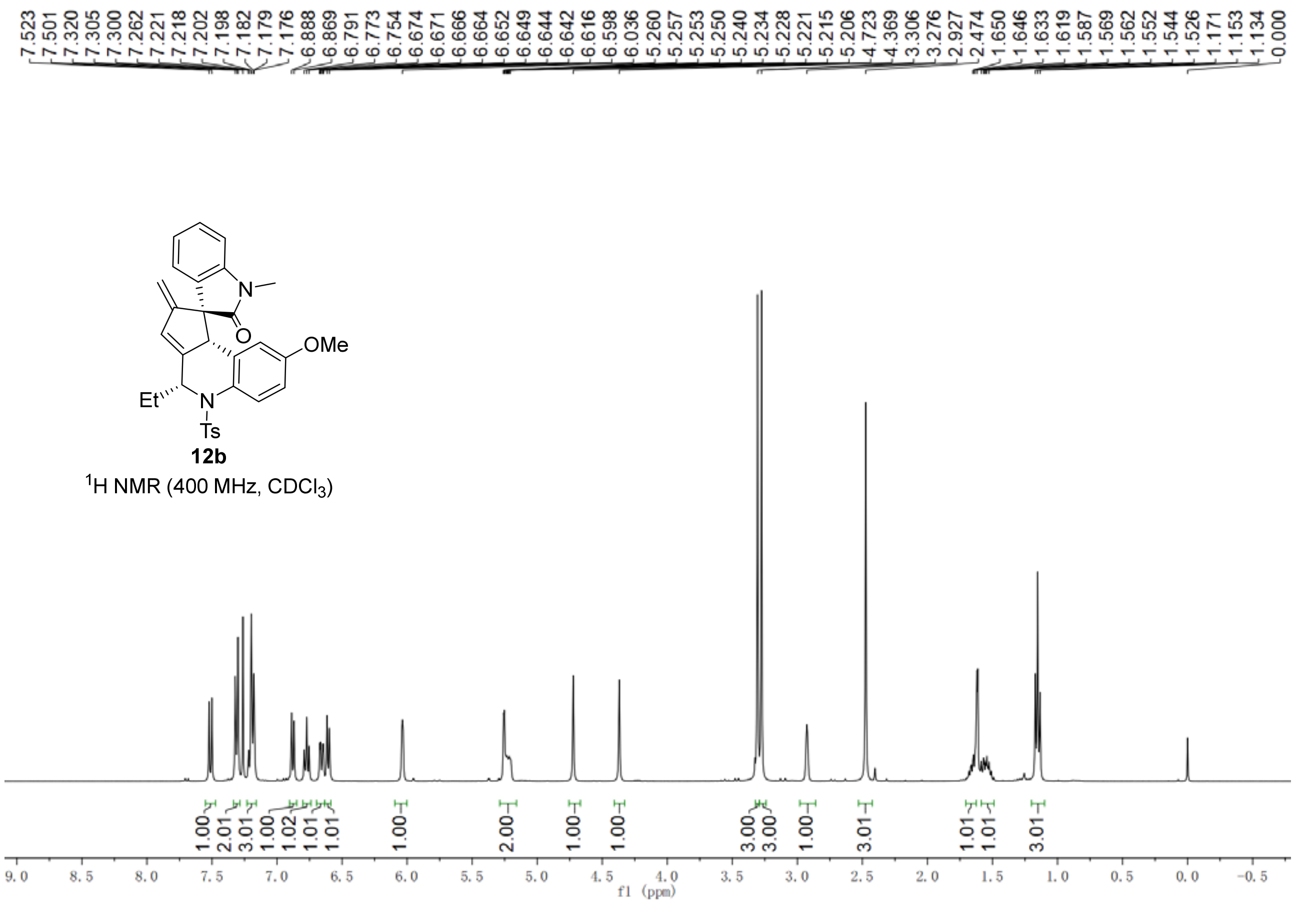
7.523
7.501
7.320
7.305
7.300
7.262
7.221
7.218
7.202
7.198
7.182
7.179
7.176
6.888
6.869
6.791
6.773
6.754
6.674
6.671
6.666
6.664
6.652
6.649
6.644
6.642
6.616
6.598
6.036
5.260
5.257
5.253
5.250
5.240
5.234
5.228
5.221
5.215
5.206
4.723
4.369
3.306
3.276
2.927
2.474
1.650
1.646
1.633
1.619
1.587
1.569
1.562
1.552
1.544
1.526
1.171
1.153
1.134
0.000
OMe
N
O
Et
N
Ts
12b
^{1}H NMR (400 MHz, $CDCl_3$)
1.00
2.01
3.01
1.00
1.02
1.01
1.01
1.00
2.00
1.00
1.00
3.00
3.00
1.00
3.01
1.01
1.01
3.01
9.0 8.5 8.0 7.5 7.0 6.5 6.0 5.5 5.0 4.5 4.0 3.5 3.0 2.5 2.0 1.5 1.0 0.5 0.0 -0.5
f1 (ppm)

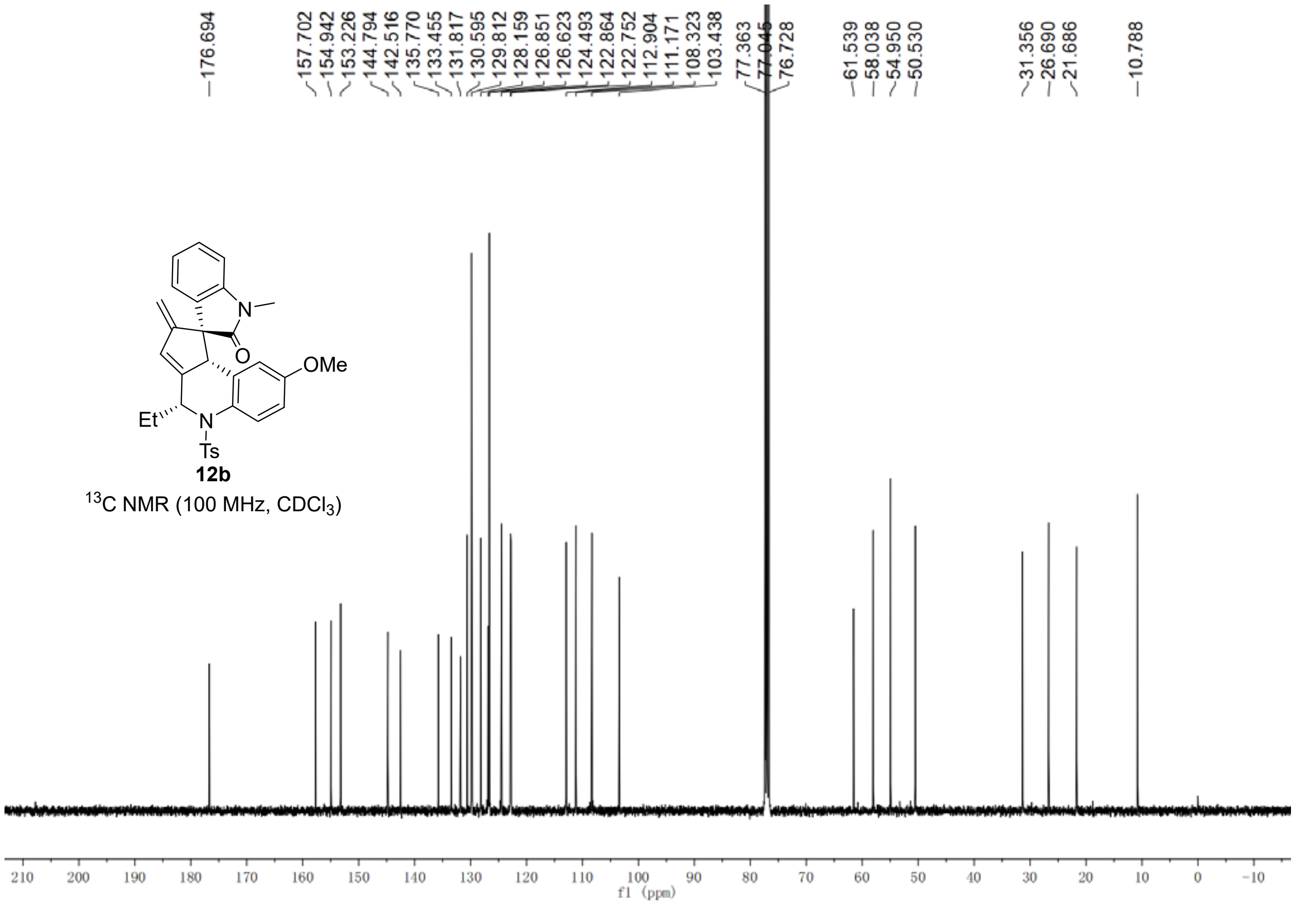
176.694
157.702
154.942
153.226
144.794
142.516
135.770
133.455
131.817
130.595
129.812
128.159
126.851
126.623
124.493
122.864
122.752
112.904
111.171
108.323
103.438
77.363
77.046
76.728
61.539
58.038
54.950
50.530
31.356
26.690
21.686
10.788
OMe
N
O
Et
N
Ts
12b
^{13}C NMR (100 MHz, $CDCl_3$)
210 200 190 180 170 160 150 140 130 120 110 100 90 80 70 60 50 40 30 20 10 0 -10
f1 (ppm)

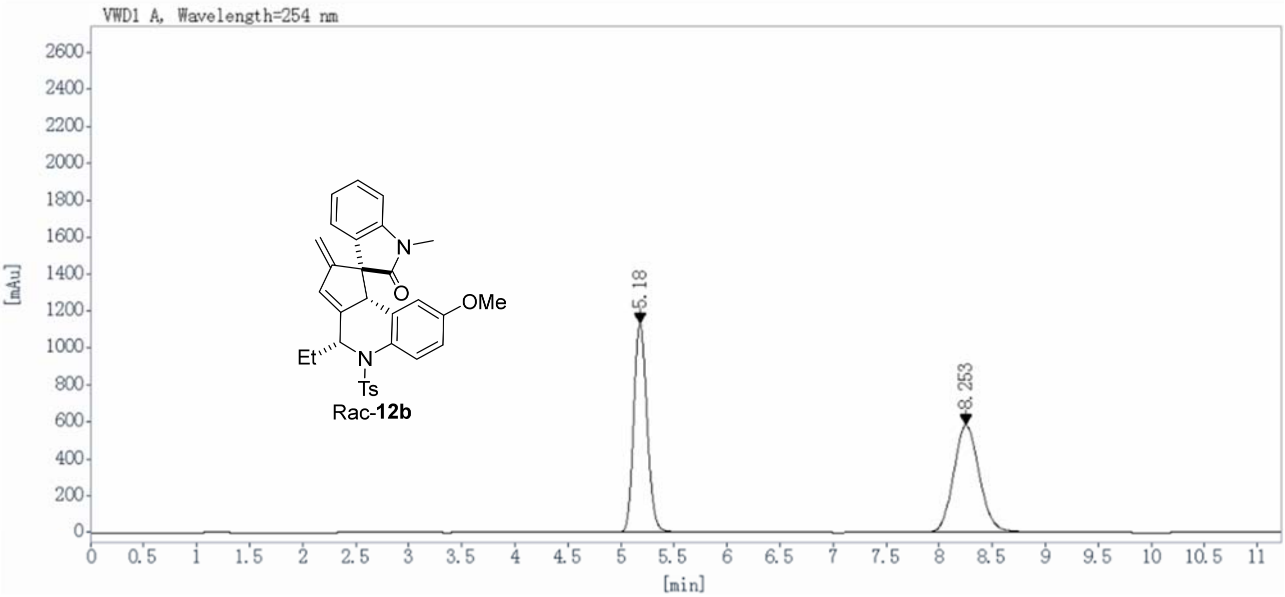

Ret Time [min]	Peak Type	Width [min]	Height [mAU]	Area [mAU*s]	Area [%]
5.180	BBA	0.13	1133.0798	9818.5264	49.8066
8.253	BBA	0.26	580.9495	9894.7607	50.1934
			Totals:	19713.2871	100.0000

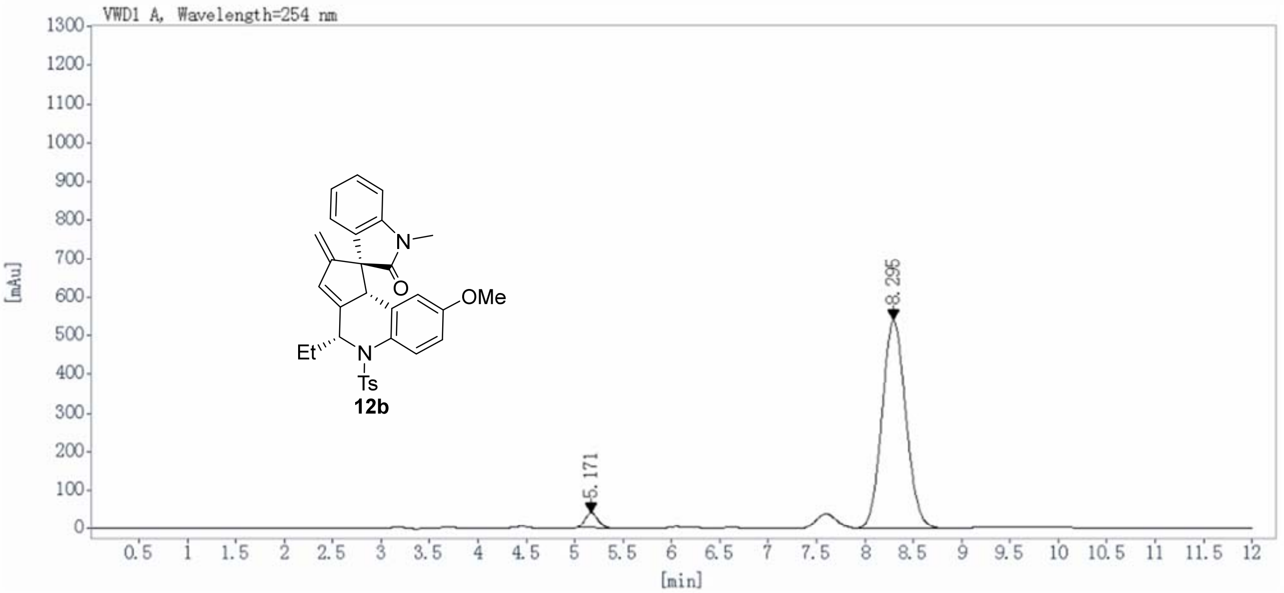

Ret Time [min]	Peak Type	Width [min]	Height [mAU]	Area [mAU*s]	Area [%]
5.171	BBA	0.14	38.6503	352.1087	3.6790
8.295	BBA	0.27	538.2534	9218.6953	96.3210
			Totals:	9570.8040	100.0000

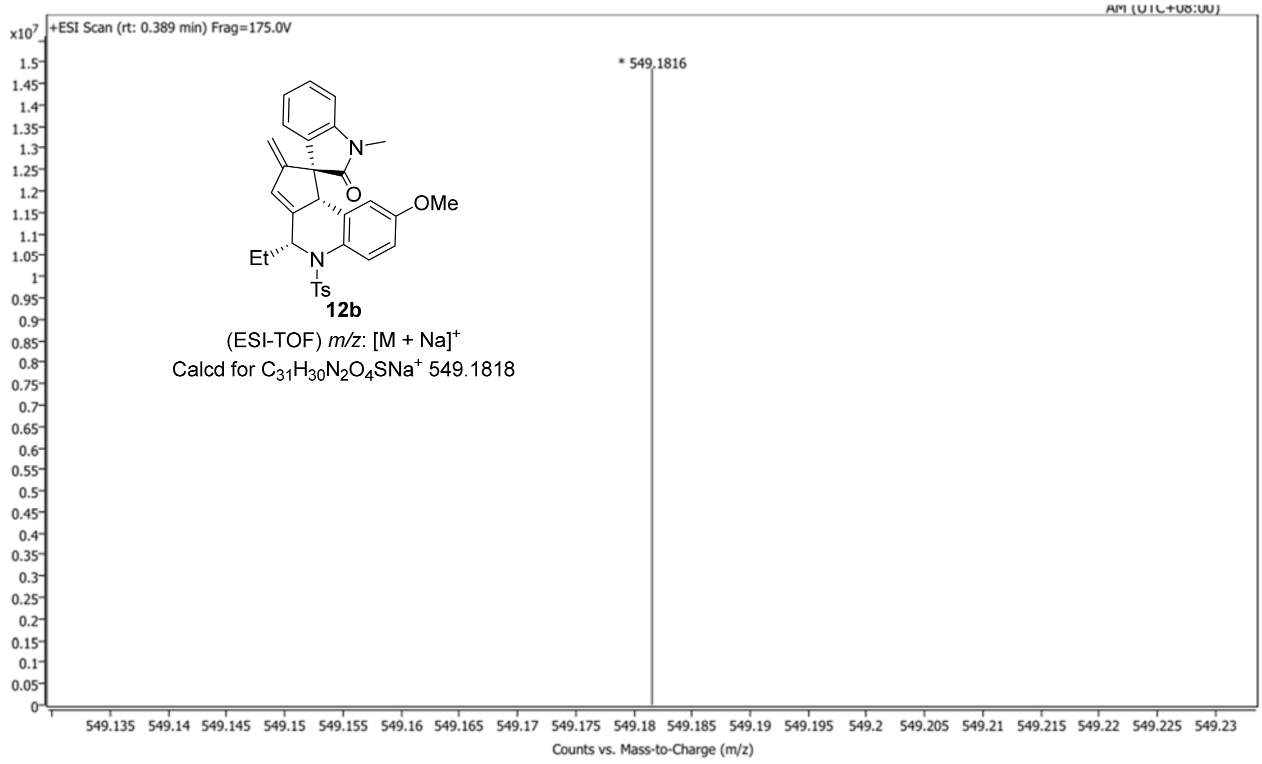

x10^7
+ESI Scan (rt: 0.389 min) Frag=175.0V
* 549.1816
OMe
N
O
Et
Ts
12b
(ESI-TOF) m/z: [M + Na]+
Calcd for C31H30N2O4SNa+ 549.1818
Counts vs. Mass-to-Charge (m/z)

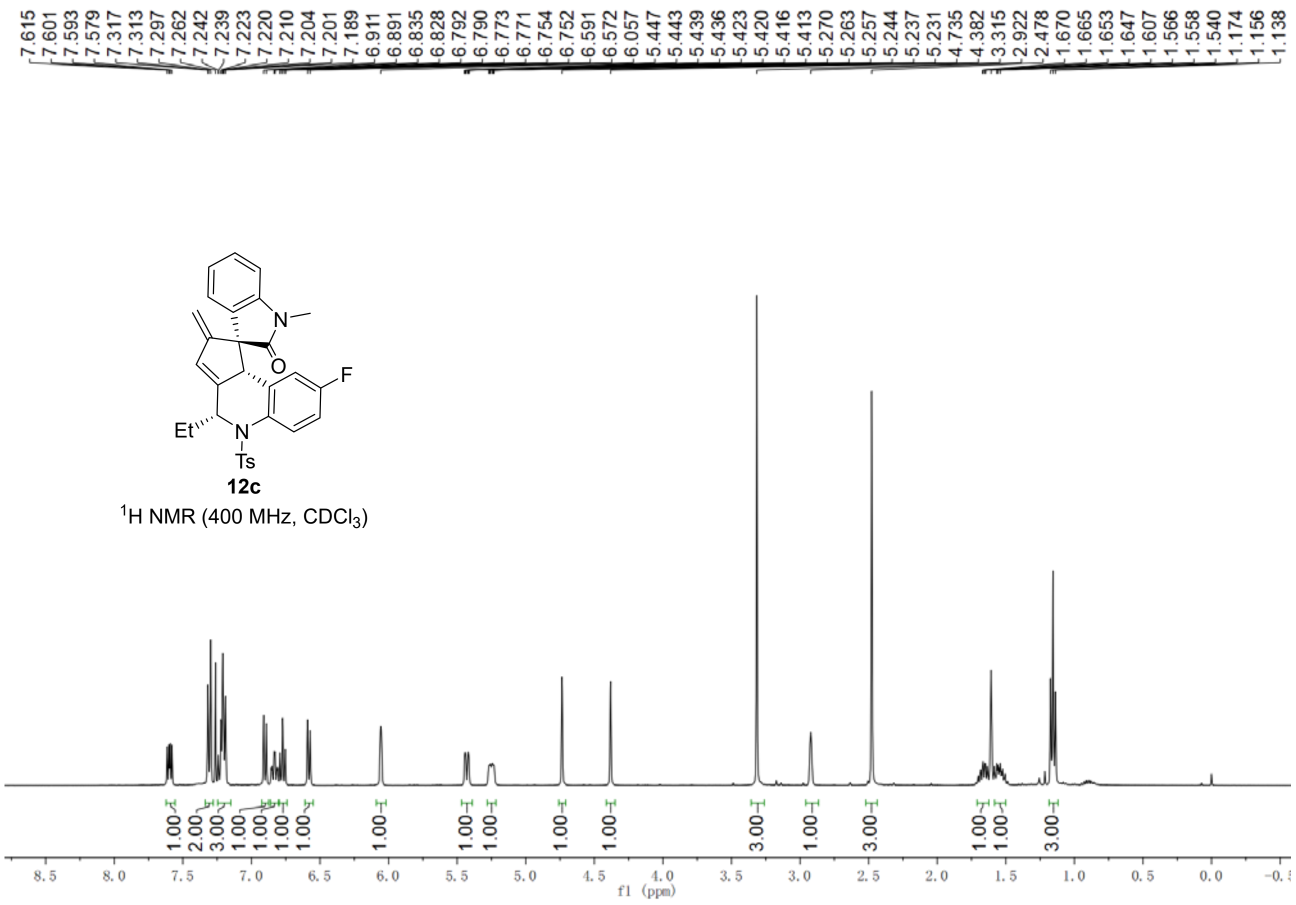

12c

^{1}H NMR (400 MHz, $CDCl_3$)

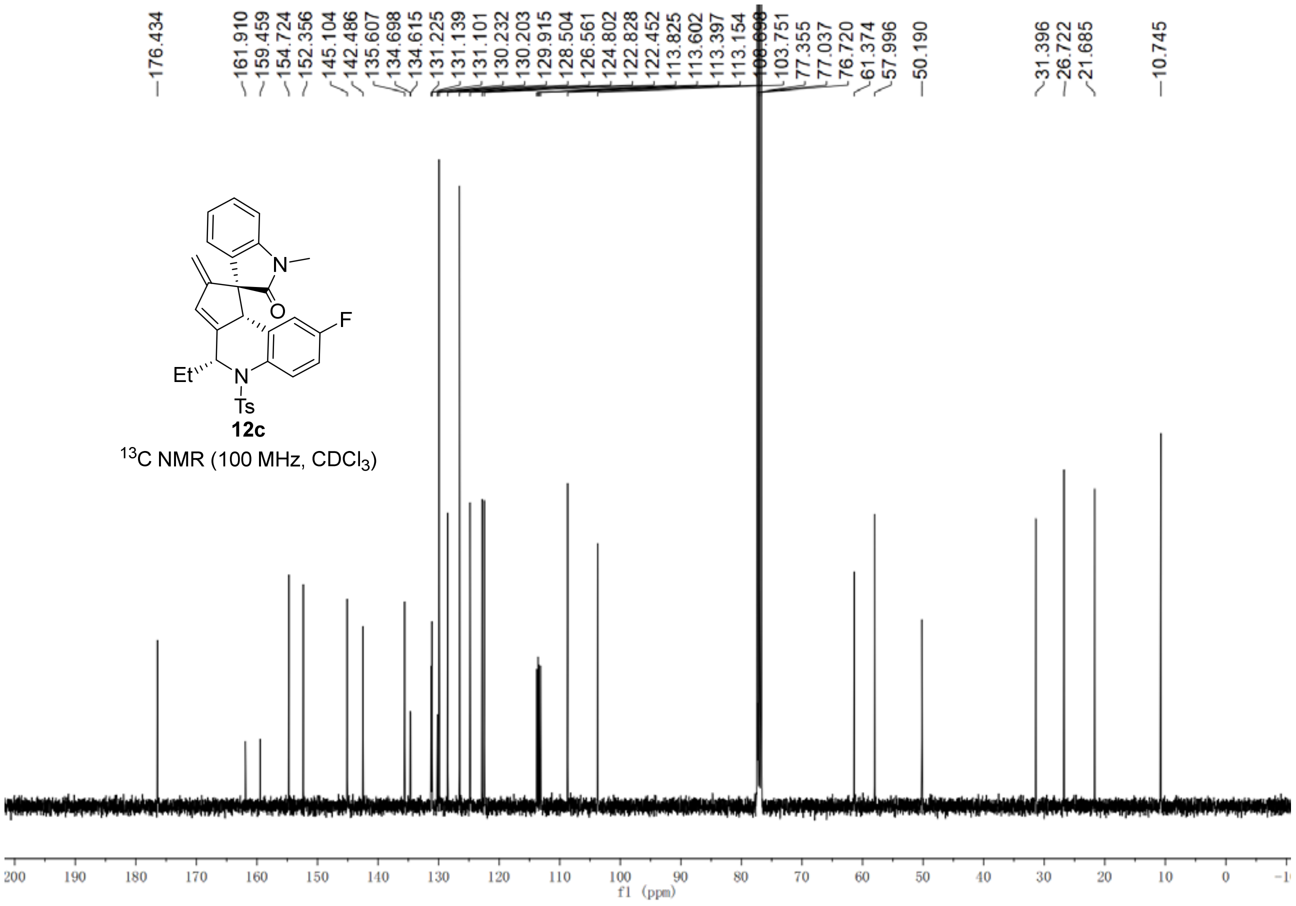

12c

^{13}C NMR (100 MHz, $CDCl_3$)

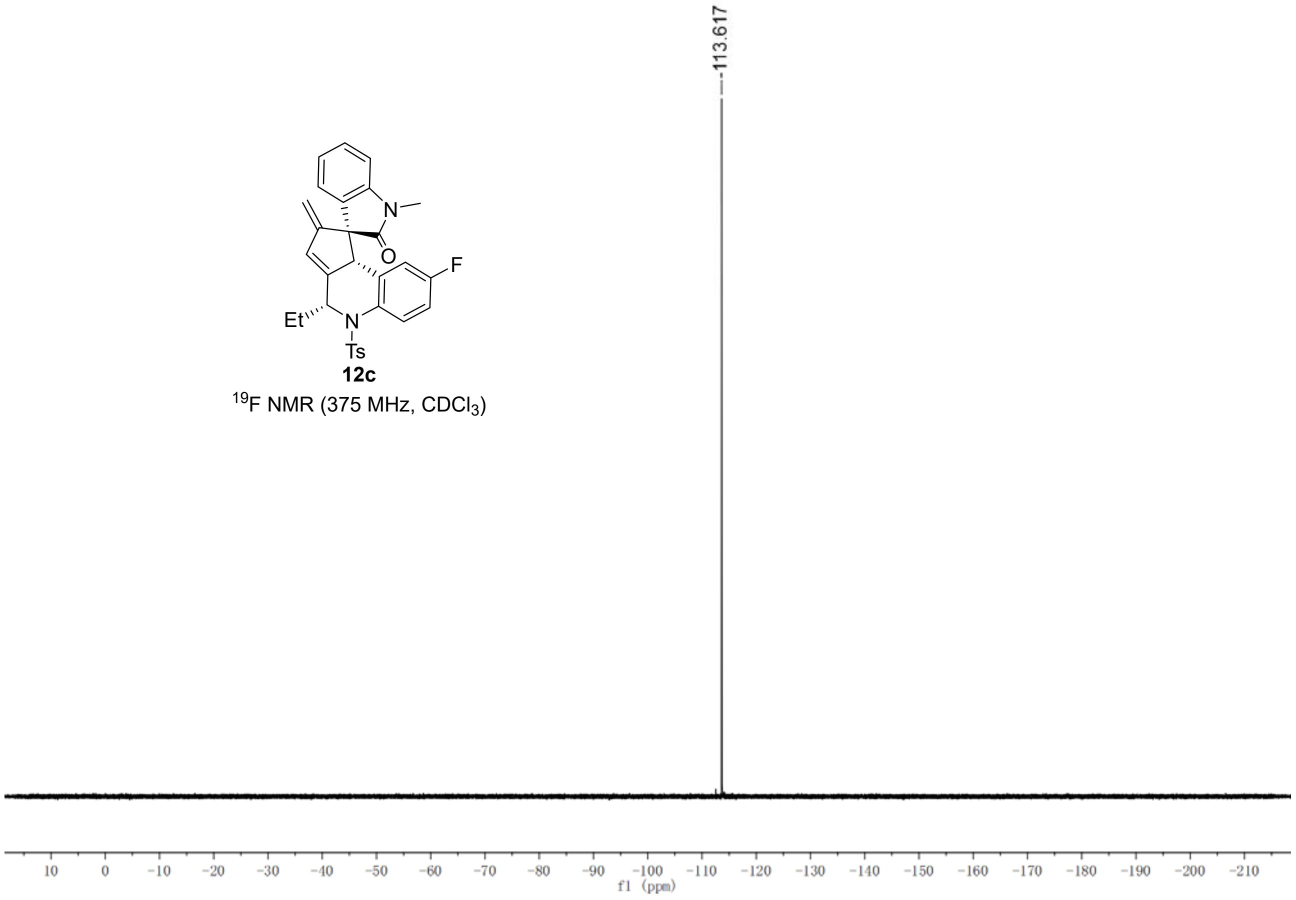

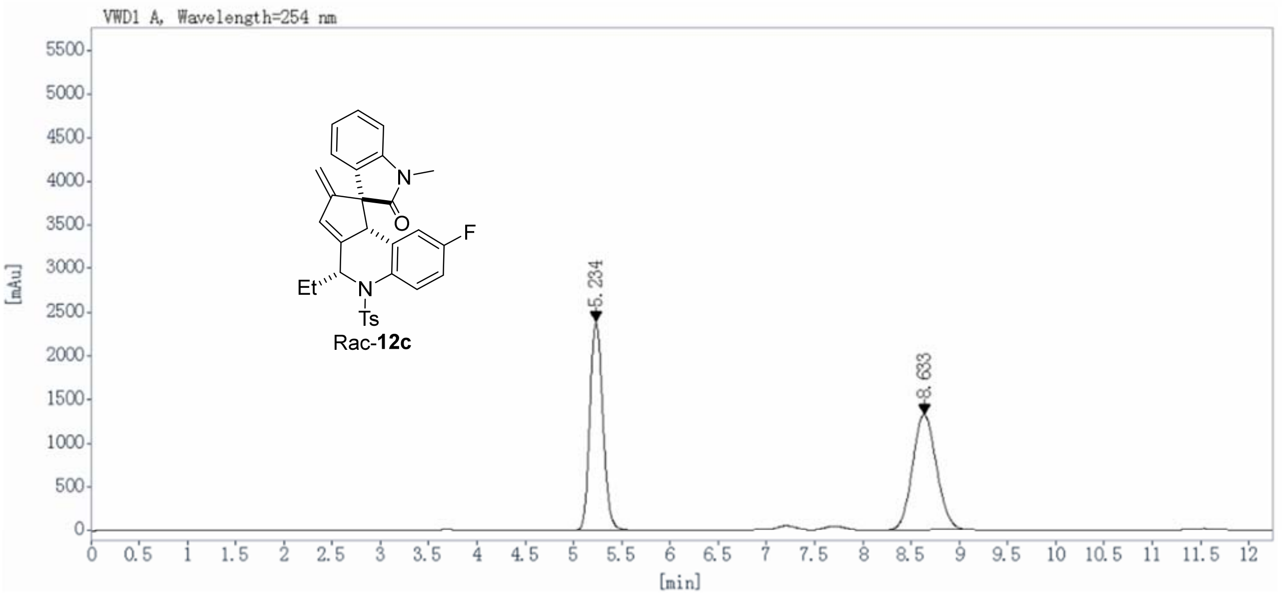

Ret Time [min]	Peak Type	Width [min]	Height [mAU]	Area [mAU*s]	Area [%]
5.234	BBA	0.15	2381.8638	22391.8848	49.2868
8.633	BBA	0.27	1325.9989	23039.9160	50.7132
			Totals:	45431.8008	100.0000

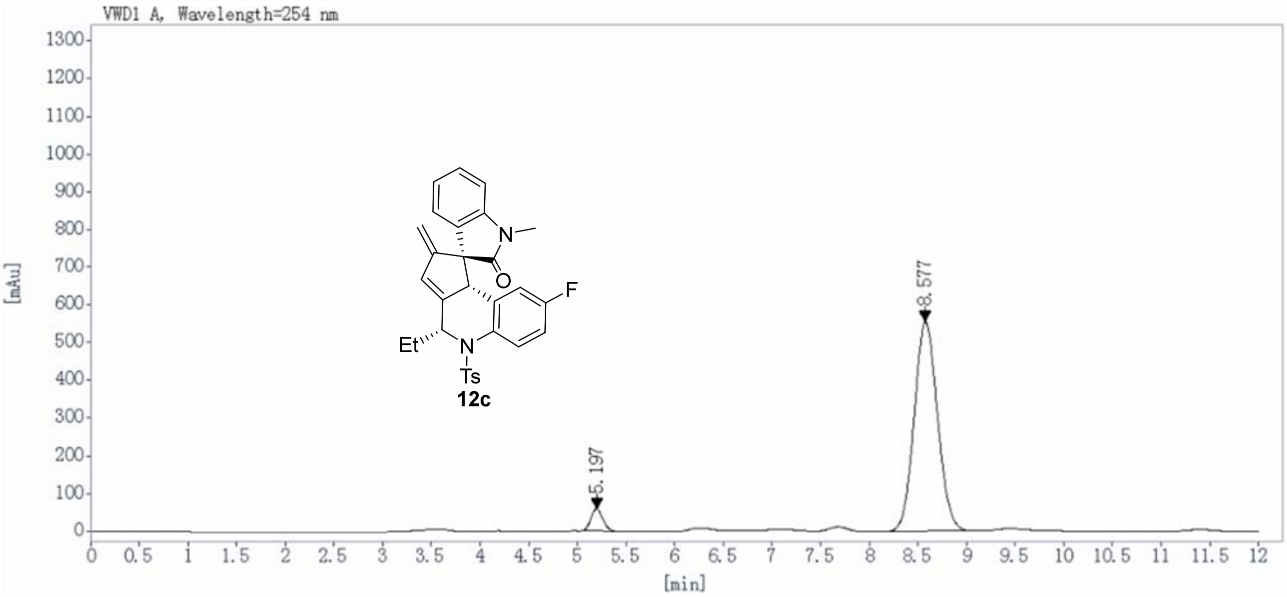

Ret Time [min]	Peak Type	Width [min]	Height [mAU]	Area [mAU*s]	Area [%]
5.197	BBA	0.13	58.9124	498.6888	5.0997
8.577	BB	0.26	555.4326	9280.1836	94.9003
			Totals:	9778.8724	100.0000

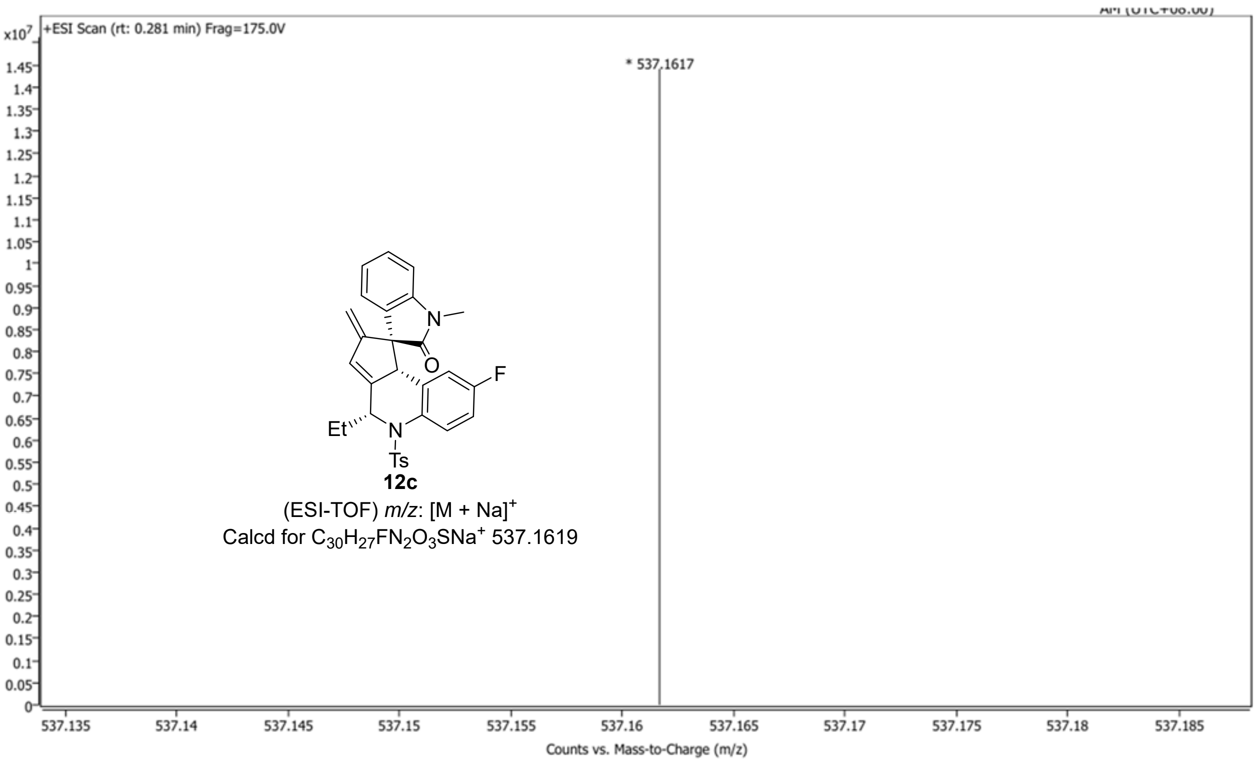

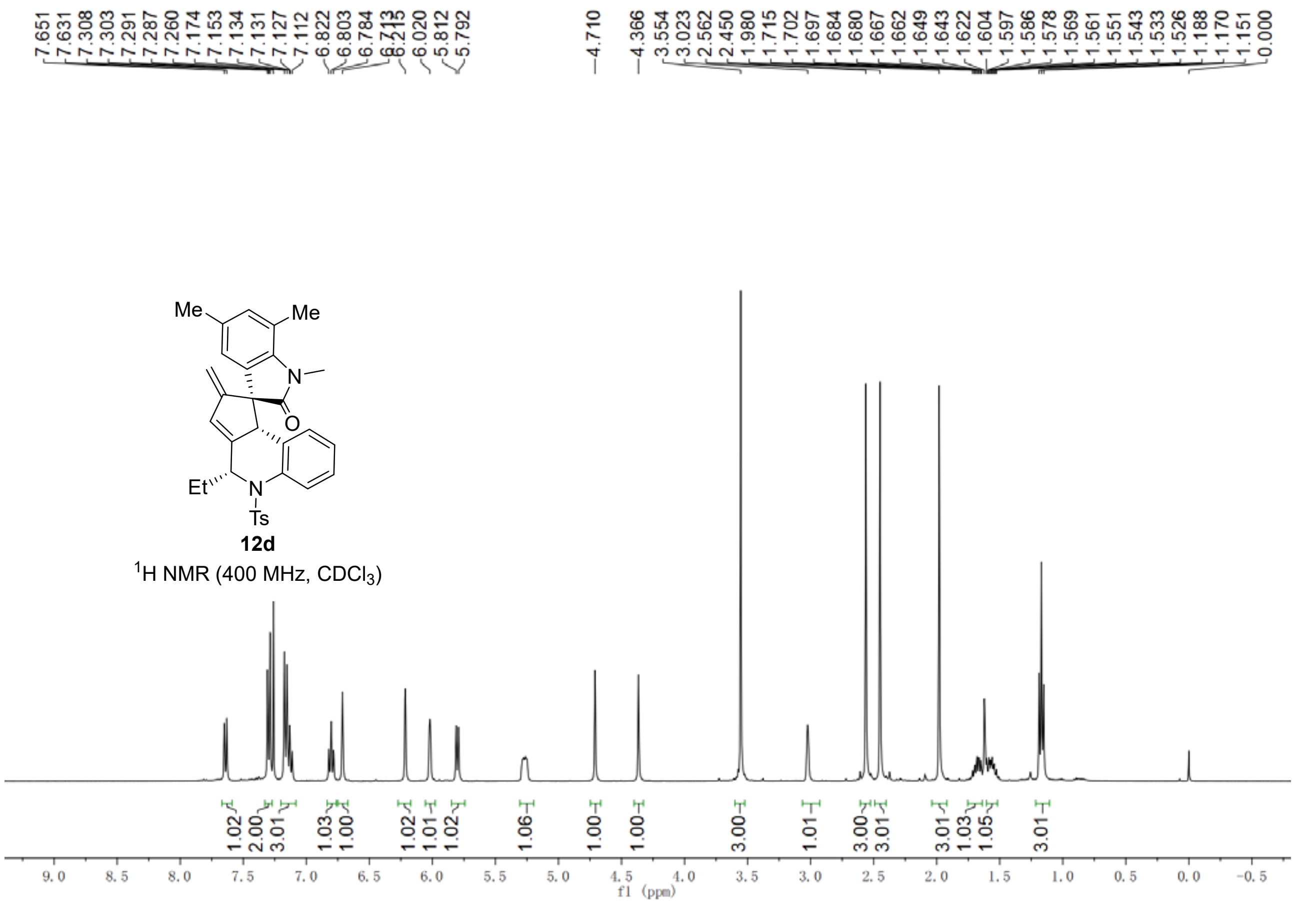

12d

^{1}H NMR (400 MHz, $CDCl_3$)

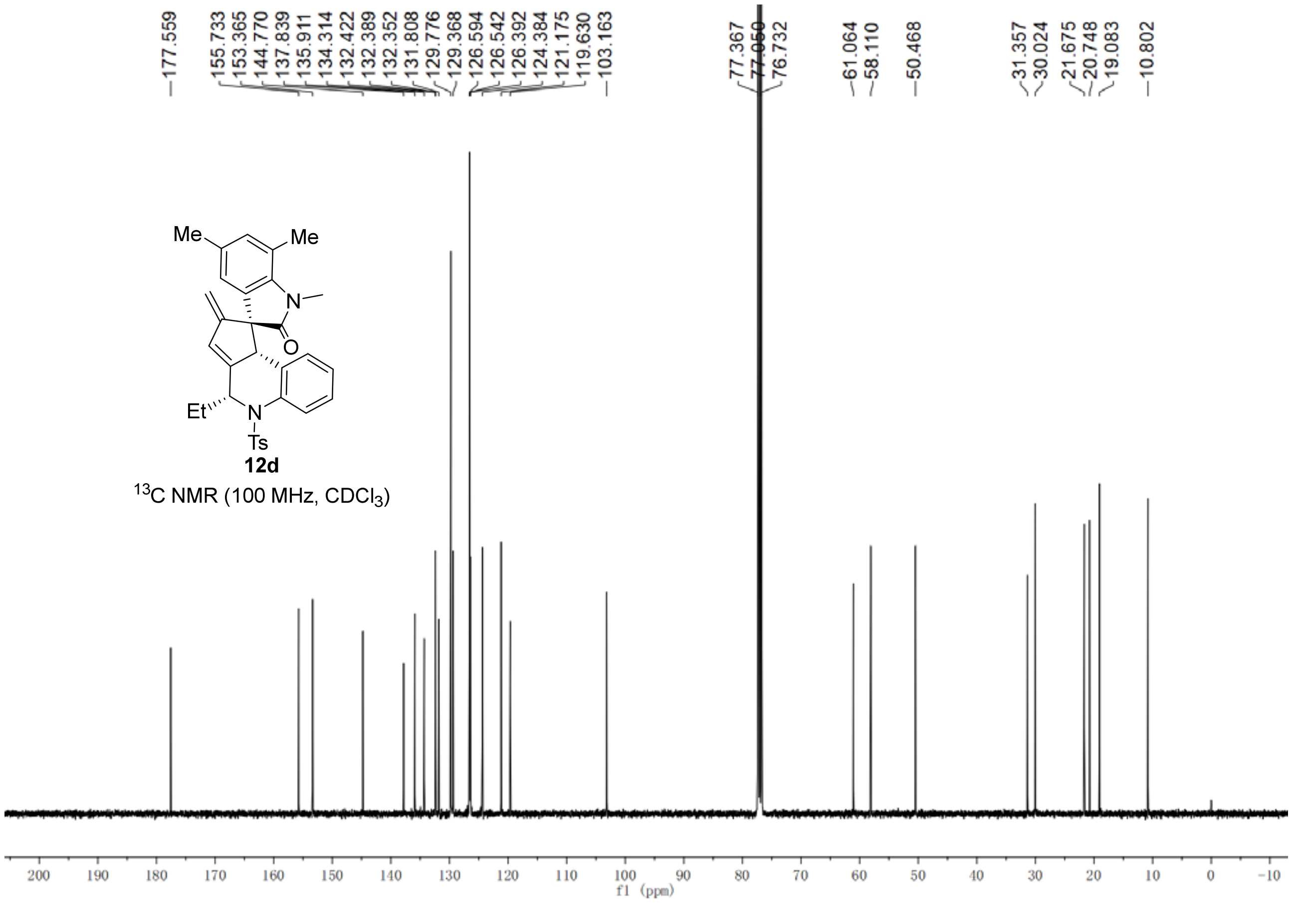

12d

^{13}C NMR (100 MHz, $CDCl_3$)

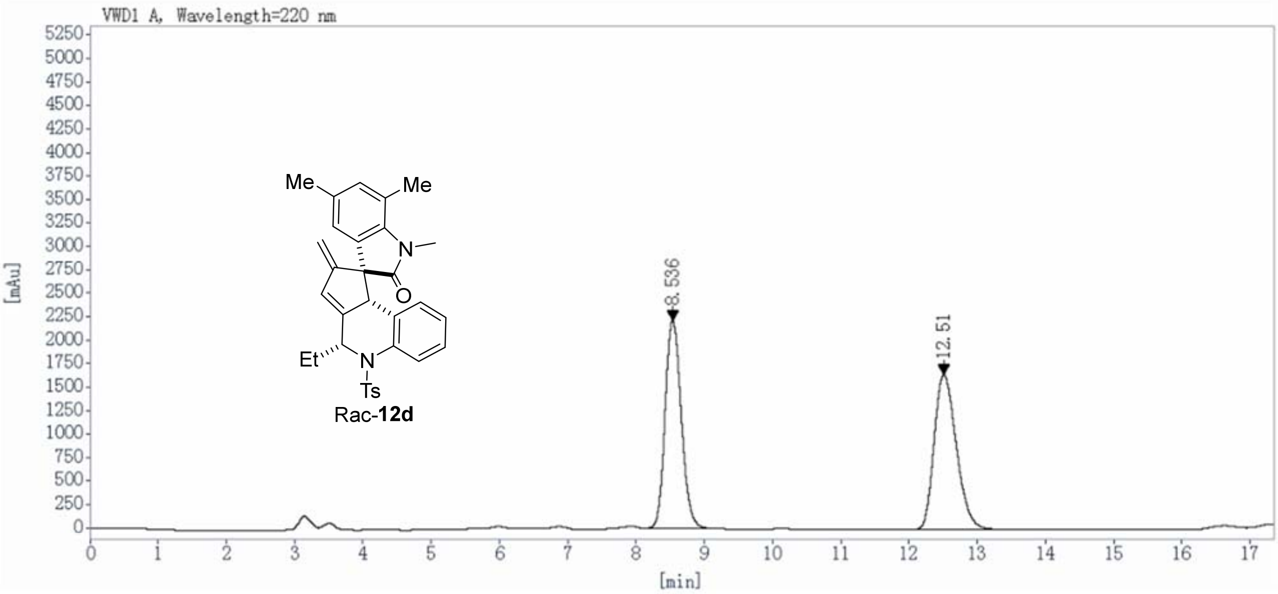

Ret Time [min]	Peak Type	Width [min]	Height [mAU]	Area [mAU*s]	Area [%]
8.536	BBA	0.26	2210.6050	36334.6484	49.2347
12.510	BBA	0.35	1650.5771	37464.1758	50.7653
			Totals:	73798.8242	100.0000

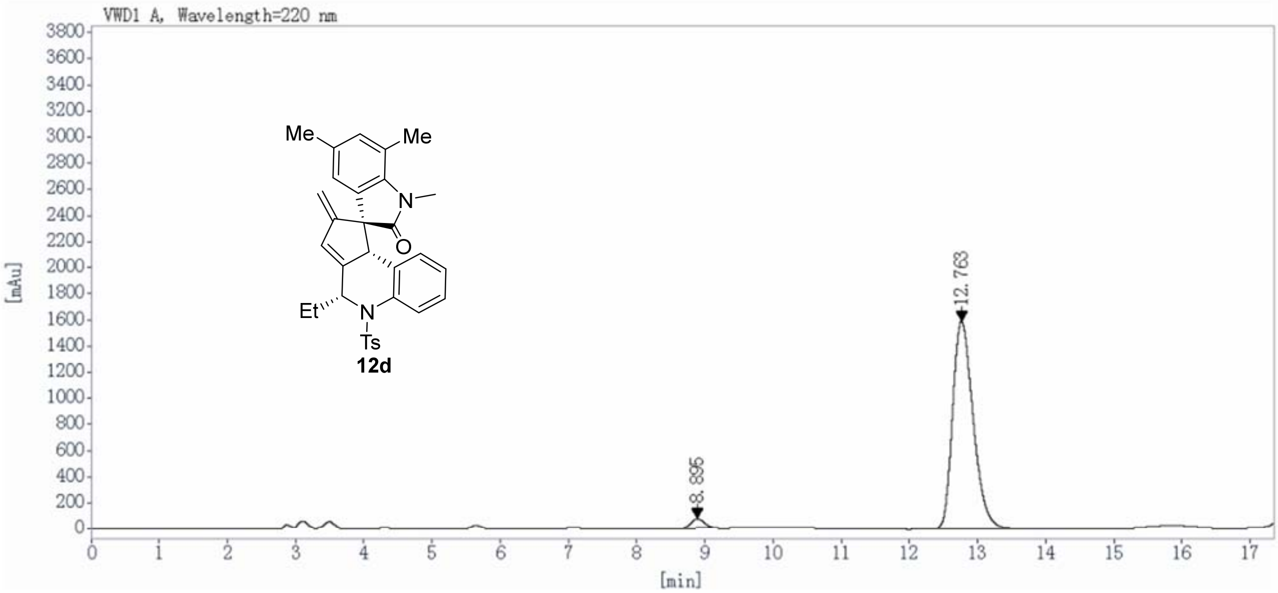

Ret Time [min]	Peak Type	Width [min]	Height [mAU]	Area [mAU*s]	Area [%]
8.895	BBA	0.21	72.8597	991.8591	2.8952
12.763	BBA	0.32	1591.7700	33266.9531	97.1048
			Totals:	34258.8123	100.0000

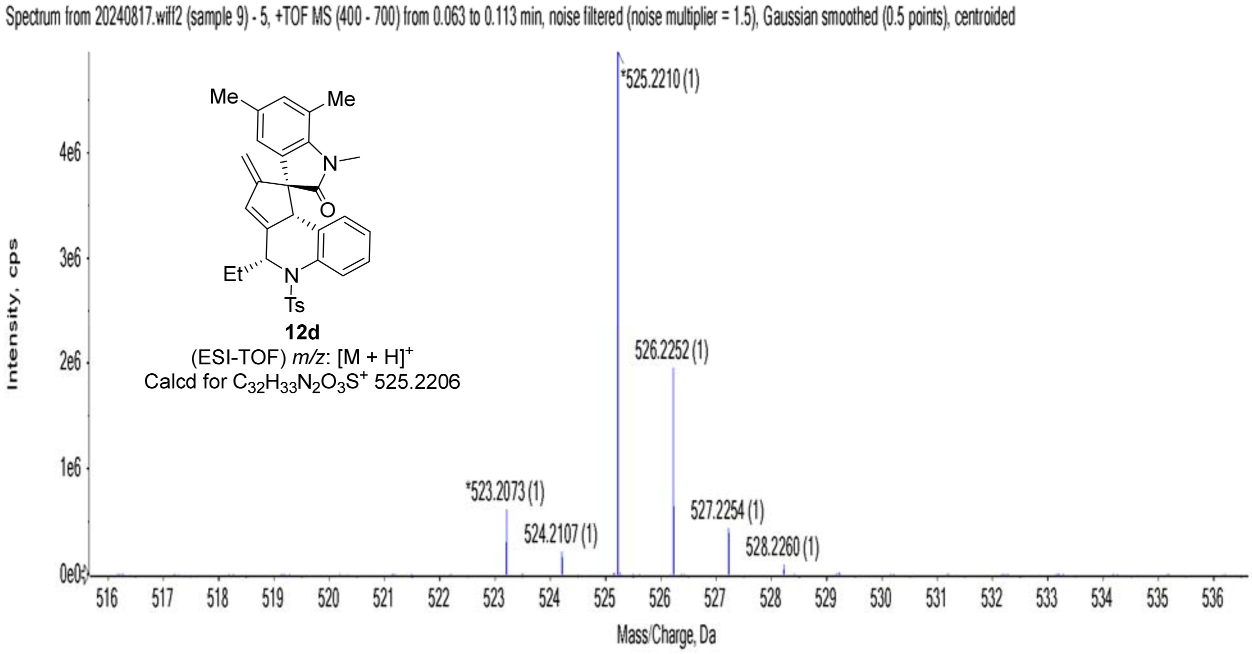

Spectrum from 20240817.wiff2 (sample 9) - 5, +TOF MS (400 - 700) from 0.063 to 0.113 min, noise filtered (noise multiplier = 1.5), Gaussian smoothed (0.5 points), centroided
Me
Me
N
O
Et
N
Ts
12d
(ESI-TOF) m/z: [M + H]+
Calcd for C32H33N2O3S+ 525.2206
Intensity, cps
4e6
3e6
2e6
1e6
0e0
*525.2210 (1)
526.2252 (1)
*523.2073 (1)
524.2107 (1)
527.2254 (1)
528.2260 (1)
516
517
518
519
520
521
522
523
524
525
526
527
528
529
530
531
532
533
534
535
536
Mass/Charge, Da

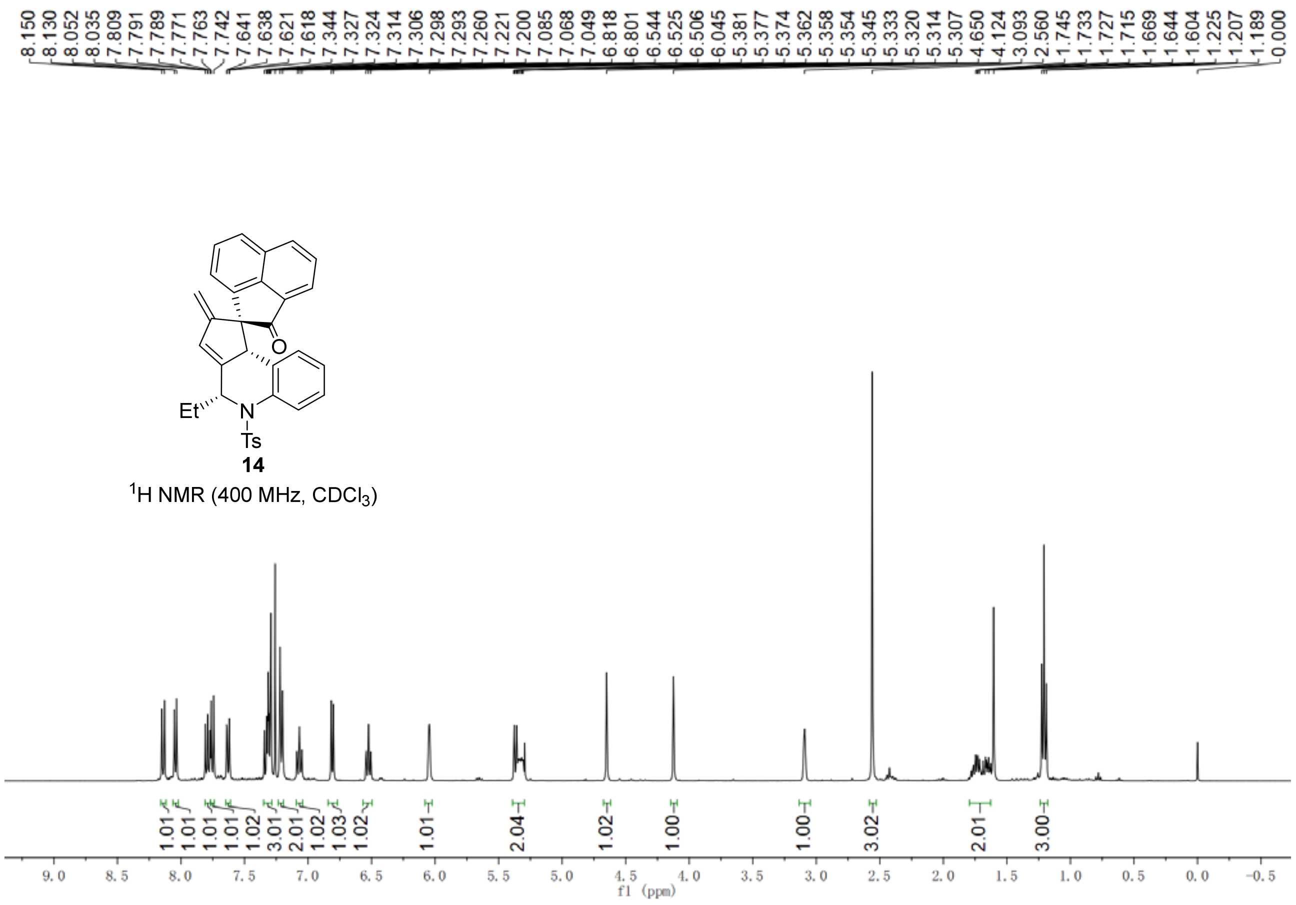
8.150
8.130
8.052
8.035
7.809
7.791
7.789
7.771
7.763
7.742
7.641
7.638
7.621
7.618
7.344
7.327
7.324
7.314
7.306
7.298
7.293
7.260
7.221
7.200
7.085
7.068
7.049
6.818
6.801
6.544
6.525
6.506
6.045
5.381
5.377
5.374
5.362
5.358
5.354
5.345
5.333
5.320
5.314
5.307
4.650
4.124
3.093
2.560
1.745
1.733
1.727
1.715
1.669
1.644
1.604
1.225
1.207
1.189
0.000
Et
N
Ts
O
14
^{1}H NMR (400 MHz, CDCl$_3$)
1.01
1.01
1.01
1.01
1.02
3.01
2.01
1.02
1.03
1.02
1.01
2.04
1.02
1.00
1.00
3.02
2.01
3.00
f1 (ppm)

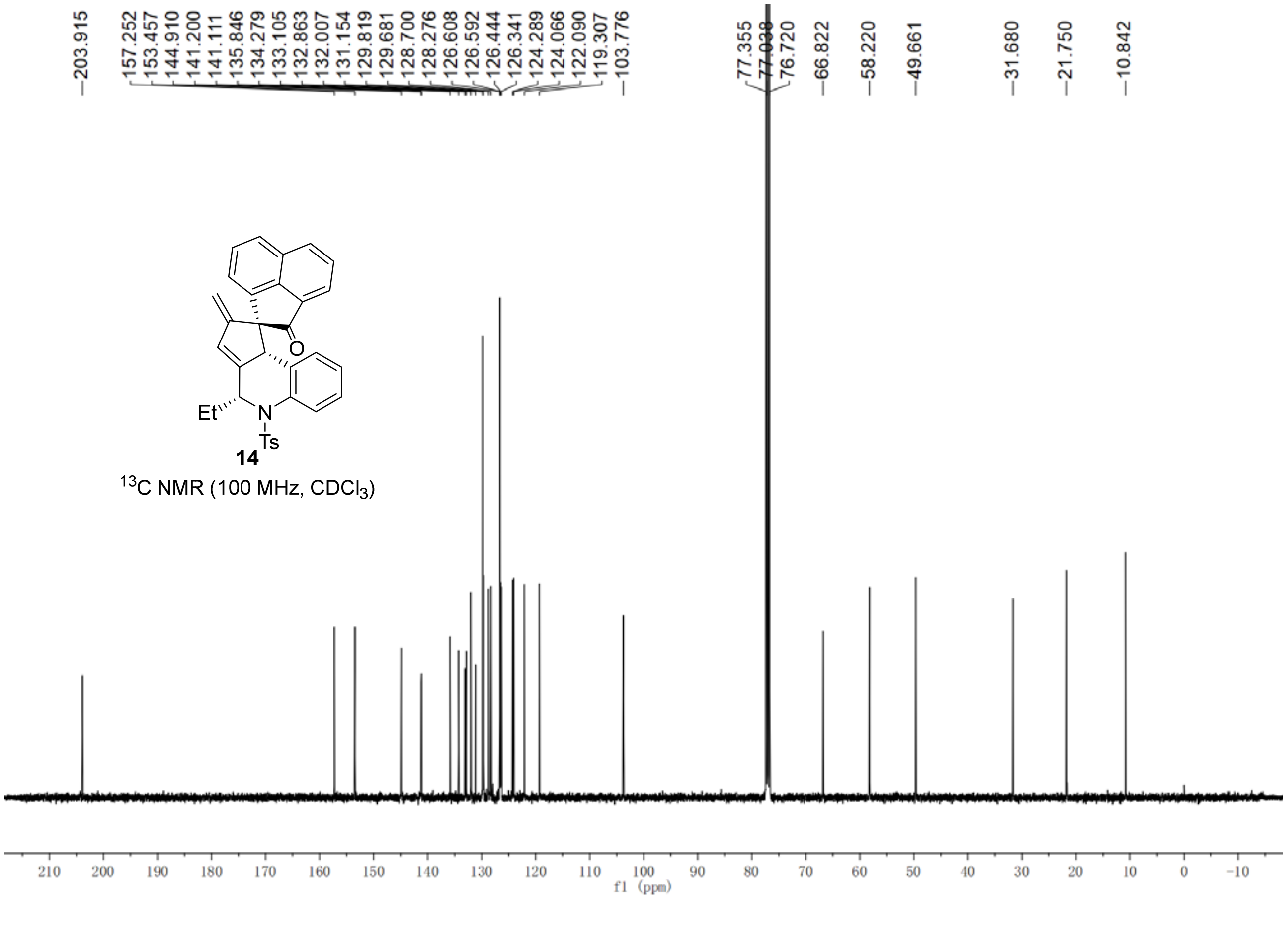
203.915
157.252
153.457
144.910
141.200
141.111
135.846
134.279
133.105
132.863
132.007
131.154
129.819
129.681
128.700
128.276
126.608
126.592
126.444
126.341
124.289
124.066
122.090
119.307
103.776
77.355
77.038
76.720
66.822
58.220
49.661
31.680
21.750
10.842
Et
N
Ts
O
14
^{13}C NMR (100 MHz, CDCl$_3$)
f1 (ppm)

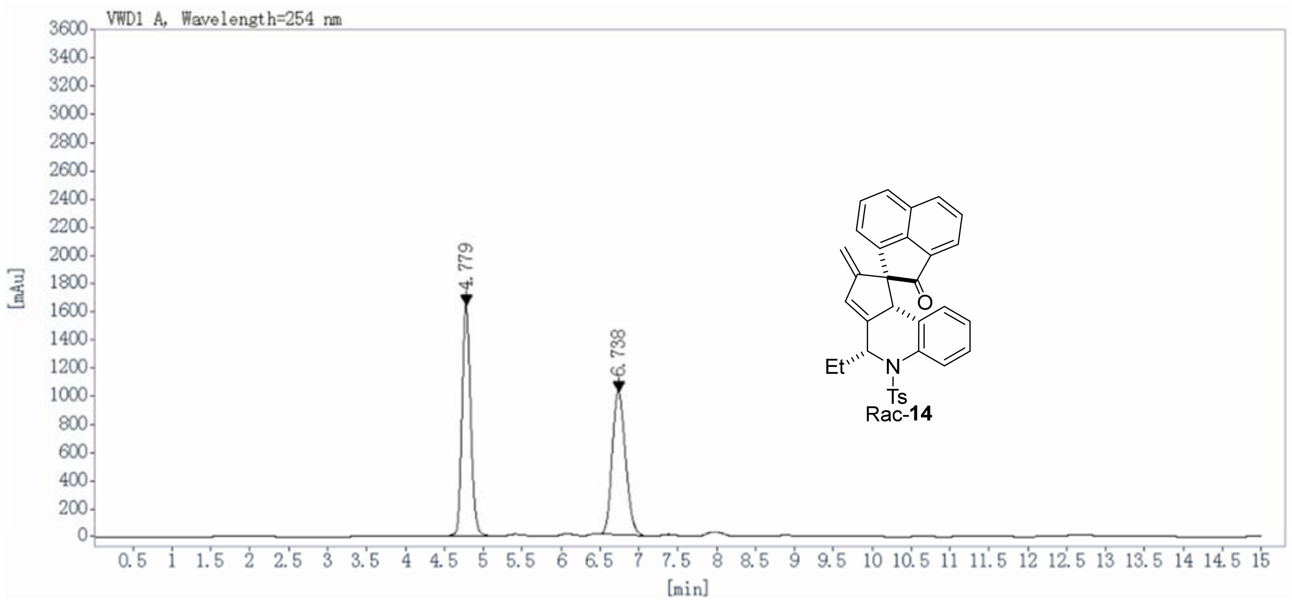

Ret Time [min]	Peak Type	Width [min]	Height [mAU]	Area [mAU*s]	Area [%]
4.779	BBA	0.12	1636.8729	12525.2695	50.5571
6.738	BBA	0.19	1018.9764	12249.2441	49.4429
			Totals:	24774.5137	100.0000

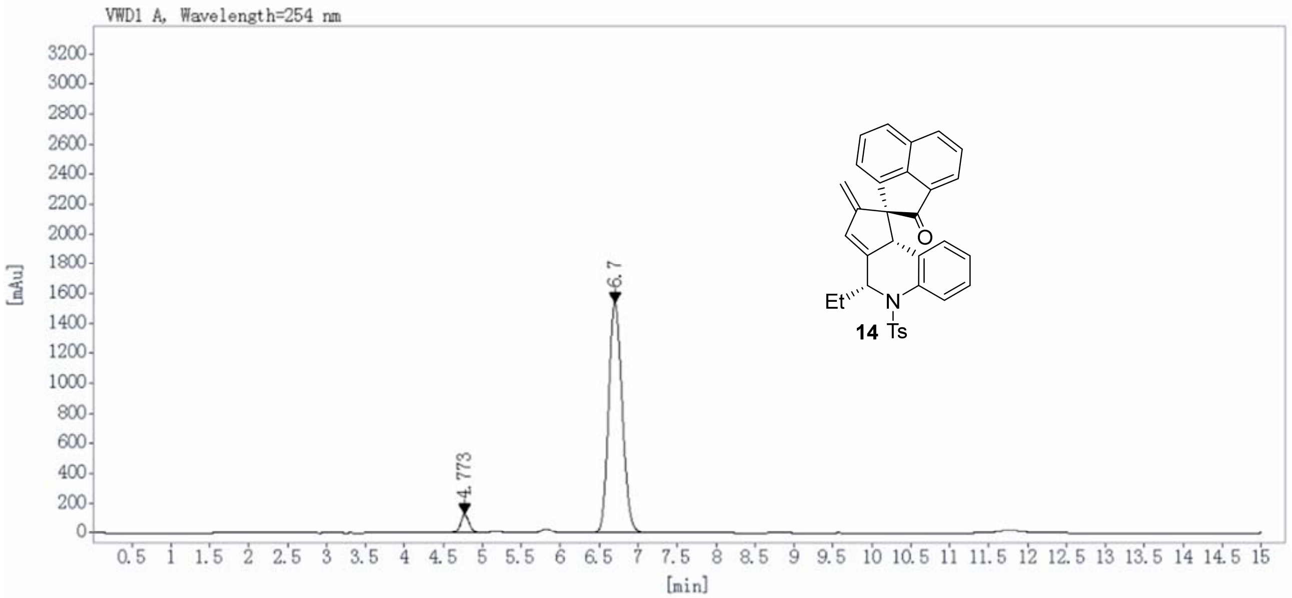

Ret Time [min]	Peak Type	Width [min]	Height [mAU]	Area [mAU*s]	Area [%]
4.773	BB	0.12	124.6385	955.9208	4.8654
6.700	BBA	0.19	1540.2458	18691.2891	95.1346
			Totals:	19647.2099	100.0000

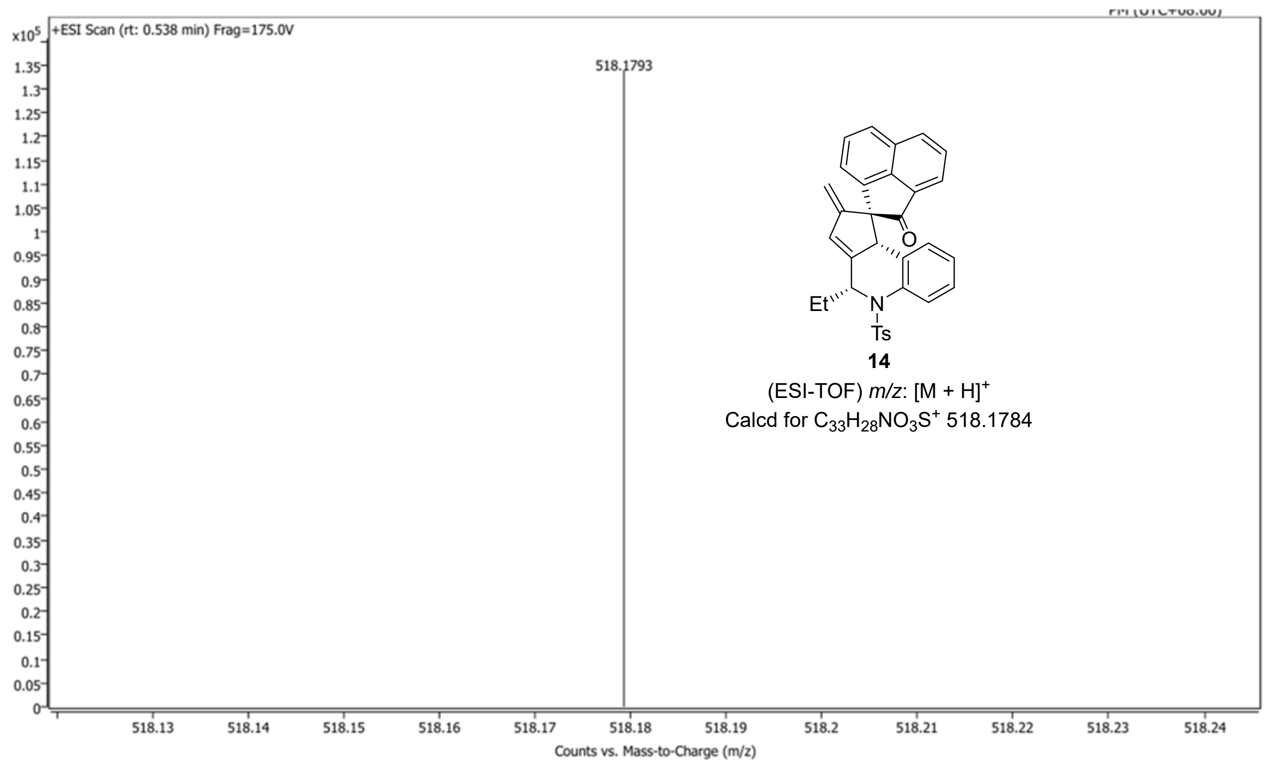

(ESI-TOF) *m/z*: $[M + H]^+$

Calcd for $C_{33}H_{28}NO_3S^+$ 518.1784

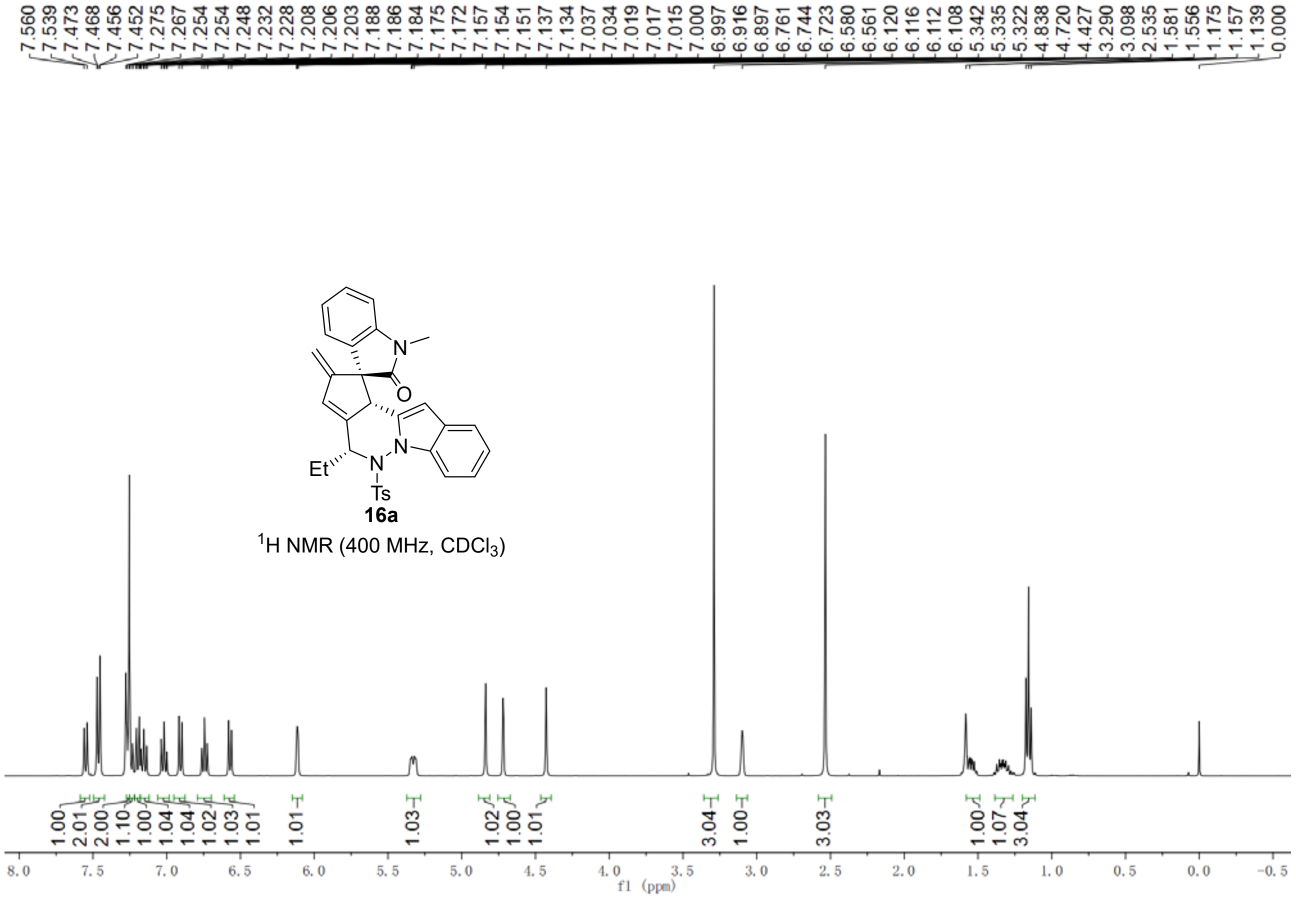

16a

^{1}H NMR (400 MHz, $CDCl_3$)

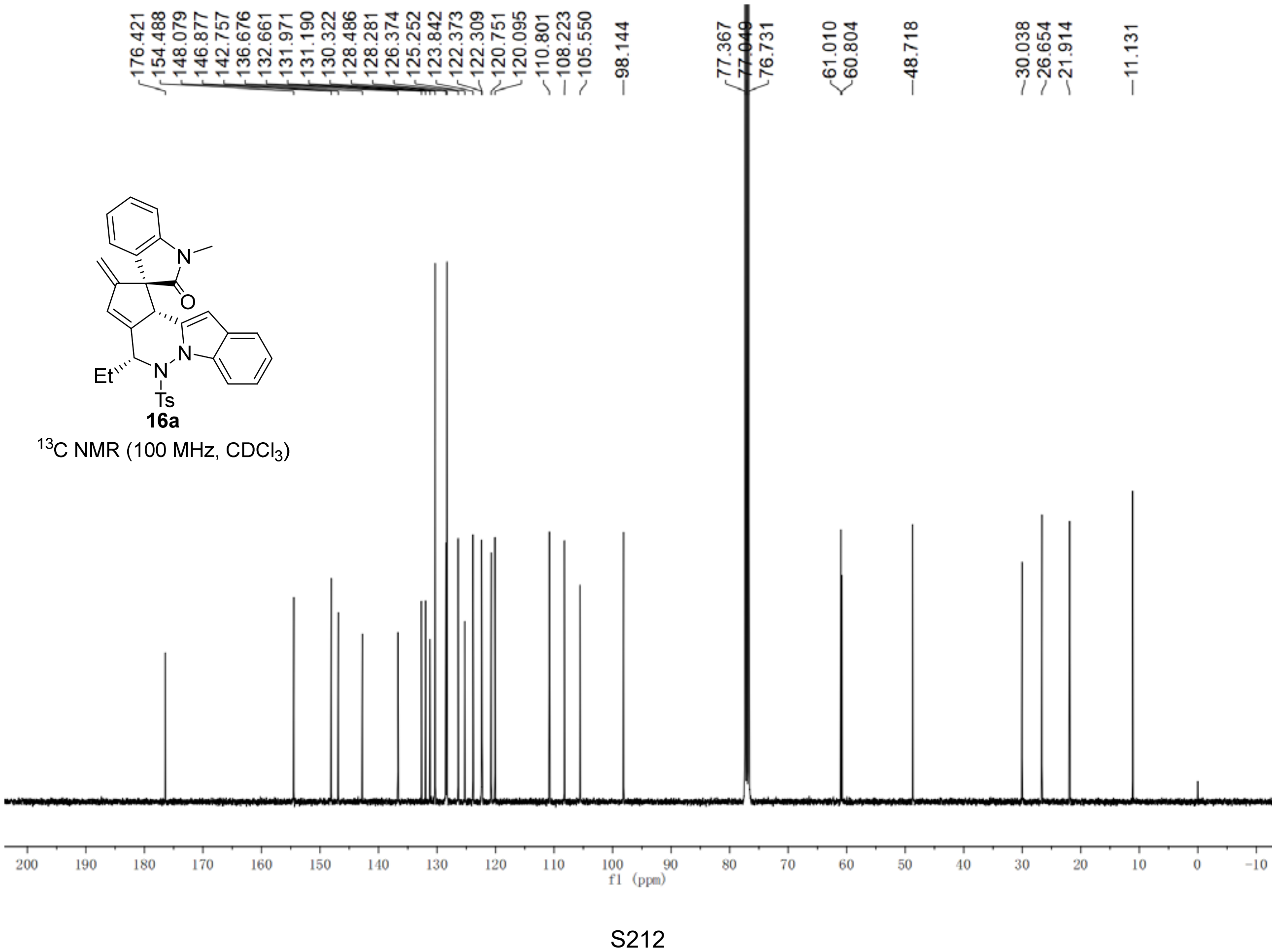

16a

^{13}C NMR (100 MHz, $CDCl_3$)

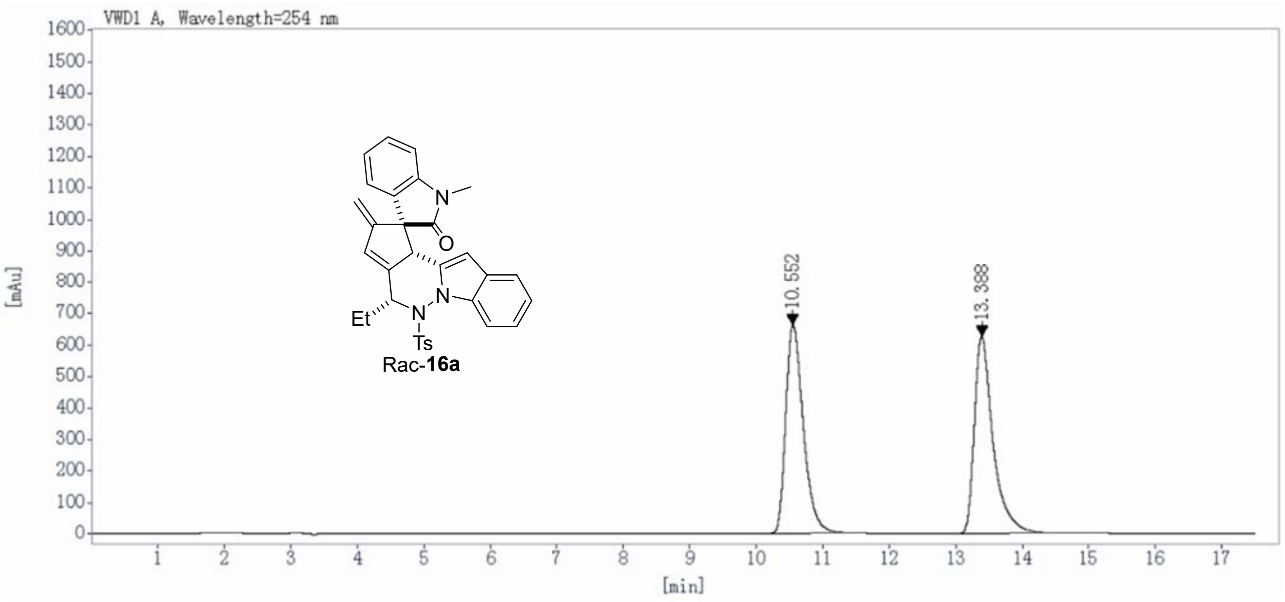

Ret Time [min]	Peak Type	Width [min]	Height [mAU]	Area [mAU*s]	Area [%]
10.552	BBA	0.28	663.3882	12264.3691	49.8749
13.388	BBA	0.29	626.3311	12325.8848	50.1251
			Totals:	24590.2539	100.0000

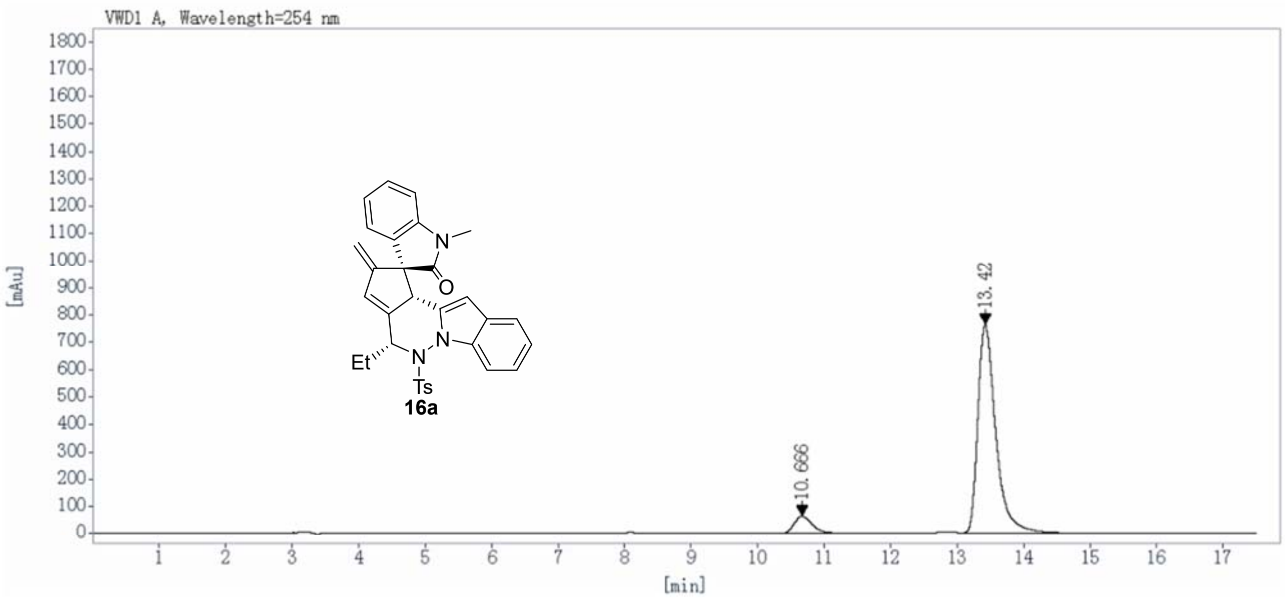

Ret Time [min]	Peak Type	Width [min]	Height [mAU]	Area [mAU*s]	Area [%]
10.666	BB	0.29	63.5017	1185.3958	7.6949
13.420	BBA	0.28	763.5140	14219.4668	92.3051
			Totals:	15404.8625	100.0000

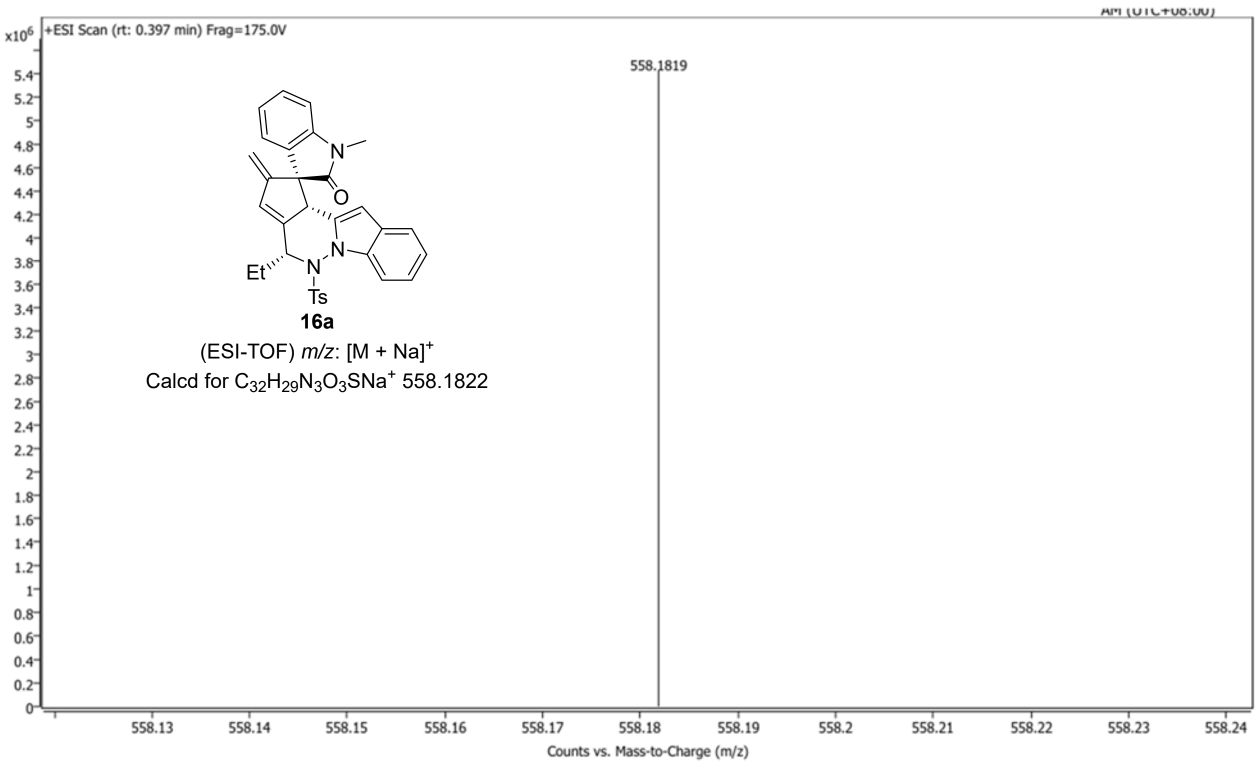

AM (UTC+08:00)
x10^6
+ESI Scan (rt: 0.397 min) Frag=175.0V
558.1819
N
O
N
N
Et
Ts
16a
(ESI-TOF) m/z: [M + Na]+
Calcd for C32H29N3O3SNa+ 558.1822
Counts vs. Mass-to-Charge (m/z)

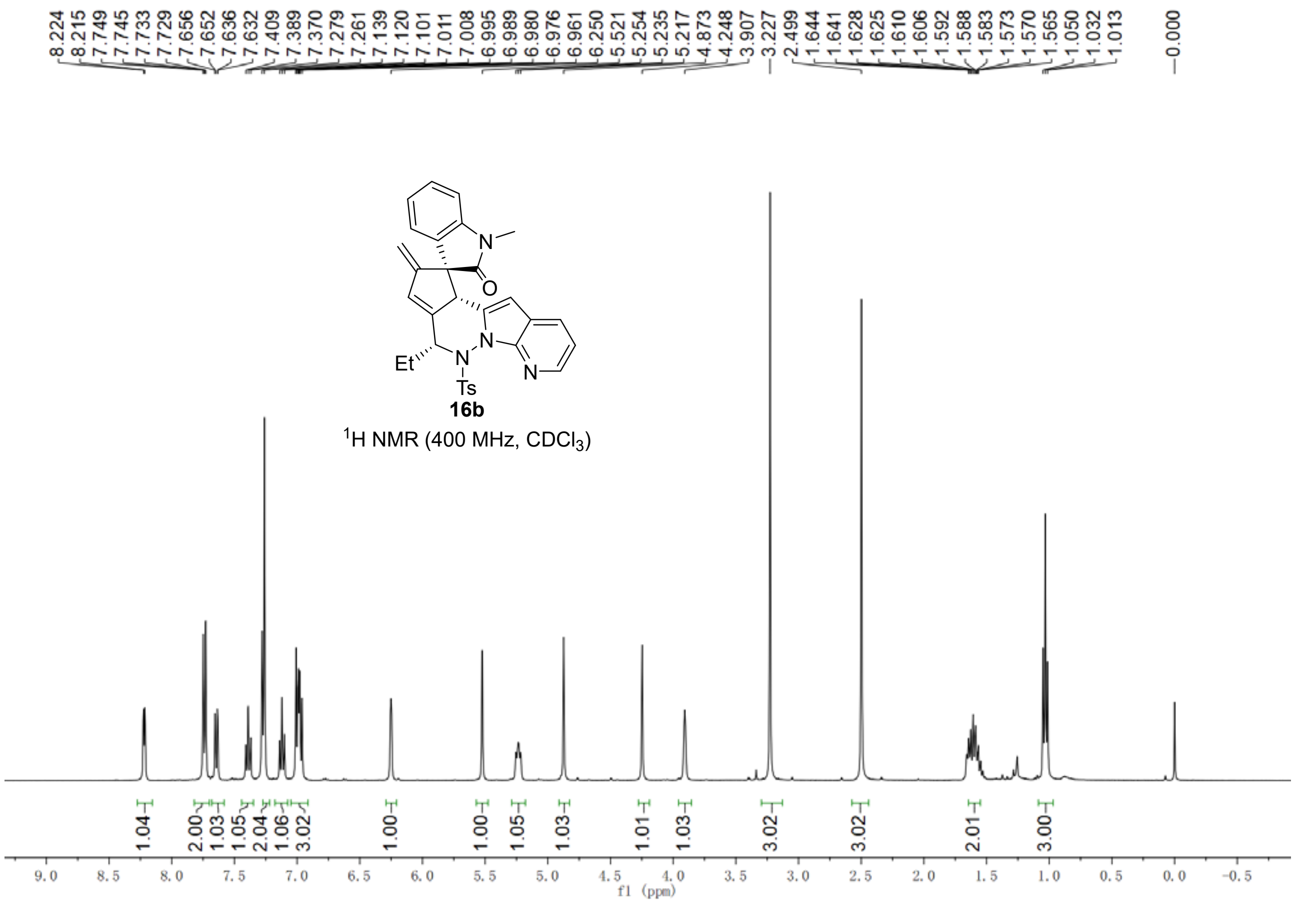
8.224
8.215
7.749
7.745
7.733
7.729
7.656
7.652
7.636
7.632
7.409
7.389
7.370
7.279
7.261
7.139
7.120
7.101
7.011
7.008
6.995
6.989
6.980
6.976
6.961
6.250
5.521
5.254
5.235
5.217
4.873
4.248
3.907
3.227
2.499
1.644
1.641
1.628
1.625
1.610
1.606
1.592
1.588
1.583
1.573
1.570
1.565
1.050
1.032
1.013
0.000
Et
N
Ts
N
O
16b
^{1}H NMR (400 MHz, $CDCl_3$)
1.04
2.00
1.03
1.05
2.04
1.06
3.02
1.00
1.00
1.05
1.03
1.01
1.03
3.02
3.02
2.01
3.00
f1 (ppm)

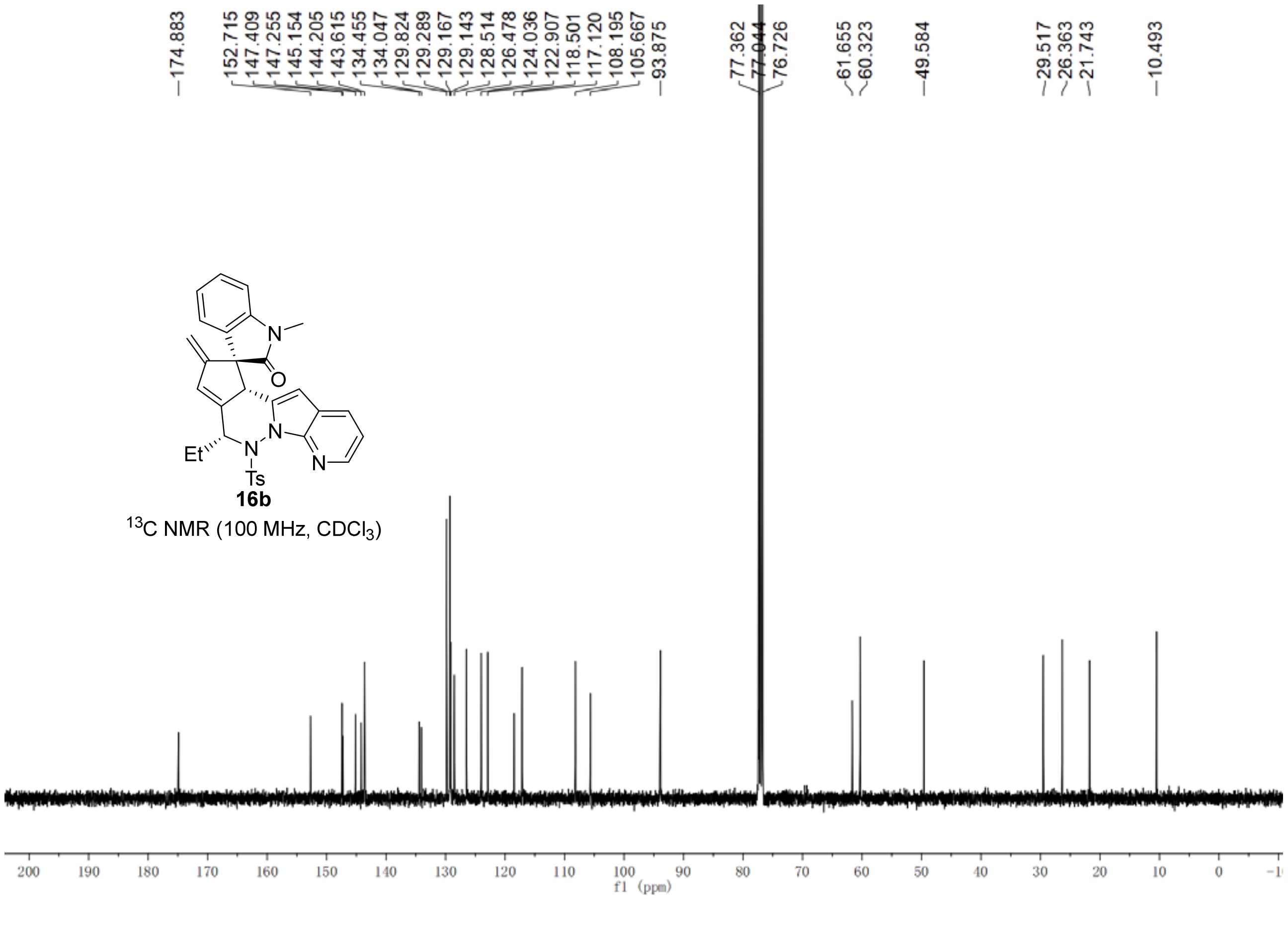
174.883
152.715
147.409
147.255
145.154
144.205
143.615
134.455
134.047
129.824
129.289
129.167
129.143
128.514
126.478
124.036
122.907
118.501
117.120
108.195
105.667
93.875
77.362
77.044
76.726
61.655
60.323
49.584
29.517
26.363
21.743
10.493
Et
N
Ts
N
O
16b
^{13}C NMR (100 MHz, $CDCl_3$)
f1 (ppm)

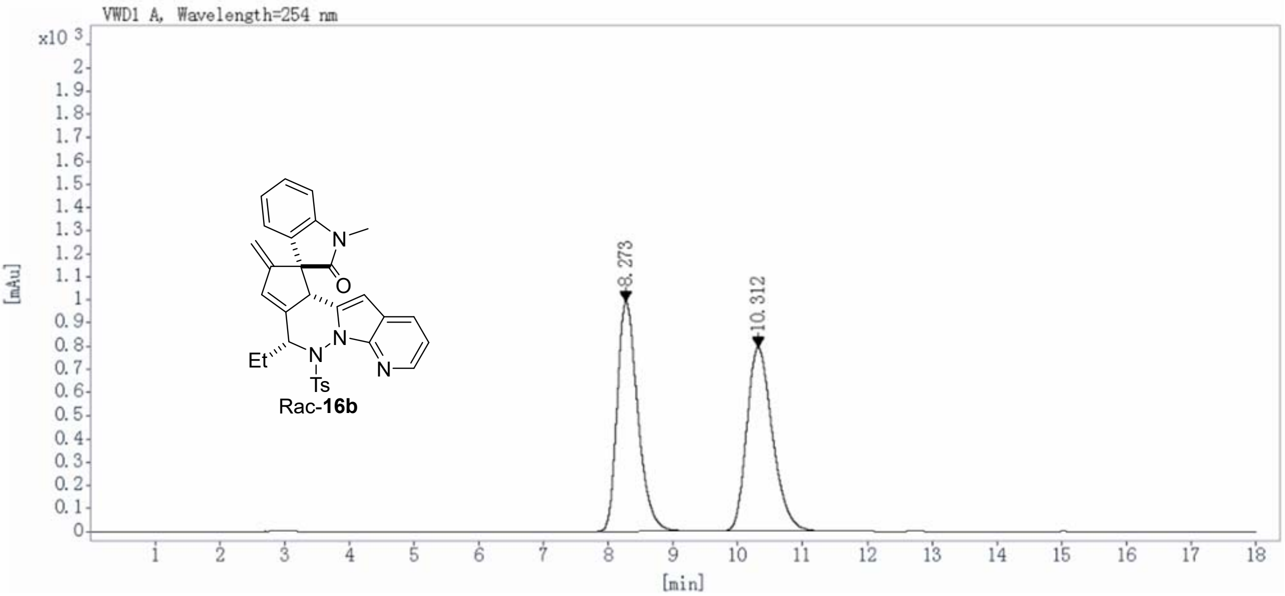

Ret Time [min]	Peak Type	Width [min]	Height [mAU]	Area [mAU*s]	Area [%]
8.273	BBA	0.34	991.5527	21815.1191	49.8887
10.312	BBA	0.43	793.1179	21912.4746	50.1113
			Totals:	43727.5938	100.0000

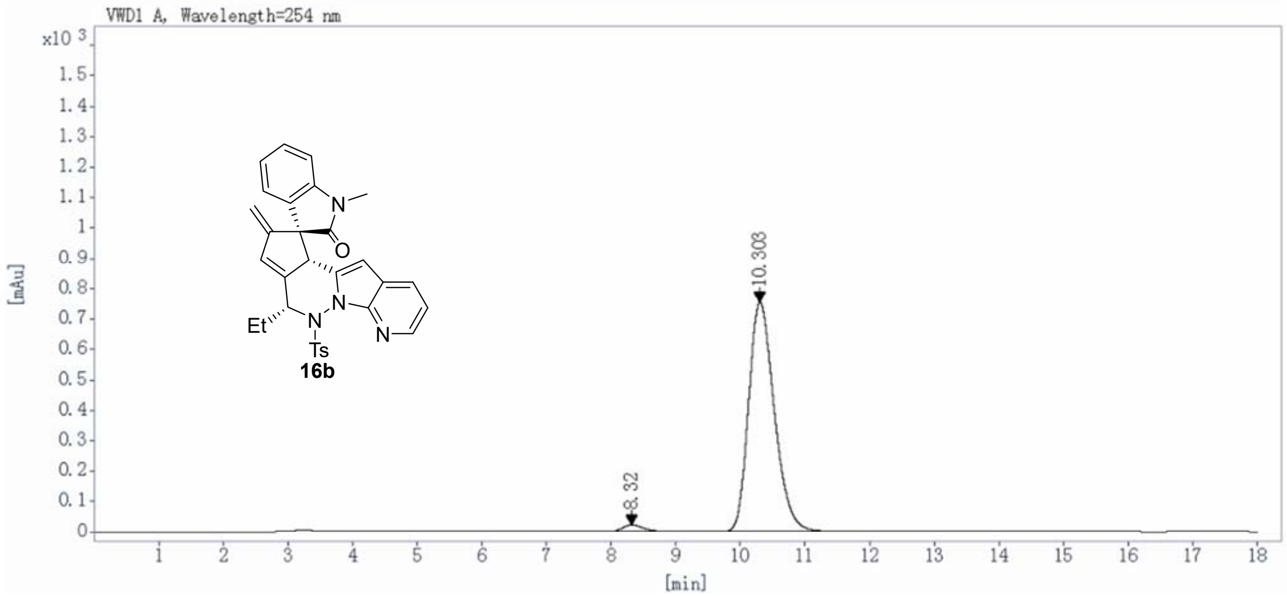

Ret Time [min]	Peak Type	Width [min]	Height [mAU]	Area [mAU*s]	Area [%]
8.320	BBA	0.33	22.4122	476.5341	2.2473
10.303	BBA	0.43	753.7796	20728.4980	97.7527
			Totals:	21205.0321	100.0000

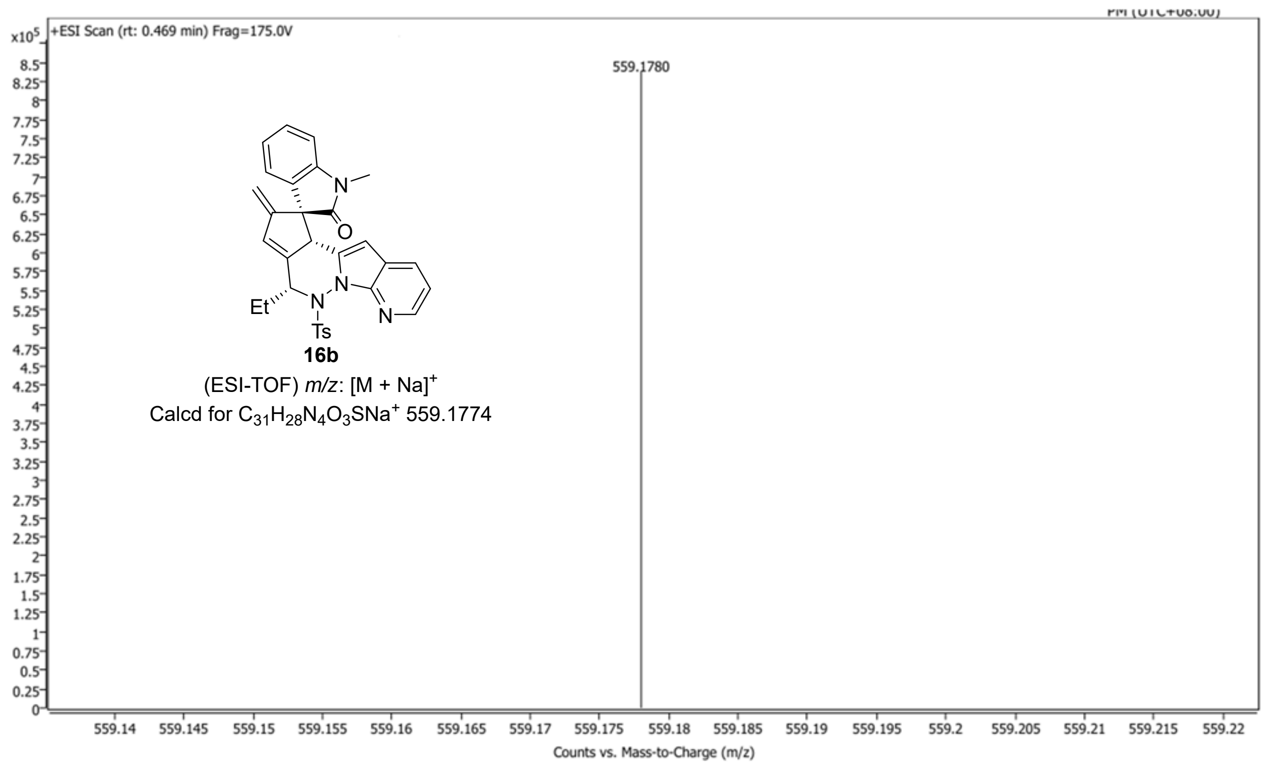
+ESI Scan (rt: 0.469 min) Frag=175.0V
x10^5
559.1780
16b
(ESI-TOF) m/z: [M + Na]+
Calcd for C31H28N4O3SNa+ 559.1774
Counts vs. Mass-to-Charge (m/z)

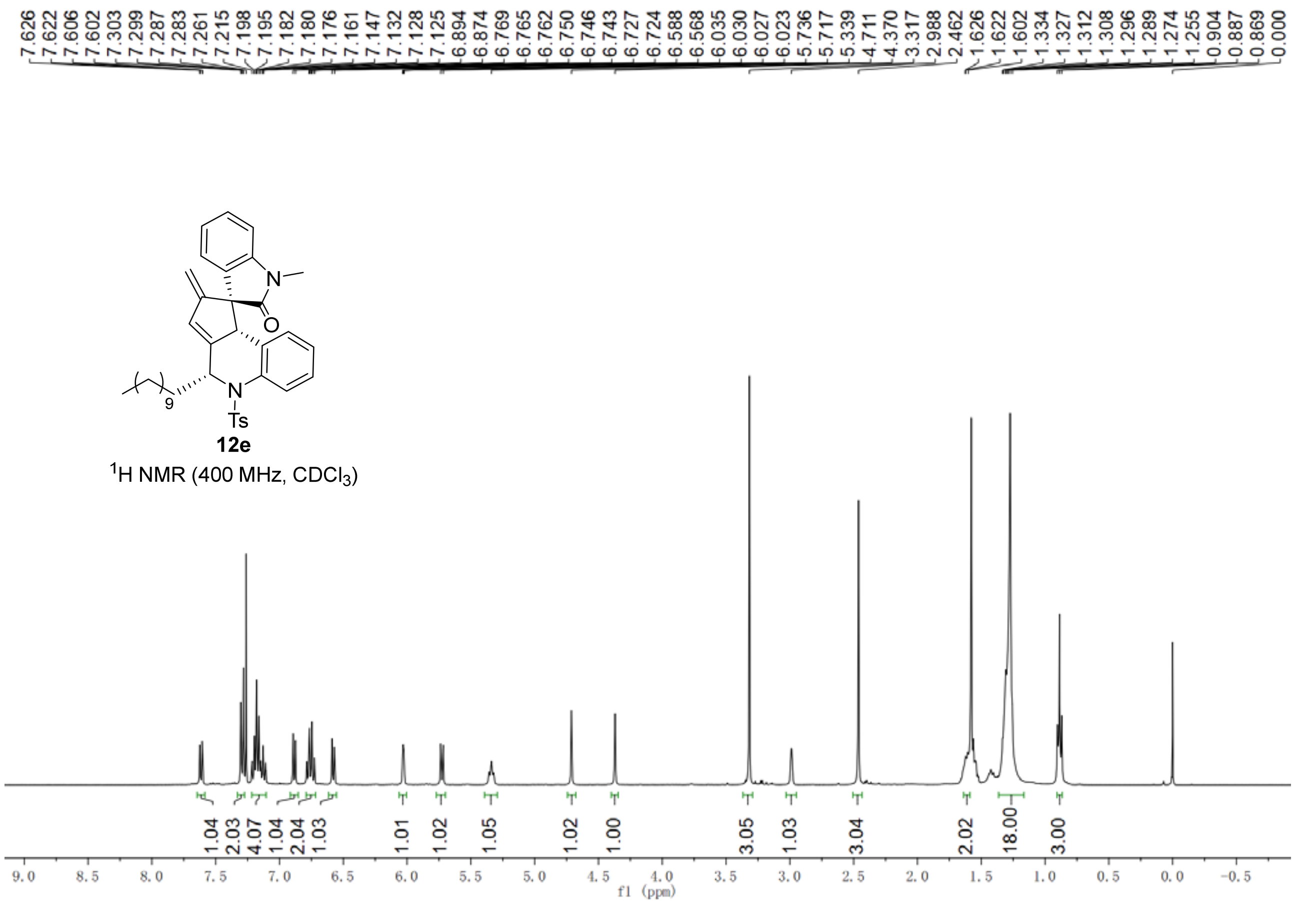
7.626
7.622
7.606
7.602
7.303
7.299
7.287
7.283
7.261
7.215
7.198
7.195
7.182
7.180
7.176
7.161
7.147
7.132
7.128
7.125
6.894
6.874
6.769
6.765
6.762
6.750
6.746
6.743
6.727
6.724
6.588
6.568
6.035
6.030
6.027
6.023
5.736
5.717
5.339
4.711
4.370
3.317
2.988
2.462
1.626
1.622
1.602
1.334
1.327
1.312
1.308
1.296
1.289
1.274
1.255
0.904
0.887
0.869
0.000
N
O
9
Ts
12e
1H NMR (400 MHz, CDCl3)
1.04
2.03
4.07
1.04
2.04
1.03
1.01
1.02
1.05
1.02
1.00
3.05
1.03
3.04
2.02
18.00
3.00
9.0 8.5 8.0 7.5 7.0 6.5 6.0 5.5 5.0 4.5 4.0 3.5 3.0 2.5 2.0 1.5 1.0 0.5 0.0 -0.5
f1 (ppm)

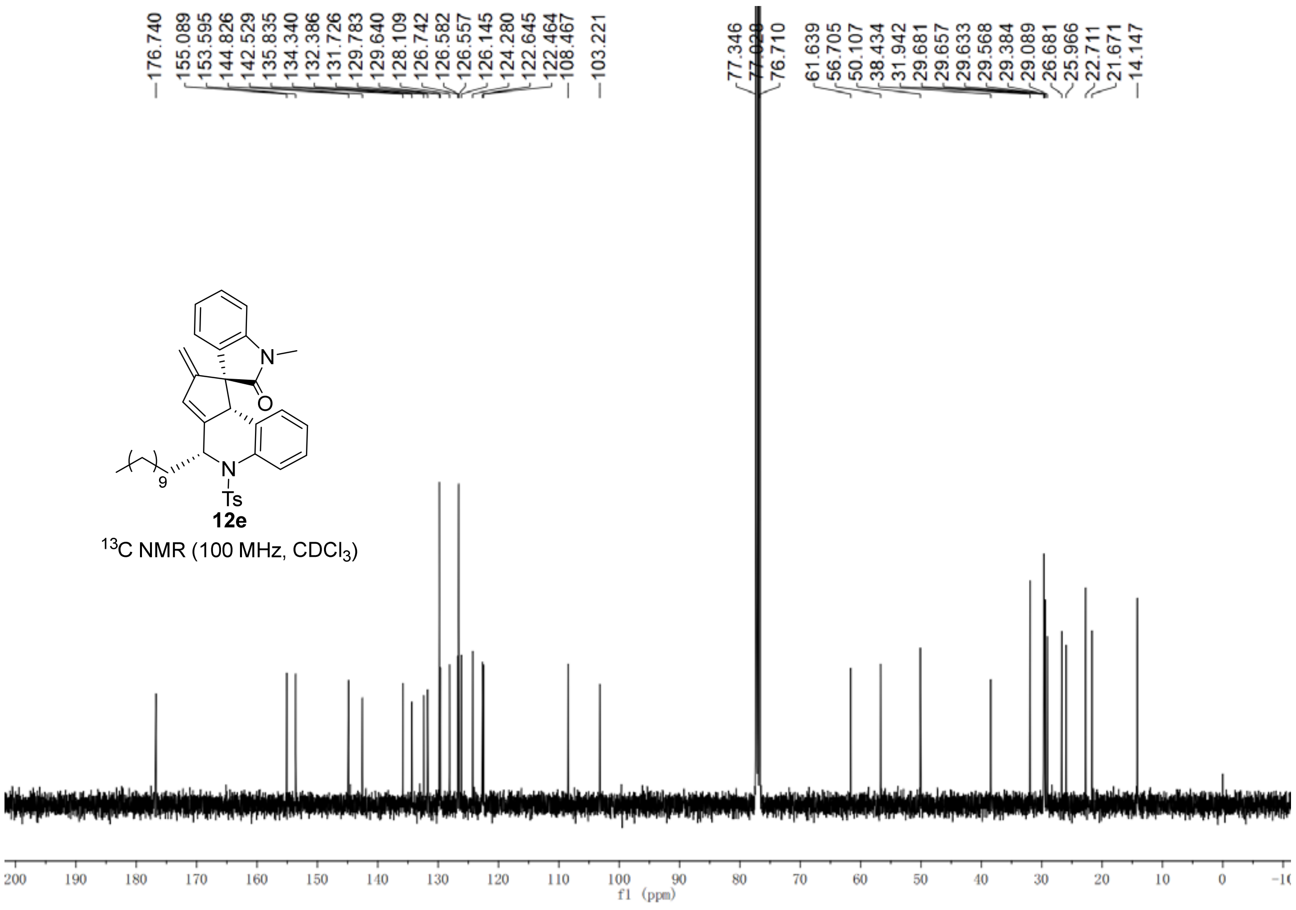
176.740
155.089
153.595
144.826
142.529
135.835
134.340
132.386
131.726
129.783
129.640
128.109
126.742
126.582
126.557
126.145
124.280
122.645
122.464
108.467
103.221
77.346
[illegible]
76.710
61.639
56.705
50.107
38.434
31.942
29.681
29.657
29.633
29.568
29.384
29.089
26.681
25.966
22.711
21.671
14.147
N
O
9
Ts
12e
13C NMR (100 MHz, CDCl3)
200 190 180 170 160 150 140 130 120 110 100 90 80 70 60 50 40 30 20 10 0 -10
f1 (ppm)

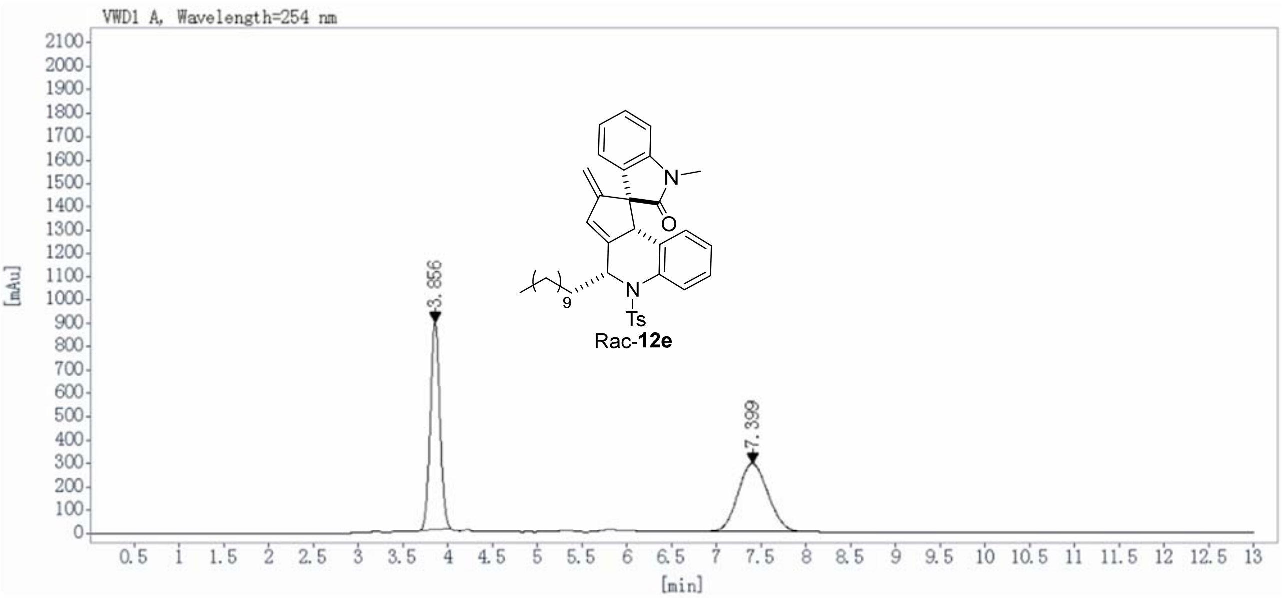

Ret Time [min]	Peak Type	Width [min]	Height [mAU]	Area [mAU*s]	Area [%]
3.856	BBA	0.12	888.1533	6696.6675	49.0735
7.399	BB	0.37	294.7439	6949.5410	50.9265
			Totals:	13646.2085	100.0000

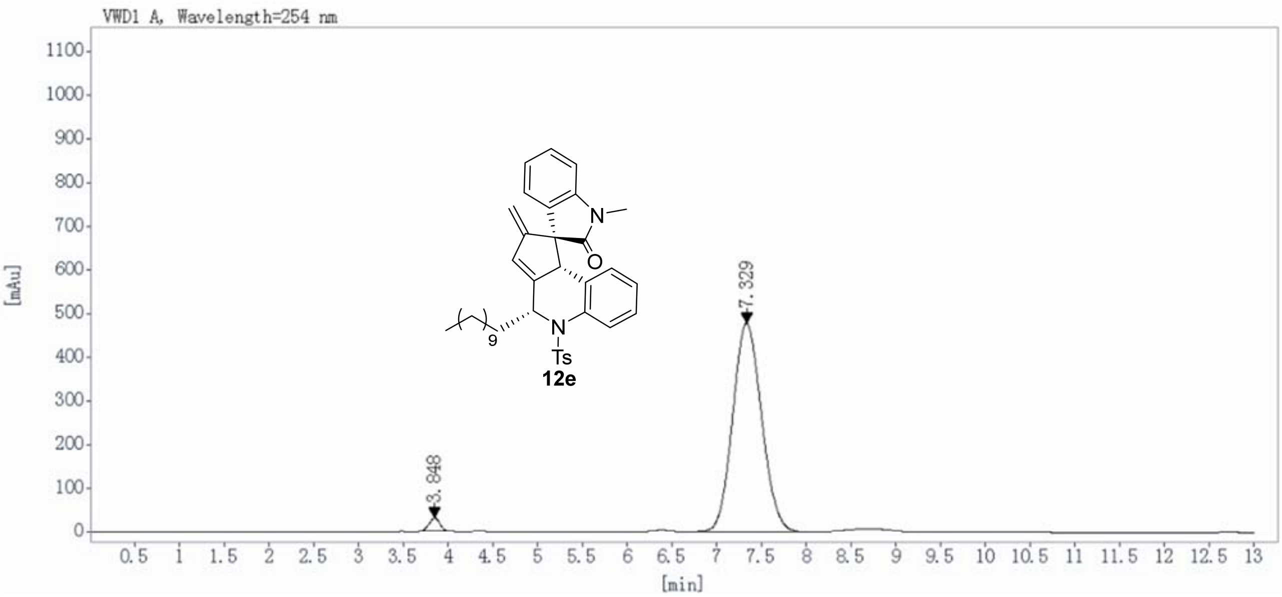

Ret Time [min]	Peak Type	Width [min]	Height [mAU]	Area [mAU*s]	Area [%]
3.848	BBA	0.13	30.5947	259.3460	2.3498
7.329	BBA	0.35	477.0155	10777.4502	97.6502
			Totals:	11036.7962	100.0000

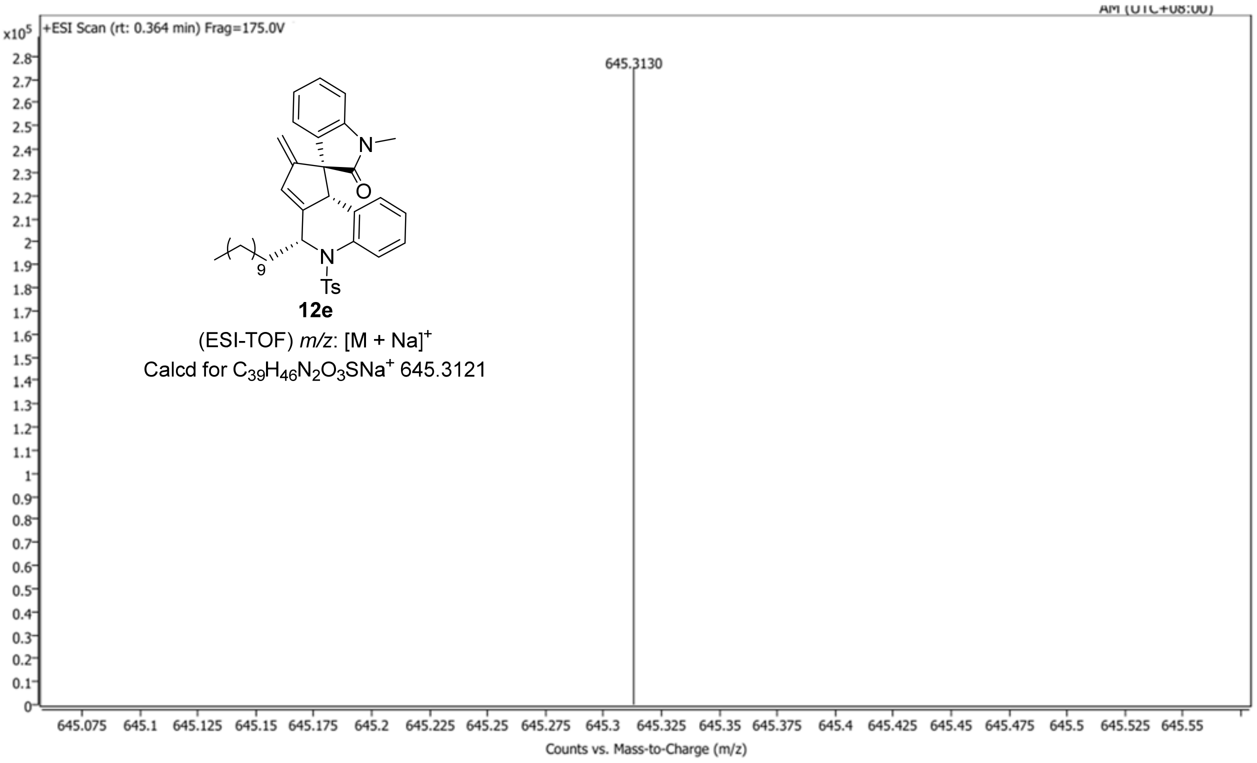
+ESI Scan (rt: 0.364 min) Frag=175.0V
x10^5
645.3130
N
O
Ts
9
12e
(ESI-TOF) m/z: [M + Na]+
Calcd for C39H46N2O3SNa+ 645.3121
Counts vs. Mass-to-Charge (m/z)

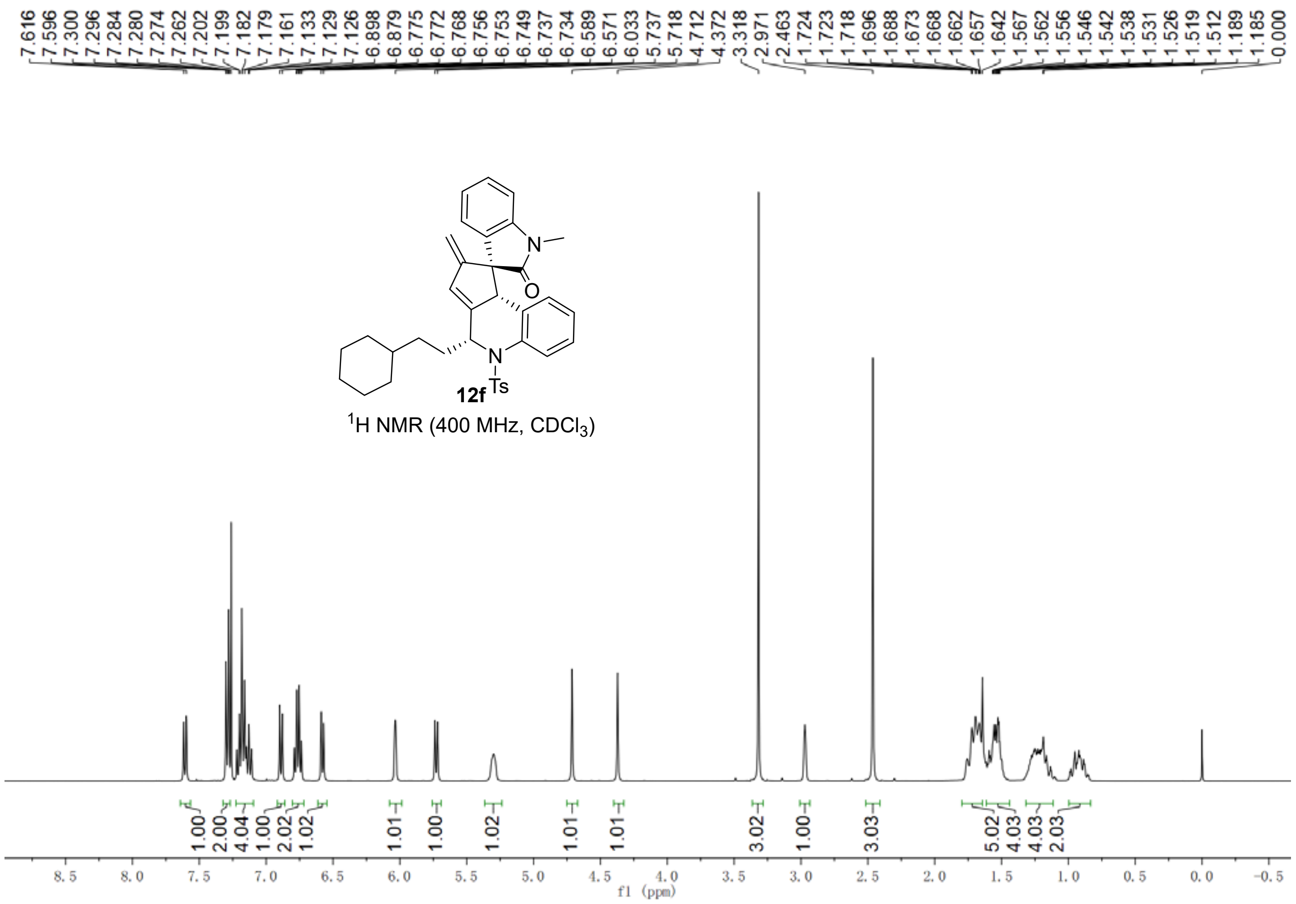
7.616 7.596 7.300 7.296 7.284 7.280 7.274 7.262 7.202 7.199 7.182 7.179 7.161 7.133 7.129 7.126 6.898 6.879 6.775 6.772 6.768 6.756 6.753 6.749 6.737 6.734 6.589 6.571 6.033 5.737 5.718 4.712 4.372 3.318 2.971 2.463 1.724 1.723 1.718 1.696 1.688 1.673 1.668 1.662 1.657 1.642 1.567 1.562 1.556 1.546 1.542 1.538 1.531 1.526 1.519 1.512 1.189 1.185 0.000
N
O
Ts
12f
^{1}H NMR (400 MHz, CDCl$_3$)
1.00 2.00 4.04 1.00 2.02 1.02 1.01 1.00 1.02 1.01 1.01 3.02 1.00 3.03 5.02 4.03 4.03 2.03
8.5 8.0 7.5 7.0 6.5 6.0 5.5 5.0 4.5 4.0 3.5 3.0 2.5 2.0 1.5 1.0 0.5 0.0 -0.5
f1 (ppm)

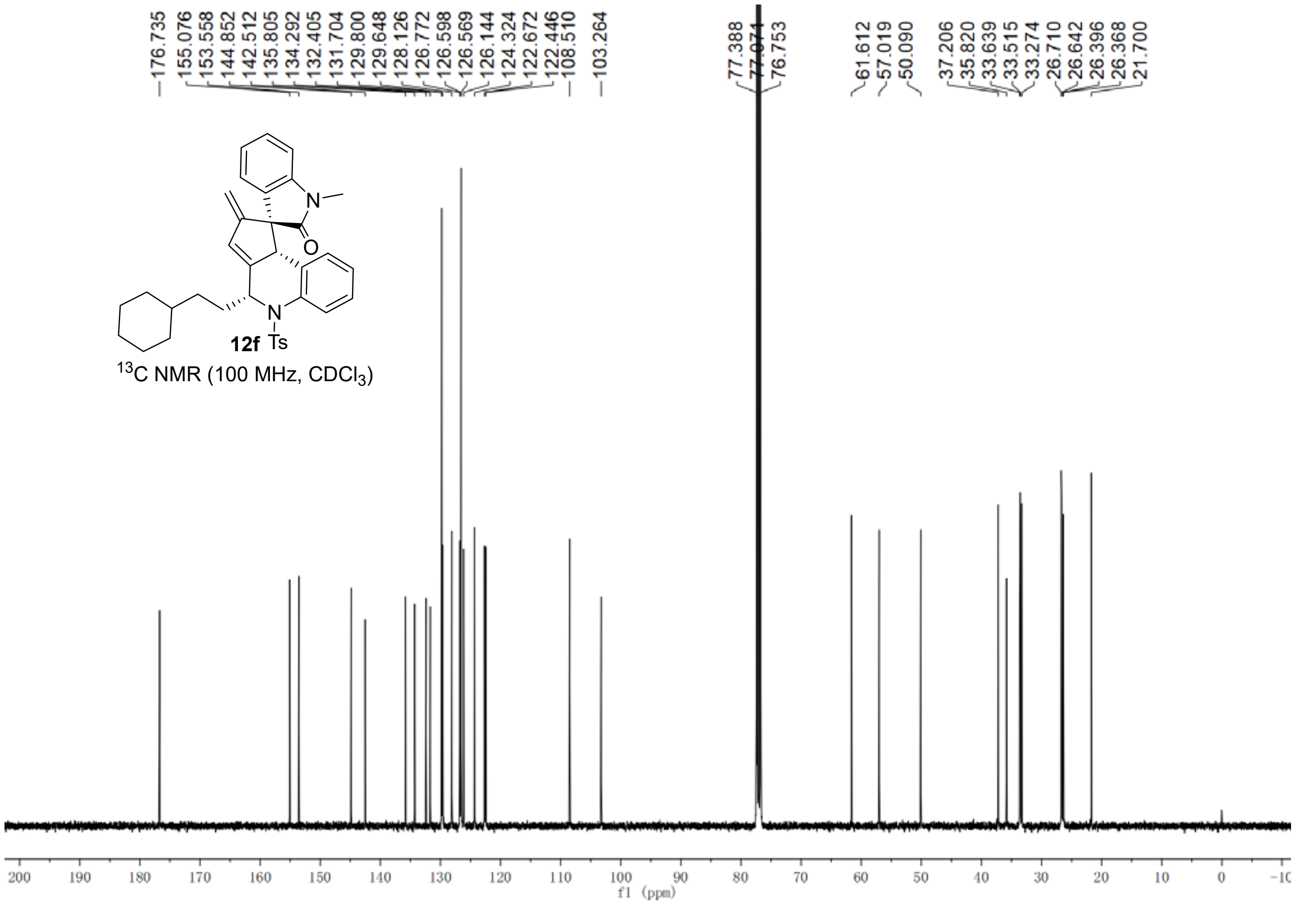
176.735 155.076 153.558 144.852 142.512 135.805 134.292 132.405 131.704 129.800 129.648 128.126 126.772 126.598 126.569 126.144 124.324 122.672 122.446 108.510 103.264 77.388 77.071 76.753 61.612 57.019 50.090 37.206 35.820 33.639 33.515 33.274 26.710 26.642 26.396 26.368 21.700
N
O
Ts
12f
^{13}C NMR (100 MHz, CDCl$_3$)
200 190 180 170 160 150 140 130 120 110 100 90 80 70 60 50 40 30 20 10 0 -10
f1 (ppm)

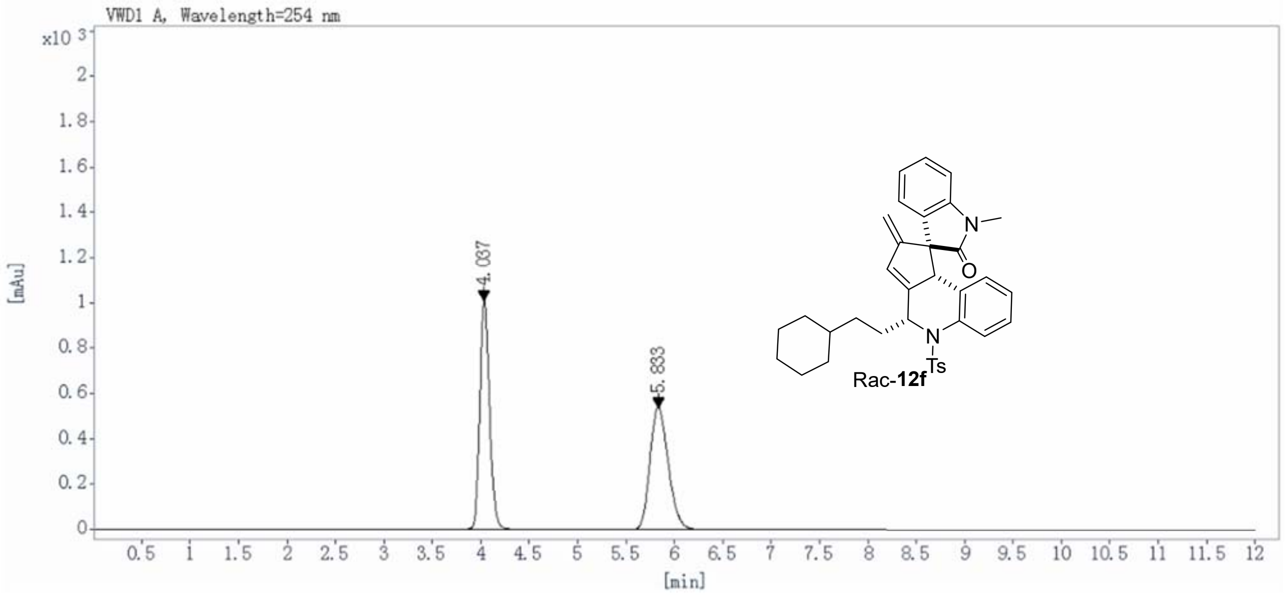

Ret Time [min]	Peak Type	Width [min]	Height [mAU]	Area [mAU*s]	Area [%]
4.037	BBA	0.11	1008.1496	7035.1875	49.8210
5.833	BBA	0.21	538.7615	7085.7324	50.1790
			Totals:	14120.9199	100.0000

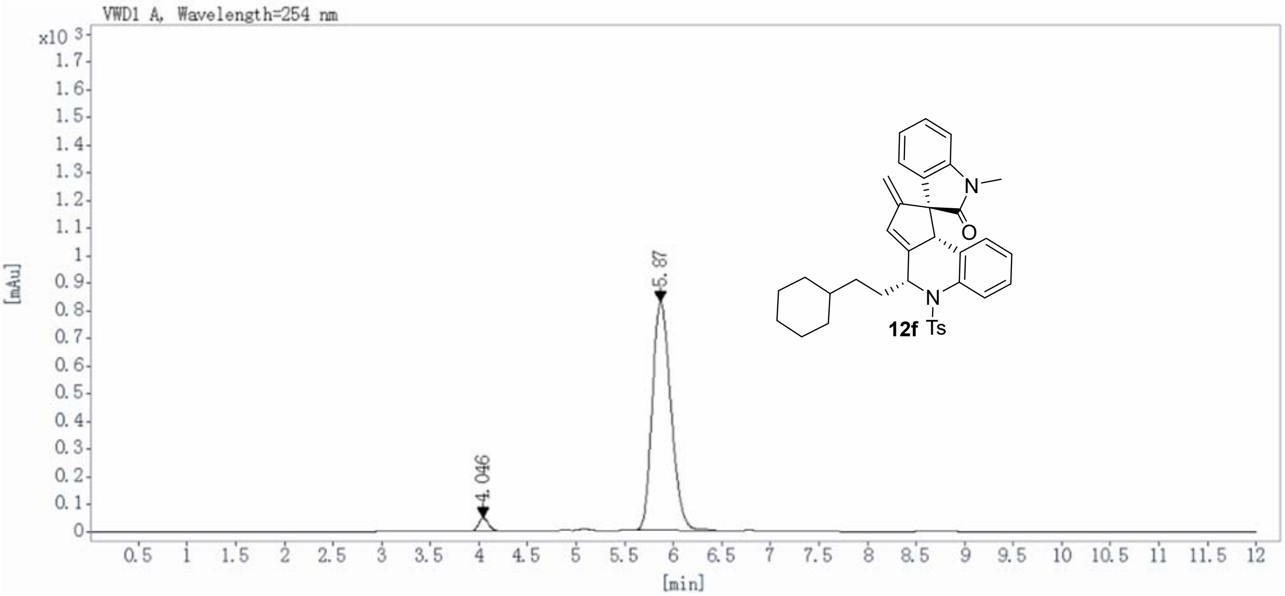

Ret Time [min]	Peak Type	Width [min]	Height [mAU]	Area [mAU*s]	Area [%]
4.046	BBA	0.11	48.3694	348.9897	3.0585
5.870	BBA	0.21	829.1635	11061.6025	96.9415
			Totals:	11410.5922	100.0000

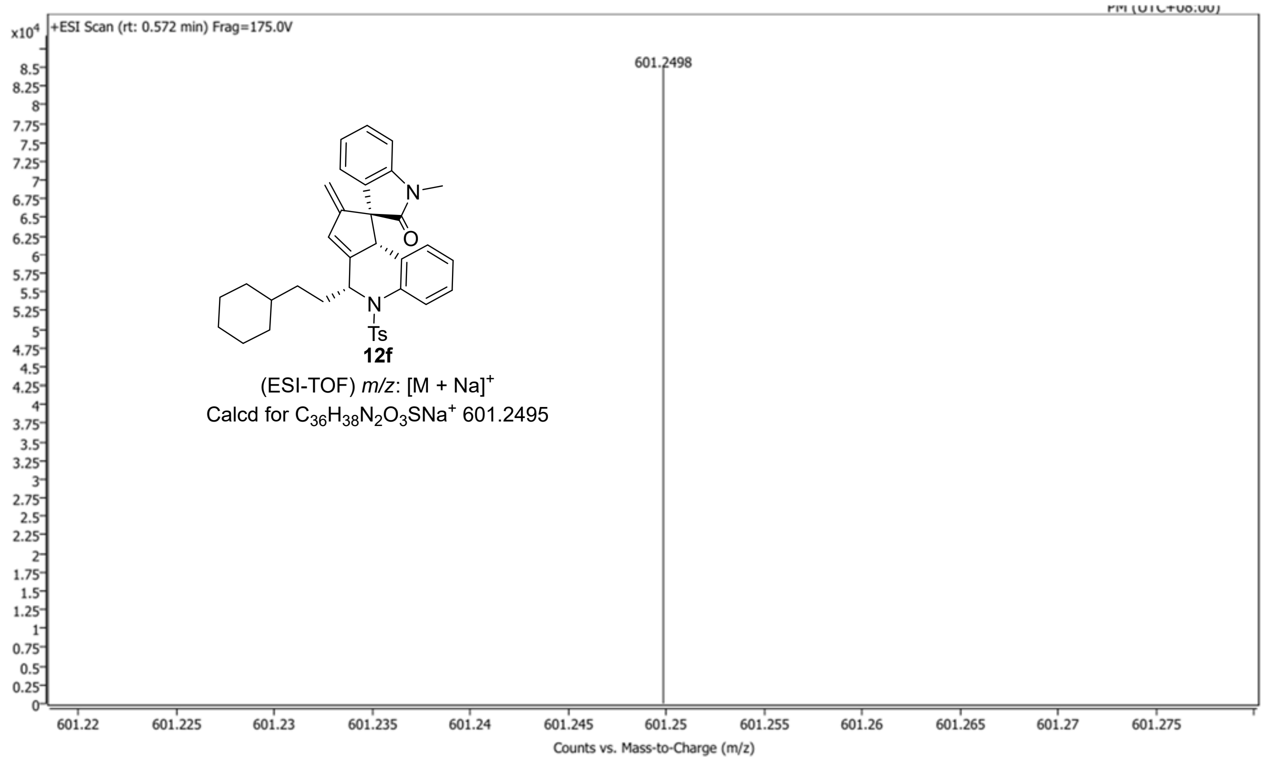
+ESI Scan (rt: 0.572 min) Frag=175.0V
x10^4
601.2498
12f
(ESI-TOF) m/z: [M + Na]+
Calcd for C36H38N2O3SNa+ 601.2495
Counts vs. Mass-to-Charge (m/z)

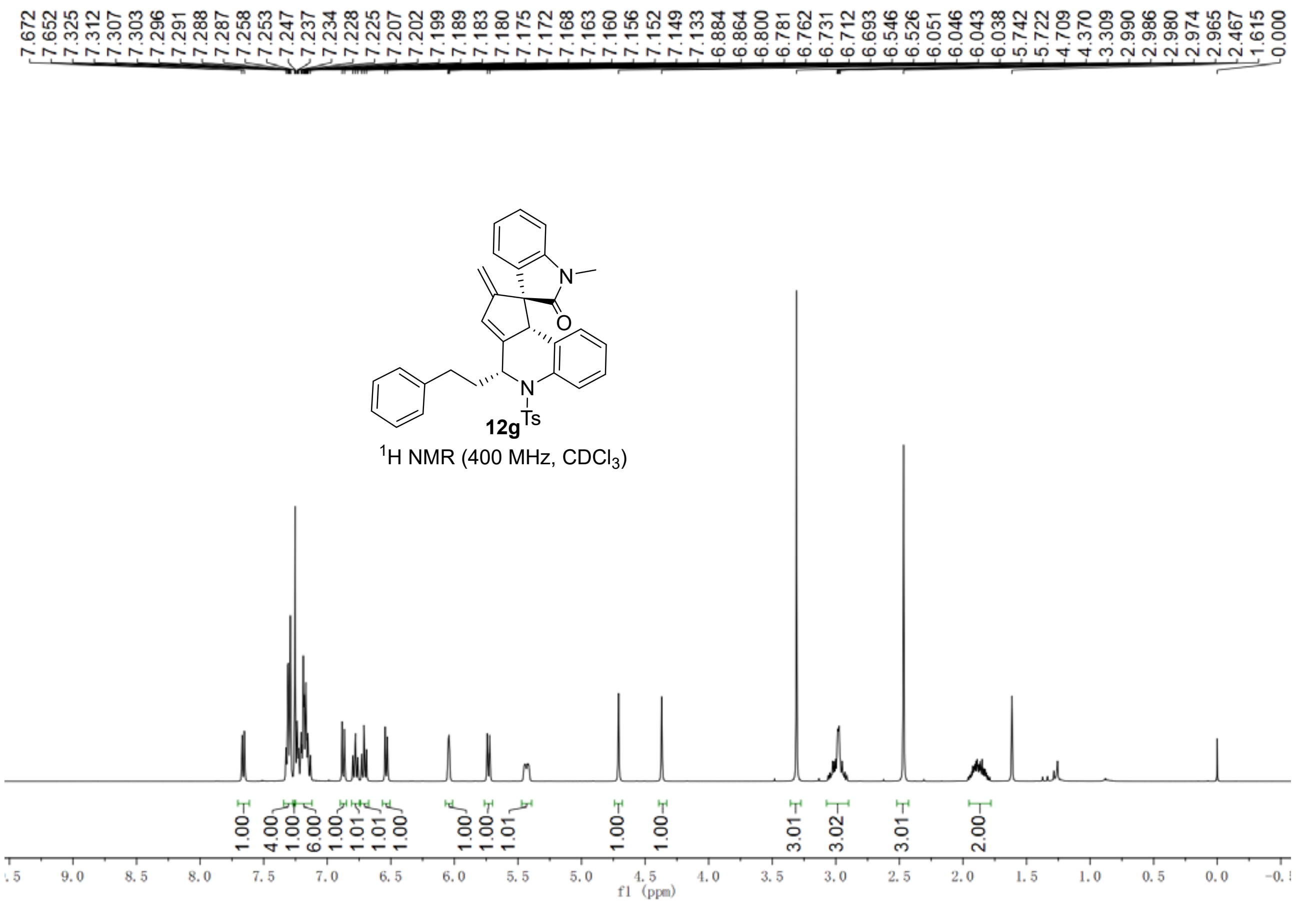
7.672
7.652
7.325
7.312
7.307
7.303
7.296
7.291
7.288
7.287
7.258
7.253
7.247
7.237
7.234
7.228
7.225
7.207
7.202
7.199
7.189
7.183
7.180
7.175
7.172
7.168
7.163
7.160
7.156
7.152
7.149
7.133
6.884
6.864
6.800
6.781
6.762
6.731
6.712
6.693
6.546
6.526
6.051
6.046
6.043
6.038
5.742
5.722
4.709
4.370
3.309
2.990
2.986
2.980
2.974
2.965
2.467
1.615
0.000
N
O
N
Ts
12g
^{1}H NMR (400 MHz, $CDCl_3$)
1.00
4.00
1.00
6.00
1.00
1.01
1.01
1.00
1.00
1.00
1.01
1.00
1.00
3.01
3.02
3.01
2.00
9.0 8.5 8.0 7.5 7.0 6.5 6.0 5.5 5.0 4.5 4.0 3.5 3.0 2.5 2.0 1.5 1.0 0.5 0.0
f1 (ppm)

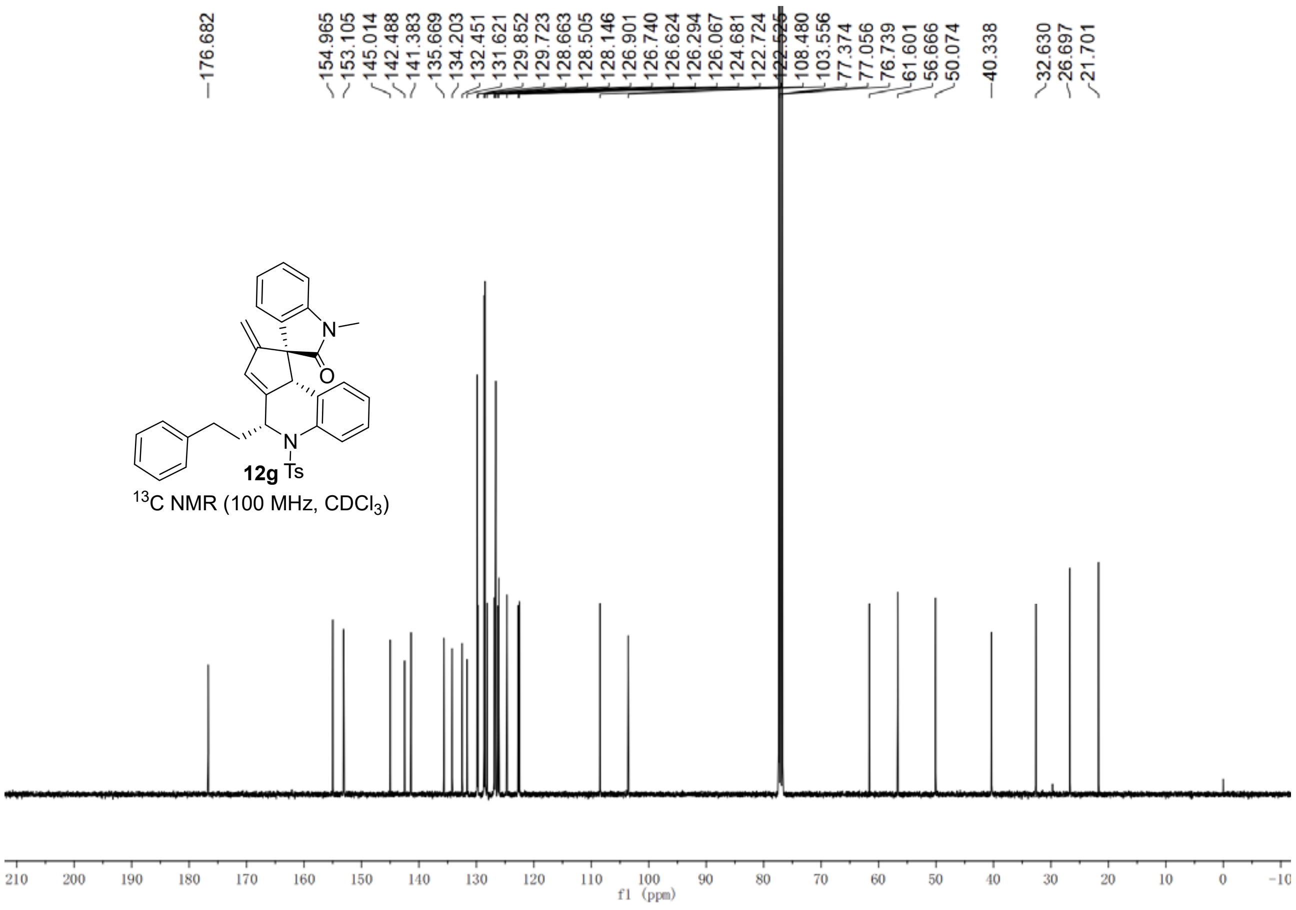
176.682
154.965
153.105
145.014
142.488
141.383
135.669
134.203
132.451
131.621
129.852
129.723
128.663
128.505
128.146
126.901
126.740
126.624
126.294
126.067
124.681
122.724
122.525
108.480
103.556
77.374
77.056
76.739
61.601
56.666
50.074
40.338
32.630
26.697
21.701
N
O
N
Ts
12g
^{13}C NMR (100 MHz, $CDCl_3$)
210 200 190 180 170 160 150 140 130 120 110 100 90 80 70 60 50 40 30 20 10 0 -10
f1 (ppm)

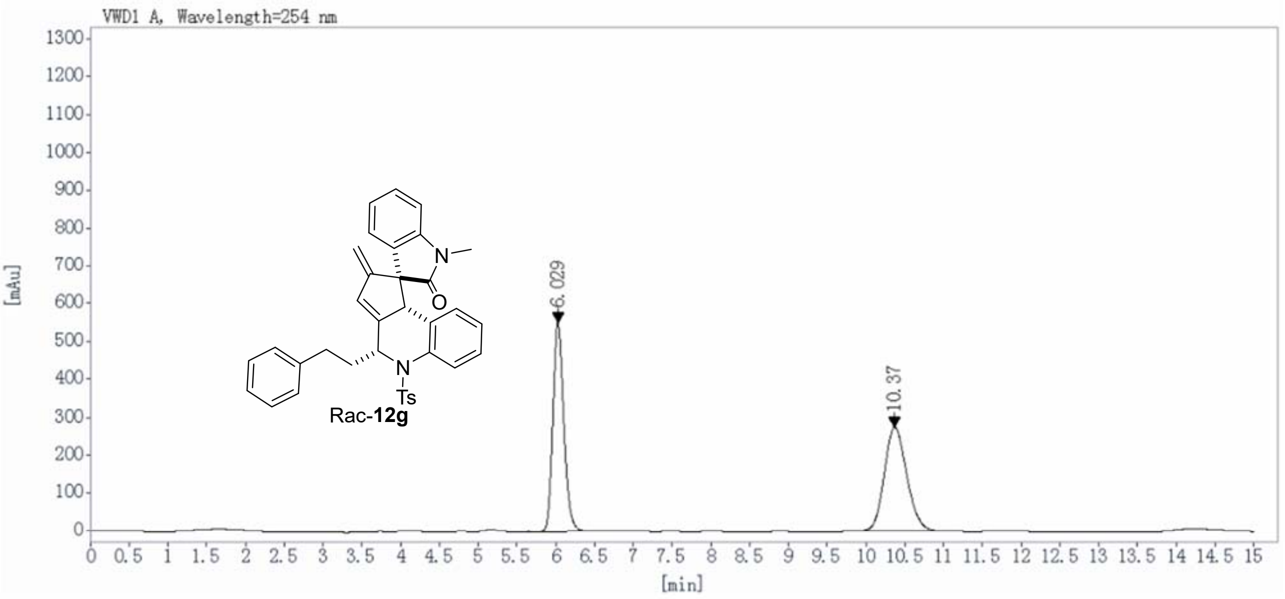

Ret Time [min]	Peak Type	Width [min]	Height [mAU]	Area [mAU*s]	Area [%]
6.029	BBA	0.15	551.4871	5511.3047	49.7055
10.370	BBA	0.31	276.2112	5576.6040	50.2945
			Totals:	11087.9087	100.0000

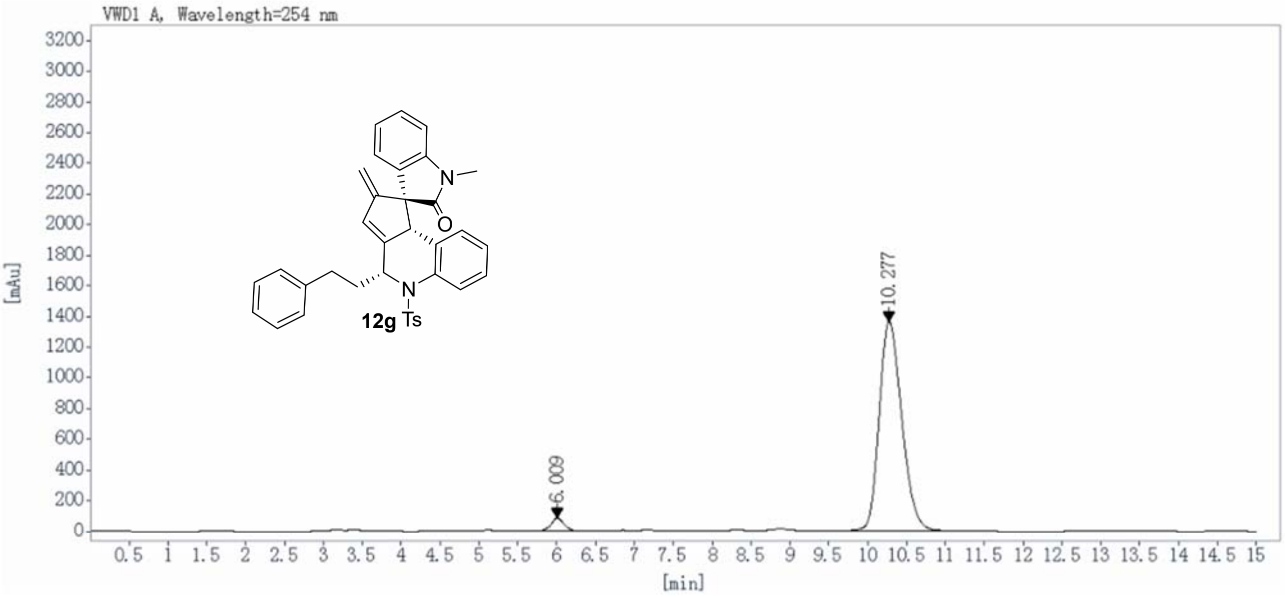

Ret Time [min]	Peak Type	Width [min]	Height [mAU]	Area [mAU*s]	Area [%]
6.009	BBA	0.17	85.6434	1001.8586	3.5056
10.277	BBA	0.31	1364.2518	27576.8809	96.4944
			Totals:	28578.7395	100.0000

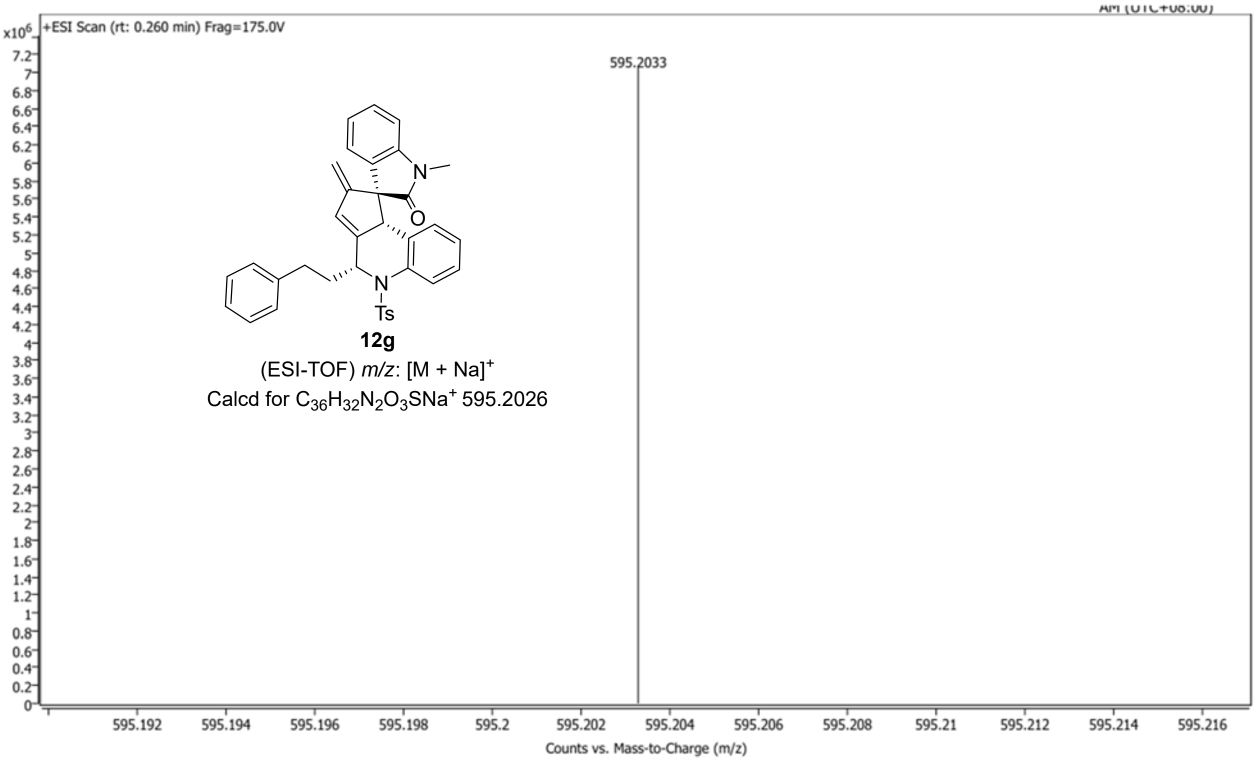
AM (UTC+08:00)
x10^6
+ESI Scan (rt: 0.260 min) Frag=175.0V
595.2033
12g
(ESI-TOF) m/z: [M + Na]+
Calcd for C36H32N2O3SNa+ 595.2026
Counts vs. Mass-to-Charge (m/z)

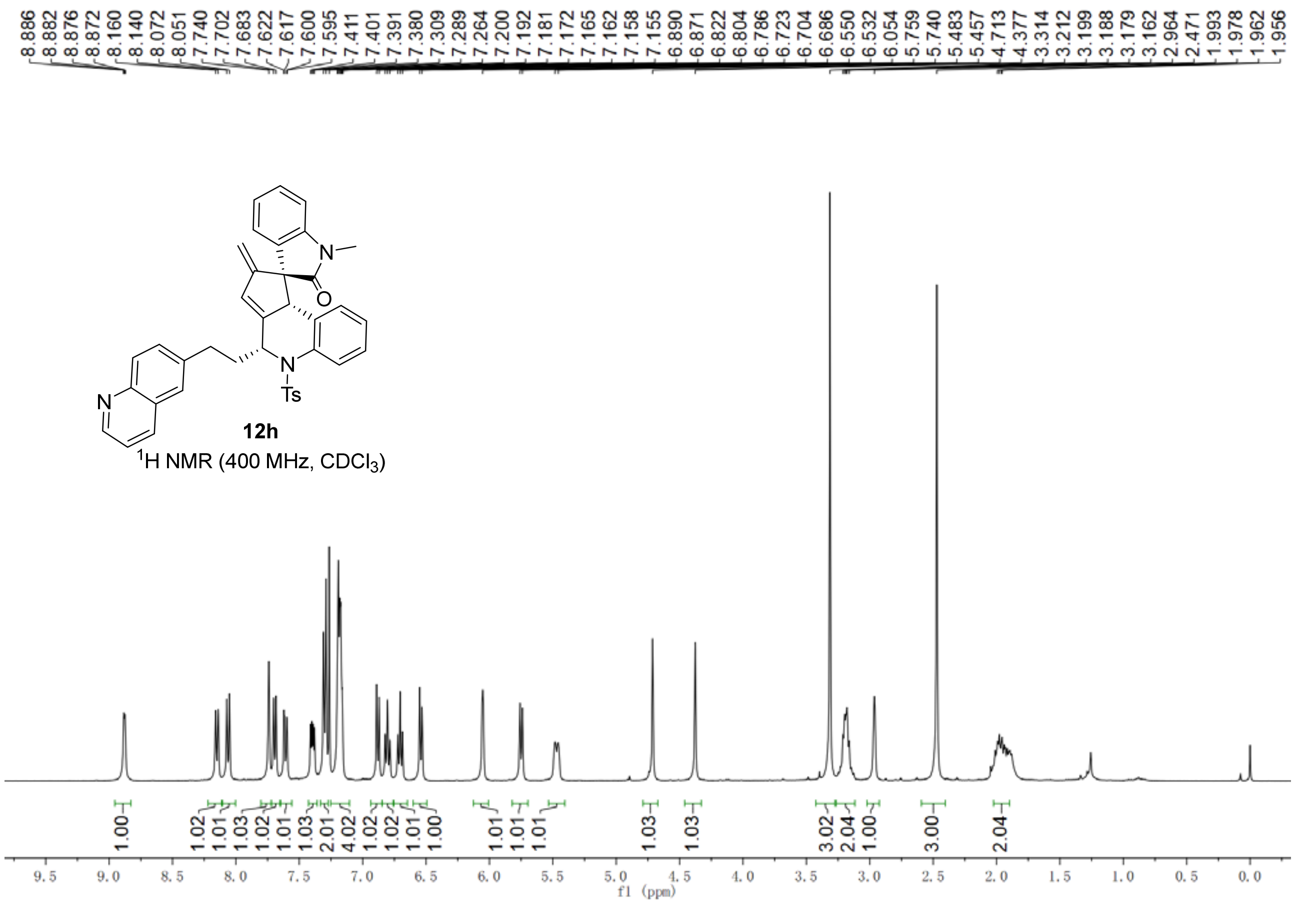
8.886
8.882
8.876
8.872
8.160
8.140
8.072
8.051
7.740
7.702
7.683
7.622
7.617
7.600
7.595
7.411
7.401
7.391
7.380
7.309
7.289
7.264
7.200
7.192
7.181
7.172
7.165
7.162
7.158
7.155
6.890
6.871
6.822
6.804
6.786
6.723
6.704
6.686
6.550
6.532
6.054
5.759
5.740
5.483
5.457
4.713
4.377
3.314
3.212
3.199
3.188
3.179
3.162
2.964
2.471
1.993
1.978
1.962
1.956
N
O
N
Ts
N
12h
^{1}H NMR (400 MHz, CDCl$_3$)
1.00
1.02
1.01
1.03
1.02
1.01
1.03
2.01
4.02
1.02
1.02
1.01
1.00
1.01
1.01
1.01
1.03
1.03
3.02
2.04
1.00
3.00
2.04
f1 (ppm)

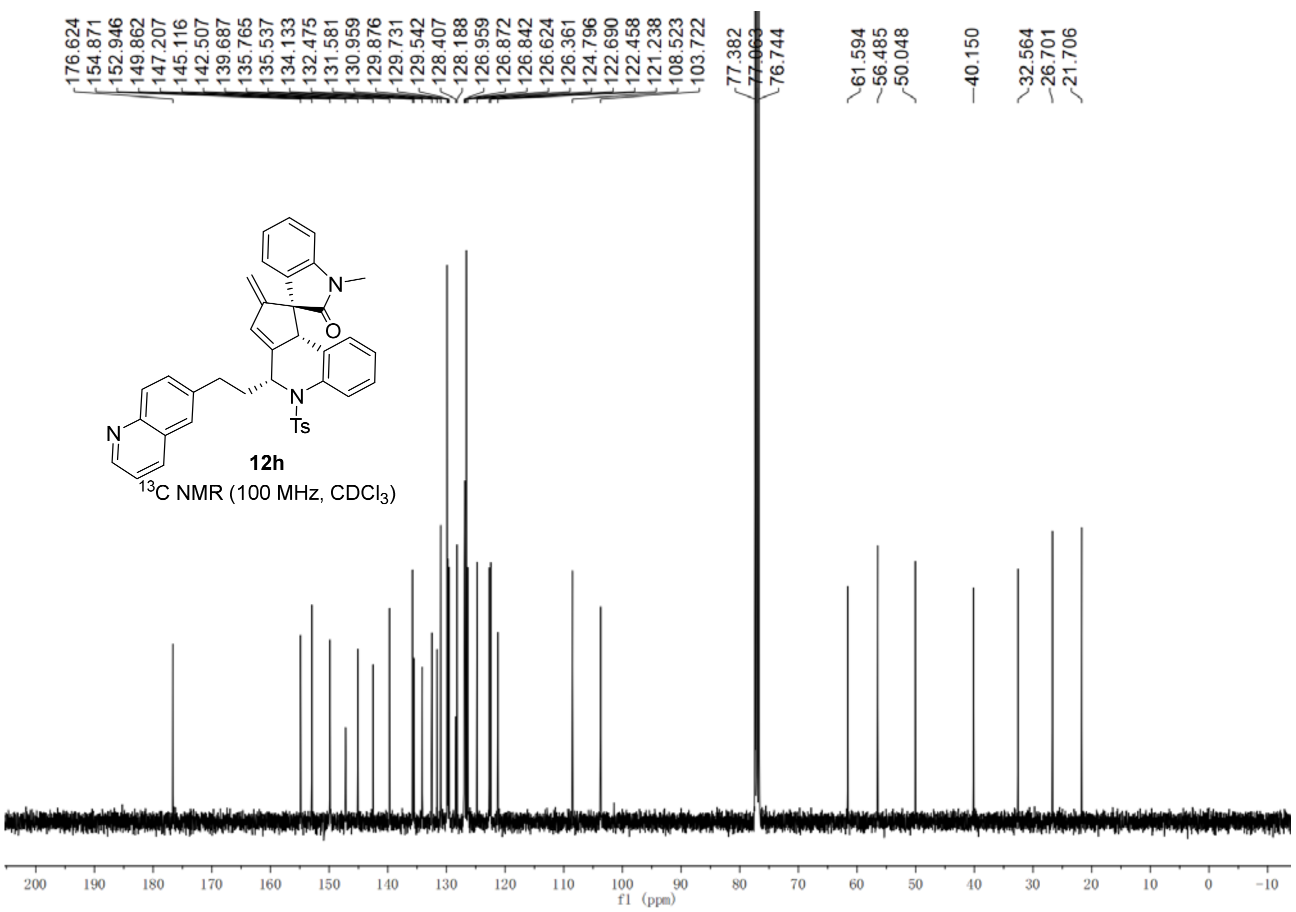
176.624
154.871
152.946
149.862
147.207
145.116
142.507
139.687
135.765
135.537
134.133
132.475
131.581
130.959
129.876
129.731
129.542
128.407
128.188
126.959
126.872
126.842
126.624
126.361
124.796
122.690
122.458
121.238
108.523
103.722
77.382
77.063
76.744
61.594
56.485
50.048
40.150
32.564
26.701
21.706
N
O
N
Ts
N
12h
^{13}C NMR (100 MHz, CDCl$_3$)
f1 (ppm)

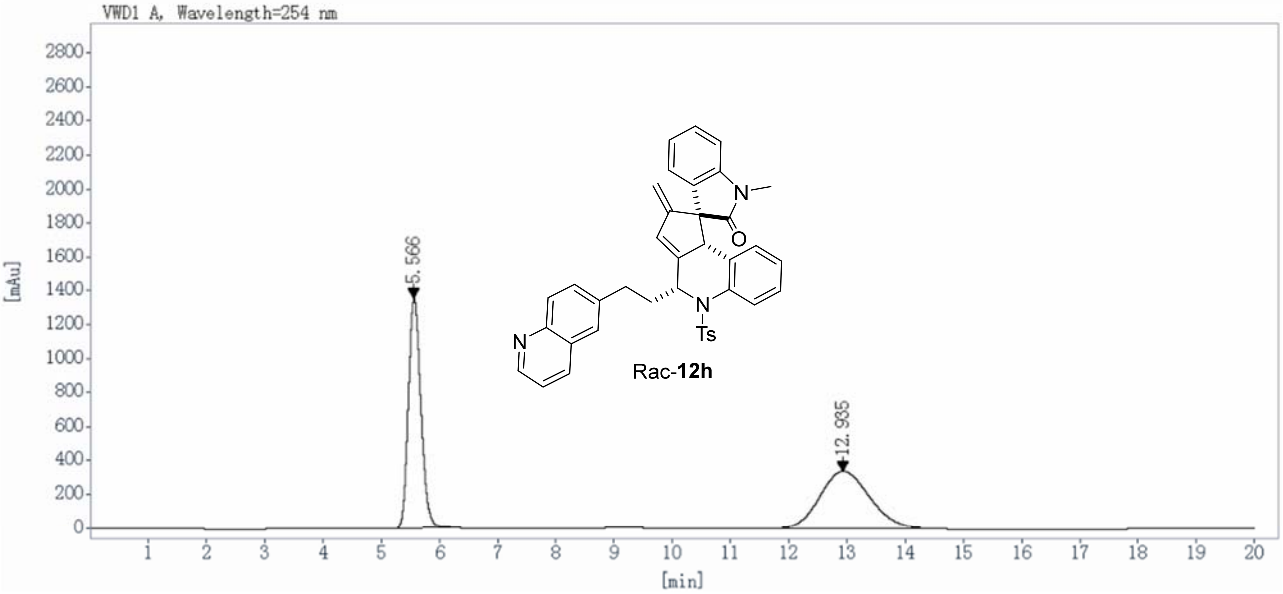

Ret Time [min]	Peak Type	Width [min]	Height [mAU]	Area [mAU*s]	Area [%]
5.566	BBA	0.23	1352.6578	20139.9414	50.0449
12.935	BBA	0.93	338.6057	20103.8398	49.9551
			Totals:	40243.7813	100.0000

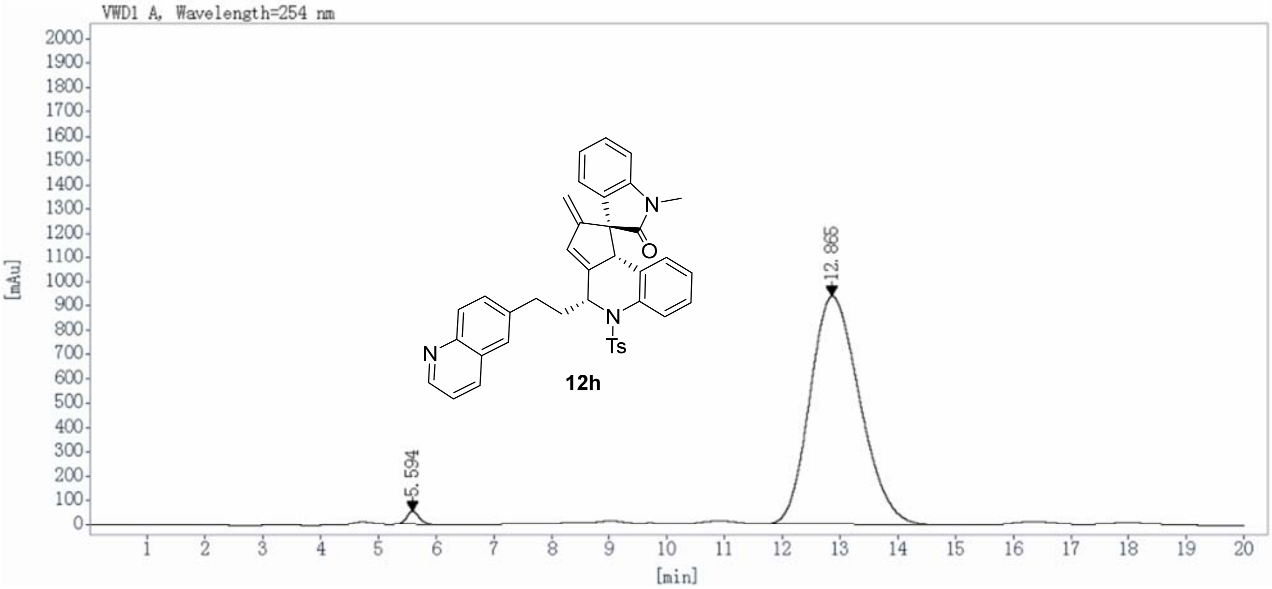

Ret Time [min]	Peak Type	Width [min]	Height [mAU]	Area [mAU*s]	Area [%]
5.594	BBA	0.24	53.7615	821.4728	1.4394
12.865	BBA	0.94	937.7807	56249.4727	98.5606
			Totals:	57070.9454	100.0000

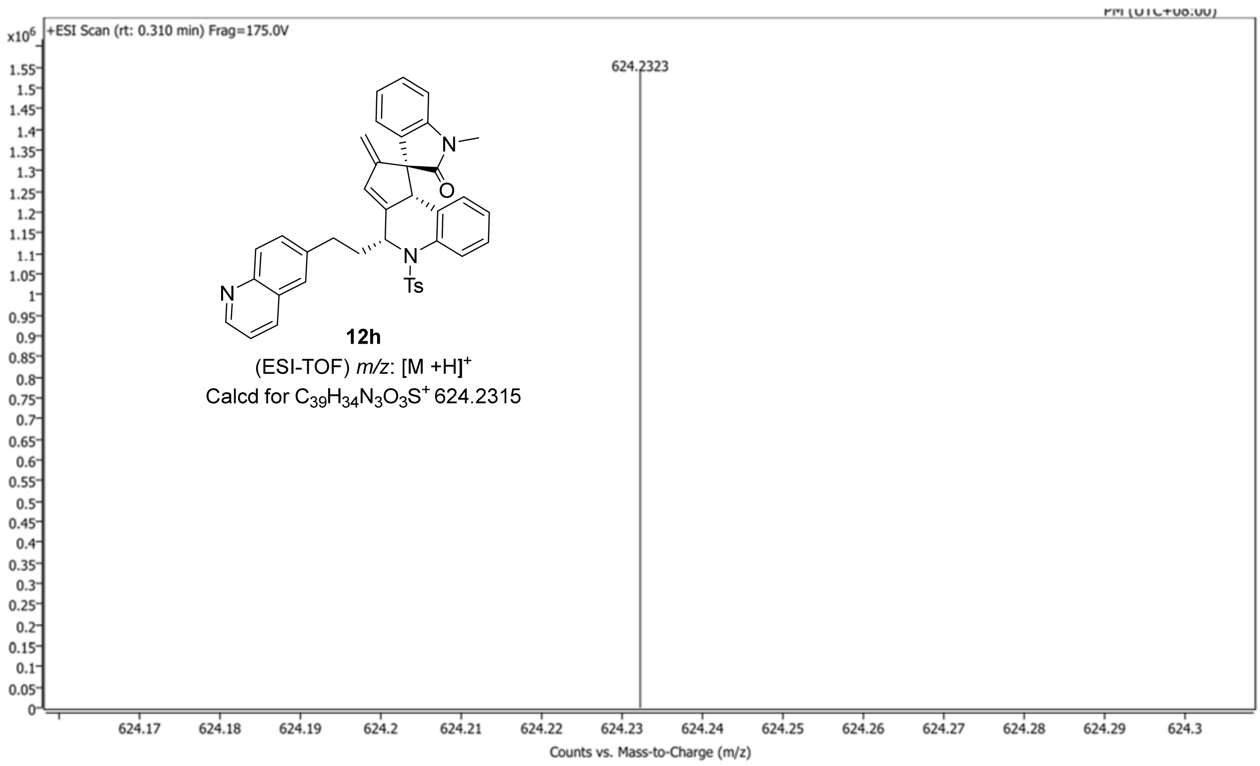
PM (UTC+08:00)
x10^6
+ESI Scan (rt: 0.310 min) Frag=175.0V
624.2323
N
O
N
Ts
N
12h
(ESI-TOF) m/z: [M +H]+
Calcd for C39H34N3O3S+ 624.2315
1.55
1.5
1.45
1.4
1.35
1.3
1.25
1.2
1.15
1.1
1.05
1
0.95
0.9
0.85
0.8
0.75
0.7
0.65
0.6
0.55
0.5
0.45
0.4
0.35
0.3
0.25
0.2
0.15
0.1
0.05
0
624.17
624.18
624.19
624.2
624.21
624.22
624.23
624.24
624.25
624.26
624.27
624.28
624.29
624.3
Counts vs. Mass-to-Charge (m/z)

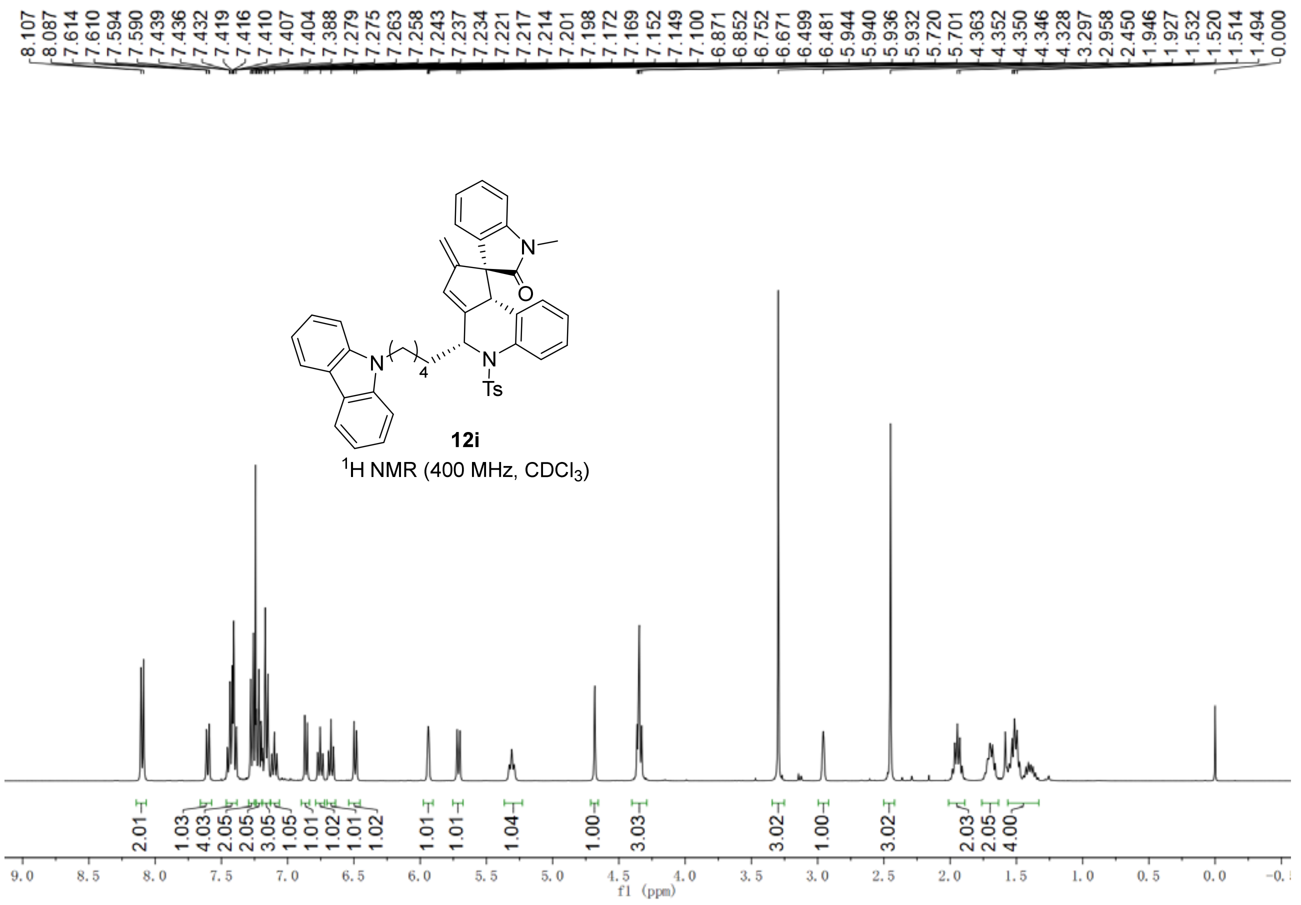
8.107
8.087
7.614
7.610
7.594
7.590
7.439
7.436
7.432
7.419
7.416
7.410
7.407
7.404
7.388
7.279
7.275
7.263
7.258
7.243
7.237
7.234
7.221
7.217
7.214
7.201
7.198
7.172
7.169
7.152
7.149
7.100
6.871
6.852
6.752
6.671
6.499
6.481
5.944
5.940
5.936
5.932
5.720
5.701
4.363
4.352
4.350
4.346
4.328
3.297
2.958
2.450
1.946
1.927
1.532
1.520
1.514
1.494
0.000
N
O
N
Ts
N
4
12i
1H NMR (400 MHz, CDCl3)
2.01
1.03
4.03
2.05
2.05
3.05
1.05
1.01
1.02
1.01
1.02
1.01
1.01
1.04
1.00
3.03
3.02
1.00
3.02
2.03
2.05
4.00
f1 (ppm)

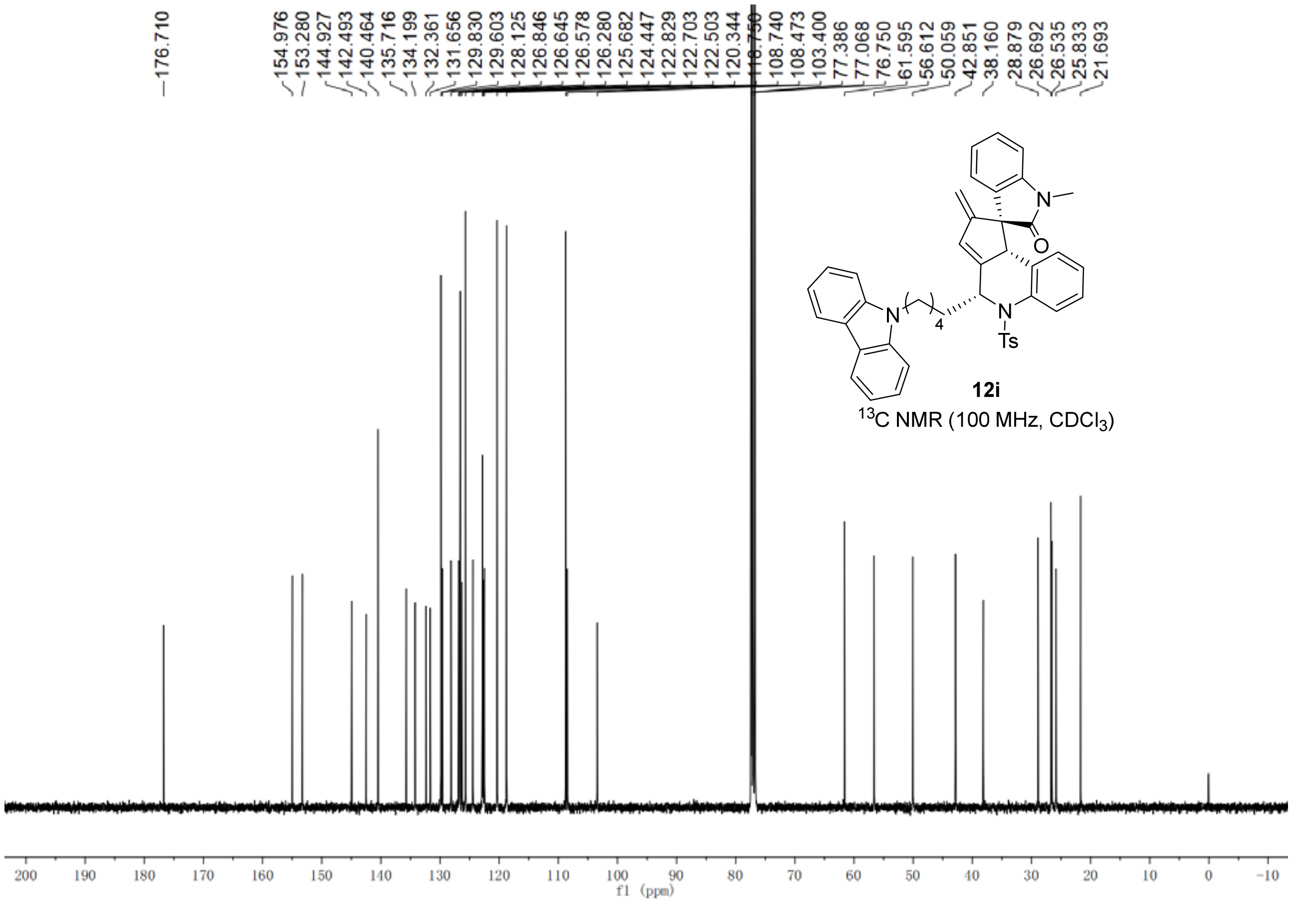
176.710
154.976
153.280
144.927
142.493
140.464
135.716
134.199
132.361
131.656
129.830
129.603
128.125
126.846
126.645
126.578
126.280
125.682
124.447
122.829
122.703
122.503
120.344
108.740
108.473
103.400
77.386
77.068
76.750
61.595
56.612
50.059
42.851
38.160
28.879
26.692
26.535
25.833
21.693
N
O
N
Ts
N
4
12i
13C NMR (100 MHz, CDCl3)
f1 (ppm)

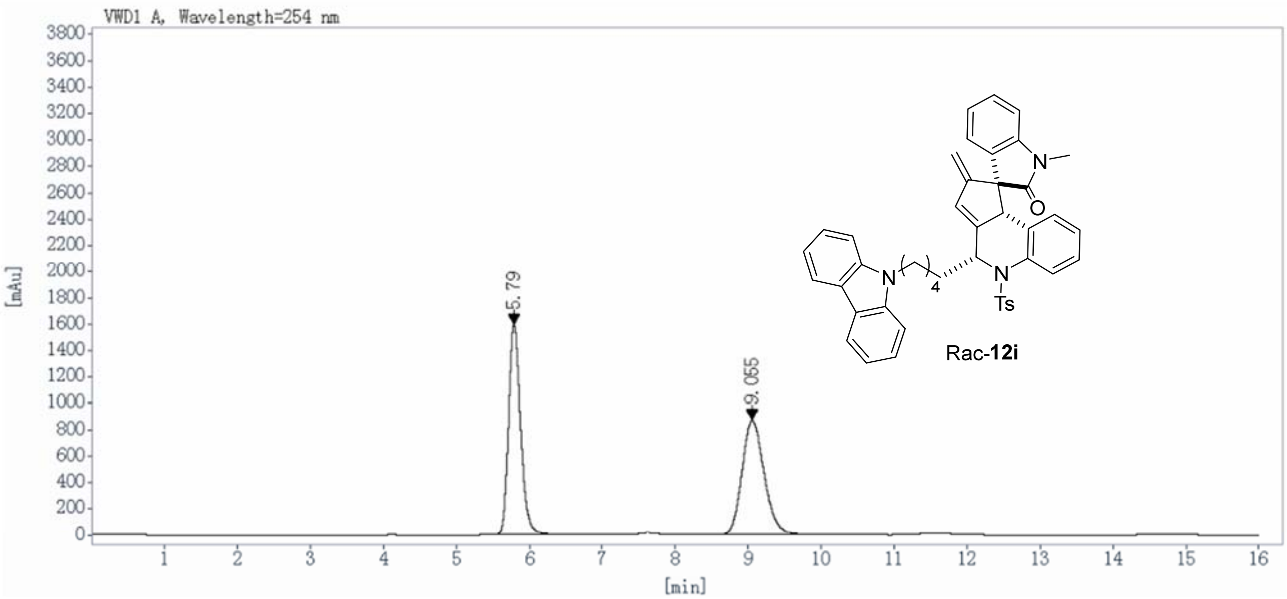

Ret Time [min]	Peak Type	Width [min]	Height [mAU]	Area [mAU*s]	Area [%]
5.790	BBA	0.18	1591.1262	18135.9199	49.7910
9.055	BBA	0.33	869.1802	18288.1602	50.2090
			Totals:	36424.0801	100.0000

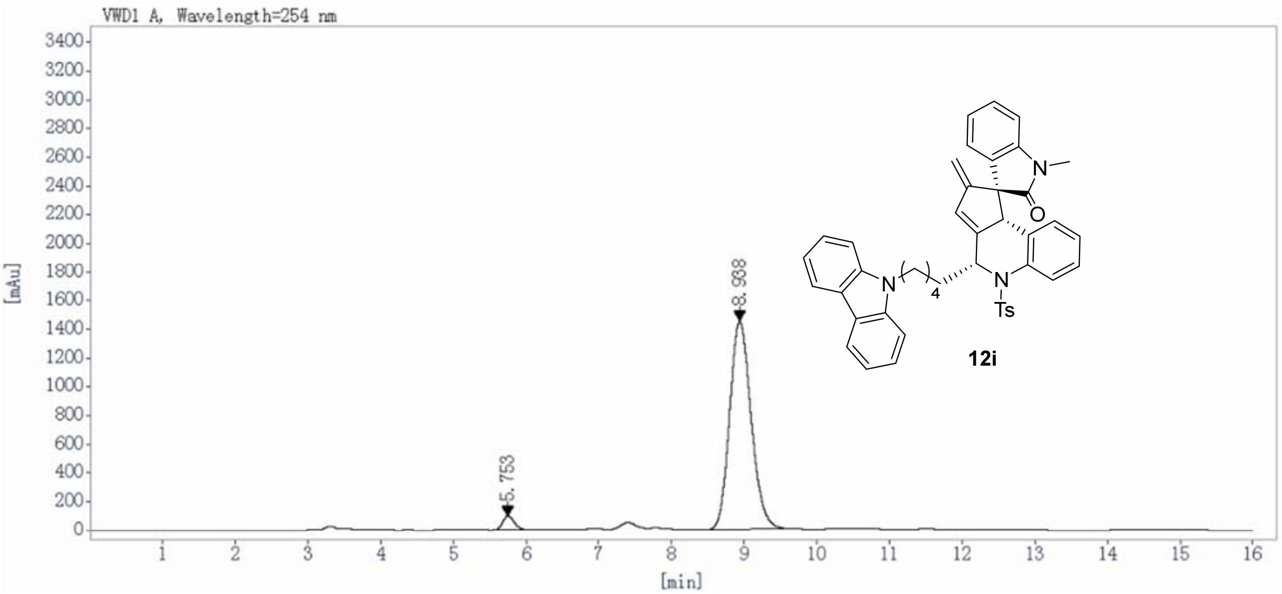

Ret Time [min]	Peak Type	Width [min]	Height [mAU]	Area [mAU*s]	Area [%]
5.753	BBA	0.17	99.4315	1099.3254	3.5334
8.938	BBA	0.32	1450.0912	30013.2988	96.4666
			Totals:	31112.6243	100.0000

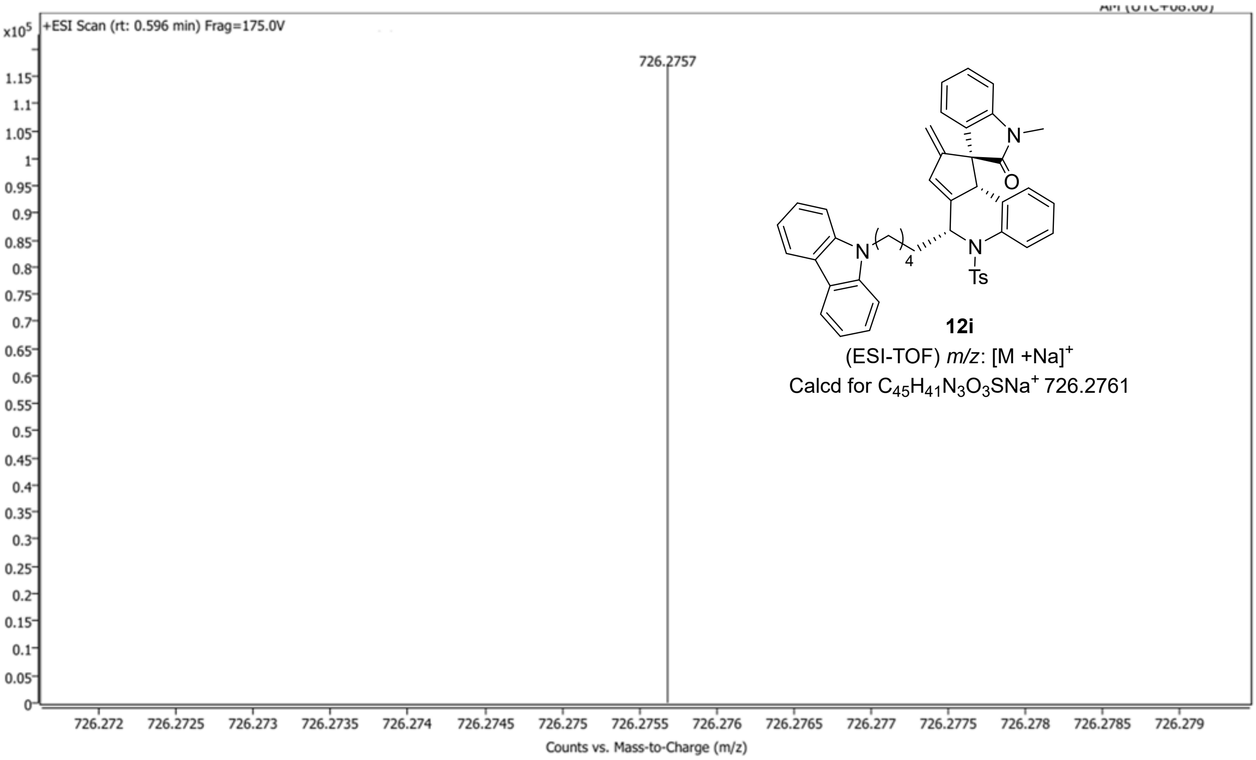

12i

(ESI-TOF) *m/z*: [M +Na]$^+$

Calcd for $C_{45}H_{41}N_3O_3SNa^+$ 726.2761

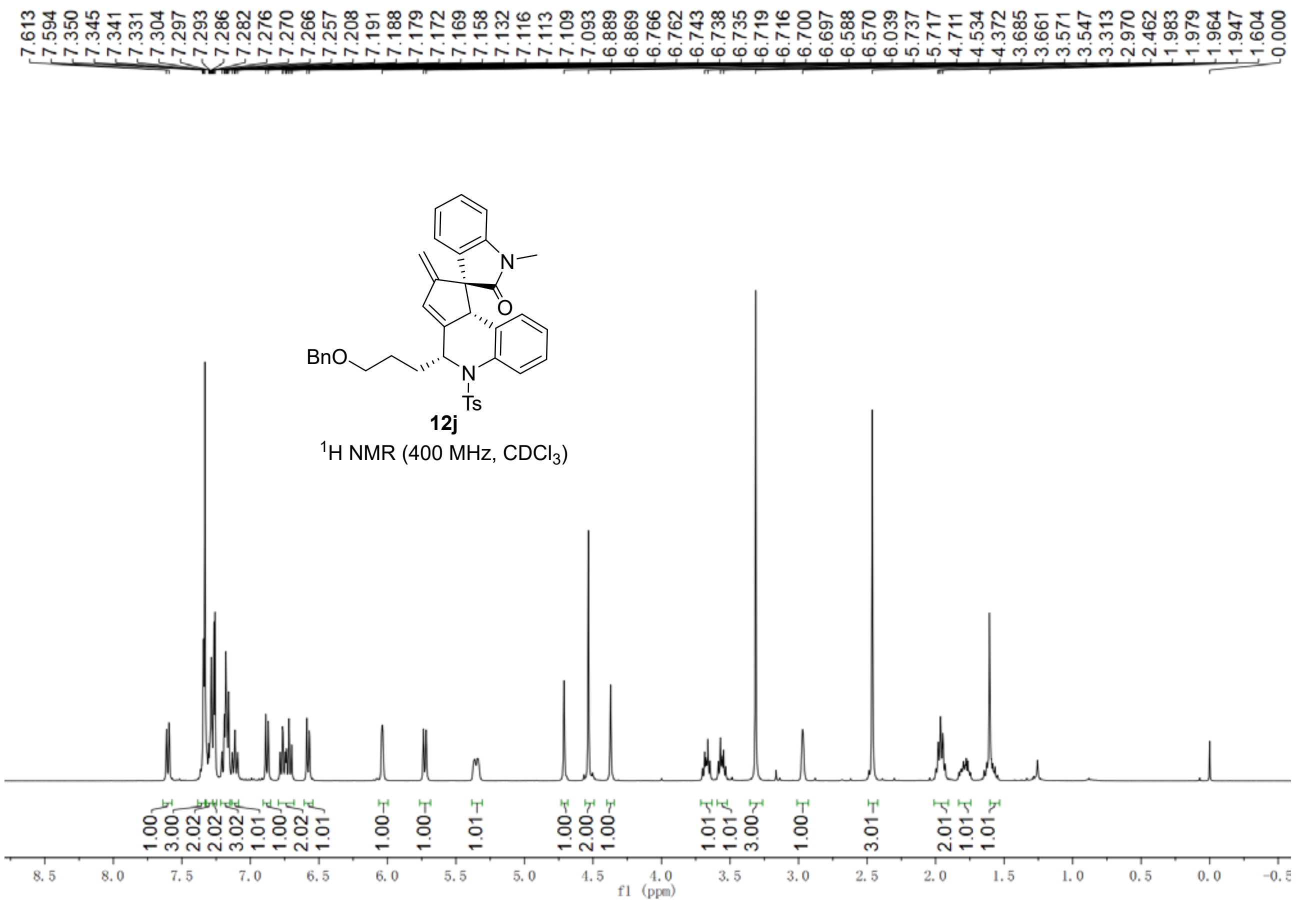
7.613 7.594 7.350 7.345 7.341 7.331 7.304 7.297 7.293 7.286 7.282 7.276 7.270 7.266 7.257 7.208 7.191 7.188 7.179 7.172 7.169 7.158 7.132 7.116 7.113 7.109 7.093 6.889 6.869 6.766 6.762 6.743 6.738 6.735 6.719 6.716 6.700 6.697 6.588 6.570 6.039 5.737 5.717 4.711 4.534 4.372 3.685 3.661 3.571 3.547 3.313 2.970 2.462 1.983 1.979 1.964 1.947 1.604 0.000
BnO
N
O
N
Ts
12j
^{1}H NMR (400 MHz, CDCl$_3$)
1.00 3.00 2.02 2.02 3.02 1.01 1.00 2.02 1.01 1.00 1.00 1.01 1.00 2.00 1.00 1.01 1.01 3.00 1.00 3.01 2.01 1.01 1.01
8.5 8.0 7.5 7.0 6.5 6.0 5.5 5.0 4.5 4.0 3.5 3.0 2.5 2.0 1.5 1.0 0.5 0.0 -0.5
f1 (ppm)

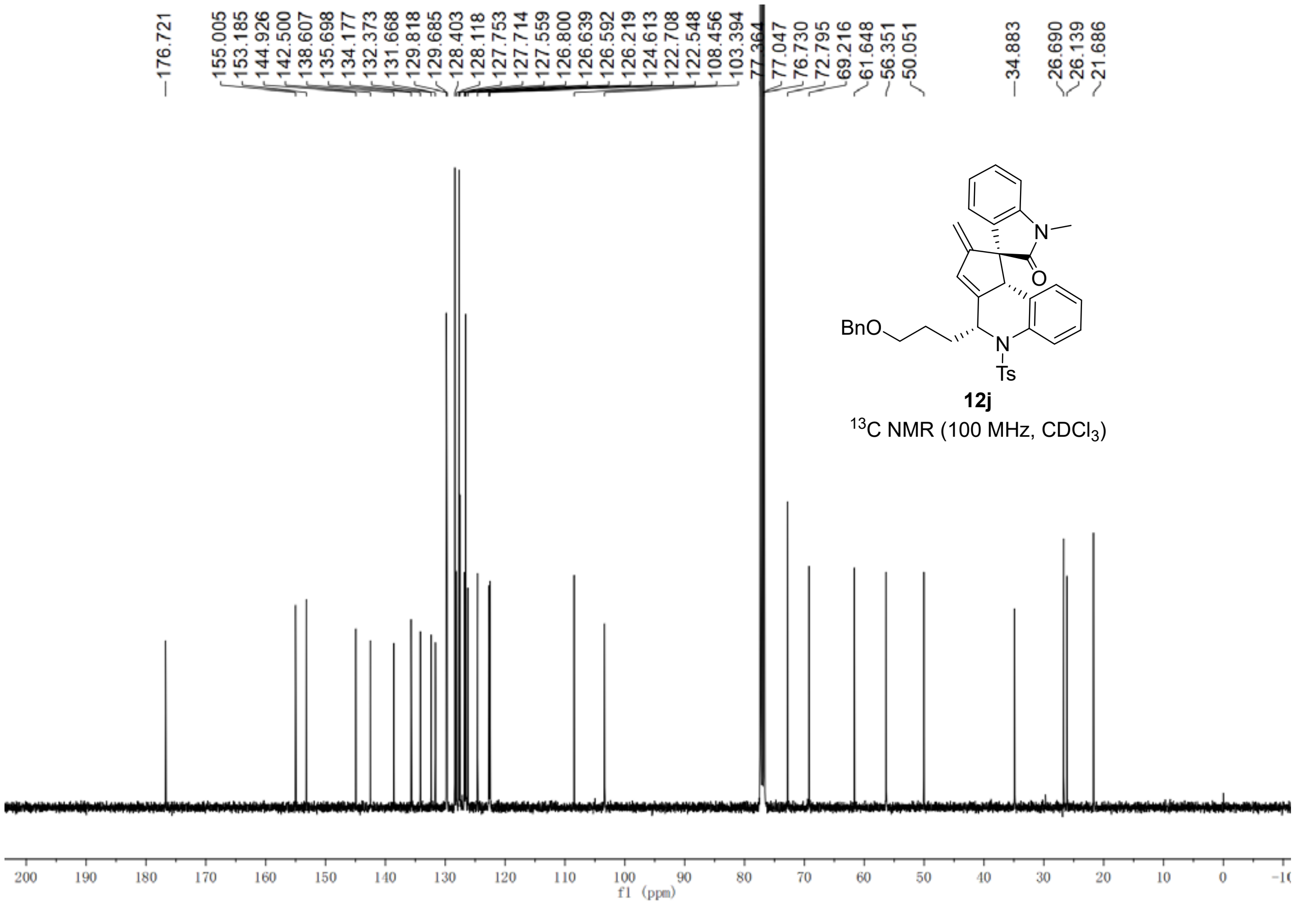
176.721 155.005 153.185 144.926 142.500 138.607 135.698 134.177 132.373 131.668 129.818 129.685 128.403 128.118 127.753 127.714 127.559 126.800 126.639 126.592 126.219 124.613 122.708 122.548 108.456 103.394 77.364 77.047 76.730 72.795 69.216 61.648 56.351 50.051 34.883 26.690 26.139 21.686
BnO
N
O
N
Ts
12j
^{13}C NMR (100 MHz, CDCl$_3$)
200 190 180 170 160 150 140 130 120 110 100 90 80 70 60 50 40 30 20 10 0 -10
f1 (ppm)

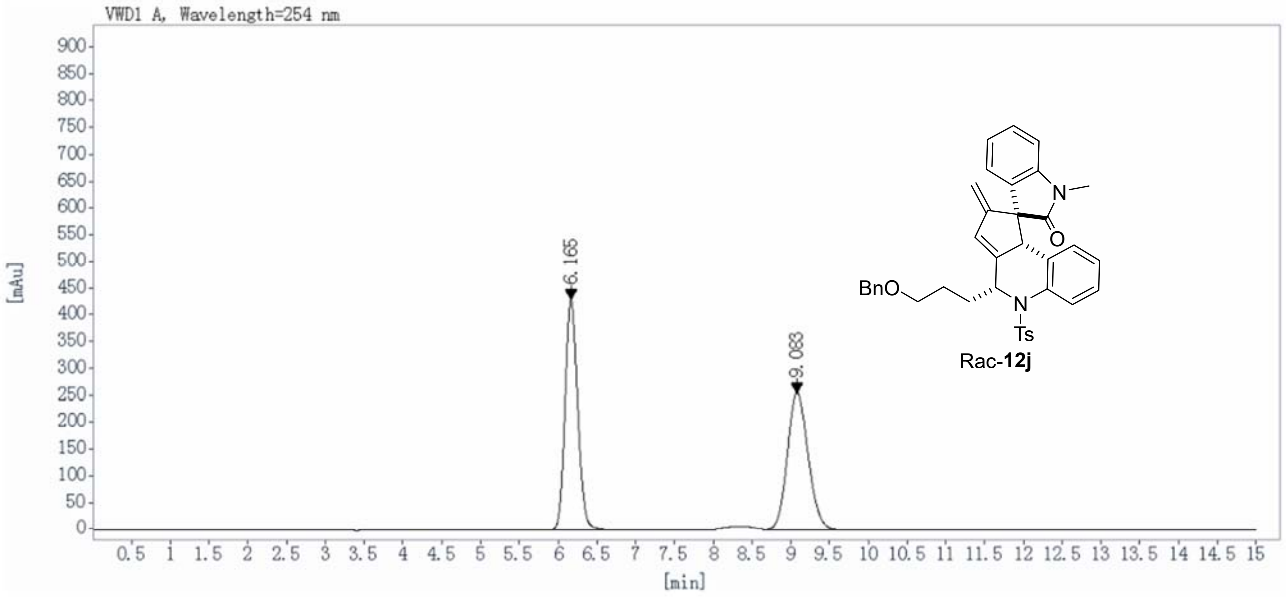

Ret Time [min]	Peak Type	Width [min]	Height [mAU]	Area [mAU*s]	Area [%]
6.165	BBA	0.17	428.2721	4798.7588	50.1795
9.083	BBA	0.29	254.8855	4764.4277	49.8205
			Totals:	9563.1865	100.0000

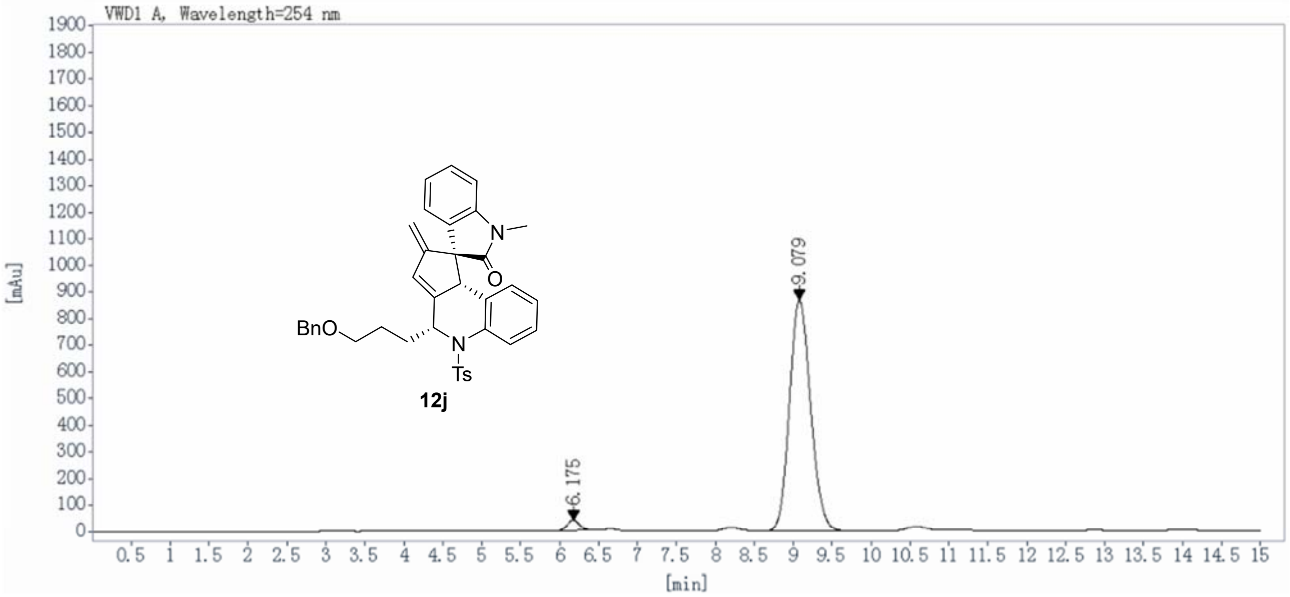

Ret Time [min]	Peak Type	Width [min]	Height [mAU]	Area [mAU*s]	Area [%]
6.175	BB	0.17	39.0287	438.8946	2.6057
9.079	BBA	0.30	865.3759	16404.4570	97.3943
			Totals:	16843.3516	100.0000

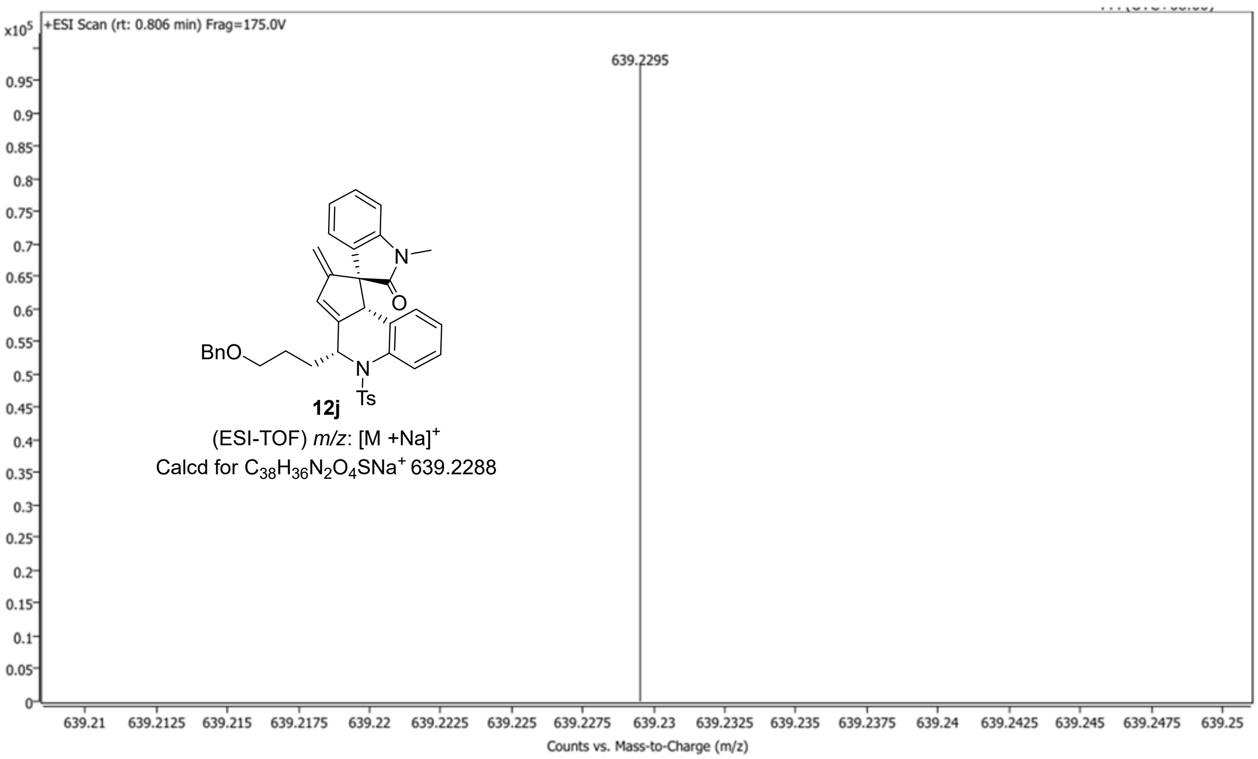

x10^5
+ESI Scan (rt: 0.806 min) Frag=175.0V
639.2295
BnO
N
O
N
Ts
12j
(ESI-TOF) m/z: [M +Na]+
Calcd for C38H36N2O4SNa+ 639.2288
0.95
0.9
0.85
0.8
0.75
0.7
0.65
0.6
0.55
0.5
0.45
0.4
0.35
0.3
0.25
0.2
0.15
0.1
0.05
0
639.21
639.2125
639.215
639.2175
639.22
639.2225
639.225
639.2275
639.23
639.2325
639.235
639.2375
639.24
639.2425
639.245
639.2475
639.25
Counts vs. Mass-to-Charge (m/z)

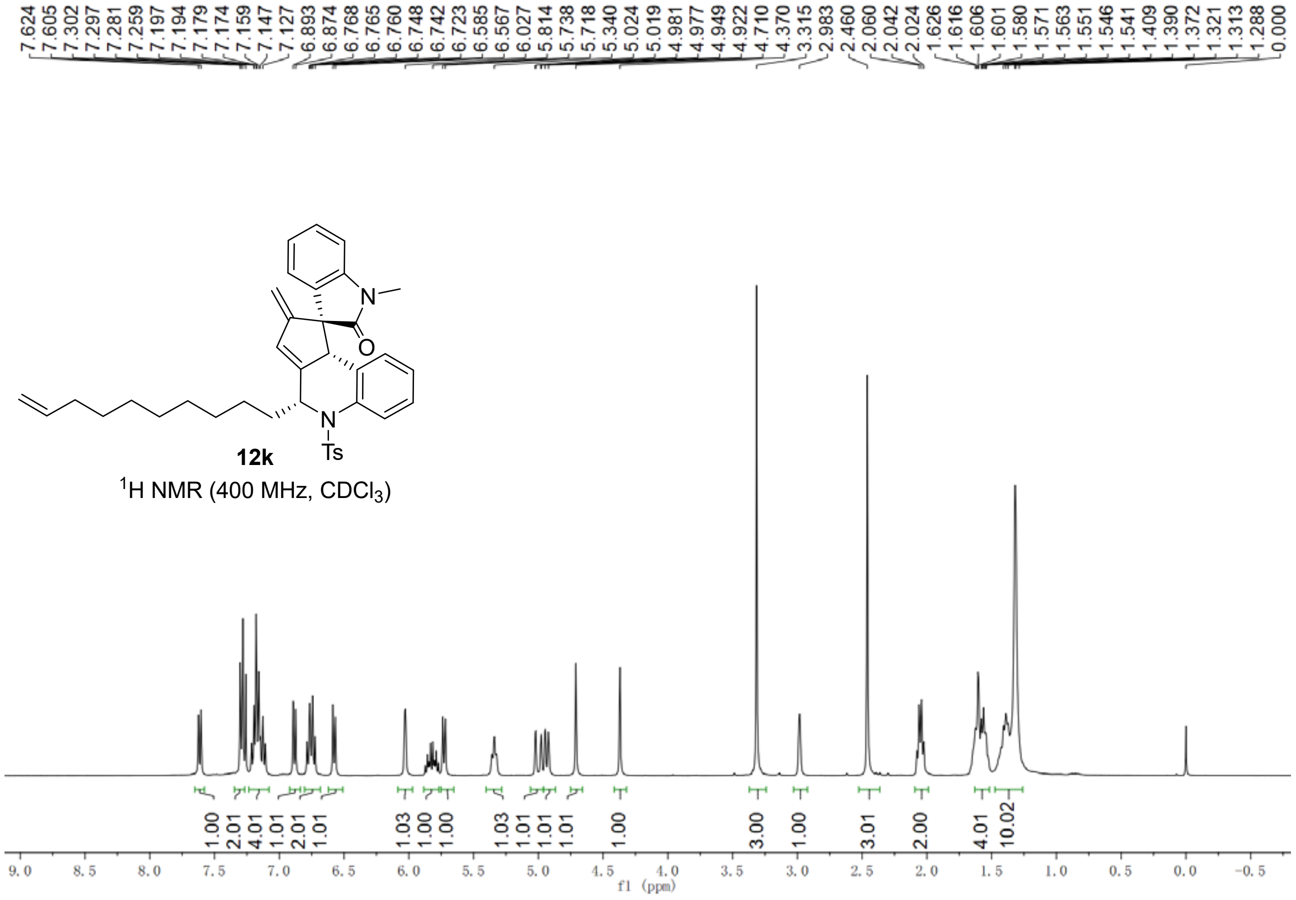

12k

^{1}H NMR (400 MHz, $CDCl_3$)

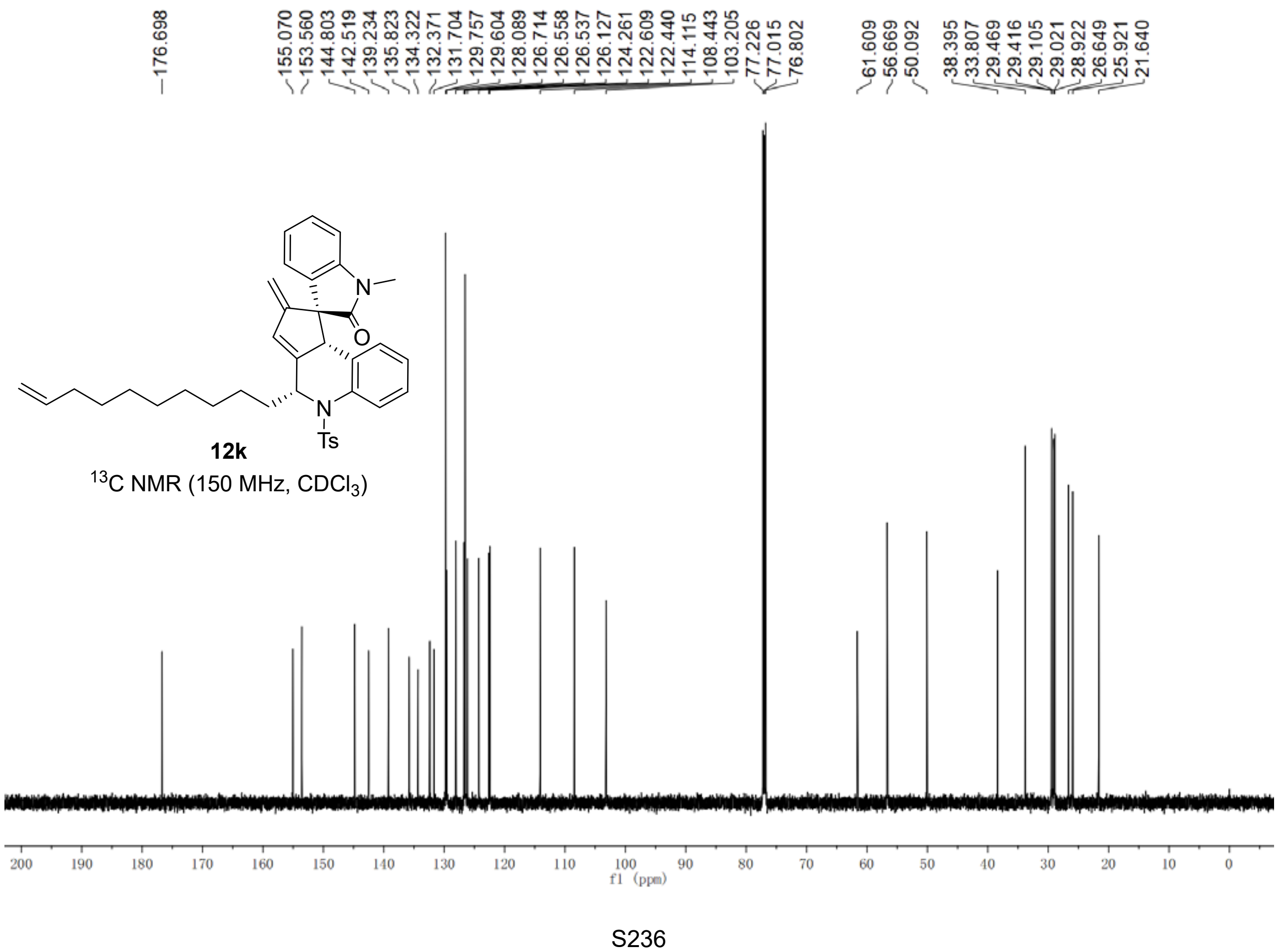

12k

^{13}C NMR (150 MHz, $CDCl_3$)

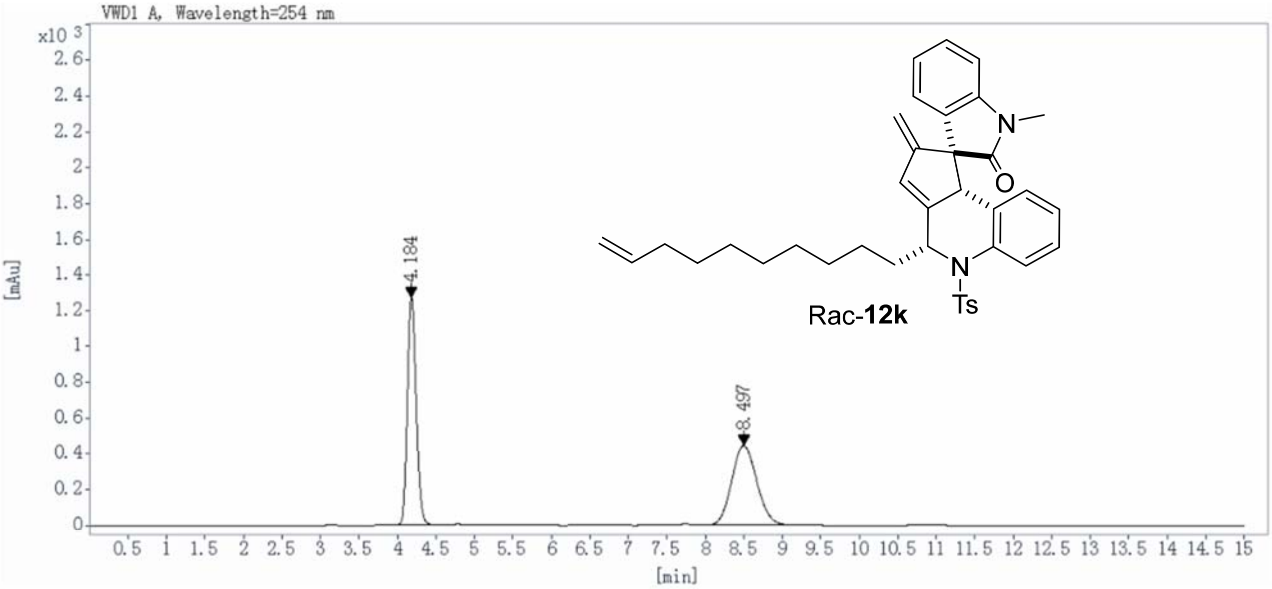

Ret Time [min]	Peak Type	Width [min]	Height [mAU]	Area [mAU*s]	Area [%]
4.184	BBA	0.12	1273.7031	10044.6152	49.9861
8.497	BBA	0.35	443.9998	10050.2139	50.0139
			Totals:	20094.8291	100.0000

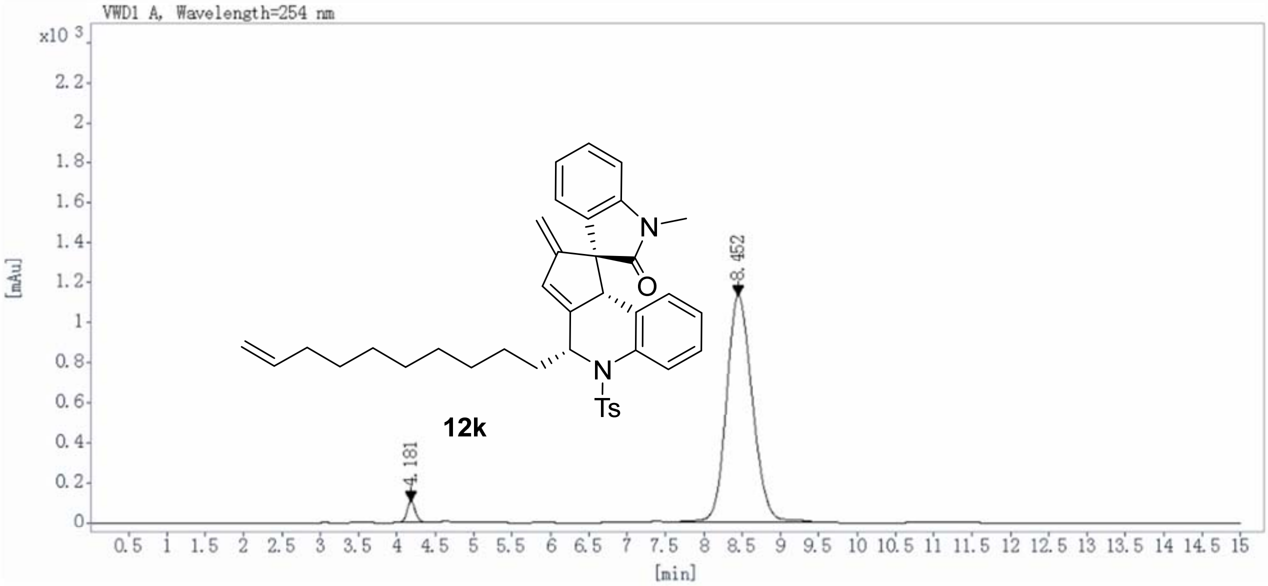

Ret Time [min]	Peak Type	Width [min]	Height [mAU]	Area [mAU*s]	Area [%]
4.181	BB	0.12	104.9507	822.0416	2.9815
8.452	BB	0.37	1132.7063	26749.0391	97.0185
			Totals:	27571.0807	100.0000

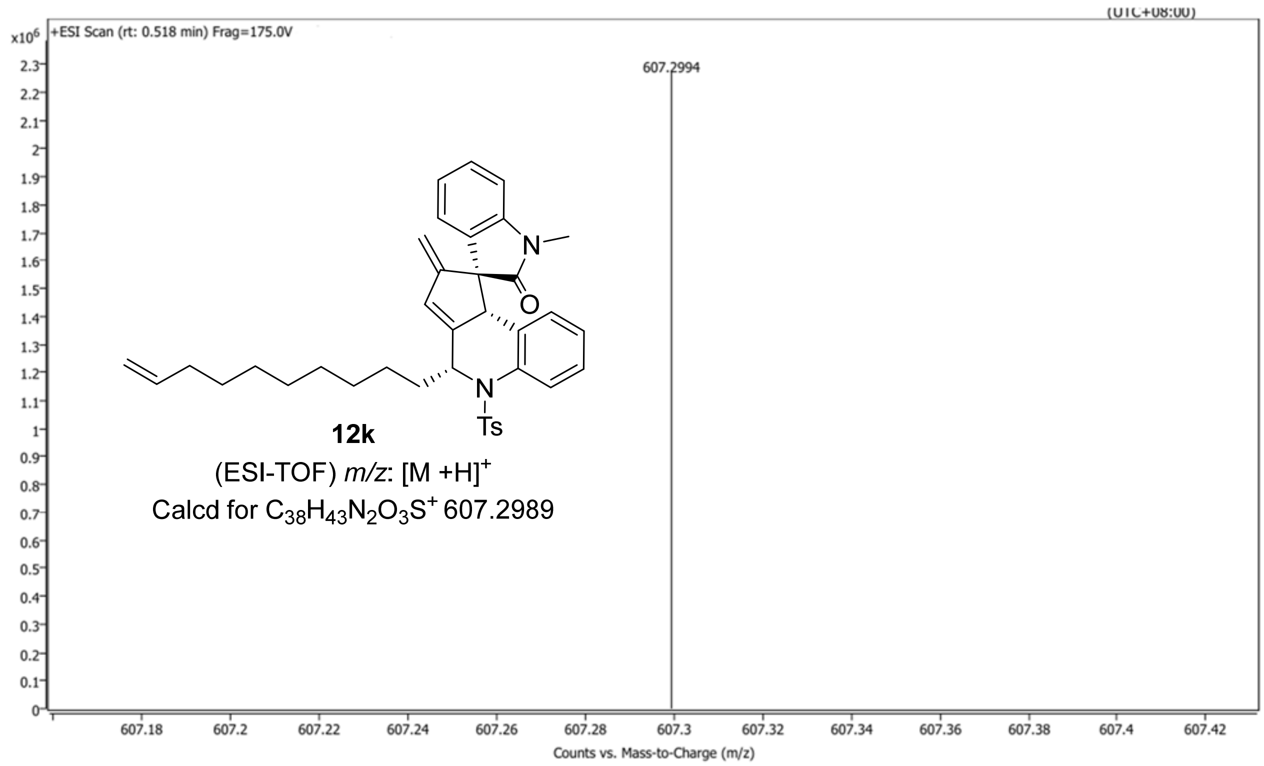
(UTC+08:00)
x10^6
+ESI Scan (rt: 0.518 min) Frag=175.0V
607.2994
12k
(ESI-TOF) m/z: [M +H]+
Calcd for C38H43N2O3S+ 607.2989
Counts vs. Mass-to-Charge (m/z)

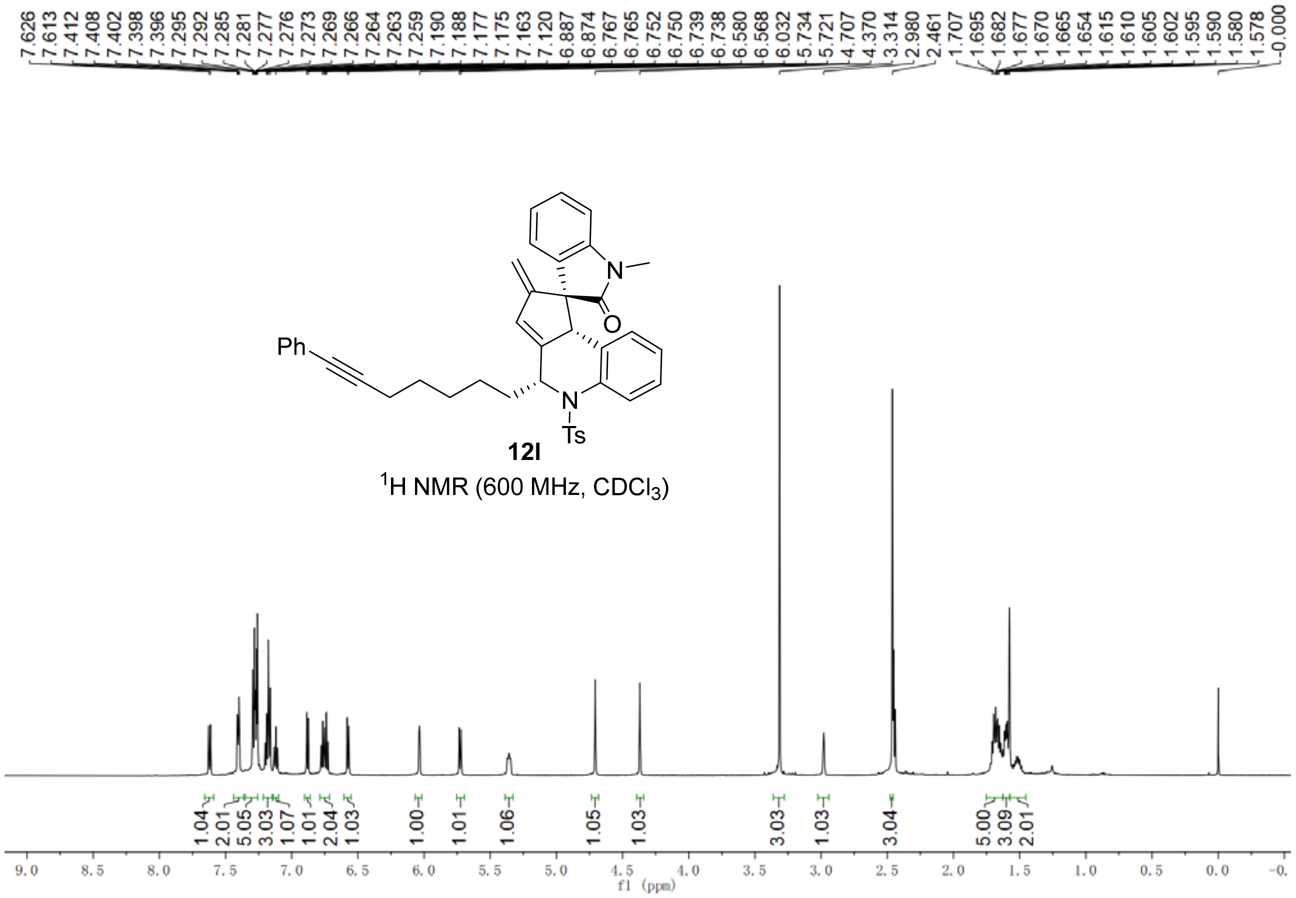
7.626
7.613
7.412
7.408
7.402
7.398
7.396
7.295
7.292
7.285
7.281
7.277
7.276
7.273
7.269
7.266
7.264
7.263
7.259
7.190
7.188
7.177
7.175
7.163
7.120
6.887
6.874
6.767
6.765
6.752
6.750
6.739
6.738
6.580
6.568
6.032
5.734
5.721
4.707
4.370
3.314
2.980
2.461
1.707
1.695
1.682
1.677
1.670
1.665
1.654
1.615
1.610
1.605
1.602
1.595
1.590
1.580
1.578
-0.000
Ph
N
O
N
Ts
12l
1H NMR (600 MHz, CDCl3)
1.04
2.01
5.05
3.03
1.07
1.01
2.04
1.03
1.00
1.01
1.06
1.05
1.03
3.03
1.03
3.04
5.00
3.09
2.01
f1 (ppm)

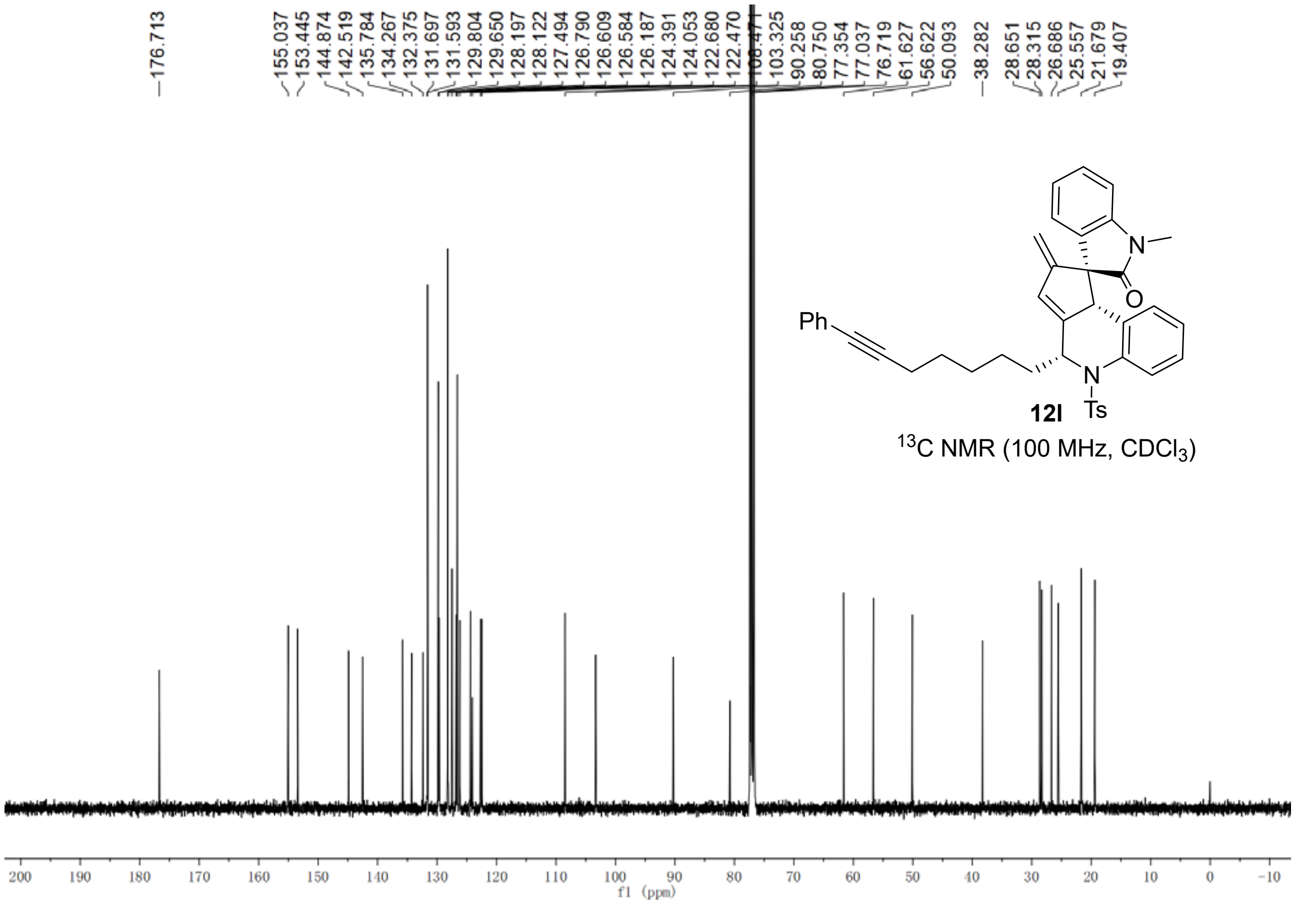
176.713
155.037
153.445
144.874
142.519
135.784
134.267
132.375
131.697
131.593
129.804
129.650
128.197
128.122
127.494
126.790
126.609
126.584
126.187
124.391
124.053
122.680
122.470
103.325
90.258
80.750
77.354
77.037
76.719
61.627
56.622
50.093
38.282
28.651
28.315
26.686
25.557
21.679
19.407
Ph
N
O
N
Ts
12l
13C NMR (100 MHz, CDCl3)
f1 (ppm)

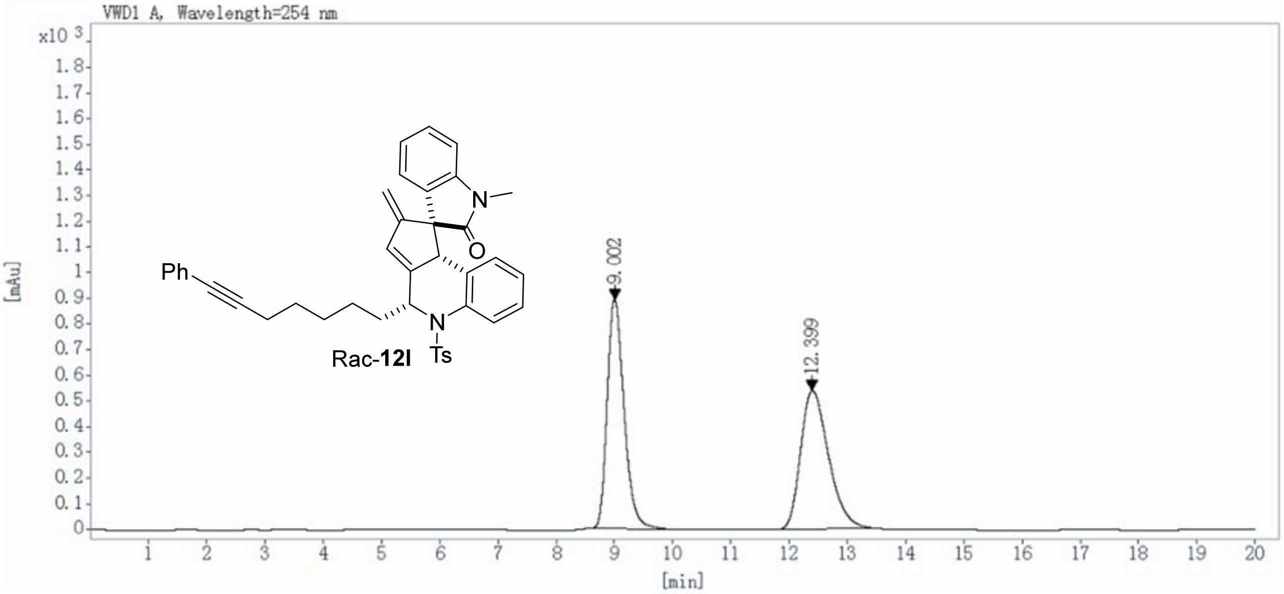

Ret Time [min]	Peak Type	Width [min]	Height [mAU]	Area [mAU*s]	Area [%]
9.002	BBA	0.31	891.9315	17841.7578	49.6578
12.399	BBA	0.52	540.7164	18087.6445	50.3422
			Totals:	35929.4023	100.0000

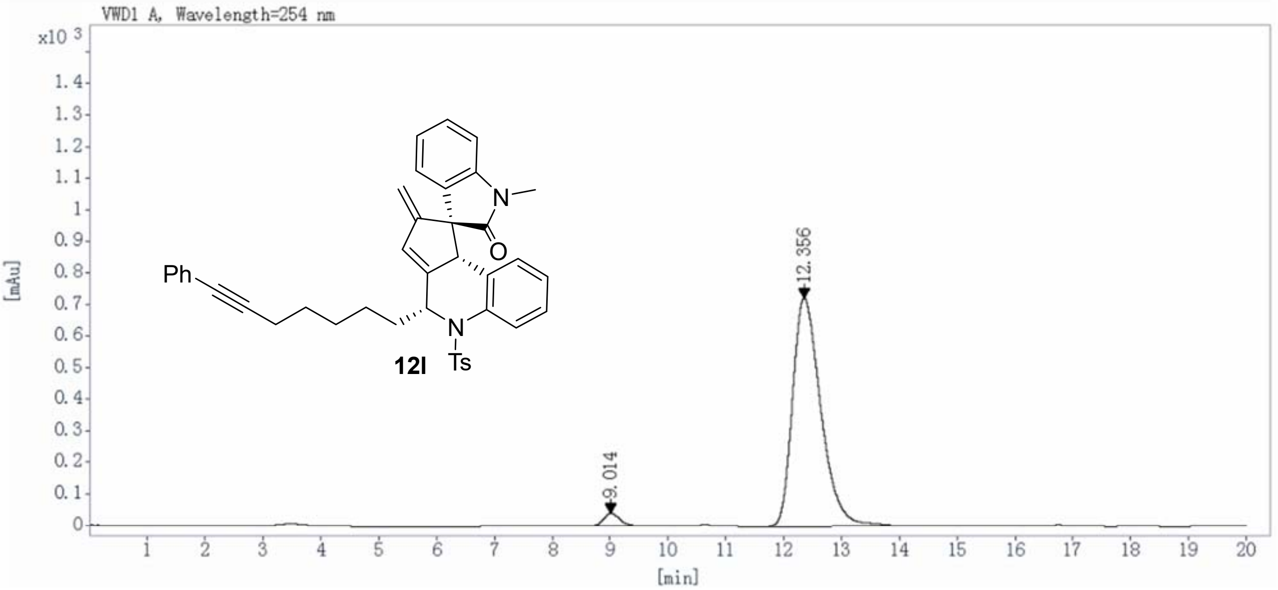

Ret Time [min]	Peak Type	Width [min]	Height [mAU]	Area [mAU*s]	Area [%]
9.014	BBA	0.29	40.3394	763.8903	3.0236
12.356	BBA	0.52	721.9957	24500.3711	96.9764
			Totals:	25264.2614	100.0000

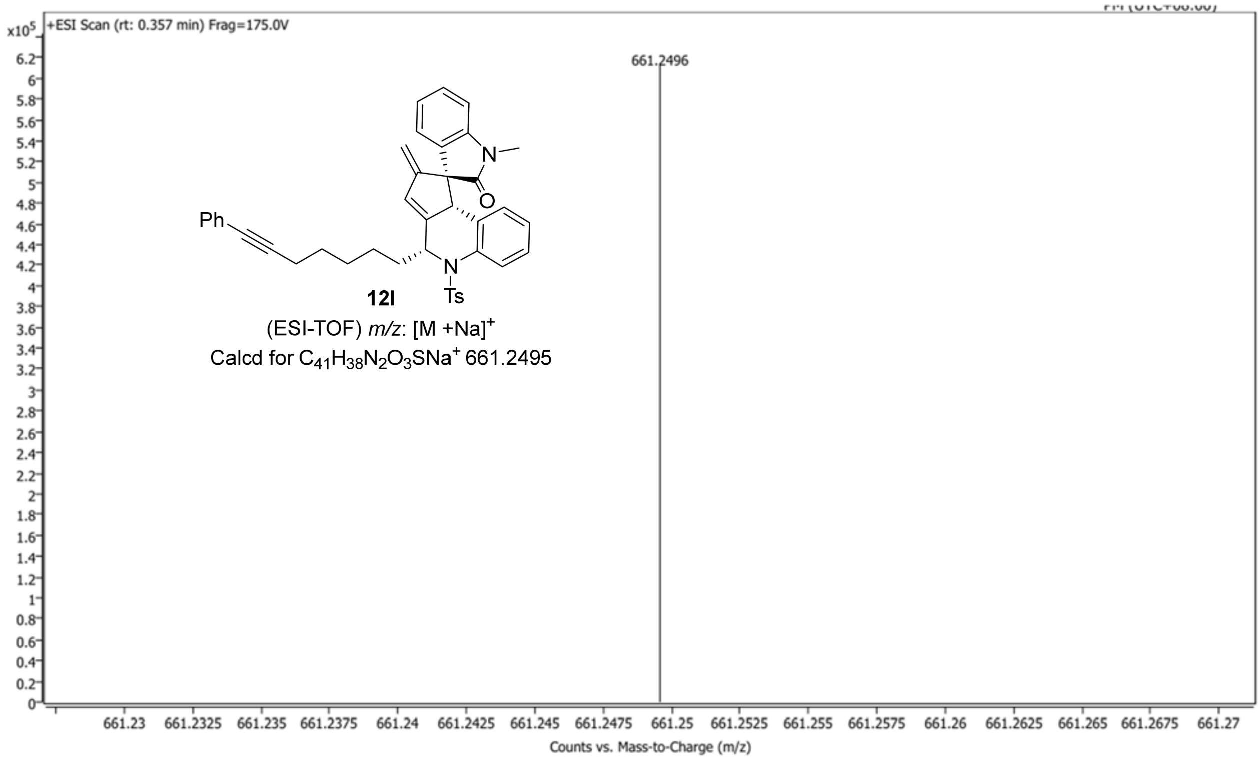

+ESI Scan (rt: 0.357 min) Frag=175.0V
x10^5
661.2496
Ph
N
O
N
Ts
12l
(ESI-TOF) m/z: [M +Na]+
Calcd for C41H38N2O3SNa+ 661.2495
Counts vs. Mass-to-Charge (m/z)

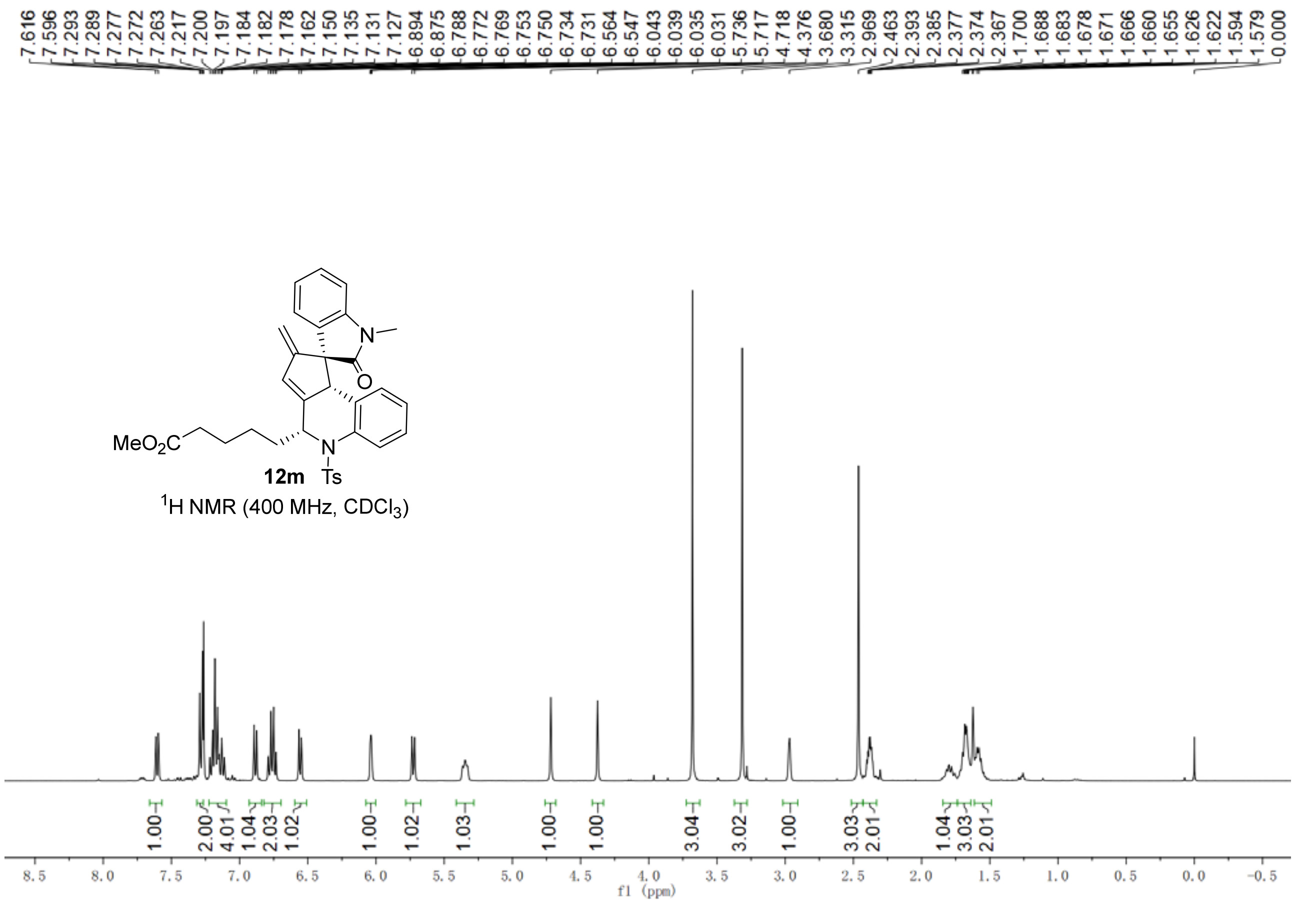

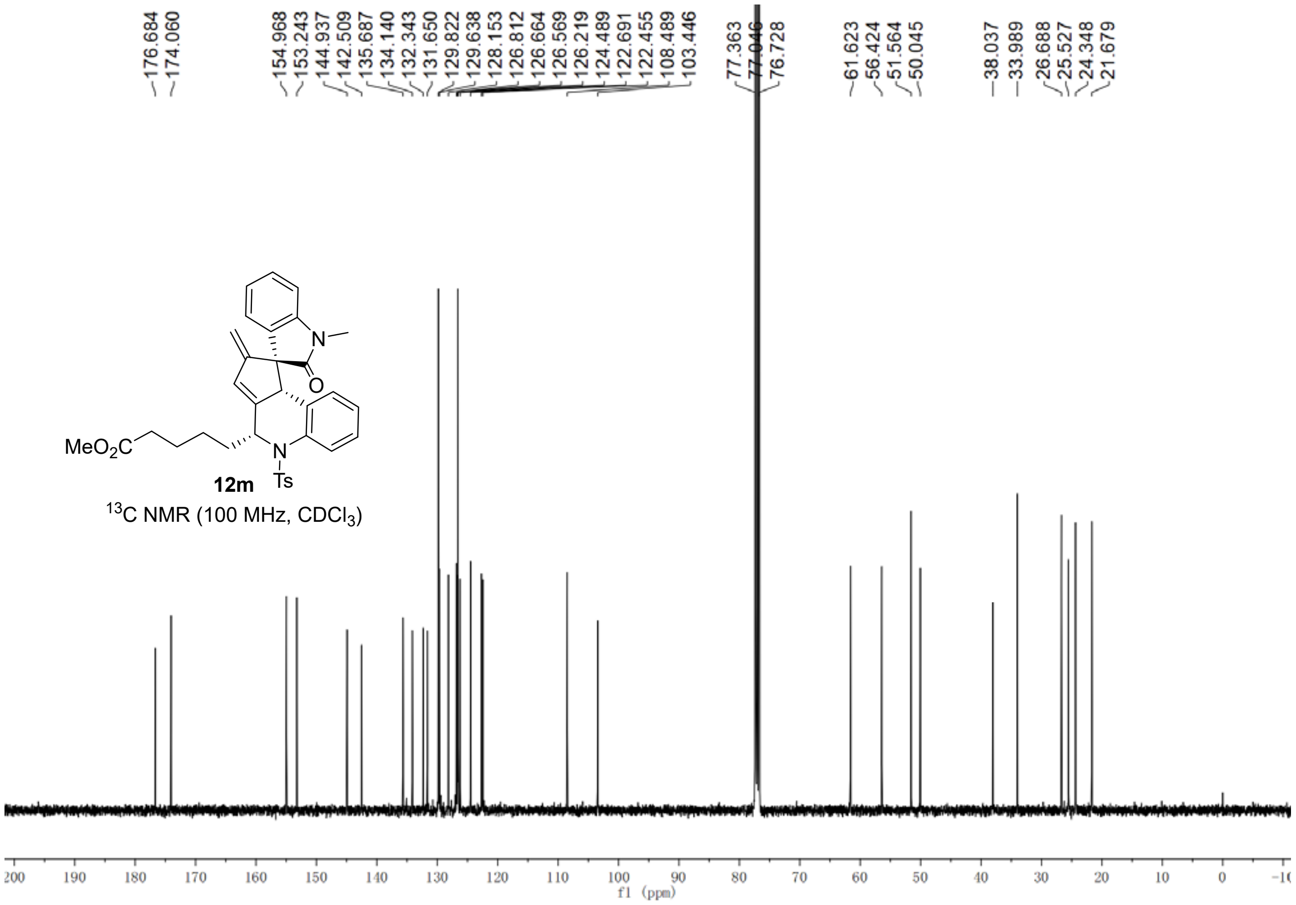

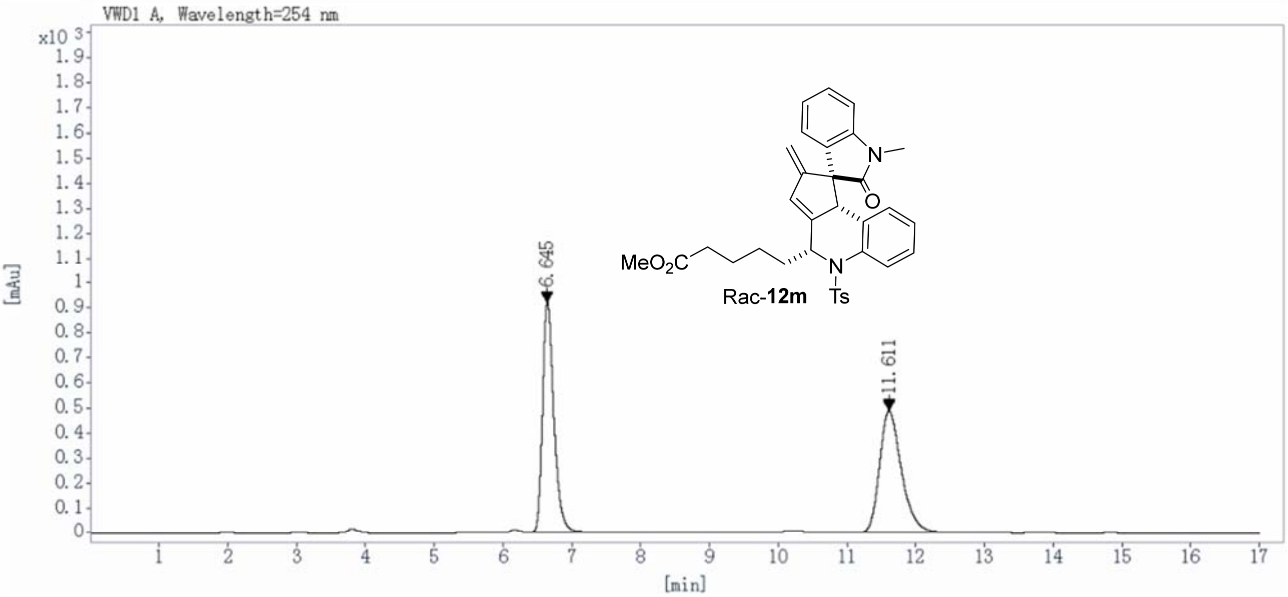

Ret Time [min]	Peak Type	Width [min]	Height [mAU]	Area [mAU*s]	Area [%]
6.645	BV R	0.17	922.2198	10522.9707	49.3114
11.611	BBA	0.34	489.4550	10816.8643	50.6886
			Totals:	21339.8350	100.0000

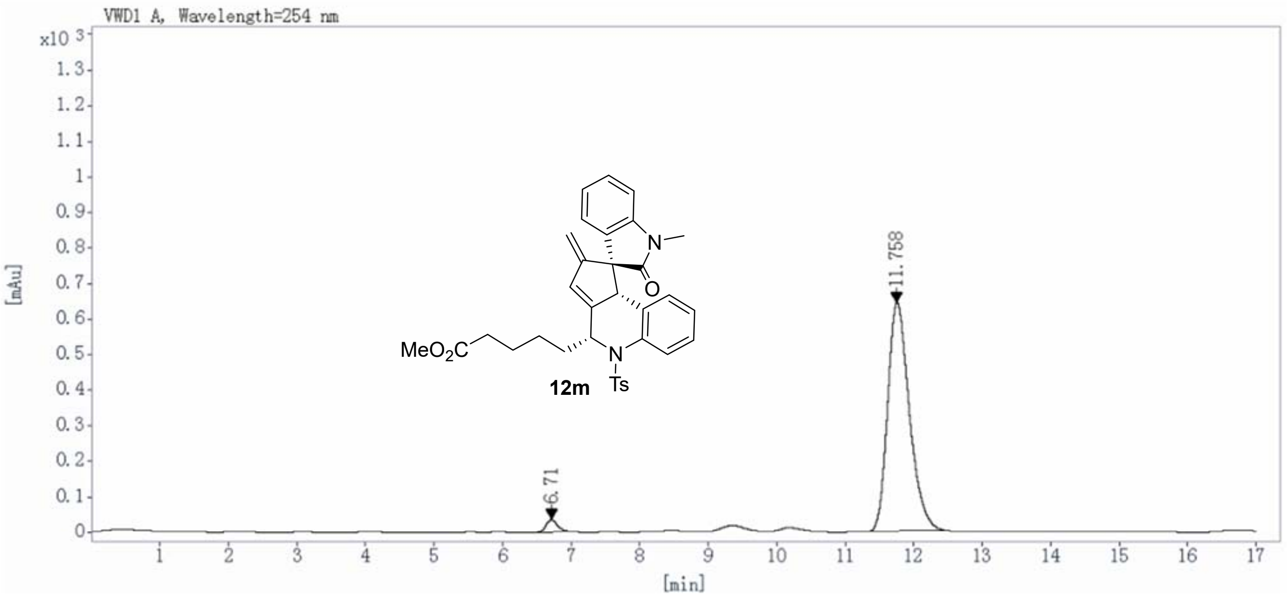

Ret Time [min]	Peak Type	Width [min]	Height [mAU]	Area [mAU*s]	Area [%]
6.710	BBA	0.17	34.9625	388.7296	2.6562
11.758	BBA	0.34	644.5510	14246.2002	97.3438
			Totals:	14634.9298	100.0000

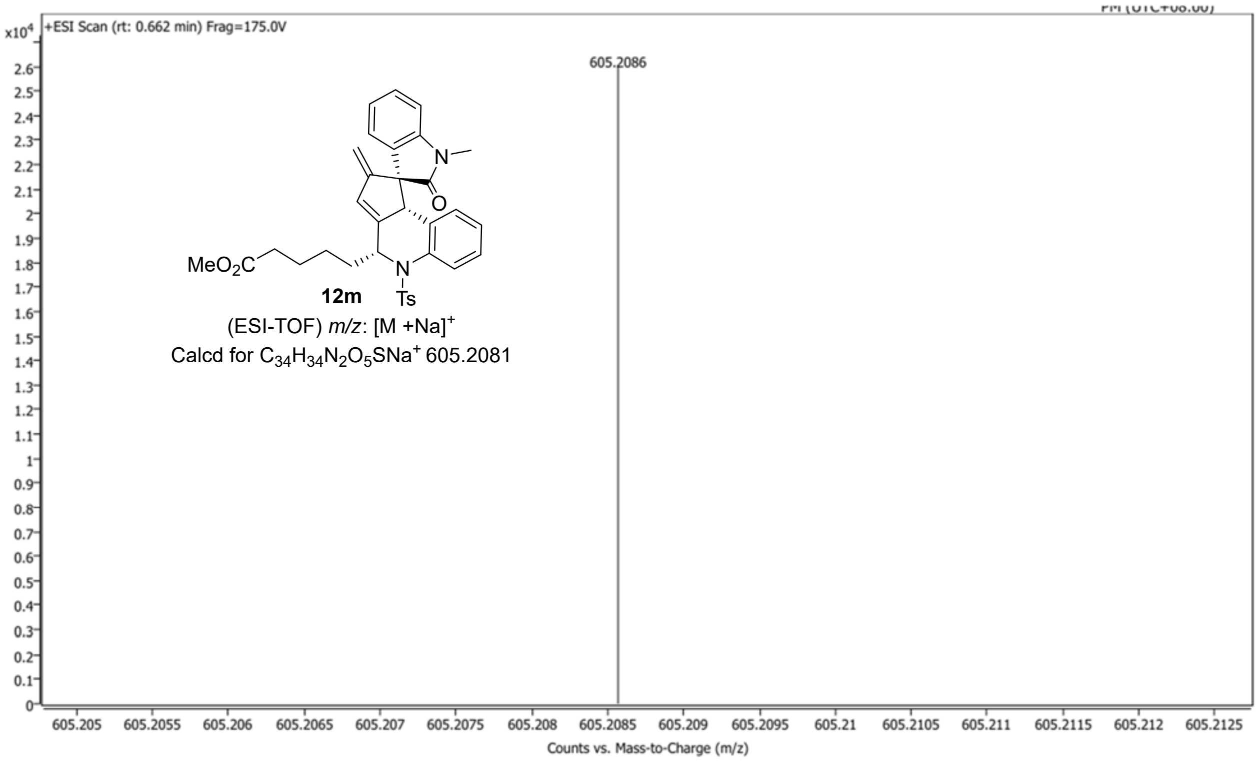

PM (UTC+08:00)
x10^4
+ESI Scan (rt: 0.662 min) Frag=175.0V
605.2086
MeO2C
N
O
N
Ts
12m
(ESI-TOF) m/z: [M +Na]+
Calcd for C34H34N2O5SNa+ 605.2081
Counts vs. Mass-to-Charge (m/z)

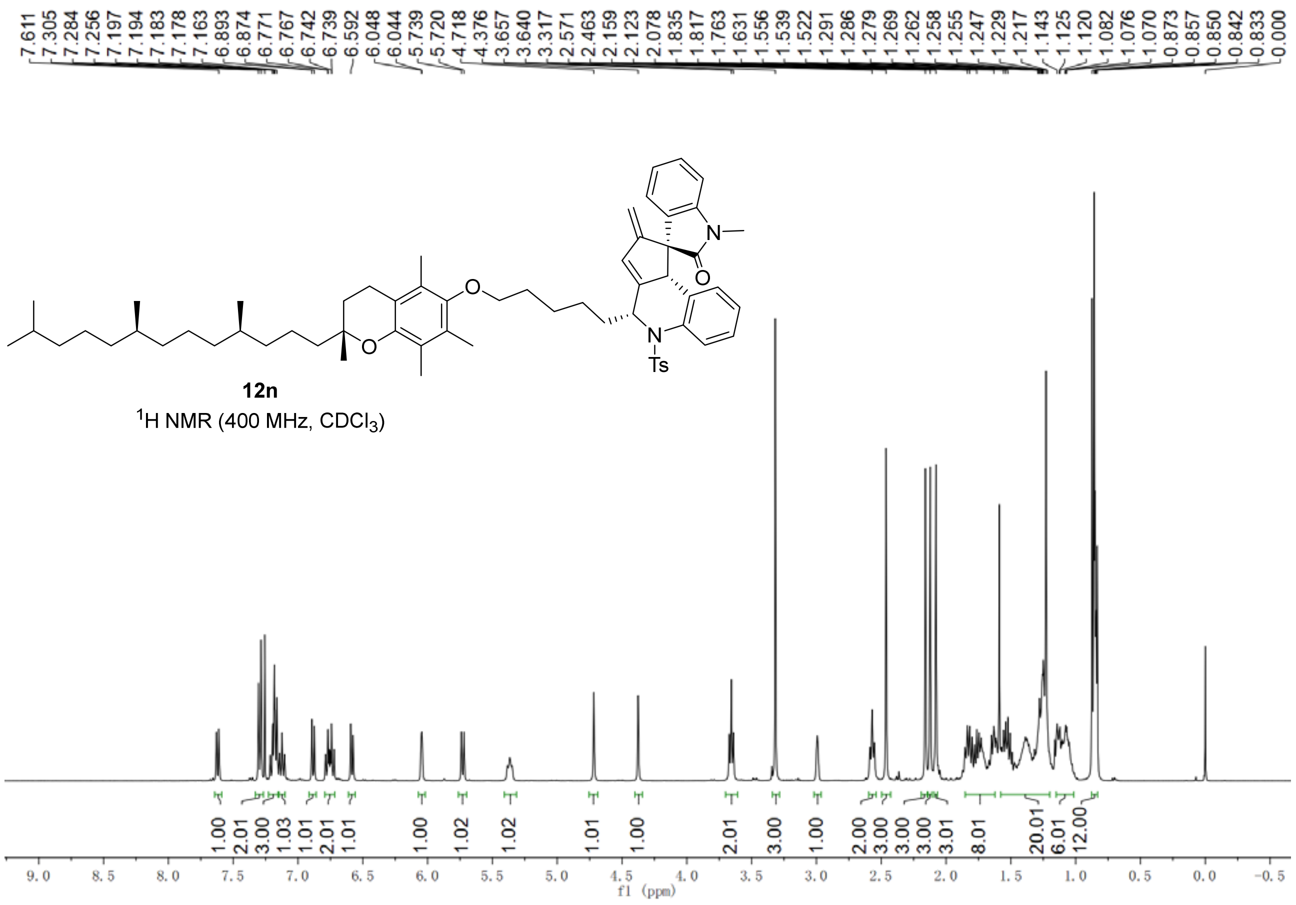

12n

^{1}H NMR (400 MHz, $CDCl_3$)

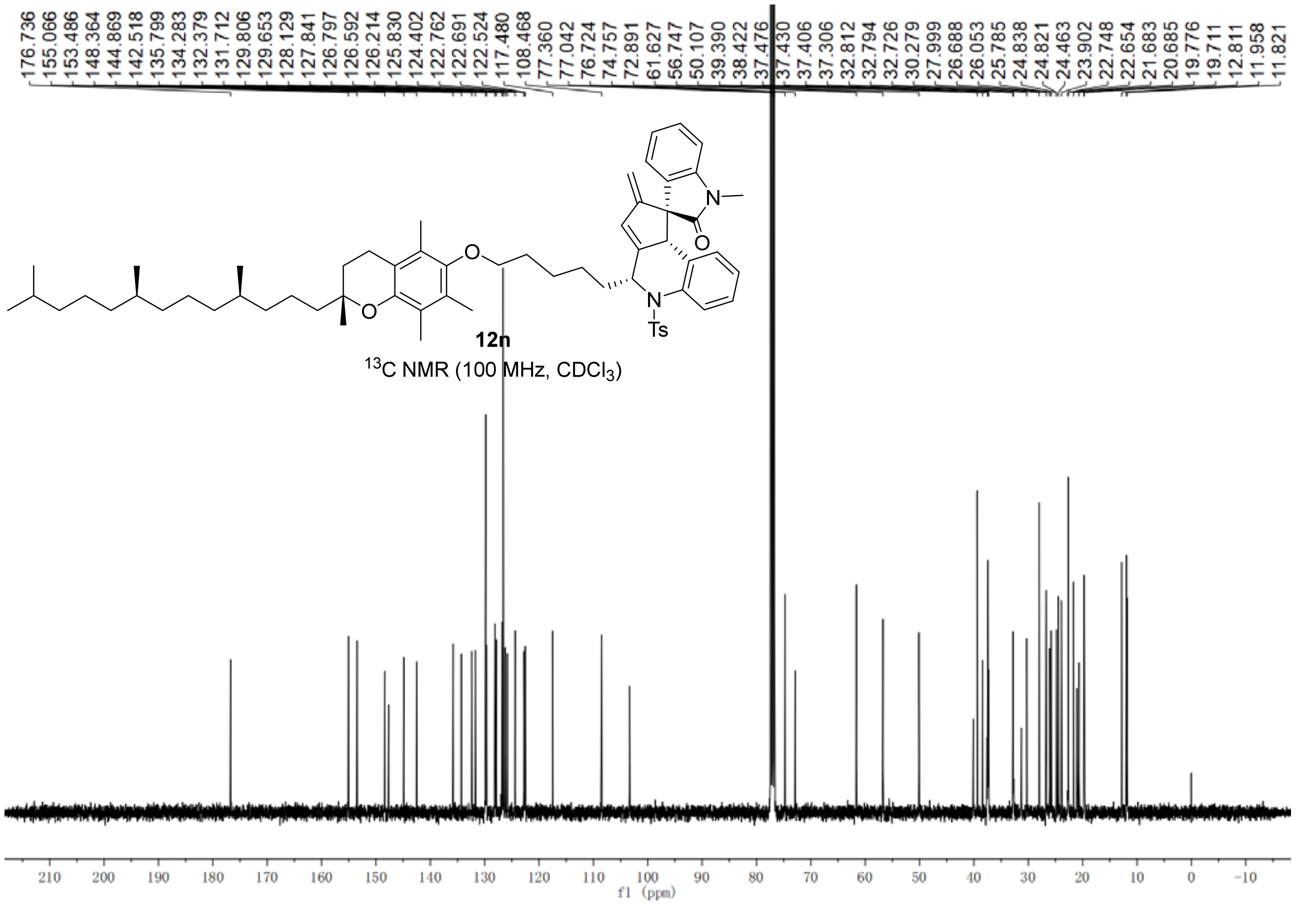

12n

^{13}C NMR (100 MHz, $CDCl_3$)

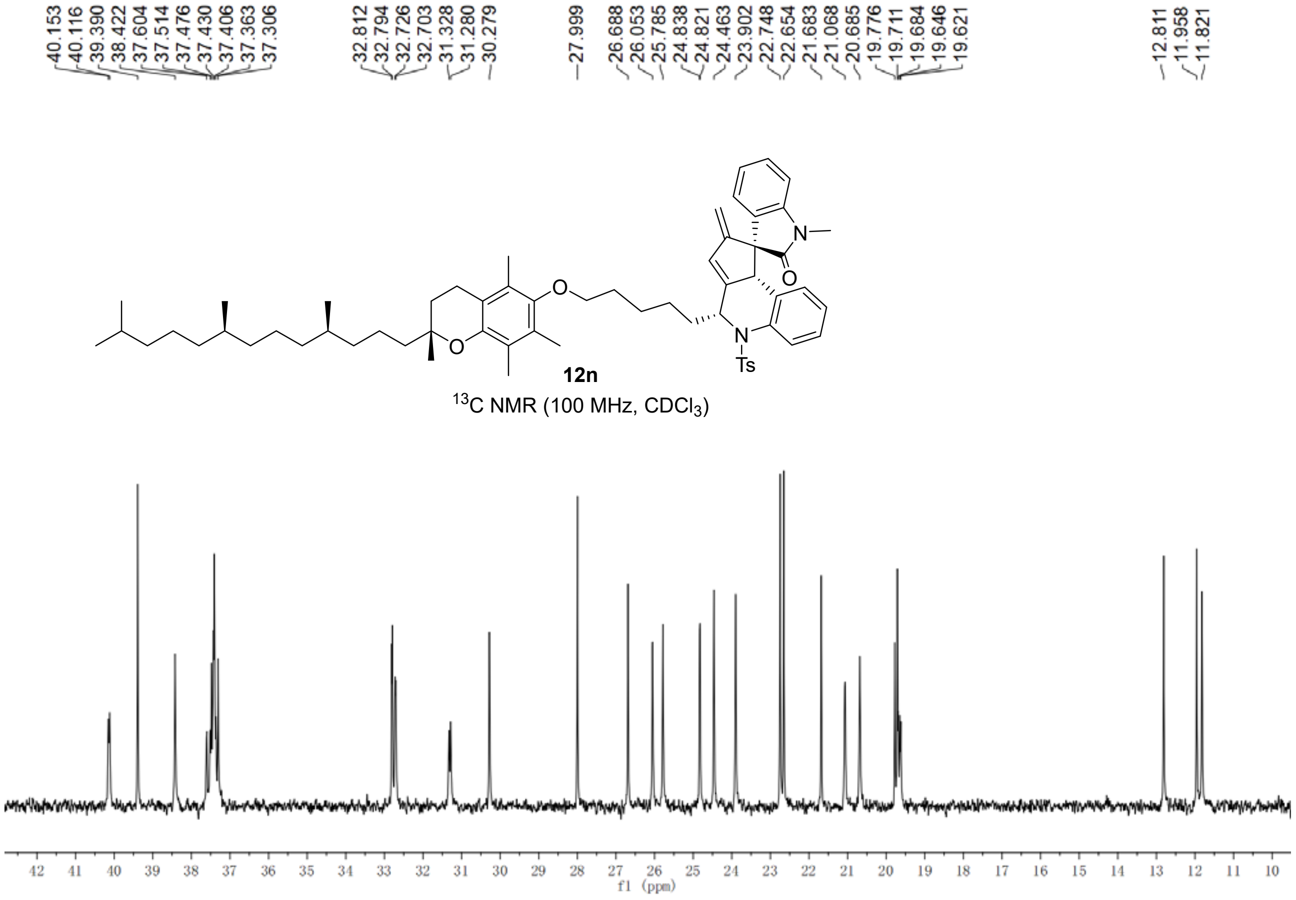
40.153
40.116
39.390
38.422
37.604
37.514
37.476
37.430
37.406
37.363
37.306
32.812
32.794
32.726
32.703
31.328
31.280
30.279
27.999
26.688
26.053
25.785
24.838
24.821
24.463
23.902
22.748
22.654
21.683
21.068
20.685
19.776
19.711
19.684
19.646
19.621
12.811
11.958
11.821
O
N
O
N
Ts
12n
^{13}C NMR (100 MHz, $CDCl_3$)
f1 (ppm)

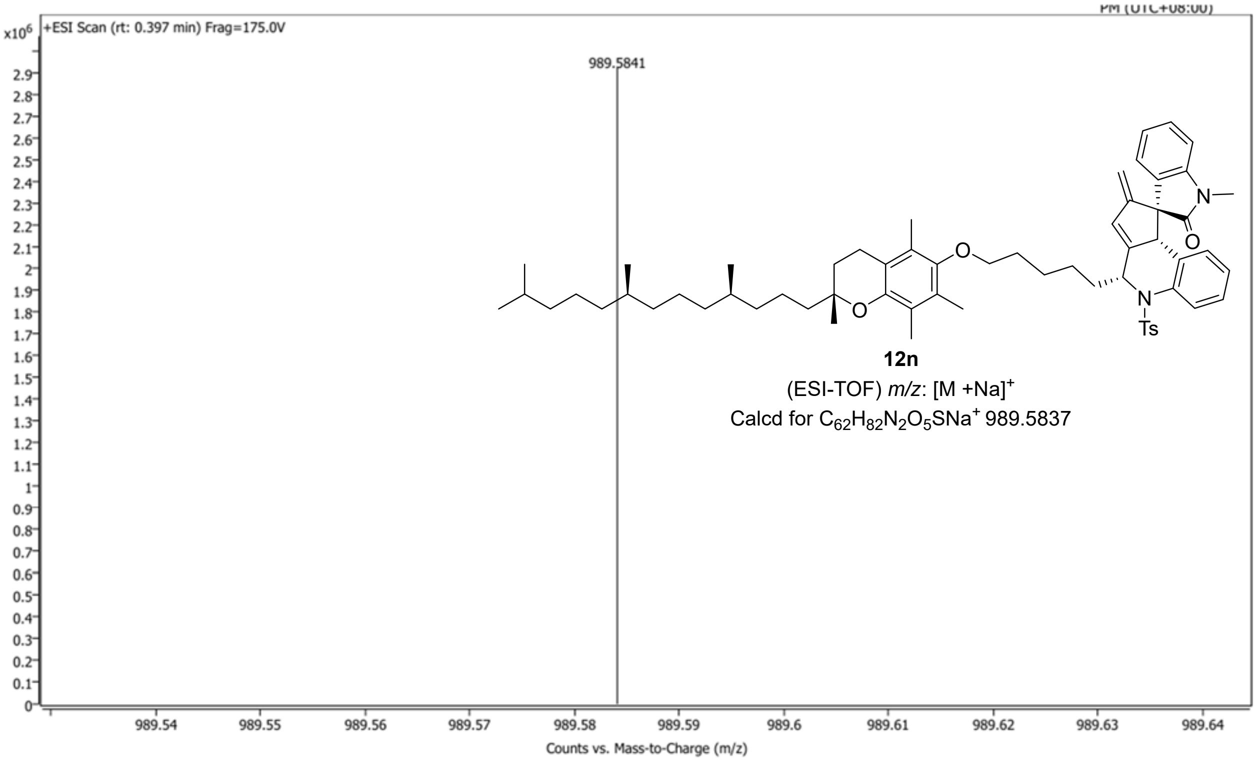
PM (UTC+08:00)
x10^6
+ESI Scan (rt: 0.397 min) Frag=175.0V
989.5841
12n
(ESI-TOF) *m/z*: [M +Na]$^+$
Calcd for $C_{62}H_{82}N_2O_5SNa^+$ 989.5837
Counts vs. Mass-to-Charge (m/z)

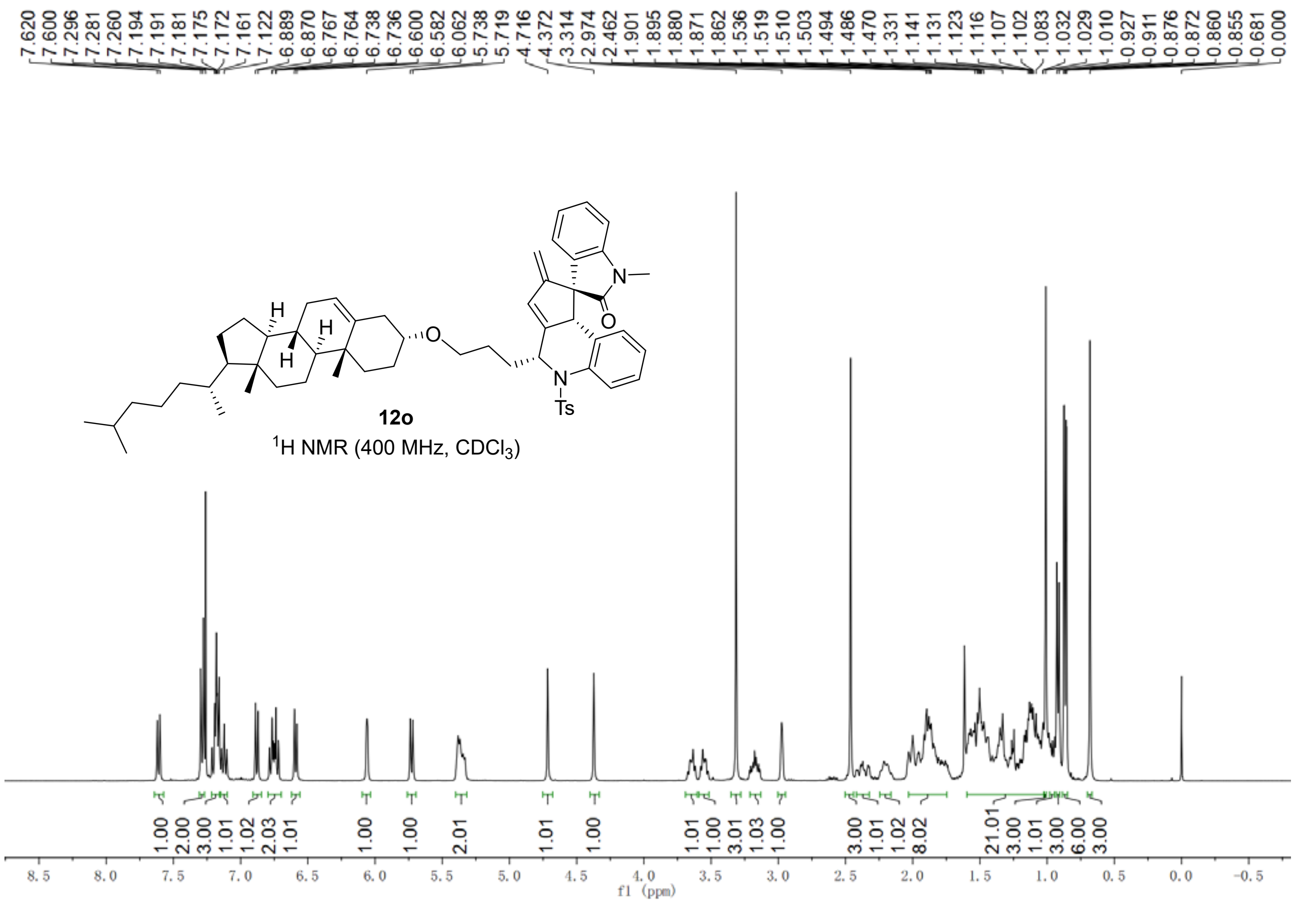
7.620 7.600 7.296 7.281 7.260 7.194 7.191 7.181 7.175 7.172 7.161 7.122 6.889 6.870 6.767 6.764 6.738 6.736 6.600 6.582 6.062 5.738 5.719 4.716 4.372 3.314 2.974 2.462 1.901 1.895 1.880 1.871 1.862 1.536 1.519 1.510 1.503 1.494 1.486 1.470 1.331 1.141 1.131 1.123 1.116 1.107 1.102 1.083 1.032 1.029 1.010 0.927 0.911 0.876 0.872 0.860 0.855 0.681 0.000
H
H
H
O
N
O
N
Ts
12o
^{1}H NMR (400 MHz, CDCl$_3$)
1.00 2.00 3.00 1.01 1.02 2.03 1.01 1.00 1.00 2.01 1.01 1.00 1.01 1.00 3.01 1.03 1.00 3.00 1.01 1.02 8.02 21.01 3.00 1.01 3.00 6.00 3.00
8.5 8.0 7.5 7.0 6.5 6.0 5.5 5.0 4.5 4.0 3.5 3.0 2.5 2.0 1.5 1.0 0.5 0.0 -0.5
f1 (ppm)

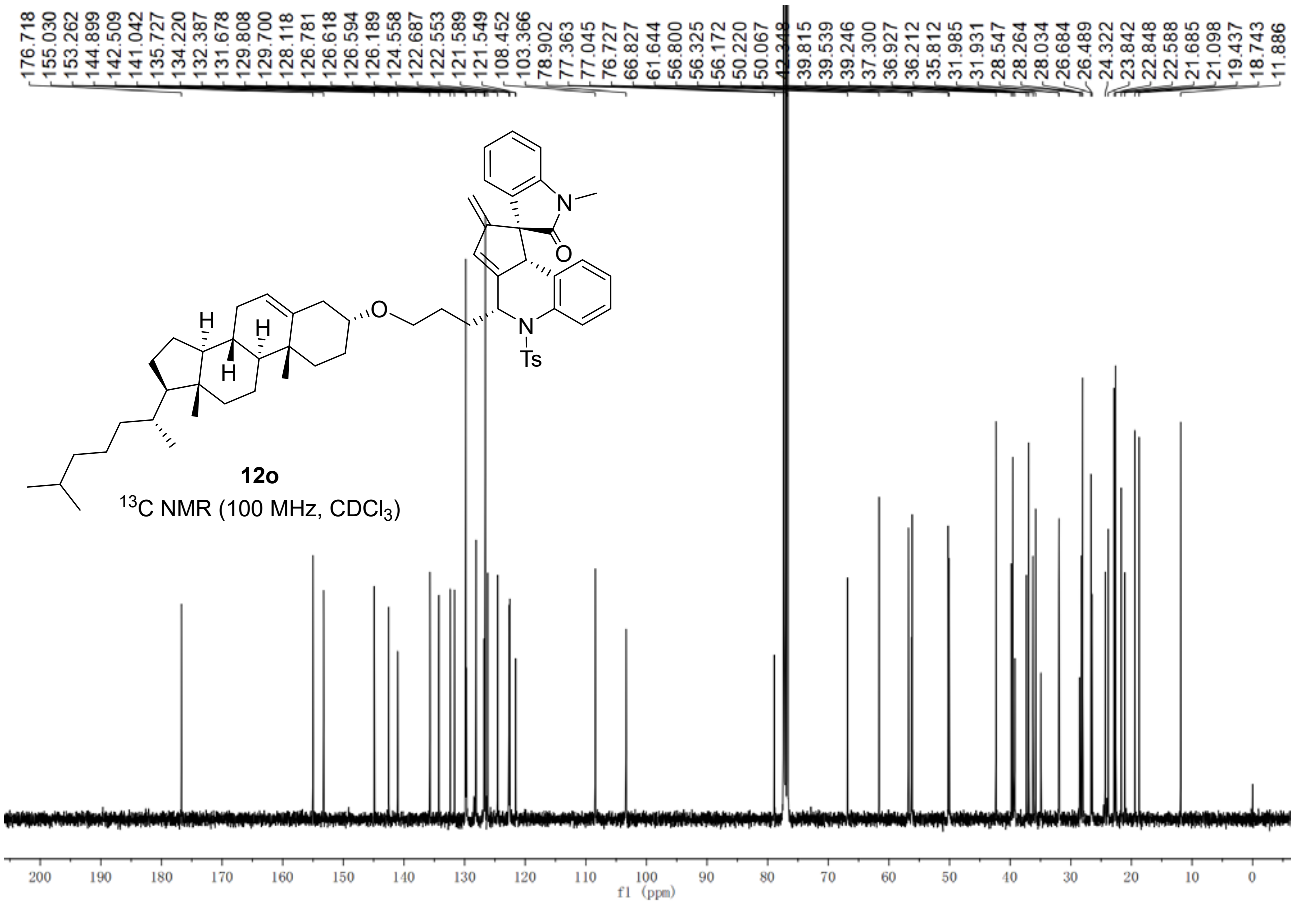
176.718 155.030 153.262 144.899 142.509 141.042 135.727 134.220 132.387 131.678 129.808 129.700 128.118 126.781 126.618 126.594 126.189 124.558 122.687 122.553 121.589 121.549 108.452 103.366 78.902 77.363 77.045 76.727 66.827 61.644 56.800 56.325 56.172 50.220 50.067 39.815 39.539 39.246 37.300 36.927 36.212 35.812 31.985 31.931 28.547 28.264 28.034 26.684 26.489 24.322 23.842 22.848 22.588 21.685 21.098 19.437 18.743 11.886
H
H
H
O
N
O
N
Ts
12o
^{13}C NMR (100 MHz, CDCl$_3$)
200 190 180 170 160 150 140 130 120 110 100 90 80 70 60 50 40 30 20 10 0
f1 (ppm)

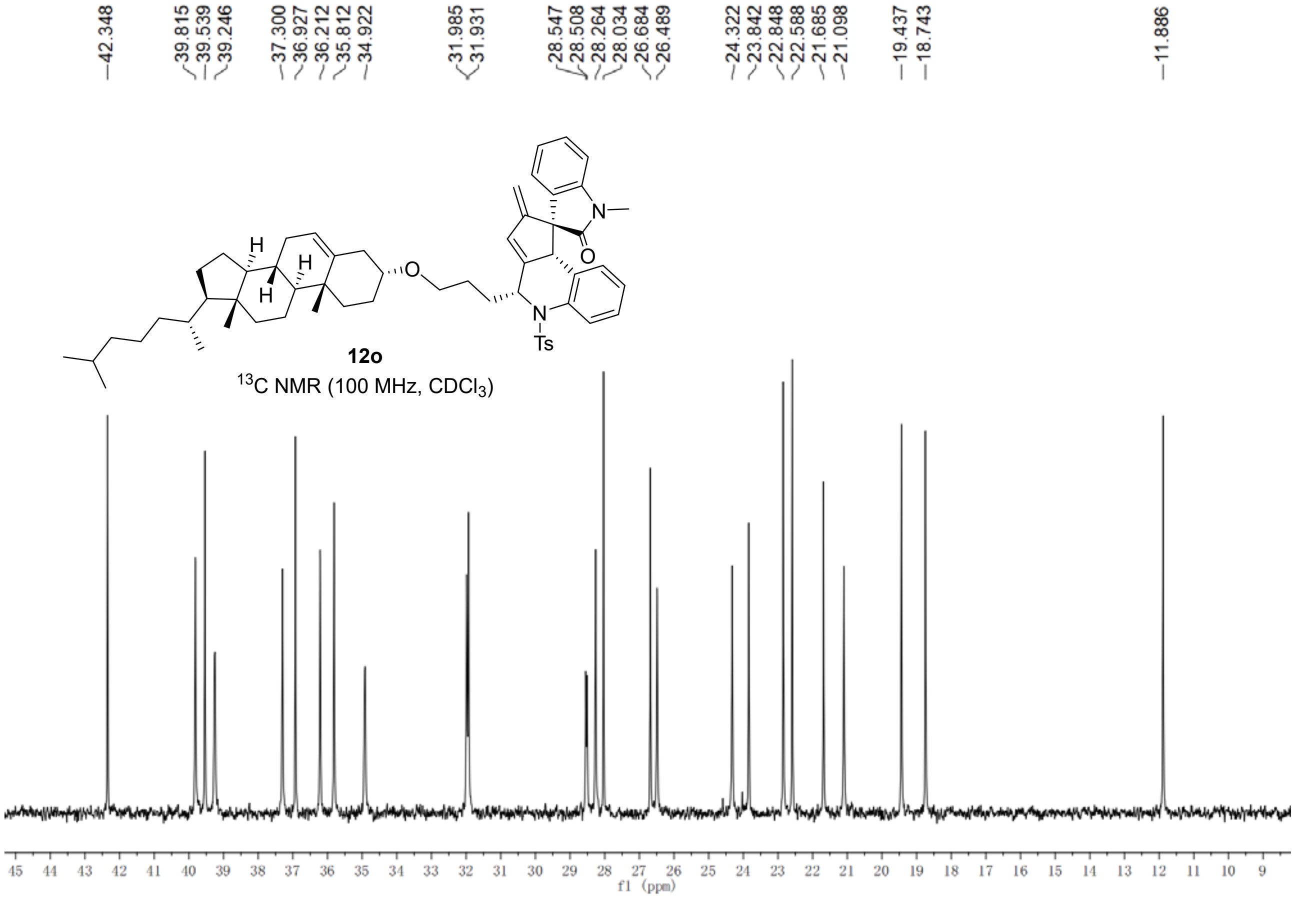

42.348
39.815
39.539
39.246
37.300
36.927
36.212
35.812
34.922
31.985
31.931
28.547
28.508
28.264
28.034
26.684
26.489
24.322
23.842
22.848
22.588
21.685
21.098
19.437
18.743
11.886
12o
^{13}C NMR (100 MHz, $CDCl_3$)
f1 (ppm)

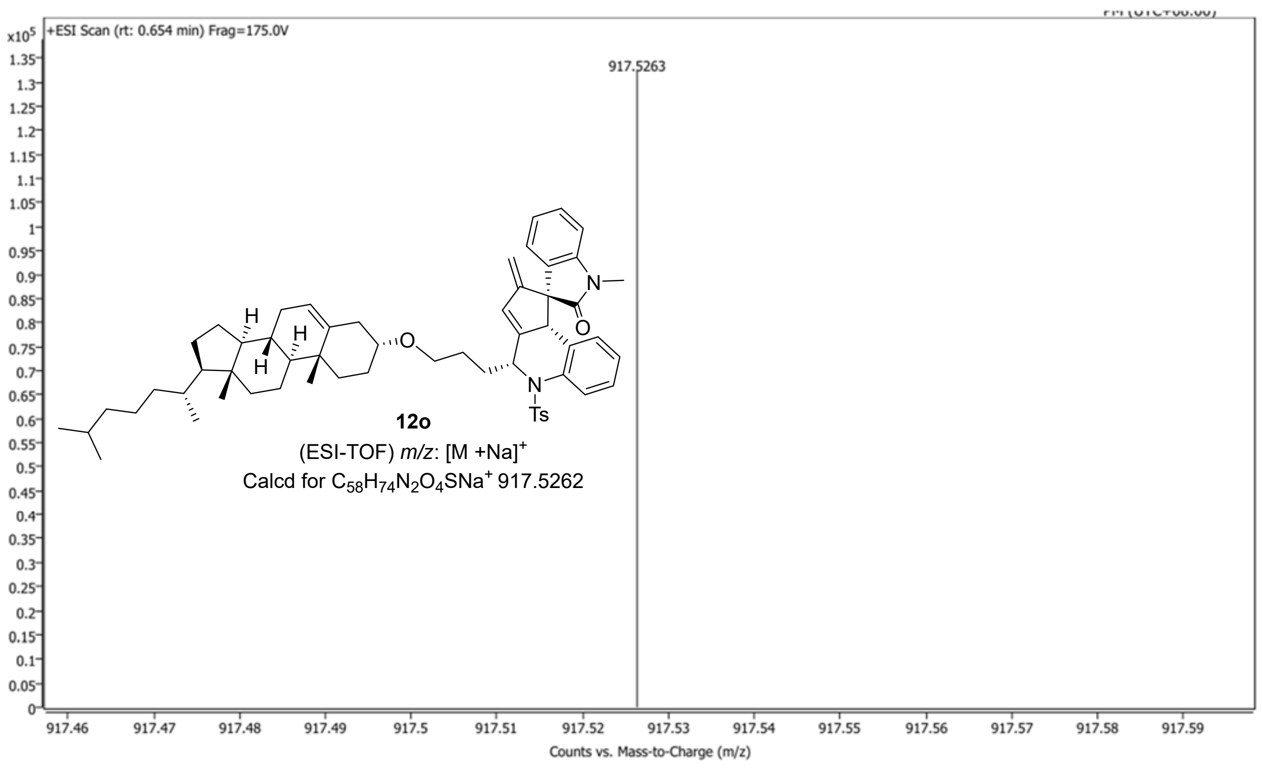

+ESI Scan (rt: 0.654 min) Frag=175.0V
x10^5
917.5263
12o
(ESI-TOF) *m/z*: [M +Na]$^+$
Calcd for $C_{58}H_{74}N_2O_4SNa^+$ 917.5262
Counts vs. Mass-to-Charge (m/z)

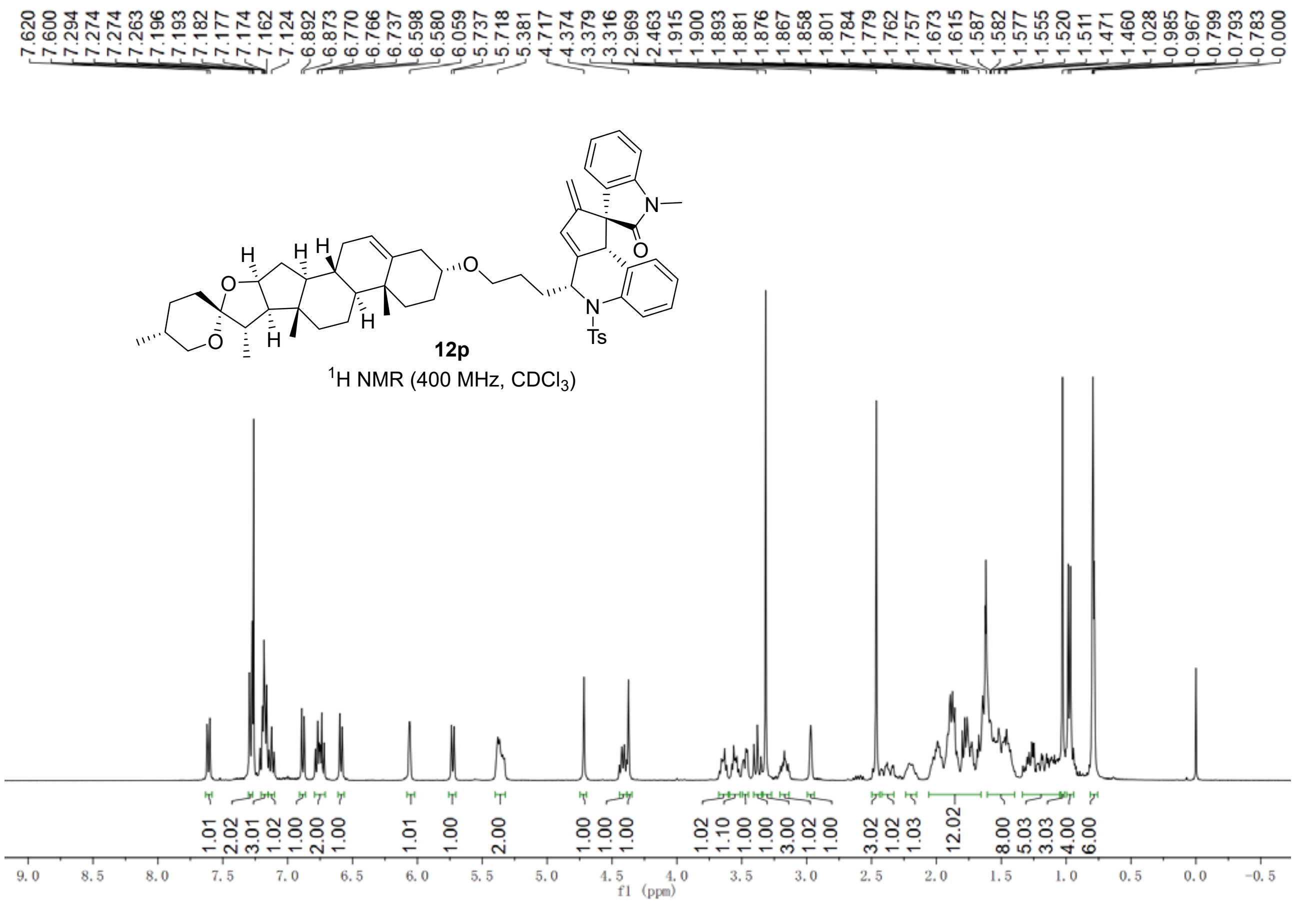

12p

^{1}H NMR (400 MHz, $CDCl_3$)

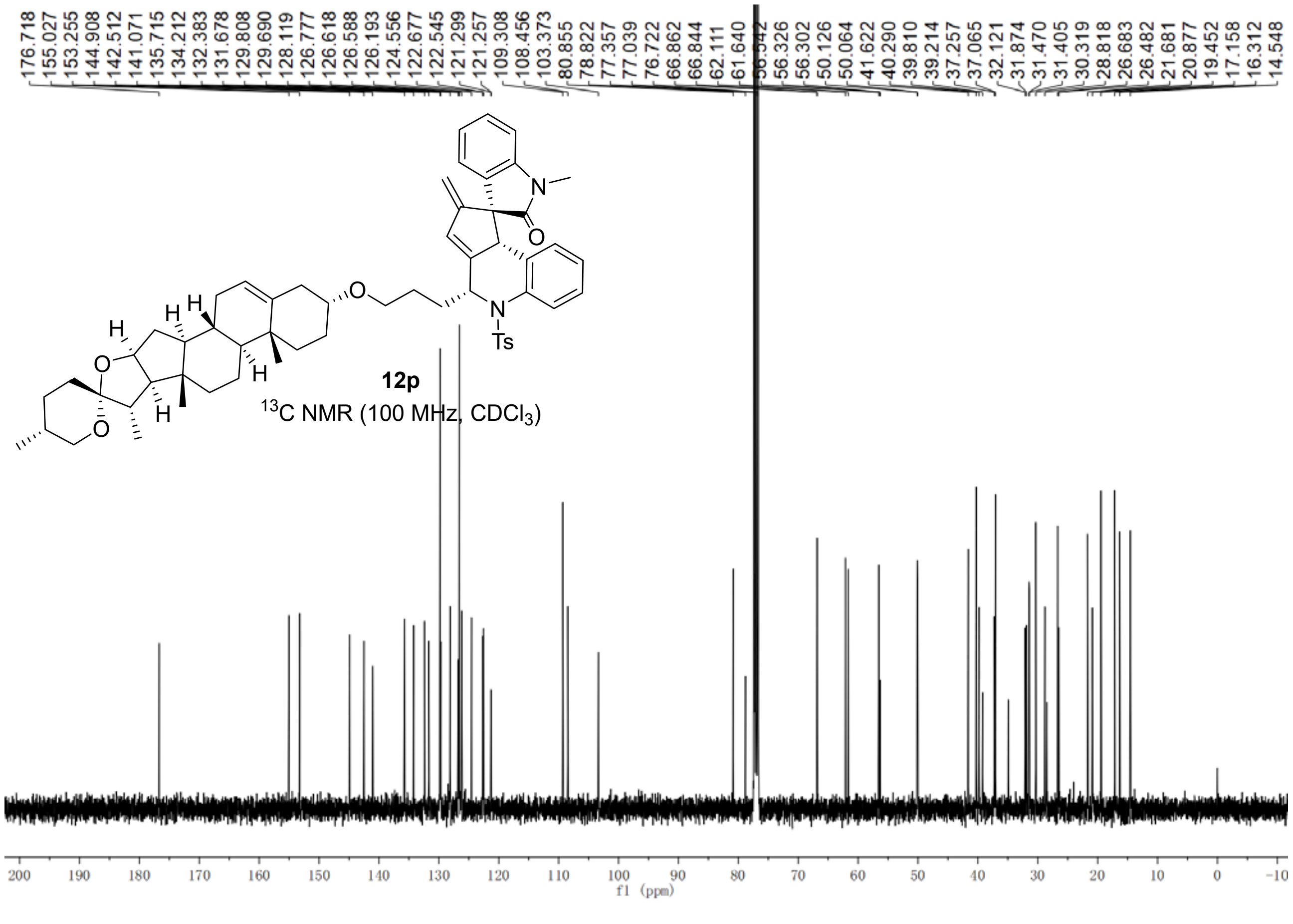

12p

^{13}C NMR (100 MHz, $CDCl_3$)

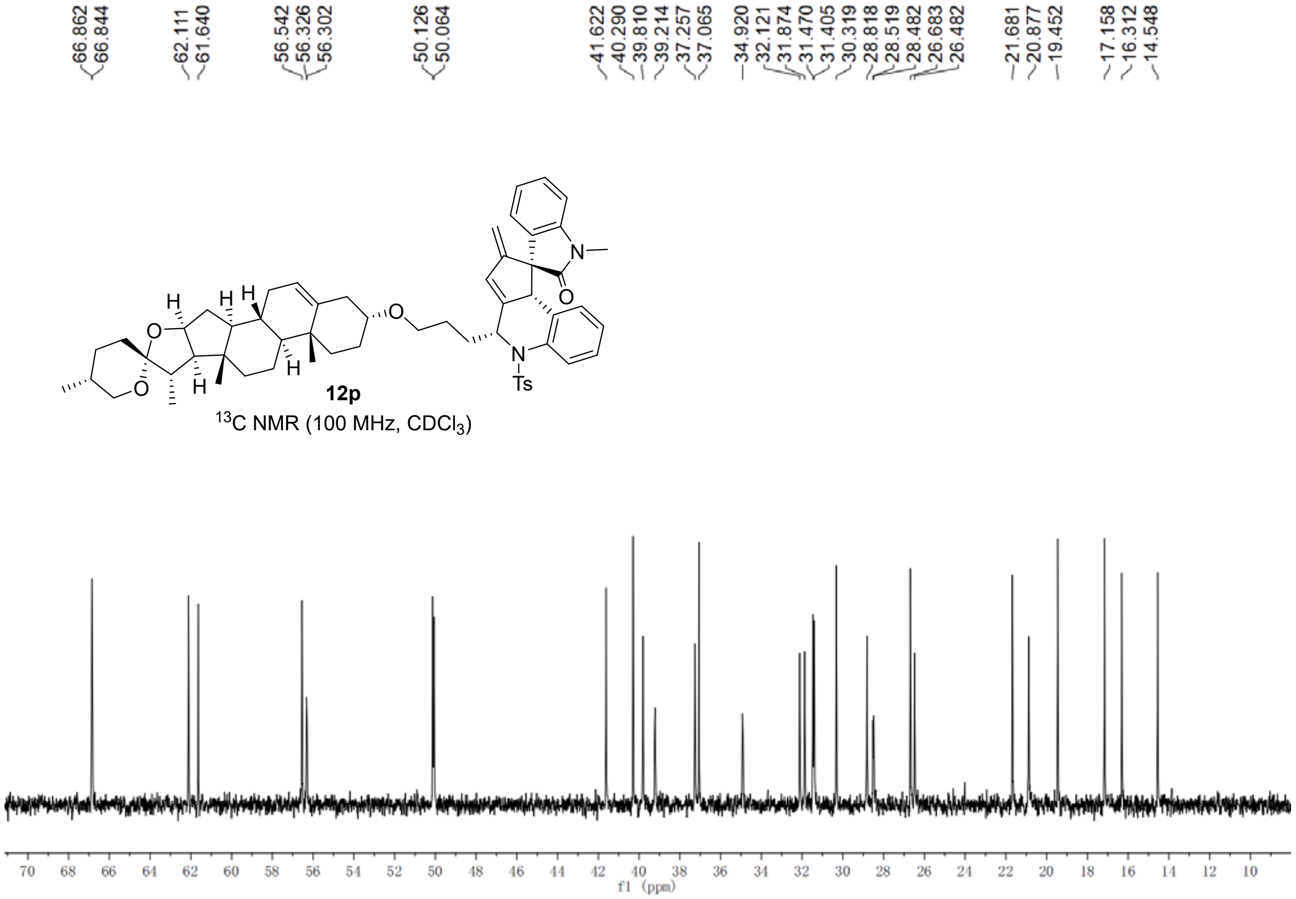
66.862
66.844
62.111
61.640
56.542
56.326
56.302
50.126
50.064
41.622
40.290
39.810
39.214
37.257
37.065
34.920
32.121
31.874
31.470
31.405
30.319
28.818
28.519
28.482
26.683
26.482
21.681
20.877
19.452
17.158
16.312
14.548
12p
^{13}C NMR (100 MHz, $CDCl_3$)
f1 (ppm)

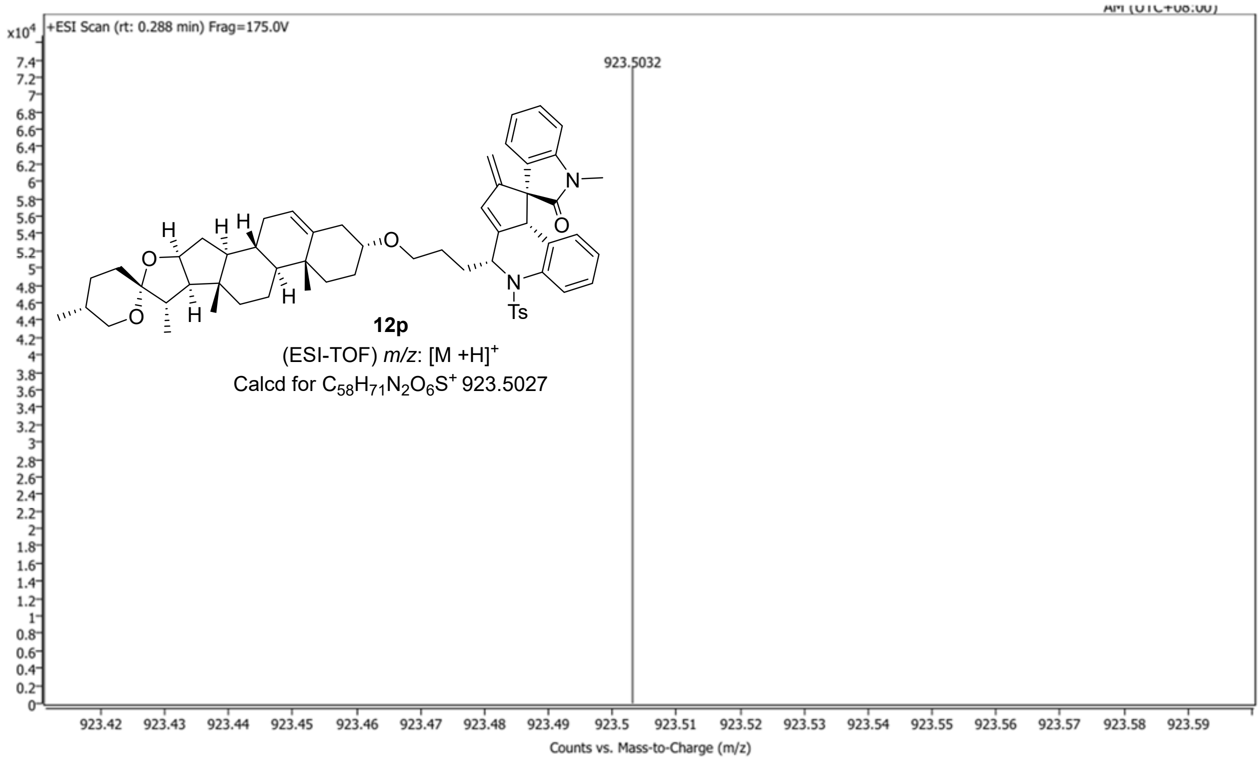
+ESI Scan (rt: 0.288 min) Frag=175.0V
x10^4
923.5032
12p
(ESI-TOF) m/z: [M +H]+
Calcd for $C_{58}H_{71}N_2O_6S^+$ 923.5027
Counts vs. Mass-to-Charge (m/z)

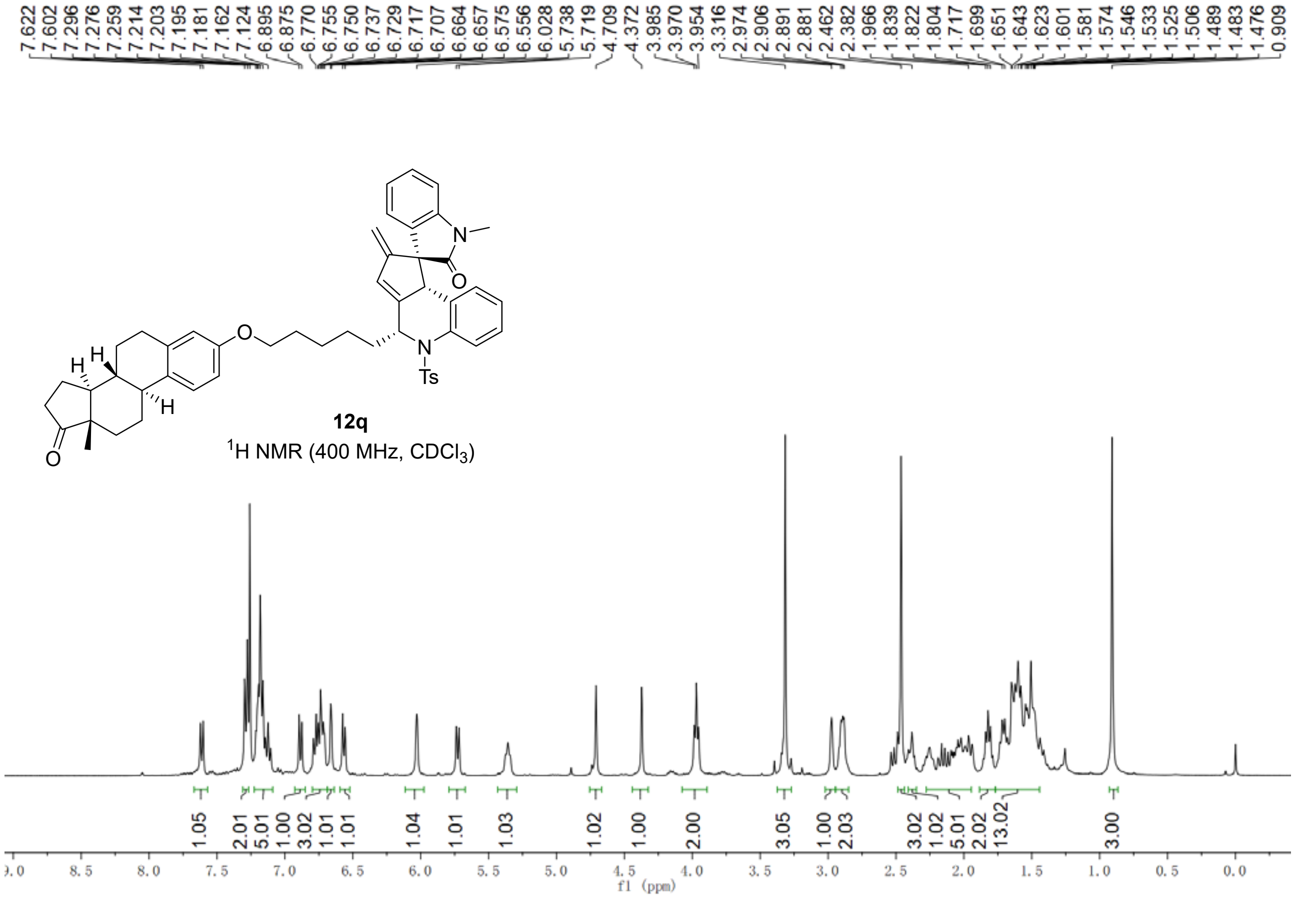
7.622
7.602
7.296
7.276
7.259
7.214
7.203
7.195
7.181
7.162
7.124
6.895
6.875
6.770
6.755
6.750
6.737
6.729
6.717
6.707
6.664
6.657
6.575
6.556
6.028
5.738
5.719
4.709
4.372
3.985
3.970
3.954
3.316
2.974
2.906
2.891
2.881
2.462
2.382
1.966
1.839
1.822
1.804
1.717
1.699
1.651
1.643
1.623
1.601
1.581
1.574
1.546
1.533
1.525
1.506
1.489
1.483
1.476
0.909
N
O
O
N
Ts
H
H
H
O
12q
^{1}H NMR (400 MHz, CDCl$_3$)
1.05
2.01
5.01
1.00
3.02
1.01
1.01
1.04
1.01
1.03
1.02
1.00
2.00
3.05
1.00
2.03
3.02
1.02
5.01
2.02
13.02
3.00
f1 (ppm)

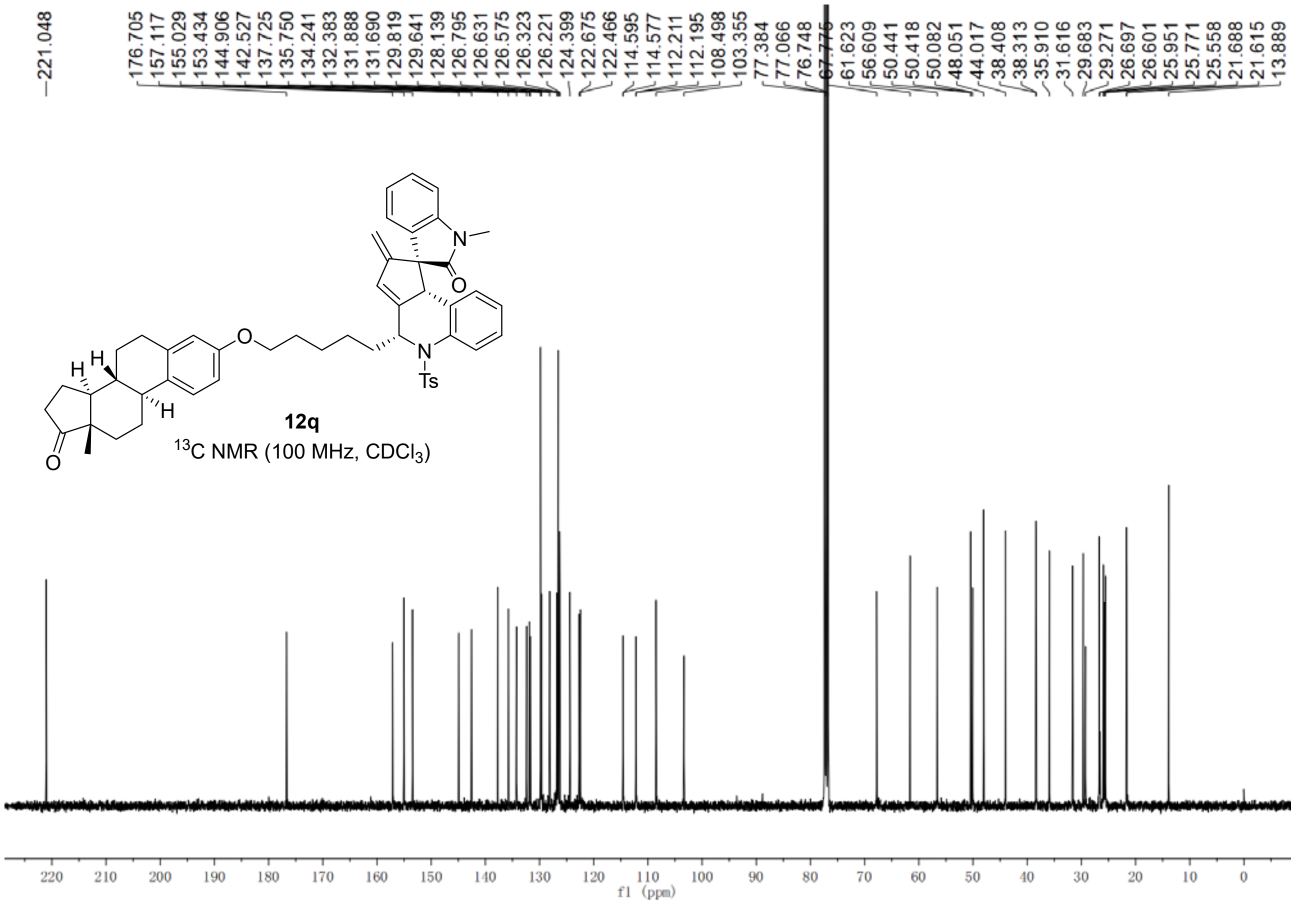
221.048
176.705
157.117
155.029
153.434
144.906
142.527
137.725
135.750
134.241
132.383
131.888
131.690
129.819
129.641
128.139
126.795
126.631
126.575
126.323
126.221
124.399
122.675
122.466
114.595
114.577
112.211
112.195
108.498
103.355
77.384
77.066
76.748
67.775
61.623
56.609
50.441
50.418
50.082
48.051
44.017
38.408
38.313
35.910
31.616
29.683
29.271
26.697
26.601
25.951
25.771
25.558
21.688
21.615
13.889
12q
^{13}C NMR (100 MHz, CDCl$_3$)
f1 (ppm)

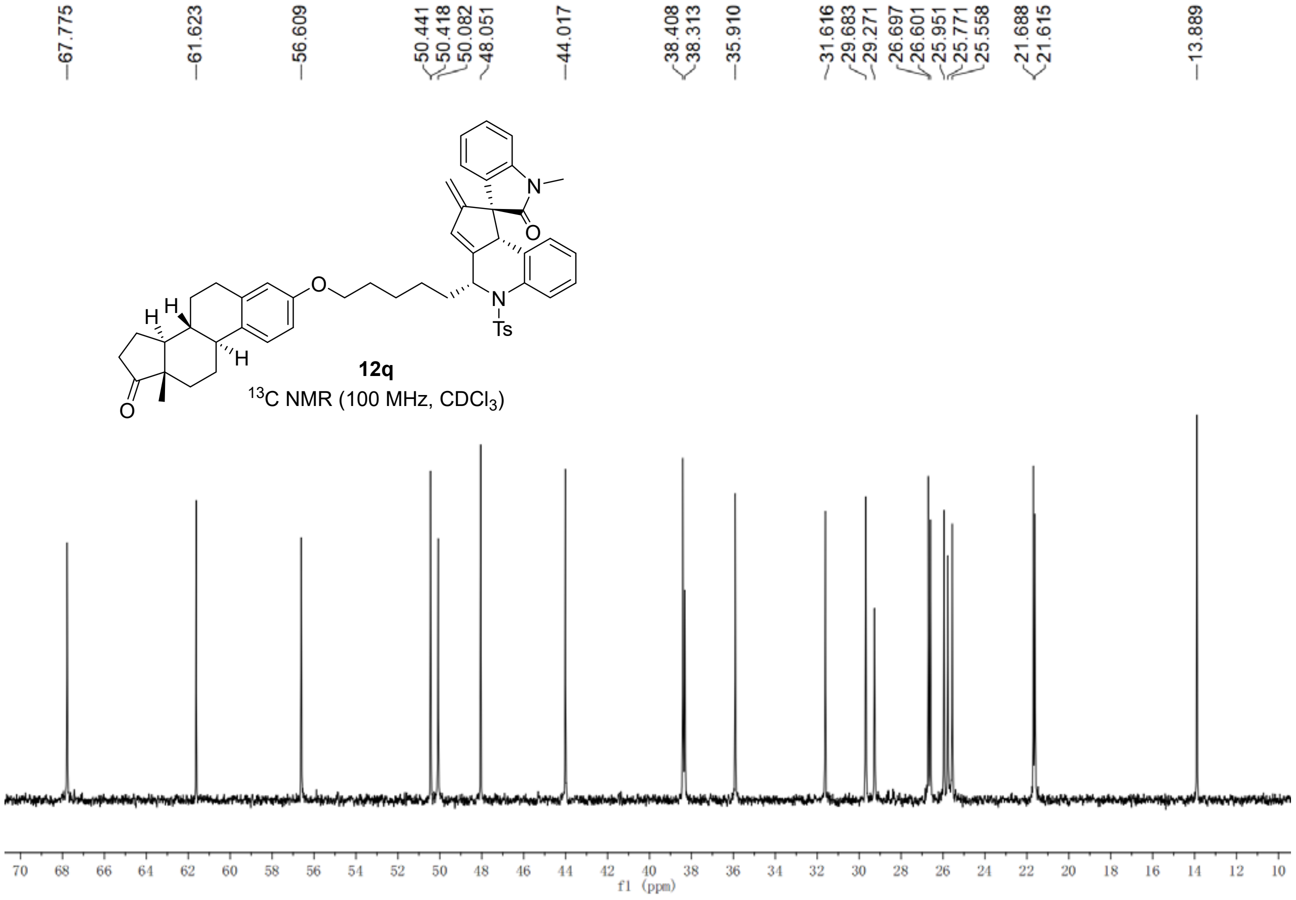

67.775
61.623
56.609
50.441
50.418
50.082
48.051
44.017
38.408
38.313
35.910
31.616
29.683
29.271
26.697
26.601
25.951
25.771
25.558
21.688
21.615
13.889
12q
^{13}C NMR (100 MHz, CDCl$_3$)
f1 (ppm)

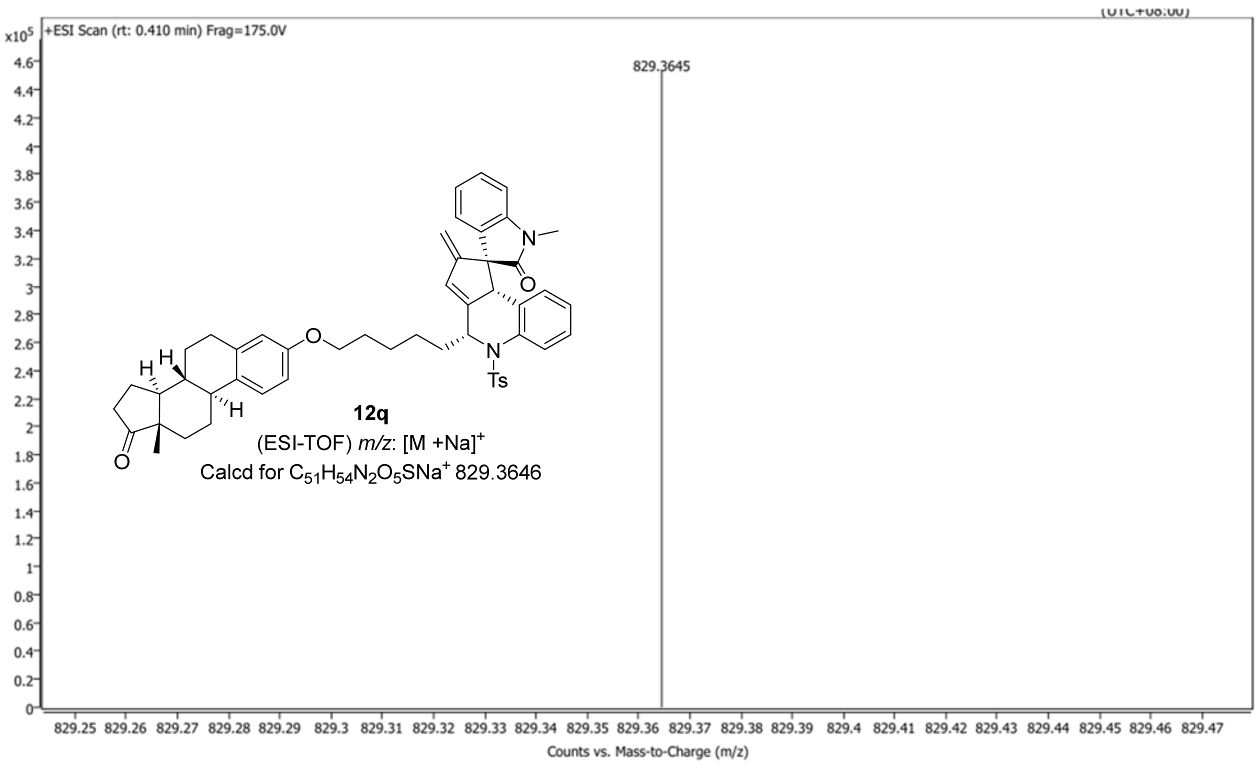

+ESI Scan (rt: 0.410 min) Frag=175.0V
x10^5
829.3645
12q
(ESI-TOF) m/z: [M +Na]$^+$
Calcd for $C_{51}H_{54}N_2O_5SNa^+$ 829.3646
Counts vs. Mass-to-Charge (m/z)

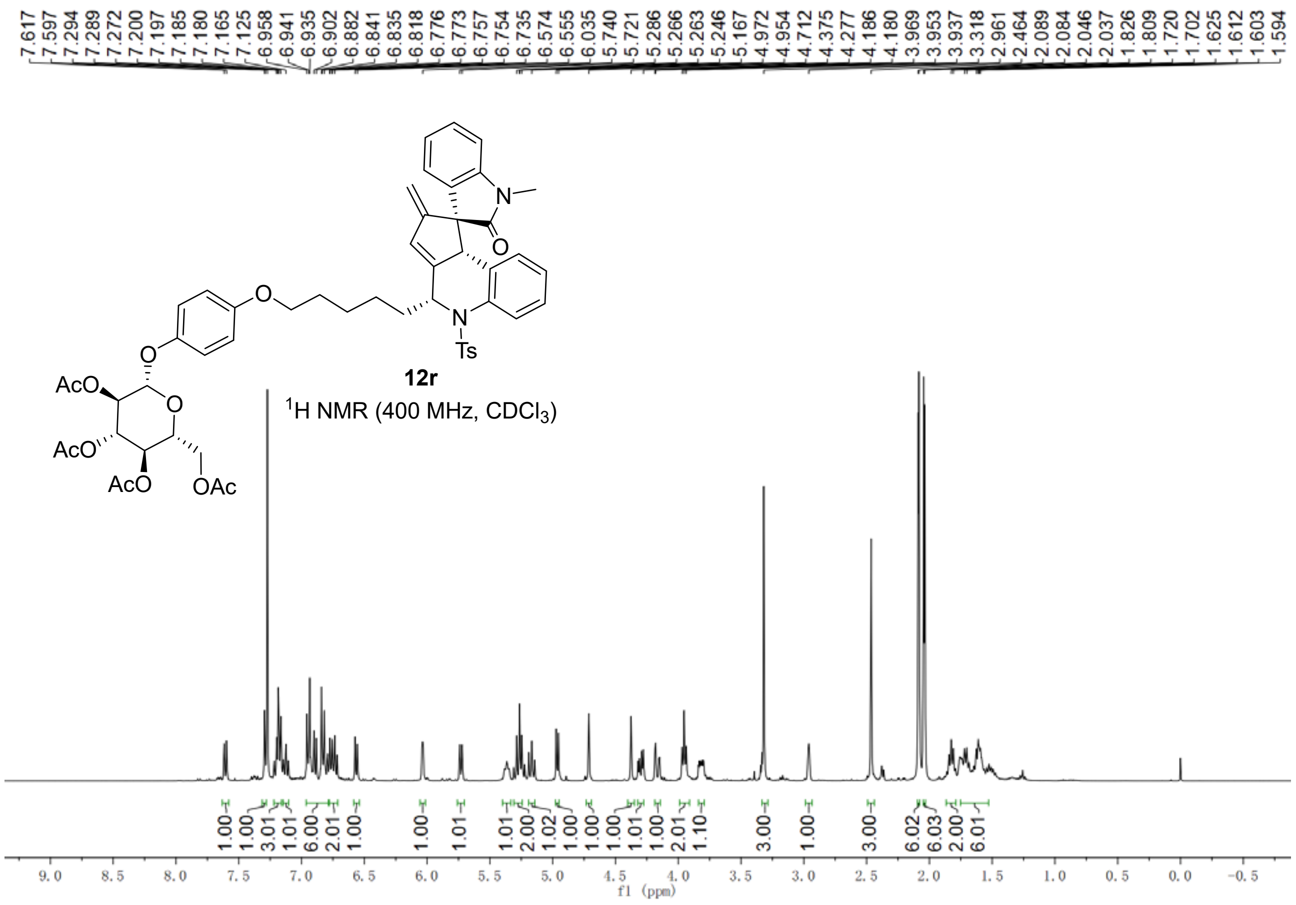
7.617 7.597 7.294 7.289 7.272 7.200 7.197 7.185 7.180 7.165 7.125 6.958 6.941 6.935 6.902 6.882 6.841 6.835 6.818 6.776 6.773 6.757 6.754 6.735 6.574 6.555 6.035 5.740 5.721 5.286 5.266 5.263 5.246 5.167 4.972 4.954 4.712 4.375 4.277 4.186 4.180 3.969 3.953 3.937 3.318 2.961 2.464 2.089 2.084 2.046 2.037 1.826 1.809 1.720 1.702 1.625 1.612 1.603 1.594
12r
1H NMR (400 MHz, CDCl3)
1.00 1.00 3.01 1.01 6.00 2.01 1.00 1.00 1.01 1.01 2.00 1.02 1.00 1.00 1.00 1.01 1.00 2.01 1.10 3.00 1.00 3.00 6.02 6.03 2.00 6.01
f1 (ppm)

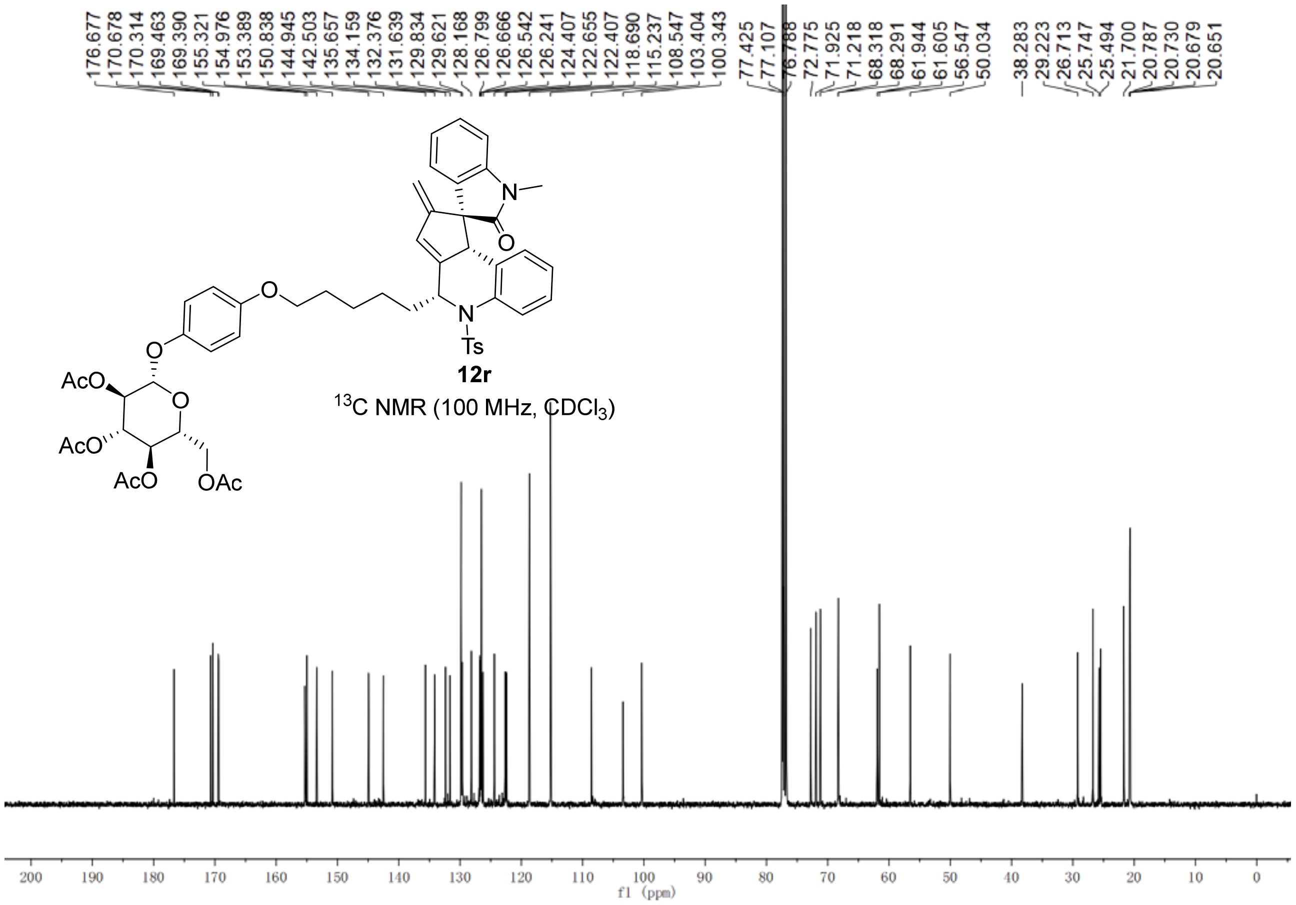
176.677 170.678 170.314 169.463 169.390 155.321 154.976 153.389 150.838 144.945 142.503 135.657 134.159 132.376 131.639 129.834 129.621 128.168 126.799 126.666 126.542 126.241 124.407 122.655 122.407 118.690 115.237 108.547 103.404 100.343 77.425 77.107 76.788 72.775 71.925 71.218 68.318 68.291 61.944 61.605 56.547 50.034 38.283 29.223 26.713 25.747 25.494 21.700 20.787 20.730 20.679 20.651
12r
13C NMR (100 MHz, CDCl3)
f1 (ppm)

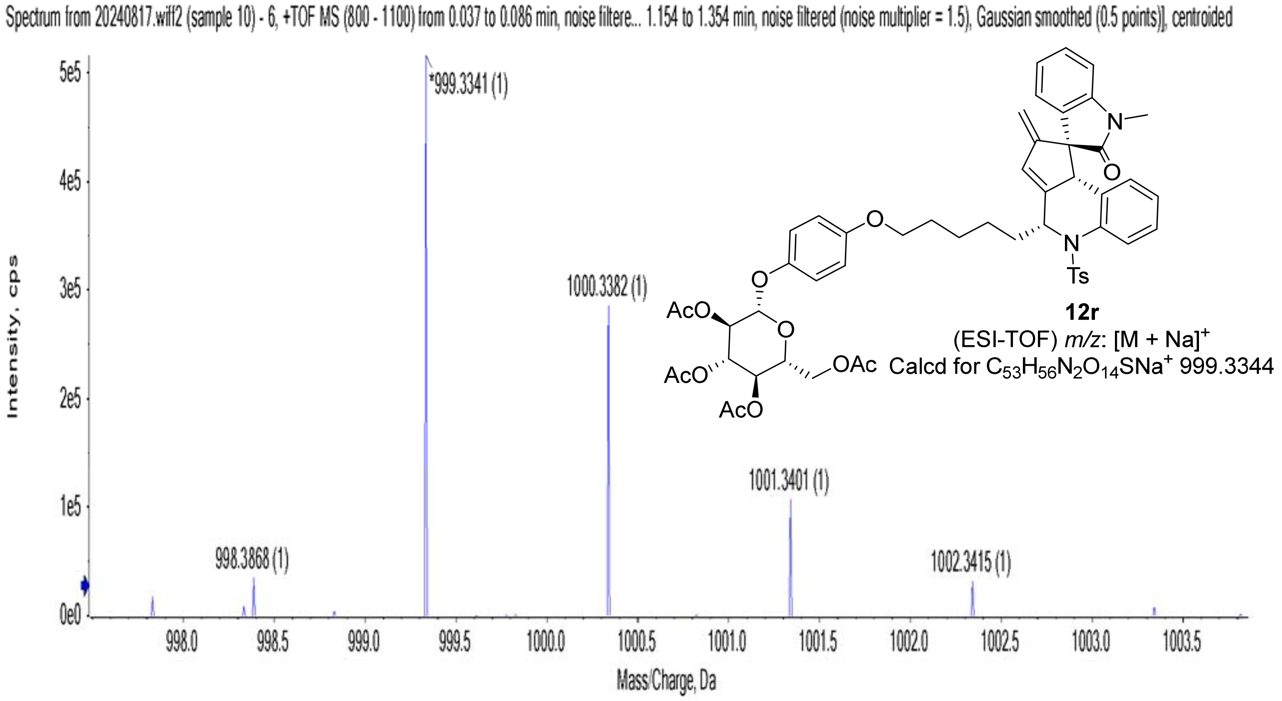

Spectrum from 20240817.wiff2 (sample 10) - 6, +TOF MS (800 - 1100) from 0.037 to 0.086 min, noise filtere... 1.154 to 1.354 min, noise filtered (noise multiplier = 1.5), Gaussian smoothed (0.5 points)], centroided
*999.3341 (1)
1000.3382 (1)
1001.3401 (1)
998.3868 (1)
1002.3415 (1)
Intensity, cps
Mass/Charge, Da
12r
(ESI-TOF) m/z: [M + Na]+
Calcd for C53H56N2O14SNa+ 999.3344

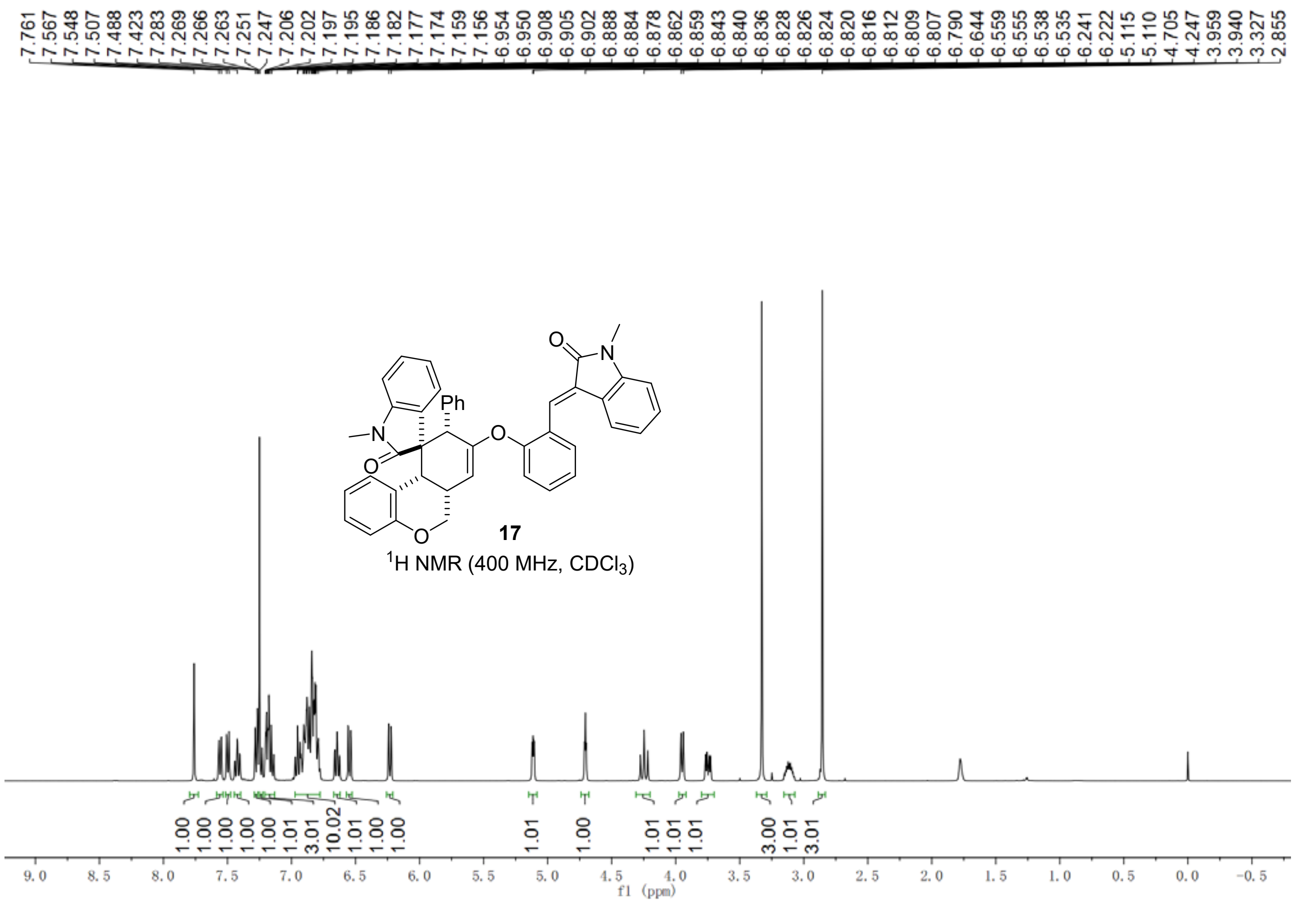
7.761 7.567 7.548 7.507 7.488 7.423 7.283 7.269 7.266 7.263 7.251 7.247 7.206 7.202 7.197 7.195 7.186 7.182 7.177 7.174 7.159 7.156 6.954 6.950 6.908 6.905 6.902 6.888 6.884 6.878 6.862 6.859 6.843 6.840 6.836 6.828 6.826 6.824 6.820 6.816 6.812 6.809 6.807 6.790 6.644 6.559 6.555 6.538 6.535 6.241 6.222 5.115 5.110 4.705 4.247 3.959 3.940 3.327 2.855
Ph
17
1H NMR (400 MHz, CDCl3)
1.00 1.00 1.00 1.00 1.00 1.01 3.01 10.02 1.01 1.00 1.00 1.01 1.00 1.01 1.01 1.01 3.00 1.01 3.01
f1 (ppm)

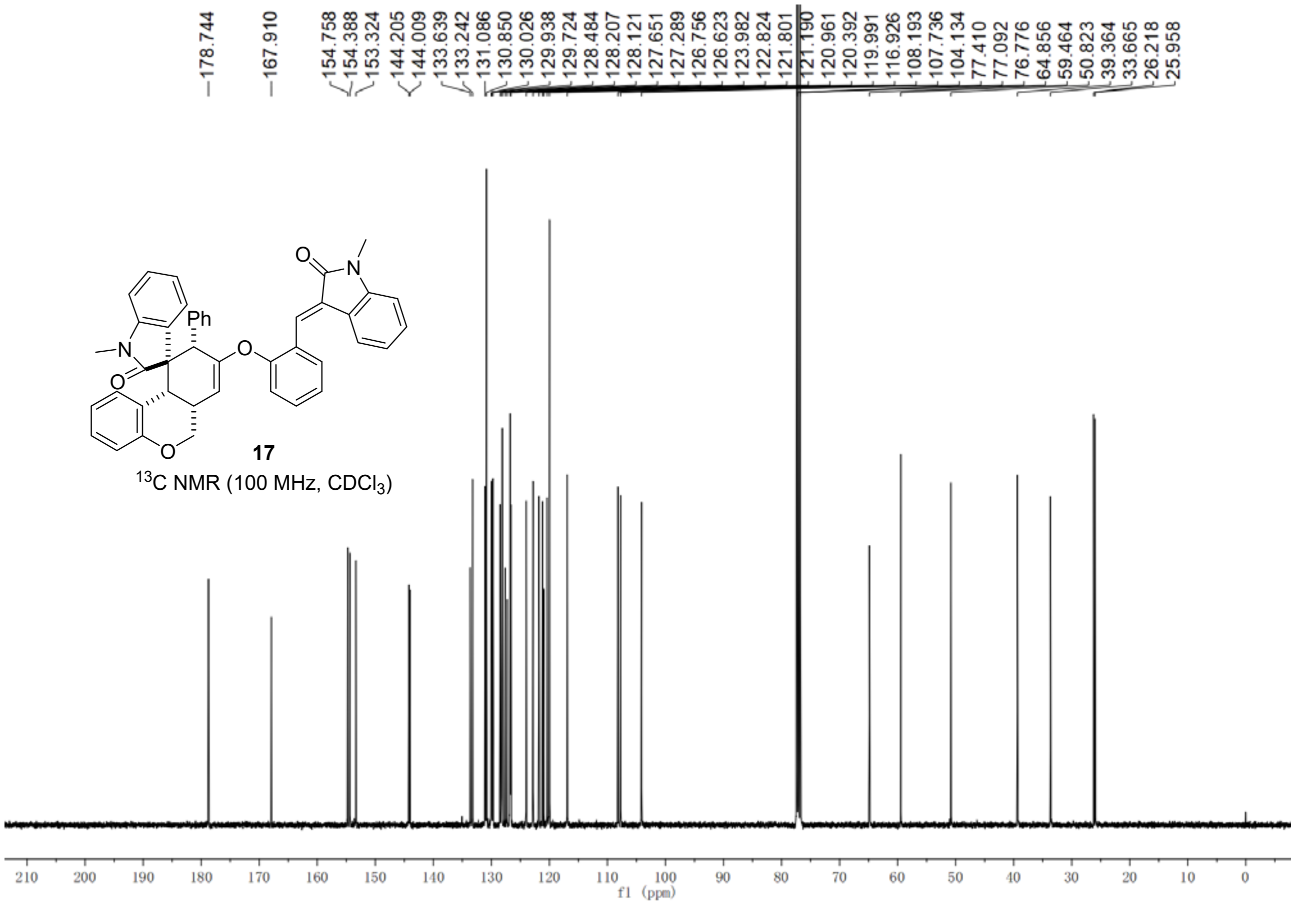
178.744 167.910 154.758 154.388 153.324 144.205 144.009 133.639 133.242 131.086 130.850 130.026 129.938 129.724 128.484 128.207 128.121 127.651 127.289 126.756 126.623 123.982 122.824 121.801 121.190 120.961 120.392 119.991 116.926 108.193 107.736 104.134 77.410 77.092 76.776 64.856 59.464 50.823 39.364 33.665 26.218 25.958
Ph
17
13C NMR (100 MHz, CDCl3)
f1 (ppm)

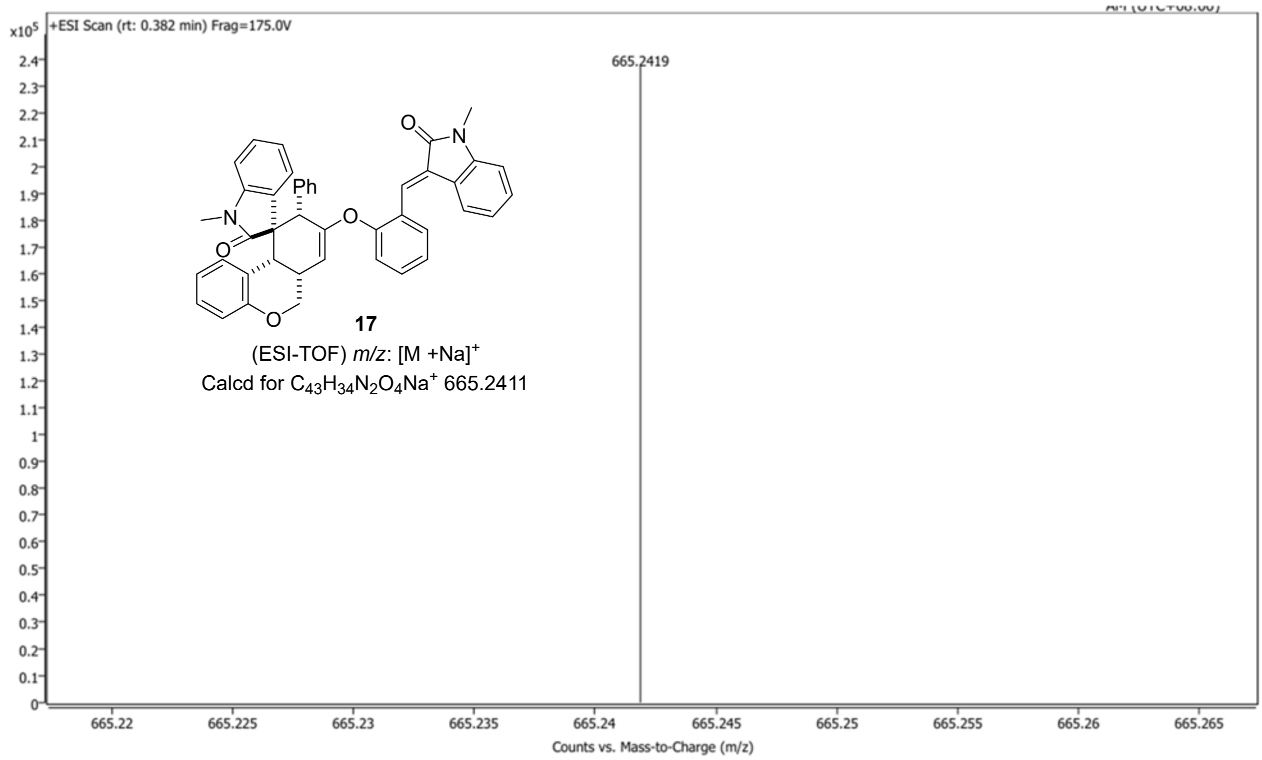

+ESI Scan (rt: 0.382 min) Frag=175.0V
x10^5
665.2419
Ph
17
(ESI-TOF) m/z: [M +Na]+
Calcd for C43H34N2O4Na+ 665.2411
Counts vs. Mass-to-Charge (m/z)

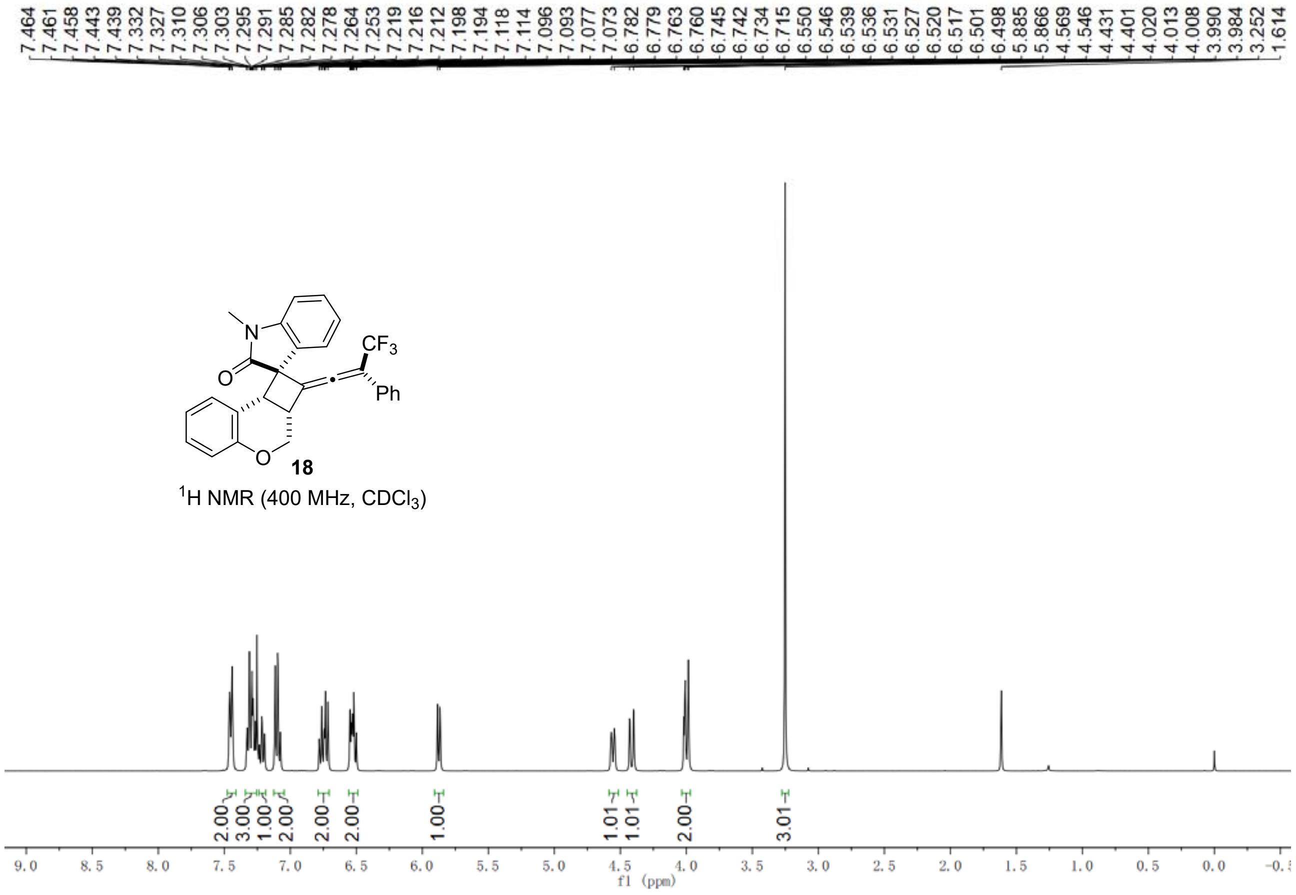
7.464 7.461 7.458 7.443 7.439 7.332 7.327 7.310 7.306 7.303 7.295 7.291 7.285 7.282 7.278 7.264 7.253 7.219 7.216 7.212 7.198 7.194 7.118 7.114 7.096 7.093 7.077 7.073 6.782 6.779 6.763 6.760 6.745 6.742 6.734 6.715 6.550 6.546 6.539 6.536 6.531 6.527 6.520 6.517 6.501 6.498 5.885 5.866 4.569 4.546 4.431 4.401 4.020 4.013 4.008 3.990 3.984 3.252 1.614
N
CF3
O
Ph
18
1H NMR (400 MHz, CDCl3)
2.00 3.00 1.00 2.00 2.00 2.00 1.00 1.01 1.01 2.00 3.01
9.0 8.5 8.0 7.5 7.0 6.5 6.0 5.5 5.0 4.5 4.0 3.5 3.0 2.5 2.0 1.5 1.0 0.5 0.0
f1 (ppm)

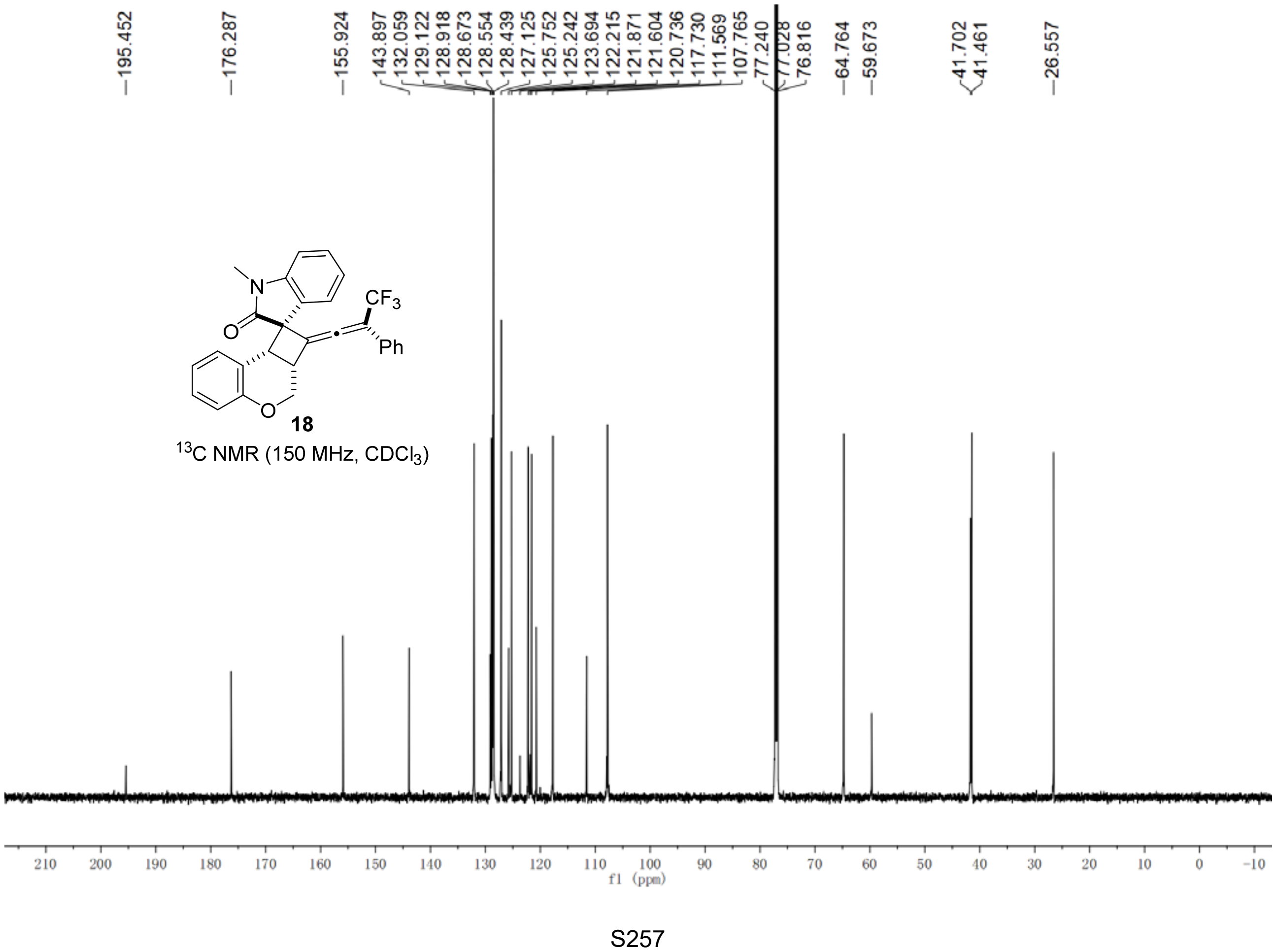
195.452 176.287 155.924 143.897 132.059 129.122 128.918 128.673 128.554 128.439 127.125 125.752 125.242 123.694 122.215 121.871 121.604 120.736 117.730 111.569 107.765 77.240 77.028 76.816 64.764 59.673 41.702 41.461 26.557
N
CF3
O
Ph
18
13C NMR (150 MHz, CDCl3)
210 200 190 180 170 160 150 140 130 120 110 100 90 80 70 60 50 40 30 20 10 0 −10
f1 (ppm)

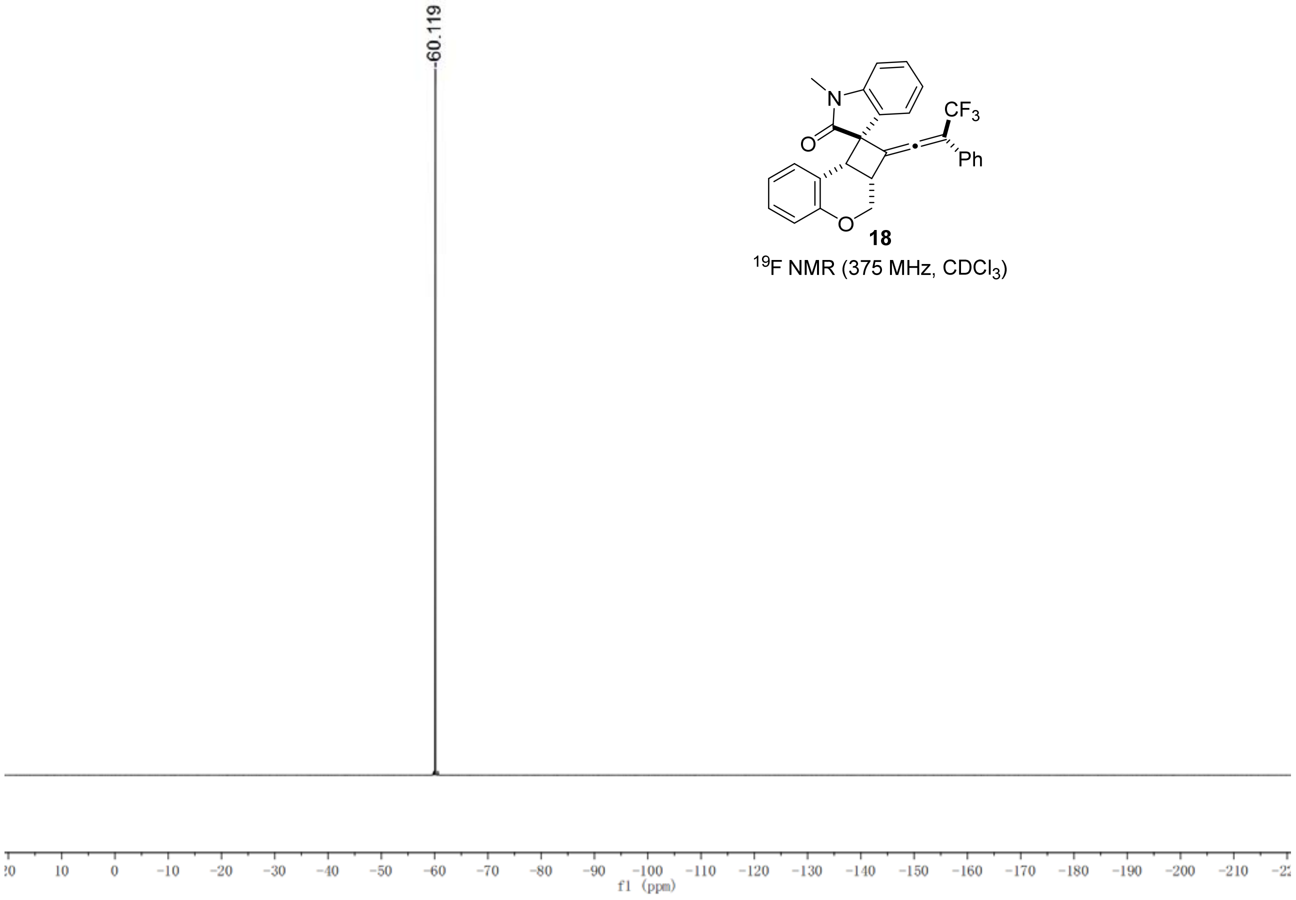

-60.119
CF3
Ph
N
O
O
18
19F NMR (375 MHz, CDCl3)
f1 (ppm)

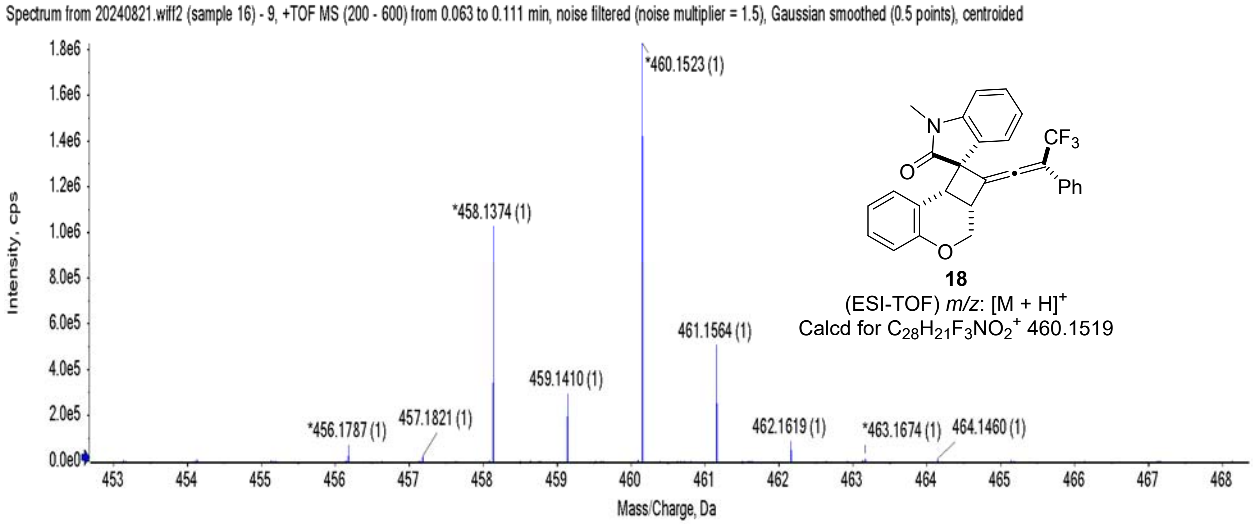

Spectrum from 20240821.wiff2 (sample 16) - 9, +TOF MS (200 - 600) from 0.063 to 0.111 min, noise filtered (noise multiplier = 1.5), Gaussian smoothed (0.5 points), centroided
Intensity, cps
Mass/Charge, Da
*460.1523 (1)
*458.1374 (1)
461.1564 (1)
459.1410 (1)
457.1821 (1)
*456.1787 (1)
462.1619 (1)
*463.1674 (1)
464.1460 (1)
18
(ESI-TOF) m/z: [M + H]+
Calcd for C28H21F3NO2+ 460.1519

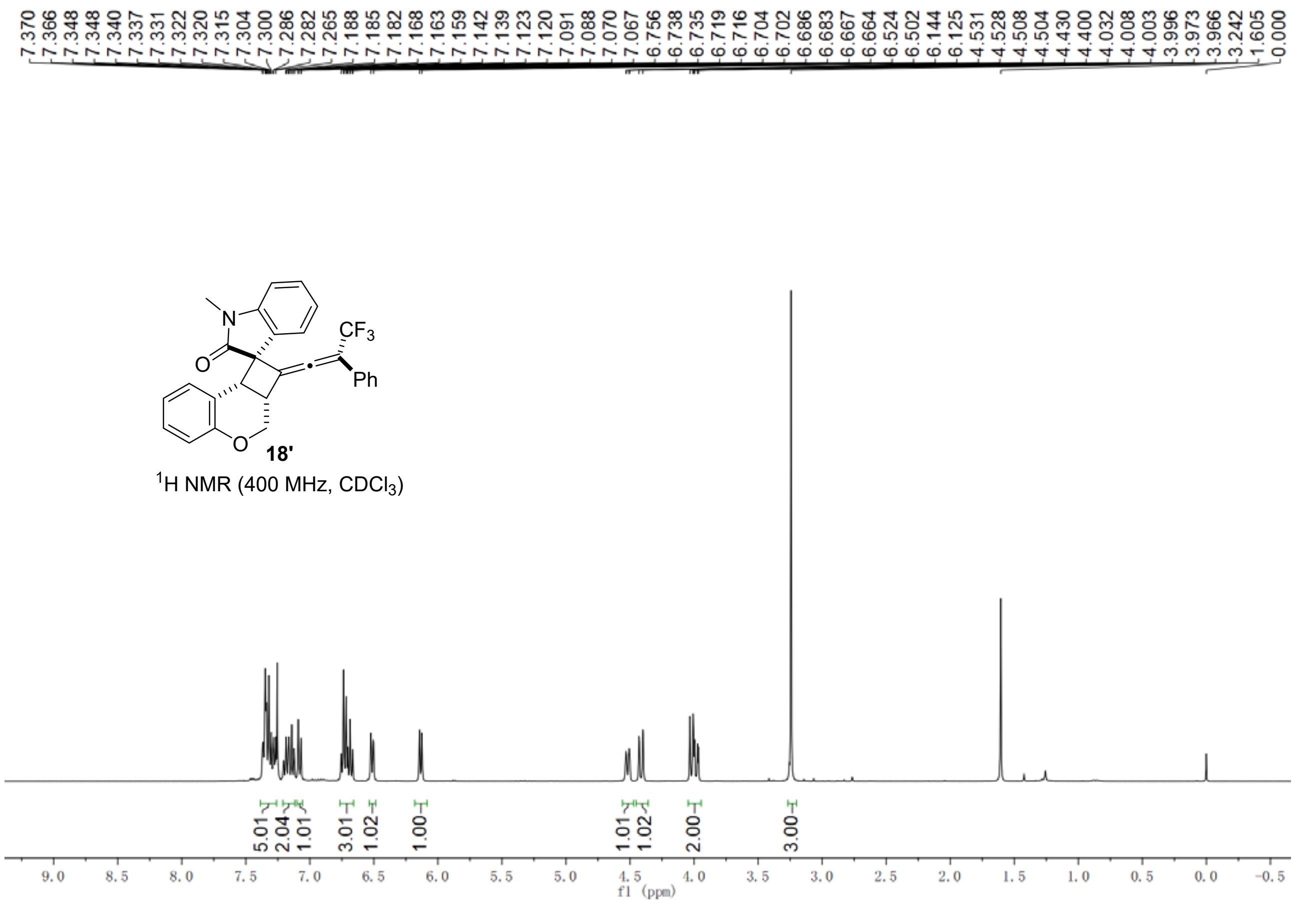

^{1}H NMR (400 MHz, $CDCl_3$)

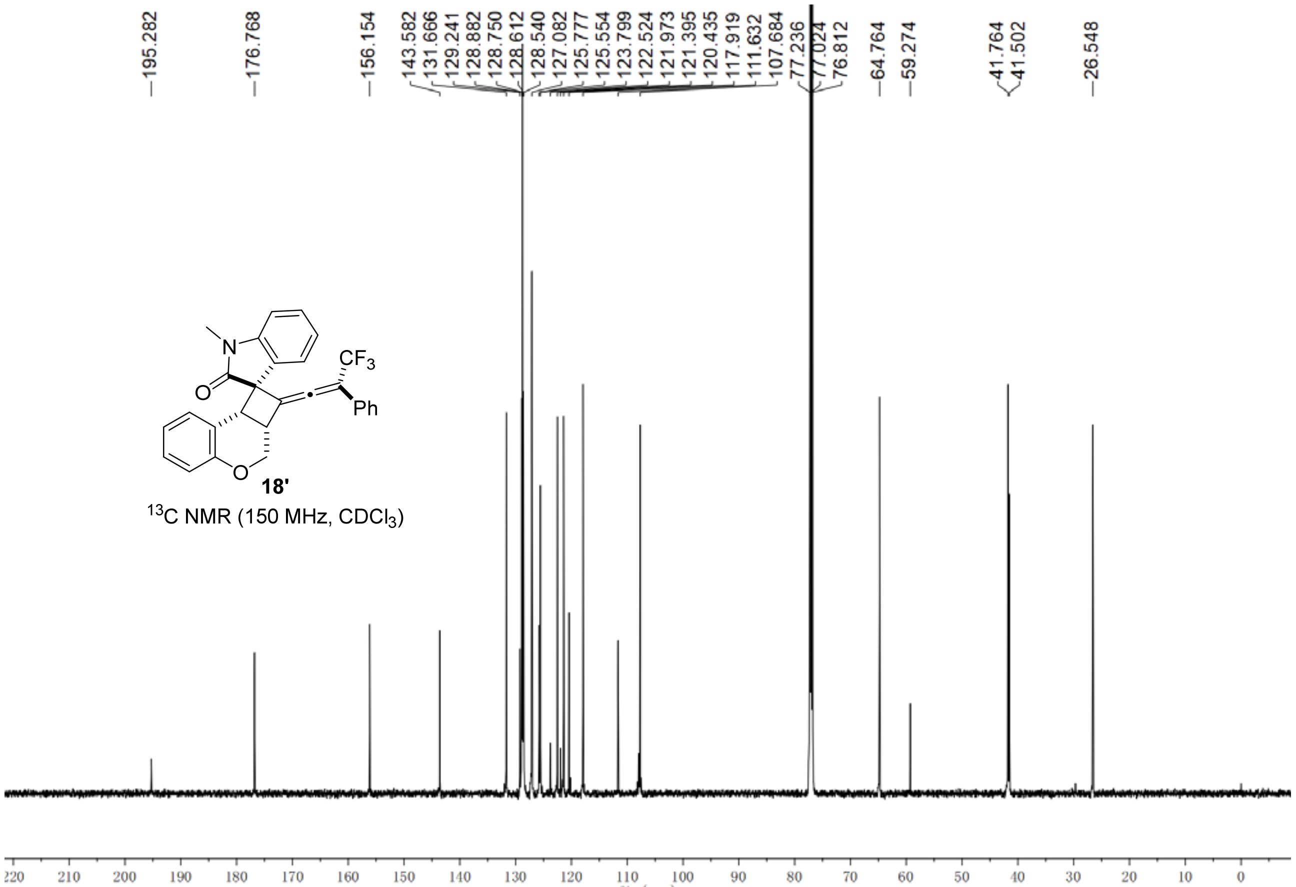

^{13}C NMR (150 MHz, $CDCl_3$)

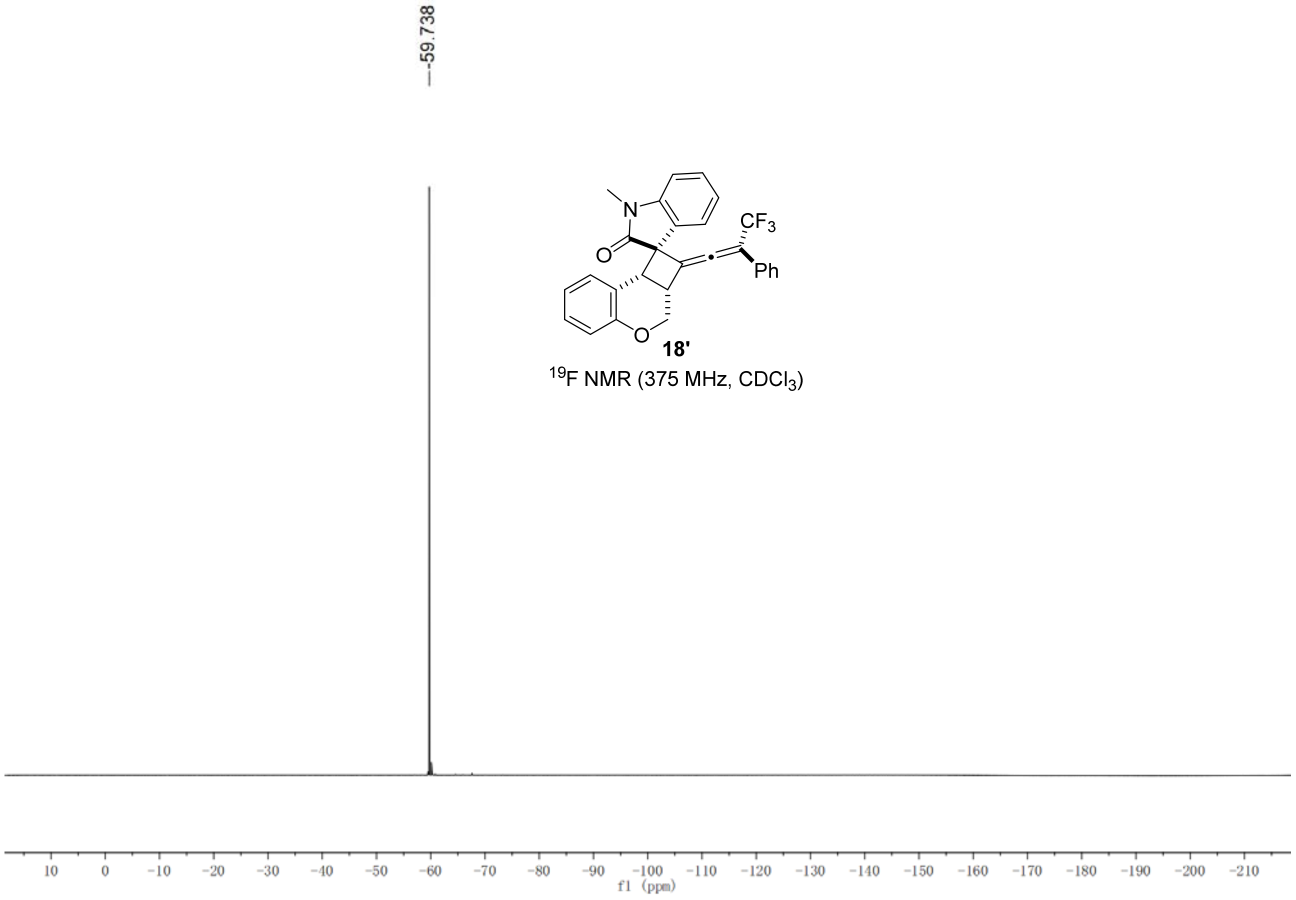
-59.738
CF3
Ph
N
O
O
18'
19F NMR (375 MHz, CDCl3)
10 0 −10 −20 −30 −40 −50 −60 −70 −80 −90 −100 −110 −120 −130 −140 −150 −160 −170 −180 −190 −200 −210
f1 (ppm)

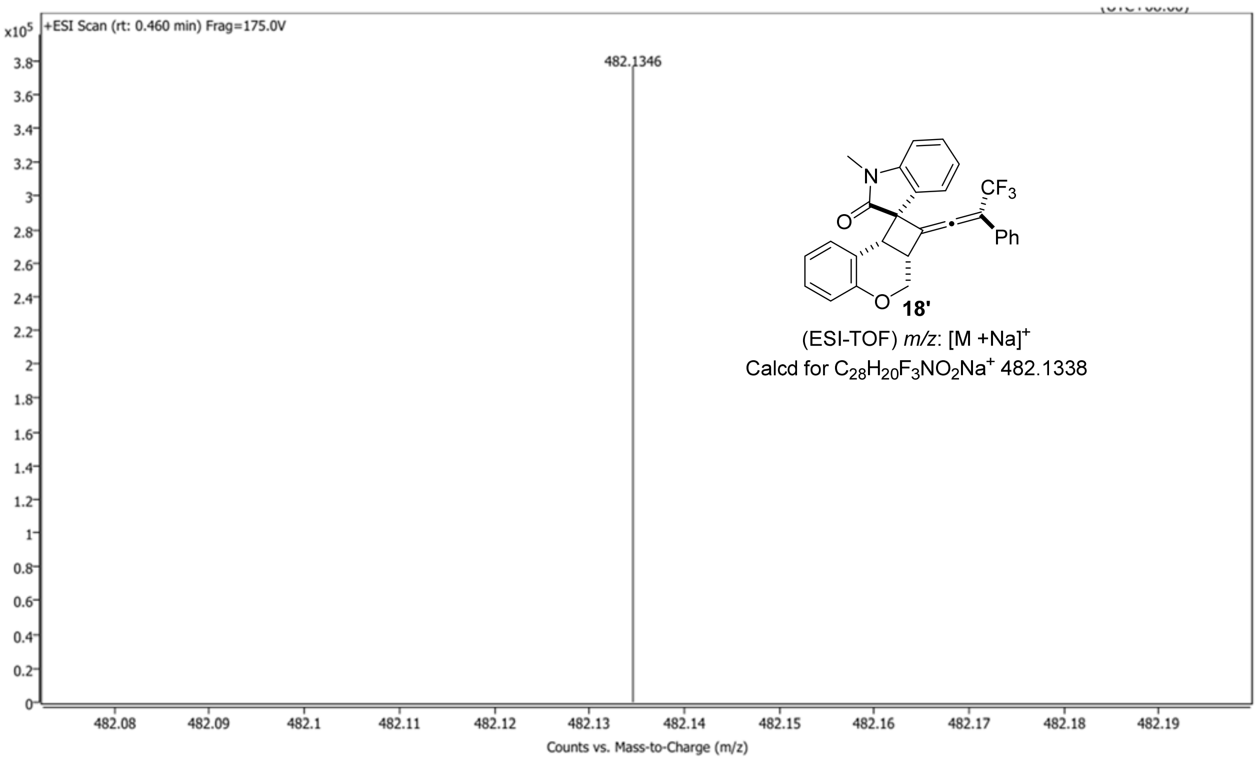
x10^5
+ESI Scan (rt: 0.460 min) Frag=175.0V
482.1346
3.8 3.6 3.4 3.2 3 2.8 2.6 2.4 2.2 2 1.8 1.6 1.4 1.2 1 0.8 0.6 0.4 0.2 0
CF3
Ph
N
O
O
18'
(ESI-TOF) m/z: [M +Na]+
Calcd for C28H20F3NO2Na+ 482.1338
482.08 482.09 482.1 482.11 482.12 482.13 482.14 482.15 482.16 482.17 482.18 482.19
Counts vs. Mass-to-Charge (m/z)

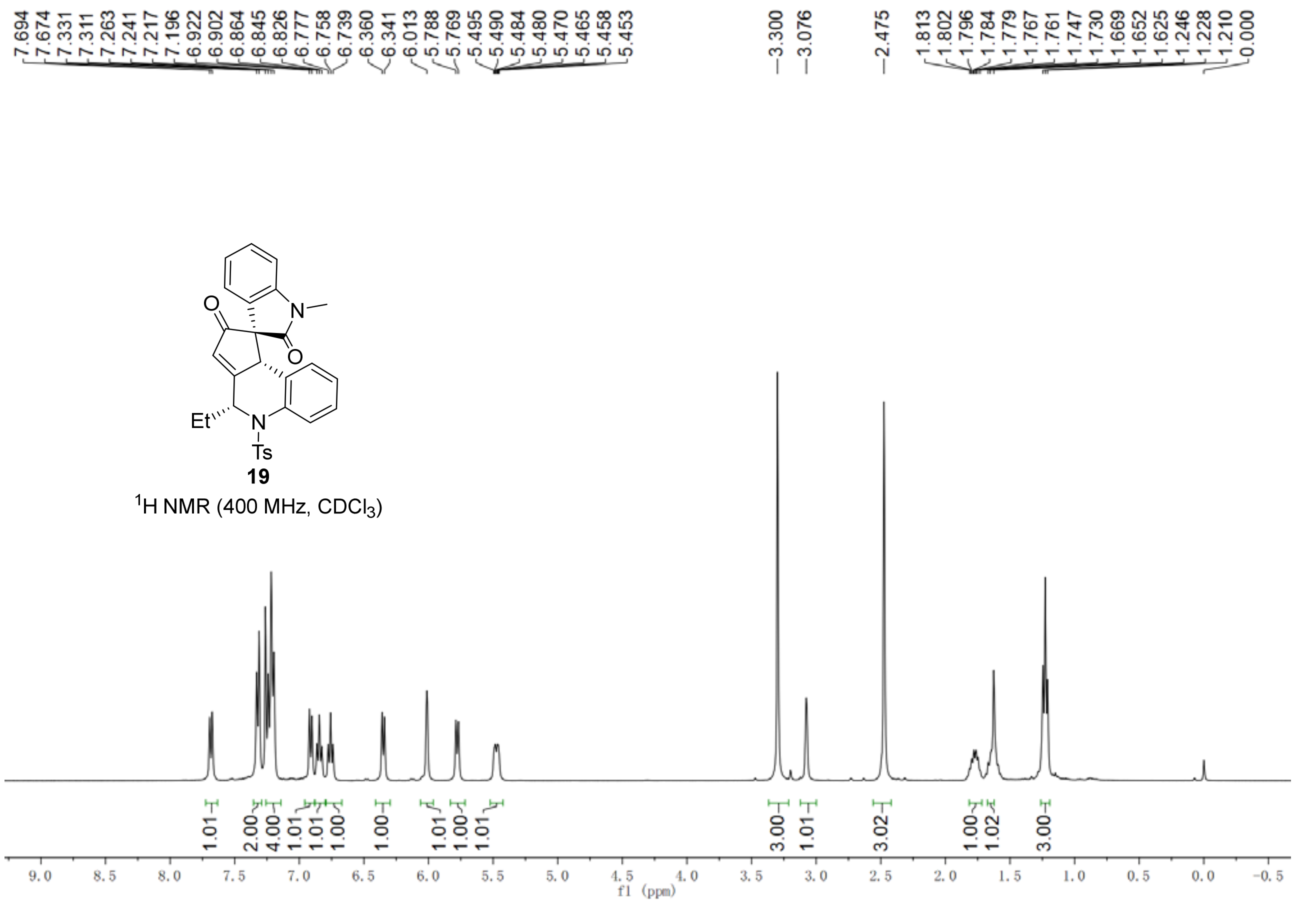
7.694
7.674
7.331
7.311
7.263
7.241
7.217
7.196
6.922
6.902
6.864
6.845
6.826
6.777
6.758
6.739
6.360
6.341
6.013
5.788
5.769
5.495
5.490
5.484
5.480
5.470
5.465
5.458
5.453
3.300
3.076
2.475
1.813
1.802
1.796
1.784
1.779
1.767
1.761
1.747
1.730
1.669
1.652
1.625
1.246
1.228
1.210
0.000
O
N
O
Et
N
Ts
19
1H NMR (400 MHz, CDCl3)
1.01
2.00
4.00
1.01
1.01
1.00
1.00
1.01
1.00
1.01
3.00
1.01
3.02
1.00
1.02
3.00
f1 (ppm)

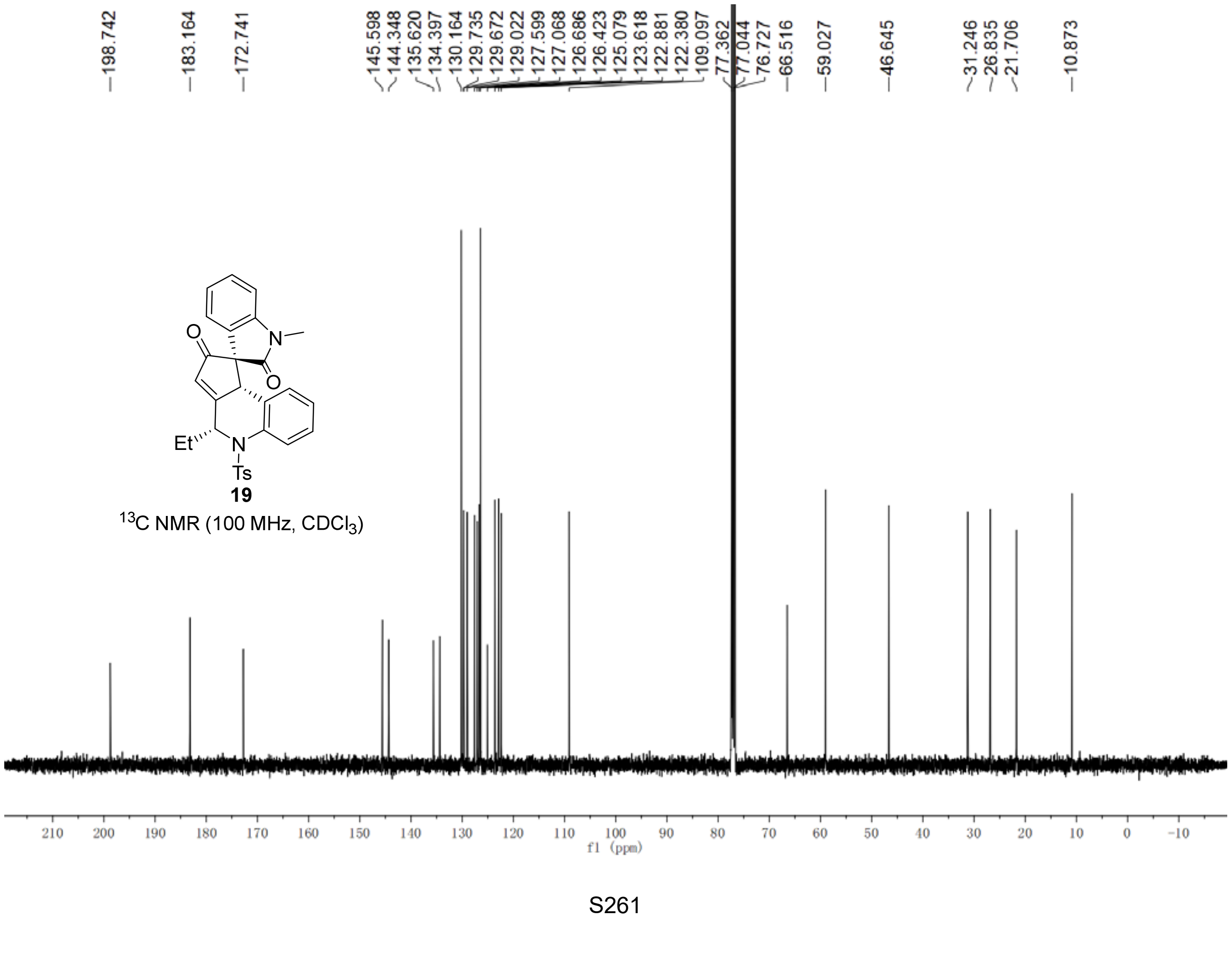
198.742
183.164
172.741
145.598
144.348
135.620
134.397
130.164
129.735
129.672
129.022
127.599
127.068
126.686
126.423
125.079
123.618
122.881
122.380
109.097
77.362
77.044
76.727
66.516
59.027
46.645
31.246
26.835
21.706
10.873
O
N
O
Et
N
Ts
19
13C NMR (100 MHz, CDCl3)
f1 (ppm)

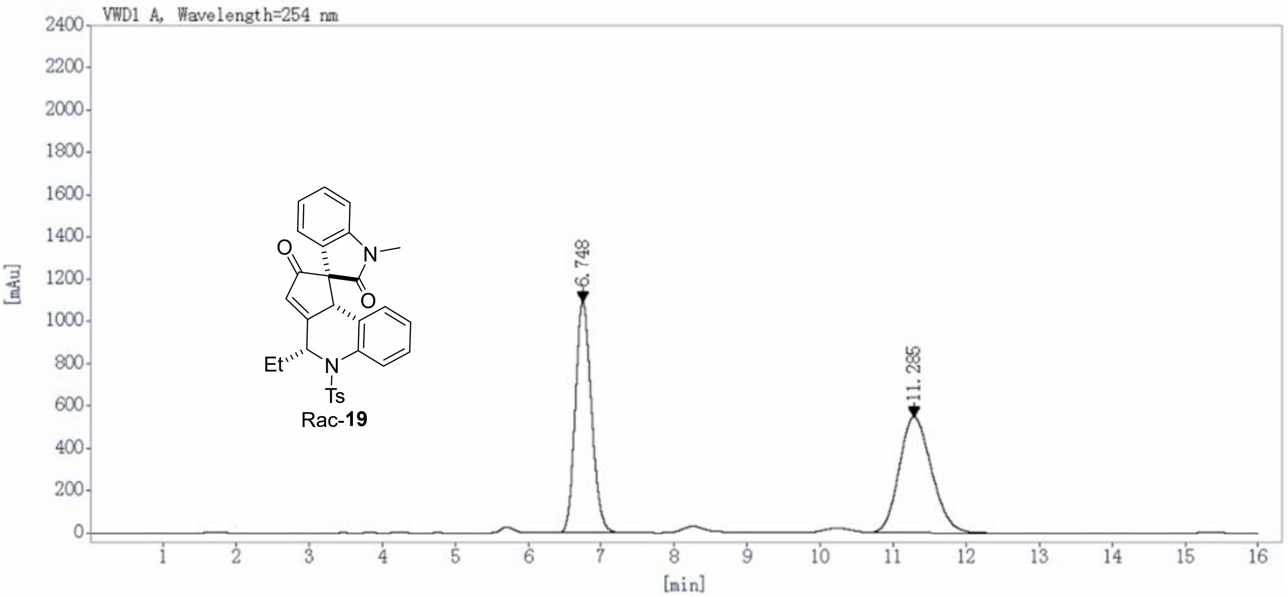

Ret Time [min]	Peak Type	Width [min]	Height [mAU]	Area [mAU*s]	Area [%]
6.748	BBA	0.24	1091.5602	16755.7148	50.0150
11.285	BBA	0.48	545.9031	16745.6582	49.9850
			Totals:	33501.3730	100.0000

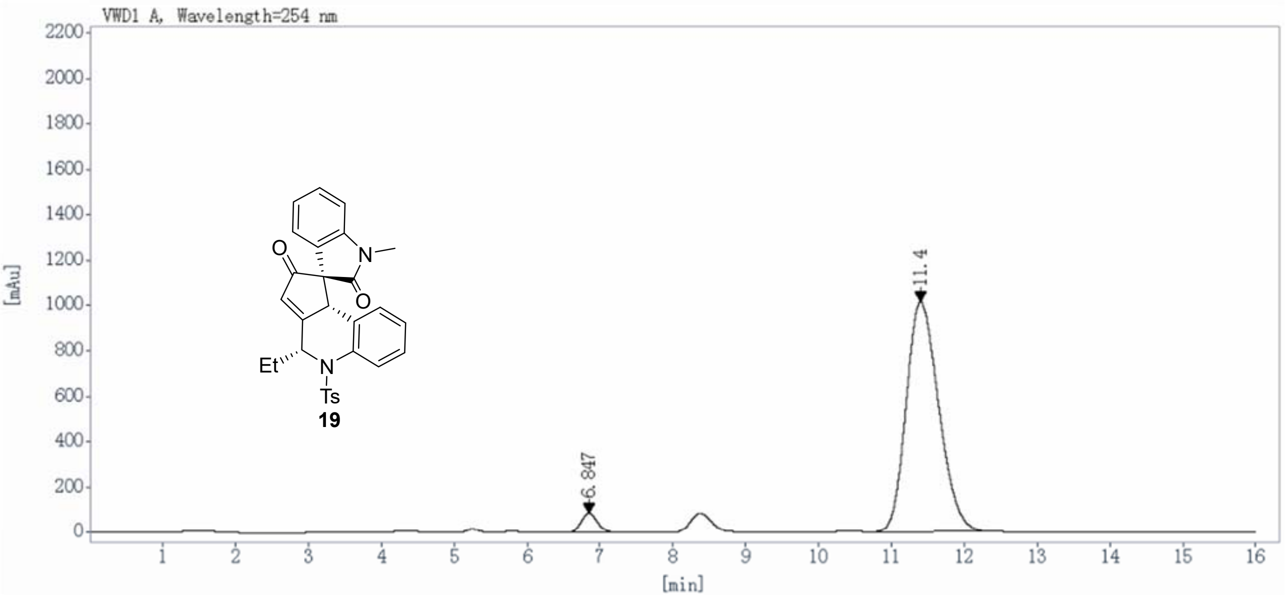

Ret Time [min]	Peak Type	Width [min]	Height [mAU]	Area [mAU*s]	Area [%]
6.847	BBA	0.24	85.6540	1293.7821	3.9508
11.400	BBA	0.48	1013.0538	31453.7012	96.0492
			Totals:	32747.4833	100.0000

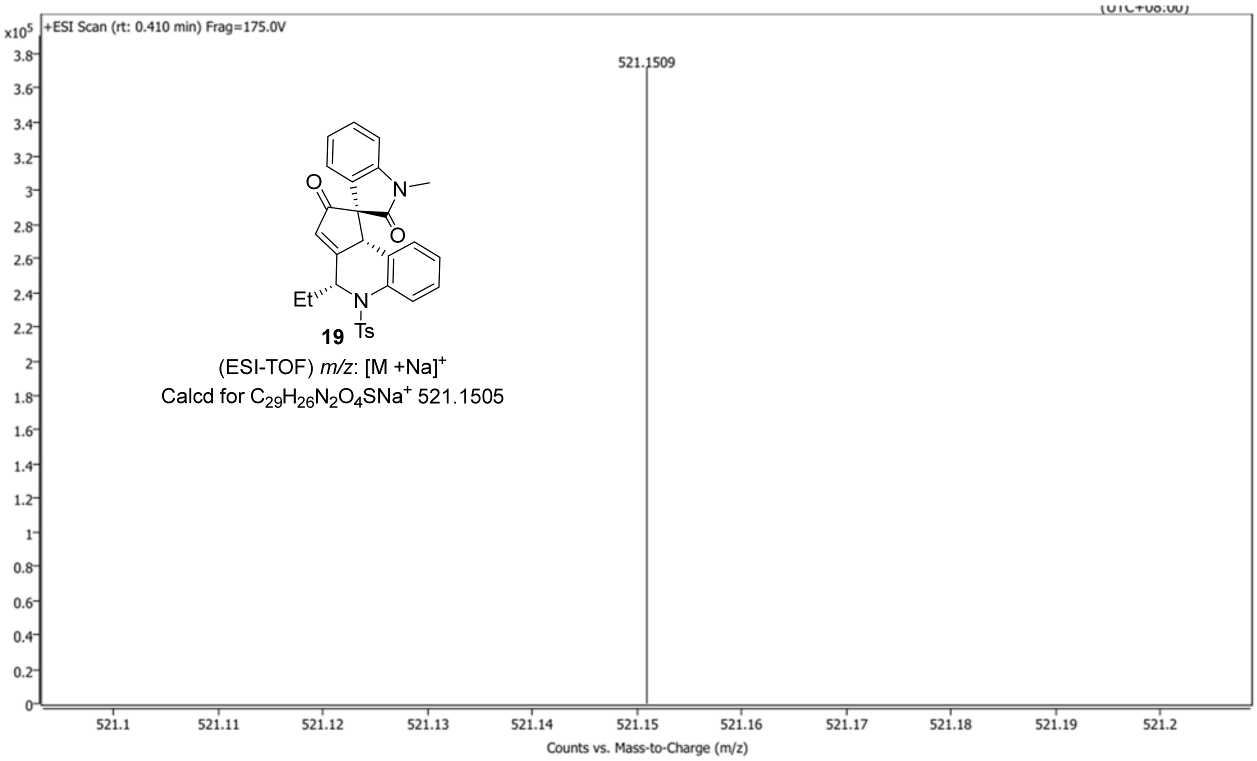

(UTC+08:00)
x10^5
+ESI Scan (rt: 0.410 min) Frag=175.0V
521.1509
O
N
O
Et
N
Ts
19
(ESI-TOF) m/z: [M +Na]+
Calcd for C29H26N2O4SNa+ 521.1505
Counts vs. Mass-to-Charge (m/z)

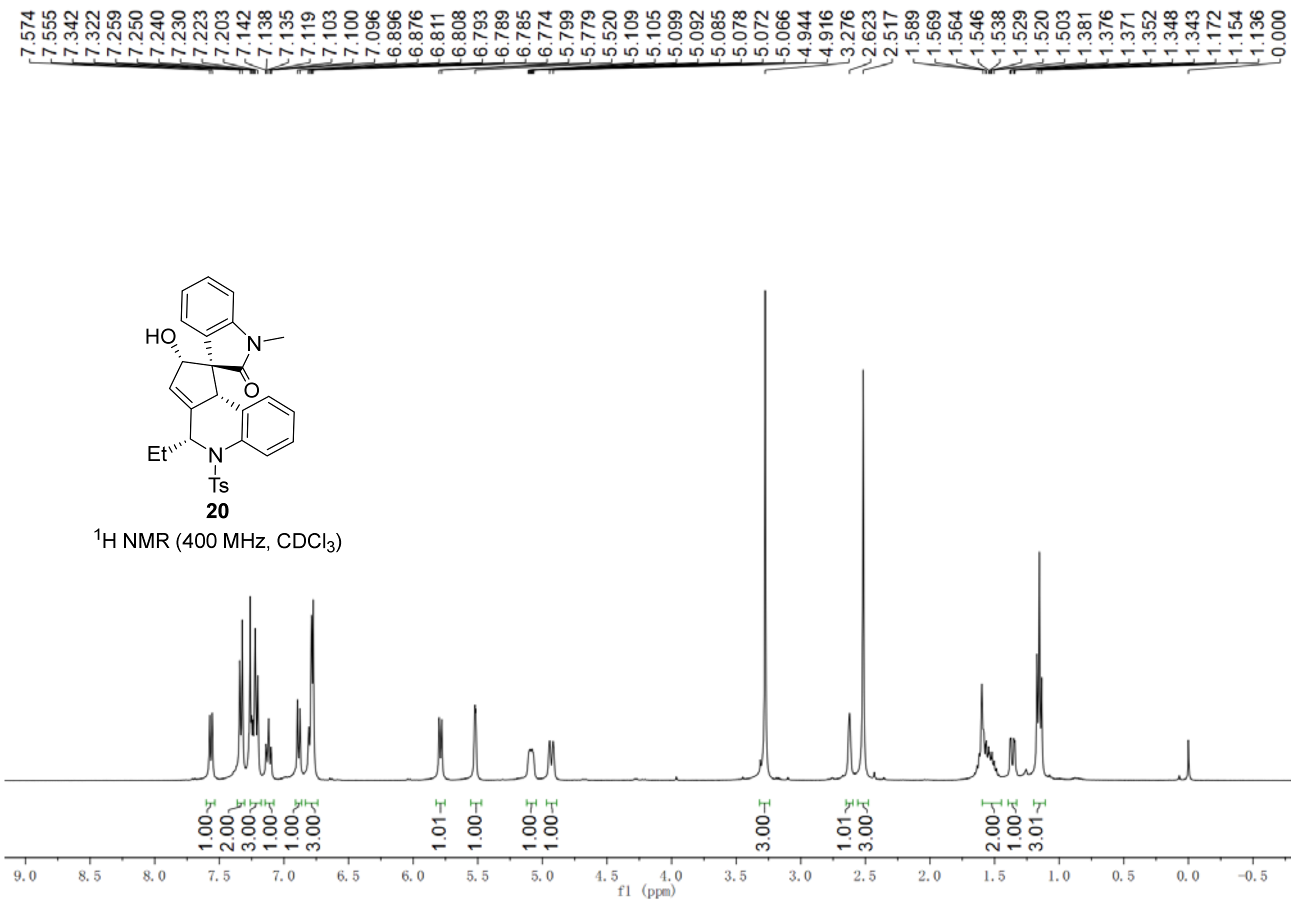
HO
N
O
Et
N
Ts
20
^{1}H NMR (400 MHz, $CDCl_3$)

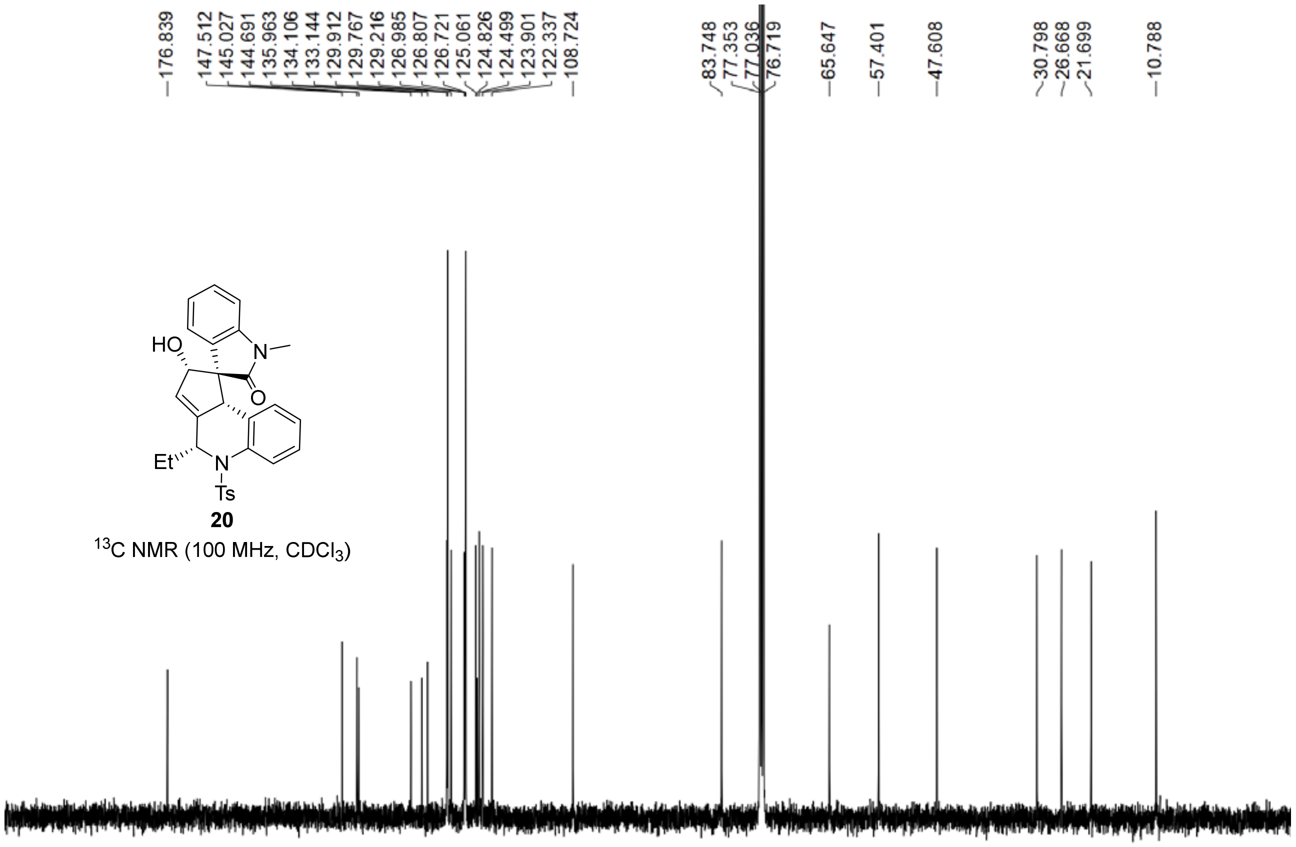
HO
N
O
Et
N
Ts
20
^{13}C NMR (100 MHz, $CDCl_3$)

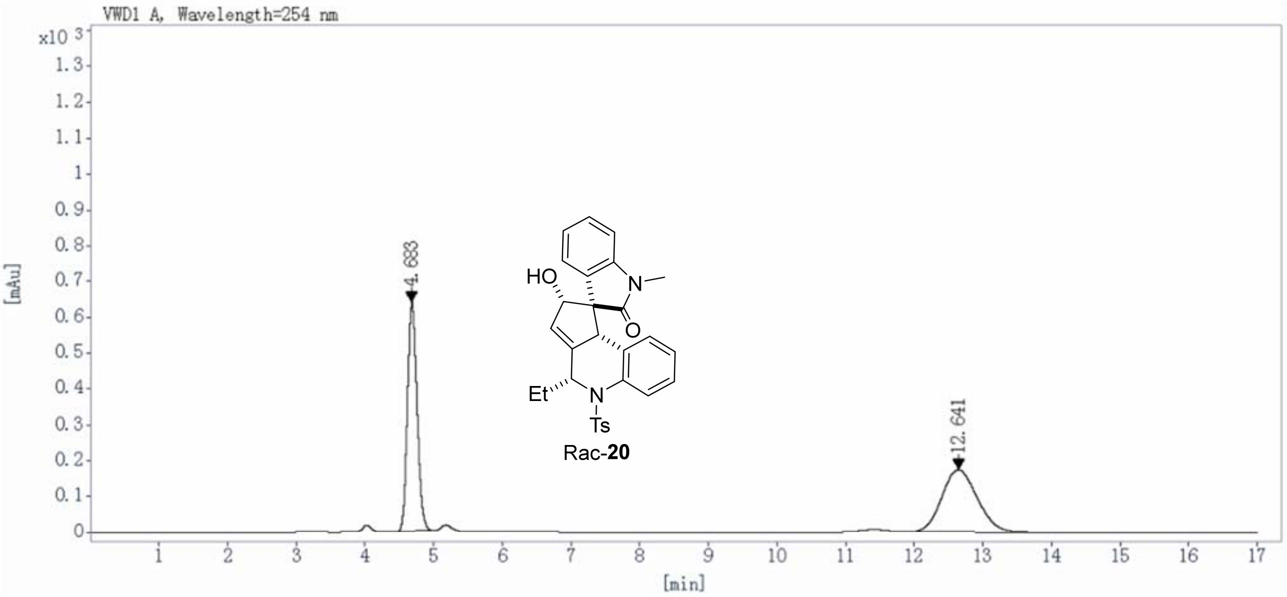

Ret Time [min]	Peak Type	Width [min]	Height [mAU]	Area [mAU*s]	Area [%]
4.683	BBA	0.15	641.1805	6237.6953	49.9388
12.641	BB	0.56	174.5945	6252.9819	50.0612
			Totals:	12490.6772	100.0000

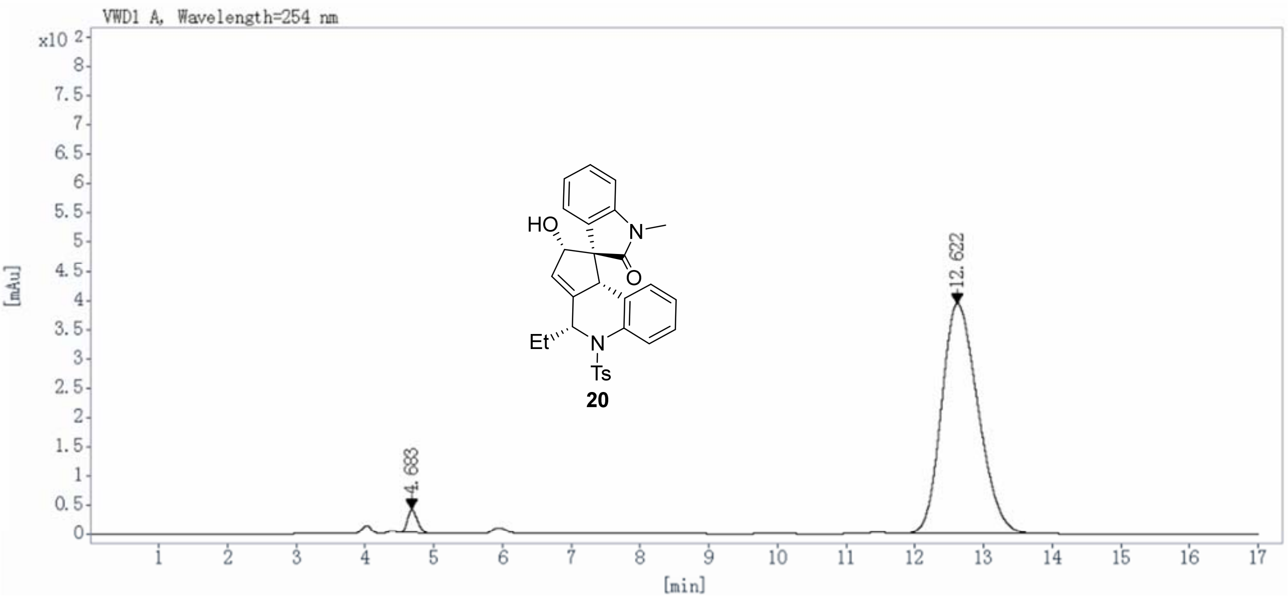

Ret Time [min]	Peak Type	Width [min]	Height [mAU]	Area [mAU*s]	Area [%]
4.683	BB	0.15	39.5163	392.4263	2.6313
12.622	BBA	0.58	393.7119	14521.3232	97.3687
			Totals:	14913.7496	100.0000

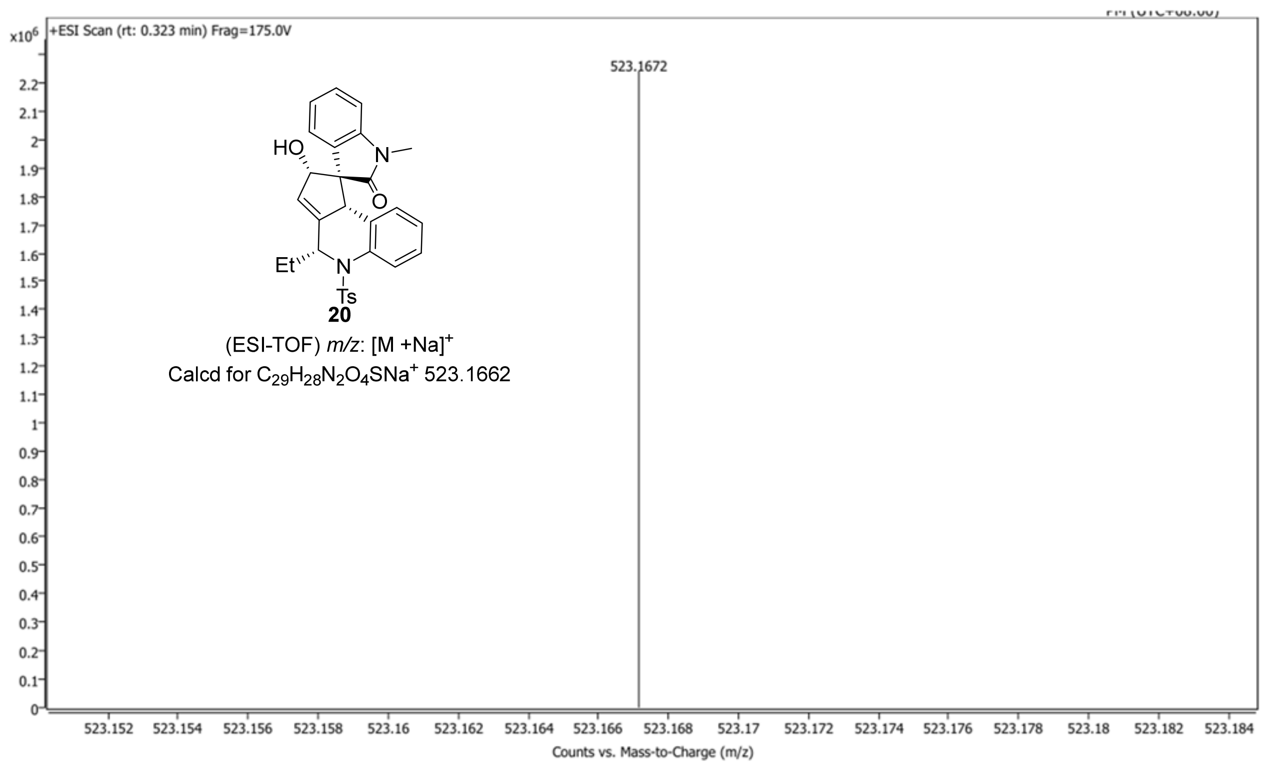

x10^6
+ESI Scan (rt: 0.323 min) Frag=175.0V
523.1672
HO
N
O
Et
N
Ts
20
(ESI-TOF) m/z: [M +Na]+
Calcd for C29H28N2O4SNa+ 523.1662
Counts vs. Mass-to-Charge (m/z)

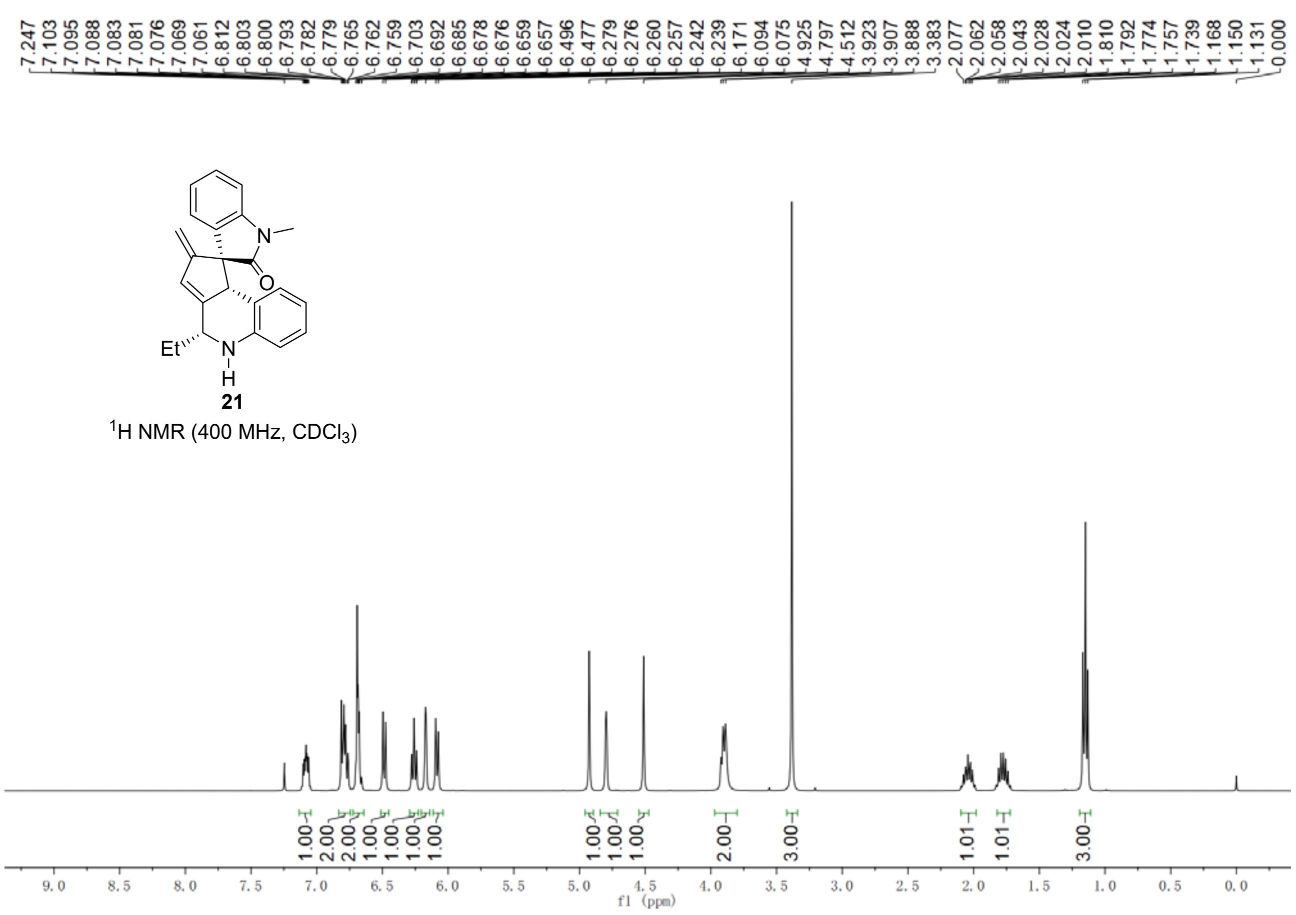
7.247
7.103
7.095
7.088
7.083
7.081
7.076
7.069
7.061
6.812
6.803
6.800
6.793
6.782
6.779
6.765
6.762
6.759
6.703
6.692
6.685
6.678
6.676
6.659
6.657
6.496
6.477
6.279
6.276
6.260
6.257
6.242
6.239
6.171
6.094
6.075
4.925
4.797
4.512
3.923
3.907
3.888
3.383
2.077
2.062
2.058
2.043
2.028
2.024
2.010
1.810
1.792
1.774
1.757
1.739
1.168
1.150
1.131
0.000
N
O
Et
N
H
21
^{1}H NMR (400 MHz, CDCl$_3$)
1.00
2.00
2.00
1.00
1.00
1.00
1.00
1.00
1.00
1.00
2.00
3.00
1.01
1.01
3.00
f1 (ppm)

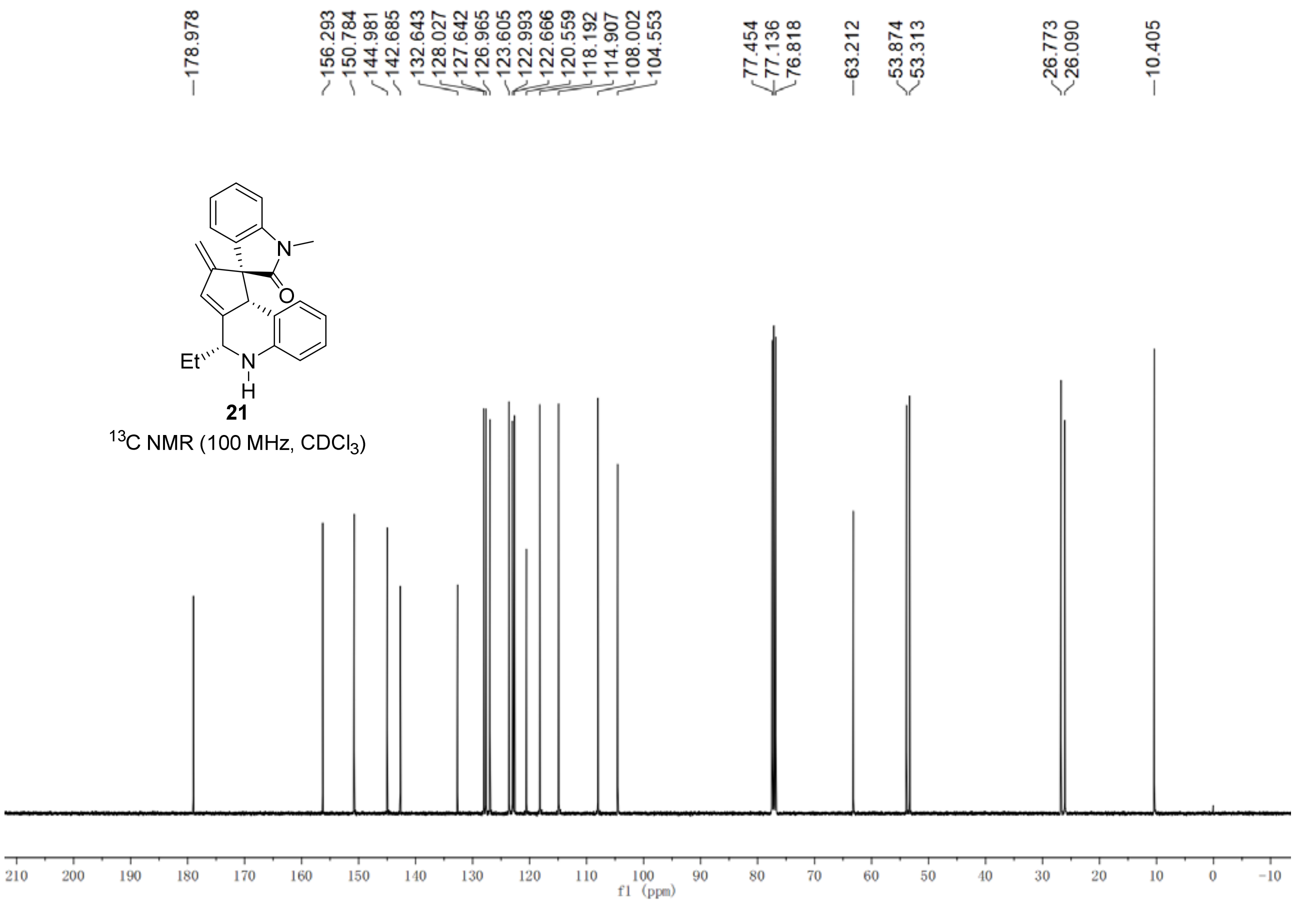
178.978
156.293
150.784
144.981
142.685
132.643
128.027
127.642
126.965
123.605
122.993
122.666
120.559
118.192
114.907
108.002
104.553
77.454
77.136
76.818
63.212
53.874
53.313
26.773
26.090
10.405
N
O
Et
N
H
21
^{13}C NMR (100 MHz, CDCl$_3$)
f1 (ppm)

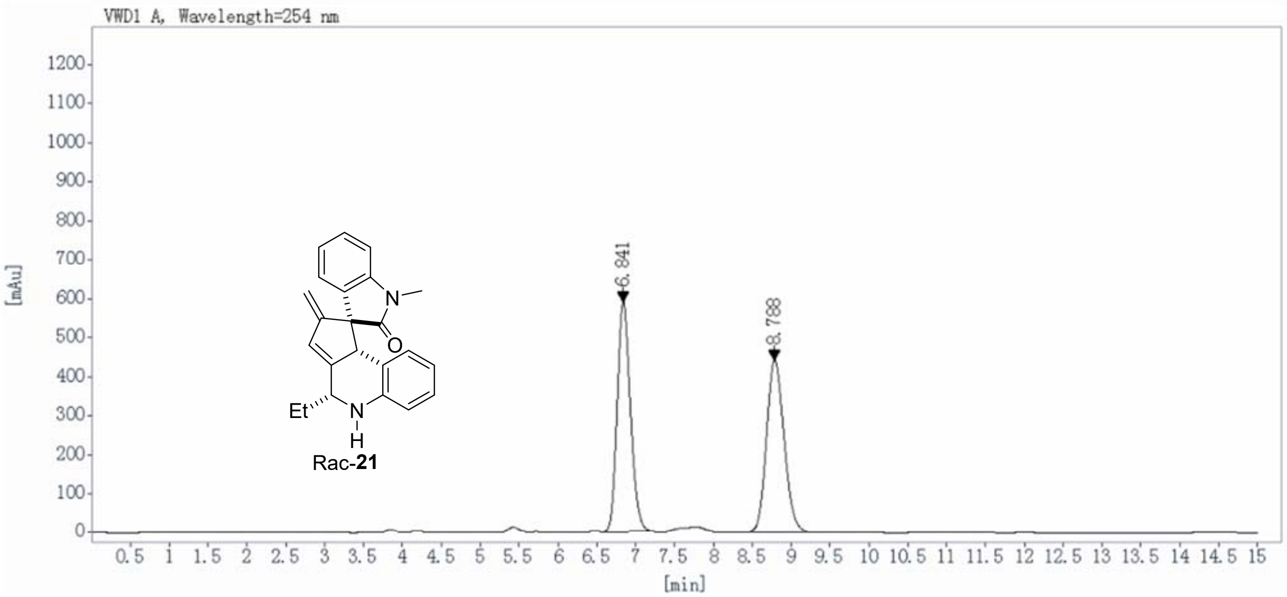

Ret Time [min]	Peak Type	Width [min]	Height [mAU]	Area [mAU*s]	Area [%]
6.841	BBA	0.19	588.1586	7042.9995	50.4276
8.788	BBA	0.24	441.0534	6923.5688	49.5724
			Totals:	13966.5684	100.0000

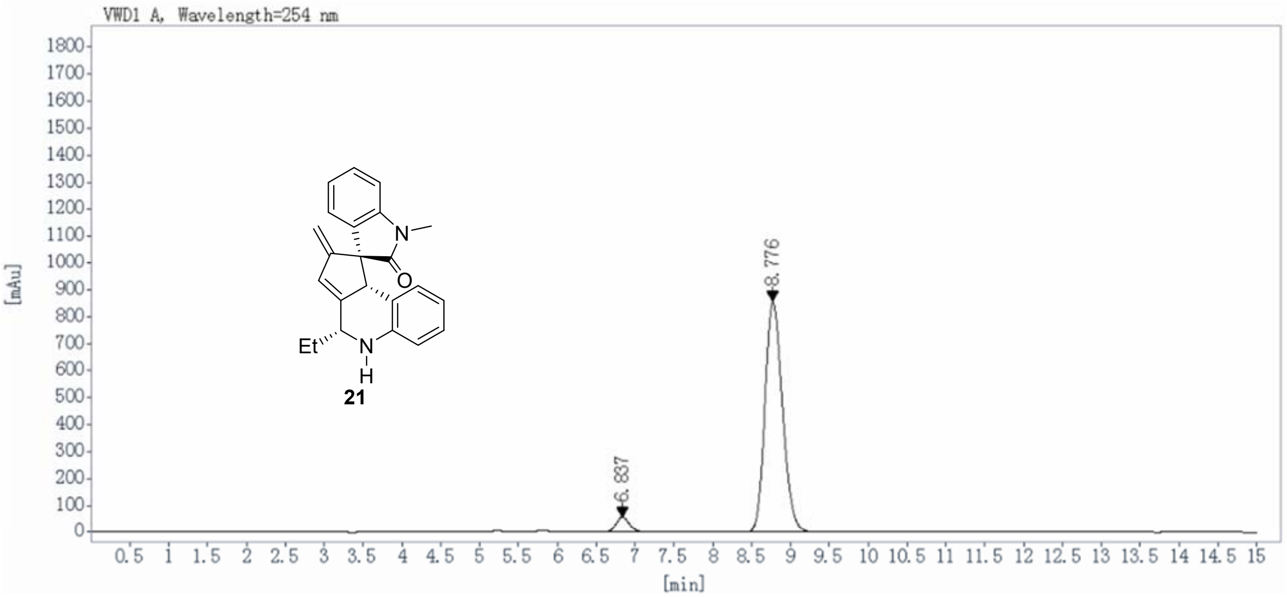

Ret Time [min]	Peak Type	Width [min]	Height [mAU]	Area [mAU*s]	Area [%]
6.837	BB	0.18	53.7135	608.9911	4.3398
8.776	BBA	0.24	854.6207	13423.7334	95.6602
			Totals:	14032.7245	100.0000

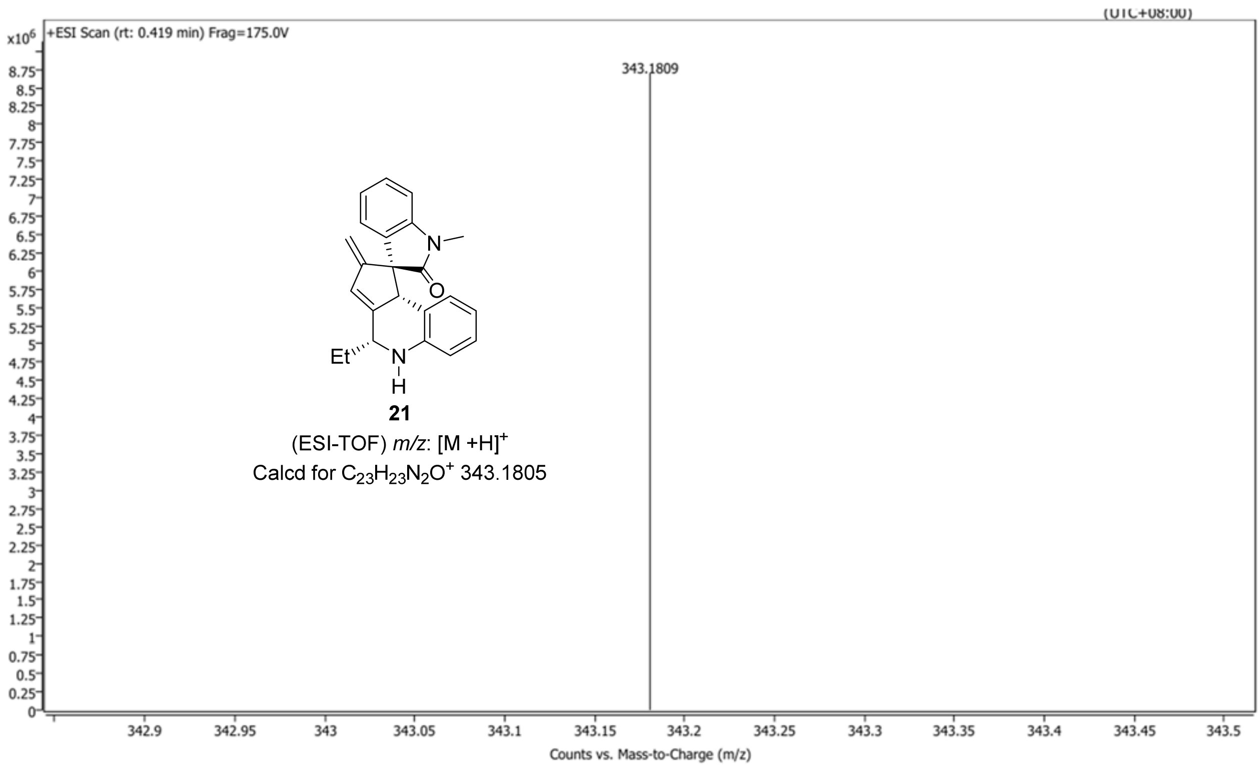
(UTC+08:00)
x10^6
+ESI Scan (rt: 0.419 min) Frag=175.0V
343.1809
21
(ESI-TOF) m/z: [M +H]+
Calcd for C23H23N2O+ 343.1805
Counts vs. Mass-to-Charge (m/z)

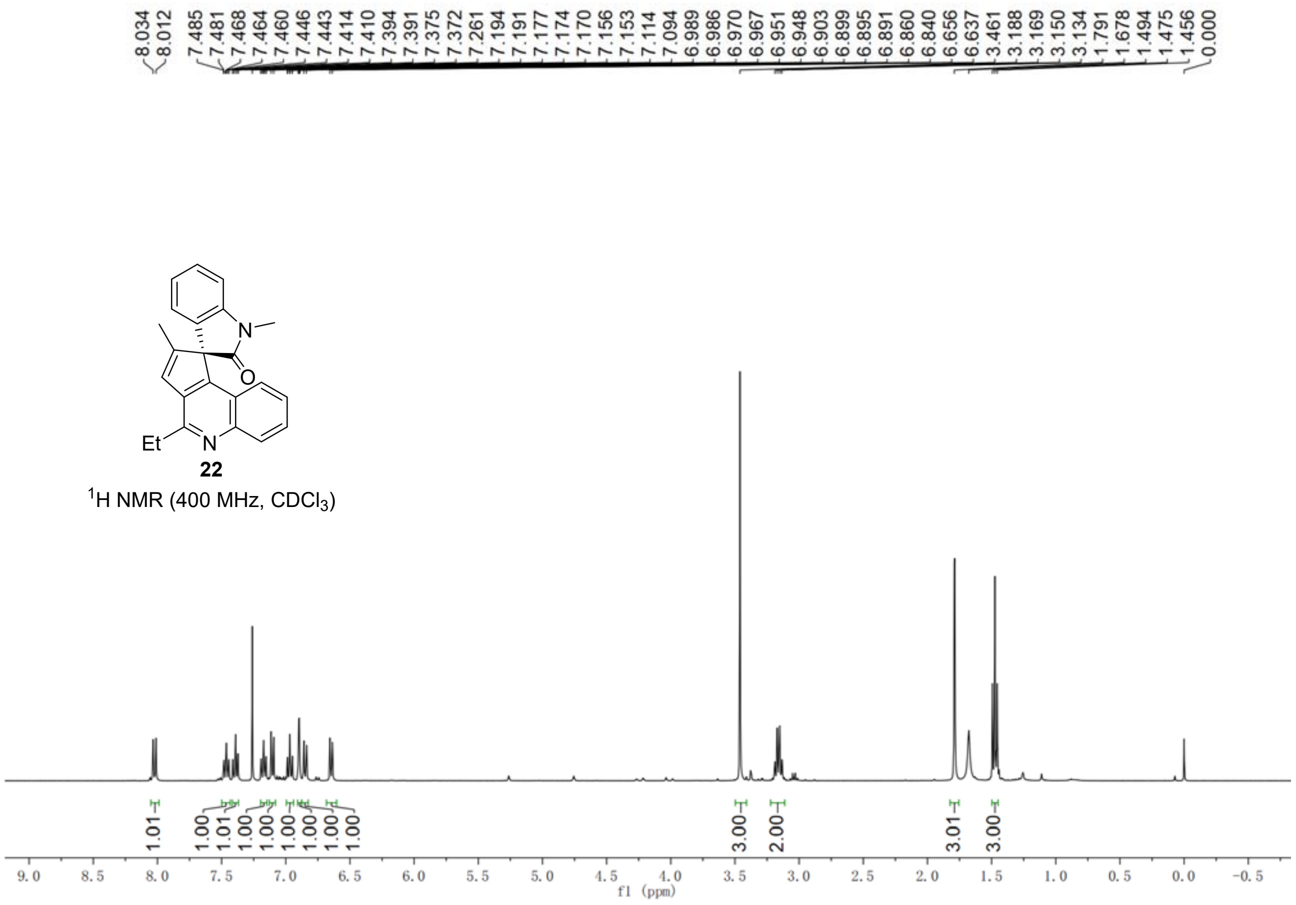

22

^{1}H NMR (400 MHz, $CDCl_3$)

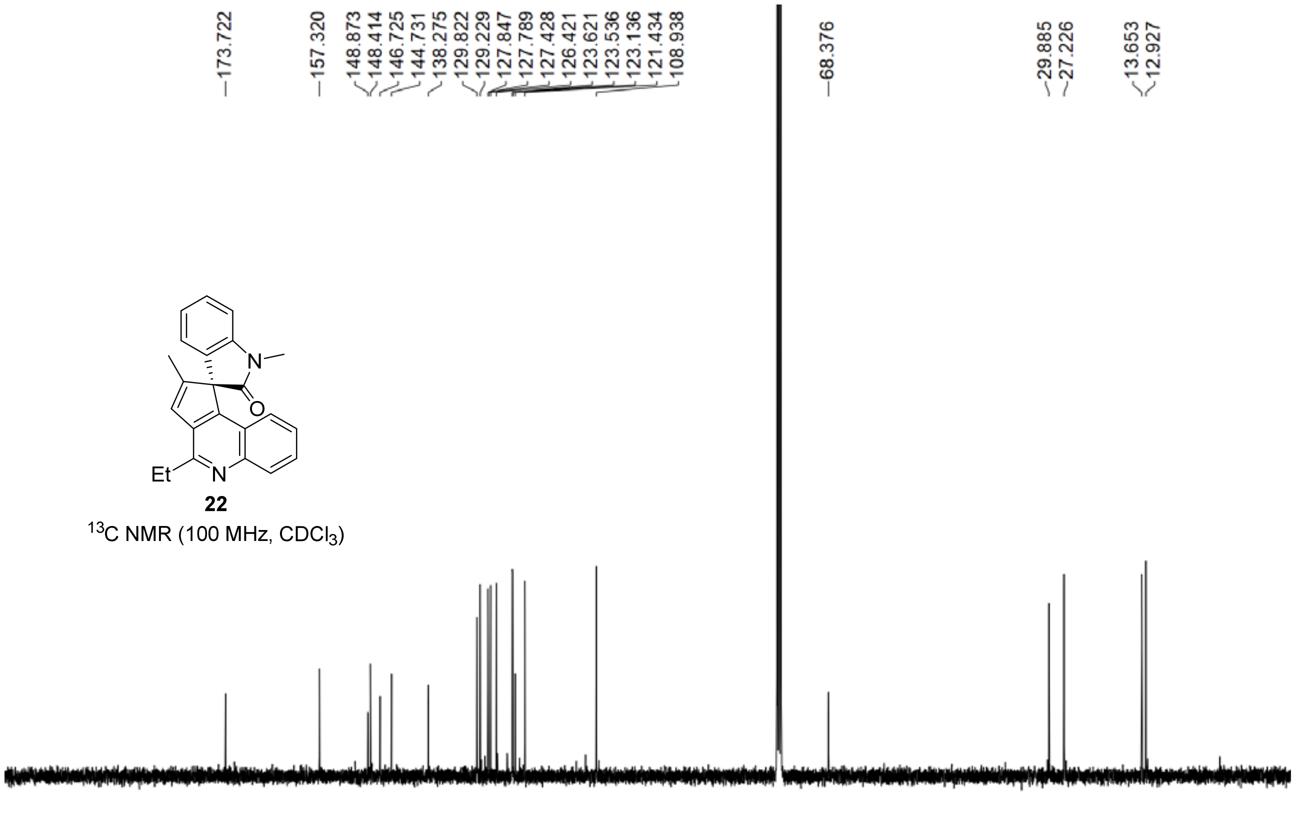

22

^{13}C NMR (100 MHz, $CDCl_3$)

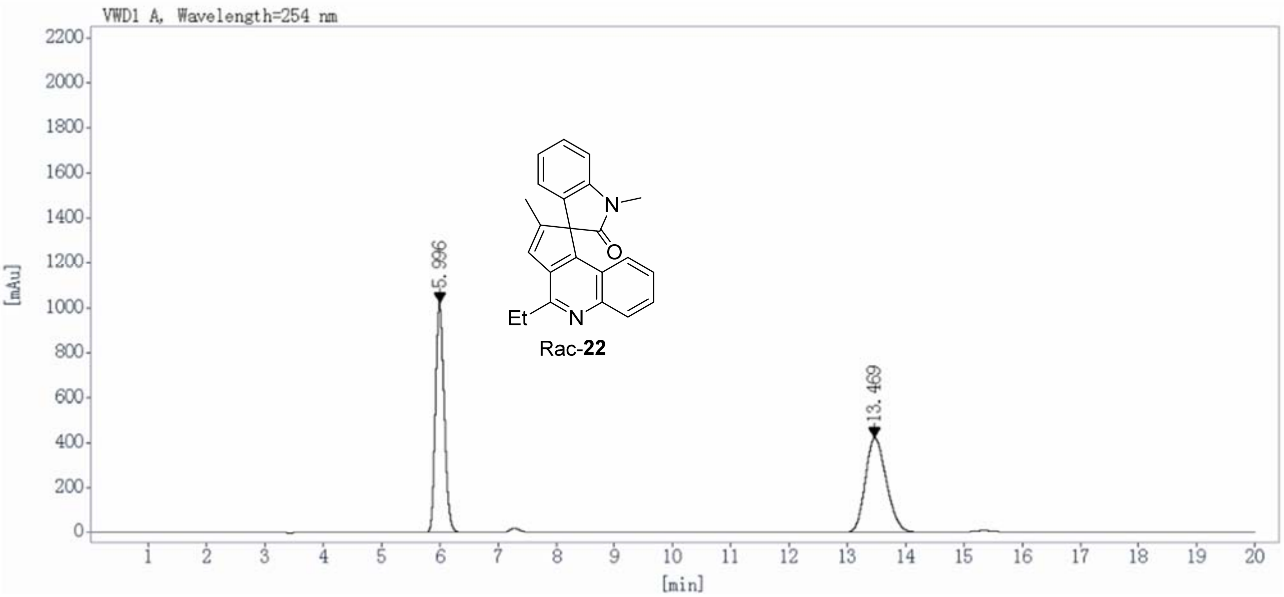

Ret Time [min]	Peak Type	Width [min]	Height [mAU]	Area [mAU*s]	Area [%]
5.996	BBA	0.16	1023.4622	10605.2090	49.9482
13.469	BBA	0.39	422.5282	10627.2080	50.0518
			Totals:	21232.4170	100.0000

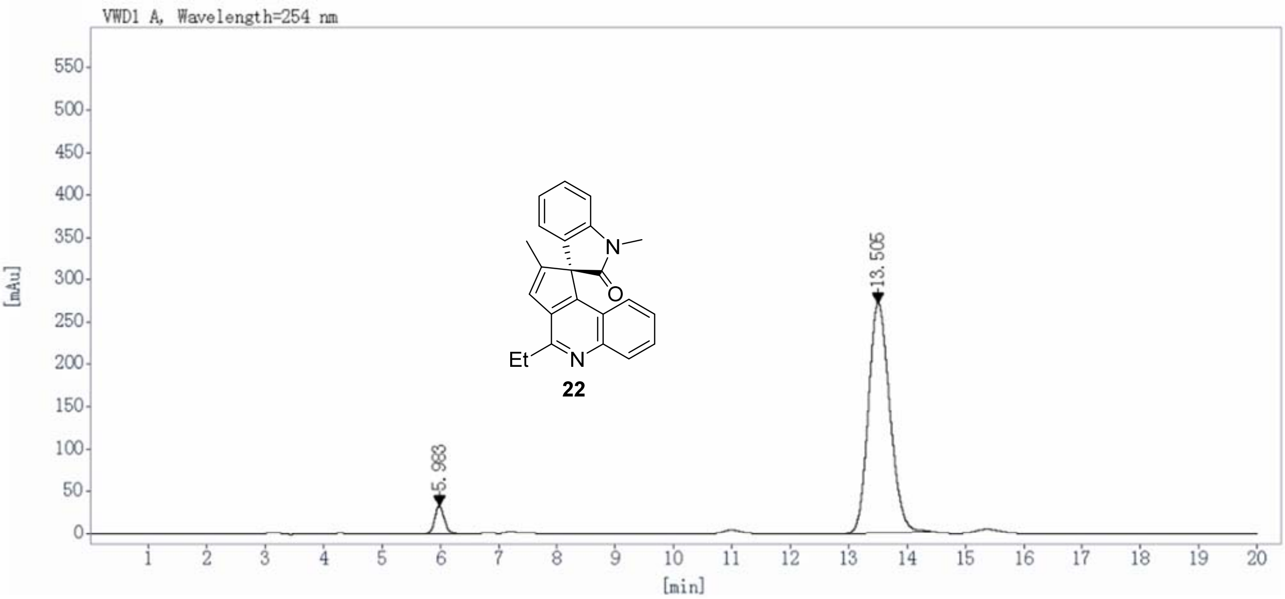

Ret Time [min]	Peak Type	Width [min]	Height [mAU]	Area [mAU*s]	Area [%]
5.983	BBA	0.17	32.8479	365.9035	5.0099
13.505	BBA	0.40	271.3038	6937.6753	94.9901
			Totals:	7303.5788	100.0000

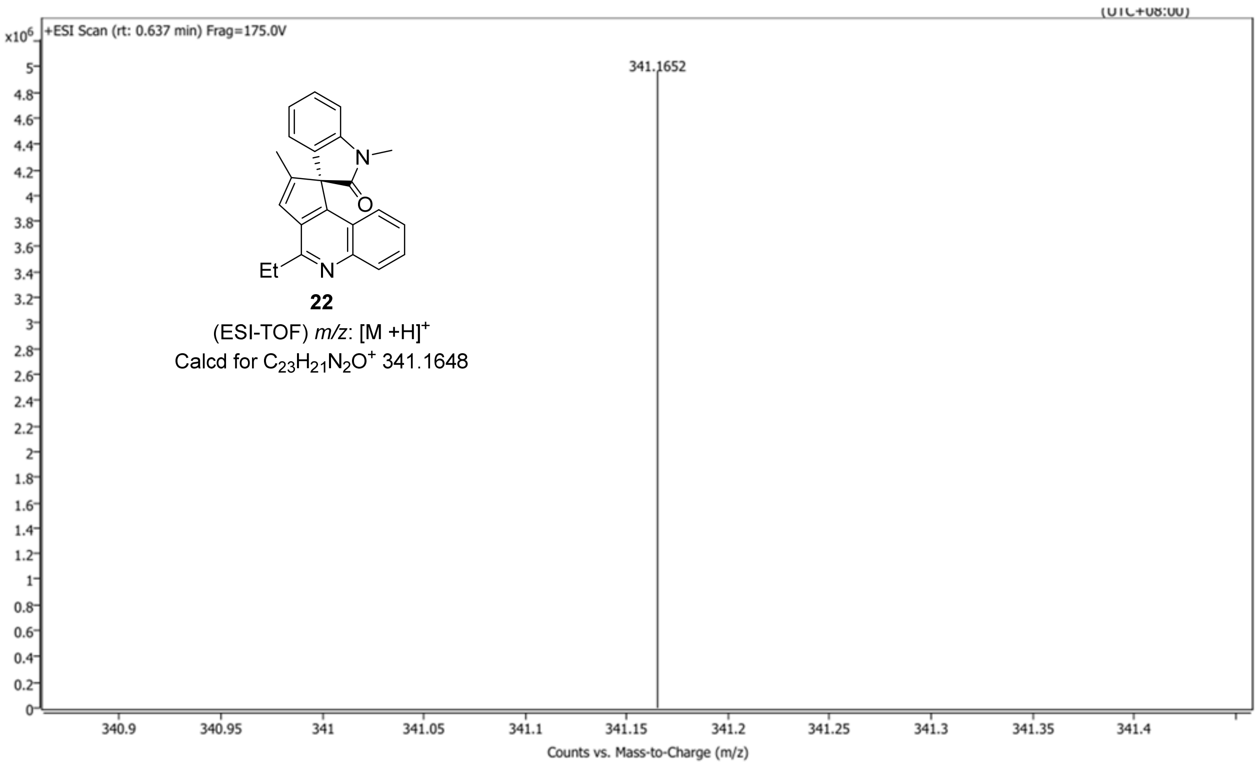
(UTC+08:00)
x10^6
+ESI Scan (rt: 0.637 min) Frag=175.0V
341.1652
N
O
Et
N
22
(ESI-TOF) m/z: [M +H]+
Calcd for C23H21N2O+ 341.1648
5
4.8
4.6
4.4
4.2
4
3.8
3.6
3.4
3.2
3
2.8
2.6
2.4
2.2
2
1.8
1.6
1.4
1.2
1
0.8
0.6
0.4
0.2
0
340.9
340.95
341
341.05
341.1
341.15
341.2
341.25
341.3
341.35
341.4
Counts vs. Mass-to-Charge (m/z)

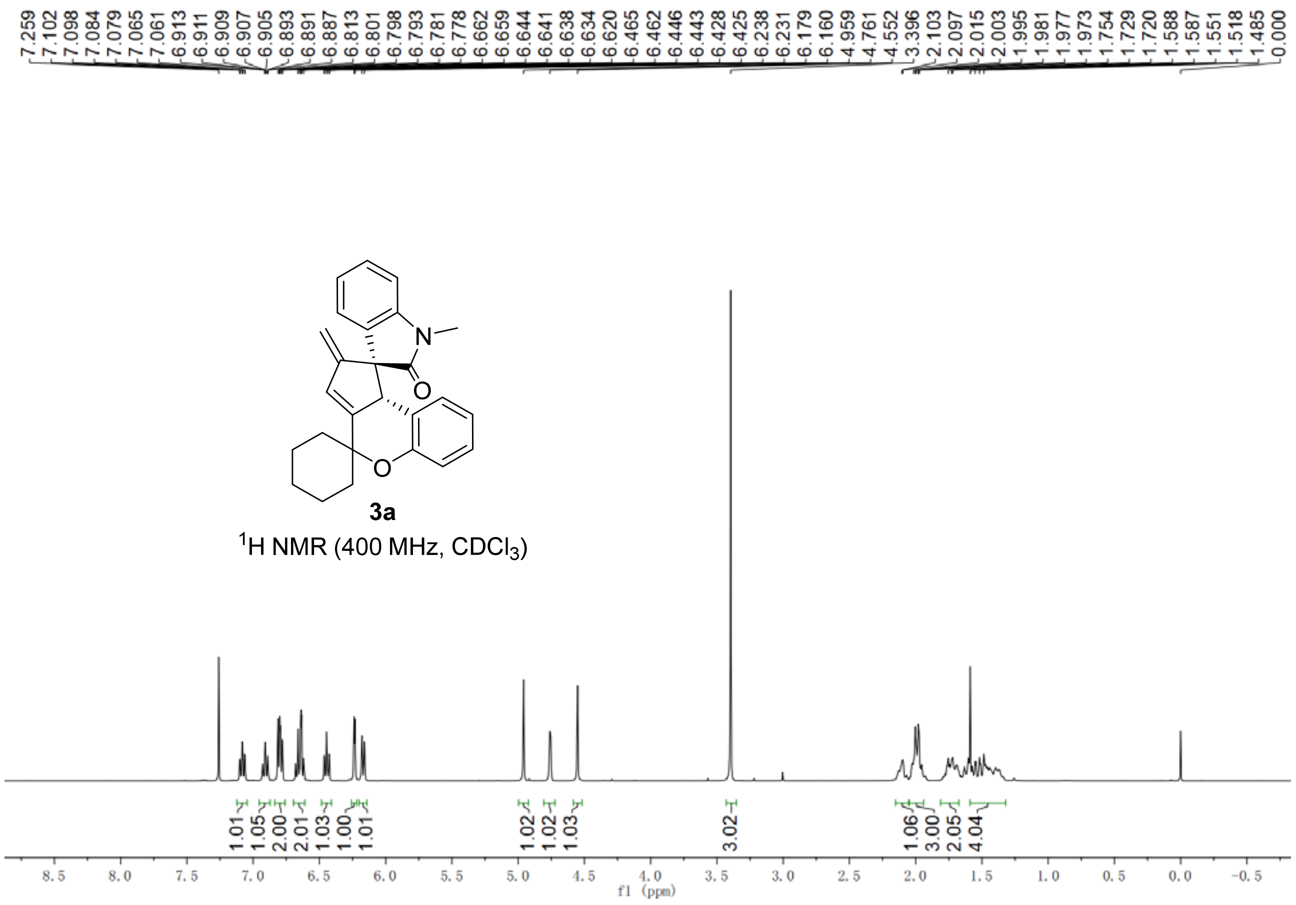
7.259 7.102 7.098 7.084 7.079 7.065 7.061 6.913 6.911 6.909 6.907 6.905 6.893 6.891 6.887 6.813 6.801 6.798 6.793 6.781 6.778 6.662 6.659 6.644 6.641 6.638 6.634 6.620 6.465 6.462 6.446 6.443 6.428 6.425 6.238 6.231 6.179 6.160 4.959 4.761 4.552 3.396 2.103 2.097 2.015 2.003 1.995 1.981 1.977 1.973 1.754 1.729 1.720 1.588 1.587 1.551 1.518 1.485 0.000
N
O
O
3a
1H NMR (400 MHz, CDCl3)
1.01 1.05 2.00 2.01 1.03 1.00 1.01 1.02 1.02 1.03 3.02 1.06 3.00 2.05 4.04
fl (ppm)

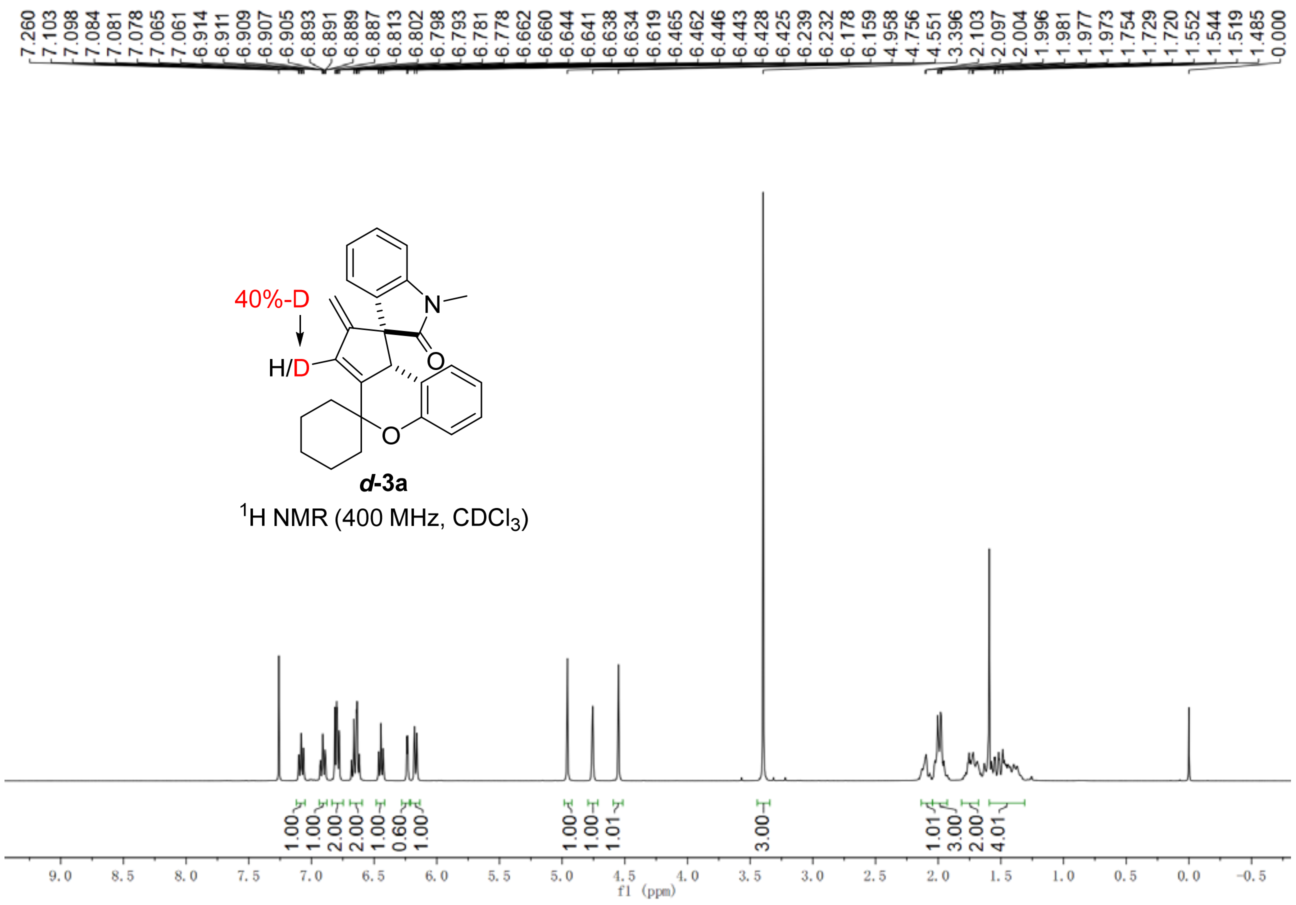
7.260 7.103 7.098 7.084 7.081 7.078 7.065 7.061 6.914 6.911 6.909 6.907 6.905 6.893 6.891 6.889 6.887 6.813 6.802 6.798 6.793 6.781 6.778 6.662 6.660 6.644 6.641 6.638 6.634 6.619 6.465 6.462 6.446 6.443 6.428 6.425 6.239 6.232 6.178 6.159 4.958 4.756 4.551 3.396 2.103 2.097 2.004 1.996 1.981 1.977 1.973 1.754 1.729 1.720 1.552 1.544 1.519 1.485 0.000
40%-D
H/D
N
O
O
d-3a
1H NMR (400 MHz, CDCl3)
1.00 1.00 2.00 2.00 1.00 0.60 1.00 1.00 1.00 1.01 3.00 1.01 3.00 2.00 4.01
fl (ppm)

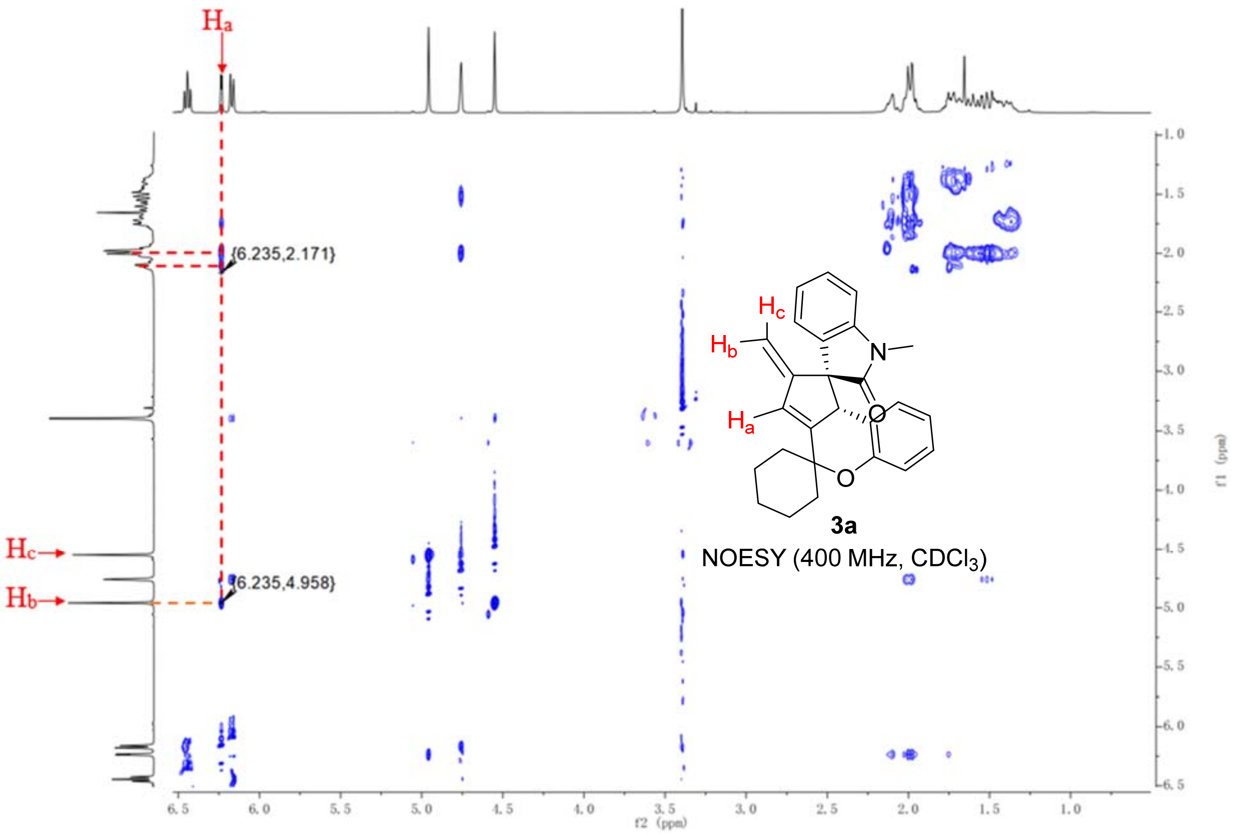

3a

NOESY (400 MHz, $CDCl_3$)
